# Genome-Wide Study of the Tomato *SlMLO* Gene Family and Its Functional Characterization in Response to the Powdery Mildew Fungus *Oidium neolycopersici*

**DOI:** 10.3389/fpls.2016.00380

**Published:** 2016-04-06

**Authors:** Zheng Zheng, Michela Appiano, Stefano Pavan, Valentina Bracuto, Luigi Ricciardi, Richard G. F. Visser, Anne-Marie A. Wolters, Yuling Bai

**Affiliations:** ^1^Institute of Vegetables and Flowers, Chinese Academy of Agricultural SciencesBeijing, China; ^2^Wageningen UR Plant Breeding, Wageningen University and Research CentreWageningen, Netherlands; ^3^Section of Genetics and Plant Breeding, Department of Plant, Soil and Food Science, University of Bari Aldo MoroBari, Italy

**Keywords:** *MLO* gene family, tomato, susceptibility, powdery mildew disease

## Abstract

The *MLO* (*Mildew Locus O*) gene family encodes plant-specific proteins containing seven transmembrane domains and likely acting in signal transduction in a calcium and calmodulin dependent manner. Some members of the *MLO* family are susceptibility factors toward fungi causing the powdery mildew disease. In tomato, for example, the loss-of-function of the *MLO* gene *SlMLO1* leads to a particular form of powdery mildew resistance, called *ol-2*, which arrests almost completely fungal penetration. This type of penetration resistance is characterized by the apposition of papillae at the sites of plant-pathogen interaction. Other *MLO* homologs in Arabidopsis regulate root response to mechanical stimuli (*AtMLO4* and *AtMLO11*) and pollen tube reception by the female gametophyte (*AtMLO7*). However, the role of most *MLO* genes remains unknown. In this work, we provide a genome-wide study of the tomato *SlMLO* gene family. Besides *SlMLO1*, other 15 *SlMLO* homologs were identified and characterized with respect to their structure, genomic organization, phylogenetic relationship, and expression profile. In addition, by analysis of transgenic plants, we demonstrated that simultaneous silencing of *SlMLO1* and two of its closely related homologs, *SlMLO5* and *SlMLO8*, confer higher level of resistance than the one associated with the *ol-2* mutation. The outcome of this study provides evidence for functional redundancy among tomato homolog genes involved in powdery mildew susceptibility. Moreover, we developed a series of transgenic lines silenced for individual *SlMLO* homologs, which lay the foundation for further investigations aimed at assigning new biological functions to the *MLO* gene family.

## Introduction

Many important crop species can be affected by the powdery mildew (PM) disease, resulting in great yield losses in agricultural settings. In barley, recessive loss-of-function mutations occurring in the *HvMLO* (*Hordeum vulgare Mildew Resistance Locus O*) gene confer resistance to all known isolates of the PM fungus *Blumeria graminis* f.sp *hordei*. Therefore, natural or induced *mlo*-mutant alleles are in use for about seven decades to introduce resistance in spring barley breeding programs (Jørgensen, 1992; Büschges et al., 1997; Reinstädler et al., 2010).

Biochemical analysis showed that the barley HvMLO protein contains seven transmembrane domains integral to the plasma membrane, with an extracellular amino-terminus and an intracellular carboxy-terminus. The latter harbors a calmodulin-binding domain likely involved in sensing calcium influxes into cells (Devoto et al., 1999). Although the domain structure of MLO proteins is related to that of metazoan G-protein coupled receptors (GPCRs), several studies could not confirm the role of MLO proteins as canonical GPCRs (Kim et al., 2002; Lorek et al., 2013). Despite further intensive efforts to explain the biochemical function of the HvMLO protein, its core activity remains elusive (Panstruga, 2005). However, HvMLO might be exploited by the fungus to impair vesicle-associated defense mechanism at plant-pathogen interaction sites, thus facilitating its penetration (Panstruga and Schulze-Lefert, 2003; Opalski et al., 2005; Miklis et al., 2007). This feature makes *HvMLO* a typical representative of susceptibility genes (*S*-genes) (Miklis et al., 2007; van Schie and Takken, 2014).

The robustness of barley *mlo*-resistance, due to its non-race-specific spectrum and durability, led in the last years to an extensive quest for identification and functional characterization of the *MLO* genes in other species affected by the PM disease. The search resulted in the identification of multiple *MLO* gene families, ranging from 12 to 39 members in Arabidopsis, rice, grapevine, cucumber, apple, peach, woodland strawberry, tobacco, and soybean (Devoto et al., 2003; Feechan et al., 2008; Liu and Zhu, 2008; Shen et al., 2012; Zhou et al., 2013; Pessina et al., 2014; Appiano et al., 2015). Moreover, specific homologs were shown to play a major role in plant-pathogen interactions (Consonni et al., 2006).

A detailed phylogenetic analysis distinguished up to eight clades in which Angiosperm MLO proteins can be found (Feechan et al., 2008; Acevedo-Garcia et al., 2014; Pessina et al., 2014). The MLO homologs involved in the interaction with PM pathogens (Arabidopsis AtMLO2, AtMLO6, AtMLO12, tomato SlMLO1, pea Er1/PsMLO1, grapevine VvMLO3 and VvMLO4, tobacco NtMLO1, pepper CaMLO2, cucumber CsaMLO8, *Lotus japonicus* LjMLO1, and barrel clover MtMLO1) are grouped into clade V. On the other hand, all the known monocot MLO homologs acting as susceptibility factors (barley HvMLO, rice OsMLO3, and wheat TaMLO_A1 and TaMLO_B1) do not cluster in clade V, but in clade IV, which is primarily but not exclusively represented by monocot MLO proteins. For example, grapevine VvMLO14, strawberry FvMLO17, and peach PpMLO12 belong also to clade IV (Elliott et al., 2002; Feechan et al., 2008; Acevedo-Garcia et al., 2014; Pessina et al., 2014).

In Arabidopsis, the PM resistance conferred by the loss-of-function of *AtMLO2* is incomplete and only mutations in all the three *AtMLO* homologs in clade V can completely prevent fungal entry (Consonni et al., 2006). In addition, more recent studies in Arabidopsis indicated that other members of the *MLO* gene family play a role in different biological processes. The homologs *AtMLO4* and *AtMLO11* are together involved in root thigmomorphogenesis, i.e., root responses to mechanical stimuli (Chen et al., 2009), while *AtMLO7* regulates pollen tube reception from the synergid cells during fertilization (Kessler et al., 2010). The biological roles of other *MLO* homologs still remain elusive.

Tomato (*Solanum lycopersicum*) is one of the most economically important vegetables in the world. It can be host of three PM species, namely *Oidium neolycopersici, Oidium lycopersici*, and *Leveillula taurica* (Seifi et al., 2014). Since 1996, when it was found that all the tomato cultivars were susceptible to *O. neolycopersici*, extensive researches were conducted by our group for sources of resistance (Seifi et al., 2014). An allele containing a 19 bp deletion in the coding region of the PM susceptibility gene *SlMLO1* was found in a wild accession of *S. lycopersicum* var. *cerasiforme*. This mutant allele, named *ol-2*, was shown to confer recessively inherited broad-spectrum resistance to a series of isolates of *O. neolycopersici* (Bai et al., 2005, 2008). Through histological analysis, it was shown that its mechanism of resistance is based on the early abortion of fungal pathogenesis at the sites of attempted penetration (Bai et al., 2005). This type of penetration resistance is characterized by papillae apposition, the same as described also for the PM resistance in the *Atmlo2* mutant of Arabidopsis (Consonni et al., 2006). Although papilla formation can significantly reduce fungal development at the host cell entry level, fungal penetration was not fully prevented in the *ol-2* mutant (Bai et al., 2005).

In this study, we exploited tomato sequence information, derived from the tomato genome sequencing Heinz 1706 and the 150 tomato genome resequencing projects (Tomato Genome Consortium, 2012; The 100 Tomato Genome Sequencing Consortium et al., 2014), in order to identify tomato *MLO* homologs (*SlMLO*). These were characterized with respect to (1) their genomic organization, (2) relation with *MLO* homologs from other species, (3) occurrence of tissue-specific differentially spliced variants, (4) expression in different tissues in axenic condition and (5) upon inoculation with the powdery mildew pathogen *O. neolycopersici*. Finally, an RNAi-based reverse genetic approach was followed to investigate the possibility that *SlMLO* homologs other than *SlMLO1* could play additional roles in the interaction with *O. neolycopersici*.

## Results

### *In silico* identification and sequencing of the tomato *SlMLO* gene family

A total of 17 tomato *MLO-*like loci were identified through BLAST interrogation of the tomato genomic sequence database (SGN), using AtMLO protein sequences as query. Two of them (referred to as Solyc09g18830 and Solyc09g18840 in the SGN database) were noticeably shorter than other predicted *MLO* homologs and physically close to each other, suggesting they are different parts of the same gene (Table 1). Search in the tomato EST database and gene prediction analysis in the *S. pimpinellifolium* genome with the FGENESH software allowed identifying a hypothetical full-length *MLO* transcript encompassing Solyc09g18830 and Solyc09g18840. PCR from leaf of the tomato cultivar Moneymaker (MM) confirmed the presence of this transcript, which was named *SlMLO7* (Supplementary Figure 1). The other 15 predicted *SlMLO* genes were named from *SlMLO1* to *SlMLO6*, and from *SlMLO8* to *SlMLO16*, as reported in Table 1. For all of them, information is available with respect to putative amino acid length and number of introns.

**Table 1:** **Features of the ***SlMLO*** gene family as inferred by the Sol Genomics Network Database**.

**SGN locus name**	***MLO* gene**	**Chromosome**	**Position**	**ORF lenght (aa)**	**Introns**
Solyc04g049090	*SlMLO1*	4	SL2.40ch04:38700445.38705951	507	14
Solyc08g015870	*SlMLO2*	8	SL2.40ch08:6074040.6078983	504	13
Solyc06g010030	*SlMLO3*	6	SL2.40ch06:4786764.4792828	591	14
Solyc00g007200	*SlMLO4*	2?	SL2.40ch00:6816892.6823417	554	14
Solyc03g095650	*SlMLO5*	3	SL2.40ch03:50279919.50288063	517	14
Solyc02g082430	*SlMLO6*	2	SL2.40ch02:40694608.40700995	553	14
Solyc09g018830 Solyc09g018840	*SlMLO7*	9	SL2.40ch09:17564555.17568214	270	10
Solyc11g069220	*SlMLO8*	11	SL2.40ch11:50939533.50946726	506	13
Solyc06g082820	*SlMLO9*	6	SL2.40ch06:44779673.44784035	511	13
Solyc02g083720	*SlMLO10*	2	SL2.40ch02:41596474.41602413	533	14
Solyc01g102520	*SlMLO11*	1	SL2.40ch01:83071860.83075439	475	13
Solyc08g067760	*SlMLO12*	8	SL2.40ch08:53957062.53962884	532	14
Solyc10g044510	*SlMLO13*	10	SL2.40ch10:22128868.22135940	558	14
Solyc07g063260	*SlMLO14*	7	SL2.40ch07:62995345.63002900	563	14
Solyc02g077570	*SlMLO15*	2	SL2.40ch02:37045094.37050486	375	10
Solyc06g010010	*SlMLO16*	6	SL2.40ch06:4699552.4706571	477	14

With the exception of *SlMLO4*, information on chromosomal localization could also be inferred (Table 1). Most *SlMLO* homologs are scattered throughout the tomato genome, thus suggesting that segmental duplication events have been a major source for the evolution of the *SlMLO* gene family. Exceptions are represented by two physical gene clusters, one containing *SlMLO6, SlMLO10*, and *SlMLO15* on chromosome 2, and the other containing *SlMLO3* and *SlMLO16* on chromosome 6.

Sequence and expression of all the predicted *SlMLO* homologs were verified by PCR amplification of cDNAs derived from four different tissues (leaf, root, flower, and ripened fruit) of MM. All the *SlMLO* homologs could be amplified at least from one plant tissue. In total, 15 *SlMLO* homologs could be cloned from leaf (with the exception of *SlMLO12*), 10 from flower, nine from fruit and eight from root (Supplementary Table 1).

Sequence alignment of cloned *SlMLO* transcripts with corresponding SGN predicted coding sequence (CDS), derived from the cultivar Heinz 1706, revealed polymorphisms for *SlMLO7, SlMLO8, SlMLO10*, and *SlMLO15* (Supplementary Figure 1). The 1339 bp *SlMLO7* cloned transcript corresponds to a short open reading frame (ORF) due to a stop codon at 137–139 bp (Supplementary Figure 1). The SGN predicted CDS of *SlMLO8* misses part of the third, seventh, eighth, and ninth exon present in the corresponding transcript cloned from MM leaf; compared to the SGN predicted CDS of *SlMLO10*, the transcript cloned from MM fruit contains a base change at the beginning of the fifth exon, which results in a stop codon (Supplementary Figure 1). Also the predicted ORF of *SlMLO15* is shorter (375 aa) than the average ORF length of other SlMLOs (Table 1). The sequence cloned from MM leaf has a longer ORF (459 aa) compared to the predicted SGN sequence (**Table 3A**).

In other cases, sequence alignments of cloned *SlMLO* from the different tissues with their corresponding genomic regions showed various types of splice variants, consisting of intron retention, exon skipping and alternative 5′ and 3′ splice sites, according to the types of alternative splicing described by Keren et al. (2010) (Table 2 and Supplementary Figure 1).

**Table 2:** **Types of differentially spliced events observed in cloned ***SlMLO*** homologs from different tissues of the tomato cv. Moneymaker**.

***SlMLO***	**Plant tissue**	**Type of alternative splicing**			
		**Intron retention**	**Exon skipping**	**Alternative 5′ splice site**	**Alternative 3′ splice site**
*SlMLO1*	Flower				√
SlMLO5^*^	Fruit	√			
*SlMLO6*	Leaf			√	√
*SlMLO9*	Leaf		√		
SlMLO11^*^	Root	√			
*SlMLO13*	Leaf		√		√
*SlMLO15*	Fruit		√	√	
SlMLO15^*^	Root	√	√	√	
SlMLO15^*^	Flower	√	√	√	

The asterisk (^*^) indicates SlMLO transcripts that can be either incompletely spliced or alternatively spliced.

### Characterization of conserved amino acids and motifs of the SlMLO proteins

To examine sequence features of the tomato SlMLO proteins, a multiple sequence alignment was performed using sequences obtained by the conceptual translation of transcripts cloned in different tissues. When no deviating transcripts were observed for a *SlMLO* gene, the sequence obtained from leaf was used for translation, with the exception of *SlMLO12* which is the only homolog that was not cloned from leaf but from flower.

The aligned amino acid sequences of the tomato SlMLO protein family showed a high degree of conservation (92%) of the 30 amino acid residues previously described to be invariable throughout the whole MLO protein family (Supplementary Figure 2; Elliott et al., 2005).

Due to aberrant transcripts, the protein sequences of SlMLO7 and SlMLO13 in leaf, SlMLO11 in root, and SlMLO15 in root, flower and fruit, were severely truncated (Table 3A). The predicted ORF of SlMLO8 in leaf was longer than the one deriving from the SGN prediction, which is missing important domains of the translated MLO protein. The protein sequence of *SlMLO9* in leaf was shorter (448 aa length) than the ones obtained from the other two tissues (512 aa length) and it is predicted to have five transmembrane (TM) domains, instead of seven as in fruit and flower (Table 3A).

**Table 3A:** **Features and motifs distribution occurring in SlMLO proteins obtained from ***in silico*** translation of leaf, root, flower, and fruit transcripts of the tomato cv. Moneymaker**.

		**ORF Length (aa)**	**MOTIF 1**	**MOTIF 2**	**MOTIF 3**	**MOTIF4**	**MOTIF 5**	**MOTIF 6**	**MOTIF 7**	**MOTIF 8**	**MOTIF 9**	**MOTIF10**
SlMLO1	Leaf	507	√	√	√	√	√	√				
	Root	507	√	√	√	√	√	√				
	Flower	491	√	√	√	√	√					
SlMLO2	Leaf	504	√	√	√	√	√					
SlMLO3	Leaf	591	√			√		√				
SlMLO4	Leaf	554	√	√	√	√		√	√	√		
SlMLO5	Leaf	517	√	√	√	√		√				
	Flower	517	√	√	√	√		√				
	Fruit	540	√	√	√	√		√				
SlMLO6	Leaf	549	√	√	√	√			√	√		
	Root	553	√	√	√	√		√	√	√		
	Flower	553	√	√		√		√	√	√		
	Fruit	553	√	√	√	√		√	√	√		
SlMLO7	Leaf	61										
SlMLO8	Leaf	561	√	√	√	√		√				
SlMLO9	Leaf	448	√		√	√	√	√		√		
	Flower	511	√	√	√	√	√	√		√		
	Fruit	511	√	√	√	√	√	√		√		
SlMLO10	Leaf	533	√	√	√	√	√	√			√	
	Root	533	√	√	√	√	√	√			√	
	Flower	533	√	√	√	√	√	√			√	
	Fruit	178		√							√	
SlMLO11	Leaf	475	√	√	√	√	√	√		√		
	Root	70										
	Flower	475	√	√	√	√	√	√		√		
	Fruit	475	√	√	√	√	√	√		√		
SlMLO12	Flower	532	√	√		√		√		√		
SlMLO13	Leaf	63	√									
	Root	558	√	√		√		√			√	√
	Flower	558	√	√		√		√			√	√
	Fruit	558	√	√		√		√			√	√
SlMLO14	Leaf	563	√	√	√	√	√					
SlMLO15	Leaf	459	√		√		√			√		
	Root	56										
	Flower	70										
	Fruit	84								√		
SlMLO16	Leaf	477	√		√	√	√					

*When no deviating transcripts are present for one SlMLO, the one from leaf has been used for motif analysis. Cells highlighted in gray indicate the absence of the corresponding motif*.

Finally, the SlMLO protein family was also used as input to search for conserved motifs. Ten patterns of consecutive amino acids, having a length ranging from 40 to 70 and shared by at least three MLO sequences (Table 3B), were found. Interestingly, four of these motifs included transmembrane domains, while the others were located in the second intracellular and extracellular domains, in the C-terminus and in the calmodulin-binding domain. The motifs seven and nine were shared only by SlMLO4/SlMLO6 and SlMLO10/SlMLO13 respectively while the motif ten was only present in the amino acid sequences of SlMLO13 of root, flower, and fruit. Those motifs might indicate regions of peculiar importance for the specific function of these homologs.

**Table 3B:** **Features details of the consensus motifs reported in Table 3A as predicted by the MEME software package (http://meme-suite.org/tools/meme)**.

	**Sequence consensus**	**Width**	**e-value**	**Location**
MOTIF 1	NAFQMAFFFWIWWEYGWKSCFWDNFIPIIIRLVMGVKVQVWCSYMTLPLYARVTQM	56	6.5e-1021	TM6
MOTIF 2	PTWAVAMVCAVIVAISIFIERIIHKLGKWLKKKNKKALYEALEKIKEELMLLGFISLLLTVCQDYISQIC	70	1.5e-1076	TM1
MOTIF 3	LLWIVCFFRQFYRSVNKSDYLTLRHGFIMAHCAPNNYNNFDYYMYRMREDDFDF	54	3.9e-840	2 IC
MOTIF 4	EGKVPFASYEALHQLHIFIFVLAVAHVLYCCTTMWLGMAKMRQWRAWEDETKT	53	6.7e-823	TM3
MOTIF 5	VGISWYLWIFVVLCLLLNINGWHSYFWIPFFPLILILLVGTKLEHIITQMAVEIAE	56	1.0e-402	TM5
MOTIF 6	GSTMKKSIFDENVRDALRKWHMTVKKRKKHKYDRSNTTRSNCPACSMAMDGPNHP	55	8.8e-386	CaMBD
MOTIF7	HRYKTTGHSSRFQGYSDQEASDLENDPTTPMTRAEIATTHIDHDDTEIHVHIPQNGESTRNEDDFSFVKP	70	2.50E-178	C-term
MOTIF 8	PPNVADTMLPCPPNNKDQAKEEEHCRHLGWYERRHLACNE	40	6.30E-149	2 EC
MOTIF9	VNSSAVSSHFYPCSPPDNDMKSAITRDAIHGSSYSNHSTS	40	1.90E-114	2 EC
MOTIF 10	SPCSSRGSFNHLDEKVLSNDHQEDCIVETTNQPGHELSFRNSEVLVTDAEEIVDDEADKIETLFELFQKT	70	2.80E-89	C-term

*For each motif, the MEME e-value for significance and the position of each motif in one of the MLO protein domains (transmembrane –TM-, extracellular –EC-, intracellular –IC-, C-terminus –C-term-, calmodulin-binding –CaMBD- domain) is indicated*.

### Phylogenetic analysis of the tomato SlMLO protein family

**Figure 1:**
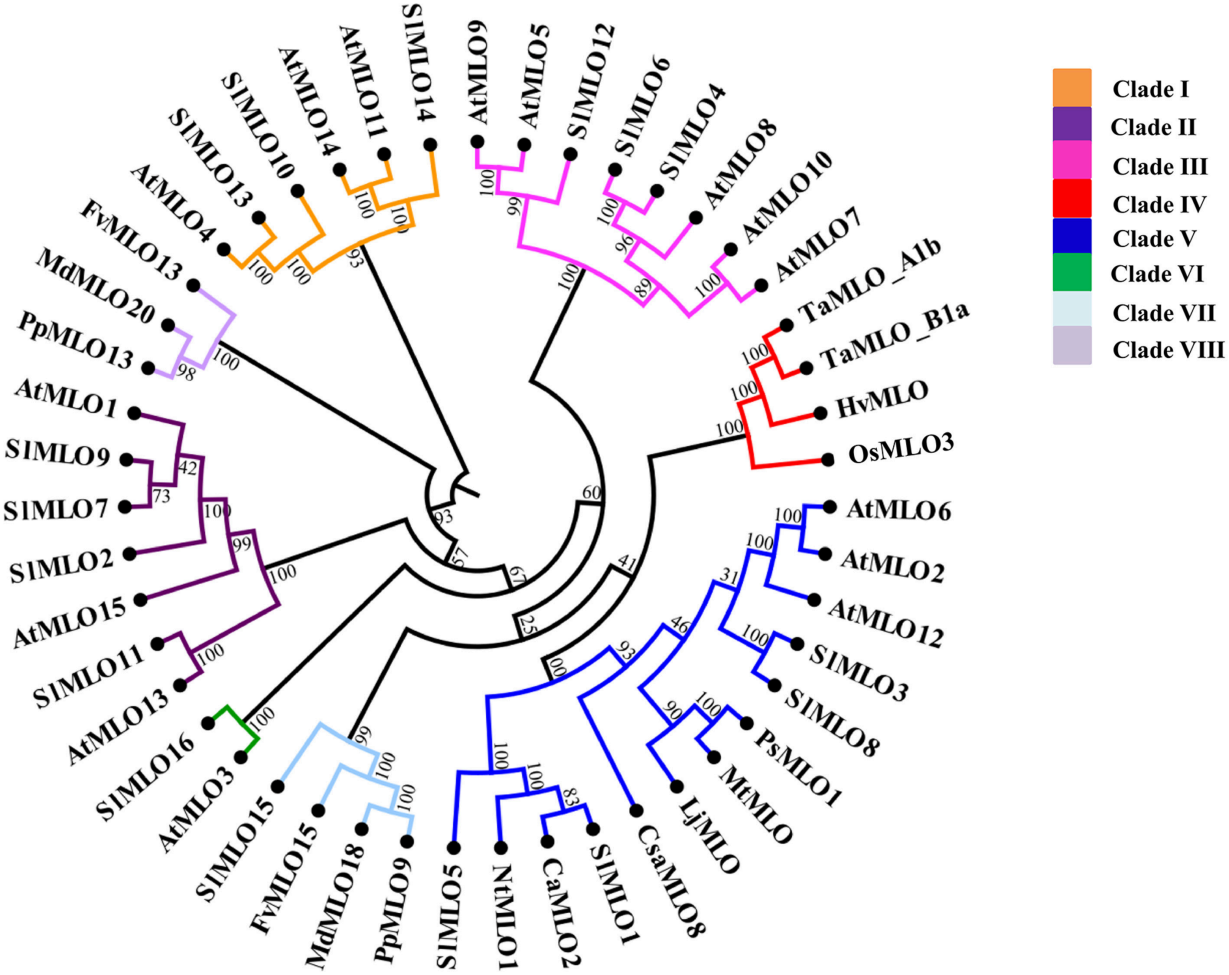
**Rooted circular cladogram showing the phylogenetic relationships of the tomato SlMLO proteins**. A UPGMA-based tree comprises all the sequences of the MLO protein family of Arabidopsis (At) and tomato (Sl). Individual sequences of apple (Md), tobacco (Nt), cucumber (Csa), pea (Ps), *Lotus japonicus* (Lj), barrel clover (Mt), pepper (Ca), barley (Hv), rice (Os), and wheat (Ta) are included. Numbers on each node represent bootstrap values based on 100 replicates. Phylogenetic clades are designated with colors and Roman numbers according to the position of AtMLO homologs and apple MdMLO, as indicated by Pessina et al. (2014). The tomato SlMLO protein sequences used for this tree derived all from the translation of the transcripts cloned from leaf of the cv. Moneymaker, except for SlMLO12, which corresponds to the translated sequence of flower. Accession numbers of the sequences used, other than tomato SlMLO, are listed in Supplementary Table 2.

A phylogenetic analysis was carried out in order to establish the relationships between SlMLO proteins and MLO proteins of other plant species (Arabidopsis AtMLO1-15, pea PsMLO1, *Lotus japonicus* LjMLO1, barrel clover MtMLO1, pepper CaMLO2, tobacco NtMLO1, cucumber CsaMLO8, apple MdMLO18 and MdMLO20, strawberry FvMLO13 and FvMLO15, peach PpMLO9 and PpMLO13, barley HvMLO, rice OsMLO3, and wheat TaMLOA1b and TaMLOB1a). The resulting tree contains eight different clades (Figure 1). These were named by Roman numerals from I to VIII, in accordance with previous studies performing phylogenetic analysis on the Arabidopsis and apple MLO protein families (Devoto et al., 2003; Pessina et al., 2014).

Five clades, namely clade I, II, III, V, and VI, contain both tomato and Arabidopsis homologs; clade IV contains only the monocot MLO homologs that were selected for this study; clade VII contains only SlMLO15 together with apple, peach and strawberry MLO proteins (MdMLO18, PpMLO9, and FvMLO15, respectively). No SlMLO homologs could be assigned to clade VIII, which only contains Rosaceae MLO homologs (Figure 1).

Three tomato MLO homologs, SlMLO3, SlMLO5, and SlMLO8, cluster together with SlMLO1 in clade V, containing all the known eudicot MLO homologs functionally related to powdery mildew susceptibility (AtMLO2, AtMLO6, AtMLO12, PsMLO1, LjMLO1, MtMLO1, CsaMLO8, NtMLO1, and CaMLO2; Figure 1; Elliott et al., 2005; Consonni et al., 2006; Bai et al., 2008; Pavan et al., 2009; Humphry et al., 2011; Várallyay et al., 2012; Zheng et al., 2013; Appiano et al., 2015; Berg et al., 2015).

The tomato homologs SlMLO4, SlMLO6, and SlMLO12 group in clade III together with AtMLO7, which regulates Arabidopsis pollen tube reception by the synergid cells, whereas SlMLO10, SlMLO13, and SlMLO14 are the closest tomato homologs to the root thigmomorphogenesis regulating proteins, AtMLO4 and AtMLO11, in clade I (Figure 1).

Finally, clade II includes four tomato SlMLO homologs (SlMLO2, SlMLO7, SlMLO9, and SlMLO11) together with three Arabidopsis proteins (AtMLO1, AtMLO13, and AtMLO15) and clade VI harbors only AtMLO3 and tomato SlMLO16 (Figure 1).

### Expression profiles of *SlMLO* homologs in axenic conditions and upon powdery mildew challenge

**Figure 2:**
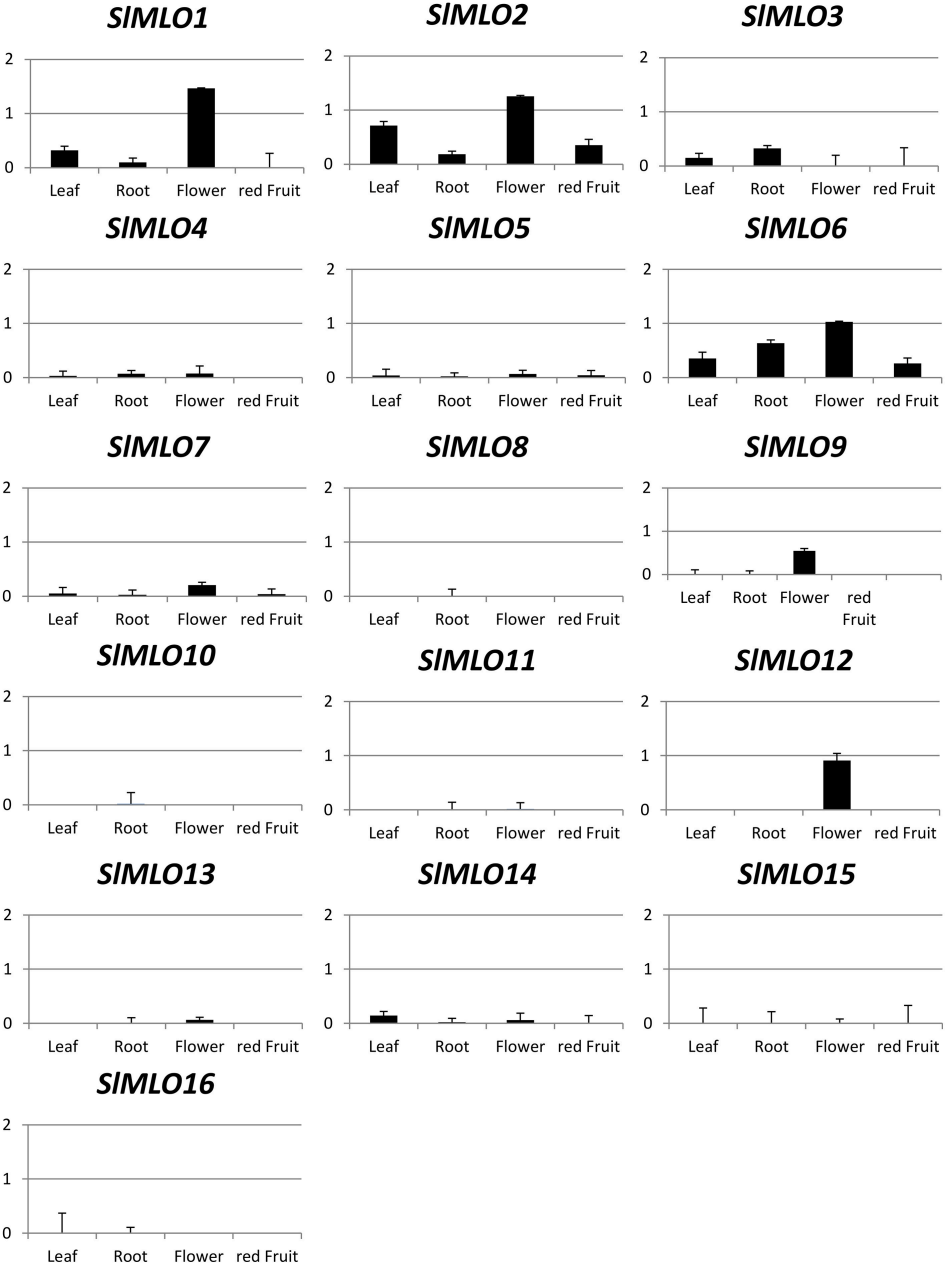
**Relative expression level of ***SlMLO*** transcripts evaluated in four different tissues (leaf, root, flower, and mature fruit) of the tomato cv. Moneymaker in axenic condition**. The expression level of each gene is compared to the abundance of Ef1α which was used as reference gene. Bars show standard errors based on three technical replicates. Similar trends are reported in Supplementary Figure 3.

The expression level of *SlMLO* genes was determined in four different tissues (leaf, root, flower, and ripened fruit). These were found to vary considerably among *SlMLO* genes, and it was not possible to assign clade-specific expression patterns (Figure 2). Concerning clade V, *SlMLO5* and *SlMLO8* were found to be characterized by very low expression levels in all the tissues. Interestingly, *SlMLO1* was found to be less expressed in leaves compared to flowers. Our results are supported by the collection of RNA-seq data, as shown by the FPKM (fragments per kilobase of exon per million fragments mapped) values for the four tissues under investigation of each homolog represented into graphs of Supplementary Figure 3.

**Figure 3:**
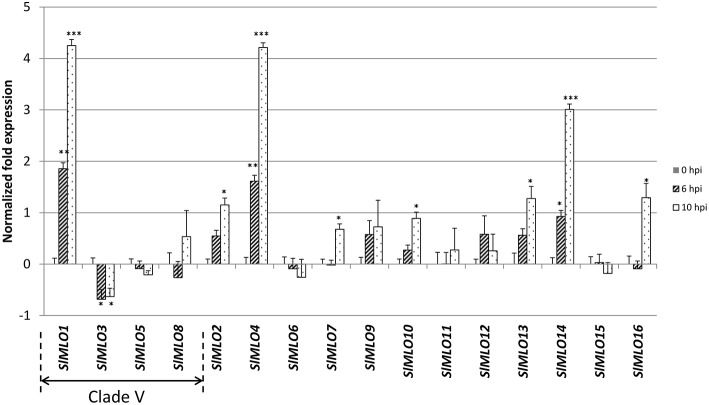
**Relative expression level of the ***SlMLO*** gene family in response to ***O. neolycopersici*** inoculation**. Samples were collected at 0, 6, and 10 h after inoculation (hpi). Transcript abundance of each *SlMLO* homolog was normalized against the transcription level of the 60S ribosomal protein L33 used as reference gene. Bars show standard errors based on four biological replicates. Asterisks refer to significant differences with respect to non-inoculated plants (0 hpi), inferred by mean comparisons with a Student's *t*-test (^*^p < 0.05, ^**^p < 0.01, ^***^p < 0.001). The *SlMLO* genes harbored in clade V, based on the phylogenetic tree of Figure 1, are indicated by an arrow spanning their corresponding bars. Similar results were obtained by using the elongation factor Ef1α as housekeeping gene (Supplementary Figure 4).

Next, we investigated the expression profile of the *SlMLO* gene family in response to *O. neolycopersici*, using L33 as a reference gene (Figure 3). *SlMLO1* expression significantly increased at 6 and 10 h after pathogen challenge. No other *SlMLO* homolog in clade V (*SlMLO3, SlMLO5, SlMLO8*) showed pathogen-dependent up-regulation.

On the other hand, a significant upregulation in response to *O. neolycopersici* was observed for *SlMLO* homologs outside clade V, namely *SlMLO2, SlMLO4, SlMLO7, SlMLO10, SlMLO13, SlMLO14*, and *SlMLO16*. In particular, the expression of *SlMLO4* and *SlMLO14* at 10 h after inoculation was comparable to the one of *SlMLO1*, and ~four-fold and ~three-fold higher than the one of control plants, respectively.

Similar results were obtained repeating the expression analysis using Ef 1α as reference gene (Supplementary Figure 4).

In order to confirm the strong up-regulation of the above mentioned genes, a second inoculation experiment was carried out, sampling leaf tissues at the same time points (0, 6, and 10 hpi). The results presented in Supplementary Figure 5 indicate that indeed *SlMLO1, SlMLO4*, and *SlMLO14* show a statistically significant up-regulated expression due to the *O. neolycopersici* challenge. The slight down-regulated expression of *SlMLO3* observed after the first pathogen inoculation was not confirmed in the second experiment.

### Functional characterization of clade V *SlMLO* homologs

Based on their relatedness with eudicot *MLO* homologs predisposing to PM susceptibility, including *SlMLO1*, the newly identified *SlMLO* homologs in clade V (*SlMLO3, SlMLO5*, and *SlMLO8*, Figure 1) were further investigated with respect to their role in the interaction with *O. neolycopersici*. Therefore, specific RNAi silencing constructs for these three homologs were developed, which were used to transform the susceptible cultivar Moneymaker (MM) (Supplementary Figure 6 and Supplementary Table 3). A silencing construct targeting *SlMLO1* was included as control, which was expected to lead to a resistant phenotype.

**Figure 4:**
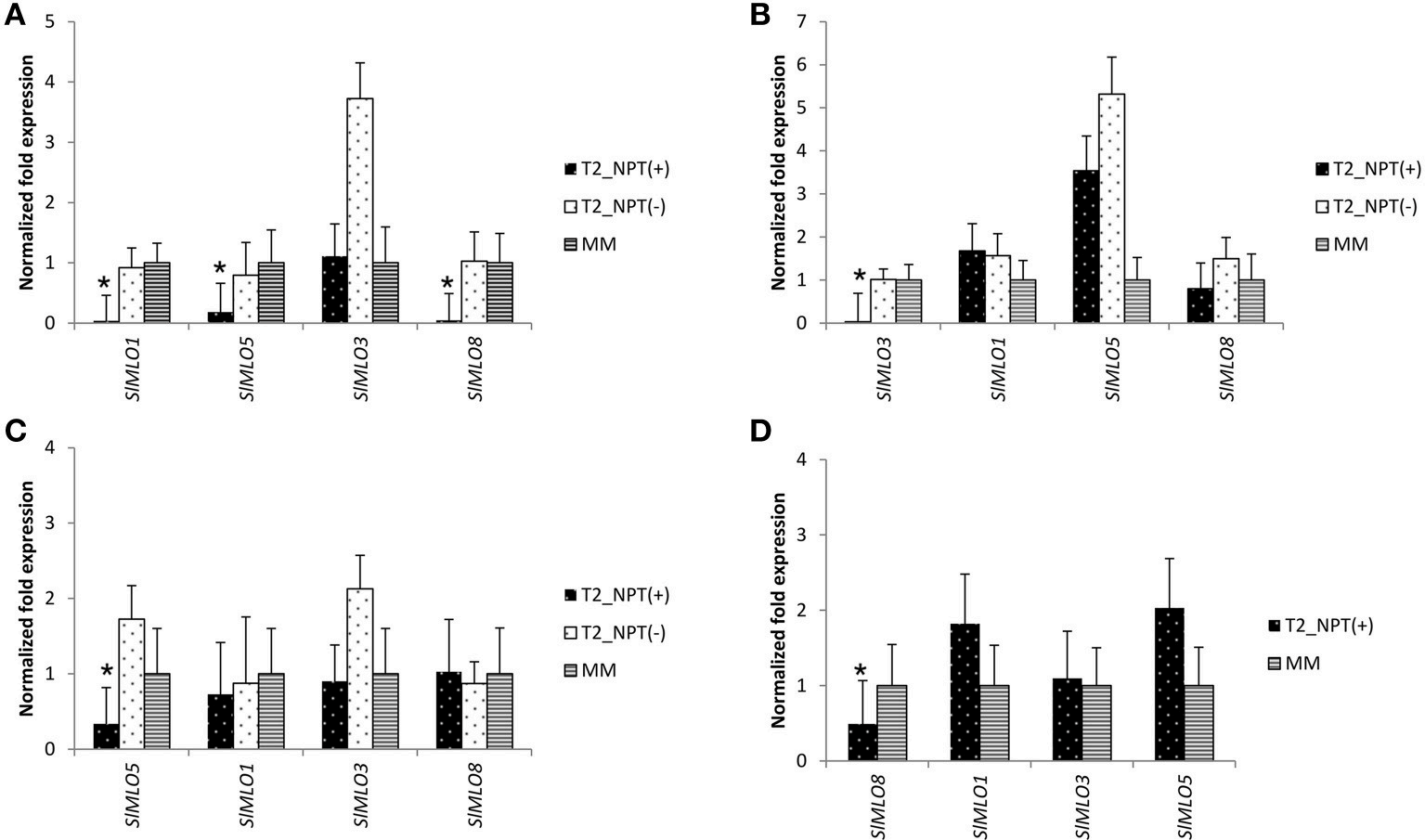
**Evaluation of the silencing effect of the RNAi constructs designed to target ***SlMLO1***, ***SlMLO3***, ***SlMLO5***, and ***SlMLO8*** in segregating T_**2**_ families of the tomato cv. Moneymaker**. Panels **(A–D)** show the expressions of clade V *SlMLO* homologs in plants of T_2_ families, derived from different transformation events and segregating for the presence [T_2__NPT(+)] or absence [T_2__NPT(-)] of the RNAi::*SlMLO1*, RNAi::*SlMLO3*, RNAi::*SLMLO5*, and RNAi::*SlMLO8* constructs, respectively. In **(A)** bars and standard errors refer to eight plants T_2__NPT(+) and four plants T_2__NPT(-) of two T_2_ families and four Moneymaker (MM) plants. In **(B)** bars and standard errors refer to ten plants T_2__NPT(+) and five plants T_2__NPT(-) of three T_2_ families and four MM individuals. In **(C)** bars and standard errors refer to ten plants T_2__NPT(+) and five plants T_2__NPT(-) of three T_2_ families and four MM individuals. In **(D)** bars and standard errors refer to 10 T_2__NPT(+) of two T_2_ families and four MM individuals.

Ten to 20 T_1_ plants were obtained for each silencing construct. The expression of the target genes was assessed by means of real-time qPCR (Supplementary Figure 7) and T_1_ plants with a reduced level of expression of the target gene were allowed to self-pollinate to develop T_2_ families. In total, two independent T_2_ families (each segregating for the presence of the silencing construct) were developed for *SlMLO1* and *SlMLO8*, and three were obtained for *SlMLO3* and *SlMLO5*. Transgenic individuals of each family were further assessed for the silencing levels of target genes and other clade V homologs. This revealed successful silencing of each target genes and no unwanted co-silencing in transgenic RNAi::*SlMLO3, SlMLO5*, and *SlMLO8* individuals (Figures 4B–D). Conversely, T_2_ transgenic plants of two T_2_ families carrying the RNAi::*SlMLO1* silencing construct were characterized by the simultaneous silencing of *SlMLO1, SlMLO5*, and *SlMLO8* (Figure 4A and Supplementary Figure 8).

**Figure 5:**
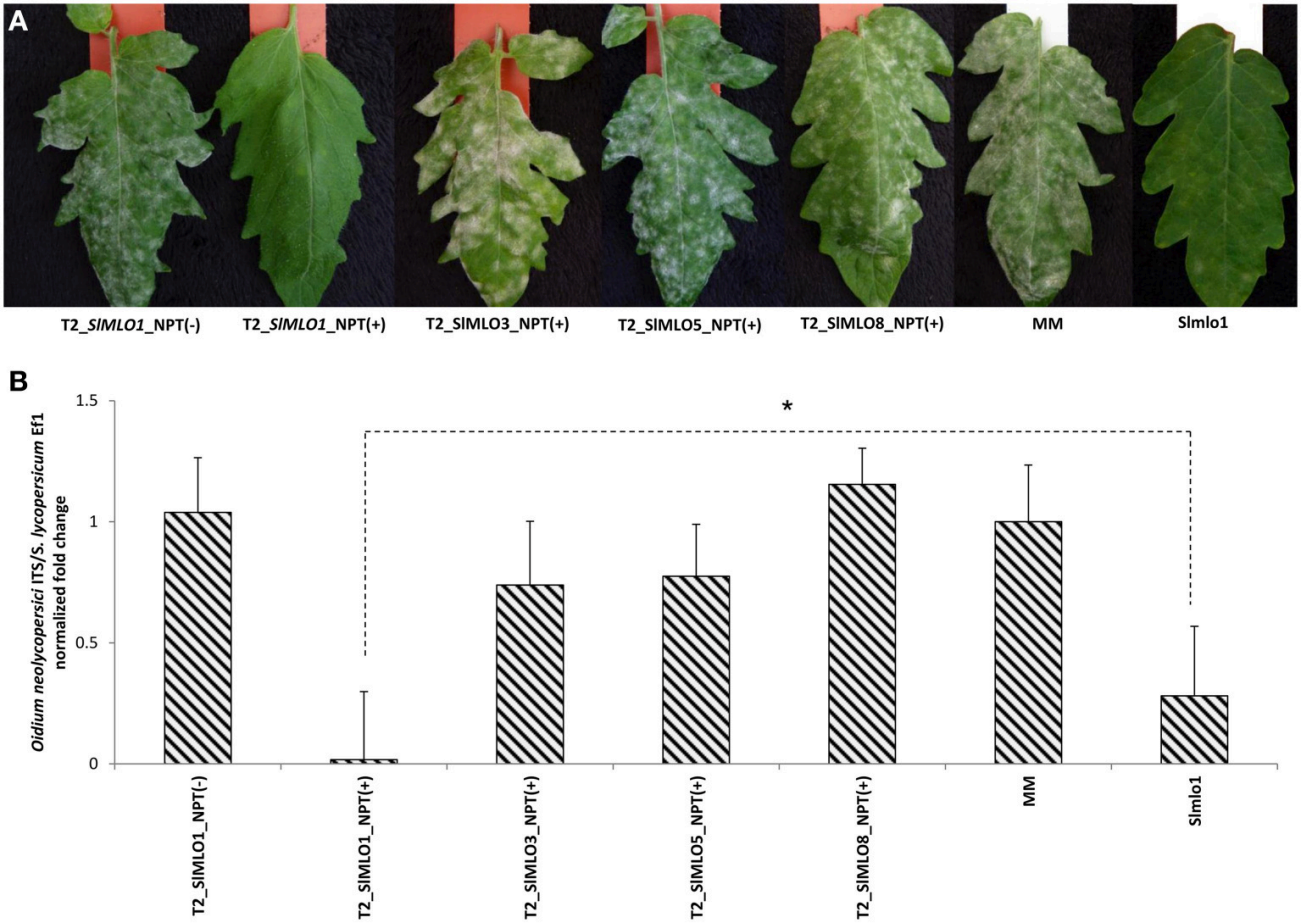
**Powdery mildew evaluation on plants of segregating T_**2**_ families obtained with silencing constructs targeting ***SlMLO*** genes to attest their involvement in ***O. neolycopersici*** susceptibility**. Panel **(A)** shows the phenotypic evaluation of the powdery mildew growth on leaves of different T_2_ individuals that have been evaluated for the (from left to right) absence of the RNAi::*SlMLO1*, presence of the RNAi::*SlMLO1*, presence of the RNAi::*SlMLO3*, presence of the RNAi::*SlMLO5*, and presence of the RNAi::*SlMLO8* silencing constructs, followed by one individual of the cv Moneymaker (MM) and one of the Slmlo1 line carrying a loss-of-function mutation in the *SlMLO1* gene. Panel **(B)** shows the relative quantification of the ratio between *Oidium neolycopersici* and plant gDNAs in transgenic individuals [NPT(+)] and not transgenic individuals [NPT(-)] segregating in T_2_ families obtained with the silencing constructs above described. Bars and standard errors refer to (from left to right) four individuals of two independent T_2_ families not carrying the RNAi::*SlMLO1*, eight individuals of the same two T_2_ families carrying the RNAi::*SlMLO1*, 18 individuals of three independent T_2_ segregating families carrying the RNAi::*SlMLO3* construct, 18 individuals of three independent T_2_ segregating families carrying the RNAi::*SlMLO5* construct and 20 individuals of two T_2_ segregating families carrying the RNAi::*SlMLO8* construct, next to 10 MM plants and 10 plants of the Slmlo1 line. The asterisk refers to the significant difference in susceptibility between individuals of the T_2__SlMLO1_NPT(+) and Slmlo1, inferred by mean comparisons with a Student's *t*-test (^*^p < 0.05).

As expected, T_2_ progenies carrying the RNAi::*SlMLO1* construct segregated for PM resistance: T_2_ plants carrying the silencing construct [T_2__*SlMLO1*_NPT(+)] were resistant, whereas non-transgenic plants [T_2__*SlMLO1*_NPT(-)] were susceptible as MM (Figure 5A). In contrast, all T_2_ progenies segregating for *SlMLO3, SlMLO5*, and *SlMLO8* silencing constructs visually appeared to be fully susceptible to *O. neolycopersici* (Figure 5A). The quantification of disease severity on these lines using real-time qPCR supported phenotypic observations, as no significant difference was found between T_2__*SlMLO3*_NPT(+), T_2__*SlMLO5*_NPT(+), T_2__*SlMLO8*_NPT(+) plants, and MM (Figure 5B and Supplementary Figure 9). For each T_2_ family, transgenic and non-transgenic plants were phenotypically indistinguishable.

The Slmlo1 line, harboring a loss-of-function mutation in the *SlMLO1* gene (Bai et al., 2008), is resistant to PM, however lower leaves displayed PM symptoms (Figure 5A). Compared to the plants of the Slmlo1 line, RNAi plants carrying the RNAi::*SlMLO1* construct [T_2__*SlMLO1*_NPT(+) plants] showed no PM symptom and also a significantly lower amount of fungal biomass (Figure 5B and Supplementary Figure 9A). Therefore, further microscopic observations were carried out to study the fungal growth on the Slmlo1 line and T_2__*SlMLO1*_NPT(+) plants.

**Figure 6:**
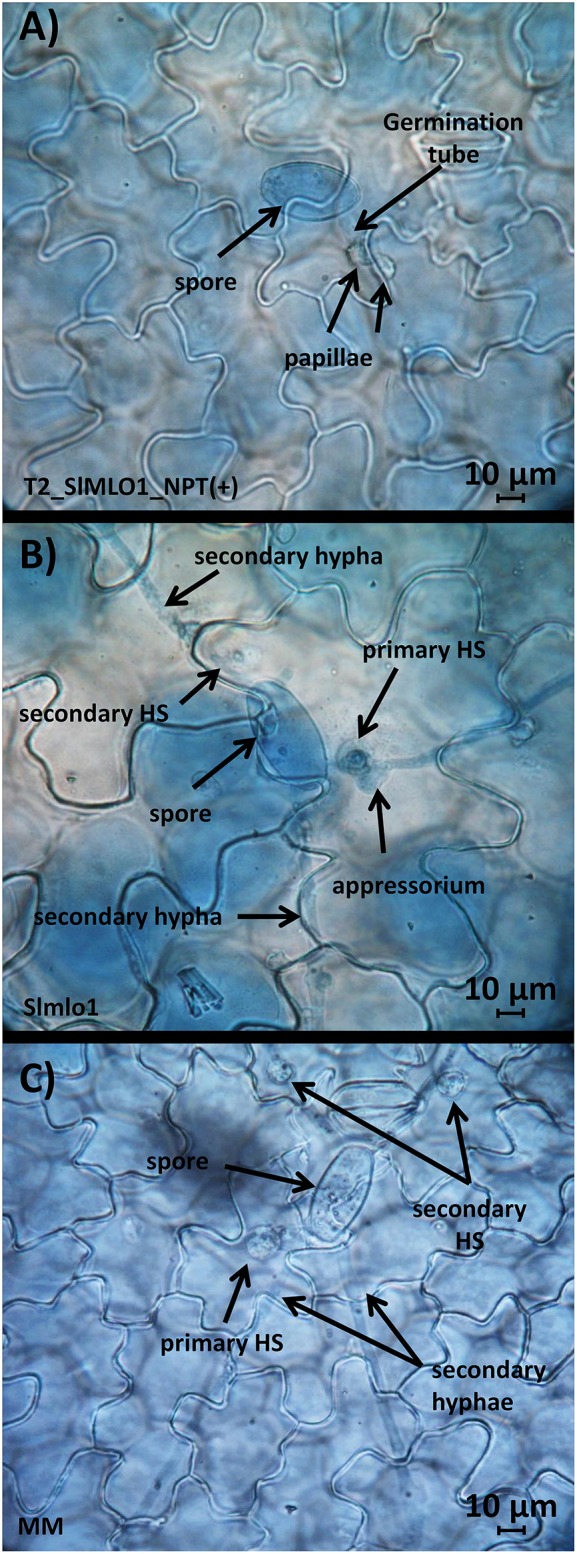
**Effect of the silencing of ***SlMLO1***, ***SlMLO5***, and ***SlMLO8*** in tomato cv Moneymaker background compared with the Slmlo1 line harboring a loss-of-function of the ***SlMLO1*** gene**. Panel **(A)** refers to a transgenic plant carrying the RNAi::*SlMLO1* construct, Panel **(B)** a plant of the Slmlo1 line and **(C)** a plant of the tomato cv. Moneymaker. Panels **(A–C)** show fungal structures (spores, germination tube, appressorium, haustorium –HS-, and hyphae) and the plant cellular reaction of papilla apposition at the sites of fungal penetration.

Since the two T_2_ families carrying the RNAi::*SlMLO1* construct showed no difference with respect to the level of reduced expression of the *SlMLO* homologs and fungal biomass quantification (Supplementary Figures 8, 9), we used one T_2_ family for microscopic study. Compared to MM, fungal growth was significantly reduced in both Slmlo1 and T_2__RNAi::*SlMLO1*_NPT(+) individuals due to the formation of a papilla beneath the appressorium (Figure 6). Interestingly, the rate of papilla formation in T_2__RNAi::*SlMLO1*_NPT(+) (93.3% of the infection units) was significantly higher than in Slmlo1 (64.4% of the infection units; Table 4). In some cases, *O. neolycopersici* was still able to penetrate epidermal cells and form haustoria with a rate of 48.9% in Slmlo1 and 30% in T_2__RNAi::*SlMLO1*_NPT(+) (Table 4 and Figure 6). The general development of the spores on the two genotypes was strikingly different: while on the Slmlo1 line the fungus could produce mostly up to two secondary hyphae (in 36.7% of the total infection units), on T_2__RNAi::*SlMLO1*_NPT(+) individuals fungal growth was significantly reduced after producing a germination tube (Table 4 and Figure 6).

**Table 4:** **Development of ***Oidium neolycopersici*** growth on the susceptible genotype Moneymaker and on the two resistant genotypes, Slmlo1 carrying a loss-of-function ***SlMLO1*** gene and plants of a T_**2**_ family selected to carry the RNAi::***SlMLO1*** silencing construct which can effectively silence ***SlMLO1, SlMLO5***, and ***SlMLO8*****.

**Genotype**	**Percentage of infection units (IU)**					**Hyphae per IU**				
	**Primary AP**	**Primary papilla**	**Primary HS**	**Secondary Papilla**	**Secondary HS**	**1**	**2**	**3**	**4**	**5**
MM	100	0	90.2	0	68.3	76.8	67.1	35.4	6.1	0
Slmlo1	100	64.4	48.9	23.3	14.4	43.3	36.7	18.9	3.3	0
T_2__RNAi::*SlMLO1*_NPT(+)	100	93.3^*^	30.0	2.2	0.0	11.1	7.8	3.3	0.0	0

*AP, appressorium; HS, haustorium*;

* *p < 0.05 compared to Slmlo1*.

## Discussion

### Structure and evolution of the *SlMLO* gene family

In this study, we followed an *in silico* approach to assign 16 homologs to the tomato *MLO* gene family. This is consistent with the results of previous studies reporting the *MLO* gene families of several diploid species made of a number of homologs variable from 13 to 21 (Devoto et al., 2003; Feechan et al., 2008; Liu and Zhu, 2008; Shen et al., 2012; Pessina et al., 2014; Schouten et al., 2014; Appiano et al., 2015). This suggests that a similar number of *MLO* homologs is likely to be retrieved in future genome-wide investigations involving diploid eudicot species.

Information on chromosomal localization was available for all the *SlMLO* homologs with the exception of *SlMLO4*. However, potato and tomato genomes are highly syntenic (Tomato Genome Consortium, 2012) and the closest *SlMLO4* homolog in potato (Sotub02g007200) is positioned on chromosome 2, thus suggesting that *SlMLO4* is also located on tomato chromosome 2.

Cloning of the *SlMLO* gene family from different tissues of the cultivar MM revealed the occurrence of transcripts deviating from predictions available at the SGN database, indicating that, despite the efforts of the tomato resequencing project, the assembly of genomic regions and the prediction of certain loci are not correct yet. Moreover, several cases of differentially spliced variants among plant tissues were observed, mostly due to intron retention and exon skipping, as it is in the case of *SlMLO5, SlMLO9, SlMLO11, SlMLO13*, and *SlMLO15*. Due to the method used in this study to amplify the *SlMLO* homologs, we cannot exclude that the intron retention is the result of the amplification of non-mature mRNA. However, intron retention was previously reported to be a very common type of alternative splicing in Arabidopsis and rice (Ner-Gaon et al., 2007). There is also a well-documented evidence indicating organ-specific regulation of alternative splicing in plants (Palusa et al., 2007). More studies need to be performed to unravel its complexity and functional significance. Certainly, alternative forms of splicing, such as the ones found in this study, can lead to aberrant mRNA isoforms that cause the loss-of-function of a *MLO* gene. An example is reported by a recent study conducted by Berg et al. (2015) in cucumber. They show that the integration of a transposable element in the genomic region of the *CsaMLO8* leads to an aberrant splicing that causes the loss-of-function of this susceptibility gene in a resistant cucumber genotype.

The identification of protein motifs conserved in transmembrane domains of specific SlMLO homologs (Tables 3A,B) corroborates previous findings in Solanaceae plant species (Appiano et al., 2015). This indicates that transmembrane domains, which are thought to provide a common scaffold invariable for the whole MLO family (Devoto et al., 1999), might also be involved in conferring specific functions to MLO homologs. Future functional studies of targeted mutagenesis of transmembrane MLO protein regions can help to unravel their actual role.

All the SlMLO proteins were found to group in six phylogenetic clades together with other eudicot MLO homologs, including the complete Arabidopsis AtMLO family and certain members of the apple, peach and strawberry MLO family. No SlMLO homolog could be assigned to clade IV, previously shown to contain monocot MLO homologs and a few eudicot homologs (grapevine VvMLO14, strawberry FvMLO17, and peach PpMLO12) (Feechan et al., 2008; Pessina et al., 2014).

Based on their sequence relatedness with Arabidopsis AtMLO proteins of known function, it is logical to argue that one or more of the tomato SlMLO homologs in clade III and clade I could regulate the processes of root response to mechanical stimuli and pollen tube reception, respectively. The RNAi silenced lines of several *SlMLO* homologs generated in this study could be useful to assign new functions to MLO proteins which have gone unnoticed by the evaluation of the available panel of Arabidopsis *Atmlo* mutants.

### Possible pleiotropic effects and co-functioning of SlMLO homologs

RNA-seq data, RT-PCR and real-time qPCR of the *SlMLO* gene family confirmed the expression of all the 16 *SlMLO* homologs. Often, it was possible to detect high level of transcript of the same *SlMLO* homolog in more than one of the four tissues under study (leaf, root, flower, and mature fruit). This is in line with the findings of the previous study of Chen et al. (2006), investigating the expression pattern of the Arabidopsis *AtMLO* gene family in several tissues. Overall, this body of evidence suggest that: (a) different *MLO* homologs may have synergistic or antagonistic roles in regulating the same biological process; (b) *MLO* homologs may have pleiotropic effects on different biological processes. Co-functioning between *MLO* homologs has been demonstrated to occur in Arabidopsis, where different *AtMLO* genes co-participate in the same tissue to determine powdery mildew susceptibility and root response to mechanical stimuli (Consonni et al., 2006; Chen et al., 2009). A yet unidentified additional biological function could be hypothesized for the *SlMLO1*, previously shown to act as a susceptibility gene toward *O. neolycopersici* (Pavan et al., 2009). This gene was found to exhibit its strongest expression level in tomato flower and moderate expression in root, two tissues which are less or not attacked by the fungus, respectively. Moreover, additional biological roles for *SlMLO1* would explain why this gene has not been excluded from evolution, despite promoting susceptibility to PM pathogen. Interestingly, evidence shows that the *SlMLO1* orthologs in barley and Arabidopsis are involved in the interaction with pathogens other than powdery mildews, such as necrotrophs and hemibiotroph (Jarosch et al., 1999; Kumar et al., 2001; Consonni et al., 2006). Thus, it is worthwhile to test the RNAi::*SlMLO1* plants with more pathogens to broaden its role in plant-pathogen interactions.

### *SlMLO* homologs involved in powdery mildew susceptibility

In this study, we mainly focused on the *SlMLO* genes grouped in the clade V containing all the *MLO* homologs associated with PM susceptibility in eudicots. The presence of multiple tomato homologs in clade V is in accordance with the existence of three Arabidopsis proteins (AtMLO2, AtMLO6, and AtMLO12) associated with increased fungal penetration (Consonni et al., 2006).

We showed that tomato *SlMLO3, SlMLO5*, and *SlMLO8*, differently from *SlMLO1*, do not increase their expression upon *O. neolycopersici* challenge. Furthermore, strong silencing of the same homologs in a susceptible tomato background (Moneymaker) did not result in a significant reduction of disease symptoms (Figures 3–5).

Plants transformed with a construct meant to silence *SlMLO1* showed co-silencing of *SlMLO5* and *SlMLO8*, due to sequence relatedness between these genes (Figure 4). Interestingly, these plants were also significantly more resistant than plants of the Slmlo1 line (Figure 5). Since the Slmlo1 line is only a BC_3_S_2_ line carrying the *Slmlo1*mutation (the *ol-2* gene) in MM background, we cannot fully exclude background effects from the *ol-2* donor, the resistant line LC-95 of *S. lycopersicum* var. *cerasiforme*, which might add to partial susceptibility phenotype of the Slmlo1 line. On the other hand, our scenario is reminiscent of the one reported in Arabidopsis, where *Atmlo2* single mutant displays partial PM resistance, whereas *Atmlo2/Atmlo6/Atmlo12* triple mutant is fully resistant (Consonni et al., 2006). Also in grape, more than one *VvMLO* genes are involved in susceptibility to powdery mildew (Feechan et al., 2008, 2013). Taken together with the knowledge of functional redundancy in Arabidopsis and grape, our data suggest that in tomato *SlMLO1, SlMLO5*, and *SlMLO8* are functionally redundant as PM susceptibility factors with *SlMLO1* playing a major role. Our results showed that the contribution of *SlMLO5* and *SlMLO8* is too small to be observed with an RNAi approach silencing individual genes, but a complementation experiment using the Slmlo1 line could be more suitable to observe their minor role.

It cannot be excluded yet that the other clade V tomato homolog, *SlMLO3*, is also involved in plant-pathogen interactions. However, it is worthwhile to notice that the SlMLO3 protein is missing three of the six motifs contained in SlMLO1, two of which are also present in SlMLO5 and SlMLO8 (Table 3B). The motif three in Table 3B is located in the second intracellular domain, which is known to be involved together with the third intracellular domain in the protein functionality (Elliott et al., 2005). This would suggest that *SlMLO3* might miss important features to be fully functional as susceptibility factor. Overexpressing *SlMLO3* in the Slmlo mutant may provide a better evidence on its eventual role as a functional susceptibility gene.

Interestingly, we noticed that *SlMLO4* and *SlMLO14*, which do not belong to clade V, are up-regulated upon *O. neolycopersici* infection (Figure 3 and Supplementary Figures 4, 5). *SlMLO14* is closely related to *AtMLO4* and *AtMLO11*, which are involved in root thigmomorphogenesis (Chen et al., 2009), while *SlMLO4* is related to *AtMLO7*, involved in pollen tube reception (Kessler et al., 2010). In Arabidopsis, mutation of *AtMLO4, AtMLO7*, and *AtMLO11* does not result in PM resistance. Thus, we expected that silencing of *SlMLO4* and *SlMLO14* in tomato will not lead to PM resistance too. The up-regulated expression of *SlMLO4* and *SlMLO14* after challenge with *O. neolycopersici* might be the result of shared regulatory cis-acting elements. We used a 2 kb region located upstream the starting codon of *SlMLO1, SlMLO4*, and *SlMLO14* coding sequences to search for shared regulatory elements through the online database Plant Care (http://bioinformatics.psb.ugent.be/webtools/plantcare/html/) (Lescot et al., 2002). We found at least five common motifs which are associated with upregulation by multiple biotic and/or abiotic stresses: ABRE (CACGTG), involved in abscisic acid responsiveness, CGTCA- and TGACG-motifs, involved in the MeJA responsiveness, HSE (AAAAAATTC), involved in heat stress responsiveness, and TCA (CCATCTTTTT/GAGAAGAATA) element, involved in salicylic acid response. It is intriguing whether *SlMLO4* and *SlMLO14* can act as a susceptibility gene to PM. Till now, only clade IV and clade V *MLO* genes have been studied for their role as a susceptibility gene. To further study these PM-induced non-clade V *SlMLO* genes, a complementation test using the Slmlo mutant could be performed.

In conclusion, this study provides a comprehensive characterization of the *MLO* gene family in tomato by analyzing their genomic structure, expression profile and predicted protein motifs. In tomato, there are 17 *MLO* genes which can be grouped into six clades. The expression of these *MLO* genes can be tissue specific and some *MLO* genes show alternative splicing variants in different tissues. The *SlMLO1* in clade V is confirmed to be the major PM susceptibility factor. In addition, two clade V genes, *SlMLO5* and *SlMLO8*, are suggested to have a partial redundant function, as described in Arabidopsis for *AtMLO2, 6*, and *12* genes (Consonni et al., 2006). To label an *MLO* gene as a PM susceptibility gene, it is recommended to combine phylogenetic analysis and expression profile to select candidates of clade IV (for monocot) and V (for dicot) that are induced by PM infection. However, the upregulation of *MLO* genes outside clade V in response to PM, as shown in this study and in Pessina et al. (2014), raises the possibility that they may act as susceptibility genes. Finally, the RNAi lines generated in this study are useful materials for further assigning new biological functions to the *MLO* gene family members.

## Materials and methods

### Plant material, fungal material, and inoculation

In this study, we used the susceptible *S. lycopersicum* cultivar Moneymaker (MM), the Slmlo1 line and transgenic T_2_ families in which individual *SlMLO* gene was silenced via RNAi in MM background. The Slmlo1 mutant (the *ol-2* gene) was a natural mutation discovered in the resistant line LC-95 of *S. lycopersicum* var. *cerasiforme*. The LC-95 line was crossed with the susceptible tomato *S. lycopersicum* cv. Super Marmande and the F2 progeny was used for mapping in 1998 (Ciccarese et al., 1998). Later, we introgressed the *ol-2* allele into *S. lycopersicum* cv Moneymaker (MM) by backcrossing and one BC3S2 line homozygous for the *ol-2* allele (the tomato Slmlo1 line) was used in the experiment.

The powdery mildew disease assay was performed by artificial inoculation in the greenhouse. For this, the Wageningen isolate of *O. neolycopersici* (*On*) was used (Bai et al., 2008). A suspension of *O. neolycopersici* conidia was prepared, by rinsing freshly sporulating leaves of infected tomato plants with tap water. This suspension was immediately sprayed on 1 month-old tomato plants. Ten plants for each of the T_2_ progenies obtained from the transformation of each silencing construct, 10 Slmlo1 plants and 10 MM plants were used for disease assay. The scoring of powdery mildew symptoms was done 10 days after inoculation, inspecting and collecting the third and fourth true leaves for each plant.

For the evaluation of the expression of the *SlMLO* gene family, two independent inoculations were set up. In both cases, we used the cultivar MM, four and three biological replicates for each of the three time points (0, 6, and 10 h post inoculation –*hpi*-) during the first and the second inoculation, respectively.

### Identification and cloning of the *SlMLO* gene family

Putative tomato MLO protein sequences were identified in the Sol Genomics Network (SGN) (http://solgenomics.net/) database by using the BLASTP and TBLASTN algorithms with Arabidopsis AtMLO protein sequences as query. Chromosomal localization, sequences of the corresponding genes and introns/exons boundaries were inferred by annotations from the International Tomato Annotation Group (ITAG).

Aiming at cloning and sequencing the *SlMLO* gene family from the cultivar MM, total RNA from leaf, root, flower and ripened fruit was isolated (RNeasy® mini kit, Qiagen). The different tissues were collected from five MM plants and pooled together to obtain enough material for the RNA isolation. For each individual *SlMLO* homolog, two primer pairs specifically amplifying overlapping products of around 800 bp of the predicted coding sequences (CDS) were designed using the Primer3 plus online software (http://www.bioinformatics.nl/cgi-bin/primer3plus/primer3plus.cgi; Rozen and Skaletsky, 2000). The forward primer and the reverse primer of product A and product B, respectively, are located in the respective UTR regions to ensure the cloning of the complete CDS. A one-step PCR was performed to obtain the desired product (SuperScript® III One-Step RT-PCR System, Invitrogen; Supplementary Table 1). Its high sensitivity and specificity ensured the amplification of these very lowly expressed genes. Indeed, a PCR performed on a cDNA obtained with oligo(dT)_20_ primers did not yield any product for many of the homologs under investigation. The use of sequence-specific primers in the one-step PCR, on the other hand, allowed the binding of only the desired mRNA sequences.

Corresponding amplicons were visualized on agarose gel and cloned into the pGEM®-T Easy vector (Promega). Recombinant plasmids were sequenced by using universal T7 and SP6 primers.

In order to reveal gene structures and polymorphisms, *SlMLO* sequences obtained by cloned amplicons were merged using the package Seqman of the software DNASTAR® Lasergene8. The obtained consensus was aligned with the coding region of the *SlMLO* identified *in silico* and the corresponding genomic region using the CLC 7.6.1 sequence viewer software (www.clcbio.com).

Finally, for the motif analysis, the MEME (http://meme.nbcr.net/) package was used to predict consensus patterns of consecutive conserved amino acids in the SlMLO proteins deriving from the *in silico* translation of the cloned transcripts from leaf, root, flower, and fruit of the cultivar MM (Bailey et al., 2015).

### Comparative analysis

The corresponding SlMLO protein sequences of translated cloned CDS obtained from leaf and flower (in the case of *SlMLO12*) were used as dataset in the CLC 7.6.1 sequence viewer software (www.clcbio.com) for ClustalW alignment and the obtainment of an UPGMA-based comparative tree (bootstrap value was set equal to 100), together with those of the 15 Arabidopsis AtMLO homologs. Moreover, MLO proteins experimentally shown to be required for PM susceptibility were added, namely pea PsMLO1, barley HvMLO, wheat TaMLO_A1b and TaMLO_B1a, rice OsMLO3, pepper CaMLO2, tobacco NtMLO1, cucumber CsaMLO8, *Lotus japonicus* LjMLO1, and barrel clover MtMLO1. Moreover, MLO homologs of the Rosaceae species that cluster in clade VII (FvMLO15, MdMLO18, PpMLO9) and VIII (FvMLO13, MdMLO20, and PpMLO13) were included (Supplementary Table 2). The obtained UPGMA-comparative tree was then displayed as circular rooted cladogram with CLC software.

### Expression analysis of the *SlMLO* gene family in response to *O. neolycopersici*

Tissue samples from the third and fourth true leaf of 1 month-old tomato plants were collected immediately before fungal inoculation and at two time points after inoculation (6 and 10 h). The RNA isolation was performed with MagMAX-96 Total RNA Isolation kit (Applied Biosystem), following the manufacturer's instructions. Included in the protocol is a DNase treatment using the TURBO™ DNase. An aliquot of the RNA isolated was run on denaturing agarose gel to assess its integrity. Purity and concentration were determined by measuring its absorbance at 260 and 280 nm using the NanoDrop® 1000A spectrophotometer. Following this protocol for RNA isolation, intact and pure RNA was obtained and the concentration was variable between 200 and 250ng/μl.

cDNAs were synthesized by using the SuperScript III first-strand synthesis kit (Invitrogen) using the oligo(dT)_20_ primer, starting from the same amount of RNA (200 ng/μl). Specific primer pairs for each of the 16 *SlMLO* homologs, amplifying fragments ranging from 70 to 230 bp, were designed as described above (Supplementary Table 3). The amplification of single fragments of the expected size for each homolog was verified by agarose gel electrophoresis and by the observation of the melting pick. Four tomato reference genes were tested for expression stability in order to determine which ones could be suitable for normalization of the expression of *SlMLO* homologs. These included the 60S ribosomal protein L33 (GeneBank number Q2MI79), the elongation factor 1α (GeneBank number X14449), actin (Genebank XP_004236747), and ubiquitin (GeneBank number XP_004248311) (Schijlen et al., 2007; Løvdal and Lillo, 2009). Gene expression stability was assayed with the BestKeeper program (Pfaffl et al., 2004), determining as best reference genes the ribosomal protein L33 and the elongation factor 1α. The cDNAs were diluted 10-fold and used in real-time qPCR with a Bio-Rad CFX96TM thermal cycler. The thermal cycling conditions used were 95°C for 1 min, followed by 40 cycles at: 95°C for 15 s, 60°C for 1 min, and 72°C for 30 s, followed by a melt cycle of 0.5°C increment per min from 65 to 95°C. Comparable amplification efficiencies between target and reference genes were determined using the LinRegPCR software (Karlen et al., 2007). Normalization was performed according to the ΔΔC_*t*_ method (Livak and Schmittgen, 2001). Four biological replicates and two technical replicates were used in this experiment. Student's *t*-tests were applied in order to assess significant differences between the treatments.

### *SlMLO* family expression analysis in different tissues

To analyze *MLO* gene expression in leaf, root, flower and ripened fruit approximately equal amount of tissues from five MM plants were pooled and used for RNA isolation and cDNA synthesis as described in the previous paragraph. Before using them as templates, cDNAs were diluted 10-fold. Real-time qPCR was performed using the set of primers reported in Supplementary Table 3 to amplify each homolog in the four tissues above mentioned. Elongation factor 1α was used as reference gene. Data analysis was performed according to the ΔC_*t*_ method (Livak and Schmittgen, 2001). Three technical replicates for each sample were performed.

### Generation of RNAi silencing lines

Four primer pairs were designed to amplify and clone fragments from *SlMLO1, SlMLO3, SlMLO5*, and *SlMLO8* into the Gateway-compatible vector pENTR D-TOPO (Invitrogen) (Supplementary Table 3). The cloned sequences of the *SlMLO1, SlMLO3, SlMLO5*, and *SlMLO8* genes are highlighted in Supplementary Figure 6. After cloning in *E. coli* (strain DH5α), the kanamycin-resistant colonies were assessed for the presence of constructs by colony PCR. Positive recombinant plasmids were further analyzed by restriction enzyme digestion and sequencing. Next, amplicons were transferred by LR recombination reaction into the pHELLSGATE12 vector for hairpin-induced RNAi (Wielopolska et al., 2005) following the instructions provided by the manufacturer (Invitrogen), and cloned again in *E. coli* DH5α. Bacterial colonies growing on a spectinomycin-containing medium were selected for the presence of the silencing construct by colony PCR and sequencing. Recombinant plasmids were transferred into the AGL1+virG strain of *Agrobacterium tumefaciens* (Lazo et al., 1991) by electroporation, and transformed bacterial cells were selected on a medium containing 100 mg/ml^−1^ spectinomycin, 50 mg/ml^−1^ carbenicillin, and 50 mg/ml^−1^ chloramphenicol. Single colonies of *A. tumefaciens* were picked and the presence of the insert was confirmed by colony PCR. Ten-fold dilutions of overnight culture from single positive colonies were re-suspended in MSO medium (4.3 g/l MS basal salt mixture, 30 g/l sucrose, 0.4 mg/l thiamine, 100 mg/l myoinositol, pH 5.8) to a final OD_600_ of 0.5 and used for transformation.

The transformation procedure for tomato cotyledons was carried out similarly to the method described by Appiano et al. (2015).

Silencing efficiency was assessed, for each of the four constructs, on 10–20 T_1_ plants and on selected T_2_ lines by real-time qPCR, as described for the analysis of the *SlMLO* gene family expression in response to *O. neolycopersici*. In addition, the T_2_ lines were assessed for the presence of the NPTII marker gene and the 35S promoter by PCR, using the primer pair NPTII_Fw (5′ACTGGGCACAACAGACAATC3′)/NPTII_Rev (5′ TCGTCCTGCAGTTCATTCAG 3′) and 35S-Fw (5′-GCTCCTACAAATG CCATCA-3′)/35S-Rev (5′- GATAGTGG GATTGTGCGTCA-3′), and visualizing the products on agarose gel.

### Disease quantification on silenced lines

T_2_ lines originating from selfing of T_1_ plants showing high level of silencing were inoculated with *O. neolycopersici* (*On*) by spraying 4 weeks old plants with a suspension of conidiospores obtained from freshly sporulating leaves of heavily infected plants and adjusted to a final concentration of 4 × 10^4^ spores/ml. Inoculated plants were grown in a greenhouse compartment at 20 ± 2°C with 70 ± 15% relative humidity and day length of 16 h. Two weeks later, infected tissues from the third and fourth true leaf were visually scored and sampled. Plant and fungal DNAs were extracted by using the DNeasy DNA extraction kit (Qiagen). In total, 15 ng of DNA was used as template for amplification with the primer pair *On*-Fw (5′-CGCCAAAGACCTAACCAAAA-3′) and *On*-Rev (5′-AGCCAAGAGATCCGTTGTTG-3′), designed on *On*-specific internal transcribed spacer sequences (GenBank accession number EU047564). The tomato Ef1α primers (Supplementary Table 3) were used as reference to determine fungal biomass relative to host plant DNA by ΔΔC_*t*_ method.

### Disease tests for microscopic evaluation in histological study

Spores of the Wageningen isolate of *O. neolycopersici* grown in a climate chamber at 20 ± 1°C, with 70 ± 10% RH and a 16-h photoperiod were water-sprayed on the third leaf of 1-month old tomato plants of the susceptible tomato cv. MM, the resistant line Slmlo1 and transgenic plants of one T_2_ family selected by PCR for the presence of the NPTII and 35S marker genes of the RNAi::*SlMLO1* silencing construct. The concentration of the spore suspension was 3 × 10^5^ conidia ml^−1^. After 65 h, a 4 cm^2^ segment was cut from the inoculated leaves. Three samples were taken from four plants of each genotype and from five plants of the T_2_ family, bleached in a 1:3 (v/v) acetic acid/ethanol solution and 48 h later stained in 0.005% trypan blue as described by Pavan et al. (2008). For each genotype, a total of 90 infection units (IU), defined as a germinated spore that produced, at least, a primary appressorium, were counted. Observations were performed using a Zeiss Axiophot bright field microscope and pictures were taken with an Axiocam ERc5s. For each IU, the number of hyphae, the presence/absence of a primary and secondary haustoria and presence/absence of papillae were recorded.

## Author contributions

Conceived and designed the experiments: ZZ, MA, SP, and VB. Performed the experiments: MA, ZZ, VB. Analyzed the data: MA, ZZ, VB. Contributed reagents/materials/ analysis tools: LR, RV. Wrote and edited the paper: SP, MA, ZZ, AW, and VB.

## Funding

The work of ZZ is supported by the Chinese Academy of Agricultural Sciences Fundamental Research Budget Increment Project (Grant No. 2015ZL008), The Agricultural Science and Technology Innovation Program (Grant No. CAAS-ASTIP-2013-IVFCAAS) and the Merit-based Scientific Research Foundation of the State Ministry of Human Resources and Social Security of China for Returned Overseas Chinese Scholars (Grant No. 2015-192). The work of SP, VB, and LR was supported by the Italian Ministry of University and Research (GenHORT project).

### Conflict of interest statement

The authors declare that the research was conducted in the absence of any commercial or financial relationships that could be construed as a potential conflict of interest.
