# Supplementary material for: Genome-Wide Study of the Tomato SlMLO Gene Family and Its Functional Characterization in Response to the Powdery Mildew Fungus Oidium neolycopersici
Source: Front Plant Sci. 2016 Apr 6;7:380. doi: 10.3389/fpls.2016.00380 (PMC4986958; doi:10.3389/fpls.2016.00380)
Supplement: Supplementary file 1 [file DataSheet1.pdf]

```
                                        20                            40
gDNA_Solyc04g049090  GACTTATTGT TTGAAACTTG CAACTACAAT TCTTCTTTAT CATCATTTGA 50
cDNA_Solyc04g049090  GACTTATTGT TTGAAACTTG CAACTACAAT TCTTCTTTAT CATCATTTGA 50
         SlMLO1_LEAF ---------- ---------- ---------- ---------- ---------- -
         SlMLO1_ROOT ---------- ---------- ---------- ---------- ---------- -
       SlMLO1_FLOWER ---------- ---------- ---------- ---------- ---------- -
                          60                           80                   100
gDNA_Solyc04g049090  CATTTCCCCT TCTTCTTATT CCTATTTTTA TTTAAATATA GGAATATTTC 100
cDNA_Solyc04g049090  CATTTCCCCT TCTTCTTATT CCTATTTTTA TTTAAATATA GGAATATTTC 100
         SlMLO1_LEAF ---------- ---------- ---------- ---------- ---------- -
         SlMLO1_ROOT ---------- ---------- ---------- ---------- ---------- -
       SlMLO1_FLOWER ---------- ---------- ---------- ---------- ---------- -
                                        120                           140
gDNA_Solyc04g049090  TTTCAAAGGA AGAAAATATA TATTTCCTTC AACACCACTA TATATAGACT 150
cDNA_Solyc04g049090  TTTCAAAGGA AGAAAATATA TATTTCCTTC AACACCACTA TATATAGACT 150
         SlMLO1_LEAF ---------- ---------- ---------- ---------- ---------- -
         SlMLO1_ROOT ---------- ---------- ---------- ---------- ---------- -
       SlMLO1_FLOWER ---------- ---------- ---------- ---------- ---------- -
                          160                          180                  200
gDNA_Solyc04g049090  TAATTCCATA ATCCTGTTAA TTTAATTGAT GGCTAAAGAA CGGTCTATGG 200
cDNA_Solyc04g049090  TAATTCCATA ATCCTGTTAA TTTAATTGAT GGCTAAAGAA CGGTCTATGG 200
         SlMLO1_LEAF ---------- ---------- ---------- ---------- ------ATGG 4
         SlMLO1_ROOT ---------- ---------- ---------- ---------- ------ATGG 4
       SlMLO1_FLOWER ---------- ---------- ---------- ---------- ------ATGG 4
                                        220                           240
gDNA_Solyc04g049090  AGGCAACCCC TACGTGGGCA ATTGCTGTGG TTTGCTTCAT CTTGCTCGCT 250
cDNA_Solyc04g049090  AGGCAACCCC TACGTGGGCA ATTGCTGTGG TTTGCTTCAT CTTGCTCGCT 250
         SlMLO1_LEAF AGGCAACCCC TACGTGGGCA ATTGCTGTGG TTTGCTTCAT CTTGCTCGCT 54
         SlMLO1_ROOT AGGCAACCCC TACGTGGGCA ATTGCTGTGG TTTGCTTCAT CTTGCTCGCT 54
       SlMLO1_FLOWER AGGCAACCCC TACGTGGGCA ATTGCTGCGG TTTGCTTCAT CTTGCTCGCT 54
                          260                          280                  300
gDNA_Solyc04g049090  ATTTCTATTT TTATTGAACA AATTATTCAT CACATTGGAG AGGTACCTAC 300
cDNA_Solyc04g049090  ATTTCTATTT TTATTGAACA AATTATTCAT CACATTGGAG AG-------- 292
         SlMLO1_LEAF ATTTCTATTT TTATTGAACA AATTATTCAT CACATTGGAG AG-------- 96
         SlMLO1_ROOT ATTTCTATTT TTATTGAACA AATTATTCAT CACATTGGAG AG-------- 96
       SlMLO1_FLOWER ATTTCTATTT TTATTGAACA AATTATTCAT CACATTGGAG AG-------- 96
                                        320                           340
gDNA_Solyc04g049090  TATATAATTT CAAAATCTTT TACTAGATCG ATATGAACAA AATATGCTTA 350
cDNA_Solyc04g049090  ---------- ---------- ---------- ---------- ---------- 292
         SlMLO1_LEAF ---------- ---------- ---------- ---------- ---------- 96
         SlMLO1_ROOT ---------- ---------- ---------- ---------- ---------- 96
       SlMLO1_FLOWER ---------- ---------- ---------- ---------- ---------- 96
                          360                          380                  400
gDNA_Solyc04g049090  TTAATTGTTG GGAAAACCAT TTTCTTTTTT AGTGGTTACT GGAAAAGCGG 400
cDNA_Solyc04g049090  ---------- ---------- ---------- --TGGTTACT GGAAAAGCGG 310
         SlMLO1_LEAF ---------- ---------- ---------- --TGGTTACT GGAAAAGCGG 114
         SlMLO1_ROOT ---------- ---------- ---------- --TGGTTACT GGAAAAGCGG 114
       SlMLO1_FLOWER ---------- ---------- ---------- --TGGTTACT GGAAAAGCGG 114
                                        420                           440
gDNA_Solyc04g049090  AAAAAGTCTC TATATGAAGC ACTTGAAAAG ATCAAAGCTG GTAAATTCAA 450
cDNA_Solyc04g049090  AAAAAGTCTC TATATGAAGC ACTTGAAAAG ATCAAAGCTG ---------- 350
         SlMLO1_LEAF AAAAAGTCTC TATATGAAGC ACTTGAAAAG ATCAAAGCTG ---------- 154
         SlMLO1_ROOT AAAAAGTCTC TATATGAAGC ACTTGAAAAG ATCAAAGCTG ---------- 154
       SlMLO1_FLOWER AAAAAGTCTC TATATGAAGC ACTTGAAAAG ATCAAAGCTG ---------- 154
                          460                          480                  500
gDNA_Solyc04g049090  AATACAGTCT TGTATTATAA AATAATATAG TTTAAAGATG GATCATACTC 500
cDNA_Solyc04g049090  ---------- ---------- ---------- ---------- ---------- 350
         SlMLO1_LEAF ---------- ---------- ---------- ---------- ---------- 154
         SlMLO1_ROOT ---------- ---------- ---------- ---------- ---------- 154
       SlMLO1_FLOWER ---------- ---------- ---------- ---------- ---------- 154
                                        520                           540
gDNA_Solyc04g049090  ATGACATTTT TGGTGTGTGT GTGTCGGACA GAACTTATGC TGTTGGGATT 550
cDNA_Solyc04g049090  ---------- ---------- ---------- -AACTTATGC TGTTGGGATT 369
         SlMLO1_LEAF ---------- ---------- ---------- -AACTTATGC TGTTGGGATT 173
         SlMLO1_ROOT ---------- ---------- ---------- -AACTTATGC TGTTGGGATT 173
       SlMLO1_FLOWER ---------- ---------- ---------- -AACTTATGC TGTTGGGATT 173
                          560                          580                  600
gDNA_Solyc04g049090  CTTATCACTG TTGTTGACAG TGTTGCAAGA TCCAGTTTCT AACTTATGTG 600
cDNA_Solyc04g049090  CTTATCACTG TTGTTGACAG TGTTGCAAGA TCCAGTTTCT AACTTATGTG 419
         SlMLO1_LEAF CTTATCACTG TTGTTGACAG TGTTGCAAGA TCCAGTTTCT AACTTATGTG 223
         SlMLO1_ROOT CTTATCACTG TTGTTGACAG TGTTGCAAGA TCCAGTTTCT AACTTATGTG 223
       SlMLO1_FLOWER CTTATCACTG TTGTTGACAG TGTTGCAAGA TCCAGTTTCT AACTTATGTG 223
                                        620                           640
gDNA_Solyc04g049090  TCCCCAAGAG TGTTGGTTAT TCATGGCATC CTTGTATGGC AAAGGAAGAT 650
cDNA_Solyc04g049090  TCCCCAAGAG TGTTGGTTAT TCATGGCATC CTTGTATGGC AAAGGAAGAT 469
         SlMLO1_LEAF TCCCCAAGAG TGTTGGTTAT TCATGGCATC CTTGTATGGC AAAGGAAGAT 273
         SlMLO1_ROOT TCCCCAAGAG TGTTGGTTAT TCATGGCATC CTTGTATGGC AAAGGAAGAT 273
       SlMLO1_FLOWER TCCCCAAGAG TGTTGGTTAT TCATGGCATC CTTGTATGGC AAAGGAAGAT 273
                          660                          680                  700
gDNA_Solyc04g049090  GCCAAGTCTG AGTATGATGA CCCTTGTCTA CCAAAGGTAC GCAATATACG 700
cDNA_Solyc04g049090  GCCAAGTCTG AGTATGATGA CCCTTGTCTA CCAAAGG--- ---------- 506
         SlMLO1_LEAF GCCAAGTCTG AGTATGATGA CCCTTGTCTA CCAAAGG--- ---------- 310
         SlMLO1_ROOT GCCAAGTCTG AGTATGATGA CCCTTGCCTA CCAAAGG--- ---------- 310
       SlMLO1_FLOWER GCCAAGTCTG AGTATGATGA CCCTTGTCTA CCAAAGG--- ---------- 310
```

```
                                        720                           740
gDNA_Solyc04g049090  ATAAGTTAAG CTCAAGTATC ATATTTTTAT ATGATATTAG AGGTCGTAGG  750
cDNA_Solyc04g049090  ---------- ---------- ---------- ---------- ----------  506
        SlMLO1_LEAF  ---------- ---------- ---------- ---------- ----------  310
        SlMLO1_ROOT  ---------- ---------- ---------- ---------- ----------  310
      SlMLO1_FLOWER  ---------- ---------- ---------- ---------- ----------  310
                              760                           780                800
gDNA_Solyc04g049090  ATTAAATTTC ACTACCACCT AACTTAAAAA GAATTTTTTA CGTGTTTAAT  800
cDNA_Solyc04g049090  ---------- ---------- ---------- ---------- ----------  506
        SlMLO1_LEAF  ---------- ---------- ---------- ---------- ----------  310
        SlMLO1_ROOT  ---------- ---------- ---------- ---------- ----------  310
      SlMLO1_FLOWER  ---------- ---------- ---------- ---------- ----------  310
                                        820                           840
gDNA_Solyc04g049090  CATCCATAAA AAGGAATTAG GTCTACATGT GAGGGGCATC ATTGAGAATA  850
cDNA_Solyc04g049090  ---------- ---------- ---------- ---------- ----------  506
        SlMLO1_LEAF  ---------- ---------- ---------- ---------- ----------  310
        SlMLO1_ROOT  ---------- ---------- ---------- ---------- ----------  310
      SlMLO1_FLOWER  ---------- ---------- ---------- ---------- ----------  310
                              860                           880                900
gDNA_Solyc04g049090  TTATTAACTA ATAAAAAGTA TGTGATCACA TAGGGATGGT ATTTCACATC  900
cDNA_Solyc04g049090  ---------- ---------- ---------- ---------- ----------  506
        SlMLO1_LEAF  ---------- ---------- ---------- ---------- ----------  310
        SlMLO1_ROOT  ---------- ---------- ---------- ---------- ----------  310
      SlMLO1_FLOWER  ---------- ---------- ---------- ---------- ----------  310
                                        920                           940
gDNA_Solyc04g049090  ATTCATTTAG GGATGACAAC TTTTTATCTG AATTAATTTT TGAGGTAGAA  950
cDNA_Solyc04g049090  ---------- ---------- ---------- ---------- ----------  506
        SlMLO1_LEAF  ---------- ---------- ---------- ---------- ----------  310
        SlMLO1_ROOT  ---------- ---------- ---------- ---------- ----------  310
      SlMLO1_FLOWER  ---------- ---------- ---------- ---------- ----------  310
                              960                           980              1,000
gDNA_Solyc04g049090  TCAAAATTTA TTTCTTTGCG GTTTCTTATG AAATTTAATT TTAAATATGC  1000
cDNA_Solyc04g049090  ---------- ---------- ---------- ---------- ----------  506
        SlMLO1_LEAF  ---------- ---------- ---------- ---------- ----------  310
        SlMLO1_ROOT  ---------- ---------- ---------- ---------- ----------  310
      SlMLO1_FLOWER  ---------- ---------- ---------- ---------- ----------  310
                                      1,020                         1,040
gDNA_Solyc04g049090  AGGGAAAAGT GCAATTTGCA TCTTCATATG CAATACACCA GCTCCATATC  1050
cDNA_Solyc04g049090  ---GAAAAGT GCAATTTGCA TCTTCATATG CAATACACCA GCTCCATATC  553
        SlMLO1_LEAF  ---GAAAAGT GCAATTTGCA TCTTCATATG CAATACACCA GCTCCATATC  357
        SlMLO1_ROOT  ---GAAAAGT GCAATTTGCA TCTTCATATG CAATACACCA GCTCCATATC  357
      SlMLO1_FLOWER  ---GAAAAGT GCAATTTGCA TCTTCATATG CAATACACCA GCTCCATATC  357
                            1,060                         1,080              1,100
gDNA_Solyc04g049090  TTCATCTTTG TATTGGCAGT TGCTCATGTA TTGTACTGTA TAGCAACTTT  1100
cDNA_Solyc04g049090  TTCATCTTTG TATTGGCAGT TGCTCATGTA TTGTACTGTA TAGCAACTTT  603
        SlMLO1_LEAF  TTCATCTTTG TATTGGCAGT TGCTCATGTA TTGTACTGTA TAGCAACTTT  407
        SlMLO1_ROOT  TTCATCTTTG CATTGGCAGT TGCTCATGTA TTGTACTGTA TAGCAACTCT  407
      SlMLO1_FLOWER  TTCATCTTTG TATTGGCAGT TGCTCATGTA TTGTACTGTA TAGCAACTTT  407
                                      1,120                         1,140
gDNA_Solyc04g049090  TGCTTTGGGC AGGCTAAAGG TTTATATATA TATATATCTA TATTCATCTT  1150
cDNA_Solyc04g049090  TGCTTTGGGC AGGCTAAAG- ---------- ---------- ----------  622
        SlMLO1_LEAF  TGCTTTGGGC AGGCTAAAG- ---------- ---------- ----------  426
        SlMLO1_ROOT  TGCTTTGGGC AGGCTAAAG- ---------- ---------- ----------  426
      SlMLO1_FLOWER  TGCTTTGGGC AGGCTAAAG- ---------- ---------- ----------  426
                            1,160                         1,180              1,200
gDNA_Solyc04g049090  TGGTCATTAA TCCATATGAT TTTCTTTTTC TCTTCTTTGG TCAATAATTT  1200
cDNA_Solyc04g049090  ---------- ---------- ---------- ---------- ----------  622
        SlMLO1_LEAF  ---------- ---------- ---------- ---------- ----------  426
        SlMLO1_ROOT  ---------- ---------- ---------- ---------- ----------  426
      SlMLO1_FLOWER  ---------- ---------- ---------- ---------- ----------  426
                                      1,220                         1,240
gDNA_Solyc04g049090  TCTATCTTAA TTTGTAGATG AGAAAATGGA GGGCATGGGA GGATGAAACA  1250
cDNA_Solyc04g049090  ---------- -------ATG AGAAAATGGA GGGCATGGGA GGATGAAACA  655
        SlMLO1_LEAF  ---------- -------ATG AGAAAATGGA GGGCATGGGA GGATGAAACA  459
        SlMLO1_ROOT  ---------- -------ATG AGAAAATGGA GGGCATGGGA GGATGAAACA  459
      SlMLO1_FLOWER  ---------- -------ATG AGAAAATGGA GGGCATGGGA GGATGAAACA  459
                            1,260                         1,280              1,300
gDNA_Solyc04g049090  AAAACAATGG AGTACCAATT CTACAACGGT TAGTATACAA GAATTTGAAT  1300
cDNA_Solyc04g049090  AAAACAATGG AGTACCAATT CTACAACG-- ---------- ----------  683
        SlMLO1_LEAF  AAAACAATGG AGTACCAATT CTACAACG-- ---------- ----------  487
        SlMLO1_ROOT  AAAACAATGG AGTACCAATT CTACAACG-- ---------- ----------  487
      SlMLO1_FLOWER  AAAACAATGG AGTACCAATT CTACAACG-- ---------- ----------  487
                                      1,320                         1,340
gDNA_Solyc04g049090  ATTATTTGAA TATTTGTCAT TTAAAATTAA TTAAGAAATT TTACGTCATT  1350
cDNA_Solyc04g049090  ---------- ---------- ---------- ---------- ----------  683
        SlMLO1_LEAF  ---------- ---------- ---------- ---------- ----------  487
        SlMLO1_ROOT  ---------- ---------- ---------- ---------- ----------  487
      SlMLO1_FLOWER  ---------- ---------- ---------- ---------- ----------  487
                            1,360                         1,380              1,400
gDNA_Solyc04g049090  AAAGTATAGA AGTATACATC ATCTCAAGCC TTGTAAATAA TATTCCTTCC  1400
cDNA_Solyc04g049090  ---------- ---------- ---------- ---------- ----------  683
        SlMLO1_LEAF  ---------- ---------- ---------- ---------- ----------  487
        SlMLO1_ROOT  ---------- ---------- ---------- ---------- ----------  487
      SlMLO1_FLOWER  ---------- ---------- ---------- ---------- ----------  487
```

```
                                      1,420                    1,440
gDNA_Solyc04g049090  CTCCATTTAA AAACTAATAA TTTAGTTTGA GAATTCAAGA AATAAAAAAG 1450
cDNA_Solyc04g049090  ---------- ---------- ---------- ---------- ---------- 683
        SlMLO1_LEAF  ---------- ---------- ---------- ---------- ---------- 487
        SlMLO1_ROOT  ---------- ---------- ---------- ---------- ---------- 487
      SlMLO1_FLOWER  ---------- ---------- ---------- ---------- ---------- 487
                           1,460                    1,480                    1,500
gDNA_Solyc04g049090  GACTTTTGTA ATTTTATAAT AAAATTATAT CACATGTGTT AAAATATCTT 1500
cDNA_Solyc04g049090  ---------- ---------- ---------- ---------- ---------- 683
        SlMLO1_LEAF  ---------- ---------- ---------- ---------- ---------- 487
        SlMLO1_ROOT  ---------- ---------- ---------- ---------- ---------- 487
      SlMLO1_FLOWER  ---------- ---------- ---------- ---------- ---------- 487
                                      1,520                    1,540
gDNA_Solyc04g049090  TTACTCTTCT TATTTTAAAT ATATTATGTA AAAAATTTAA ATTAAAATAT 1550
cDNA_Solyc04g049090  ---------- ---------- ---------- ---------- ---------- 683
        SlMLO1_LEAF  ---------- ---------- ---------- ---------- ---------- 487
        SlMLO1_ROOT  ---------- ---------- ---------- ---------- ---------- 487
      SlMLO1_FLOWER  ---------- ---------- ---------- ---------- ---------- 487
                           1,560                    1,580                    1,600
gDNA_Solyc04g049090  CATCAAAAAT GATAAAAAAT TATTTTATCG TATAAAGACA CTCAAAAGAA 1600
cDNA_Solyc04g049090  ---------- ---------- ---------- ---------- ---------- 683
        SlMLO1_LEAF  ---------- ---------- ---------- ---------- ---------- 487
        SlMLO1_ROOT  ---------- ---------- ---------- ---------- ---------- 487
      SlMLO1_FLOWER  ---------- ---------- ---------- ---------- ---------- 487
                                      1,620                    1,640
gDNA_Solyc04g049090  AATCTGTCAT TATTTTTGAA ACTATAAAAG TATGAAAAGA CAACTCTGCC 1650
cDNA_Solyc04g049090  ---------- ---------- ---------- ---------- ---------- 683
        SlMLO1_LEAF  ---------- ---------- ---------- ---------- ---------- 487
        SlMLO1_ROOT  ---------- ---------- ---------- ---------- ---------- 487
      SlMLO1_FLOWER  ---------- ---------- ---------- ---------- ---------- 487
                           1,660                    1,680                    1,700
gDNA_Solyc04g049090  CCTTAATCAT TAGATATTTA GAACCTTAGA CCTAAGGTGG AGTTGATTCA 1700
cDNA_Solyc04g049090  ---------- ---------- ---------- ---------- ---------- 683
        SlMLO1_LEAF  ---------- ---------- ---------- ---------- ---------- 487
        SlMLO1_ROOT  ---------- ---------- ---------- ---------- ---------- 487
      SlMLO1_FLOWER  ---------- ---------- ---------- ---------- ---------- 487
                                      1,720                    1,740
gDNA_Solyc04g049090  AAAATAGAAA TATATTAAAT GCTATTAAAA AATAAGCCCA CAAACATTTC 1750
cDNA_Solyc04g049090  ---------- ---------- ---------- ---------- ---------- 683
        SlMLO1_LEAF  ---------- ---------- ---------- ---------- ---------- 487
        SlMLO1_ROOT  ---------- ---------- ---------- ---------- ---------- 487
      SlMLO1_FLOWER  ---------- ---------- ---------- ---------- ---------- 487
                           1,760                    1,780                    1,800
gDNA_Solyc04g049090  AATTTAGCTA CAAATTGATG TGACATATAT CTAAAAAGTT TGGACACGTC 1800
cDNA_Solyc04g049090  ---------- ---------- ---------- ---------- ---------- 683
        SlMLO1_LEAF  ---------- ---------- ---------- ---------- ---------- 487
        SlMLO1_ROOT  ---------- ---------- ---------- ---------- ---------- 487
      SlMLO1_FLOWER  ---------- ---------- ---------- ---------- ---------- 487
                                      1,820                    1,840
gDNA_Solyc04g049090  TATTGTTATT TATTTTTTAA ATATTCTTGG ACCCCAAGTG GTAGCTATTG 1850
cDNA_Solyc04g049090  ---------- ---------- ---------- ---------- ---------- 683
        SlMLO1_LEAF  ---------- ---------- ---------- ---------- ---------- 487
        SlMLO1_ROOT  ---------- ---------- ---------- ---------- ---------- 487
      SlMLO1_FLOWER  ---------- ---------- ---------- ---------- ---------- 487
                           1,860                    1,880                    1,900
gDNA_Solyc04g049090  CCTATTGGAT AAGTAATGTG AATAAAGGGA ATCATTTGCA AGTTGCACCT 1900
cDNA_Solyc04g049090  ---------- ---------- ---------- ---------- ---------- 683
        SlMLO1_LEAF  ---------- ---------- ---------- ---------- ---------- 487
        SlMLO1_ROOT  ---------- ---------- ---------- ---------- ---------- 487
      SlMLO1_FLOWER  ---------- ---------- ---------- ---------- ---------- 487
                                      1,920                    1,940
gDNA_Solyc04g049090  TTTAATACCA AATTAATTGA CCGACACTTC ATTCGTACAT TCTTAGTTTA 1950
cDNA_Solyc04g049090  ---------- ---------- ---------- ---------- ---------- 683
        SlMLO1_LEAF  ---------- ---------- ---------- ---------- ---------- 487
        SlMLO1_ROOT  ---------- ---------- ---------- ---------- ---------- 487
      SlMLO1_FLOWER  ---------- ---------- ---------- ---------- ---------- 487
                           1,960                    1,980                    2,000
gDNA_Solyc04g049090  TTTCGATTAG TATATCACTA TTTTTATGAT GTAAATATAA AAGAATTTTA 2000
cDNA_Solyc04g049090  ---------- ---------- ---------- ---------- ---------- 683
        SlMLO1_LEAF  ---------- ---------- ---------- ---------- ---------- 487
        SlMLO1_ROOT  ---------- ---------- ---------- ---------- ---------- 487
      SlMLO1_FLOWER  ---------- ---------- ---------- ---------- ---------- 487
                                      2,020                    2,040
gDNA_Solyc04g049090  TAATTTATAC TACTATTAAT ATAATATGTA ATTAATATAA TTTTAAGAAA 2050
cDNA_Solyc04g049090  ---------- ---------- ---------- ---------- ---------- 683
        SlMLO1_LEAF  ---------- ---------- ---------- ---------- ---------- 487
        SlMLO1_ROOT  ---------- ---------- ---------- ---------- ---------- 487
      SlMLO1_FLOWER  ---------- ---------- ---------- ---------- ---------- 487
                           2,060                    2,080                    2,100
gDNA_Solyc04g049090  TTAATTAAAT TGACTTTTTA TTTTTTTTAA AAAAACATGG CAATTACAAA 2100
cDNA_Solyc04g049090  ---------- ---------- ---------- ---------- ---------- 683
        SlMLO1_LEAF  ---------- ---------- ---------- ---------- ---------- 487
        SlMLO1_ROOT  ---------- ---------- ---------- ---------- ---------- 487
      SlMLO1_FLOWER  ---------- ---------- ---------- ---------- ---------- 487
```

```
                                        2,120                         2,140
                                          |                             |
gDNA_Solyc04g049090  TAAACTGAGG GAGTACTTAT TATTGCTAGC TCTCTTTCTA GTTTGGTCCC 2150
cDNA_Solyc04g049090  ---------- ---------- ---------- ---------- ---------- 683
         SlMLO1_LEAF ---------- ---------- ---------- ---------- ---------- 487
         SlMLO1_ROOT ---------- ---------- ---------- ---------- ---------- 487
       SlMLO1_FLOWER ---------- ---------- ---------- ---------- ---------- 487
                          2,160                         2,180                2,200
                            |                             |                    |
gDNA_Solyc04g049090  CTTTGAGGTT TGACTTACTT ATTTTTCCAT TACTTGTTTG GTTTTCTTTG 2200
cDNA_Solyc04g049090  ---------- ---------- ---------- ---------- ---------- 683
         SlMLO1_LEAF ---------- ---------- ---------- ---------- ---------- 487
         SlMLO1_ROOT ---------- ---------- ---------- ---------- ---------- 487
       SlMLO1_FLOWER ---------- ---------- ---------- ---------- ---------- 487
                                        2,220                         2,240
                                          |                             |
gDNA_Solyc04g049090  GACAATTTTA TATTCTAACT TGGCAGTTTA CACGTTAAGA TTATAAATAA 2250
cDNA_Solyc04g049090  ---------- ---------- ---------- ---------- ---------- 683
         SlMLO1_LEAF ---------- ---------- ---------- ---------- ---------- 487
         SlMLO1_ROOT ---------- ---------- ---------- ---------- ---------- 487
       SlMLO1_FLOWER ---------- ---------- ---------- ---------- ---------- 487
                          2,260                         2,280                2,300
                            |                             |                    |
gDNA_Solyc04g049090  AATATATTTT GAAATTTAAA ATTATACGAA GAAAATTAAT CATGAGAAGT 2300
cDNA_Solyc04g049090  ---------- ---------- ---------- ---------- ---------- 683
         SlMLO1_LEAF ---------- ---------- ---------- ---------- ---------- 487
         SlMLO1_ROOT ---------- ---------- ---------- ---------- ---------- 487
       SlMLO1_FLOWER ---------- ---------- ---------- ---------- ---------- 487
                                        2,320                         2,340
                                          |                             |
gDNA_Solyc04g049090  CAAAATGAAA GAAAATTAAA ATTATTATTG TTGACTTTAT TTATTGGTAT 2350
cDNA_Solyc04g049090  ---------- ---------- ---------- ---------- ---------- 683
         SlMLO1_LEAF ---------- ---------- ---------- ---------- ---------- 487
         SlMLO1_ROOT ---------- ---------- ---------- ---------- ---------- 487
       SlMLO1_FLOWER ---------- ---------- ---------- ---------- ---------- 487
                          2,360                         2,380                2,400
                            |                             |                    |
gDNA_Solyc04g049090  TTGATGGTTG CAGACCCTGA GAGATTCAGA TTTGCAAGGG AGACCTCGTT 2400
cDNA_Solyc04g049090  ---------- ---ACCCTGA GAGATTCAGA TTTGCAAGGG AGACCTCGTT 720
         SlMLO1_LEAF ---------- ---ACCCTGA GAGATTCAGA TTTGCAAGGG AGACCTCGTT 524
         SlMLO1_ROOT ---------- ---ACCCTGA GAGATTCAGA TTTGCAAGGG AGACCTCGTT 524
       SlMLO1_FLOWER ---------- ---ACCCTGA GAGATTCAGA TTTGCAAGGG AGACCTCGTT 524
                                        2,420                         2,440
                                          |                             |
gDNA_Solyc04g049090  TGGACGTAGG CATTTGCATT TCTGGAGCAA GTCCCCCGTG TTGCTCTCGA 2450
cDNA_Solyc04g049090  TGGACGTAGG CATTTGCATT TCTGGAGCAA GTCCCCCGTG TTGCTCTCGA 770
         SlMLO1_LEAF TGGACGTAGG CATTTGCATT TCTGGAGCAA GTCCCCCGTG TTGCTCTCGA 574
         SlMLO1_ROOT TGGACGTAGG CATTTGCATT TCTGGAGCAA GTCCCCCGTG TTGCTCTCGA 574
       SlMLO1_FLOWER TGGACGTAGG CATTTGCATT TCTGGAGCAA GTCCCCCGTG TTGCTCTCGA 574
                          2,460                         2,480                2,500
                            |                             |                    |
gDNA_Solyc04g049090  TAGTGAGTCC ATCCATTATA TATAATATAA TATTATTTTT ATTACGTTAT 2500
cDNA_Solyc04g049090  TA-------- ---------- ---------- ---------- ---------- 772
         SlMLO1_LEAF TA-------- ---------- ---------- ---------- ---------- 576
         SlMLO1_ROOT TA-------- ---------- ---------- ---------- ---------- 576
       SlMLO1_FLOWER TA-------- ---------- ---------- ---------- ---------- 576
                                        2,520                         2,540
                                          |                             |
gDNA_Solyc04g049090  AATTGTTATG ATTGAAATAA TTAGATTGAA TGAGTAAATA AGTCCTCAAA 2550
cDNA_Solyc04g049090  ---------- ---------- ---------- ---------- ---------- 772
         SlMLO1_LEAF ---------- ---------- ---------- ---------- ---------- 576
         SlMLO1_ROOT ---------- ---------- ---------- ---------- ---------- 576
       SlMLO1_FLOWER ---------- ---------- ---------- ---------- ---------- 576
                          2,560                         2,580                2,600
                            |                             |                    |
gDNA_Solyc04g049090  AAATTATGAA TAAAAAGATA AACGAGAAAC ATATTGAAAG ATACAAATTA 2600
cDNA_Solyc04g049090  ---------- ---------- ---------- ---------- ---------- 772
         SlMLO1_LEAF ---------- ---------- ---------- ---------- ---------- 576
         SlMLO1_ROOT ---------- ---------- ---------- ---------- ---------- 576
       SlMLO1_FLOWER ---------- ---------- ---------- ---------- ---------- 576
                                        2,620                         2,640
                                          |                             |
gDNA_Solyc04g049090  ACGTGATTCA GTCAATCGAT TTATATCCAT AAGAGAAATG AGCAATCTAC 2650
cDNA_Solyc04g049090  ---------- ---------- ---------- ---------- ---------- 772
         SlMLO1_LEAF ---------- ---------- ---------- ---------- ---------- 576
         SlMLO1_ROOT ---------- ---------- ---------- ---------- ---------- 576
       SlMLO1_FLOWER ---------- ---------- ---------- ---------- ---------- 576
                          2,660                         2,680                2,700
                            |                             |                    |
gDNA_Solyc04g049090  TGTATAAATA TATATAAAAA AATATTAAGA AAAATAACGG TACAATTTAC 2700
cDNA_Solyc04g049090  ---------- ---------- ---------- ---------- ---------- 772
         SlMLO1_LEAF ---------- ---------- ---------- ---------- ---------- 576
         SlMLO1_ROOT ---------- ---------- ---------- ---------- ---------- 576
       SlMLO1_FLOWER ---------- ---------- ---------- ---------- ---------- 576
                                        2,720                         2,740
                                          |                             |
gDNA_Solyc04g049090  TCAAAATAAT AAGAAGTTAA TACTTGTGTC TAATCGTTTC TCCCCCGTAC 2750
cDNA_Solyc04g049090  ---------- ---------- ---------- ---------- ---------- 772
         SlMLO1_LEAF ---------- ---------- ---------- ---------- ---------- 576
         SlMLO1_ROOT ---------- ---------- ---------- ---------- ---------- 576
       SlMLO1_FLOWER ---------- ---------- ---------- ---------- ---------- 576
                          2,760                         2,780                2,800
                            |                             |                    |
gDNA_Solyc04g049090  AAAACTCAAA GAATGAACTT CTATATATTT CTGTTTATAG AGTTAAAAAA 2800
cDNA_Solyc04g049090  ---------- ---------- ---------- ---------- ---------- 772
         SlMLO1_LEAF ---------- ---------- ---------- ---------- ---------- 576
         SlMLO1_ROOT ---------- ---------- ---------- ---------- ---------- 576
       SlMLO1_FLOWER ---------- ---------- ---------- ---------- ---------- 576
```

```
                              2,820               2,840
gDNA_Solyc04g049090  AAATAAACTA TCCAAATATG AAAACATTAT TATTTTCCTT TAAAGAAACA 2850
cDNA_Solyc04g049090  ---------- ---------- ---------- ---------- ---------- 772
        SlMLO1_LEAF  ---------- ---------- ---------- ---------- ---------- 576
        SlMLO1_ROOT  ---------- ---------- ---------- ---------- ---------- 576
      SlMLO1_FLOWER  ---------- ---------- ---------- ---------- ---------- 576

                   2,860               2,880               2,900
gDNA_Solyc04g049090  AAAAGGAAAA CCCTTAAGAA ACTAGGGCAA ACAATATCCC ATATTTATCA 2900
cDNA_Solyc04g049090  ---------- ---------- ---------- ---------- ---------- 772
        SlMLO1_LEAF  ---------- ---------- ---------- ---------- ---------- 576
        SlMLO1_ROOT  ---------- ---------- ---------- ---------- ---------- 576
      SlMLO1_FLOWER  ---------- ---------- ---------- ---------- ---------- 576

                              2,920               2,940
gDNA_Solyc04g049090  TAAAATTTAT TACTAGTAGC ATAAATAATT ATTGTGTAAG GATTCATTAA 2950
cDNA_Solyc04g049090  ---------- ---------- ---------- ---------- ---------- 772
        SlMLO1_LEAF  ---------- ---------- ---------- ---------- ---------- 576
        SlMLO1_ROOT  ---------- ---------- ---------- ---------- ---------- 576
      SlMLO1_FLOWER  ---------- ---------- ---------- ---------- ---------- 576

                   2,960               2,980               3,000
gDNA_Solyc04g049090  ACTGATATTT TTGTGATTCT ATTTACAGGT TTGTTTCTTT CGGCAATTCT 3000
cDNA_Solyc04g049090  ---------- ---------- --------GT TTGTTTCTTT CGGCAATTCT 794
        SlMLO1_LEAF  ---------- ---------- --------GT TTGTTTCTTT CGGCAATTCT 598
        SlMLO1_ROOT  ---------- ---------- --------GT TTGTTTCTTT CGGCAATTCT 598
      SlMLO1_FLOWER  ---------- ---------- --------GT TTGTTTCTTT CGGCAATTCT 598

                              3,020               3,040
gDNA_Solyc04g049090  TCTCATCAGT TGCAAAAGTT GACTATTTAA CCCTTAGACA TGGGTTCATG 3050
cDNA_Solyc04g049090  TCTCATCAGT TGCAAAAGTT GACTATTTAA CCCTTAGACA TGGGTTCATG 844
        SlMLO1_LEAF  TCTCATCAGT TGCAAAAGTT GACTATTTAA CCCTTAGACA TGGGTTCATG 648
        SlMLO1_ROOT  TCTCATCAGT TGCAAAAGTT GACTATTTAA CCCTTAGACA TGGGTTCATG 648
      SlMLO1_FLOWER  TCTCATCAGT TGCAAAAGTT GACTATTTAA CCCTTAGACA TGGGTTCATG 648

                   3,060               3,080               3,100
gDNA_Solyc04g049090  ATGGTAAGTC AATCTAGCAC CAATATAACG TGAAATTATA CGATATCCAA 3100
cDNA_Solyc04g049090  ATGG------ ---------- ---------- ---------- ---------- 848
        SlMLO1_LEAF  ATGG------ ---------- ---------- ---------- ---------- 652
        SlMLO1_ROOT  ATGG------ ---------- ---------- ---------- ---------- 652
      SlMLO1_FLOWER  ATGG------ ---------- ---------- ---------- ---------- 652

                              3,120               3,140
gDNA_Solyc04g049090  GATACTATTG AAAAATAATT ATTTTTTTTG GATCCATGTA ATTAACTGCA 3150
cDNA_Solyc04g049090  ---------- ---------- ---------- ---------- ---------- 848
        SlMLO1_LEAF  ---------- ---------- ---------- ---------- ---------- 652
        SlMLO1_ROOT  ---------- ---------- ---------- ---------- ---------- 652
      SlMLO1_FLOWER  ---------- ---------- ---------- ---------- ---------- 652

                   3,160               3,180               3,200
gDNA_Solyc04g049090  GGCACATTTA ACTCCACAAA ATCAAAATAA TTTTGATTTT CAATTATACA 3200
cDNA_Solyc04g049090  --CACATTTA ACTCCACAAA ATCAAAATAA TTTTGATTTT CAATTATACA 896
        SlMLO1_LEAF  --CACATTTA ACTCCACAAA ATCAAAATAA TTTTGATTTT CAATTATACA 700
        SlMLO1_ROOT  --CACATTTA ACTCCACAAA ATCAAAATAA TTTTGATTTT CAATTATACA 700
      SlMLO1_FLOWER  --CACATTTA ACTCCACAAA ATCAAAATAA TTTTGATTTT CAATTATACA 700

                              3,220               3,240
gDNA_Solyc04g049090  TTAACAGAGC AGTTGACAAA GACTTCAAAG TTGTTGTTGG AATAAGGTTT 3250
cDNA_Solyc04g049090  TTAACAGAGC AGTTGACAAA GACTTCAAAG TTGTTGTTGG AATAAG---- 942
        SlMLO1_LEAF  TTAACAGAGC AGTTGACAAA GACTTCAAAG TTGTTGTTGG AATAAG---- 746
        SlMLO1_ROOT  TTAACAGAGC AGTTGACAGA GACTTCAAAG TTGTTGTTGG AATAAG---- 746
      SlMLO1_FLOWER  TTAACAGAGC AGTTGACAAA GACTTCAAAG TTGTTGTTGG AATAAG---- 746

                   3,260               3,280               3,300
gDNA_Solyc04g049090  ACATCTTTCT TCATTGGCAT AATACCTAAT TTGACTTTAA ATTTTTACTT 3300
cDNA_Solyc04g049090  ---------- ---------- ---------- ---------- ---------- 942
        SlMLO1_LEAF  ---------- ---------- ---------- ---------- ---------- 746
        SlMLO1_ROOT  ---------- ---------- ---------- ---------- ---------- 746
      SlMLO1_FLOWER  ---------- ---------- ---------- ---------- ---------- 746

                              3,320               3,340
gDNA_Solyc04g049090  GACCTTCAAT TTTTATAATG CACAAACATA CACTTTACTT ATCAAACTTT 3350
cDNA_Solyc04g049090  ---------- ---------- ---------- ---------- ---------- 942
        SlMLO1_LEAF  ---------- ---------- ---------- ---------- ---------- 746
        SlMLO1_ROOT  ---------- ---------- ---------- ---------- ---------- 746
      SlMLO1_FLOWER  ---------- ---------- ---------- ---------- ---------- 746

                   3,360               3,380               3,400
gDNA_Solyc04g049090  TAAATAAATA AACACATGAA TCATACATGA CACAATACAC GTAAGATACC 3400
cDNA_Solyc04g049090  ---------- ---------- ---------- ---------- ---------- 942
        SlMLO1_LEAF  ---------- ---------- ---------- ---------- ---------- 746
        SlMLO1_ROOT  ---------- ---------- ---------- ---------- ---------- 746
      SlMLO1_FLOWER  ---------- ---------- ---------- ---------- ---------- 746

                              3,420               3,440
gDNA_Solyc04g049090  ACGTAGGATA AAAAATGACA CATAGAACAT GTGTGCCTAT TTGTTCGACT 3450
cDNA_Solyc04g049090  ---------- ---------- ---------- ---------- ---------- 942
        SlMLO1_LEAF  ---------- ---------- ---------- ---------- ---------- 746
        SlMLO1_ROOT  ---------- ---------- ---------- ---------- ---------- 746
      SlMLO1_FLOWER  ---------- ---------- ---------- ---------- ---------- 746

                   3,460               3,480               3,500
gDNA_Solyc04g049090  TTGTACAATT TTAAGTATCT ACTTGTGCAT ATCCAAAATT AAAAGACATA 3500
cDNA_Solyc04g049090  ---------- ---------- ---------- ---------- ---------- 942
        SlMLO1_LEAF  ---------- ---------- ---------- ---------- ---------- 746
        SlMLO1_ROOT  ---------- ---------- ---------- ---------- ---------- 746
      SlMLO1_FLOWER  ---------- ---------- ---------- ---------- ---------- 746
```

```
                                         3,520                          3,540
gDNA_Solyc04g049090  AATATAATTT AAAGCCAAAT TAAAGGATAT ATTTATGTAT TATGCCTTCT 3550
cDNA_Solyc04g049090  ---------- ---------- ---------- ---------- ---------- 942
         SlMLO1_LEAF ---------- ---------- ---------- ---------- ---------- 746
         SlMLO1_ROOT ---------- ---------- ---------- ---------- ---------- 746
       SlMLO1_FLOWER ---------- ---------- ---------- ---------- ---------- 746
                              3,560                          3,580               3,600
gDNA_Solyc04g049090  TTATTTCCTC AAGCAATTCT TCTTTTACTA TTAAAGCTAT ACCATGATAA 3600
cDNA_Solyc04g049090  ---------- ---------- ---------- ---------- ---------- 942
         SlMLO1_LEAF ---------- ---------- ---------- ---------- ---------- 746
         SlMLO1_ROOT ---------- ---------- ---------- ---------- ---------- 746
       SlMLO1_FLOWER ---------- ---------- ---------- ---------- ---------- 746
                                         3,620                          3,640
gDNA_Solyc04g049090  TTATAATAAC ACTATTTATG TTGCAGTCCT GCATTATGGC TCTTCACGGT 3650
cDNA_Solyc04g049090  ---------- ---------- ------TCCT GCATTATGGC TCTTCACGGT 966
         SlMLO1_LEAF ---------- ---------- ------TCCT GCATTATGGC TCTTCACGGT 770
         SlMLO1_ROOT ---------- ---------- ------TCCT GCATTATGGC TCTTCACGGT 770
       SlMLO1_FLOWER ---------- ---------- ------TCCT GCATTATGGC TCTTCACGGT 770
                              3,660                          3,680               3,700
gDNA_Solyc04g049090  GCTATATTTT CTGACTACTA CCGATCGTAC GTATTAAATT GGCCTTATTT 3700
cDNA_Solyc04g049090  GCTATATTTT CTGACTACTA CCGATCG--- ---------- ---------- 993
         SlMLO1_LEAF GCTATATTTT CTGACTACTA CCGATCG--- ---------- ---------- 797
         SlMLO1_ROOT GCTATATTTT CTGACTACTA CCGATCG--- ---------- ---------- 797
       SlMLO1_FLOWER GCTATATTTT CTGACTACTA CCGATCG--- ---------- ---------- 797
                                         3,720                          3,740
gDNA_Solyc04g049090  CACATTTATA TATTCTAATT TTGAGTGTAA ATAAGTAGAT ATATAGTTCT 3750
cDNA_Solyc04g049090  ---------- ---------- ---------- ---------- ---------- 993
         SlMLO1_LEAF ---------- ---------- ---------- ---------- ---------- 797
         SlMLO1_ROOT ---------- ---------- ---------- ---------- ---------- 797
       SlMLO1_FLOWER ---------- ---------- ---------- ---------- ---------- 797
                              3,760                          3,780               3,800
gDNA_Solyc04g049090  GCGTGTCATA ATACATGTAT GAAGCAAATT ATCACGTATG ACATGTATGT 3800
cDNA_Solyc04g049090  ---------- ---------- ---------- ---------- ---------- 993
         SlMLO1_LEAF ---------- ---------- ---------- ---------- ---------- 797
         SlMLO1_ROOT ---------- ---------- ---------- ---------- ---------- 797
       SlMLO1_FLOWER ---------- ---------- ---------- ---------- ---------- 797
                                         3,820                          3,840
gDNA_Solyc04g049090  TTATTTATTT TTACAAATTT GAATATCTAC TTATTTGCAC TCAAAATTGG 3850
cDNA_Solyc04g049090  ---------- ---------- ---------- ---------- ---------- 993
         SlMLO1_LEAF ---------- ---------- ---------- ---------- ---------- 797
         SlMLO1_ROOT ---------- ---------- ---------- ---------- ---------- 797
       SlMLO1_FLOWER ---------- ---------- ---------- ---------- ---------- 797
                              3,860                          3,880               3,900
gDNA_Solyc04g049090  AGGATATAAA TGTGAATCGG GACCACACAT ATTTATGTGT TATGGCATTG 3900
cDNA_Solyc04g049090  ---------- ---------- ---------- ---------- ---------- 993
         SlMLO1_LEAF ---------- ---------- ---------- ---------- ---------- 797
         SlMLO1_ROOT ---------- ---------- ---------- ---------- ---------- 797
       SlMLO1_FLOWER ---------- ---------- ---------- ---------- ---------- 797
                                         3,920                          3,940
gDNA_Solyc04g049090  AAAGGATTAT TAATTAGTTG AATAAAGCAT ATGTAATTGA AAGTTGTTAC 3950
cDNA_Solyc04g049090  ---------- ---------- ---------- ---------- ---------- 993
         SlMLO1_LEAF ---------- ---------- ---------- ---------- ---------- 797
         SlMLO1_ROOT ---------- ---------- ---------- ---------- ---------- 797
       SlMLO1_FLOWER ---------- ---------- ---------- ---------- ---------- 797
                              3,960                          3,980               4,000
gDNA_Solyc04g049090  TTTTAATATT GGACAGGATT GTACTCGTAT CTTTGGGTGC CATTTATCCC 4000
cDNA_Solyc04g049090  ---------- -------ATT GTACTCGTAT CTTTGGGTGC CATTTATCCC 1026
         SlMLO1_LEAF ---------- -------ATT GTACTCGTAT CTTTGGGTGC CATTTATCCC 830
         SlMLO1_ROOT ---------- -------ATT GTACTCGTAT CTTTGGGTGC CATTTATCCC 830
       SlMLO1_FLOWER ---------- -------ATT GTACTCGTAT CTTTGGGTGC CATTTATCCC 830
                                         4,020                          4,040
gDNA_Solyc04g049090  ACTTGTAGTA AGTTCATTAT TAATTCATTA ATAATAAGAG TAATAAGTAT 4050
cDNA_Solyc04g049090  ACTTGTA--- ---------- ---------- ---------- ---------- 1033
         SlMLO1_LEAF ACTTGTA--- ---------- ---------- ---------- ---------- 837
         SlMLO1_ROOT ACTTGTA--- ---------- ---------- ---------- ---------- 837
       SlMLO1_FLOWER ACTTGTA--- ---------- ---------- ---------- ---------- 837
                              4,060                          4,080               4,100
gDNA_Solyc04g049090  GTTCATGATT GACTAAAGTA TAAAATGAAA TTGAATTAAT GAAAATGCAG 4100
cDNA_Solyc04g049090  ---------- ---------- ---------- ---------- ---------- 1033
         SlMLO1_LEAF ---------- ---------- ---------- ---------- ---------- 837
         SlMLO1_ROOT ---------- ---------- ---------- ---------- ---------- 837
       SlMLO1_FLOWER ---------- ---------- ---------- ---------- ---------- 837
                                         4,120                          4,140
gDNA_Solyc04g049090  ATAATATTGC TAGTTGGCAC AAAACTTCAA ATGATCATAA CAGAAATGGG 4150
cDNA_Solyc04g049090  ATAATATTGC TAGTTGGCAC AAAACTTCAA ATGATCATAA CAGAAATGGG 1083
         SlMLO1_LEAF ATAATATTGC TAGTTGGCAC AAAACTTCAA ATGATCATAA CAGAAATGGG 887
         SlMLO1_ROOT ATAATATTGC TAGTTGGCAC AAAACTTCAA ATGATCATAA CAGAAATGGG 887
       SlMLO1_FLOWER ATAATATTGC TAGTTGGCAC AAAACTTCAA ATGATCATAA CAGAAATGGG 887
                              4,160                          4,180               4,200
gDNA_Solyc04g049090  AGTAAGGATT TCAGAAAGGG GAGACATAGT AAAAGGTGTA CCTGTGGTGG 4200
cDNA_Solyc04g049090  AGTAAGGATT TCAGAAAGGG GAGACATAGT AAAAGGTGTA CCTGTGGTGG 1133
         SlMLO1_LEAF AGTAAGGATT TCAGAAAGGG GAGACATAGT AAAAGGTGTA CCTGTGGTGG 937
         SlMLO1_ROOT AGTAAGGATT TCAGAAAGGG GAGACATAGT AAAAGGTGTA CCTGTGGTGG 937
       SlMLO1_FLOWER AGTAAGGATT TCAGAAAGGG GAGACATAGT AAAAGGTGTA CCTGTGGTGG 937
```

```
                                        4,220                 4,240
gDNA_Solyc04g049090  AGACTGGTGA CCATCTTTTC TGGTTTAATC GCCCTGCCCT TGTCCTATTC 4250
cDNA_Solyc04g049090  AGACTGGTGA CCATCTTTTC TGGTTTAATC GCCCTGCCCT TGTCCTATTC 1183
        SlMLO1_LEAF  AGACTGGTGA CCATCTTTTC TGGTTTAATC GCCCTGCCCT TGTCCTATTC 987
        SlMLO1_ROOT  AGACTGGTGA CCATCTTTTC TGGTTTAATC GCCCTGCCCT TGTCCTATTC 987
      SlMLO1_FLOWER  AGACTGGTGA CCATCTTTTC TGGTTTAATC GCCCTGCCCT TGTCCTATTC 987
                              4,260                 4,280                 4,300
gDNA_Solyc04g049090  TTGATTAACT TTGTACTCTT TCAGGTACCC TTTTTTAAAC TCTCACTTAC 4300
cDNA_Solyc04g049090  TTGATTAACT TTGTACTCTT TCAG------ ---------- ---------- 1207
        SlMLO1_LEAF  TTGATTAACT TTGTACTCTT TCAG------ ---------- ---------- 1011
        SlMLO1_ROOT  TTGATTAACT TTGTACTCTT TCAG------ ---------- ---------- 1011
      SlMLO1_FLOWER  TTGATTAACT TTGTACTCTT TCAG------ ---------- ---------- 1011
                                        4,320                 4,340
gDNA_Solyc04g049090  TTTTATTTTT AGTTGATCGA TTTATATATA AAAAGAATCA TCTATTATTT 4350
cDNA_Solyc04g049090  ---------- ---------- ---------- ---------- ---------- 1207
        SlMLO1_LEAF  ---------- ---------- ---------- ---------- ---------- 1011
        SlMLO1_ROOT  ---------- ---------- ---------- ---------- ---------- 1011
      SlMLO1_FLOWER  ---------- ---------- ---------- ---------- ---------- 1011
                              4,360                 4,380                 4,400
gDNA_Solyc04g049090  CTTTTTCATA TATTAATTTA TGATGATCAC AAAATTGAAA AGAAAAACTA 4400
cDNA_Solyc04g049090  ---------- ---------- ---------- ---------- ---------- 1207
        SlMLO1_LEAF  ---------- ---------- ---------- ---------- ---------- 1011
        SlMLO1_ROOT  ---------- ---------- ---------- ---------- ---------- 1011
      SlMLO1_FLOWER  ---------- ---------- ---------- ---------- ---------- 1011
                                        4,420                 4,440
gDNA_Solyc04g049090  TGTAATTTTT TTTTAAAAAA AAATAAACGA AAAGAGTATA TAAAATCGGT 4450
cDNA_Solyc04g049090  ---------- ---------- ---------- ---------- ---------- 1207
        SlMLO1_LEAF  ---------- ---------- ---------- ---------- ---------- 1011
        SlMLO1_ROOT  ---------- ---------- ---------- ---------- ---------- 1011
      SlMLO1_FLOWER  ---------- ---------- ---------- ---------- ---------- 1011
                              4,460                 4,480                 4,500
gDNA_Solyc04g049090  ACAAACTCCT AACTTGGAAT CTCATAATGA TTTTTTTTTT GGTTTGGCAG 4500
cDNA_Solyc04g049090  ---------- ---------- ---------- ---------- ---------- 1207
        SlMLO1_LEAF  ---------- ---------- ---------- ---------- ---------- 1011
        SlMLO1_ROOT  ---------- ---------- ---------- ---------- ---------- 1011
      SlMLO1_FLOWER  ---------- ---------- ---------- ---------- ---------- 1011
                                        4,520                 4,540
gDNA_Solyc04g049090  AATGCGTTTC AAGTTGCTTT CTTTTTTTGG AGTTGGGTAA GGAGTTGGAT 4550
cDNA_Solyc04g049090  AATGCGTTTC AAGTTGCTTT CTTTTTTTGG AGTTGG---- ---------- 1243
        SlMLO1_LEAF  AATGCGTTTC AAGTTGCTTT CTTTTTTTGG AGTTGG---- ---------- 1047
        SlMLO1_ROOT  AATGCGTTTC AAGTTGCTTT CTTTTTTTGG AGTTGG---- ---------- 1047
      SlMLO1_FLOWER  AATGCGTTTC AAGTTGCTTT CTTTTTTTGG AGTTGG---- ---------- 1047
                              4,560                 4,580                 4,600
gDNA_Solyc04g049090  TTCCATTTTT CCTTTTTTTC TTTTGGAATG AAATTTAAAT ATATTGTCCT 4600
cDNA_Solyc04g049090  ---------- ---------- ---------- ---------- ---------- 1243
        SlMLO1_LEAF  ---------- ---------- ---------- ---------- ---------- 1047
        SlMLO1_ROOT  ---------- ---------- ---------- ---------- ---------- 1047
      SlMLO1_FLOWER  ---------- ---------- ---------- ---------- ---------- 1047
                                        4,620                 4,640
gDNA_Solyc04g049090  GAATATTTTT TTTTCCTTCA AATTTTCAGT GGAAATTTGG TTTCCCATCT 4650
cDNA_Solyc04g049090  ---------- ---------- ---------T GGAAATTTGG TTTCCCATCT 1264
        SlMLO1_LEAF  ---------- ---------- ---------T GGAAATTTGG TTTCCCATCT 1068
        SlMLO1_ROOT  ---------- ---------- ---------T GGAAATTTGG TTTCCCATCT 1068
      SlMLO1_FLOWER  ---------- ---------- ---------T GGAAATTTGG TTTCCCATCT 1068
                              4,660                 4,680                 4,700
gDNA_Solyc04g049090  TGCTTTCATA AGAATGCTGC AGACCTAGCC ATAAGGCTAA CCATGGGGTG 4700
cDNA_Solyc04g049090  TGCTTTCATA AGAATGCTGC AGACCTAGCC ATAAGGCTAA CCATGGGG-- 1312
        SlMLO1_LEAF  TGCTTTCATA AGAATGCTGC AGACCTAGCC ATAAGGCTAA CCATGGGG-- 1116
        SlMLO1_ROOT  TGCTTTCATA AGAATGCTGC AGACCTAGCC ATAAGGCTAA CCATGGGG-- 1116
      SlMLO1_FLOWER  TGCTTTCATA AGAATGCTGC AGACCTAGCC ATAAGGCTAA CCATGGGG-- 1116
                                        4,720                 4,740
gDNA_Solyc04g049090  AGTTTCACTT GCAACTAAAA AACACAGACA CATTACACTT TTAAACTCTC 4750
cDNA_Solyc04g049090  ---------- ---------- ---------- ---------- ---------- 1312
        SlMLO1_LEAF  ---------- ---------- ---------- ---------- ---------- 1116
        SlMLO1_ROOT  ---------- ---------- ---------- ---------- ---------- 1116
      SlMLO1_FLOWER  ---------- ---------- ---------- ---------- ---------- 1116
                              4,760                 4,780                 4,800
gDNA_Solyc04g049090  ACCTGTTTCA GATAAAAAAT TTCAAAAGTC TTTCATTGTT TCTTAAAGTT 4800
cDNA_Solyc04g049090  ---------- ---------- ---------- ---------- ---------- 1312
        SlMLO1_LEAF  ---------- ---------- ---------- ---------- ---------- 1116
        SlMLO1_ROOT  ---------- ---------- ---------- ---------- ---------- 1116
      SlMLO1_FLOWER  ---------- ---------- ---------- ---------- ---------- 1116
                                        4,820                 4,840
gDNA_Solyc04g049090  TATGTCAAGT CAAAACATTA TGGAAGTTGA TGCATATATA TATTCAGGGT 4850
cDNA_Solyc04g049090  ---------- ---------- ---------- ---------- --------GT 1314
        SlMLO1_LEAF  ---------- ---------- ---------- ---------- --------GT 1118
        SlMLO1_ROOT  ---------- ---------- ---------- ---------- --------GT 1118
      SlMLO1_FLOWER  ---------- ---------- ---------- ---------- --------GT 1118
                              4,860                 4,880                 4,900
gDNA_Solyc04g049090  GATCATACAG GTCCATTGCA GCTATGTGAC TCTCCCTCTT TATGCCTTAG 4900
cDNA_Solyc04g049090  GATCATACAG GTCCATTGCA GCTATGTGAC TCTCCCTCTT TATGCCTTAG 1364
        SlMLO1_LEAF  GATCATACAG GTCCATTGCA GCTATGTGAC TCTCCCTCTT TATGCCTTAG 1168
        SlMLO1_ROOT  GATCATACAG GTCCATTGCA GCTATGTGAC TCTCCCTCTT TATGCCTTAG 1168
      SlMLO1_FLOWER  GATCATACAG GTCCATTGCA GCTATGTGAC TCTCCCTCTT TATGCCTTAG 1168
```

```
                                     4,920                          4,940
gDNA_Solyc04g049090  TTACACAGGT AGGTACTCTG ATCTACATTA TGAAAATGTT ATATATATAT 4950
cDNA_Solyc04g049090  TTACACAG-- ---------- ---------- ---------- ---------- 1372
        SlMLO1_LEAF  TTACACAG-- ---------- ---------- ---------- ---------- 1176
        SlMLO1_ROOT  TTACACAG-- ---------- ---------- ---------- ---------- 1176
      SlMLO1_FLOWER  TTACACAG-- ---------- ---------- ---------- ---------- 1176
                          4,960                          4,980            5,000
gDNA_Solyc04g049090  ATATATATAT ATATATATAT ATATATATAC TTATATAGAG AAATTCTGTA 5000
cDNA_Solyc04g049090  ---------- ---------- ---------- ---------- ---------- 1372
        SlMLO1_LEAF  ---------- ---------- ---------- ---------- ---------- 1176
        SlMLO1_ROOT  ---------- ---------- ---------- ---------- ---------- 1176
      SlMLO1_FLOWER  ---------- ---------- ---------- ---------- ---------- 1176
                                     5,020                          5,040
gDNA_Solyc04g049090  GATGGGTTCA TCAATGAAGC CTATCATCTT TGGTGATAAT GTGGCAACAG 5050
cDNA_Solyc04g049090  -ATGGGTTCA TCAATGAAGC CTATCATCTT TGGTGATAAT GTGGCAACAG 1421
        SlMLO1_LEAF  -ATGGGTTCA TCAATGAAGC CTATCATCTT TGGTGATAAT GTGGCAACAG 1225
        SlMLO1_ROOT  -ATGGGTTCA TCAATGAAGC CTATCATCTT TGGTGATAAT GTGGCAACAG 1225
      SlMLO1_FLOWER  ---------- ---------- ---------- ---------- ---------- 1176
                          5,060                          5,080            5,100
gDNA_Solyc04g049090  CTCTTAGAAG CTGGCACCAT ACAGCGAAAA AACGGGTGAA ACATGGGCTA 5100
cDNA_Solyc04g049090  CTCTTAGAAG CTGGCACCAT ACAGCGAAAA AACGGGTGAA ACATGGGCTA 1471
        SlMLO1_LEAF  CTCTTAGAAG CTGGCACCAT ACAGCGAAAA AACGGGTGAA ACATGGGCTA 1275
        SlMLO1_ROOT  CTCTTAGAAG CTGGCACCAT ACAGCGAAAA AACGGGTGAA ACATGGGCTA 1275
      SlMLO1_FLOWER  CTCTTAGAAG CTGGCACCAT ACAGCGAAAA AACGGGTGAA ACATGGGCTA 1226
                                     5,120                          5,140
gDNA_Solyc04g049090  TCAGGACATA CCACCCCTGC AAACAGCAGA CCAACCACAC CATTGCGTGG 5150
cDNA_Solyc04g049090  TCAGGACATA CCACCCCTGC AAACAGCAGA CCAACCACAC CATTGCGTGG 1521
        SlMLO1_LEAF  TCAGGACATA CCACCCCTGC AAACAGCAGA CCAACCACAC CATTGCGTGG 1325
        SlMLO1_ROOT  TCAGGACATA CCACCCCTGC AAACAGCAGA CCAACCGCAC CATTGCGTGG 1325
      SlMLO1_FLOWER  TCAGGACATA CCACCCCTGC AAACAGCAGA CCAACCACAC CATTGCGTGG 1276
                          5,160                          5,180            5,200
gDNA_Solyc04g049090  TACCTCCCCT GTTCACTTAT TACGCGGTTA TCCACAATAT AATGAGGACA 5200
cDNA_Solyc04g049090  TACCTCCCCT GTTCACTTAT TACGCGGTTA TCCACAATAT AATGAGGACA 1571
        SlMLO1_LEAF  TACCTCCCCT GTTCACTTAT TACGCGGTTA TCCACAATAT AATGAGGACA 1375
        SlMLO1_ROOT  TACCTCCCCT GTTCACTTCT TACGCGGTTA TCCACAATAT AATGAGGACA 1375
      SlMLO1_FLOWER  TACCTCCCCT GTTCACTTAT TACGCGGTTA TCCACAATAT AATGAGGACA 1326
                                     5,220                          5,240
gDNA_Solyc04g049090  GTGTTCAAGC ATCTCCTCGG ACATCCAATG TCGAAAATGA AGGGTGGGCT 5250
cDNA_Solyc04g049090  GTGTTCAAGC ATCTCCTCGG ACATCCAATG TCGAAAATGA AGGGTGGGCT 1621
        SlMLO1_LEAF  GTGTTCAAGC ATCTCCTCGG ACATCCAATG TCGAAAATGA AGGGTGGGCT 1425
        SlMLO1_ROOT  GTGTTCAAGC ATCTCCTCGG ACATCCAATG TCGAAAATGA AGGGTGGGCT 1425
      SlMLO1_FLOWER  GTGTTCAAGC ATCTCCTCGG ACATCCAATG TCGAAAATGA AGGGTGGGCT 1376
                          5,260                          5,280            5,300
gDNA_Solyc04g049090  AATGAAAATC AGGAGGGAGA GATCCTGCAG CATGCCTCCA CTGATCATAA 5300
cDNA_Solyc04g049090  AATGAAAATC AGGAGGGAGA GATCCTGCAG CATGCCTCCA CTGATCATAA 1671
        SlMLO1_LEAF  AATGAAAATC AGGAGGGAGA GATCCTGCAG CATGCCTCCA CTGATCATAA 1475
        SlMLO1_ROOT  AATGAAAATC AGGAGGGAGA GATCCTGCAG CATGCCTCCA CTGATCATAA 1475
      SlMLO1_FLOWER  AATGAAAATC AGGAGGGAGA GATCCTGCAG CATGCCTCCA CTGATCATAA 1426
                                     5,320                          5,340
gDNA_Solyc04g049090  CAAGCAAATT GAGATTACAA TGTCAGATTT TACTTTTGGA AACAAATAAA 5350
cDNA_Solyc04g049090  CAAGCAAATT GAGATTACAA TGTCAGATTT TACTTTTGGA AACAAATAAA 1721
        SlMLO1_LEAF  CAAGCAAATT GAGATTACAA TGTCAGATTT TACTTTTGGA AACAAATAA- 1524
        SlMLO1_ROOT  CAAGCAAATT GAGATTACAA TGTCAGATTT TACTTTTGGA AACAAATAA- 1524
      SlMLO1_FLOWER  CAAGCAAATT GAGATTACAA TGTCAGATTT TACTTTTGGA AACAAATAA- 1475
                          5,360                          5,380            5,400
gDNA_Solyc04g049090  TGTAAAAACG AATTTTCTTC TTCATTGTTT TAAGTTCATT ACTGTAGTTC 5400
cDNA_Solyc04g049090  TGTAAAAACG AATTTTCTTC TTCATTGTTT TAAGTTCATT ACTGTAGTTC 1771
        SlMLO1_LEAF  ---------- ---------- ---------- ---------- ---------- 1524
        SlMLO1_ROOT  ---------- ---------- ---------- ---------- ---------- 1524
      SlMLO1_FLOWER  ---------- ---------- ---------- ---------- ---------- 1475
                                     5,420                          5,440
gDNA_Solyc04g049090  AAATGGCAAT GATTTTGTAA AATTTTATAC AGAGGTACTC ATGCATGGTG 5450
cDNA_Solyc04g049090  AAATGGCAAT GATTTTGTAA AATTTTATAC AGAGGTACTC ATGCATGGTG 1821
        SlMLO1_LEAF  ---------- ---------- ---------- ---------- ---------- 1524
        SlMLO1_ROOT  ---------- ---------- ---------- ---------- ---------- 1524
      SlMLO1_FLOWER  ---------- ---------- ---------- ---------- ---------- 1475
                          5,460                          5,480            5,500
gDNA_Solyc04g049090  CTCTTCATTT CAAGGTAAGA ACCTTCTTAT ATCGATTTAT AGCTACTTTA 5500
cDNA_Solyc04g049090  CTCTTCATTT CAAGGTAAGA ACCTTCTTAT ATCGATTTAT AGCTACTTTA 1871
        SlMLO1_LEAF  ---------- ---------- ---------- ---------- ---------- 1524
        SlMLO1_ROOT  ---------- ---------- ---------- ---------- ---------- 1524
      SlMLO1_FLOWER  ---------- ---------- ---------- ---------- ---------- 1475

gDNA_Solyc04g049090  CATCTCA 5507
cDNA_Solyc04g049090  CATCTCA 1878
        SlMLO1_LEAF  ------- 1524
        SlMLO1_ROOT  ------- 1524
      SlMLO1_FLOWER  ------- 1475
```

```
                                        20                    40
gDNA_Solyc03g095650  TATATTTAAA ATATAAAGTC TTTGTTTTTT CTCAAAGAAG TCTCTCACAA  50
cDNA_Solyc03g095650  TATATTTAAA ATATAAAGTC TTTGTTTTTT CTCAAAGAAG TCTCTCACAA  50
SlMLO5_LEAF          ---------- ---------- ---------- ---------- ----------  -
SlMLO5_FLOWER        ---------- ---------- ---------- ---------- ----------  -
SlMLO5_FRUIT         ---------- ---------- ---------- ---------- ----------  -

                              60                    80                   100
gDNA_Solyc03g095650  ATTTCCCTTA TCATTAAAAA CAAAAAAAAA GTTGTATAGT ACTGAAGCTT  100
cDNA_Solyc03g095650  ATTTCCCTTA TCATTAAAAA CAAAAAAAAA GTTGTATAGT ACTGAAGCTT  100
SlMLO5_LEAF          ---------- ---------- ---------- ---------- ----------  -
SlMLO5_FLOWER        ---------- ---------- ---------- ---------- ----------  -
SlMLO5_FRUIT         ---------- ---------- ---------- ---------- ----------  -

                                       120                   140
gDNA_Solyc03g095650  AATGGCTAGC ACAGGCTGTA TTAGAACGTG TGATGAACGT CCTCTAGATG  150
cDNA_Solyc03g095650  AATGGCTAGC ACAGGCTGTA TTAGAACGTG TGATGAACGT CCTCTAGATG  150
SlMLO5_LEAF          -ATGGCTAGC ACAGGCTGTA TTAGAACGTG TGATGAACGT CCTCTAGATG  49
SlMLO5_FLOWER        -ATGGCTAGC ACAGGCTGTA TTAGAACGTG TGATGAACGT CCTCTAGATG  49
SlMLO5_FRUIT         -ATGGCTAGC ACAGGCTGTA TTAGAACGTG TGATGAACGT CCTCTAGATG  49

                             160                   180                   200
gDNA_Solyc03g095650  AGACACCAAC ATGGGCTGTA GCCATGGTTT GCTTTGTATT AGTTGTAATC  200
cDNA_Solyc03g095650  AGACACCAAC ATGGGCTGTA GCCATGGTTT GCTTTGTATT AGTTGTAATC  200
SlMLO5_LEAF          AGACACCAAC ATGGGCTGTA GCCATGGTTT GCTTTGTATT AGTTGTAATC  99
SlMLO5_FLOWER        AGACACCAAC ATGGGCTGTA GCCATGGTTT GCTTTGTATT AGTTGTAATC  99
SlMLO5_FRUIT         AGACACCAAC ATGGGCTGTA GCCATGGTTT GCTTTGTATT AGTTGTAATC  99

                                       220                   240
gDNA_Solyc03g095650  TCCCTTTTCA TTGAACAACT TATTCATCAT CTTGGAGAGG TAAACATTCA  250
cDNA_Solyc03g095650  TCCCTTTTCA TTGAACAACT TATTCATCAT CTTGGAGAG- ----------  239
SlMLO5_LEAF          TCCCTTTTCA TTGAACAACT TATTCATCAT CTTGGAGAG- ----------  138
SlMLO5_FLOWER        TCCCTTTTCA TTGAACAACT TATTCATCAT CTTGGAGAG- ----------  138
SlMLO5_FRUIT         TCCCTTTTCA TTGAACAACT TATTCATCAT CTTGGAGAG- ----------  138

                             260                   280                   300
gDNA_Solyc03g095650  TTTACCTTTC ATATTTTTAT ATATGTTTGA ATCACAAAAT TATATGTTGC  300
cDNA_Solyc03g095650  ---------- ---------- ---------- ---------- ----------  239
SlMLO5_LEAF          ---------- ---------- ---------- ---------- ----------  138
SlMLO5_FLOWER        ---------- ---------- ---------- ---------- ----------  138
SlMLO5_FRUIT         ---------- ---------- ---------- ---------- ----------  138

                                       320                   340
gDNA_Solyc03g095650  CTTTTGATGA TTTGTGTTTA TACAAGTCCA TGCTGCTATC AGTGAGGAAA  350
cDNA_Solyc03g095650  ---------- ---------- ---------- ---------- ----------  239
SlMLO5_LEAF          ---------- ---------- ---------- ---------- ----------  138
SlMLO5_FLOWER        ---------- ---------- ---------- ---------- ----------  138
SlMLO5_FRUIT         ---------- ---------- ---------- ---------- ----------  138

                             360                   380                   400
gDNA_Solyc03g095650  ACACAAATTT TTAGGTGGAG GGGGACCAAA ATGTTTTAAG AGGAAAAGTA  400
cDNA_Solyc03g095650  ---------- ---------- ---------- ---------- ----------  239
SlMLO5_LEAF          ---------- ---------- ---------- ---------- ----------  138
SlMLO5_FLOWER        ---------- ---------- ---------- ---------- ----------  138
SlMLO5_FRUIT         ---------- ---------- ---------- ---------- ----------  138

                                       420                   440
gDNA_Solyc03g095650  GCAAGGAGCA AAACTTAAAA ACTTCTATAT GCAATTTTGT TTTAGGTAAA  450
cDNA_Solyc03g095650  ---------- ---------- ---------- ---------- ----------  239
SlMLO5_LEAF          ---------- ---------- ---------- ---------- ----------  138
SlMLO5_FLOWER        ---------- ---------- ---------- ---------- ----------  138
SlMLO5_FRUIT         ---------- ---------- ---------- ---------- ----------  138

                             460                   480                   500
gDNA_Solyc03g095650  TATAGTATAA TATCATATTA ACGTACAGAG TTGTTAATAT GGACCAACTT  500
cDNA_Solyc03g095650  ---------- ---------- ---------- ---------- ----------  239
SlMLO5_LEAF          ---------- ---------- ---------- ---------- ----------  138
SlMLO5_FLOWER        ---------- ---------- ---------- ---------- ----------  138
SlMLO5_FRUIT         ---------- ---------- ---------- ---------- ----------  138

                                       520                   540
gDNA_Solyc03g095650  TCCTCAGCTT ATGCCGTGGC TTTGAAATTT GACGAGCTAA TTGGACCAGC  550
cDNA_Solyc03g095650  ---------- ---------- ---------- ---------- ----------  239
SlMLO5_LEAF          ---------- ---------- ---------- ---------- ----------  138
SlMLO5_FLOWER        ---------- ---------- ---------- ---------- ----------  138
SlMLO5_FRUIT         ---------- ---------- ---------- ---------- ----------  138

                             560                   580                   600
gDNA_Solyc03g095650  TCACTATTTT AATAGGCCTT AAAATAGTCA ACCCCAGTAT TATGTGGGCA  600
cDNA_Solyc03g095650  ---------- ---------- ---------- ---------- ----------  239
SlMLO5_LEAF          ---------- ---------- ---------- ---------- ----------  138
SlMLO5_FLOWER        ---------- ---------- ---------- ---------- ----------  138
SlMLO5_FRUIT         ---------- ---------- ---------- ---------- ----------  138

                                       620                   640
gDNA_Solyc03g095650  GTGGATTAGG GCTATTGGTT CATCATTTTT TCAAAACGTA TTCTTTTTAA  650
cDNA_Solyc03g095650  ---------- ---------- ---------- ---------- ----------  239
SlMLO5_LEAF          ---------- ---------- ---------- ---------- ----------  138
SlMLO5_FLOWER        ---------- ---------- ---------- ---------- ----------  138
SlMLO5_FRUIT         ---------- ---------- ---------- ---------- ----------  138

                             660                   680                   700
gDNA_Solyc03g095650  CTCTTTTCGT TTCACAAAGA ATGACCTGAT TTGACTTGAT GCGGAGTTAA  700
cDNA_Solyc03g095650  ---------- ---------- ---------- ---------- ----------  239
SlMLO5_LEAF          ---------- ---------- ---------- ---------- ----------  138
SlMLO5_FLOWER        ---------- ---------- ---------- ---------- ----------  138
SlMLO5_FRUIT         ---------- ---------- ---------- ---------- ----------  138
```

```
                                                 720                           740
gDNA_Solyc03g095650  AGAGTATAAA TAAGATTTTT GTATTTTGTA ATTCTAAATT AAAGTTGGTT  750
cDNA_Solyc03g095650  ---------- ---------- ---------- ---------- ----------  239
       SlMLO5_LEAF   ---------- ---------- ---------- ---------- ----------  138
     SlMLO5_FLOWER   ---------- ---------- ---------- ---------- ----------  138
      SlMLO5_FRUIT   ---------- ---------- ---------- ---------- ----------  138
                                 760                           780                    800
gDNA_Solyc03g095650  AAATGTAAAA AATTGTTTTT TGATCTTGTG GTCTTAAACA TGTCAGGCAG  800
cDNA_Solyc03g095650  ---------- ---------- ---------- ---------- ----------  239
       SlMLO5_LEAF   ---------- ---------- ---------- ---------- ----------  138
     SlMLO5_FLOWER   ---------- ---------- ---------- ---------- ----------  138
      SlMLO5_FRUIT   ---------- ---------- ---------- ---------- ----------  138
                                                 820                           840
gDNA_Solyc03g095650  AAAGTTGGAA TTAAAATATT ACCAAAAAAG AAAAAAGTCA TTATTTTTTA  850
cDNA_Solyc03g095650  ---------- ---------- ---------- ---------- ----------  239
       SlMLO5_LEAF   ---------- ---------- ---------- ---------- ----------  138
     SlMLO5_FLOWER   ---------- ---------- ---------- ---------- ----------  138
      SlMLO5_FRUIT   ---------- ---------- ---------- ---------- ----------  138
                                 860                           880                    900
gDNA_Solyc03g095650  TACGAATCAA AAAGAAAAGG AGATCATTCT TTTTTAAATA GAGAGAATAT  900
cDNA_Solyc03g095650  ---------- ---------- ---------- ---------- ----------  239
       SlMLO5_LEAF   ---------- ---------- ---------- ---------- ----------  138
     SlMLO5_FLOWER   ---------- ---------- ---------- ---------- ----------  138
      SlMLO5_FRUIT   ---------- ---------- ---------- ---------- ----------  138
                                                 920                           940
gDNA_Solyc03g095650  AATTATTTTA ATCCAAACTT AACATAAAAT GCAAAAATAT AAATAATGTA  950
cDNA_Solyc03g095650  ---------- ---------- ---------- ---------- ----------  239
       SlMLO5_LEAF   ---------- ---------- ---------- ---------- ----------  138
     SlMLO5_FLOWER   ---------- ---------- ---------- ---------- ----------  138
      SlMLO5_FRUIT   ---------- ---------- ---------- ---------- ----------  138
                                 960                           980                  1,000
gDNA_Solyc03g095650  AGACAATATT ACATAACGTA AAATAAAATA ATTGAAAAGA ATACCTTATA  1000
cDNA_Solyc03g095650  ---------- ---------- ---------- ---------- ----------  239
       SlMLO5_LEAF   ---------- ---------- ---------- ---------- ----------  138
     SlMLO5_FLOWER   ---------- ---------- ---------- ---------- ----------  138
      SlMLO5_FRUIT   ---------- ---------- ---------- ---------- ----------  138
                                               1,020                         1,040
gDNA_Solyc03g095650  TTTGTCAATA CTTTATGTCC TTGTCATTGA CGTCAAGAAT TTGTTGTCAC  1050
cDNA_Solyc03g095650  ---------- ---------- ---------- ---------- ----------  239
       SlMLO5_LEAF   ---------- ---------- ---------- ---------- ----------  138
     SlMLO5_FLOWER   ---------- ---------- ---------- ---------- ----------  138
      SlMLO5_FRUIT   ---------- ---------- ---------- ---------- ----------  138
                               1,060                         1,080                  1,100
gDNA_Solyc03g095650  GTAGTCACAA GTAATAAAGT GGTTAGATTA TAAGGGTCGT TTGGTTTGAT  1100
cDNA_Solyc03g095650  ---------- ---------- ---------- ---------- ----------  239
       SlMLO5_LEAF   ---------- ---------- ---------- ---------- ----------  138
     SlMLO5_FLOWER   ---------- ---------- ---------- ---------- ----------  138
      SlMLO5_FRUIT   ---------- ---------- ---------- ---------- ----------  138
                                               1,120                         1,140
gDNA_Solyc03g095650  TAAGAGCAGA CTCGAAAGGG AAGAGCGCGG TCTTGGGTTT AGTCTAAAAA  1150
cDNA_Solyc03g095650  ---------- ---------- ---------- ---------- ----------  239
       SlMLO5_LEAF   ---------- ---------- ---------- ---------- ----------  138
     SlMLO5_FLOWER   ---------- ---------- ---------- ---------- ----------  138
      SlMLO5_FRUIT   ---------- ---------- ---------- ---------- ----------  138
                               1,160                         1,180                  1,200
gDNA_Solyc03g095650  TTCAATACAT CGATAAGGGG CAGGGAATCA ATAAAACTTT GTTTGGTGTT  1200
cDNA_Solyc03g095650  ---------- ---------- ---------- ---------- ----------  239
       SlMLO5_LEAF   ---------- ---------- ---------- ---------- ----------  138
     SlMLO5_FLOWER   ---------- ---------- ---------- ---------- ----------  138
      SlMLO5_FRUIT   ---------- ---------- ---------- ---------- ----------  138
                                               1,220                         1,240
gDNA_Solyc03g095650  TTTTTTAAAT TTATGTATAA CTAATGTATG CATTAGCATG TTTAAAGAGG  1250
cDNA_Solyc03g095650  ---------- ---------- ---------- ---------- ----------  239
       SlMLO5_LEAF   ---------- ---------- ---------- ---------- ----------  138
     SlMLO5_FLOWER   ---------- ---------- ---------- ---------- ----------  138
      SlMLO5_FRUIT   ---------- ---------- ---------- ---------- ----------  138
                               1,260                         1,280                  1,300
gDNA_Solyc03g095650  TAATTGCCCT TAAAACCTTT TTTTATATCT TTTGGAGGAT ATCTTTGTAA  1300
cDNA_Solyc03g095650  ---------- ---------- ---------- ---------- ----------  239
       SlMLO5_LEAF   ---------- ---------- ---------- ---------- ----------  138
     SlMLO5_FLOWER   ---------- ---------- ---------- ---------- ----------  138
      SlMLO5_FRUIT   ---------- ---------- ---------- ---------- ----------  138
                                               1,320                         1,340
gDNA_Solyc03g095650  ATAATTTTTT TTATGCAATG CATGATATTT TTAATGCATC AAATCGGACA  1350
cDNA_Solyc03g095650  ---------- ---------- ---------- ---------- ----------  239
       SlMLO5_LEAF   ---------- ---------- ---------- ---------- ----------  138
     SlMLO5_FLOWER   ---------- ---------- ---------- ---------- ----------  138
      SlMLO5_FRUIT   ---------- ---------- ---------- ---------- ----------  138
                               1,360                         1,380                  1,400
gDNA_Solyc03g095650  ATATATAAAA ATAATCTATG TAAAATCAAT ACAAGCATAA CTAATACATG  1400
cDNA_Solyc03g095650  ---------- ---------- ---------- ---------- ----------  239
       SlMLO5_LEAF   ---------- ---------- ---------- ---------- ----------  138
     SlMLO5_FLOWER   ---------- ---------- ---------- ---------- ----------  138
      SlMLO5_FRUIT   ---------- ---------- ---------- ---------- ----------  138
```

```
                                       1,420                         1,440
gDNA_Solyc03g095650  CATTATTAAG GCAAACATTA TTAATACACT CTATTTAGCA TTATTAATCT 1450
cDNA_Solyc03g095650  ---------- ---------- ---------- ---------- ---------- 239
         SlMLO5_LEAF ---------- ---------- ---------- ---------- ---------- 138
       SlMLO5_FLOWER ---------- ---------- ---------- ---------- ---------- 138
        SlMLO5_FRUIT ---------- ---------- ---------- ---------- ---------- 138
                          1,460                         1,480                1,500
gDNA_Solyc03g095650  TGTATACTCT ATTAAGCGAA CCCTTAAAAT TGTTACGAGA GATATGATAT 1500
cDNA_Solyc03g095650  ---------- ---------- ---------- ---------- ---------- 239
         SlMLO5_LEAF ---------- ---------- ---------- ---------- ---------- 138
       SlMLO5_FLOWER ---------- ---------- ---------- ---------- ---------- 138
        SlMLO5_FRUIT ---------- ---------- ---------- ---------- ---------- 138
                                       1,520                         1,540
gDNA_Solyc03g095650  TAAGACCTAA GGTTATGGCG CTTGCATATT CAATAAAAGT GTTATCCCCT 1550
cDNA_Solyc03g095650  ---------- ---------- ---------- ---------- ---------- 239
         SlMLO5_LEAF ---------- ---------- ---------- ---------- ---------- 138
       SlMLO5_FLOWER ---------- ---------- ---------- ---------- ---------- 138
        SlMLO5_FRUIT ---------- ---------- ---------- ---------- ---------- 138
                          1,560                         1,580                1,600
gDNA_Solyc03g095650  CTCTCTCTCT ACAAATATCA TATGGTTGGC ATTTTTATAT TTCTTAATGT 1600
cDNA_Solyc03g095650  ---------- ---------- ---------- ---------- ---------- 239
         SlMLO5_LEAF ---------- ---------- ---------- ---------- ---------- 138
       SlMLO5_FLOWER ---------- ---------- ---------- ---------- ---------- 138
        SlMLO5_FRUIT ---------- ---------- ---------- ---------- ---------- 138
                                       1,620                         1,640
gDNA_Solyc03g095650  TTTTATAGTG GTTATGGAAG AAACAAAAGA GACCATTGTA TGAAGCACTT 1650
cDNA_Solyc03g095650  --------TG GTTATGGAAG AAACAAAAGA GACCATTGTA TGAAGCACTT 281
         SlMLO5_LEAF --------TG GTTATGGAAG AAACAAAAGA GACCATTGTA TGAAGCACTT 180
       SlMLO5_FLOWER --------TG GTTATGGAAG AAACAAAAGA GACCATTGTA TGAAGCACTT 180
        SlMLO5_FRUIT --------TG GTTATGGAAG AAACAAAAGA GACCATTGTA TGAAGCACTT 180
                          1,660                         1,680                1,700
gDNA_Solyc03g095650  GAGAAGATCA AGTCAGGTAA AAGAAATTTT GTTTTTTCAT TACAAAATCA 1700
cDNA_Solyc03g095650  GAGAAGATCA AGTCAG---- ---------- ---------- ---------- 297
         SlMLO5_LEAF GAGAAGATCA AGTCAG---- ---------- ---------- ---------- 196
       SlMLO5_FLOWER GAGAAGATCA AGTCAG---- ---------- ---------- ---------- 196
        SlMLO5_FRUIT GAGAAGATCA AGTCAG---- ---------- ---------- ---------- 196
                                       1,720                         1,740
gDNA_Solyc03g095650  TTGATTTTTG GACTAATAAT TTTATTTTGT AAATTTATCG TGGTGCAGAA 1750
cDNA_Solyc03g095650  ---------- ---------- ---------- ---------- --------AA 299
         SlMLO5_LEAF ---------- ---------- ---------- ---------- --------AA 198
       SlMLO5_FLOWER ---------- ---------- ---------- ---------- --------AA 198
        SlMLO5_FRUIT ---------- ---------- ---------- ---------- --------AA 198
                          1,760                         1,780                1,800
gDNA_Solyc03g095650  CTCATGTTAT TAGGGTTTAT ATCCTTATTC TTGACGGTTG TACAGGATCC 1800
cDNA_Solyc03g095650  CTCATGTTAT TAGGGTTTAT ATCCTTATTC TTGACGGTTG TACAGGATCC 349
         SlMLO5_LEAF CTCATGTTAT TAGGGTTTAT ATCCTTATTC TTGACGGTTG TACAGGATCC 248
       SlMLO5_FLOWER CTCATGTTAT TAGGGTTTAT ATCCTTATTC TTGACGGTTG TACAGGATCC 248
        SlMLO5_FRUIT CTCATGTTAT TAGGGTTTAT ATCCTTATTC TTGACGGTTG TACAGGATCC 248
                                       1,820                         1,840
gDNA_Solyc03g095650  TATGTCTAAG ATATGTATTC CTAGGAGTGT TGGACGCTCT TGGCATCCAT 1850
cDNA_Solyc03g095650  TATGTCTAAG ATATGTATTC CTAGGAGTGT TGGACGCTCT TGGCATCCAT 399
         SlMLO5_LEAF TATGTCTAAG ATATGTATTC CTAGGAGTGT TGGACGCTCT TGGCATCCAT 298
       SlMLO5_FLOWER TATGTCTAAG ATATGTATTC CTAGGAGTGT TGGACGCTCT TGGCATCCAT 298
        SlMLO5_FRUIT TATGTCTAAG ATATGTATTC CTAGGAGTGT TGGACGCTCT TGGCATCCAT 298
                          1,860                         1,880                1,900
gDNA_Solyc03g095650  GTGACATAAA CAAACATATT GATGACCAAT ATCTCGATCC ATGTAGAATT 1900
cDNA_Solyc03g095650  GTGACATAAA CAAACATATT GATGACCAAT ATCTCGATCC ATGTAGAATT 449
         SlMLO5_LEAF GTGACATAAA CAAACATATT GATGACCAAT ATCTCGATCC ATGTAGAATT 348
       SlMLO5_FLOWER GTGACATAAA CAAACATATT GATGACCAAT ATCTCGATCC ATGTAGAATT 348
        SlMLO5_FRUIT GTGACATAAA CAAACATATT GATGACCAAT ATCTCGATCC ATGTAGAATT 348
                                       1,920                         1,940
gDNA_Solyc03g095650  AAGGTATTTT TTTAAATCGA AACTCAAAGG TTACCCCTTT CTAAGAGCTT 1950
cDNA_Solyc03g095650  AAGG------ ---------- ---------- ---------- ---------- 453
         SlMLO5_LEAF AAGG------ ---------- ---------- ---------- ---------- 352
       SlMLO5_FLOWER AAGG------ ---------- ---------- ---------- ---------- 352
        SlMLO5_FRUIT AAGG------ ---------- ---------- ---------- ---------- 352
                          1,960                         1,980                2,000
gDNA_Solyc03g095650  ACACCCTTTG TGTTCTCTTG ATCAAAGACA TCTCTCTGTC AATGGATATA 2000
cDNA_Solyc03g095650  ---------- ---------- ---------- ---------- ---------- 453
         SlMLO5_LEAF ---------- ---------- ---------- ---------- ---------- 352
       SlMLO5_FLOWER ---------- ---------- ---------- ---------- ---------- 352
        SlMLO5_FRUIT ---------- ---------- ---------- ---------- ---------- 352
                                       2,020                         2,040
gDNA_Solyc03g095650  ACCCTTTTGG TATGAATAGT TTATCGGTTT GGGCATGGAT GTTCTTATTT 2050
cDNA_Solyc03g095650  ---------- ---------- ---------- ---------- ---------- 453
         SlMLO5_LEAF ---------- ---------- ---------- ---------- ---------- 352
       SlMLO5_FLOWER ---------- ---------- ---------- ---------- ---------- 352
        SlMLO5_FRUIT ---------- ---------- ---------- ---------- ---------- 352
                          2,060                         2,080                2,100
gDNA_Solyc03g095650  GGACATCTTG TTTGGGCTAC TGAATTTATG TTCTTAATTT CTTGGCGTGG 2100
cDNA_Solyc03g095650  ---------- ---------- ---------- ---------- ---------- 453
         SlMLO5_LEAF ---------- ---------- ---------- ---------- ---------- 352
       SlMLO5_FLOWER ---------- ---------- ---------- ---------- ---------- 352
        SlMLO5_FRUIT ---------- ---------- ---------- ---------- ---------- 352
```

```
                                              2,120                 2,140
gDNA_Solyc03g095650  ATATTGGCAG GAATTGTTTA TTGAGAATAC TAACCCTCTA GACAACCCAA 2150
cDNA_Solyc03g095650  ---------- ---------- ---------- ---------- ---------- 453
        SlMLO5_LEAF  ---------- ---------- ---------- ---------- ---------- 352
      SlMLO5_FLOWER  ---------- ---------- ---------- ---------- ---------- 352
       SlMLO5_FRUIT  ---------- ---------- ---------- ---------- ---------- 352
                                   2,160                 2,180                 2,200
gDNA_Solyc03g095650  CCTCTAAATT TTACTTACTA TAAAAGTAAA GACACAAATC TAAACTCTAG 2200
cDNA_Solyc03g095650  ---------- ---------- ---------- ---------- ---------- 453
        SlMLO5_LEAF  ---------- ---------- ---------- ---------- ---------- 352
      SlMLO5_FLOWER  ---------- ---------- ---------- ---------- ---------- 352
       SlMLO5_FRUIT  ---------- ---------- ---------- ---------- ---------- 352
                                              2,220                 2,240
gDNA_Solyc03g095650  CCACTATAAC ATCAAAACCA CTCCACTTTA CTAGAATGTC AAAACATACC 2250
cDNA_Solyc03g095650  ---------- ---------- ---------- ---------- ---------- 453
        SlMLO5_LEAF  ---------- ---------- ---------- ---------- ---------- 352
      SlMLO5_FLOWER  ---------- ---------- ---------- ---------- ---------- 352
       SlMLO5_FRUIT  ---------- ---------- ---------- ---------- ---------- 352
                                   2,260                 2,280                 2,300
gDNA_Solyc03g095650  ATTAAGATTG ACTTTATTAT TGTTATAATT GGTGAAGTTT TATAATTTCA 2300
cDNA_Solyc03g095650  ---------- ---------- ---------- ---------- ---------- 453
        SlMLO5_LEAF  ---------- ---------- ---------- ---------- ---------- 352
      SlMLO5_FLOWER  ---------- ---------- ---------- ---------- ---------- 352
       SlMLO5_FRUIT  ---------- ---------- ---------- ---------- ---------- 352
                                              2,320                 2,340
gDNA_Solyc03g095650  CTTTTATAAT GGATAGTTCA ATTGCTAACA AATATTGATT TTCTTTTTTA 2350
cDNA_Solyc03g095650  ---------- ---------- ---------- ---------- ---------- 453
        SlMLO5_LEAF  ---------- ---------- ---------- ---------- ---------- 352
      SlMLO5_FLOWER  ---------- ---------- ---------- ---------- ---------- 352
       SlMLO5_FRUIT  ---------- ---------- ---------- ---------- ---------- 352
                                   2,360                 2,380                 2,400
gDNA_Solyc03g095650  CTATTCTAAG GACAAAGTGT AGTTATTTTA ATAGCAAAAT AATTTAATAG 2400
cDNA_Solyc03g095650  ---------- ---------- ---------- ---------- ---------- 453
        SlMLO5_LEAF  ---------- ---------- ---------- ---------- ---------- 352
      SlMLO5_FLOWER  ---------- ---------- ---------- ---------- ---------- 352
       SlMLO5_FRUIT  ---------- ---------- ---------- ---------- ---------- 352
                                              2,420                 2,440
gDNA_Solyc03g095650  TTTATAAAGA TCAATACTTA TGAGTAAAAT TAACCAAGAG AAATGTCTTA 2450
cDNA_Solyc03g095650  ---------- ---------- ---------- ---------- ---------- 453
        SlMLO5_LEAF  ---------- ---------- ---------- ---------- ---------- 352
      SlMLO5_FLOWER  ---------- ---------- ---------- ---------- ---------- 352
       SlMLO5_FRUIT  ---------- ---------- ---------- ---------- ---------- 352
                                   2,460                 2,480                 2,500
gDNA_Solyc03g095650  TGTATATTGA TTTTAAAAAA TTATACTTTG AATATCTTTT TTTTCTACTT 2500
cDNA_Solyc03g095650  ---------- ---------- ---------- ---------- ---------- 453
        SlMLO5_LEAF  ---------- ---------- ---------- ---------- ---------- 352
      SlMLO5_FLOWER  ---------- ---------- ---------- ---------- ---------- 352
       SlMLO5_FRUIT  ---------- ---------- ---------- ---------- ---------- 352
                                              2,520                 2,540
gDNA_Solyc03g095650  TGAATACCAA GATAGAGCTC GTTGATCTAA ATAAATCATC CTTCCGGCCT 2550
cDNA_Solyc03g095650  ---------- ---------- ---------- ---------- ---------- 453
        SlMLO5_LEAF  ---------- ---------- ---------- ---------- ---------- 352
      SlMLO5_FLOWER  ---------- ---------- ---------- ---------- ---------- 352
       SlMLO5_FRUIT  ---------- ---------- ---------- ---------- ---------- 352
                                   2,560                 2,580                 2,600
gDNA_Solyc03g095650  CTCTCCGCTC CACACCCCGA AACTGACTCG AGTTCTTAGA CATTTGTTAA 2600
cDNA_Solyc03g095650  ---------- ---------- ---------- ---------- ---------- 453
        SlMLO5_LEAF  ---------- ---------- ---------- ---------- ---------- 352
      SlMLO5_FLOWER  ---------- ---------- ---------- ---------- ---------- 352
       SlMLO5_FRUIT  ---------- ---------- ---------- ---------- ---------- 352
                                              2,620                 2,640
gDNA_Solyc03g095650  TTACTAACGA AAATTTAGAT TAATCAGGTC AAAATTAATG AGTGTCAGTT 2650
cDNA_Solyc03g095650  ---------- ---------- ---------- ---------- ---------- 453
        SlMLO5_LEAF  ---------- ---------- ---------- ---------- ---------- 352
      SlMLO5_FLOWER  ---------- ---------- ---------- ---------- ---------- 352
       SlMLO5_FRUIT  ---------- ---------- ---------- ---------- ---------- 352
                                   2,660                 2,680                 2,700
gDNA_Solyc03g095650  AAGAAATGGT AAACAAAAAA AAAATGATTC CTTCTTTTAT TAAAGTCGAA 2700
cDNA_Solyc03g095650  ---------- ---------- ---------- ---------- ---------- 453
        SlMLO5_LEAF  ---------- ---------- ---------- ---------- ---------- 352
      SlMLO5_FLOWER  ---------- ---------- ---------- ---------- ---------- 352
       SlMLO5_FRUIT  ---------- ---------- ---------- ---------- ---------- 352
                                              2,720                 2,740
gDNA_Solyc03g095650  TACATATATT TGGTATTGAT CTAATATGAC CATAGAATAA ATATGTTGCT 2750
cDNA_Solyc03g095650  ---------- ---------- ---------- ---------- ---------- 453
        SlMLO5_LEAF  ---------- ---------- ---------- ---------- ---------- 352
      SlMLO5_FLOWER  ---------- ---------- ---------- ---------- ---------- 352
       SlMLO5_FRUIT  ---------- ---------- ---------- ---------- ---------- 352
                                   2,760                 2,780                 2,800
gDNA_Solyc03g095650  GACTTGCCTT ATATATTCTG ATTGAAATGC AGGGGAAACT CCAATTTGCT 2800
cDNA_Solyc03g095650  ---------- ---------- ---------- ---GGAAACT CCAATTTGCT 470
        SlMLO5_LEAF  ---------- ---------- ---------- ---GGAAACT CCAATTTGCT 369
      SlMLO5_FLOWER  ---------- ---------- ---------- ---GGAAACT CCAATTTGCT 369
       SlMLO5_FRUIT  ---------- ---------- ---------- ---GGAAACT CCAATTTGCT 369
```

```
                              2,820                2,840
gDNA_Solyc03g095650  TCAAAATATG CAATTCACCA ACTCCACATT TTTATCTTTG TGTTAGCCGT 2850
cDNA_Solyc03g095650  TCAAAATATG CAATTCACCA ACTCCACATT TTTATCTTTG TGTTAGCCGT 520
        SlMLO5_LEAF  TCAAAATATG CAATTCACCA ACTCCACATT TTTATCTTTG TGTTASCCGT 419
      SlMLO5_FLOWER  TCAAAATATG CAATTCACCA ACTCCACATT TTTATCTTTG TGTTAGCCGT 419
       SlMLO5_FRUIT  TCAAAATATG CAATTCACCA ACTCCACATT TTTATCTTTG TGTTAGCCGT 419
                  2,860                2,880                2,900
gDNA_Solyc03g095650  TGCACATGTG TTGTATTGTA TTACCACTTT GGGAATTGGC AAACTAAGGG 2900
cDNA_Solyc03g095650  TGCACATGTG TTGTATTGTA TTACCACTTT GGGAATTGGC AAACTAAGG- 569
        SlMLO5_LEAF  TGCACATGTG TTGTATTGTA TTACCACTTT GGGAATTGGC AAACTAAGG- 468
      SlMLO5_FLOWER  TGCACATGTG TTGTATTGTA TTACCACTTT GGGAATTGGC AAACTAAGG- 468
       SlMLO5_FRUIT  TGCACATGTG TTGTATTGTA TTACCACTTT GGGAATTGGC AAACTAAGG- 468
                              2,920                2,940
gDNA_Solyc03g095650  TAACTTCTTA AGATATATTT AATACTCCGT TTCTTTTATA TAATAATAAT 2950
cDNA_Solyc03g095650  ---------- ---------- ---------- ---------- ---------- 569
        SlMLO5_LEAF  ---------- ---------- ---------- ---------- ---------- 468
      SlMLO5_FLOWER  ---------- ---------- ---------- ---------- ---------- 468
       SlMLO5_FRUIT  ---------- ---------- ---------- ---------- ---------- 468
                  2,960                2,980                3,000
gDNA_Solyc03g095650  TATCCCAAGT ACTCACTCTT TTTTCTAAAA AAAAAATGAA TAAAATTTAT 3000
cDNA_Solyc03g095650  ---------- ---------- ---------- ---------- ---------- 569
        SlMLO5_LEAF  ---------- ---------- ---------- ---------- ---------- 468
      SlMLO5_FLOWER  ---------- ---------- ---------- ---------- ---------- 468
       SlMLO5_FRUIT  ---------- ---------- ---------- ---------- ---------- 468
                              3,020                3,040
gDNA_Solyc03g095650  GATTCTAAAA TACAGTTATG TCCTTAAATA TATCGCGTGA AAAACTGCAA 3050
cDNA_Solyc03g095650  ---------- ---------- ---------- ---------- ---------- 569
        SlMLO5_LEAF  ---------- ---------- ---------- ---------- ---------- 468
      SlMLO5_FLOWER  ---------- ---------- ---------- ---------- ---------- 468
       SlMLO5_FRUIT  ---------- ---------- ---------- ---------- ---------- 468
                  3,060                3,080                3,100
gDNA_Solyc03g095650  TTAAAGTGTT GTTAAAAAAT AAAAATGGTC ATTCTATTTT AAATAATTTT 3100
cDNA_Solyc03g095650  ---------- ---------- ---------- ---------- ---------- 569
        SlMLO5_LEAF  ---------- ---------- ---------- ---------- ---------- 468
      SlMLO5_FLOWER  ---------- ---------- ---------- ---------- ---------- 468
       SlMLO5_FRUIT  ---------- ---------- ---------- ---------- ---------- 468
                              3,120                3,140
gDNA_Solyc03g095650  TTTTTAAAAA AAAAACGATC ATTCTTTTTG TAACAAAATA GACGGTAATT 3150
cDNA_Solyc03g095650  ---------- ---------- ---------- ---------- ---------- 569
        SlMLO5_LEAF  ---------- ---------- ---------- ---------- ---------- 468
      SlMLO5_FLOWER  ---------- ---------- ---------- ---------- ---------- 468
       SlMLO5_FRUIT  ---------- ---------- ---------- ---------- ---------- 468
                  3,160                3,180                3,200
gDNA_Solyc03g095650  AATGATTTGT GTACATTGAA ATGCAGATGA GGACATGGAG AGCTTGGGAG 3200
cDNA_Solyc03g095650  ---------- ---------- ------ATGA GGACATGGAG AGCTTGGGAG 593
        SlMLO5_LEAF  ---------- ---------- ------ATGA GGACATGGAG AGCTTGGGAG 492
      SlMLO5_FLOWER  ---------- ---------- ------ATGA GGACATGGAG AGCTTGGGAG 492
       SlMLO5_FRUIT  ---------- ---------- ------ATGA GGACATGGAG AGCTTGGGAG 492
                              3,220                3,240
gDNA_Solyc03g095650  GATGAATCTA AAACAATTGA ATACCAATTC TATAACGGTT AGTTTTATTG 3250
cDNA_Solyc03g095650  GATGAATCTA AAACAATTGA ATACCAATTC TATAACG--- ---------- 630
        SlMLO5_LEAF  GATGAATCTA AAACAATTGA ATACCAATTC TATAACG--- ---------- 529
      SlMLO5_FLOWER  GATGAATCTA AAACAATTGA ATACCAATTC TATAACG--- ---------- 529
       SlMLO5_FRUIT  GATGAATCTA AAACAATTGA ATACCAATTC TATAACG--- ---------- 529
                  3,260                3,280                3,300
gDNA_Solyc03g095650  ATCAGGATTC AGAGCAATGT TCTTGTTAAT ATTTAATTAA AATTACTTCT 3300
cDNA_Solyc03g095650  ---------- ---------- ---------- ---------- ---------- 630
        SlMLO5_LEAF  ---------- ---------- ---------- ---------- ---------- 529
      SlMLO5_FLOWER  ---------- ---------- ---------- ---------- ---------- 529
       SlMLO5_FRUIT  ---------- ---------- ---------- ---------- ---------- 529
                              3,320                3,340
gDNA_Solyc03g095650  TAAAATAATT TATTCTAATT AACTTTTTTT GTGTTCAATT ATTTTATAGT 3350
cDNA_Solyc03g095650  ---------- ---------- ---------- ---------- ---------- 630
        SlMLO5_LEAF  ---------- ---------- ---------- ---------- ---------- 529
      SlMLO5_FLOWER  ---------- ---------- ---------- ---------- ---------- 529
       SlMLO5_FRUIT  ---------- ---------- ---------- ---------- ---------- 529
                  3,360                3,380                3,400
gDNA_Solyc03g095650  TCCACATTAC ATATGCTTAA AACATATTTT CACTTTTTCA CTTCATTTTT 3400
cDNA_Solyc03g095650  ---------- ---------- ---------- ---------- ---------- 630
        SlMLO5_LEAF  ---------- ---------- ---------- ---------- ---------- 529
      SlMLO5_FLOWER  ---------- ---------- ---------- ---------- ---------- 529
       SlMLO5_FRUIT  ---------- ---------- ---------- ---------- ---------- 529
                              3,420                3,440
gDNA_Solyc03g095650  TGATTGGTGA GTTCATAATA TATACAAATT AAAAGAGAAA AAAATATCAT 3450
cDNA_Solyc03g095650  ---------- ---------- ---------- ---------- ---------- 630
        SlMLO5_LEAF  ---------- ---------- ---------- ---------- ---------- 529
      SlMLO5_FLOWER  ---------- ---------- ---------- ---------- ---------- 529
       SlMLO5_FRUIT  ---------- ---------- ---------- ---------- ---------- 529
                  3,460                3,480                3,500
gDNA_Solyc03g095650  AATTTTTTTT GTTGTTGTTG CAGATCCTGA GAGATTTAGA TTTGCAAGAG 3500
cDNA_Solyc03g095650  ---------- ---------- ---ATCCTGA GAGATTTAGA TTTGCAAGAG 657
        SlMLO5_LEAF  ---------- ---------- ---ATCCTGA GAGATTTAGA TTTGCAAGAG 556
      SlMLO5_FLOWER  ---------- ---------- ---ATCCTGA GAGATTTAGA TTTGCAAGAG 556
       SlMLO5_FRUIT  ---------- ---------- ---ATCCTGA GAGATTTAGA TTTGCAAGAG 556
```

```
                                         3,520                 3,540
gDNA_Solyc03g095650  AAACATCATT TGGACGTAAA CATTTGCATT TCTGGAGCAA CTCTCCAATT 3550
cDNA_Solyc03g095650  AAACATCATT TGGACGTAAA CATTTGCATT TCTGGAGCAA CTCTCCAATT 707
        SIMLO5_LEAF  AAACATCATT TGGACGTAAA CATTTGCATT TCTGGAGCAA CTCTCCAATT 606
      SIMLO5_FLOWER  AAACATCATT TGGACGTAAA CATTTGCATT TCTGGAGCAA CTCTCCAATT 606
       SIMLO5_FRUIT  AAACATCATT TGGACGTAAA CATTTGCATT TCTGGAGCAA CTCTCCAATT 606

                              3,560                 3,580                 3,600
gDNA_Solyc03g095650  CTTCTCTGGA TAGTATGATT TAATATTTTT TTTCTTTTTT ATTTCCTCTA 3600
cDNA_Solyc03g095650  CTTCTCTGGA TAGT------ ---------- ---------- ---------- 721
        SIMLO5_LEAF  CTTCTCTGGA TAGT------ ---------- ---------- ---------- 620
      SIMLO5_FLOWER  CTTCTCTGGA TAGT------ ---------- ---------- ---------- 620
       SIMLO5_FRUIT  CTTCTCTGGA TAGT------ ---------- ---------- ---------- 620

                                         3,620                 3,640
gDNA_Solyc03g095650  ATTTAAAAGC TATAAAATTT AAATTTTTTT GTTAATTAAA AATGTTTGTT 3650
cDNA_Solyc03g095650  ---------- ---------- ---------- ---------- ---------- 721
        SIMLO5_LEAF  ---------- ---------- ---------- ---------- ---------- 620
      SIMLO5_FLOWER  ---------- ---------- ---------- ---------- ---------- 620
       SIMLO5_FRUIT  ---------- ---------- ---------- ---------- ---------- 620

                              3,660                 3,680                 3,700
gDNA_Solyc03g095650  TTTTTAAAAA TATGGTTGAT TTGTTCCAGG TTTGTTTCTT CAGACAGTTC 3700
cDNA_Solyc03g095650  ---------- ---------- ---------- -TTGTTTCTT CAGACAGTTC 740
        SIMLO5_LEAF  ---------- ---------- ---------- -TTGTTTCTT CAGACAGTTC 639
      SIMLO5_FLOWER  ---------- ---------- ---------- -TTGTTTCTT CAGACAGTTC 639
       SIMLO5_FRUIT  ---------- ---------- ---------- -TTGTTTCTT CAGACAGTTC 639

                                         3,720                 3,740
gDNA_Solyc03g095650  TATGCATCAG TTGAAAAAGT AGACTATCTT ACCCTTAGAC ATGGCTTTGC 3750
cDNA_Solyc03g095650  TATGCATCAG TTGAAAAAGT AGACTATCTT ACCCTTAGAC ATGGCTTTGC 790
        SIMLO5_LEAF  TATGCATCAG TTGAAAAAGT AGACTATCTT ACCCTTAGAC ATGGCTTTGC 689
      SIMLO5_FLOWER  TATGCATCAG TTGAAAAAGT AGACTATCTT ACCCTTAGAC ATGGCTTTGC 689
       SIMLO5_FRUIT  TATGCATCAG TTGAAAAAGT AGACTATCTT ACCCTTAGAC ATGGCTTTGC 689

                              3,760                 3,780                 3,800
gDNA_Solyc03g095650  TATGGTAATT AATTTTGATA CAACTTATTA AAATTATTAG ATAGTGGGAA 3800
cDNA_Solyc03g095650  TATGG----- ---------- ---------- ---------- ---------- 795
        SIMLO5_LEAF  TATGG----- ---------- ---------- ---------- ---------- 694
      SIMLO5_FLOWER  TATGG----- ---------- ---------- ---------- ---------- 694
       SIMLO5_FRUIT  TATGG----- ---------- ---------- ---------- ---------- 694

                                         3,820                 3,840
gDNA_Solyc03g095650  ATATTAATTT TAATATTGAT GATATTATTT TGGATGATGA CAGGCACATT 3850
cDNA_Solyc03g095650  ---------- ---------- ---------- ---------- ----CACATT 801
        SIMLO5_LEAF  ---------- ---------- ---------- ---------- ----CACATT 700
      SIMLO5_FLOWER  ---------- ---------- ---------- ---------- ----CACATT 700
       SIMLO5_FRUIT  ---------- ---------- ---------- ---------- ----CACATT 700

                              3,860                 3,880                 3,900
gDNA_Solyc03g095650  TAGCACCTCA GCAAGAAAAG AATTTTGATT TTCAATTGTA TATAAATAGA 3900
cDNA_Solyc03g095650  TAGCACCTCA GCAAGAAAAG AATTTTGATT TTCAATTGTA TATAAATAGA 851
        SIMLO5_LEAF  TAGCACCTCA GCAAGAAAAG AATTTTGATT TTCAATTGTA TATAAATAGA 750
      SIMLO5_FLOWER  TAGCACCTCA GCAAGAAAAG AATTTTGATT TTCAATTGTA TATAAATAGA 750
       SIMLO5_FRUIT  TAGCACCTCA GCAAGAAAAG AATTTTGATT TTCAATTGTA TATAAATAGA 750

                                         3,920                 3,940
gDNA_Solyc03g095650  GCACTTGAAG AAGATTTTAA AGATGTTGTG GGAATAAGGT TAGCTAAACA 3950
cDNA_Solyc03g095650  GCACTTGAAG AAGATTTTAA AGATGTTGTG GGAATAAG-- ---------- 889
        SIMLO5_LEAF  GCACTTGAAG AAGATTTTAA AGATGTTGTG GGAATAAG-- ---------- 788
      SIMLO5_FLOWER  GCACTTGAAG AAGATTTTAA AGATGTTGTG GGAATAAG-- ---------- 788
       SIMLO5_FRUIT  GCACTTGAAG AAGATTTTAA AGATGTTGTG GGAATAAG-- ---------- 788

                              3,960                 3,980                 4,000
gDNA_Solyc03g095650  AATCATAATT ATTACTTTTT TTTCTCTTCA AATAGTAAAA AAAATGTAGT 4000
cDNA_Solyc03g095650  ---------- ---------- ---------- -----T---- ---------- 890
        SIMLO5_LEAF  ---------- ---------- ---------- -----T---- ---------- 789
      SIMLO5_FLOWER  ---------- ---------- ---------- -----T---- ---------- 789
       SIMLO5_FRUIT  ---------- ---------- ---------- -----TAAAA AAAATGTAGT 803

                                         4,020                 4,040
gDNA_Solyc03g095650  ATATATTATT CATGAGAATT TCACCCTCTT GTATGTGGCA GTCCACTGTT 4050
cDNA_Solyc03g095650  ---------- ---------- ---------- ---------- --CCACTGTT 898
        SIMLO5_LEAF  ---------- ---------- ---------- ---------- --CCACTGTT 797
      SIMLO5_FLOWER  ---------- ---------- ---------- ---------- --CCACTGTT 797
       SIMLO5_FRUIT  ATATATTATT CATGAGAATT TCACCCTCTT GTATGTGGCA GTCCACTGTT 853

                              4,060                 4,080                 4,100
gDNA_Solyc03g095650  ATGGATGTTT GCAGTCCTCT ACTTTCTCAC TACTACCAAT GGTAAGCTAC 4100
cDNA_Solyc03g095650  ATGGATGTTT GCAGTCCTCT ACTTTCTCAC TACTACCAAT G--------- 939
        SIMLO5_LEAF  ATGGATGTTT GCAGCCCTCT ACTTTCTCAC TACTACCAAT G--------- 838
      SIMLO5_FLOWER  ATGGATGTTT GCAGYCCTCT ACTTTCTCAC TACTACCAAT G--------- 838
       SIMLO5_FRUIT  ATGGATGTTT GCAGYCCTCT ACTTTCTCAC TACTACCAAT G--------- 894

                                         4,120                 4,140
gDNA_Solyc03g095650  ATTGAGATAA AAAAAAGTTT GGATTTAATT ATATGAATGA AATAAAAATG 4150
cDNA_Solyc03g095650  ---------- ---------- ---------- ---------- ---------- 939
        SIMLO5_LEAF  ---------- ---------- ---------- ---------- ---------- 838
      SIMLO5_FLOWER  ---------- ---------- ---------- ---------- ---------- 838
       SIMLO5_FRUIT  ---------- ---------- ---------- ---------- ---------- 894

                              4,160                 4,180                 4,200
gDNA_Solyc03g095650  ATTAATTTTG ATTTGAATCG TTTGATAACT GAGAAGAGTT TTAATGATAT 4200
cDNA_Solyc03g095650  ---------- ---------- ---------- ---------- ---------- 939
        SIMLO5_LEAF  ---------- ---------- ---------- ---------- ---------- 838
      SIMLO5_FLOWER  ---------- ---------- ---------- ---------- ---------- 838
       SIMLO5_FRUIT  ---------- ---------- ---------- ---------- ---------- 894
```

```
                                             4,220                         4,240
gDNA_Solyc03g095650  TAGTAATGTA TAAATTAGCT ATGAGGGAAT CTATATCAGT ATTAGTTATT 4250
cDNA_Solyc03g095650  ---------- ---------- ---------- ---------- ---------- 939
         SlMLO5_LEAF ---------- ---------- ---------- ---------- ---------- 838
       SlMLO5_FLOWER ---------- ---------- ---------- ---------- ---------- 838
        SlMLO5_FRUIT ---------- ---------- ---------- ---------- ---------- 894
                               4,260                         4,280                4,300
gDNA_Solyc03g095650  CCACCCTTCA TTCTATCTAT CTATTTTATA ATTGATTAAT AACTTATGGG 4300
cDNA_Solyc03g095650  ---------- ---------- ---------- ---------- ---------- 939
         SlMLO5_LEAF ---------- ---------- ---------- ---------- ---------- 838
       SlMLO5_FLOWER ---------- ---------- ---------- ---------- ---------- 838
        SlMLO5_FRUIT ---------- ---------- ---------- ---------- ---------- 894
                                             4,320                         4,340
gDNA_Solyc03g095650  GGTCGTTTAG TAATTGCATA AAATAAACTT TATTCATATA TTCGAGTTTG 4350
cDNA_Solyc03g095650  ---------- ---------- ---------- ---------- ---------- 939
         SlMLO5_LEAF ---------- ---------- ---------- ---------- ---------- 838
       SlMLO5_FLOWER ---------- ---------- ---------- ---------- ---------- 838
        SlMLO5_FRUIT ---------- ---------- ---------- ---------- ---------- 894
                               4,360                         4,380                4,400
gDNA_Solyc03g095650  TATAACTTAT ACGATATTTG GTAGGTAGAT AACAAAAAAG TTATTCATGT 4400
cDNA_Solyc03g095650  ---------- ---------- ---------- ---------- ---------- 939
         SlMLO5_LEAF ---------- ---------- ---------- ---------- ---------- 838
       SlMLO5_FLOWER ---------- ---------- ---------- ---------- ---------- 838
        SlMLO5_FRUIT ---------- ---------- ---------- ---------- ---------- 894
                                             4,420                         4,440
gDNA_Solyc03g095650  ATATAGTCGA TACGATGTTT GGTAGATAGA TAAGAAAAAA ATTATTCATG 4450
cDNA_Solyc03g095650  ---------- ---------- ---------- ---------- ---------- 939
         SlMLO5_LEAF ---------- ---------- ---------- ---------- ---------- 838
       SlMLO5_FLOWER ---------- ---------- ---------- ---------- ---------- 838
        SlMLO5_FRUIT ---------- ---------- ---------- ---------- ---------- 894
                               4,460                         4,480                4,500
gDNA_Solyc03g095650  CATAAAATTA ATACGACATT TGATAGATAG ATAAAAAAAA ATTCATATAT 4500
cDNA_Solyc03g095650  ---------- ---------- ---------- ---------- ---------- 939
         SlMLO5_LEAF ---------- ---------- ---------- ---------- ---------- 838
       SlMLO5_FLOWER ---------- ---------- ---------- ---------- ---------- 838
        SlMLO5_FRUIT ---------- ---------- ---------- ---------- ---------- 894
                                             4,520                         4,540
gDNA_Solyc03g095650  AAAATTAATA CAACGCTTGA TTAACAATTT TTAAAATTAA CATAACTAAT 4550
cDNA_Solyc03g095650  ---------- ---------- ---------- ---------- ---------- 939
         SlMLO5_LEAF ---------- ---------- ---------- ---------- ---------- 838
       SlMLO5_FLOWER ---------- ---------- ---------- ---------- ---------- 838
        SlMLO5_FRUIT ---------- ---------- ---------- ---------- ---------- 894
                               4,560                         4,580                4,600
gDNA_Solyc03g095650  ATATATATAA GTTACCAGAA AATTTATGTA ATATTTATGC GCAGTAAAAA 4600
cDNA_Solyc03g095650  ---------- ---------- ---------- ---------- ---------- 939
         SlMLO5_LEAF ---------- ---------- ---------- ---------- ---------- 838
       SlMLO5_FLOWER ---------- ---------- ---------- ---------- ---------- 838
        SlMLO5_FRUIT ---------- ---------- ---------- ---------- ---------- 894
                                             4,620                         4,640
gDNA_Solyc03g095650  GTGAAATAAT TAATAAGTAC GGCTAACACA TGAATAAAAA GTTAAAATGA 4650
cDNA_Solyc03g095650  ---------- ---------- ---------- ---------- ---------- 939
         SlMLO5_LEAF ---------- ---------- ---------- ---------- ---------- 838
       SlMLO5_FLOWER ---------- ---------- ---------- ---------- ---------- 838
        SlMLO5_FRUIT ---------- ---------- ---------- ---------- ---------- 894
                               4,660                         4,680                4,700
gDNA_Solyc03g095650  CAGATCTTAT CATTCCCACT TGATTACTTT CTTCTTCTAC ATAATTAAAG 4700
cDNA_Solyc03g095650  ---------- ---------- ---------- ---------- ---------- 939
         SlMLO5_LEAF ---------- ---------- ---------- ---------- ---------- 838
       SlMLO5_FLOWER ---------- ---------- ---------- ---------- ---------- 838
        SlMLO5_FRUIT ---------- ---------- ---------- ---------- ---------- 894
                                             4,720                         4,740
gDNA_Solyc03g095650  AAATGTTTTA ATTTTGATAC AAAGTTAAAG AATGAAAGAA TATAATTGTA 4750
cDNA_Solyc03g095650  ---------- ---------- ---------- ---------- ---------- 939
         SlMLO5_LEAF ---------- ---------- ---------- ---------- ---------- 838
       SlMLO5_FLOWER ---------- ---------- ---------- ---------- ---------- 838
        SlMLO5_FRUIT ---------- ---------- ---------- ---------- ---------- 894
                               4,760                         4,780                4,800
gDNA_Solyc03g095650  AAATAAAATG TTTTAATTTA AAAAAATATA ATTTTACTAA TTTTTAATAT 4800
cDNA_Solyc03g095650  ---------- ---------- ---------- ---------- ---------- 939
         SlMLO5_LEAF ---------- ---------- ---------- ---------- ---------- 838
       SlMLO5_FLOWER ---------- ---------- ---------- ---------- ---------- 838
        SlMLO5_FRUIT ---------- ---------- ---------- ---------- ---------- 894
                                             4,820                         4,840
gDNA_Solyc03g095650  ATAAAAACGA CATTCAAACA AATAATTTAT GCATAACTAA ACTCCACATA 4850
cDNA_Solyc03g095650  ---------- ---------- ---------- ---------- ---------- 939
         SlMLO5_LEAF ---------- ---------- ---------- ---------- ---------- 838
       SlMLO5_FLOWER ---------- ---------- ---------- ---------- ---------- 838
        SlMLO5_FRUIT ---------- ---------- ---------- ---------- ---------- 894
                               4,860                         4,880                4,900
gDNA_Solyc03g095650  ATAAACCTAC ATAGCTGATA TTTATATAAC TAATACCTGC ATAACTAATA 4900
cDNA_Solyc03g095650  ---------- ---------- ---------- ---------- ---------- 939
         SlMLO5_LEAF ---------- ---------- ---------- ---------- ---------- 838
       SlMLO5_FLOWER ---------- ---------- ---------- ---------- ---------- 838
        SlMLO5_FRUIT ---------- ---------- ---------- ---------- ---------- 894
```

```
                                          4,920                          4,940
gDNA_Solyc03g095650  TCTGCACAAC TAGTACAAAC ATAACTAATA CTTCCATTAT TTATTTGCAT 4950
cDNA_Solyc03g095650  ---------- ---------- ---------- ---------- ---------- 939
        SIMLO5_LEAF  ---------- ---------- ---------- ---------- ---------- 838
      SIMLO5_FLOWER  ---------- ---------- ---------- ---------- ---------- 838
       SIMLO5_FRUIT  ---------- ---------- ---------- ---------- ---------- 894
                               4,960                          4,980                     5,000
gDNA_Solyc03g095650  AACTAGTATT CACATTACTA ATATATGTAT AACTTTATCC AGTCACCAAA 5000
cDNA_Solyc03g095650  ---------- ---------- ---------- ---------- ---------- 939
        SIMLO5_LEAF  ---------- ---------- ---------- ---------- ---------- 838
      SIMLO5_FLOWER  ---------- ---------- ---------- ---------- ---------- 838
       SIMLO5_FRUIT  ---------- ---------- ---------- ---------- ---------- 894
                                          5,020                          5,040
gDNA_Solyc03g095650  CGATACCCTA AACATTTTAA ACAACCATTA AATATGATAT TACTTATACA 5050
cDNA_Solyc03g095650  ---------- ---------- ---------- ---------- ---------- 939
        SIMLO5_LEAF  ---------- ---------- ---------- ---------- ---------- 838
      SIMLO5_FLOWER  ---------- ---------- ---------- ---------- ---------- 838
       SIMLO5_FRUIT  ---------- ---------- ---------- ---------- ---------- 894
                               5,060                          5,080                     5,100
gDNA_Solyc03g095650  TAATTTAATA TCTCCATAAC TAAATGACCC AAAATATTTT GGATCATATA 5100
cDNA_Solyc03g095650  ---------- ---------- ---------- ---------- ---------- 939
        SIMLO5_LEAF  ---------- ---------- ---------- ---------- ---------- 838
      SIMLO5_FLOWER  ---------- ---------- ---------- ---------- ---------- 838
       SIMLO5_FRUIT  ---------- ---------- ---------- ---------- ---------- 894
                                          5,120                          5,140
gDNA_Solyc03g095650  TTTTAATTGT TACTAAATAG ATTTGTGCTT CAGGTTGGTA TTCATACTAT 5150
cDNA_Solyc03g095650  ---------- ---------- ---------- ---GTTGGTA TTCATACTAT 956
        SIMLO5_LEAF  ---------- ---------- ---------- ---GTTGGTA TTCATACTAT 855
      SIMLO5_FLOWER  ---------- ---------- ---------- ---GTTGGTA TTCATACTAT 855
       SIMLO5_FRUIT  ---------- ---------- ATTTGTGCTT CAGGTTGGTA TTCATACTAT 924
                               5,160                          5,180                     5,200
gDNA_Solyc03g095650  TGGCTGCCGT TCATTCCTTT AATTGTAAGT CAATCCAGAG TACCACATAT 5200
cDNA_Solyc03g095650  TGGCTGCCGT TCATTCCTTT AATT------ ---------- ---------- 980
        SIMLO5_LEAF  TGGCTGCCGT TCATTCCTTT AATT------ ---------- ---------- 879
      SIMLO5_FLOWER  TGGCTGCCGT TCATTCCTTT AATT------ ---------- ---------- 879
       SIMLO5_FRUIT  TGGCTGCCGT TCATTCCTTT AATT------ ---------- ---------- 948
                                          5,220                          5,240
gDNA_Solyc03g095650  TTTCTATTCT TATATTAAAA TTTCTAATTC TACTATTAAT TAATGTGTTT 5250
cDNA_Solyc03g095650  ---------- ---------- ---------- ---------- ---------- 980
        SIMLO5_LEAF  ---------- ---------- ---------- ---------- ---------- 879
      SIMLO5_FLOWER  ---------- ---------- ---------- ---------- ---------- 879
       SIMLO5_FRUIT  ---------- ---------- ---------- ---------- ---------- 948
                               5,260                          5,280                     5,300
gDNA_Solyc03g095650  CATTATGTAA TTAAAGTAAT TTTTTTGCAG ATAATATTAC TGGTGGGCAC 5300
cDNA_Solyc03g095650  ---------- ---------- ---------- ATAATATTAC TGGTGGGCAC 1000
        SIMLO5_LEAF  ---------- ---------- ---------- ATAATATTAC TGGTGGGCAC 899
      SIMLO5_FLOWER  ---------- ---------- ---------- ATAATATTAC TGGTGGGCAC 899
       SIMLO5_FRUIT  ---------- ---------- ---------- ATAATATTAC TGGTGGGCAC 968
                                          5,320                          5,340
gDNA_Solyc03g095650  AAAATTACAA GTGATTATAA CAAAAATGGG ATTAAGGATT AAAGAAAGAG 5350
cDNA_Solyc03g095650  AAAATTACAA GTGATTATAA CAAAAATGGG ATTAAGGATT AAAGAAAGAG 1050
        SIMLO5_LEAF  AAAATTACAA GTGATTATAA CAAAAATGGG ATTAAGGATT AAAGAAAGAG 949
      SIMLO5_FLOWER  AAAATTACAA GTGATTATAA CAAAAATGGG ATTAAGGATT AAAGAAAGAG 949
       SIMLO5_FRUIT  AAAATTACAA GTGATTATAA CAAAAATGGG ATTAAGGATT AAAGAAAGAG 1018
                               5,360                          5,380                     5,400
gDNA_Solyc03g095650  GAGACATTGT TAAAGGAACA CCATTAGTTG AACCAGGGGA TGATCTTTTC 5400
cDNA_Solyc03g095650  GAGACATTGT TAAAGGAACA CCATTAGTTG AACCAGGGGA TGATCTTTTC 1100
        SIMLO5_LEAF  GAGACATTGT TAAAGGAACA CCATTAGTTG AACCAGGGGA TGATCTTTTC 999
      SIMLO5_FLOWER  GAGACATTGT TAAAGGAACA CCATTAGTTG AACCAGGGGA TGATCTTTTC 999
       SIMLO5_FRUIT  GAGACATTGT TAAAGGAACA CCATTAGTTG AACCAGGGGA TGATCTTTTC 1068
                                          5,420                          5,440
gDNA_Solyc03g095650  TGGTTTAATC GTCCTGATCT TTTGCACTTC TTCATTCACT TTGTTCTCTT 5450
cDNA_Solyc03g095650  TGGTTTAATC GTCCTGATCT TTTGCACTTC TTCATTCACT TTGTTCTCTT 1150
        SIMLO5_LEAF  TGGTTTAATC GTCCTGATCT TTTGCACTTC TTCATTCACT TTGTTCTCTT 1049
      SIMLO5_FLOWER  TGGTTTAATC GTCCTGATCT TTTGCACTTC TTCATTCACT TTGTTCTCTT 1049
       SIMLO5_FRUIT  TGGTTTAATC GTCCTGATCT TTTGCACTTC TTCATTCACT TTGTTCTCTT 1118
                               5,460                          5,480                     5,500
gDNA_Solyc03g095650  TCAGGTAATA ATTAAGTCAT AATACATAAA TGTGCCTTTT AACTTGGTTT 5500
cDNA_Solyc03g095650  TCAG------ ---------- ---------- ---------- ---------- 1154
        SIMLO5_LEAF  TCAG------ ---------- ---------- ---------- ---------- 1053
      SIMLO5_FLOWER  TCAG------ ---------- ---------- ---------- ---------- 1053
       SIMLO5_FRUIT  TCAG------ ---------- ---------- ---------- ---------- 1122
                                          5,520                          5,540
gDNA_Solyc03g095650  CAAATTACAT TTATGCCATT TAATTTTGGA TATGCACAAA TGGATATTTA 5550
cDNA_Solyc03g095650  ---------- ---------- ---------- ---------- ---------- 1154
        SIMLO5_LEAF  ---------- ---------- ---------- ---------- ---------- 1053
      SIMLO5_FLOWER  ---------- ---------- ---------- ---------- ---------- 1053
       SIMLO5_FRUIT  ---------- ---------- ---------- ---------- ---------- 1122
                               5,560                          5,580                     5,600
gDNA_Solyc03g095650  AACTTGTATA AAGTTGAACA AATAGATACA CATATCCTAC GTGTCATCTT 5600
cDNA_Solyc03g095650  ---------- ---------- ---------- ---------- ---------- 1154
        SIMLO5_LEAF  ---------- ---------- ---------- ---------- ---------- 1053
      SIMLO5_FLOWER  ---------- ---------- ---------- ---------- ---------- 1053
       SIMLO5_FRUIT  ---------- ---------- ---------- ---------- ---------- 1122
```

```
                                        5,620                 5,640
gDNA_Solyc03g095650  ACATGTCATT TTTTGTCCTA CGTAATGTCC TACATGTATT GTTACATGTA 5650
cDNA_Solyc03g095650  ---------- ---------- ---------- ---------- ---------- 1154
       SIMLO5_LEAF   ---------- ---------- ---------- ---------- ---------- 1053
     SIMLO5_FLOWER   ---------- ---------- ---------- ---------- ---------- 1053
      SIMLO5_FRUIT   ---------- ---------- ---------- ---------- ---------- 1122
                              5,660                 5,680                 5,700
gDNA_Solyc03g095650  GAACTCATGA GTTTATTTAT CTAAAAGTTG GATAGTTAAA GTGTCTGTTT 5700
cDNA_Solyc03g095650  ---------- ---------- ---------- ---------- ---------- 1154
       SIMLO5_LEAF   ---------- ---------- ---------- ---------- ---------- 1053
     SIMLO5_FLOWER   ---------- ---------- ---------- ---------- ---------- 1053
      SIMLO5_FRUIT   ---------- ---------- ---------- ---------- ---------- 1122
                                        5,720                 5,740
gDNA_Solyc03g095650  GTGCATTATG AAAATTGAAG GTCAACGTTA AAATTTGAAA TCAAGTTTAT 5750
cDNA_Solyc03g095650  ---------- ---------- ---------- ---------- ---------- 1154
       SIMLO5_LEAF   ---------- ---------- ---------- ---------- ---------- 1053
     SIMLO5_FLOWER   ---------- ---------- ---------- ---------- ---------- 1053
      SIMLO5_FRUIT   ---------- ---------- ---------- ---------- ---------- 1122
                              5,760                 5,780                 5,800
gDNA_Solyc03g095650  GATCCAATAT ATATCTTATG TCAATAATTA ATTACACTTA CTAATTAATG 5800
cDNA_Solyc03g095650  ---------- ---------- ---------- ---------- ---------- 1154
       SIMLO5_LEAF   ---------- ---------- ---------- ---------- ---------- 1053
     SIMLO5_FLOWER   ---------- ---------- ---------- ---------- ---------- 1053
      SIMLO5_FRUIT   ---------- ---------- ---------- ---------- ---------- 1122
                                        5,820                 5,840
gDNA_Solyc03g095650  GATCCGAAAT CAACGTTTGT TTATGATATC AATCTGTATA AATTTCAACT 5850
cDNA_Solyc03g095650  ---------- ---------- ---------- ---------- ---------- 1154
       SIMLO5_LEAF   ---------- ---------- ---------- ---------- ---------- 1053
     SIMLO5_FLOWER   ---------- ---------- ---------- ---------- ---------- 1053
      SIMLO5_FRUIT   ---------- ---------- ---------- ---------- ---------- 1122
                              5,860                 5,880                 5,900
gDNA_Solyc03g095650  TCAAATCCCG AATCGAGAAT TTGATGATAA TATGTTTGTC TAATAATTAC 5900
cDNA_Solyc03g095650  ---------- ---------- ---------- ---------- ---------- 1154
       SIMLO5_LEAF   ---------- ---------- ---------- ---------- ---------- 1053
     SIMLO5_FLOWER   ---------- ---------- ---------- ---------- ---------- 1053
      SIMLO5_FRUIT   ---------- ---------- ---------- ---------- ---------- 1122
                                        5,920                 5,940
gDNA_Solyc03g095650  TACTACTACT ACTTTGTTTG GTTTTCCAGA ATGCATATCA ACTTGCTTTC 5950
cDNA_Solyc03g095650  ---------- ---------- ---------A ATGCATATCA ACTTGCTTTC 1175
       SIMLO5_LEAF   ---------- ---------- ---------A ATGCATATCA ACTTGCTTTC 1074
     SIMLO5_FLOWER   ---------- ---------- ---------A ATGCATATCA ACTTGCTTTC 1074
      SIMLO5_FRUIT   ---------- ---------- ---------A ATGCATATCA ACTTGCTTTC 1143
                              5,960                 5,980                 6,000
gDNA_Solyc03g095650  TTTGCTTGGA GCTGGGTAAG TAGCTTAATT ATTGAATTTT AACTAGTATT 6000
cDNA_Solyc03g095650  TTTGCTTGGA GCTGG----- ---------- ---------- ---------- 1190
       SIMLO5_LEAF   TTTGCTTGGA GCTGG----- ---------- ---------- ---------- 1089
     SIMLO5_FLOWER   TTTGCTTGGA GCTGG----- ---------- ---------- ---------- 1089
      SIMLO5_FRUIT   TTTGCTTGGA GCTGG----- ---------- ---------- ---------- 1158
                                        6,020                 6,040
gDNA_Solyc03g095650  ATATATATGT ATCATCATTT TTTTTTTTTG TGCAGTGGAA ATTTAATTTA 6050
cDNA_Solyc03g095650  ---------- ---------- ---------- -----TGGAA ATTTAATTTA 1205
       SIMLO5_LEAF   ---------- ---------- ---------- -----TGGAA ATTTAATTTA 1104
     SIMLO5_FLOWER   ---------- ---------- ---------- -----TGGAA ATTTAATTTA 1104
      SIMLO5_FRUIT   ---------- ---------- ---------- -----TGGAA ATTTAATTTA 1173
                              6,060                 6,080                 6,100
gDNA_Solyc03g095650  CCATCTTGCT TCCACAAAAA TGTAACAGAC ATAGCCATAA CACTTTCCAT 6100
cDNA_Solyc03g095650  CCATCTTGCT TCCACAAAAA TGTAACAGAC ATAGCCATAA CACTTTCCAT 1255
       SIMLO5_LEAF   CCATCTTGCT TCCACAAAAA TGTAACAGAC ATAGCCATAA CACTTTCCAT 1154
     SIMLO5_FLOWER   CCATCTTGCT TCCACAAAAA TGTAACAGAC ATAGCCATAA CACTTTCCAT 1154
      SIMLO5_FRUIT   CCATCTTGCT TCCACAAAAA TGTAACAGAC ATAGCCATAA CACTTTCCAT 1223
                                        6,120                 6,140
gDNA_Solyc03g095650  GGGGTGAGCA TTTTTACTAC TCCCTCCGGT CTCATTTTAT ATATGAATTA 6150
cDNA_Solyc03g095650  GGGG------ ---------- ---------- ---------- ---------- 1259
       SIMLO5_LEAF   GGGG------ ---------- ---------- ---------- ---------- 1158
     SIMLO5_FLOWER   GGGG------ ---------- ---------- ---------- ---------- 1158
      SIMLO5_FRUIT   GGGG------ ---------- ---------- ---------- ---------- 1227
                              6,160                 6,180                 6,200
gDNA_Solyc03g095650  GTTTAATTTA ATGCATGAAA GTTTAAAAGG GAAAGAGCCA CTTTTGAGAT 6200
cDNA_Solyc03g095650  ---------- ---------- ---------- ---------- ---------- 1259
       SIMLO5_LEAF   ---------- ---------- ---------- ---------- ---------- 1158
     SIMLO5_FLOWER   ---------- ---------- ---------- ---------- ---------- 1158
      SIMLO5_FRUIT   ---------- ---------- ---------- ---------- ---------- 1227
                                        6,220                 6,240
gDNA_Solyc03g095650  TGTGGTCTAA ATAAGGTATA GATGATTGTA TGTGTATAAA TCATTTTATT 6250
cDNA_Solyc03g095650  ---------- ---------- ---------- ---------- ---------- 1259
       SIMLO5_LEAF   ---------- ---------- ---------- ---------- ---------- 1158
     SIMLO5_FLOWER   ---------- ---------- ---------- ---------- ---------- 1158
      SIMLO5_FRUIT   ---------- ---------- ---------- ---------- ---------- 1227
                              6,260                 6,280                 6,300
gDNA_Solyc03g095650  AAAAATAAAA TAAGCATTTT AAAGTTAAAT TGTTACTTAA TATAGAAAAA 6300
cDNA_Solyc03g095650  ---------- ---------- ---------- ---------- ---------- 1259
       SIMLO5_LEAF   ---------- ---------- ---------- ---------- ---------- 1158
     SIMLO5_FLOWER   ---------- ---------- ---------- ---------- ---------- 1158
      SIMLO5_FRUIT   ---------- ---------- ---------- ---------- ---------- 1227
```

```
                                          6,320                                   6,340
gDNA_Solyc03g095650  TATCTCATTA  TTTTTTAGAT  TTATGCGGAA  AAACTCATTA  TCTTTTCTTT  6350
cDNA_Solyc03g095650  ----------  ----------  ----------  ----------  ----------  1259
        SlMLO5_LEAF  ----------  ----------  ----------  ----------  ----------  1158
      SlMLO5_FLOWER  ----------  ----------  ----------  ----------  ----------  1158
       SlMLO5_FRUIT  ----------  ----------  ----------  ----------  ----------  1227
                              6,360                                   6,380                  6,400
gDNA_Solyc03g095650  ATAACTCAAA  TTTTATAACT  TATTACTTTT  ACCTAATTAA  ACAAAGATTA  6400
cDNA_Solyc03g095650  ----------  ----------  ----------  ----------  ----------  1259
        SlMLO5_LEAF  ----------  ----------  ----------  ----------  ----------  1158
      SlMLO5_FLOWER  ----------  ----------  ----------  ----------  ----------  1158
       SlMLO5_FRUIT  ----------  ----------  ----------  ----------  ----------  1227
                                          6,420                                   6,440
gDNA_Solyc03g095650  AAACGATAGT  CGTCAATATG  AGCTAGATCT  GTTAGGCTGG  TGTGGCTTAA  6450
cDNA_Solyc03g095650  ----------  ----------  ----------  ----------  ----------  1259
        SlMLO5_LEAF  ----------  ----------  ----------  ----------  ----------  1158
      SlMLO5_FLOWER  ----------  ----------  ----------  ----------  ----------  1158
       SlMLO5_FRUIT  ----------  ----------  ----------  ----------  ----------  1227
                              6,460                                   6,480                  6,500
gDNA_Solyc03g095650  CCCGTGATTT  GGTAGGGATG  AATTATGATT  TTTAAAGTCC  ATTTAAGAAA  6500
cDNA_Solyc03g095650  ----------  ----------  ----------  ----------  ----------  1259
        SlMLO5_LEAF  ----------  ----------  ----------  ----------  ----------  1158
      SlMLO5_FLOWER  ----------  ----------  ----------  ----------  ----------  1158
       SlMLO5_FRUIT  ----------  ----------  ----------  ----------  ----------  1227
                                          6,520                                   6,540
gDNA_Solyc03g095650  AAGGTTTTTC  AGCCTAGCCT  GAATAAGCTT  GTCGATTTGT  GGAGCTTGAG  6550
cDNA_Solyc03g095650  ----------  ----------  ----------  ----------  ----------  1259
        SlMLO5_LEAF  ----------  ----------  ----------  ----------  ----------  1158
      SlMLO5_FLOWER  ----------  ----------  ----------  ----------  ----------  1158
       SlMLO5_FRUIT  ----------  ----------  ----------  ----------  ----------  1227
                              6,560                                   6,580                  6,600
gDNA_Solyc03g095650  AAATATAGAT  GAGACCGACT  CGTGAGTCTA  AAACAATTAA  TTAAAATAAA  6600
cDNA_Solyc03g095650  ----------  ----------  ----------  ----------  ----------  1259
        SlMLO5_LEAF  ----------  ----------  ----------  ----------  ----------  1158
      SlMLO5_FLOWER  ----------  ----------  ----------  ----------  ----------  1158
       SlMLO5_FRUIT  ----------  ----------  ----------  ----------  ----------  1227
                                          6,620                                   6,640
gDNA_Solyc03g095650  AATAAAATAG  AGTATTAAAA  ATAACAGACA  CCAAAATTTG  TTTTAAGTTT  6650
cDNA_Solyc03g095650  ----------  ----------  ----------  ----------  ----------  1259
        SlMLO5_LEAF  ----------  ----------  ----------  ----------  ----------  1158
      SlMLO5_FLOWER  ----------  ----------  ----------  ----------  ----------  1158
       SlMLO5_FRUIT  ----------  ----------  ----------  ----------  ----------  1227
                              6,660                                   6,680                  6,700
gDNA_Solyc03g095650  AGAATATTAC  AATTTAAATA  CAAACTATGT  TTATAAAATA  TGTACTATAT  6700
cDNA_Solyc03g095650  ----------  ----------  ----------  ----------  ----------  1259
        SlMLO5_LEAF  ----------  ----------  ----------  ----------  ----------  1158
      SlMLO5_FLOWER  ----------  ----------  ----------  ----------  ----------  1158
       SlMLO5_FRUIT  ----------  ----------  ----------  ----------  ----------  1227
                                          6,720                                   6,740
gDNA_Solyc03g095650  ATAATAAAGT  TCCTAAAATT  TCTAGGGAGT  CACTGCATAG  GTCTATTTTT  6750
cDNA_Solyc03g095650  ----------  ----------  ----------  ----------  ----------  1259
        SlMLO5_LEAF  ----------  ----------  ----------  ----------  ----------  1158
      SlMLO5_FLOWER  ----------  ----------  ----------  ----------  ----------  1158
       SlMLO5_FRUIT  ----------  ----------  ----------  ----------  ----------  1227
                              6,760                                   6,780                  6,800
gDNA_Solyc03g095650  TTGTGTTTTA  TTATGATCAT  TGAAAAACAA  TTAGATTAAT  ATTTCTATTT  6800
cDNA_Solyc03g095650  ----------  ----------  ----------  ----------  ----------  1259
        SlMLO5_LEAF  ----------  ----------  ----------  ----------  ----------  1158
      SlMLO5_FLOWER  ----------  ----------  ----------  ----------  ----------  1158
       SlMLO5_FRUIT  ----------  ----------  ----------  ----------  ----------  1227
                                          6,820                                   6,840
gDNA_Solyc03g095650  AGCTATTTAT  TTTTTTACTT  GAAATCAAAC  TTAATAAATA  TTATATAGAT  6850
cDNA_Solyc03g095650  ----------  ----------  ----------  ----------  ----------  1259
        SlMLO5_LEAF  ----------  ----------  ----------  ----------  ----------  1158
      SlMLO5_FLOWER  ----------  ----------  ----------  ----------  ----------  1158
       SlMLO5_FRUIT  ----------  ----------  ----------  ----------  ----------  1227
                              6,860                                   6,880                  6,900
gDNA_Solyc03g095650  AATTTTACTT  GTGTAGTTGA  TTAACAAGAT  AATAACACTA  GTCAATATTT  6900
cDNA_Solyc03g095650  ----------  ----------  ----------  ----------  ----------  1259
        SlMLO5_LEAF  ----------  ----------  ----------  ----------  ----------  1158
      SlMLO5_FLOWER  ----------  ----------  ----------  ----------  ----------  1158
       SlMLO5_FRUIT  ----------  ----------  ----------  ----------  ----------  1227
                                          6,920                                   6,940
gDNA_Solyc03g095650  ATGAAACTCT  CTCCGTTTAA  AAAAGAATGA  CCTCTTTTCC  TTTTTAGTCT  6950
cDNA_Solyc03g095650  ----------  ----------  ----------  ----------  ----------  1259
        SlMLO5_LEAF  ----------  ----------  ----------  ----------  ----------  1158
      SlMLO5_FLOWER  ----------  ----------  ----------  ----------  ----------  1158
       SlMLO5_FRUIT  ----------  ----------  ----------  ----------  ----------  1227
                              6,960                                   6,980                  7,000
gDNA_Solyc03g095650  GTTCGGAAAA  AGATGACCTC  TTTCTTTTTT  CGGTAACAAC  TTTTCACGTG  7000
cDNA_Solyc03g095650  ----------  ----------  ----------  ----------  ----------  1259
        SlMLO5_LEAF  ----------  ----------  ----------  ----------  ----------  1158
      SlMLO5_FLOWER  ----------  ----------  ----------  ----------  ----------  1158
       SlMLO5_FRUIT  ----------  ----------  ----------  ----------  ----------  1227
```

```
                                            7,020                          7,040
gDNA_Solyc03g095650  GCATGTTTAA GACTACTAGA ATAAAAGACA ATTTTGTACA TTTGACATAA 7050
cDNA_Solyc03g095650  ---------- ---------- ---------- ---------- ---------- 1259
        SlMLO5_LEAF  ---------- ---------- ---------- ---------- ---------- 1158
      SlMLO5_FLOWER  ---------- ---------- ---------- ---------- ---------- 1158
       SlMLO5_FRUIT  ---------- ---------- ---------- ---------- ---------- 1227
                             7,060                          7,080                7,100
gDNA_Solyc03g095650  ATTCAATTTA GGATCACATG ATTAAAAGTC TTTTTTATTT ACTTGAACTT 7100
cDNA_Solyc03g095650  ---------- ---------- ---------- ---------- ---------- 1259
        SlMLO5_LEAF  ---------- ---------- ---------- ---------- ---------- 1158
      SlMLO5_FLOWER  ---------- ---------- ---------- ---------- ---------- 1158
       SlMLO5_FRUIT  ---------- ---------- ---------- ---------- ---------- 1227
                                            7,120                          7,140
gDNA_Solyc03g095650  CGTGTCAAGT CGAACTAGAC CATTCTTTGA GAAATAGAGA GAGTAATATT 7150
cDNA_Solyc03g095650  ---------- ---------- ---------- ---------- ---------- 1259
        SlMLO5_LEAF  ---------- ---------- ---------- ---------- ---------- 1158
      SlMLO5_FLOWER  ---------- ---------- ---------- ---------- ---------- 1158
       SlMLO5_FRUIT  ---------- ---------- ---------- ---------- ---------- 1227
                             7,160                          7,180                7,200
gDNA_Solyc03g095650  TTTAAATTAT AATTTAATAA AAAATTTATA AAAATATACT TTTAAGAAAA 7200
cDNA_Solyc03g095650  ---------- ---------- ---------- ---------- ---------- 1259
        SlMLO5_LEAF  ---------- ---------- ---------- ---------- ---------- 1158
      SlMLO5_FLOWER  ---------- ---------- ---------- ---------- ---------- 1158
       SlMLO5_FRUIT  ---------- ---------- ---------- ---------- ---------- 1227
                                            7,220                          7,240
gDNA_Solyc03g095650  GAGGGCTGAA AGTCCATAGC CCATACACTT GTGGGCTGAG CCGATTATTT 7250
cDNA_Solyc03g095650  ---------- ---------- ---------- ---------- ---------- 1259
        SlMLO5_LEAF  ---------- ---------- ---------- ---------- ---------- 1158
      SlMLO5_FLOWER  ---------- ---------- ---------- ---------- ---------- 1158
       SlMLO5_FRUIT  ---------- ---------- ---------- ---------- ---------- 1227
                             7,260                          7,280                7,300
gDNA_Solyc03g095650  TCTAACCCAC ATAAAAATGG GCTAACCCAG CCTAATCCAT CAAATTGCAA 7300
cDNA_Solyc03g095650  ---------- ---------- ---------- ---------- ---------- 1259
        SlMLO5_LEAF  ---------- ---------- ---------- ---------- ---------- 1158
      SlMLO5_FLOWER  ---------- ---------- ---------- ---------- ---------- 1158
       SlMLO5_FRUIT  ---------- ---------- ---------- ---------- ---------- 1227
                                            7,320                          7,340
gDNA_Solyc03g095650  AGCCCGAACG AAAATGAATC AACCCATATT GACAGCTCTA GCTAAAACCC 7350
cDNA_Solyc03g095650  ---------- ---------- ---------- ---------- ---------- 1259
        SlMLO5_LEAF  ---------- ---------- ---------- ---------- ---------- 1158
      SlMLO5_FLOWER  ---------- ---------- ---------- ---------- ---------- 1158
       SlMLO5_FRUIT  ---------- ---------- ---------- ---------- ---------- 1227
                             7,360                          7,380                7,400
gDNA_Solyc03g095650  TCTTTGTCTT ATATTTCCTC TATTTTTGTG GCCATTATAT AAAAAAGCAT 7400
cDNA_Solyc03g095650  ---------- ---------- ---------- ---------- ---------- 1259
        SlMLO5_LEAF  ---------- ---------- ---------- ---------- ---------- 1158
      SlMLO5_FLOWER  ---------- ---------- ---------- ---------- ---------- 1158
       SlMLO5_FRUIT  ---------- ---------- ---------- ---------- ---------- 1227
                                            7,420                          7,440
gDNA_Solyc03g095650  ATTTAACTTA TTGTTGGATT TTGATGTTGC TATAATTAGG GCTCTCATTC 7450
cDNA_Solyc03g095650  ---------- ---------- ---------- ---------- GCTCTCATTC 1269
        SlMLO5_LEAF  ---------- ---------- ---------- ---------- GCTCTCATTC 1168
      SlMLO5_FLOWER  ---------- ---------- ---------- ---------- GCTCTCATTC 1168
       SlMLO5_FRUIT  ---------- ---------- ---------- ---------- GCTCTCATTC 1237
                             7,460                          7,480                7,500
gDNA_Solyc03g095650  AAGTTCTTTG CAGCTACGTG ACACTCCCCC TATATGCCTT AGTTACTCAG 7500
cDNA_Solyc03g095650  AAGTTCTTTG CAGCTACGTG ACACTCCCCC TATATGCCTT AGTTACTCAG 1319
        SlMLO5_LEAF  AAGTTCTTTG CAGCTACGTG ACACTCCCCC TATATGCCTT AGTTACTCAG 1218
      SlMLO5_FLOWER  AAGTTCTTTG CAGCTACGTG ACACTCCCCC TATATGCCTT AGTTACTCAG 1218
       SlMLO5_FRUIT  AAGTTCTTTG CAGCTACGTG ACACTCCCCC TATATGCCTT AGTTACTCAG 1287
                                            7,520                          7,540
gDNA_Solyc03g095650  GTATATATTC TCTTCATCTG TCCTCGATCC ATGAATATAC AATTAGAAAT 7550
cDNA_Solyc03g095650  ---------- ---------- ---------- ---------- ---------- 1319
        SlMLO5_LEAF  ---------- ---------- ---------- ---------- ---------- 1218
      SlMLO5_FLOWER  ---------- ---------- ---------- ---------- ---------- 1218
       SlMLO5_FRUIT  ---------- ---------- ---------- ---------- ---------- 1287
                             7,560                          7,580                7,600
gDNA_Solyc03g095650  ATTTAAGTTA TACATACTTA CAAAAAAAAG TGAATATATT TAACTTGTTG 7600
cDNA_Solyc03g095650  ---------- ---------- ---------- ---------- ---------- 1319
        SlMLO5_LEAF  ---------- ---------- ---------- ---------- ---------- 1218
      SlMLO5_FLOWER  ---------- ---------- ---------- ---------- ---------- 1218
       SlMLO5_FRUIT  ---------- ---------- ---------- ---------- ---------- 1287
                                            7,620                          7,640
gDNA_Solyc03g095650  TAAAATTTTG CAGATGGGAT CAACAATGAA ACCAGTTATC TTTGGTGACA 7650
cDNA_Solyc03g095650  ---------- ---ATGGGAT CAACAATGAA ACCAGTTATC TTTGGTGACA 1356
        SlMLO5_LEAF  ---------- ---ATGGGAT CAACAATGAA ACCAGTTATC TTTGGTGACA 1255
      SlMLO5_FLOWER  ---------- ---ATGGGAT CAACAATGAA ACCAGTTATC TTTGGTGACA 1255
       SlMLO5_FRUIT  ---------- ---ATGGGAT CAACAATGAA ACCAGTTATC TTTGGTGACA 1324
                             7,660                          7,680                7,700
gDNA_Solyc03g095650  ACGTGGCATA CGCGATACGG ACATGGCATC AGACAGCAAA GCAACGGGCA 7700
cDNA_Solyc03g095650  ACGTGGCATA CGCGATACGG ACATGGCATC AGACAGCAAA GCAACGGGCA 1406
        SlMLO5_LEAF  ACGTGGCATA CGCGATACGG ACATGGCATC AGACAGCAAA GCAACGGGCA 1305
      SlMLO5_FLOWER  ACGTGGCATA CGCGATACGG ACATGGCATC AGACAGCAAA GCAACGGGCA 1305
       SlMLO5_FRUIT  ACGTGGCATA CGCGATACGG ACATGGCATC AGACAGCAAA GCAACGGGCA 1374
```

```
                                    7,720                           7,740
                                      |                               |
gDNA_Solyc03g095650  AAAGACGGGC GTCCGTCGAA AAATGCGAGT CCAGTGAGAA GCAGGGCAGT 7750
cDNA_Solyc03g095650  AAAGACGGGC GTCCGTCGAA AAATGCGAGT CCAGTGAGAA GCAGGGCAGT 1456
        SlMLO5_LEAF  AAAGACGGGC GTCCGTCGAA AAATGCGAGT CCAGTGAGAA GCAGGGCAGT 1355
      SlMLO5_FLOWER  AAAGACGGGC GTCCGTCGAA AAATGCGAGT CCAGTGAGAA GCAGGGCAGT 1355
       SlMLO5_FRUIT  AAAGACGGGC GTCCGTCGAA AAATGCGAGT CCAGTGAGAA GCAGGGCAGT 1424
                          7,760                           7,780                 7,800
                            |                               |                     |
gDNA_Solyc03g095650  GTCACCATTG CGTGGAGGTT CCTCTCCGGT TCAACAAAAA CACGGGCAAT 7800
cDNA_Solyc03g095650  GTCACCATTG CGTGGAGGTT CCTCTCCGGT TCAACAAAAA CACGGGCAAT 1506
        SlMLO5_LEAF  GTCACCATTG CGTGGAGGTT CCTCTCCGGT TCAACAAAAA CACGGGCAAT 1405
      SlMLO5_FLOWER  GTCACCATTG CGTGGAGGTT CCTCTCCGGT TCAACAAAAA CACGGGCAAT 1405
       SlMLO5_FRUIT  GTCACCATTG CGTGGAGGTT CCTCTCCGGT TCAACAAAAA CACGGGCAAT 1474
                                    7,820                           7,840
                                      |                               |
gDNA_Solyc03g095650  TATATCCTCC ATCACCTAAT CCTTCGCGTA GGAGGAGTGG AGGTAATCCA 7850
cDNA_Solyc03g095650  TATATCCTCC ATCACCTAAT CCTTCGCGTA GGAGGAGTGG AGGTAATCCA 1556
        SlMLO5_LEAF  TATATCCTCC ATCACCTAAT CCTTCGCGTA GGAGGAGTGG AGGTAATCCA 1455
      SlMLO5_FLOWER  TATATCCTCC ATCACCTAAT CCTTCGCGTA GGAGGAGTGG AGGTAATCCA 1455
       SlMLO5_FRUIT  TATATCCTCC ATCACCTAAT CCTTCGCGTA GGAGGAGTGG AGGTAATCCA 1524
                          7,860                           7,880                 7,900
                            |                               |                     |
gDNA_Solyc03g095650  GAATCGAGTT CTAGGCAAAT CTTTGATGAT GGAAGTCATG AGCAATCTGA 7900
cDNA_Solyc03g095650  GAATCGAGTT CTAGGCAAAT CTTTGATGAT GGAAGTCATG AGCAATCTGA 1606
        SlMLO5_LEAF  GAATCGAGTT CTAGGCAAAT CTTTGATGAT GGAAGTCATG AGCAATCTGA 1505
      SlMLO5_FLOWER  GAATCGAGTT CTAGGCAAAT CTTTGATGAT GGAAGTCATG AGCAATCTGA 1505
       SlMLO5_FRUIT  GAATCGAGTT CTAGGCAAAT CTTTGATGAT GGAAGTCATG AGCAATCTGA 1574
                                    7,920                           7,940
                                      |                               |
gDNA_Solyc03g095650  AATTGAAATT ACCTTGAATG ATTTATCACT TGAAAACAAA TTAAGTTGAA 7950
cDNA_Solyc03g095650  AATTGAAATT ACCTTGAATG ATTTATCACT TGAAAACAAA TTAAGTTGAA 1656
        SlMLO5_LEAF  AATTGAAATT ACCTTGAATG ATTTATCACT TGAAAACAAA TTAAGTTGA- 1554
      SlMLO5_FLOWER  AATTGAAATT ACCTTGAATG ATTTATCACT TGAAAACAAA TTAAGTTGA- 1554
       SlMLO5_FRUIT  AATTGAAATT ACCTTGAATG ATTTATCACT TGAAAACAAA TTAAGTTGA- 1623
                          7,960                           7,980                 8,000
                            |                               |                     |
gDNA_Solyc03g095650  CTTGTTAGGG TCTGATCCAT GTACGTATAT AGTTAAGTAG TGCATCTTGT 8000
cDNA_Solyc03g095650  CTTGTTAGGG TCTGATCCAT GTACGTATAT AGTTAAGTAG TGCATCTTGT 1706
        SlMLO5_LEAF  ---------- ---------- ---------- ---------- ---------- 1554
      SlMLO5_FLOWER  ---------- ---------- ---------- ---------- ---------- 1554
       SlMLO5_FRUIT  ---------- ---------- ---------- ---------- ---------- 1623
                                    8,020                           8,040
                                      |                               |
gDNA_Solyc03g095650  ACGTGTTTAC CTTTGAAATC TTTTTGTATA ATTGTATGTG GGAACTGAGC 8050
cDNA_Solyc03g095650  ACGTGTTTAC CTTTGAAATC TTTTTGTATA ATTGTATGTG GGAACTGAGC 1756
        SlMLO5_LEAF  ---------- ---------- ---------- ---------- ---------- 1554
      SlMLO5_FLOWER  ---------- ---------- ---------- ---------- ---------- 1554
       SlMLO5_FRUIT  ---------- ---------- ---------- ---------- ---------- 1623
                          8,060                           8,080                 8,100
                            |                               |                     |
gDNA_Solyc03g095650  TATGAAGCGC CAAGTGTGTG AATGTATTTT TGGTACATTT ATGGACTTGC 8100
cDNA_Solyc03g095650  TATGAAGCGC CAAGTGTGTG AATGTATTTT TGGTACATTT ATGGACTTGC 1806
        SlMLO5_LEAF  ---------- ---------- ---------- ---------- ---------- 1554
      SlMLO5_FLOWER  ---------- ---------- ---------- ---------- ---------- 1554
       SlMLO5_FRUIT  ---------- ---------- ---------- ---------- ---------- 1623
                                    8,120                           8,140
                                      |                               |
gDNA_Solyc03g095650  AAGTTGCACA TATATTATGT TGGTTCTTAT GTTAAAAGTG ATCTA  》8145
cDNA_Solyc03g095650  AAGTTGCACA TATATTATGT TGGTTCTTAT GTTAAAAGTG ATCTA》1851
        SlMLO5_LEAF  ---------- ---------- ---------- ---------- -----》1554
      SlMLO5_FLOWER  ---------- ---------- ---------- ---------- -----  1554
       SlMLO5_FRUIT  ---------- ---------- ---------- ---------- -----  1623
```

```
                                       20                            40
gDNA_Solyc02g082430  GTGAATTGTT CCCTCAACGA CGTGAGAAAT TATACCAACT TGTTGTCTAT 50
cDNA_Solyc02g082430  GTGAATTGTT CCCTCAACGA CGTGAGAAAT TATACCAACT TGTTGTCTAT 50
        SlMLO6_LEAF  ---------- ---------- ---------- ---------- ---------- -
        SlMLO6_ROOT  ---------- ---------- ---------- ---------- ---------- -
      SlMLO6_FLOWER  ---------- ---------- ---------- ---------- ---------- -
       SlMLO6_FRUIT  ---------- ---------- ---------- ---------- ---------- -

                             60                            80                 100
gDNA_Solyc02g082430  ACATATCTGT GAAATTATAC CTACCAAGTC TGGTCCTAAC TACTGCCAAT 100
cDNA_Solyc02g082430  ACATATCTGT GAAATTATAC CTACCAAGTC TGGTCCTAAC TACTGCCAAT 100
        SlMLO6_LEAF  ---------- ---------- ---------- ---------- ---------- -
        SlMLO6_ROOT  ---------- ---------- ---------- ---------- ---------- -
      SlMLO6_FLOWER  ---------- ---------- ---------- ---------- ---------- -
       SlMLO6_FRUIT  ---------- ---------- ---------- ---------- ---------- -

                                      120                           140
gDNA_Solyc02g082430  TCTATAAAAT TCCCTTTCAC CACTATAATT ATTTATGTAC TACTTATTAC 150
cDNA_Solyc02g082430  TCTATAAAAT TCCCTTTCAC CACTATAATT ATTTATGTAC TACTTATTAC 150
        SlMLO6_LEAF  ---------- ---------- ---------- ---------- ---------- -
        SlMLO6_ROOT  ---------- ---------- ---------- ---------- ---------- -
      SlMLO6_FLOWER  ---------- ---------- ---------- ---------- ---------- -
       SlMLO6_FRUIT  ---------- ---------- ---------- ---------- ---------- -

                            160                           180                 200
gDNA_Solyc02g082430  AACTCTGTTC TAGTTGAAAA TGTATGTCTT TTTGGCATTT TCTTGATTCT 200
cDNA_Solyc02g082430  AACTCTGTTC TAGTTGAAAA TGTATGTCTT TTTGGCATTT TCTTGATTCT 200
        SlMLO6_LEAF  ---------- ---------- ---------- ---------- ---------- -
        SlMLO6_ROOT  ---------- ---------- ---------- ---------- ---------- -
      SlMLO6_FLOWER  ---------- ---------- ---------- ---------- ---------- -
       SlMLO6_FRUIT  ---------- ---------- ---------- ---------- ---------- -

                                      220                           240
gDNA_Solyc02g082430  TGAGAGTCTT TTGAGGGTTT TAACAAGAAC CCATTTAGTT TTCTTGAAAT 250
cDNA_Solyc02g082430  TGAGAGTCTT TTGAGGGTTT TAACAAGAAC CCATTTAGTT TTCTTGAAAT 250
        SlMLO6_LEAF  ---------- ---------- ---------- ---------- ---------- -
        SlMLO6_ROOT  ---------- ---------- ---------- ---------- ---------- -
      SlMLO6_FLOWER  ---------- ---------- ---------- ---------- ---------- -
       SlMLO6_FRUIT  ---------- ---------- ---------- ---------- ---------- -

                            260                           280                 300
gDNA_Solyc02g082430  TCTTGATACA TCTGAAGCTG AAATACTGTG TTTCAAGATA AAGTTTGAAA 300
cDNA_Solyc02g082430  TCTTGATACA TCTGAAGCTG AAATACTGTG TTTCAAGATA AAGTTTGAAA 300
        SlMLO6_LEAF  ---------- ---------- ---------- ---------- ---------- -
        SlMLO6_ROOT  ---------- ---------- ---------- ---------- ---------- -
      SlMLO6_FLOWER  ---------- ---------- ---------- ---------- ---------- -
       SlMLO6_FRUIT  ---------- ---------- ---------- ---------- ---------- -

                                      320                           340
gDNA_Solyc02g082430  TTTTGTTGGG TTGAGGGTTA GTAATGGCAG GTGGGGGTGA TGGTACATCA 350
cDNA_Solyc02g082430  TTTTGTTGGG TTGAGGGTTA GTAATGGCAG GTGGGGGTGA TGGTACATCA 350
        SlMLO6_LEAF  ---------- ---------- ---ATGGCAG GTGGGGGTGA TGGTACATCA 27
        SlMLO6_ROOT  ---------- ---------- ---ATGGCAG GTGGGGGTGA TGGTACATCA 27
      SlMLO6_FLOWER  ---------- ---------- ---ATGGCAG GTGGGGGTGA TGGTACATCA 27
       SlMLO6_FRUIT  ---------- ---------- ---ATGGCAG GTGGGGGTGA TGGTACATCA 27

                            360                           380                 400
gDNA_Solyc02g082430  AGGCAACTTG ATCAAACACC AACTTGGGCT GTGGCTGGTG TTTGTGCAGT 400
cDNA_Solyc02g082430  AGGCAACTTG ATCAAACACC AACTTGGGCT GTGGCTGGTG TTTGTGCAGT 400
        SlMLO6_LEAF  AGGCAACTTG ATCAAACACC AACTTGGGCT GTGGCTGGTG TTTGTGCAGT 77
        SlMLO6_ROOT  AGGCAACTTG ATCAAACACC AACTTGGGCT GTGGCTGGTG TTTGTGCAGT 77
      SlMLO6_FLOWER  AGGCAACTTG ATCAAACACC AACTTGGGCT GTGGCTGGTG TTTGTGCAGT 77
       SlMLO6_FRUIT  AGGCAACTTG ATCAAACACC AACTTGGGCT GTGGCTGGTG TTTGTGCAGT 77

                                      420                           440
gDNA_Solyc02g082430  TATTATCCTC ATTTCTATTG CCTTAGAGAA GATATTGCAC AAACTTGGAA 450
cDNA_Solyc02g082430  TATTATCCTC ATTTCTATTG CCTTAGAGAA GATATTGCAC AAACTTGGAA 450
        SlMLO6_LEAF  TATTATCCTC ATTTCTATTG CCTTAGAGAA GATATTGCAC AAACTTGGAA 127
        SlMLO6_ROOT  TATTATCCTC ATTTCTATTG CCTTAGAGAA GATATTGCAC AAACTTGGAA 127
      SlMLO6_FLOWER  TATTATCCTC ATTTCTATTG CCTTAGAGAA GATATTGCAC AAACTTGGAA 127
       SlMLO6_FRUIT  TATTATCCTC ATTTCTATTG CCTTAGAGAA GATATTGCAC AAACTTGGAA 127

                            460                           480                 500
gDNA_Solyc02g082430  CGGTATGATG ACATAAAAAC TTAGTCTTTC TTTGTTTCTT TAATGACCCT 500
cDNA_Solyc02g082430  CG-------- ---------- ---------- ---------- ---------- 452
        SlMLO6_LEAF  CG-------- ---------- ---------- ---------- ---------- 129
        SlMLO6_ROOT  CG-------- ---------- ---------- ---------- ---------- 129
      SlMLO6_FLOWER  CG-------- ---------- ---------- ---------- ---------- 129
       SlMLO6_FRUIT  CG-------- ---------- ---------- ---------- ---------- 129

                                      520                           540
gDNA_Solyc02g082430  GTTCGTTGAC CTTCTTGTTT TGCGTTTAGG AAGAAGAATT CTTGAATTTT 550
cDNA_Solyc02g082430  ---------- ---------- ---------- ---------- ---------- 452
        SlMLO6_LEAF  ---------- ---------- ---------- ---------- ---------- 129
        SlMLO6_ROOT  ---------- ---------- ---------- ---------- ---------- 129
      SlMLO6_FLOWER  ---------- ---------- ---------- ---------- ---------- 129
       SlMLO6_FRUIT  ---------- ---------- ---------- ---------- ---------- 129

                            560                           580                 600
gDNA_Solyc02g082430  GTTGTTGATG TTTCTTGAAA ATATCCTGTT TTTTGTGAAA AAATGTTTGT 600
cDNA_Solyc02g082430  ---------- ---------- ---------- ---------- ---------- 452
        SlMLO6_LEAF  ---------- ---------- ---------- ---------- ---------- 129
        SlMLO6_ROOT  ---------- ---------- ---------- ---------- ---------- 129
      SlMLO6_FLOWER  ---------- ---------- ---------- ---------- ---------- 129
       SlMLO6_FRUIT  ---------- ---------- ---------- ---------- ---------- 129
```

```
                                                      620                           640
gDNA_Solyc02g082430  CTTTATTTAA GTTAATTTTC ACCCTTAATT GTTCTTGTAC TTTATCAACA  650
cDNA_Solyc02g082430  ---------- ---------- ---------- ---------- ----------  452
        SlMLO6_LEAF  ---------- ---------- ---------- ---------- ----------  129
        SlMLO6_ROOT  ---------- ---------- ---------- ---------- ----------  129
      SlMLO6_FLOWER  ---------- ---------- ---------- ---------- ----------  129
       SlMLO6_FRUIT  ---------- ---------- ---------- ---------- ----------  129
                                660                           680                 700
gDNA_Solyc02g082430  AGTCTTAGCC AAGAGCATTT ATTAAAGTGG GACCTGTAGT AGCTCTCTTT  700
cDNA_Solyc02g082430  ---------- ---------- ---------- ---------- ----------  452
        SlMLO6_LEAF  ---------- ---------- ---------- ---------- ----------  129
        SlMLO6_ROOT  ---------- ---------- ---------- ---------- ----------  129
      SlMLO6_FLOWER  ---------- ---------- ---------- ---------- ----------  129
       SlMLO6_FRUIT  ---------- ---------- ---------- ---------- ----------  129
                                           720                           740
gDNA_Solyc02g082430  TGTGTTTCCT AGTTTTGGAA ATCAAGGGTT TTAAGTTGCA GCTATGTTGA  750
cDNA_Solyc02g082430  ---------- ---------- ---------- ---------- ----------  452
        SlMLO6_LEAF  ---------- ---------- ---------- ---------- ----------  129
        SlMLO6_ROOT  ---------- ---------- ---------- ---------- ----------  129
      SlMLO6_FLOWER  ---------- ---------- ---------- ---------- ----------  129
       SlMLO6_FRUIT  ---------- ---------- ---------- ---------- ----------  129
                                760                           780                 800
gDNA_Solyc02g082430  TAATTGACTT CTTATTTTGT AACATCATTT GACTGTGGAA GTCTTGCCTT  800
cDNA_Solyc02g082430  ---------- ---------- ---------- ---------- ----------  452
        SlMLO6_LEAF  ---------- ---------- ---------- ---------- ----------  129
        SlMLO6_ROOT  ---------- ---------- ---------- ---------- ----------  129
      SlMLO6_FLOWER  ---------- ---------- ---------- ---------- ----------  129
       SlMLO6_FRUIT  ---------- ---------- ---------- ---------- ----------  129
                                           820                           840
gDNA_Solyc02g082430  GCCAAATTGG AAGTGATATC ATGTCAACTA TAAACTCTAG AACCTACAAA  850
cDNA_Solyc02g082430  ---------- ---------- ---------- ---------- ----------  452
        SlMLO6_LEAF  ---------- ---------- ---------- ---------- ----------  129
        SlMLO6_ROOT  ---------- ---------- ---------- ---------- ----------  129
      SlMLO6_FLOWER  ---------- ---------- ---------- ---------- ----------  129
       SlMLO6_FRUIT  ---------- ---------- ---------- ---------- ----------  129
                                860                           880                 900
gDNA_Solyc02g082430  ATGTTCTTGA TTGCTCAGAT GCTTGTTGCG TCCTCTATAT AATGATGGTG  900
cDNA_Solyc02g082430  ---------- ---------- ---------- ---------- ----------  452
        SlMLO6_LEAF  ---------- ---------- ---------- ---------- ----------  129
        SlMLO6_ROOT  ---------- ---------- ---------- ---------- ----------  129
      SlMLO6_FLOWER  ---------- ---------- ---------- ---------- ----------  129
       SlMLO6_FRUIT  ---------- ---------- ---------- ---------- ----------  129
                                           920                           940
gDNA_Solyc02g082430  ACCGAGCCTG CTTGCACGCA TCTTGATTAT TACTCTGTGT CAGCTATAAG  950
cDNA_Solyc02g082430  ---------- ---------- ---------- ---------- ----------  452
        SlMLO6_LEAF  ---------- ---------- ---------- ---------- ----------  129
        SlMLO6_ROOT  ---------- ---------- ---------- ---------- ----------  129
      SlMLO6_FLOWER  ---------- ---------- ---------- ---------- ----------  129
       SlMLO6_FRUIT  ---------- ---------- ---------- ---------- ----------  129
                                960                           980               1,000
gDNA_Solyc02g082430  GAAAATGCAC AATGGAAAAA ACAAAGTGCT ATCTGTGGGA GTATATGTAT  1000
cDNA_Solyc02g082430  ---------- ---------- ---------- ---------- ----------  452
        SlMLO6_LEAF  ---------- ---------- ---------- ---------- ----------  129
        SlMLO6_ROOT  ---------- ---------- ---------- ---------- ----------  129
      SlMLO6_FLOWER  ---------- ---------- ---------- ---------- ----------  129
       SlMLO6_FRUIT  ---------- ---------- ---------- ---------- ----------  129
                                         1,020                         1,040
gDNA_Solyc02g082430  ATGAGTTCTC GGTAATCATA ATTCATAGTT TTACTATGGC TATTCAAGTT  1050
cDNA_Solyc02g082430  ---------- ---------- ---------- ---------- ----------  452
        SlMLO6_LEAF  ---------- ---------- ---------- ---------- ----------  129
        SlMLO6_ROOT  ---------- ---------- ---------- ---------- ----------  129
      SlMLO6_FLOWER  ---------- ---------- ---------- ---------- ----------  129
       SlMLO6_FRUIT  ---------- ---------- ---------- ---------- ----------  129
                              1,060                         1,080               1,100
gDNA_Solyc02g082430  GAGCTCGTGT TTATTTACAG TGATCCCTAA TTTGTCATGG AAGTTACATG  1100
cDNA_Solyc02g082430  ---------- ---------- ---------- ---------- ----------  452
        SlMLO6_LEAF  ---------- ---------- ---------- ---------- ----------  129
        SlMLO6_ROOT  ---------- ---------- ---------- ---------- ----------  129
      SlMLO6_FLOWER  ---------- ---------- ---------- ---------- ----------  129
       SlMLO6_FRUIT  ---------- ---------- ---------- ---------- ----------  129
                                         1,120                         1,140
gDNA_Solyc02g082430  ATTGAACAAA GGAGTGAGTG GATTCAAATG AAGCGGCA TT GGTATTGAGG  1150
cDNA_Solyc02g082430  ---------- ---------- ---------- ---------- ----------  452
        SlMLO6_LEAF  ---------- ---------- ---------- ---------- ----------  129
        SlMLO6_ROOT  ---------- ---------- ---------- ---------- ----------  129
      SlMLO6_FLOWER  ---------- ---------- ---------- ---------- ----------  129
       SlMLO6_FRUIT  ---------- ---------- ---------- ---------- ----------  129
                              1,160                         1,180               1,200
gDNA_Solyc02g082430  ATTCGTATAG CTAAACCAGT CTAGAGGGTC GGTAGTTACT GTTTCTGTTG  1200
cDNA_Solyc02g082430  ---------- ---------- ---------- ---------- ----------  452
        SlMLO6_LEAF  ---------- ---------- ---------- ---------- ----------  129
        SlMLO6_ROOT  ---------- ---------- ---------- ---------- ----------  129
      SlMLO6_FLOWER  ---------- ---------- ---------- ---------- ----------  129
       SlMLO6_FRUIT  ---------- ---------- ---------- ---------- ----------  129
```

```
                                            1,220                          1,240
gDNA_Solyc02g082430  TTAATCTGCT GCTGTAATAT TAGCTCTCAA ATCTTTTGCT TTTAAGAAAG  1250
cDNA_Solyc02g082430  ---------- ---------- ---------- ---------- ----------  452
        SlMLO6_LEAF  ---------- ---------- ---------- ---------- ----------  129
        SlMLO6_ROOT  ---------- ---------- ---------- ---------- ----------  129
      SlMLO6_FLOWER  ---------- ---------- ---------- ---------- ----------  129
       SlMLO6_FRUIT  ---------- ---------- ---------- ---------- ----------  129
                              1,260                          1,280                          1,300
gDNA_Solyc02g082430  CTGTAGTCTT TATATGTATG TATCCTGGAT GATTTGCTTT AGTACTATTG  1300
cDNA_Solyc02g082430  ---------- ---------- ---------- ---------- ----------  452
        SlMLO6_LEAF  ---------- ---------- ---------- ---------- ----------  129
        SlMLO6_ROOT  ---------- ---------- ---------- ---------- ----------  129
      SlMLO6_FLOWER  ---------- ---------- ---------- ---------- ----------  129
       SlMLO6_FRUIT  ---------- ---------- ---------- ---------- ----------  129
                                            1,320                          1,340
gDNA_Solyc02g082430  TTTCCTCTGT TGCATATCGA GAAACAAGGA ACTACGACTA CTGTTTTCTC  1350
cDNA_Solyc02g082430  ---------- ---------- ---------- ---------- ----------  452
        SlMLO6_LEAF  ---------- ---------- ---------- ---------- ----------  129
        SlMLO6_ROOT  ---------- ---------- ---------- ---------- ----------  129
      SlMLO6_FLOWER  ---------- ---------- ---------- ---------- ----------  129
       SlMLO6_FRUIT  ---------- ---------- ---------- ---------- ----------  129
                              1,360                          1,380                          1,400
gDNA_Solyc02g082430  ACTTGGTTGG TTTATTTGTG CACTTTTGAG TTGTTTTACA CATATTGGTT  1400
cDNA_Solyc02g082430  ---------- ---------- ---------- ---------- ----------  452
        SlMLO6_LEAF  ---------- ---------- ---------- ---------- ----------  129
        SlMLO6_ROOT  ---------- ---------- ---------- ---------- ----------  129
      SlMLO6_FLOWER  ---------- ---------- ---------- ---------- ----------  129
       SlMLO6_FRUIT  ---------- ---------- ---------- ---------- ----------  129
                                            1,420                          1,440
gDNA_Solyc02g082430  CTTTGGACAA CATAACTTCT GATTGTTGTA ATTGGTTCTT TCTGCATCTT  1450
cDNA_Solyc02g082430  ---------- ---------- ---------- ---------- ----------  452
        SlMLO6_LEAF  ---------- ---------- ---------- ---------- ----------  129
        SlMLO6_ROOT  ---------- ---------- ---------- ---------- ----------  129
      SlMLO6_FLOWER  ---------- ---------- ---------- ---------- ----------  129
       SlMLO6_FRUIT  ---------- ---------- ---------- ---------- ----------  129
                              1,460                          1,480                          1,500
gDNA_Solyc02g082430  ATTTAATTTT GACCAGTAAA CCAGGGATAA GAGTGAAAAA TAGTTCAATG  1500
cDNA_Solyc02g082430  ---------- ---------- ---------- ---------- ----------  452
        SlMLO6_LEAF  ---------- ---------- ---------- ---------- ----------  129
        SlMLO6_ROOT  ---------- ---------- ---------- ---------- ----------  129
      SlMLO6_FLOWER  ---------- ---------- ---------- ---------- ----------  129
       SlMLO6_FRUIT  ---------- ---------- ---------- ---------- ----------  129
                                            1,520                          1,540
gDNA_Solyc02g082430  TTTGTGTTGT TCCTTGTGAA CTTATTCCTT TGTGCTGCAG AAAATATCAA  1550
cDNA_Solyc02g082430  ---------- ---------- ---------- ---------- ----------  452
        SlMLO6_LEAF  ---------- ---------- ---------- ---------- ----------  129
        SlMLO6_ROOT  ---------- ---------- ---------- ---------- ----------  129
      SlMLO6_FLOWER  ---------- ---------- ---------- ---------- ----------  129
       SlMLO6_FRUIT  ---------- ---------- ---------- ---------- ----------  129
                              1,560                          1,580                          1,600
gDNA_Solyc02g082430  TAACATTGAG TTCGGATTCA TTTTTCGTCC TTTCATTTAA TTGTAATAAC  1600
cDNA_Solyc02g082430  ---------- ---------- ---------- ---------- ----------  452
        SlMLO6_LEAF  ---------- ---------- ---------- ---------- ----------  129
        SlMLO6_ROOT  ---------- ---------- ---------- ---------- ----------  129
      SlMLO6_FLOWER  ---------- ---------- ---------- ---------- ----------  129
       SlMLO6_FRUIT  ---------- ---------- ---------- ---------- ----------  129
                                            1,620                          1,640
gDNA_Solyc02g082430  TTGCTTCTTT TTATTTTCTT CATTTTTCCC CTTTTTGGCT TTTGGTCAGC  1650
cDNA_Solyc02g082430  ---------- ---------- ---------- ---------- ----------  452
        SlMLO6_LEAF  ---------- ---------- ---------- ---------- ----------  129
        SlMLO6_ROOT  ---------- ---------- ---------- ---------- ----------  129
      SlMLO6_FLOWER  ---------- ---------- ---------- ---------- ----------  129
       SlMLO6_FRUIT  ---------- ---------- ---------- ---------- ----------  129
                              1,660                          1,680                          1,700
gDNA_Solyc02g082430  ATTTTATAAC CAAGTTTGCT CCGCTATTGC ACTGATATTG AAGTTTGTTG  1700
cDNA_Solyc02g082430  ---------- ---------- ---------- ---------- ----------  452
        SlMLO6_LEAF  ---------- ---------- ---------- ---------- ----------  129
        SlMLO6_ROOT  ---------- ---------- ---------- ---------- ----------  129
      SlMLO6_FLOWER  ---------- ---------- ---------- ---------- ----------  129
       SlMLO6_FRUIT  ---------- ---------- ---------- ---------- ----------  129
                                            1,720                          1,740
gDNA_Solyc02g082430  TCTTAAAGTT TTTTACTTTT CAAAACTTAT TTTAATTGCA TGAGTTGATG  1750
cDNA_Solyc02g082430  ---------- ---------- ---------- ---------- ----------  452
        SlMLO6_LEAF  ---------- ---------- ---------- ---------- ----------  129
        SlMLO6_ROOT  ---------- ---------- ---------- ---------- ----------  129
      SlMLO6_FLOWER  ---------- ---------- ---------- ---------- ----------  129
       SlMLO6_FRUIT  ---------- ---------- ---------- ---------- ----------  129
                              1,760                          1,780                          1,800
gDNA_Solyc02g082430  TACTCAGCTC ATTTGTTGGT TCCTTGAATT ACTACTACTA TAGAAAATAT  1800
cDNA_Solyc02g082430  ---------- ---------- ---------- ---------- ----------  452
        SlMLO6_LEAF  ---------- ---------- ---------- ---------- ----------  129
        SlMLO6_ROOT  ---------- ---------- ---------- ---------- ----------  129
      SlMLO6_FLOWER  ---------- ---------- ---------- ---------- ----------  129
       SlMLO6_FRUIT  ---------- ---------- ---------- ---------- ----------  129
```

```
                                     1,820                         1,840
gDNA_Solyc02g082430  GAATAACTGA CACAATGAAA TCTTTGCTCA GTGGTTGACT GACAGGCATA 1850
cDNA_Solyc02g082430  ---------- ---------- ---------- -TGGTTGACT GACAGGCATA 471
        SIMLO6_LEAF  ---------- ---------- ---------- -TGGTTGACT GACAGGCATA 148
        SIMLO6_ROOT  ---------- ---------- ---------- -TGGTTGACT GACAGGCATA 148
      SIMLO6_FLOWER  ---------- ---------- ---------- -TGGTTGACT GACAGGCATA 148
       SIMLO6_FRUIT  ---------- ---------- ---------- -TGGTTGACT GACAGGCATA 148
                          1,860                         1,880                1,900
gDNA_Solyc02g082430  AAAAAGCTCT CTTTGAGGCC TTGGAGAAGG TTAAGGCCGG TAAGATTTTT 1900
cDNA_Solyc02g082430  AAAAAGCTCT CTTTGAGGCC TTGGAGAAGG TTAAGGCCG- ---------- 510
        SIMLO6_LEAF  AAAAAGCTCT CTTTGAGGCC TTGGAGAAGG TTAAGGCCG- ---------- 187
        SIMLO6_ROOT  AAAAAGCTCT CTTTGAGGCC TTGGAGAAGG TTAAGGCCG- ---------- 187
      SIMLO6_FLOWER  AAAAAGCTCT CTTTGAGGCC TTGGAGAAGG TTAAGGCCG- ---------- 187
       SIMLO6_FRUIT  AAAAAGCTCT CTTTGAGGCC TTGGAGAAGG TTAAGGCCG- ---------- 187
                                     1,920                         1,940
gDNA_Solyc02g082430  TCTTTCTCCT CTTGTTATCA TTTTTTTATT AGAGGTGACG TGTTTCCATT 1950
cDNA_Solyc02g082430  ---------- ---------- ---------- ---------- ---------- 510
        SIMLO6_LEAF  ---------- ---------- ---------- ---------- ---------- 187
        SIMLO6_ROOT  ---------- ---------- ---------- ---------- ---------- 187
      SIMLO6_FLOWER  ---------- ---------- ---------- ---------- ---------- 187
       SIMLO6_FRUIT  ---------- ---------- ---------- ---------- ---------- 187
                          1,960                         1,980                2,000
gDNA_Solyc02g082430  TTGATTGAAT CATCTAATTC TTGCAGAGTT GATGATTCTC GGTTTCATCT 2000
cDNA_Solyc02g082430  ---------- ---------- ------AGTT GATGATTCTC GGTTTCATCT 534
        SIMLO6_LEAF  ---------- ---------- ------AGTT GATGATTCTC GGTTTCATCT 211
        SIMLO6_ROOT  ---------- ---------- ------AGTT GATGATTCTC GGTTTCATCT 211
      SIMLO6_FLOWER  ---------- ---------- ------AGTT GATGATTCTC GGTTTCATCT 211
       SIMLO6_FRUIT  ---------- ---------- ------AGTT GATGATTCTC GGTTTCATCT 211
                                     2,020                         2,040
gDNA_Solyc02g082430  CGTTAACCCT TGTATTTAGT CAATATTACA TTGCTGGAAT TTGTATCCCC 2050
cDNA_Solyc02g082430  CGTTAACCCT TGTATTTAGT CAATATTACA TTGCTGGAAT TTGTATCCCC 584
        SIMLO6_LEAF  CGTTAACCCT TGTATTTAGT CAATATTACA TTGCTGGAAT TTGTATCCCC 261
        SIMLO6_ROOT  CGTTAACCCT TGTATTTAGT CAATATTACA TTGCTGGAAT TTGTATCCCC 261
      SIMLO6_FLOWER  CGTTAACCCT TGTATTTAGT CAATATTACA TTGCTGGAAT TTGTATCCCC 261
       SIMLO6_FRUIT  CGTTAACCCT TGTATTTAGT CAATATTACA TTGCTGGAAT TTGTATCCCC 261
                          2,060                         2,080                2,100
gDNA_Solyc02g082430  CCAAGTGTTG CTGATACAAT GTTGCCATGC CCTGCAAACA ACAAAGATGC 2100
cDNA_Solyc02g082430  CCAAGTGTTG CTGATACAAT GTTGCCATGC CCTGCAAACA ACAAAGATGC 634
        SIMLO6_LEAF  CCAAGTGTTG CTGATACAAT GTTGCCATGC CCTGCAAACA ACAAAGATGC 311
        SIMLO6_ROOT  CCAAGTGTTG CTGATACAAT GTTGCCATGC CCTGCAAACA ACAAAGATGC 311
      SIMLO6_FLOWER  CCAAGTGTTG CTGATACAAT GTTGCCATGC CCTGCAAACA ACAAAGATGC 311
       SIMLO6_FRUIT  CCAAGTGTTG CTGATACAAT GTTGCCATGC CCTGCAAACA ACAAAGATGC 311
                                     2,120                         2,140
gDNA_Solyc02g082430  AGCAAAGGAG GAGGAACACC GTAGGAAGCT TTTATGGTAT GAGCGTAGAA 2150
cDNA_Solyc02g082430  AGCAAAGGAG GAGGAACACC GTAGGAAGCT TTTATGGTAT GAGCGTAGAA 684
        SIMLO6_LEAF  AGCAAAGGAG GAGGAACACC GTAGGAAGCT TTTATGGTAT GAGCGTAGAA 361
        SIMLO6_ROOT  AGCAAAGGAG GAGGAACACC GTAGGAAGCT TTTATGGTAT GAGCGTAGAA 361
      SIMLO6_FLOWER  AGCAAAGGAG GAGGAACACC GTAGGAAGCT TTTATGGTAT GAGCGTAGAA 361
       SIMLO6_FRUIT  AGCAAAGGAG GAGGAACACC GTAGGAAGCT TTTATGGTAT GAGCGTAGAA 361
                          2,160                         2,180                2,200
gDNA_Solyc02g082430  TTTTGGCAGG TGCAGAGCCT AAATGCAAAG AGGTAACGTG TAGATGGCCC 2200
cDNA_Solyc02g082430  TTTTGGCAGG TGCAGAGCCT AAATGCAAAG ---------- ---------- 714
        SIMLO6_LEAF  TTTTGGCAGG TGCAGAGCCT AAATGCAAAG ---------- ---------- 391
        SIMLO6_ROOT  TTTTGGCAGG TGCAGAGCCT AAATGCAAAG ---------- ---------- 391
      SIMLO6_FLOWER  TTTTGGCAGG TGCAGAGCCT AAATGCAAAG ---------- ---------- 391
       SIMLO6_FRUIT  TTTTGGCAGG TGCAGAGCCT AAATGCAAAG ---------- ---------- 391
                                     2,220                         2,240
gDNA_Solyc02g082430  TTGACAATAT GGGTCAGACT TCACTGATGT ATTTAGTTGT ATTAATGAGT 2250
cDNA_Solyc02g082430  ---------- ---------- ---------- ---------- ---------- 714
        SIMLO6_LEAF  ---------- ---------- ---------- ---------- ---------- 391
        SIMLO6_ROOT  ---------- ---------- ---------- ---------- ---------- 391
      SIMLO6_FLOWER  ---------- ---------- ---------- ---------- ---------- 391
       SIMLO6_FRUIT  ---------- ---------- ---------- ---------- ---------- 391
                          2,260                         2,280                2,300
gDNA_Solyc02g082430  ACTGATAGCA AAAATGGCTC TGGGAGAGTT GTGCTTTTGG TAAATTTACT 2300
cDNA_Solyc02g082430  ---------- ---------- ---------- ---------- ---------- 714
        SIMLO6_LEAF  ---------- ---------- ---------- ---------- ---------- 391
        SIMLO6_ROOT  ---------- ---------- ---------- ---------- ---------- 391
      SIMLO6_FLOWER  ---------- ---------- ---------- ---------- ---------- 391
       SIMLO6_FRUIT  ---------- ---------- ---------- ---------- ---------- 391
                                     2,320                         2,340
gDNA_Solyc02g082430  AATCCTATCT TGAATTACAA ATGGAGACAT TACTAAGAGG GTGTTAGGAT 2350
cDNA_Solyc02g082430  ---------- ---------- ---------- ---------- ---------- 714
        SIMLO6_LEAF  ---------- ---------- ---------- ---------- ---------- 391
        SIMLO6_ROOT  ---------- ---------- ---------- ---------- ---------- 391
      SIMLO6_FLOWER  ---------- ---------- ---------- ---------- ---------- 391
       SIMLO6_FRUIT  ---------- ---------- ---------- ---------- ---------- 391
                          2,360                         2,380                2,400
gDNA_Solyc02g082430  TGGCTTAAAA GTTGGTCAAA CCTACTTTTA AGTTAGTTTT TGACTTATAG 2400
cDNA_Solyc02g082430  ---------- ---------- ---------- ---------- ---------- 714
        SIMLO6_LEAF  ---------- ---------- ---------- ---------- ---------- 391
        SIMLO6_ROOT  ---------- ---------- ---------- ---------- ---------- 391
      SIMLO6_FLOWER  ---------- ---------- ---------- ---------- ---------- 391
       SIMLO6_FRUIT  ---------- ---------- ---------- ---------- ---------- 391
```

```
                                             2,420                         2,440
gDNA_Solyc02g082430  AAGTGCCTGA AAAGCATAAA AAATAACTTA AAATAAGTCA AAAATGACTT  2450
cDNA_Solyc02g082430  ---------- ---------- ---------- ---------- ----------  714
        SlMLO6_LEAF  ---------- ---------- ---------- ---------- ----------  391
        SlMLO6_ROOT  ---------- ---------- ---------- ---------- ----------  391
      SlMLO6_FLOWER  ---------- ---------- ---------- ---------- ----------  391
       SlMLO6_FRUIT  ---------- ---------- ---------- ---------- ----------  391
                              2,460                         2,480                  2,500
gDNA_Solyc02g082430  AAGATTAGTT GAGAAGTGTT TGGCAATGTC AAGAATGACT TAAAATAAGT  2500
cDNA_Solyc02g082430  ---------- ---------- ---------- ---------- ----------  714
        SlMLO6_LEAF  ---------- ---------- ---------- ---------- ----------  391
        SlMLO6_ROOT  ---------- ---------- ---------- ---------- ----------  391
      SlMLO6_FLOWER  ---------- ---------- ---------- ---------- ----------  391
       SlMLO6_FRUIT  ---------- ---------- ---------- ---------- ----------  391
                                             2,520                         2,540
gDNA_Solyc02g082430  TTAAAATGAC TTAGAATAAG TCAAAAATCA AAAGTAGGTC TCCCCCTACT  2550
cDNA_Solyc02g082430  ---------- ---------- ---------- ---------- ----------  714
        SlMLO6_LEAF  ---------- ---------- ---------- ---------- ----------  391
        SlMLO6_ROOT  ---------- ---------- ---------- ---------- ----------  391
      SlMLO6_FLOWER  ---------- ---------- ---------- ---------- ----------  391
       SlMLO6_FRUIT  ---------- ---------- ---------- ---------- ----------  391
                              2,560                         2,580                  2,600
gDNA_Solyc02g082430  TTTTATTTTT TAACATAAAA TTTCAGTTTG ACTTTTTATT TTTGACTTAA  2600
cDNA_Solyc02g082430  ---------- ---------- ---------- ---------- ----------  714
        SlMLO6_LEAF  ---------- ---------- ---------- ---------- ----------  391
        SlMLO6_ROOT  ---------- ---------- ---------- ---------- ----------  391
      SlMLO6_FLOWER  ---------- ---------- ---------- ---------- ----------  391
       SlMLO6_FRUIT  ---------- ---------- ---------- ---------- ----------  391
                                             2,620                         2,640
gDNA_Solyc02g082430  AAGTTACTTT TTTTTTTAAG CCAATCCAAA CAGGCCCCAA ATGTTTTTTC  2650
cDNA_Solyc02g082430  ---------- ---------- ---------- ---------- ----------  714
        SlMLO6_LEAF  ---------- ---------- ---------- ---------- ----------  391
        SlMLO6_ROOT  ---------- ---------- ---------- ---------- ----------  391
      SlMLO6_FLOWER  ---------- ---------- ---------- ---------- ----------  391
       SlMLO6_FRUIT  ---------- ---------- ---------- ---------- ----------  391
                              2,660                         2,680                  2,700
gDNA_Solyc02g082430  GCTTCTATCA ATTGGCCTAG ACAATGAGAT ATTTTGAGCA AACCTACTAA  2700
cDNA_Solyc02g082430  ---------- ---------- ---------- ---------- ----------  714
        SlMLO6_LEAF  ---------- ---------- ---------- ---------- ----------  391
        SlMLO6_ROOT  ---------- ---------- ---------- ---------- ----------  391
      SlMLO6_FLOWER  ---------- ---------- ---------- ---------- ----------  391
       SlMLO6_FRUIT  ---------- ---------- ---------- ---------- ----------  391
                                             2,720                         2,740
gDNA_Solyc02g082430  AAATTATTGC AAACTGTCTT AGGATTTTCG ACTCTTCAAT TCAAATGAAT  2750
cDNA_Solyc02g082430  ---------- ---------- ---------- ---------- ----------  714
        SlMLO6_LEAF  ---------- ---------- ---------- ---------- ----------  391
        SlMLO6_ROOT  ---------- ---------- ---------- ---------- ----------  391
      SlMLO6_FLOWER  ---------- ---------- ---------- ---------- ----------  391
       SlMLO6_FRUIT  ---------- ---------- ---------- ---------- ----------  391
                              2,760                         2,780                  2,800
gDNA_Solyc02g082430  GGACATTTCA AATAACATGG TGTCATCACA AAAAAGGAAC TGCAAAAGAA  2800
cDNA_Solyc02g082430  ---------- ---------- ---------- ---------- ----------  714
        SlMLO6_LEAF  ---------- ---------- ---------- ---------- ----------  391
        SlMLO6_ROOT  ---------- ---------- ---------- ---------- ----------  391
      SlMLO6_FLOWER  ---------- ---------- ---------- ---------- ----------  391
       SlMLO6_FRUIT  ---------- ---------- ---------- ---------- ----------  391
                                             2,820                         2,840
gDNA_Solyc02g082430  ATTTTACACA AAGCACCTTC ATTCCATTAG GGTGTATGCC AAGTGATGGA  2850
cDNA_Solyc02g082430  ---------- ---------- ---------- ---------- ----------  714
        SlMLO6_LEAF  ---------- ---------- ---------- ---------- ----------  391
        SlMLO6_ROOT  ---------- ---------- ---------- ---------- ----------  391
      SlMLO6_FLOWER  ---------- ---------- ---------- ---------- ----------  391
       SlMLO6_FRUIT  ---------- ---------- ---------- ---------- ----------  391
                              2,860                         2,880                  2,900
gDNA_Solyc02g082430  CTCATGTAGT GTAGCCTAAT TGAGGATTAG GAAACACTTA GCTTTTTACC  2900
cDNA_Solyc02g082430  ---------- ---------- ---------- ---------- ----------  714
        SlMLO6_LEAF  ---------- ---------- ---------- ---------- ----------  391
        SlMLO6_ROOT  ---------- ---------- ---------- ---------- ----------  391
      SlMLO6_FLOWER  ---------- ---------- ---------- ---------- ----------  391
       SlMLO6_FRUIT  ---------- ---------- ---------- ---------- ----------  391
                                             2,920                         2,940
gDNA_Solyc02g082430  ATCACTGTTT TATTCTTACG CTATCCTGTC TGACATAGGA AGAAGGAATT  2950
cDNA_Solyc02g082430  ---------- ---------- ---------- ---------- ----------  714
        SlMLO6_LEAF  ---------- ---------- ---------- ---------- ----------  391
        SlMLO6_ROOT  ---------- ---------- ---------- ---------- ----------  391
      SlMLO6_FLOWER  ---------- ---------- ---------- ---------- ----------  391
       SlMLO6_FRUIT  ---------- ---------- ---------- ---------- ----------  391
                              2,960                         2,980                  3,000
gDNA_Solyc02g082430  GATGACTAAA AAAGCACCAT GTATGCGCTT CAACTGTCTT TAGACTATGA  3000
cDNA_Solyc02g082430  ---------- ---------- ---------- ---------- ----------  714
        SlMLO6_LEAF  ---------- ---------- ---------- ---------- ----------  391
        SlMLO6_ROOT  ---------- ---------- ---------- ---------- ----------  391
      SlMLO6_FLOWER  ---------- ---------- ---------- ---------- ----------  391
       SlMLO6_FRUIT  ---------- ---------- ---------- ---------- ----------  391
```

```
                                           3,020                          3,040
gDNA_Solyc02g082430  GCTTCAAAAA TTTGTGGTCG GCTTAGGTTT TGGGTAAATT TTTCATGTAA 3050
cDNA_Solyc02g082430  ---------- ---------- ---------- ---------- ---------- 714
        SlMLO6_LEAF  ---------- ---------- ---------- ---------- ---------- 391
        SlMLO6_ROOT  ---------- ---------- ---------- ---------- ---------- 391
      SlMLO6_FLOWER  ---------- ---------- ---------- ---------- ---------- 391
       SlMLO6_FRUIT  ---------- ---------- ---------- ---------- ---------- 391
                              3,060                          3,080                   3,100
gDNA_Solyc02g082430  AGTTAATATT TGAAGGTAAA GTTGTTCTAC CAGATGCAGC ATCCTTGCAT 3100
cDNA_Solyc02g082430  ---------- ---------- ---------- ---------- ---------- 714
        SlMLO6_LEAF  ---------- ---------- ---------- ---------- ---------- 391
        SlMLO6_ROOT  ---------- ---------- ---------- ---------- ---------- 391
      SlMLO6_FLOWER  ---------- ---------- ---------- ---------- ---------- 391
       SlMLO6_FRUIT  ---------- ---------- ---------- ---------- ---------- 391
                                           3,120                          3,140
gDNA_Solyc02g082430  TCCGTTAGCA CTGTGTTCTG GCCCATCTTT TTCAGCTATA TTTTGGACCT 3150
cDNA_Solyc02g082430  ---------- ---------- ---------- ---------- ---------- 714
        SlMLO6_LEAF  ---------- ---------- ---------- ---------- ---------- 391
        SlMLO6_ROOT  ---------- ---------- ---------- ---------- ---------- 391
      SlMLO6_FLOWER  ---------- ---------- ---------- ---------- ---------- 391
       SlMLO6_FRUIT  ---------- ---------- ---------- ---------- ---------- 391
                              3,160                          3,180                   3,200
gDNA_Solyc02g082430  TTTATGTGGT ACTTATTATT AGCATCAACA GCAAGCTTTC CTTTCTTCAT 3200
cDNA_Solyc02g082430  ---------- ---------- ---------- ---------- ---------- 714
        SlMLO6_LEAF  ---------- ---------- ---------- ---------- ---------- 391
        SlMLO6_ROOT  ---------- ---------- ---------- ---------- ---------- 391
      SlMLO6_FLOWER  ---------- ---------- ---------- ---------- ---------- 391
       SlMLO6_FRUIT  ---------- ---------- ---------- ---------- ---------- 391
                                           3,220                          3,240
gDNA_Solyc02g082430  CAAACTAATC TAAAGTATTC CTATCAGGGA CGTGTACCGC TTGTTACTGT 3250
cDNA_Solyc02g082430  ---------- ---------- -----AGGGA CGTGTACCGC TTGTTACTGT 739
        SlMLO6_LEAF  ---------- ---------- -----AGGGA CGTGTACCGC TTGTTACTGT 416
        SlMLO6_ROOT  ---------- ---------- -----AGGGA CGTGTACCGC TTGTTACTGT 416
      SlMLO6_FLOWER  ---------- ---------- -----AGGGA CGTGTACCGC TTGTTACTGT 416
       SlMLO6_FRUIT  ---------- ---------- -----AGGGA CGTGTACCGC TTGTTACTGT 416
                              3,260                          3,280                   3,300
gDNA_Solyc02g082430  TGAAGCACTG CATCAAATAC ACATCCTCAT TTTCTTTTTG GCAGTCCTTC 3300
cDNA_Solyc02g082430  TGAAGCACTG CATCAAATAC ACATCCTCAT TTTCTTTTTG GCAGTCCTTC 789
        SlMLO6_LEAF  TGAAGCACTG CATCAAATAC ACATCCTCAT TTTCTTTTTG GCAGTCCTTC 466
        SlMLO6_ROOT  TGAAGCACTG CATCAAATAC ACATCCTCAT TTTCTTTTTG GCAGTCCTTC 466
      SlMLO6_FLOWER  TGAAGCACTG CATCAAATAC ACATCCTCAT TTTCTTTTTG GCAGTCCTTC 466
       SlMLO6_FRUIT  TGAAGCACTG CATCAAATAC ACATCCTCAT TTTCTTTTTG GCAGTCCTTC 466
                                           3,320                          3,340
gDNA_Solyc02g082430  ATGTGTTATA CAGTGCGATT ACCATGTGGT TGGGAAGACT TAAGGTAATT 3350
cDNA_Solyc02g082430  ATGTGTTATA CAGTGCGATT ACCATGTGGT TGGGAAGACT TAAG------ 833
        SlMLO6_LEAF  ATGTGTTATA CAGTGCGATT ACCATGTGGT TGGGAAGACT TAAG------ 510
        SlMLO6_ROOT  ATGTGTTATA CAGTGCGATT ACCATGTGGT TGGGAAGACT TAAG------ 510
      SlMLO6_FLOWER  ATGTGTTATA CAGTGCGATT ACCATGTGGT TGGGAAGACT TAAG------ 510
       SlMLO6_FRUIT  ATGTGTTATA CAGTGCGATT ACCATGTGGT TGGGAAGACT TAAG------ 510
                              3,360                          3,380                   3,400
gDNA_Solyc02g082430  CATAATATAC TTTATTTAGT TATTCCTCTA CTATGCTTTA GTTGCCATCT 3400
cDNA_Solyc02g082430  ---------- ---------- ---------- ---------- ---------- 833
        SlMLO6_LEAF  ---------- ---------- ---------- ---------- ---------- 510
        SlMLO6_ROOT  ---------- ---------- ---------- ---------- ---------- 510
      SlMLO6_FLOWER  ---------- ---------- ---------- ---------- ---------- 510
       SlMLO6_FRUIT  ---------- ---------- ---------- ---------- ---------- 510
                                           3,420                          3,440
gDNA_Solyc02g082430  ATACTGCTTC TTAGGTAATC CAGGAAGGTT GTAGGTCCCA ATTATTGACT 3450
cDNA_Solyc02g082430  ---------- ---------- ---------- ---------- ---------- 833
        SlMLO6_LEAF  ---------- ---------- ---------- ---------- ---------- 510
        SlMLO6_ROOT  ---------- ---------- ---------- ---------- ---------- 510
      SlMLO6_FLOWER  ---------- ---------- ---------- ---------- ---------- 510
       SlMLO6_FRUIT  ---------- ---------- ---------- ---------- ---------- 510
                              3,460                          3,480                   3,500
gDNA_Solyc02g082430  GCATTTTTAG TAGATTTTCA ATTAACTCAC AAGATCAGTC AAATTTTCAT 3500
cDNA_Solyc02g082430  ---------- ---------- ---------- ---------- ---------- 833
        SlMLO6_LEAF  ---------- ---------- ---------- ---------- ---------- 510
        SlMLO6_ROOT  ---------- ---------- ---------- ---------- ---------- 510
      SlMLO6_FLOWER  ---------- ---------- ---------- ---------- ---------- 510
       SlMLO6_FRUIT  ---------- ---------- ---------- ---------- ---------- 510
                                           3,520                          3,540
gDNA_Solyc02g082430  GGTGATCCAA TACTCTGGTG GTACTAATGG GAGGATGCAA AAAGCTATAT 3550
cDNA_Solyc02g082430  ---------- ---------- ---------- ---------- ---------- 833
        SlMLO6_LEAF  ---------- ---------- ---------- ---------- ---------- 510
        SlMLO6_ROOT  ---------- ---------- ---------- ---------- ---------- 510
      SlMLO6_FLOWER  ---------- ---------- ---------- ---------- ---------- 510
       SlMLO6_FRUIT  ---------- ---------- ---------- ---------- ---------- 510
                              3,560                          3,580                   3,600
gDNA_Solyc02g082430  GCTTTATGCT TCATTTTGGA TTGTTTCTCT ATCTTACTTG GTTGACACAT 3600
cDNA_Solyc02g082430  ---------- ---------- ---------- ---------- ---------- 833
        SlMLO6_LEAF  ---------- ---------- ---------- ---------- ---------- 510
        SlMLO6_ROOT  ---------- ---------- ---------- ---------- ---------- 510
      SlMLO6_FLOWER  ---------- ---------- ---------- ---------- ---------- 510
       SlMLO6_FRUIT  ---------- ---------- ---------- ---------- ---------- 510
```

3,620 3,640

gDNA_Solyc02g082430	TCAAACAAGT	TAGGAAACTA	AGTTGCATGA	CAAACTGGTC	TTGGTATGAT	3650
cDNA_Solyc02g082430	----------	----------	----------	----------	----------	833
SlMLO6_LEAF	----------	----------	----------	----------	----------	510
SlMLO6_ROOT	----------	----------	----------	----------	----------	510
SlMLO6_FLOWER	----------	----------	----------	----------	----------	510
SlMLO6_FRUIT	----------	----------	----------	----------	----------	510

3,660 3,680 3,700

gDNA_Solyc02g082430	AGGAAGTGTT	CAACTTCAAA	TAATTTTATG	GCAGTTAAGT	GTTGGATCAT	3700
cDNA_Solyc02g082430	----------	----------	----------	----------	----------	833
SlMLO6_LEAF	----------	----------	----------	----------	----------	510
SlMLO6_ROOT	----------	----------	----------	----------	----------	510
SlMLO6_FLOWER	----------	----------	----------	----------	----------	510
SlMLO6_FRUIT	----------	----------	----------	----------	----------	510

3,720 3,740

gDNA_Solyc02g082430	TCAAACTGTT	TTCCCCTAAA	ATCGCTGCTG	TTATACTCCT	AGCTATAATT	3750
cDNA_Solyc02g082430	----------	----------	----------	----------	----------	833
SlMLO6_LEAF	----------	----------	----------	----------	----------	510
SlMLO6_ROOT	----------	----------	----------	----------	----------	510
SlMLO6_FLOWER	----------	----------	----------	----------	----------	510
SlMLO6_FRUIT	----------	----------	----------	----------	----------	510

3,760 3,780 3,800

gDNA_Solyc02g082430	GACTAGCTAC	CAAGGCTCAA	AATTGTTACT	GGAATTTACC	ACTTGTCTAG	3800
cDNA_Solyc02g082430	----------	----------	----------	----------	----------	833
SlMLO6_LEAF	----------	----------	----------	----------	----------	510
SlMLO6_ROOT	----------	----------	----------	----------	----------	510
SlMLO6_FLOWER	----------	----------	----------	----------	----------	510
SlMLO6_FRUIT	----------	----------	----------	----------	----------	510

3,820 3,840

gDNA_Solyc02g082430	AGTGATAAAC	TATTAGGATG	CTTTTATGGT	ATTAATCCAT	TTGCTAAATG	3850
cDNA_Solyc02g082430	----------	----------	----------	----------	----------	833
SlMLO6_LEAF	----------	----------	----------	----------	----------	510
SlMLO6_ROOT	----------	----------	----------	----------	----------	510
SlMLO6_FLOWER	----------	----------	----------	----------	----------	510
SlMLO6_FRUIT	----------	----------	----------	----------	----------	510

3,860 3,880 3,900

gDNA_Solyc02g082430	CCTGAAACTT	TATTCTTCAG	ATTCGTGGCT	GGAAAGGTTG	GGAGCAAGAG	3900
cDNA_Solyc02g082430	----------	----------	ATTCGTGGCT	GGAAAGGTTG	GGAGCAAGAG	863
SlMLO6_LEAF	----------	----------	ATTCGTGGCT	GGAAAGGTTG	GGAGCAAGAG	540
SlMLO6_ROOT	----------	----------	ATTCGTGGCT	GGAAAGGTTG	GGAGCAAGAG	540
SlMLO6_FLOWER	----------	----------	ATTCGTGGCT	GGAAAGGTTG	GGAGCAAGAG	540
SlMLO6_FRUIT	----------	----------	ATTCGTGGCT	GGAAAGGTTG	GGAGCAAGAG	540

3,920 3,940

gDNA_Solyc02g082430	ACTTCAACCC	ATAGTTACGA	GTTTACAAAT	GGTATAAATT	TGCTTGCTGT	3950
cDNA_Solyc02g082430	ACTTCAACCC	ATAGTTACGA	GTTTACAAAT	G---------	----------	894
SlMLO6_LEAF	ACTTCAACCC	ATAGTTACGA	GTTTACAAAT	G---------	----------	571
SlMLO6_ROOT	ACTTCAACCC	ATAGTTACGA	GTTTACAAAT	G---------	----------	571
SlMLO6_FLOWER	ACTTCAACCC	ATAGTTACGA	GTTTACAAAC	G---------	----------	571
SlMLO6_FRUIT	ACTTCAACCC	ATAGTTACGA	GTTTACAAAT	G---------	----------	571

3,960 3,980 4,000

gDNA_Solyc02g082430	AAAGCCAGAA	CTTTGGATTG	TTGCTTGCTC	TCTGTTTATT	TGCTCATTTG	4000
cDNA_Solyc02g082430	----------	----------	----------	----------	----------	894
SlMLO6_LEAF	----------	----------	----------	----------	----------	571
SlMLO6_ROOT	----------	----------	----------	----------	----------	571
SlMLO6_FLOWER	----------	----------	----------	----------	----------	571
SlMLO6_FRUIT	----------	----------	----------	----------	----------	571

4,020 4,040

gDNA_Solyc02g082430	GTTTTGACTC	TCTCACTATG	TTTCTAACTT	GGGTCTTGCA	GATCCTTCAA	4050
cDNA_Solyc02g082430	----------	----------	----------	----------	-ATCCTTCAA	903
SlMLO6_LEAF	----------	----------	----------	----------	-ATCCTTCAA	580
SlMLO6_ROOT	----------	----------	----------	----------	-ATCCTTCAA	580
SlMLO6_FLOWER	----------	----------	----------	----------	-ATCCTTCAA	580
SlMLO6_FRUIT	----------	----------	----------	----------	-ATCCTTCAA	580

4,060 4,080 4,100

gDNA_Solyc02g082430	GATTTAGACT	TACTCATGAG	ACATCTTTTG	TCCGAGCGCA	TACTAGTTTC	4100
cDNA_Solyc02g082430	GATTTAGACT	TACTCATGAG	ACATCTTTTG	TCCGAGCGCA	TACTAGTTTC	953
SlMLO6_LEAF	GATTTAGACT	TACTCATGAG	ACATCTTTTG	TCCGAGCGCA	TACTAGTTTC	630
SlMLO6_ROOT	GATTTAGACT	TACTCATGAG	ACATCTTTTG	TCCGAGCGCA	TACTAGTTTC	630
SlMLO6_FLOWER	GATTTAGACT	TACTCATGAG	ACATCTTTTG	TCCGAGCGCA	TACTAGTTTC	630
SlMLO6_FRUIT	GATTTAGACT	TACTCATGAG	ACATCTTTTG	TCCGAGCGCA	TACTAGTTTC	630

4,120 4,140

gDNA_Solyc02g082430	TGGACAAGGA	TCCCGATCTT	CTTTTACATT	GTGAGGAATT	AATACAATGT	4150
cDNA_Solyc02g082430	TGGACAAGGA	TCCCGATCTT	CTTTTACATT	G---------	----------	984
SlMLO6_LEAF	TGGACAAGGA	TCCCGATCTT	CTTT------	G---------	----------	655
SlMLO6_ROOT	TGGACAAGGA	TCCCGATCTT	CTTTTACATT	G---------	----------	661
SlMLO6_FLOWER	TGGACAAGGA	TCCCGATCTT	CTTTTACATT	G---------	----------	661
SlMLO6_FRUIT	TGGACAAGGA	TCCCGATCTT	CTTTTACATT	G---------	----------	661

4,160 4,180 4,200

gDNA_Solyc02g082430	GCATTCATAG	TTTTGTACCA	GAAGGTCAAA	CTAGTGCTAA	GGTGACTGAC	4200
cDNA_Solyc02g082430	----------	----------	----------	----------	----------	984
SlMLO6_LEAF	----------	----------	----------	----------	----------	655
SlMLO6_ROOT	----------	----------	----------	----------	----------	661
SlMLO6_FLOWER	----------	----------	----------	----------	----------	661
SlMLO6_FRUIT	----------	----------	----------	----------	----------	661

```
                                            4,220                         4,240
gDNA_Solyc02g082430  TGTCGCAAAC TTACATCATG CAGGGATGCT TCTTCAGACA ATTTTTCAAG 4250
cDNA_Solyc02g082430  ---------- ---------- ----GATGCT TCTTCAGACA ATTTTTCAAG 1010
         SlMLO6_LEAF ---------- ---------- ----GATGCT TCTTCAGACA ATTTTTCAAG 681
         SlMLO6_ROOT ---------- ---------- ----GATGCT TCTTCAGACA ATTTTTCAAG 687
       SlMLO6_FLOWER ---------- ---------- ----GATGCT TCTTCAGACA ATTTTTCAAG 687
        SlMLO6_FRUIT ---------- ---------- ----GATGCT TCTTCAGACA ATTTTTCAAG 687
                                 4,260                         4,280                4,300

gDNA_Solyc02g082430  TCTGTCAGTA AGTCAGACTA CTTGGCTCTG CGCAATGGTT TCATAAGTGT 4300
cDNA_Solyc02g082430  TCTGTCAGTA AGTCAGACTA CTTGGCTCTG CGCAATGGTT TCATAAGTGT 1060
         SlMLO6_LEAF TCTGTCAGTA AGTCAGACTA CTTGGCTCTG CGCAATGGTT TCATAAGTGT 731
         SlMLO6_ROOT TCTGTCAGTA AGTCAGACTA CTTGGCTCTG CGCAATGGTT TCATAAGTGT 737
       SlMLO6_FLOWER TCTGTCAGTA AGTCAGACTA CTTGGCTCTG CGCAATGGTT TCATAAGTGT 737
        SlMLO6_FRUIT TCTGTCAGTA AGTCAGACTA CTTGGCTCTG CGCAATGGTT TCATAAGTGT 737
                                            4,320                         4,340

gDNA_Solyc02g082430  GAGTGTTCAT TGTGATTTGT TCTTCCTTGG TAATTCTTTG TTACCCATGA 4350
cDNA_Solyc02g082430  ---------- ---------- ---------- ---------- ---------- 1060
         SlMLO6_LEAF ---------- ---------- ---------- ---------- ---------- 731
         SlMLO6_ROOT ---------- ---------- ---------- ---------- ---------- 737
       SlMLO6_FLOWER ---------- ---------- ---------- ---------- ---------- 737
        SlMLO6_FRUIT ---------- ---------- ---------- ---------- ---------- 737
                                 4,360                         4,380                4,400

gDNA_Solyc02g082430  AACTAAAATT CCTCCTTTCG CAGGTTCATC TGGCTCCTGG AAGTAAATTT 4400
cDNA_Solyc02g082430  ---------- ---------- -----TCATC TGGCTCCTGG AAGTAAATTT 1085
         SlMLO6_LEAF ---------- ---------- -----GCATC TGGCTCCTGG AAGTAAATTT 756
         SlMLO6_ROOT ---------- ---------- -----TCATC TGGCTCCTGG AAGTAAATTT 762
       SlMLO6_FLOWER ---------- ---------- -----TCATC TGGCTCCTGG AAGTAAATTT 762
        SlMLO6_FRUIT ---------- ---------- -----TCATC TGGCTCCTGG AAGTAAATTT 762
                                            4,420                         4,440

gDNA_Solyc02g082430  AACTTCCAAA AGTACATCAA GAGGTCATTA GAGGATGACT TCAAGGTAGT 4450
cDNA_Solyc02g082430  AACTTCCAAA AGTACATCAA GAGGTCATTA GAGGATGACT TCAAGGTAGT 1135
         SlMLO6_LEAF AACTTCCAAA AGTACATCAA GAGGTCATTA GAGGATGACT TCAAGGTAGT 806
         SlMLO6_ROOT AACTTCCAAA AGTACATCAA GAGGACATTA GAGGATGACT TCAAGGTAGT 812
       SlMLO6_FLOWER AACTTCCAAA AGTACATCAA GAGGACATTA GAGGATGACT TCAAGGTAGT 812
        SlMLO6_FRUIT AACTTCCAAA AGTACATCAA GAGGACATTA GAGGATGACT TCAAGGTAGT 812
                                 4,460                         4,480                4,500

gDNA_Solyc02g082430  TGTCGGTGTC AGGTAGCTAG ATGCTCTATT TGCTCTCTCA ATTTTTTCAG 4500
cDNA_Solyc02g082430  TGTCGGTGTC AG-------- ---------- ---------- ---------- 1147
         SlMLO6_LEAF TGTCGGTGTC AG-------- ---------- ---------- ---------- 818
         SlMLO6_ROOT TGTCGGTGTC AG-------- ---------- ---------- ---------- 824
       SlMLO6_FLOWER TGTCGGTGTC AG-------- ---------- ---------- ---------- 824
        SlMLO6_FRUIT TGTCGGTGTC AG-------- ---------- ---------- ---------- 824
                                            4,520                         4,540

gDNA_Solyc02g082430  CATATTTATG TGTGCATATG TGCCTAATTA CCATATATAT CCTTGATCTC 4550
cDNA_Solyc02g082430  ---------- ---------- ---------- ---------- ---------- 1147
         SlMLO6_LEAF ---------- ---------- ---------- ---------- ---------- 818
         SlMLO6_ROOT ---------- ---------- ---------- ---------- ---------- 824
       SlMLO6_FLOWER ---------- ---------- ---------- ---------- ---------- 824
        SlMLO6_FRUIT ---------- ---------- ---------- ---------- ---------- 824
                                 4,560                         4,580                4,600

gDNA_Solyc02g082430  TGATTGGCTT TTTACTTGTG TTTTTTTTCC GGGCAGTCCA GTTTTATGGG 4600
cDNA_Solyc02g082430  ---------- ---------- ---------- ------TCCA GTTTTATGGG 1161
         SlMLO6_LEAF ---------- ---------- ---------- ---CAGTCCA GTTTTATGGG 835
         SlMLO6_ROOT ---------- ---------- ---------- ------TCCA GTTTTATGGG 838
       SlMLO6_FLOWER ---------- ---------- ---------- ------TCCA GTTTTATGGG 838
        SlMLO6_FRUIT ---------- ---------- ---------- ------TCCA GTTTTATGGG 838
                                            4,620                         4,640

gDNA_Solyc02g082430  CATCATTTGT GCTTTTCTTG CTTCTGAATG TTAGCGGTAA GTTATGTTCT 4650
cDNA_Solyc02g082430  CATCATTTGT GCTTTTCTTG CTTCTGAATG TTAGCGG--- ---------- 1198
         SlMLO6_LEAF CATCATTTGT GCTTTTCTTG CTTCTGAATG TTAGCGG--- ---------- 872
         SlMLO6_ROOT CATCATTTGT GCTTTTCTTG CTTCTGAATG TTAGCGG--- ---------- 875
       SlMLO6_FLOWER CATCATTTGT GCTTTTCTTG CTTCTGAATG TTAGCGG--- ---------- 875
        SlMLO6_FRUIT CATCATTTGT GCTTTTCTTG CTTCTGAATG TTAGCGG--- ---------- 875
                                 4,660                         4,680                4,700

gDNA_Solyc02g082430  GAGGTCTGAT CCATTTCTGG AAATTGTTTA GTTCTTCCGT TTTAGTCACC 4700
cDNA_Solyc02g082430  ---------- ---------- ---------- ---------- ---------- 1198
         SlMLO6_LEAF ---------- ---------- ---------- ---------- ---------- 872
         SlMLO6_ROOT ---------- ---------- ---------- ---------- ---------- 875
       SlMLO6_FLOWER ---------- ---------- ---------- ---------- ---------- 875
        SlMLO6_FRUIT ---------- ---------- ---------- ---------- ---------- 875
                                            4,720                         4,740

gDNA_Solyc02g082430  ATCTACTACA TGTTTTCATA TATTCTGAAT TATTTGACCC ATTGCAGGGT 4750
cDNA_Solyc02g082430  ---------- ---------- ---------- ---------- --------GT 1200
         SlMLO6_LEAF ---------- ---------- ---------- ---------- --------GT 874
         SlMLO6_ROOT ---------- ---------- ---------- ---------- --------GT 877
       SlMLO6_FLOWER ---------- ---------- ---------- ---------- --------GT 877
        SlMLO6_FRUIT ---------- ---------- ---------- ---------- --------GT 877
                                 4,760                         4,780                4,800

gDNA_Solyc02g082430  GGCAAGCATT GTTCTGGGCA TCCTTAATTC CTCTAATTGT AAGATTCTTT 4800
cDNA_Solyc02g082430  GGCAAGCATT GTTCTGGGCA TCCTTAATTC CTCTAATT-- ---------- 1238
         SlMLO6_LEAF GGCAAGCATT GTTCTGGGCA TCCTTAATTC CTCTAATT-- ---------- 912
         SlMLO6_ROOT GGCAAGCATT GTTCTGGGCA TCCTTAATTC CTCTAATT-- ---------- 915
       SlMLO6_FLOWER GGCAAGCATT GTTCTGGGCA TCCTTAATTC CTCTAATT-- ---------- 915
        SlMLO6_FRUIT GGCAAGCATT GTTCTGGGCA TCCTTAATTC CTCTAATT-- ---------- 915
```

```
                                     4,820                      4,840
gDNA_Solyc02g082430  TCCATTGTTC ATCTTCTGAA ACTACTTTAT GGGGAAATTC TAATTAATGA 4850
cDNA_Solyc02g082430  ---------- ---------- ---------- ---------- ---------- 1238
        SlMLO6_LEAF  ---------- ---------- ---------- ---------- ---------- 912
        SlMLO6_ROOT  ---------- ---------- ---------- ---------- ---------- 915
      SlMLO6_FLOWER  ---------- ---------- ---------- ---------- ---------- 915
       SlMLO6_FRUIT  ---------- ---------- ---------- ---------- ---------- 915

                          4,860                      4,880                 4,900
gDNA_Solyc02g082430  AATCTTCTCA GATCATTTTA GCTGTTGGAA CAAAGCTTCA AGCTGTTTTG 4900
cDNA_Solyc02g082430  ---------- -ATCATTTTA GCTGTTGGAA CAAAGCTTCA AGCTGTTTTG 1277
        SlMLO6_LEAF  ---------- -ATCATTTTA GCTGTTGGAA CAAAGCTTCA AGCTGTTTTG 951
        SlMLO6_ROOT  ---------- -ATCATTTTA GCTGTTGGAA CAAAGCTTCA AGCTGTTTTG 954
      SlMLO6_FLOWER  ---------- -ATCATTTTA GCTGTTGGAA CAAAGCTTCA AGCTGTTTTG 954
       SlMLO6_FRUIT  ---------- -ATCATTTTA GCTGTTGGAA CAAAGCTTCA AGCTGTTTTG 954

                                     4,920                      4,940
gDNA_Solyc02g082430  ACTAGGATGG CCCTTGACAT TAAAGAGAGA CATGCAGTAG TTCAAGGAAT 4950
cDNA_Solyc02g082430  ACTAGGATGG CCCTTGACAT TAAAGAGAGA CATGCAGTAG TTCAAGGAAT 1327
        SlMLO6_LEAF  ACTAGGATGG CCCTTGACAT TAAAGAGAGA CATGCAGTAG TTCAAGGAAT 1001
        SlMLO6_ROOT  ACTAGGATGG CCCTTGACAT TAAAGAGAGA CATGCAGTAG TTCAAGGAAT 1004
      SlMLO6_FLOWER  ACTAGGATGG CCCTTGACAT TAAAGAGAGA CATGCAGTAG TTCAAGGAAT 1004
       SlMLO6_FRUIT  ACTAGGATGG CCCTTGACAT TAAAGAGAGA CATGCAGTAG TTCAAGGAAT 1004

                          4,960                      4,980                 5,000
gDNA_Solyc02g082430  CCCTCTTGTA CAAGCCTCAG ACAAATATTT TTGGTTTGGT CGACCACGAC 5000
cDNA_Solyc02g082430  CCCTCTTGTA CAAGCCTCAG ACAAATATTT TTGGTTTGGT CGACCACGAC 1377
        SlMLO6_LEAF  CCCTCTTGTA CAAGCCTCAG ACAAATATTT TTGGTTTGGT CGACCACGAC 1051
        SlMLO6_ROOT  CCCTCTTGTA CAAGCCTCAG ACAAATATTT TTGGTTTGGT CGACCACGAC 1054
      SlMLO6_FLOWER  CCCTCTTGTA CAAGCCTCAG ACAAATATTT TTGGTTTGGT CGACCACGAC 1054
       SlMLO6_FRUIT  CCCTCTTGTA CAAGCCTCGG ACAAATATTT TTGGTTTGGT CGACCACGAC 1054

                                     5,020                      5,040
gDNA_Solyc02g082430  TGGTTCTTCA CCTCATTCAT TTTGCCTTGT TTCAGGTTTG CATTTCCAAT 5050
cDNA_Solyc02g082430  TGGTTCTTCA CCTCATTCAT TTTGCCTTGT TTCAG----- ---------- 1412
        SlMLO6_LEAF  TGGTTCTTCA CCTCATTCAT TTTGCCTTGT TTCAG----- ---------- 1086
        SlMLO6_ROOT  TGGTTCTTCA CCTCATTCAT TTTGCCTTGT TTCAG----- ---------- 1089
      SlMLO6_FLOWER  TGGTTCTTCA CCTCATTCAT TTTGCCTTGT TTCAG----- ---------- 1089
       SlMLO6_FRUIT  TGGTTCTTCA CCTCATTCAT TTTGCCTTGT TTCAG----- ---------- 1089

                          5,060                      5,080                 5,100
gDNA_Solyc02g082430  TTGCAGAATT TTAAAGAAGT ATTATTAGTA AACCAGTCGT TTGGGCTCAA 5100
cDNA_Solyc02g082430  ---------- ---------- ---------- ---------- ---------- 1412
        SlMLO6_LEAF  ---------- ---------- ---------- ---------- ---------- 1086
        SlMLO6_ROOT  ---------- ---------- ---------- ---------- ---------- 1089
      SlMLO6_FLOWER  ---------- ---------- ---------- ---------- ---------- 1089
       SlMLO6_FRUIT  ---------- ---------- ---------- ---------- ---------- 1089

                                     5,120                      5,140
gDNA_Solyc02g082430  CTTTGCAAGT ACTATAGTTT CCATAAGACA AATCACAAAT ATGGTGCATG 5150
cDNA_Solyc02g082430  ---------- ---------- ---------- ---------- ---------- 1412
        SlMLO6_LEAF  ---------- ---------- ---------- ---------- ---------- 1086
        SlMLO6_ROOT  ---------- ---------- ---------- ---------- ---------- 1089
      SlMLO6_FLOWER  ---------- ---------- ---------- ---------- ---------- 1089
       SlMLO6_FRUIT  ---------- ---------- ---------- ---------- ---------- 1089

                          5,160                      5,180                 5,200
gDNA_Solyc02g082430  GAGTAGTTTG CTTTTCATAT CTATATATTC CCTTGGCTAA ACTCTTTTTT 5200
cDNA_Solyc02g082430  ---------- ---------- ---------- ---------- ---------- 1412
        SlMLO6_LEAF  ---------- ---------- ---------- ---------- ---------- 1086
        SlMLO6_ROOT  ---------- ---------- ---------- ---------- ---------- 1089
      SlMLO6_FLOWER  ---------- ---------- ---------- ---------- ---------- 1089
       SlMLO6_FRUIT  ---------- ---------- ---------- ---------- ---------- 1089

                                     5,220                      5,240
gDNA_Solyc02g082430  TGCACTTATC AAACTTTGCA GAATGCATTC CAGATAACAT ATTTTCTGTG 5250
cDNA_Solyc02g082430  ---------- ---------- -AATGCATTC CAGATAACAT ATTTTCTGTG 1441
        SlMLO6_LEAF  ---------- --------CA GAATGCATTC CAGATAACAT ATTTTCTGTG 1118
        SlMLO6_ROOT  ---------- ---------- -AATGCATTC CAGATAACAT ATTTTCTGTG 1118
      SlMLO6_FLOWER  ---------- ---------- -AATGCATTC CAGATAACAT ATTTTCTGTG 1118
       SlMLO6_FRUIT  ---------- ---------- -AATGCATTC CAGATAACAT ATTTTCTGTG 1118

                          5,260                      5,280                 5,300
gDNA_Solyc02g082430  GATATGGGTA ATAACTCTTC TCCCTGTATC AAGATCAAAT TCTGATATTG 5300
cDNA_Solyc02g082430  GATATGG--- ---------- ---------- ---------- ---------- 1448
        SlMLO6_LEAF  GATATGG--- ---------- ---------- ---------- ---------- 1125
        SlMLO6_ROOT  GATATGG--- ---------- ---------- ---------- ---------- 1125
      SlMLO6_FLOWER  GATATGG--- ---------- ---------- ---------- ---------- 1125
       SlMLO6_FRUIT  GATATGG--- ---------- ---------- ---------- ---------- 1125

                                     5,320                      5,340
gDNA_Solyc02g082430  CAAGATATTC TTTTTCTTTT TATTTAATGT CCATAAAACT ATGATAGAGA 5350
cDNA_Solyc02g082430  ---------- ---------- ---------- ---------- ---------- 1448
        SlMLO6_LEAF  ---------- ---------- ---------- ---------- ---------- 1125
        SlMLO6_ROOT  ---------- ---------- ---------- ---------- ---------- 1125
      SlMLO6_FLOWER  ---------- ---------- ---------- ---------- ---------- 1125
       SlMLO6_FRUIT  ---------- ---------- ---------- ---------- ---------- 1125

                          5,360                      5,380                 5,400
gDNA_Solyc02g082430  TGAGGATTCT ACTTTTTCTG TAGTATGAGT ATGGGCTAAA ATCTTGCTTC 5400
cDNA_Solyc02g082430  ---------- ---------- ---TATGAGT ATGGGCTAAA ATCTTGCTTC 1475
        SlMLO6_LEAF  ---------- ---------- ---TATGAGT ATGGGCTAAA ATCTTGCTTC 1152
        SlMLO6_ROOT  ---------- ---------- ---TATGAGT ATGGGCTAAA ATCTTGCTTC 1152
      SlMLO6_FLOWER  ---------- ---------- ---TATGAGT ATGGGCTAAA ATCTTGCTTC 1152
       SlMLO6_FRUIT  ---------- ---------- ---TATGAGT ATGGGCTAAA ATCTTGCTTC 1152
```

```
                                          5,420                         5,440
gDNA_Solyc02g082430  CACGAGGCGT TTGAGCTGGT CATTGCAAAA ATTGTTATAG GGTATGTTTC 5450
cDNA_Solyc02g082430  CACGAGGCGT TTGAGCTGGT CATTGCAAAA ATTGTTATAG G--------- 1516
         SlMLO6_LEAF  CACGAGGCGT TTGAGCTGGT CATTGCAAAA ATTGTTATAG G--------- 1193
         SlMLO6_ROOT  CACGAGGCGT TTGAGCTGGT CATTGCAAAA ATTGTTATAG G--------- 1193
       SlMLO6_FLOWER  CACGAGGCGT TTGAGCTGGT CATTGCAAAA ATTGTTATAG G--------- 1193
        SlMLO6_FRUIT  CACGAGGCGT TTGAGCTGGT CATTGCAAAA ATTGTTATAG G--------- 1193
                               5,460                         5,480                  5,500
gDNA_Solyc02g082430  GTGCTACAGA AACTCACTTT AAAATATTCA TTATCTTAGT TCCTAAGCTT 5500
cDNA_Solyc02g082430  ---------- ---------- ---------- ---------- ---------- 1516
         SlMLO6_LEAF  ---------- ---------- ---------- ---------- ---------- 1193
         SlMLO6_ROOT  ---------- ---------- ---------- ---------- ---------- 1193
       SlMLO6_FLOWER  ---------- ---------- ---------- ---------- ---------- 1193
        SlMLO6_FRUIT  ---------- ---------- ---------- ---------- ---------- 1193
                                          5,520                         5,540
gDNA_Solyc02g082430  CACAATTTCA GCTTGCTCTC GCTAAATCAA TTTTTTTCAT TGTATTACAG 5550
cDNA_Solyc02g082430  ---------- ---------- ---------- ---------- ---------- 1516
         SlMLO6_LEAF  ---------- ---------- ---------- ---------- ---------- 1193
         SlMLO6_ROOT  ---------- ---------- ---------- ---------- ---------- 1193
       SlMLO6_FLOWER  ---------- ---------- ---------- ---------- ---------- 1193
        SlMLO6_FRUIT  ---------- ---------- ---------- ---------- ---------- 1193
                               5,560                         5,580                  5,600
gDNA_Solyc02g082430  AGTGGGAGTC TTATTCCTAT GCAGTTATAT CACTCTTCCG CTTTATGCCC 5600
cDNA_Solyc02g082430  AGTGGGAGTC TTATTCCTAT GCAGTTATAT CACTCTTCCG CTTTATGCCC 1566
         SlMLO6_LEAF  G--------- ---TTCCTAT GCAGTTATAT CACTCTTCCG CTTTATGCCC 1231
         SlMLO6_ROOT  AGTGGGAGTC TTATTCCTAT GCAGTTATAT CACTCTTCCG CTTTATGCCC 1243
       SlMLO6_FLOWER  AGTGGGAGTC TTATTCCTAT GCAGTTATAT CACTCTTCCG CTTTATGCCC 1243
        SlMLO6_FRUIT  AGTGGGAGTC TTATTCCTAT ACAGTTATAT CACTCTTCCG CTTTATGCCC 1243
                                          5,620                         5,640
gDNA_Solyc02g082430  TTATAACTCA GGTACTATCA TAGGCTCGTC TCTCAAACCA ATAGTATAAT 5650
cDNA_Solyc02g082430  TTATAACTCA G--------- ---------- ---------- ---------- 1577
         SlMLO6_LEAF  TTATAACTCA G--------- ---------- ---------- ---------- 1242
         SlMLO6_ROOT  TTATAACTCA G--------- ---------- ---------- ---------- 1254
       SlMLO6_FLOWER  TTATAACTCA G--------- ---------- ---------- ---------- 1254
        SlMLO6_FRUIT  TTATAACTCA G--------- ---------- ---------- ---------- 1254
                               5,660                         5,680                  5,700
gDNA_Solyc02g082430  TGTATCTACG TTTTATTTCG TGCCATATTA CCTTAGACAT TTGCTATTGC 5700
cDNA_Solyc02g082430  ---------- ---------- ---------- ---------- ---------- 1577
         SlMLO6_LEAF  ---------- ---------- ---------- ---------- ---------- 1242
         SlMLO6_ROOT  ---------- ---------- ---------- ---------- ---------- 1254
       SlMLO6_FLOWER  ---------- ---------- ---------- ---------- ---------- 1254
        SlMLO6_FRUIT  ---------- ---------- ---------- ---------- ---------- 1254
                                          5,720                         5,740
gDNA_Solyc02g082430  TCACTTCGTT GAACAATGTA AGCTCACTAC ATAAAACTAC TGAAATTTCT 5750
cDNA_Solyc02g082430  ---------- ---------- ---------- ---------- ---------- 1577
         SlMLO6_LEAF  ---------- ---------- ---------- ---------- ---------- 1242
         SlMLO6_ROOT  ---------- ---------- ---------- ---------- ---------- 1254
       SlMLO6_FLOWER  ---------- ---------- ---------- ---------- ---------- 1254
        SlMLO6_FRUIT  ---------- ---------- ---------- ---------- ---------- 1254
                               5,760                         5,780                  5,800
gDNA_Solyc02g082430  AGCCTTTTTA GCATGATCTA TTGTTGTTAA GTTAGATAAA GCATTGGCCT 5800
cDNA_Solyc02g082430  ---------- ---------- ---------- ---------- ---------- 1577
         SlMLO6_LEAF  ---------- ---------- ---------- ---------- ---------- 1242
         SlMLO6_ROOT  ---------- ---------- ---------- ---------- ---------- 1254
       SlMLO6_FLOWER  ---------- ---------- ---------- ---------- ---------- 1254
        SlMLO6_FRUIT  ---------- ---------- ---------- ---------- ---------- 1254
                                          5,820                         5,840
gDNA_Solyc02g082430  TTCTGCAGAT GGGATCCCAT ATGAAAAAAT CCATCTTTGA TGAGCAAACC 5850
cDNA_Solyc02g082430  --------AT GGGATCCCAT ATGAAAAAAT CCATCTTTGA TGAGCAAACC 1619
         SlMLO6_LEAF  --------AT GGGATCCCAT ATGAAAAAAT CCATCTTTGA TGAGCAAACC 1284
         SlMLO6_ROOT  --------AT GGGATCCCAT ATGAAAAAAT CCATCTTTGA TGAGCAAACC 1296
       SlMLO6_FLOWER  --------AT GGGATCCCAT ATGAAAAAAT CCATCTTTGA TGAGCAAACC 1296
        SlMLO6_FRUIT  --------AT GGGATCCCAT ATGAAAAAAT CCATCTTTGA TGAGCAAACC 1296
                               5,860                         5,880                  5,900
gDNA_Solyc02g082430  TCCAAGGCAC TAAAGAAGTG GCATATGGCT GTGAAGAAGC GGACAGGAGC 5900
cDNA_Solyc02g082430  TCCAAGGCAC TAAAGAAGTG GCATATGGCT GTGAAGAAGC GGACAGGAGC 1669
         SlMLO6_LEAF  TCCAAGGCAC TAAAGAAGTG GCATATGGCT GTGAAGAAGC GGACAGGAGC 1334
         SlMLO6_ROOT  TCCAAGGCAC TAAAGAAGTG GCATATGGCT GTGAAGAAGC GGACAGGAGC 1346
       SlMLO6_FLOWER  TCCAAGGCAC TAAAGAAGTG GCATATGGCT GTGAAGAAGC GGACAGGAGC 1346
        SlMLO6_FRUIT  TCCAAGGCAC TAAAGAAGTG GCATATGGCT GTGAAGAAGC GGACAGGAGC 1346
                                          5,920                         5,940
gDNA_Solyc02g082430  GAGGGGTGAC AGGTCCCCTA CCCGAACACT GGGTAATGCA AGTCCAAGGT 5950
cDNA_Solyc02g082430  GAGGGGTGAC AGGTCCCCTA CCCGAACACT GGGTAATGCA AGTCCAAGGT 1719
         SlMLO6_LEAF  GAGGGGTGAC AGGTCCCCTA CCCGAACACT GGGTAATGCA AGTCCAAGGT 1384
         SlMLO6_ROOT  GAGGGGTGAC AGGTCCCCTA CCCGAACACT GGGTAATGCA AGTCCAAGGT 1396
       SlMLO6_FLOWER  GAGGGGTGAC AGGTCCCCTA CCCGAACACT GGGTAATGCA AGTCCAAGGT 1396
        SlMLO6_FRUIT  GAGGGGTGAC AGGTCCCCTA CCCGAACACT GGGTAATGCA AGTCCAAGGT 1396
                               5,960                         5,980                  6,000
gDNA_Solyc02g082430  CTGCAATGAG CTCACCTGTG CACCCATCAG GCCCTGGTCT TCATCGATAC 6000
cDNA_Solyc02g082430  CTGCAATGAG CTCACCTGTG CACCCATCAG GCCCTGGTCT TCATCGATAC 1769
         SlMLO6_LEAF  CTGCAATGAG CTCACCTGTG CACCCATCAG GCCCTGGTCT TCATCGATAC 1434
         SlMLO6_ROOT  CTGCAATGAG CTCACCTGTG CACCCATCAG GCCCTGGTCT TCATCGATAC 1446
       SlMLO6_FLOWER  CTGCAATGAG CTCACCTGTG CACCCATCAG GCCCTGGTCT TCATCGATAC 1446
        SlMLO6_FRUIT  CTGCAATGAG CTCACCTGTG CACCCATCAG GCCCTGGTCT TCATCGATAC 1446
```

```
                                          6,020                         6,040
gDNA_Solyc02g082430 AAAACCACTG GTCACTCATC GCGTTTCCAG GGCTACAGTG ATCAAGAAGC 6050
cDNA_Solyc02g082430 AAAACCACTG GTCACTCATC GCGTTTCCAG GGCTACAGTG ATCAAGAAGC 1819
         SIMLO6_LEAF AAAACCACTG GTCACTCATC GCGTTTCCAG GGCTACAGTG ATCAAGAAGC 1484
         SIMLO6_ROOT AAAACCACTG GTCACTCATC GCGTTTCCAG GGCTACAGTG ATCAAGAAGC 1496
       SIMLO6_FLOWER AAAACCACTG GTCACTCATC GCGTTTCCAG GGCTACAGTG ATCAAGAAGC 1496
        SIMLO6_FRUIT AAAACCACTG GTCACTCATC GCGTTTCCAG GGCTACAGTG ATCAAGAAGC 1496
                               6,060                         6,080                    6,100

gDNA_Solyc02g082430 ATCCGACCTT GAAAATGATC CAACAACACC GATGACTCGT GCAGAGATAG 6100
cDNA_Solyc02g082430 ATCCGACCTT GAAAATGATC CAACAACACC GATGACTCGT GCAGAGATAG 1869
         SIMLO6_LEAF ATCCGACCTT GAAAATGATC CAACAACACC GATGACTCGT GCAGAGATAG 1534
         SIMLO6_ROOT ATCCGACCTT GAAAATGATC CAACAACACC GATGACTCGT GCAGAGATAG 1546
       SIMLO6_FLOWER ATCCGACCTT GAAAATGATC CAACAACACC GATGACTCGT GCAGAGATAG 1546
        SIMLO6_FRUIT ATCCGACCTT GAAAATGATC CAACAACACC GATGACTCGT GCAGAGATAG 1546
                                          6,120                         6,140

gDNA_Solyc02g082430 CAACTACACA TATTGATCAT GATGACACAG AAATTCATGT GCATATTCCT 6150
cDNA_Solyc02g082430 CAACTACACA TATTGATCAT GATGACACAG AAATTCATGT GCATATTCCT 1919
         SIMLO6_LEAF CAACTACACA TATTGATCAT GATGACACAG AAATTCATGT GCATATTCCT 1584
         SIMLO6_ROOT CAACTACACA TATTGATCAT GATGACACAG AAATTCATGT GCATATTCCT 1596
       SIMLO6_FLOWER CAACTACACA TATTGATCAT GATGACACAG AAATTCATGT GCATATTCCT 1596
        SIMLO6_FRUIT CAACTACACA TATTGATCAT GATGACACAG AAATTCATGT GCATATTCCT 1596
                               6,160                         6,180                    6,200

gDNA_Solyc02g082430 CAAAATGGAG AATCTACTAG GAATGAAGAT GACTTCTCAT TTGTGAAGCC 6200
cDNA_Solyc02g082430 CAAAATGGAG AATCTACTAG GAATGAAGAT GACTTCTCAT TTGTGAAGCC 1969
         SIMLO6_LEAF CAAAATGGAG AATCTACTAG GAATGAAGAT GACTTCTCAT TTGTGAAGCC 1634
         SIMLO6_ROOT CAAAATGGAG AATCTACTAG GAATGAAGAT GACTTCTCAT TTGTGAAGCC 1646
       SIMLO6_FLOWER CAAAATGGAG AATCTACTAG GAATGAAGAT GACTTCTCAT TTGTGAAGCC 1646
        SIMLO6_FRUIT CAAAATGGAG AATCTACTAG GAATGAAGAT GACTTCTCAT TTATGAAGCC 1646
                                          6,220                         6,240

gDNA_Solyc02g082430 TGCTCCTCAA AGGTGAAAAG GAAGTAGGTA GCCAACTTGT TACCTTTTTT 6250
cDNA_Solyc02g082430 TGCTCCTCAA AGGTGAAAAG GAAGTAGGTA GCCAACTTGT TACCTTTTTT 2019
         SIMLO6_LEAF TGCTCCTCAA AGGTGA---- ---------- ---------- ---------- 1650
         SIMLO6_ROOT TGCTCCTCAA AGGTGA---- ---------- ---------- ---------- 1662
       SIMLO6_FLOWER TGCTCCTCAA AGGTGA---- ---------- ---------- ---------- 1662
        SIMLO6_FRUIT TGCTCCTCAA AGGTGA---- ---------- ---------- ---------- 1662
                               6,260                         6,280                    6,300

gDNA_Solyc02g082430 TTTTTTTAAT CCTTTTCCTT TTCCCTCAAG TCATATCAAT GTGCTTATAC 6300
cDNA_Solyc02g082430 TTTTTTTAAT CCTTTTCCTT TTCCCTCAAG TCATATCAAT GTGCTTATAC 2069
         SIMLO6_LEAF ---------- ---------- ---------- ---------- ---------- 1650
         SIMLO6_ROOT ---------- ---------- ---------- ---------- ---------- 1662
       SIMLO6_FLOWER ---------- ---------- ---------- ---------- ---------- 1662
        SIMLO6_FRUIT ---------- ---------- ---------- ---------- ---------- 1662
                                          6,320                         6,340

gDNA_Solyc02g082430 ATTTTAGGTA CATCATATAA GCAGTTTGGC ACATATAGGT GAATTCCTTG 6350
cDNA_Solyc02g082430 ATTTTAGGTA CATCATATAA GCAGTTTGGC ACATATAGGT GAATTCCTTG 2119
         SIMLO6_LEAF ---------- ---------- ---------- ---------- ---------- 1650
         SIMLO6_ROOT ---------- ---------- ---------- ---------- ---------- 1662
       SIMLO6_FLOWER ---------- ---------- ---------- ---------- ---------- 1662
        SIMLO6_FRUIT ---------- ---------- ---------- ---------- ---------- 1662
                               6,360                         6,380

gDNA_Solyc02g082430 TACTATATTT TTACTCTCTA ATTGTTTGCC TTAATAAG ⟫6388
cDNA_Solyc02g082430 TACTATATTT TTACTCTCTA ATTGTTTGCC TTAATAAG ⟫2157
         SIMLO6_LEAF ---------- ---------- ---------- --------  1650
         SIMLO6_ROOT ---------- ---------- ---------- -------- ⟫1662
       SIMLO6_FLOWER ---------- ---------- ---------- -------- ⟫1662
        SIMLO6_FRUIT ---------- ---------- ---------- -------- ⟫1662
```

```
                                                                  20                   40
gDNA_Solyc09g018830+Solyc09g018840  ATGAGTGGCG GAGAAGGAGA AGCAGAAGGG TCAACATTGG AATTTACCCC  50
                     SIMLO7_FGENESH  ATGAGTGGCG GAGAAGGAGA AGCAGAAGGG TCAACATTGG AATTTACCCC  50
                        SIMLO7_LEAF  ATGAGTGGCG GAGAAGGAGA AGCAGAAGGG TCAACATTGG AATTTACCCC  50
                                             60                   80                   100
gDNA_Solyc09g018830+Solyc09g018840  TACTTGGGTG GTGGCCACCG TCTGCACCGT CATCGCTGCC ATTTCTCTTG 100
                     SIMLO7_FGENESH  TACTTGGGTG GTGGCCACCG TCTGCACCGT CATCGCTGCC ATTTCTCTTG 100
                        SIMLO7_LEAF  TACTTGGGTG GTGGCCACCG TCTGCACCGT CATCGCTGCC ATTTCTCTTG 100
                                                        120                  140
gDNA_Solyc09g018830+Solyc09g018840  CCGTTGAGCG TCTCGTCCAT TATACTGGAA AGGTATGTAT ATAAATTATT 150
                     SIMLO7_FGENESH  CCGTTGAGCG TCTCGTCCAT TATACTGGAA AG-------- ---------- 132
                        SIMLO7_LEAF  CCGTTGAGCG TCTCGTCCAT TATACTGGAA AG-------- ---------- 132
                                             160                  180                  200
gDNA_Solyc09g018830+Solyc09g018840  CTTTTTTTAA TTCAATTTCA TATTCAACTC ACATACTATC TCTTCACAGT 200
                     SIMLO7_FGENESH  ---------- ---------- ---------- ---------- ---------T 133
                        SIMLO7_LEAF  ---------- ---------- ---------- ---------- ---------T 133
                                                        220                  240
gDNA_Solyc09g018830+Solyc09g018840  ATCTGAAGAA GAAGAATCAG AAGCCTTTAT ATGAAGCGCT ACAGAAAGTT 250
                     SIMLO7_FGENESH  ATCTGAAGAA GAAGAATCAG AAGCCTTTAT ATGAAGCGCT ACAGAAAGTT 183
                        SIMLO7_LEAF  ATCTGAAGAA GAAGAATCAG AAGCCTTTAT ATGAAGCGCT ACAGAAA--- 180
                                             260                  280                  300
gDNA_Solyc09g018830+Solyc09g018840  AAAGAAGGTC CATACCAAGA TCTGTAAATT TTTAATTCTA GTTATAATTA 300
                     SIMLO7_FGENESH  AAAGAAG--- ---------- ---------- ---------- ---------- 190
                        SIMLO7_LEAF  ---------- ---------- ---------- ---------- ---------- 180
                                                        320                  340
gDNA_Solyc09g018830+Solyc09g018840  ATTTGTAATG TTGAATATGC AGAGTTGATG CTTTTGGGGT TTATATCCCT 350
                     SIMLO7_FGENESH  ---------- ---------- --AGTTGATG CTTTTGGGGT TTATATCCCT 218
                        SIMLO7_LEAF  ---------- ---------- --AGTTGATG CTTTTGGGGT TTATATCCCT 208
                                             360                  380                  400
gDNA_Solyc09g018830+Solyc09g018840  TTTACTGACG GTTCTTCAAA GTAGCATAAT CAAGATTTGT GTGCCTAACC 400
                     SIMLO7_FGENESH  TTTACTGACG GTTCTTCAAA GTAGCATAAT CAAGATTTGT GTGCCTAACC 268
                        SIMLO7_LEAF  TTTACTGACG GTTCTTCAAA GTAGCATAAT CAAGATTTGT GTGCCTAACC 258
                                                        420                  440
gDNA_Solyc09g018830+Solyc09g018840  ATGTGGTCTT GCACTTGCTA CCTTGTTCAT TATCTCATGA GTCACAACAT 450
                     SIMLO7_FGENESH  ATGTGGTCTT GCACTTGCTA CCTTGTTCAT TATCTCATGA GTCACAACAT 318
                        SIMLO7_LEAF  ATGTGGTCTT GCACTTGCTA CCTTGTTCAT TATCTCATGA GTCACAACAT 308
                                             460                  480                  500
gDNA_Solyc09g018830+Solyc09g018840  CTTCGCCGTC TGTTAGCTGA AGAAGAAGCA GTTGTTGGCG ATTGCATTGC 500
                     SIMLO7_FGENESH  CTTCGCCGTC TGTTAGCTGA AGAAGAAGCA GTTGTTGGCG ATTGCATTGC 368
                        SIMLO7_LEAF  CTTCGCCGTC TGTTAGCTGA AGAAGAAGCA GTTGTTGGCG ATTGCATTGC 358
                                                        520                  540
gDNA_Solyc09g018830+Solyc09g018840  TAAAGTAATC AATTTGTTTT ACTTGAATTA TTTTCTTAAG TATCCTGACT 550
                     SIMLO7_FGENESH  TAAA------ ---------- ---------- ---------- ---------- 372
                        SIMLO7_LEAF  TAAA------ ---------- ---------- ---------- ---------- 362
                                             560                  580                  600
gDNA_Solyc09g018830+Solyc09g018840  CTTGAGTTGA ATTCCAGTAT CTCATTCTCG TCATTTTTCG TTAGTTAGAT 600
                     SIMLO7_FGENESH  ---------- ---------- ---------- ---------- ---------- 372
                        SIMLO7_LEAF  ---------- ---------- ---------- ---------- ---------- 362
                                                        620                  640
gDNA_Solyc09g018830+Solyc09g018840  TACTCTACTT CACTACATAC TGACTTCTAA TATCATTATT CCTTTTTTTT 650
                     SIMLO7_FGENESH  ---------- ---------- ---------- ---------- ---------- 372
                        SIMLO7_LEAF  ---------- ---------- ---------- ---------- ---------- 362
                                             660                  680                  700
gDNA_Solyc09g018830+Solyc09g018840  TTGTTTCAGC ATAAGGTACC ATTACTCTCT CTTGAAGCCT TGCATCACCT 700
                     SIMLO7_FGENESH  ---------C ATAAGGTACC ATTACTCTCT CTTGAAGCCT TGCATCACCT 413
                        SIMLO7_LEAF  ---------C ATAAGGTACC ATTACTCTCT CTTGAAGCCT TGCATCACCT 403
                                                        720                  740
gDNA_Solyc09g018830+Solyc09g018840  TCACATTTTC ATCTTTGTCC TTGCAATTGT TCACGTGACT TTCTCTCTTC 750
                     SIMLO7_FGENESH  TCACATTTTC ATCTTTGTCC TTGCAATTGT TCACGTGACT TTCTCTCTTC 463
                        SIMLO7_LEAF  TCACATTTTC ATCTTTGTCC TTGCAATTGT TCACGTGACT TTCTCTCTTC 453
                                             760                  780                  800
gDNA_Solyc09g018830+Solyc09g018840  TGACTATTGT ATTTGGAGGA GCCAAGGTCT CTACTTTATT AACTTGTTCC 800
                     SIMLO7_FGENESH  TGACTATTGT ATTTGGAGGA GCCAAG---- ---------- ---------- 489
                        SIMLO7_LEAF  TGACTATTGT ATTTGGAGGA GCCAAG---- ---------- ---------- 479
                                                        820                  840
gDNA_Solyc09g018830+Solyc09g018840  TCGTTTCTTC TCTTCAACTT TGTTTGTTGT TTGAATTGAG TGTTTGTAAT 850
                     SIMLO7_FGENESH  ---------- ---------- ---------- ---------- ---------- 489
                        SIMLO7_LEAF  ---------- ---------- ---------- ---------- ---------- 479
                                             860                  880                  900
gDNA_Solyc09g018830+Solyc09g018840  TTACTGATGG TTCAGATACG TCAACGGAAG CACTGTGAGG ACAGTATTGC 900
                     SIMLO7_FGENESH  ---------- -----ATACG TCAACGGAAG CACTGTGAGG ACAGTATTGC 524
                        SIMLO7_LEAF  ---------- -----ATACG TCAACGGAAG CACTGTGAGG ACAGTATTGC 514
                                                        920                  940
gDNA_Solyc09g018830+Solyc09g018840  AAAAGCTAAT TATGAGACTG CACAAGGTAT GTATGATTGA TTACCTGTGA 950
                     SIMLO7_FGENESH  AAAAGCTAAT TATGAGACTG CACAAG---- ---------- ---------- 550
                        SIMLO7_LEAF  AAAAGCTAAT TATGAGACTG CACAAG---- ---------- ---------- 540
                                             960                  980                  1,000
gDNA_Solyc09g018830+Solyc09g018840  GGTCTTTTAT CAAACTCTTT GATCCATATC GCCAAAAAAA CAGATATAAT 1000
                     SIMLO7_FGENESH  ---------- ---------- ---------- ---------- ---------- 550
                        SIMLO7_LEAF  ---------- ---------- ---------- ---------- ---------- 540
```

```
                                                          1,020                        1,040
gDNA_Solyc09g018830+Solyc09g018840  TTGATTTATT  TTCTCTATAA  GGAGCTTAAT  TTTACATGAT  AATTGGAGCC  1050
                   SlMLO7_FGENESH   ----------  ----------  ----------  ----------  ----------  550
                        SlMLO7_LEAF ----------  ----------  ----------  ----------  ----------  540
                                             1,060                        1,080                   1,100
gDNA_Solyc09g018830+Solyc09g018840  GCTTAACACT  GTTCTAAATG  ACGATTAGCA  AAGAAGAAAT  AAAGTAAATG  1100
                   SlMLO7_FGENESH   ----------  ----------  ----------  ----------  ----------  550
                        SlMLO7_LEAF ----------  ----------  ----------  ----------  ----------  540
                                                          1,120                        1,140
gDNA_Solyc09g018830+Solyc09g018840  ACCTAATGAA  AAGCTTATGT  GGAAAACTTT  AATTTTACAT  AACAATTGGG  1150
                   SlMLO7_FGENESH   ----------  ----------  ----------  ----------  ----------  550
                        SlMLO7_LEAF ----------  ----------  ----------  ----------  ----------  540
                                             1,160                        1,180                   1,200
gDNA_Solyc09g018830+Solyc09g018840  AACCGCTTTA  CACGGTTCGA  AATGATAATA  ATCAAAGATG  AAAGAAATTA  1200
                   SlMLO7_FGENESH   ----------  ----------  ----------  ----------  ----------  550
                        SlMLO7_LEAF ----------  ----------  ----------  ----------  ----------  540
                                                          1,220                        1,240
gDNA_Solyc09g018830+Solyc09g018840  AATGGCCGAT  ATAAAAGTTT  ATGTCGTCCC  TGTAGTTAAT  GTGCTCCCTA  1250
                   SlMLO7_FGENESH   ----------  ----------  ----------  ----------  ----------  550
                        SlMLO7_LEAF ----------  ----------  ----------  ----------  ----------  540
                                             1,260                        1,280                   1,300
gDNA_Solyc09g018830+Solyc09g018840  TAAAACTAGT  TACTATCTTA  ACCAAGTCTT  TAAGGTAGTG  AACTGTTCTC  1300
                   SlMLO7_FGENESH   ----------  ----------  ----------  ----------  ----------  550
                        SlMLO7_LEAF ----------  ----------  ----------  ----------  ----------  540
                                                          1,320                        1,340
gDNA_Solyc09g018830+Solyc09g018840  ATACTGCAAC  CAATGAGAGG  AAAGATAAGT  AAGAAGCAAT  ATGGAGTTAC  1350
                   SlMLO7_FGENESH   ----------  ----------  ----------  ----------  ----------  550
                        SlMLO7_LEAF ----------  ----------  ----------  ----------  ----------  540
                                             1,360                        1,380                   1,400
gDNA_Solyc09g018830+Solyc09g018840  TCCCCTCATG  CATTATATCT  AGATCAAGCT  GATCTTAAAA  CTAAAATAGT  1400
                   SlMLO7_FGENESH   ----------  ----------  ----------  ----------  ----------  550
                        SlMLO7_LEAF ----------  ----------  ----------  ----------  ----------  540
                                                          1,420                        1,440
gDNA_Solyc09g018830+Solyc09g018840  TTGATGCTTT  CTTTTGCAGT  TTTAAAACCA  CGGGTTACAC  ATGTCCAGCA  1450
                   SlMLO7_FGENESH   ----------  ---------T  TTTAAAACCA  CGGGTTACAC  ATGTCCAGCA  581
                        SlMLO7_LEAF ----------  ---------T  TTTAAAACCA  CGGGTTACAC  ATGTCCAGCA  571
                                             1,460                        1,480                   1,500
gDNA_Solyc09g018830+Solyc09g018840  ACACGATTTC  ATCAGGGATC  GATTTGTGGG  TATGGGCAAA  TGCTCAGCCA  1500
                   SlMLO7_FGENESH   ACACGATTTC  ATCAGGGATC  GATTTGTGGG  TATGGGCAAA  TGCTCAGCCA  631
                        SlMLO7_LEAF ACACGATTTC  ATCAGGGATC  GATTTGTGGG  TATGGGCAAA  TGCTCAGCCA  621
                                                          1,520                        1,540
gDNA_Solyc09g018830+Solyc09g018840  TTTTTGGTTG  GTTGGTAAGC  AGTTTCTTTC  CTCAATAAGC  AATCATTCTT  1550
                   SlMLO7_FGENESH   TTTTTGGTTG  GTT-------  ----------  ----------  ----------  644
                        SlMLO7_LEAF TTTTTGGTTG  GTT-------  ----------  ----------  ----------  634
                                             1,560                        1,580                   1,600
gDNA_Solyc09g018830+Solyc09g018840  TCCCCATCAT  ATATTTCTCC  TATGTATGGT  AAAACTTACC  TTGGGAACTG  1600
                   SlMLO7_FGENESH   ----------  ----------  ----------  ----------  ----------  644
                        SlMLO7_LEAF ----------  ----------  ----------  ----------  ----------  634
                                                          1,620                        1,640
gDNA_Solyc09g018830+Solyc09g018840  CTCTTACAAC  ACATTTATGT  GGAAAGAAAA  GAAAGATCAT  TAAATTTAAG  1650
                   SlMLO7_FGENESH   ----------  ----------  ----------  ----------  ----------  644
                        SlMLO7_LEAF ----------  ----------  ----------  ----------  ----------  634
                                             1,660                        1,680                   1,700
gDNA_Solyc09g018830+Solyc09g018840  CTATTAAAGA  TGGGGAAGGT  GCATACGCTT  TCAAATGACA  GGGGAATATG  1700
                   SlMLO7_FGENESH   ----------  ----------  ----------  ----------  ----------  644
                        SlMLO7_LEAF ----------  ----------  ----------  ----------  ----------  634
                                                          1,720                        1,740
gDNA_Solyc09g018830+Solyc09g018840  TGATGGACAT  ATATGATCAC  TTTCTTTTAT  CCGTTTATGT  TGTGAGAAAG  1750
                   SlMLO7_FGENESH   ----------  ----------  ----------  ----------  ----------  644
                        SlMLO7_LEAF ----------  ----------  ----------  ----------  ----------  634
                                             1,760                        1,780                   1,800
gDNA_Solyc09g018830+Solyc09g018840  GCAAATGTTG  GATCCTGTCT  CCAGTAACTC  CATCATATAT  CTAGTTTAGC  1800
                   SlMLO7_FGENESH   ----------  ----------  ----------  ----------  ----------  644
                        SlMLO7_LEAF ----------  ----------  ----------  ----------  ----------  634
                                                          1,820                        1,840
gDNA_Solyc09g018830+Solyc09g018840  TAATATATGG  TTCACTACTT  AAGTGCGTTG  AAAGAATTTA  TATTATTAGC  1850
                   SlMLO7_FGENESH   ----------  ----------  ----------  ----------  ----------  644
                        SlMLO7_LEAF ----------  ----------  ----------  ----------  ----------  634
                                             1,860                        1,880                   1,900
gDNA_Solyc09g018830+Solyc09g018840  AAGTTTTATT  TACTGTTGTG  TTAGGGCCCT  GTTACCTGAG  AATTCTATAC  1900
                   SlMLO7_FGENESH   ----------  ----------  ----------  ----------  ----------  644
                        SlMLO7_LEAF ----------  ----------  ----------  ----------  ----------  634
                                                          1,920                        1,940
gDNA_Solyc09g018830+Solyc09g018840  AAGCAAGTAC  TAGTTCTTAT  TCTCAACAAA  TATGATAAGT  TGGCACTAAC  1950
                   SlMLO7_FGENESH   ----------  ----------  ----------  ----------  ----------  644
                        SlMLO7_LEAF ----------  ----------  ----------  ----------  ----------  634
                                             1,960                        1,980                   2,000
gDNA_Solyc09g018830+Solyc09g018840  AAAAATTTGA  AAAACATGCT  GAACAAACAT  CAATTTATTT  ATGAGTAAGA  2000
                   SlMLO7_FGENESH   ----------  ----------  ----------  ----------  ----------  644
                        SlMLO7_LEAF ----------  ----------  ----------  ----------  ----------  634
```

```
                                            2,020                           2,040
                                              |                               |
gDNA_Solyc09g018830+Solyc09g018840  CAGAAAATGT AGAAATGGGG AATTTATAGT AGAGTAATCA CGTGGTTTTA 2050
                    SIMLO7_FGENESH  ---------- ---------- ---------- ---------- ---------- 644
                       SIMLO7_LEAF  ---------- ---------- ---------- ---------- ---------- 634
                               2,060                           2,080                     2,100
                                 |                               |                         |
gDNA_Solyc09g018830+Solyc09g018840  TAGTGCCTTT ACATTAACAT AAGCATCTCA TACTTTTATT TGGTTTCCAC 2100
                    SIMLO7_FGENESH  ---------- ---------- ---------- ---------- ---------- 644
                       SIMLO7_LEAF  ---------- ---------- ---------- ---------- ---------- 634
                                            2,120                           2,140
                                              |                               |
gDNA_Solyc09g018830+Solyc09g018840  AAAACTTGCA GCACTCTTTC TTTAAGCAAT TTTATGGTTC TGTCAGCAAG 2150
                    SIMLO7_FGENESH  ---------- GCACTCTTTC TTTAAGCAAT TTTATGGTTC TGTCAGCAAG 684
                       SIMLO7_LEAF  ---------- G--------- ---------- ---------- ---------- 635
                               2,160                           2,180                     2,200
                                 |                               |                         |
gDNA_Solyc09g018830+Solyc09g018840  TTAGATTATG CTACTCTGTG TTTGGGGTTC ATTATGGTAA CTTCACTACG 2200
                    SIMLO7_FGENESH  TTAGATTATG CTACTCTGTG TTTGGGGTTC ATTATG---- ---------- 720
                       SIMLO7_LEAF  ---------- ---------- ---------- ---------- ---------- 635
                                            2,220                           2,240
                                              |                               |
gDNA_Solyc09g018830+Solyc09g018840  TTACTTGTCT GAGATAATTG TTTAGTGTTT TCCCATCTTT AGGTGTTTAA 2250
                    SIMLO7_FGENESH  ---------- ---------- ---------- ---------- ---------- 720
                       SIMLO7_LEAF  ---------- ---------- ---------- ---------- ---------- 635
                               2,260                           2,280                     2,300
                                 |                               |                         |
gDNA_Solyc09g018830+Solyc09g018840  TTGCTCATTT AGTATTAAGT TTCTTCTCTT TGGCAGACAC ATTGCAAACA 2300
                    SIMLO7_FGENESH  ---------- ---------- ---------- ------ACAC ATTGCAAACA 734
                       SIMLO7_LEAF  ---------- ---------- ---------- ------ACAC ATTGCAAACA 649
                                            2,320                           2,340
                                              |                               |
gDNA_Solyc09g018830+Solyc09g018840  AAACCCAAAA TTTAATTTTC ACAAGTACAT GATTCGTGCT CTGGAAGATG 2350
                    SIMLO7_FGENESH  AAACCCAAAA TTTAATTTTC ACAAGTACAT GATTCGTGCT CTGGAAGATG 784
                       SIMLO7_LEAF  AAACCCAAAA TTTAATTTTC ACAAGTACAT GATTCGTGCT CTGGAAGATG 699
                               2,360                           2,380                     2,400
                                 |                               |                         |
gDNA_Solyc09g018830+Solyc09g018840  ATTTTAAGAC TGTCGTTGGT ATCAGGTTAG TGTCCATTCT CCTTACCTCC 2400
                    SIMLO7_FGENESH  ATTTTAAGAC TGTCGTTGGT ATCAG----- ---------- ---------- 809
                       SIMLO7_LEAF  ATTTTAAGAC TGTCGTTGGT ATCAG----- ---------- ---------- 724
                                            2,420                           2,440
                                              |                               |
gDNA_Solyc09g018830+Solyc09g018840  TCTGCCACAT CGGAGATTTC TCTATGAGAA ATATGAATCG GAGTTTGAAG 2450
                    SIMLO7_FGENESH  ---------- ---------- ---------- ---------- ---------- 809
                       SIMLO7_LEAF  ---------- ---------- ---------- ---------- ---------- 724
                               2,460                           2,480                     2,500
                                 |                               |                         |
gDNA_Solyc09g018830+Solyc09g018840  AAGGTGAGGG ATAAGGAGCC CATCCCACCG GGAAGAATTT GCGGTGTTTT 2500
                    SIMLO7_FGENESH  ---------- ---------- ---------- ---------- ---------- 809
                       SIMLO7_LEAF  ---------- ---------- ---------- ---------- ---------- 724
                                            2,520                           2,540
                                              |                               |
gDNA_Solyc09g018830+Solyc09g018840  TCTTACTTGT TGCCATTTTC CTGTGTTTTT TTTTGCAGTT AGTATCTCTG 2550
                    SIMLO7_FGENESH  ---------- ---------- ---------- ---------- ---------- 809
                       SIMLO7_LEAF  ---------- ---------- ---------- --------TT AGTATCTCTG 736
                               2,560                           2,580                     2,600
                                 |                               |                         |
gDNA_Solyc09g018830+Solyc09g018840  GGTATTTGTT GTCCTCTTCT TGTTTCTGAA TGTTAATGGT ATGCCAACTC 2600
                    SIMLO7_FGENESH  ---------- ---------- ---------- ----AACAAA ---------- 815
                       SIMLO7_LEAF  GGTATTTGTT GTCCTCTTCT TGTTTCTGAA TGTTAATGGT ---------- 776
                                            2,620                           2,640
                                              |                               |
gDNA_Solyc09g018830+Solyc09g018840  AAACTTGGAA TAGTCATTTT TACCTTGCAG CTGCTTTGAA ATGTTTCAAT 2650
                    SIMLO7_FGENESH  -----TGAAA AA-------- ---------- ---------- ---------- 822
                       SIMLO7_LEAF  -----TGGCA TACATACTTC T--------- ---------- ---------- 792
                               2,660                           2,680                     2,700
                                 |                               |                         |
gDNA_Solyc09g018830+Solyc09g018840  GGTATACTTA TGCAGAACAA ATGAAAAAGG GTTGTCTTGG CAAGTAATAA 2700
                    SIMLO7_FGENESH  ---------- ---------- --------GG GTTGTCTTGG C--------A 836
                       SIMLO7_LEAF  ---------- ---------- --------GG ATTGCGTTCA TTCCTTTTGA 814
                                            2,720                           2,740
                                              |                               |
gDNA_Solyc09g018830+Solyc09g018840  TTGGCTACCA GCAATCTTAG TGTTGTACAT GCTATAGTTG TAATTTGATA 2750
                    SIMLO7_FGENESH  AC-------- ---------- --TTCTGATT GCGGTTGGCA CGAAGTTGGA 866
                       SIMLO7_LEAF  TC-------- ---------- --TTCTGATT GCGGTTGGCA CGAAGTTGGA 844
                               2,760                           2,780                     2,800
                                 |                               |                         |
gDNA_Solyc09g018830+Solyc09g018840  ACATCTCCTT ATGCATCCCC AAGTTATGAT TCAATAAAGC CACGTGTTAG 2800
                    SIMLO7_FGENESH  GCATGTAATT ATACAGTT-- AGCTCATGAG GTAGCAGAGA AACATGTCGC 914
                       SIMLO7_LEAF  GCATGTAATT ATACAGTT-- AGCTCATGAG GTAGCAGAGA AACATGTCGC 892
                                            2,820                           2,840
                                              |                               |
gDNA_Solyc09g018830+Solyc09g018840  AATTCT-GTT GGTTTTGTGT TACTATTTTC ATTTTTCAGC ACTCTTTTAG 2849
                    SIMLO7_FGENESH  GATAGAAGGT GAATTGGTGG TCCAACCTTC T--------- ---------G 946
                       SIMLO7_LEAF  GATAGAAGGT GAATTGGTGG TCCAACCTTC T--------- ---------G 924
                               2,860                           2,880                     2,900
                                 |                               |                         |
gDNA_Solyc09g018830+Solyc09g018840  ATGATTTATT CAGACACTGC TTTTAACAGT GTTTCCTGCA TAACTTTCAC 2899
                    SIMLO7_FGENESH  ATGATCACTT C--------- ---------- ---------- ---------- 957
                       SIMLO7_LEAF  ATGATCACTT C--------- ---------- ---------- ---------- 935
                                            2,920                           2,940
                                              |                               |
gDNA_Solyc09g018830+Solyc09g018840  TTGGCTTTGA TATTTTACAA GTTTCATTGA TTTTTCAGTT TGGTCATTTG 2949
                    SIMLO7_FGENESH  -TGGTTCAAG CGCCCTCAAA TTGTCCTTGT CTT------- ---------- 989
                       SIMLO7_LEAF  -TGGTTCAAG CGCCCTCAAA TTGTCCTTGT CTT------- ---------- 967
                               2,960                           2,980                     3,000
                                 |                               |                         |
gDNA_Solyc09g018830+Solyc09g018840  GTAGTGGTTT ATATATGAAA GCTGATTAAT TCATTTTATA CTCTCCAGGT 2999
                    SIMLO7_FGENESH  ---------- ---------- -------GAT ACACTTTATC CTCTTCCA-- 1010
                       SIMLO7_LEAF  ---------- ---------- -------GAT ACACTTTATC CTCTTCCA-- 988
```

```
                                            3,020                3,040
gDNA_Solyc09g018830+Solyc09g018840  TGGCATACAT ACTTCTG-GA TTGCGTTCAT TCCTTTTGAT GTGAGTTGTA 3048
                    SIMLO7_FGENESH  ------AAAT GCTTTTGAGA TAGCATTCTT TTTTCAGGAT ATTGG----- 1049
                       SIMLO7_LEAF  ------AAAT GCTTTTGAGA TAGCATTCTT TTTTCAGGAT ATTGGTGCAA 1032
                                 3,060                3,080                3,100
gDNA_Solyc09g018830+Solyc09g018840  TCTTTACTAA GCCTTCTGCT AGATCTAATT CAAATTTTAA GAATAGCCAT 3098
                    SIMLO7_FGENESH  ---------- ---------- ---------- ---------- ---------- 1049
                       SIMLO7_LEAF  TATGGGTTTG ATTCTTGCAT AATGGGACAA GTTGGTTTCA TTGTTCCACG 1082
                                            3,120                3,140
gDNA_Solyc09g018830+Solyc09g018840  TCAAACAAAA CAATCACTCC CTCTGTCCCA GTTTATGTGA CACACTTTTT 3148
                    SIMLO7_FGENESH  ---------- ---------- ---------- ---------- --CACGTTGC 1057
                       SIMLO7_LEAF  ACTTGTTATA GGGGTAATAA TTCAAGTGTT ATGAAGCTAT AGCACGTTGC 1132
                                 3,160                3,180                3,200
gDNA_Solyc09g018830+Solyc09g018840  CTGTTTAGTT TGTCGCCAAA AAAATG---A CACGTTTCTT TATTTGTCAA 3195
                    SIMLO7_FGENESH  CACTTTATGC TCTTGTTACA CAGATGGGTA CCCATTTC-- ---------- 1095
                       SIMLO7_LEAF  CACTTTATGC TCTTGTTACA CAGATGGGTA CCCATTTC-- ---------- 1170
                                            3,220                3,240
gDNA_Solyc09g018830+Solyc09g018840  ATGTTTAGTG GCAGAATCAA TATTTTAACT ATGTTGGATC CCACTTATTT 3245
                    SIMLO7_FGENESH  ---------- AAGAAGTCAA TATTTGAT-- ------GAGC CCATTCA--- 1124
                       SIMLO7_LEAF  ---------- AAGAAGTCAA TATTTGAT-- ------GAGC CCATTCA--- 1199
                                 3,260                3,280                3,300
gDNA_Solyc09g018830+Solyc09g018840  AGTAAAAAAG AAAGTCCACA AAAGTGTACC ATTCTATTTA AAAGTCATTT 3295
                    SIMLO7_FGENESH  --------AG TTGGTCTTCT TGGTTGGGC- ------TTTG AAAGCGA--- 1156
                       SIMLO7_LEAF  --------AG TTGGTCTTCT TGGTTGGGC- ------TTTG AAAGCGA--- 1231
                                            3,320                3,340
gDNA_Solyc09g018830+Solyc09g018840  AATTTAAGGG TAGCCTGGAG CATTTCTAGA TTCCTATCAA CCTTCTCATG 3345
                    SIMLO7_FGENESH  AAATGAAGAG GGGACTCAAG ---------- ---------- ---------- 1176
                       SIMLO7_LEAF  AAATGAAGAG GGGACTCAAG ---------- ---------- ---------- 1251
                                 3,360                3,380                3,400
gDNA_Solyc09g018830+Solyc09g018840  AGTTAAGATG CATGTTGAAT GTGGGTGTTT CCTTTCTCAC ACATTCAATT 3395
                    SIMLO7_FGENESH  -GCTGCTACA GATGCTGGAA CT-------- ---------- ----TCAACT 1203
                       SIMLO7_LEAF  -GCTGCTACA GATGCTGGAA CT-------- ---------- ----TCAACT 1278
                                            3,420                3,440
gDNA_Solyc09g018830+Solyc09g018840  ACTTTTCTTT CTTAATTACT CTTAATATTT TTTTTAAACA CGAGGCTCCT 3445
                    SIMLO7_FGENESH  ---------- ---------- ---------- ---------- -GAGGGTTCT 1212
                       SIMLO7_LEAF  ---------- ---------- ---------- ---------- -GAGGGTTCT 1287
                                 3,460                3,480                3,500
gDNA_Solyc09g018830+Solyc09g018840  TTAGCAACAA CCTCATCTTC TTCCACTTAA ACTTCTTTTT TTTTGGTTTA 3495
                    SIMLO7_FGENESH  ACAGTAA--- ---------- ---------- ---------- ---------- 1219
                       SIMLO7_LEAF  ACAGTAA--- ---------- ---------- ---------- ---------- 1294
                                            3,520                3,540
gDNA_Solyc09g018830+Solyc09g018840  TATCAGCTTC TGATTGCGGT TGGCACGAAG TTGGAGCATG TAATTATACA 3545
                    SIMLO7_FGENESH  ---------- -GATTACAGA TGTCA----- ---------G CAATCAACCA 1244
                       SIMLO7_LEAF  ---------- -GATTACAGA TGTCA----- ---------G CAATCAACCA 1319
                                 3,560                3,580                3,600
gDNA_Solyc09g018830+Solyc09g018840  GTTAGCTCAT GAGGTAGCAG AGAAACATGT CGCGATAGAA GGTGAATTGG 3595
                    SIMLO7_FGENESH  G--------- --------AA GGAAACAA-- ---------- ---------- 1255
                       SIMLO7_LEAF  G--------- --------AA GGAA-CAC-- ---------- ---------- 1329
                                            3,620                3,640
gDNA_Solyc09g018830+Solyc09g018840  TGGTCCAACC TTCTGATGAT CACTTCTGGT TCAAGCGCCC TCAAATTGTC 3645
                    SIMLO7_FGENESH  ---CCCATCT TAATTGGGAA CACAATTATA ATCAAC---- ---------- 1288
                       SIMLO7_LEAF  ---CCCATCT ---------- ---------- ---------- ---------- 1336
                                 3,660
gDNA_Solyc09g018830+Solyc09g018840  CTTGTCTTGA TACAC 3660
                    SIMLO7_FGENESH  ---------- TATAG 1293
                       SIMLO7_LEAF  ---------- ---AA» 1338
```

```
                                                        20                            40
                                                         |                             |
gDNA_Solyc11g069220  ATGGCGGGAG GAGGAGGAGG AAGATCGTTG GAGCAAACGC CGACGTGGGC 50
cDNA_Solyc11g069220  ATGGCGGGAG GAGGAGGAGG AAGATCGTTG GAGCAAACGC CGACGTGGGC 50
        SIMLO8_LEAF  ATGGCGGGAG GAGGAGGAGG AAGATCGTTG GAGCAAACGC CGACGTGGGC 50
                                     60                            80                           100
                                      |                             |                             |
gDNA_Solyc11g069220  GGTTGCCCTA GTTTGTTTTG CATTGGTTGC CATTTCCATT GTAATAGAGC 100
cDNA_Solyc11g069220  GGTTGCCCTA GTTTGTTTTG CATTGGTTGC CATTTCCATT GTAATAGAGC 100
        SIMLO8_LEAF  GGTTGCCCTA GTTTGTTTTG CATTGGTTGC CATTTCCATT GTAATAGAGC 100
                                                       120                           140
                                                         |                             |
gDNA_Solyc11g069220  TCATCATCCA TCTTATTGGC AAGGTAGGCC ACTAACCATG CAATATTTTG 150
cDNA_Solyc11g069220  TCATCATCCA TCTTATTGGC AAG------- ---------- ---------- 123
        SIMLO8_LEAF  TCATCATCCA TCTTATTGGC AAG------- ---------- ---------- 123
                                    160                           180                           200
                                      |                             |                             |
gDNA_Solyc11g069220  ATAGAGTTTA AGTTATATAT ATATATACGT ATTATTATTA TTATGTATTA 200
cDNA_Solyc11g069220  ---------- ---------- ---------- ---------- ---------- 123
        SIMLO8_LEAF  ---------- ---------- ---------- ---------- ---------- 123
                                                       220                           240
                                                         |                             |
gDNA_Solyc11g069220  TTCAAAAGAT ATCTACTAAG TCTGATCATT GAATTATGAG ATTTATAATT 250
cDNA_Solyc11g069220  ---------- ---------- ---------- ---------- ---------- 123
        SIMLO8_LEAF  ---------- ---------- ---------- ---------- ---------- 123
                                    260                           280                           300
                                      |                             |                             |
gDNA_Solyc11g069220  TTATAATTAA TATATTTTAC TTGTTCCAAC AAGTGAACAT GTCTGTTTAT 300
cDNA_Solyc11g069220  ---------- ---------- ---------- ---------- ---------- 123
        SIMLO8_LEAF  ---------- ---------- ---------- ---------- ---------- 123
                                                       320                           340
                                                         |                             |
gDNA_Solyc11g069220  ATAAAAAATT CTTACACTAA AAAGGTAAAA AGTATTTATA TAATTGGGTC 350
cDNA_Solyc11g069220  ---------- ---------- ---------- ---------- ---------- 123
        SIMLO8_LEAF  ---------- ---------- ---------- ---------- ---------- 123
                                    360                           380                           400
                                      |                             |                             |
gDNA_Solyc11g069220  ATAAATTCAT GAATATTTAT AGTGTACGAT ATAACATAGA TATATTTCAA 400
cDNA_Solyc11g069220  ---------- ---------- ---------- ---------- ---------- 123
        SIMLO8_LEAF  ---------- ---------- ---------- ---------- ---------- 123
                                                       420                           440
                                                         |                             |
gDNA_Solyc11g069220  TAAATTCACA AATATAATTT TATCATTAAT ATTCTTTTGC CCTTGCATGT 450
cDNA_Solyc11g069220  ---------- ---------- ---------- ---------- ---------- 123
        SIMLO8_LEAF  ---------- ---------- ---------- ---------- ---------- 123
                                    460                           480                           500
                                      |                             |                             |
gDNA_Solyc11g069220  ATAGTGGTTG AAGTCTAAAC ATAAAAGAGC ATTATATGAA GCACTTGAGA 500
cDNA_Solyc11g069220  ----TGGTTG AAGTCTAAAC ATAAAAGAGC ATTATATGAA GCACTTGAGA 169
        SIMLO8_LEAF  ----TGGTTG AAGTCTAAAC ATAAAAGAGC ATTATATGAA GCACTTGAGA 169
                                                       520                           540
                                                         |                             |
gDNA_Solyc11g069220  AGATAAAAGC AGGTACACTT TAAATTCGTA TTATTCGGAT CTTTCAAATA 550
cDNA_Solyc11g069220  AGATAAAAGC AG-------- ---------- ---------- ---------- 181
        SIMLO8_LEAF  AGATAAAAGC AG-------- ---------- ---------- ---------- 181
                                    560                           580                           600
                                      |                             |                             |
gDNA_Solyc11g069220  TATTGTTGTT GTTGTTCTGT TATTATCTGA TTCTTCATAA TTATATAACT 600
cDNA_Solyc11g069220  ---------- ---------- ---------- ---------- ---------- 181
        SIMLO8_LEAF  ---------- ---------- ---------- ---------- ---------- 181
                                                       620                           640
                                                         |                             |
gDNA_Solyc11g069220  TTTGAAGGAT TGATCTGATC AATATACACC AAATGATATT TTTATAATAC 650
cDNA_Solyc11g069220  ---------- ---------- ---------- ---------- ---------- 181
        SIMLO8_LEAF  ---------- ---------- ---------- ---------- ---------- 181
                                    660                           680                           700
                                      |                             |                             |
gDNA_Solyc11g069220  ACTTTAAATT CATATTATCC GGACCTTTCA AATATATTGT CGTTGTTATT 700
cDNA_Solyc11g069220  ---------- ---------- ---------- ---------- ---------- 181
        SIMLO8_LEAF  ---------- ---------- ---------- ---------- ---------- 181
                                                       720                           740
                                                         |                             |
gDNA_Solyc11g069220  CTGTTATTAT TTGATTCTTC ATAATTATAT AACTTTTGAA GGATTGATCT 750
cDNA_Solyc11g069220  ---------- ---------- ---------- ---------- ---------- 181
        SIMLO8_LEAF  ---------- ---------- ---------- ---------- ---------- 181
                                    760                           780                           800
                                      |                             |                             |
gDNA_Solyc11g069220  GATCAATATA CACTAAATGA TATTTTTGTA ATACATTTTA AATTCATATT 800
cDNA_Solyc11g069220  ---------- ---------- ---------- ---------- ---------- 181
        SIMLO8_LEAF  ---------- ---------- ---------- ---------- ---------- 181
                                                       820                           840
                                                         |                             |
gDNA_Solyc11g069220  GTTCGAATCT TTCAAATATA TTGTCATTTT TGTTCTGTTA TTATCTGATT 850
cDNA_Solyc11g069220  ---------- ---------- ---------- ---------- ---------- 181
        SIMLO8_LEAF  ---------- ---------- ---------- ---------- ---------- 181
                                    860                           880                           900
                                      |                             |                             |
gDNA_Solyc11g069220  CTTCATAATT TACATAACTT TTGAAGGATT GGTCTGATCA ACATACACTG 900
cDNA_Solyc11g069220  ---------- ---------- ---------- ---------- ---------- 181
        SIMLO8_LEAF  ---------- ---------- ---------- ---------- ---------- 181
                                                       920                           940
                                                         |                             |
gDNA_Solyc11g069220  CAACGATATT TTTGTAGAAT TTGAACAACA TACATATACT CGTTAATTTT 950
cDNA_Solyc11g069220  ---------- ---------- ---------- ---------- ---------- 181
        SIMLO8_LEAF  ---------- ---------- ---------- ---------- ---------- 181
                                    960                           980                         1,000
                                      |                             |                             |
gDNA_Solyc11g069220  CTAACGAACA TATTTTTATT TATTTTCACT AATATTTTAT TTTTAATTTT 1000
cDNA_Solyc11g069220  ---------- ---------- ---------- ---------- ---------- 181
        SIMLO8_LEAF  ---------- ---------- ---------- ---------- ---------- 181
```

```
                                                  1,020               1,040
gDNA_Solyc11g069220  TTTTATAGAG TTAATGTTGT TGGGATTTAT ATCCTTACTA TTAACAGTAG 1050
cDNA_Solyc11g069220  ---------- ---------- ---------- ---------- -------TAG 184
        SIMLO8_LEAF  --------AG TTAATGTTGT TGGGATTTAT ATCCTTACTA TTAACAGTAG 223
                              1,060               1,080               1,100
gDNA_Solyc11g069220  GGCAAGATCC AATTTCAAAT ATTTGTGTAT CTGAAAAAAT TGCAAGTACG 1100
cDNA_Solyc11g069220  GGCAAGATCC AATTTCAAAT ATTTGTGTAT CTGAAAAAAT TGCAAGTACG 234
        SIMLO8_LEAF  GGCAAGATCC AATTTCAAAT ATTTGTGTAT CTGAAAAAAT TGCAAGTACG 273
                                        1,120               1,140
gDNA_Solyc11g069220  TGGCATCCAT GTAGTAAACA AAAAGAAGCT GAAATGAACA AATATATTTC 1150
cDNA_Solyc11g069220  TGGCATCCAT GTAGTAAACA AAAAGAAGCT GAAATGAACA AATATATTTC 284
        SIMLO8_LEAF  TGGCATCCAT GTAGTAAACA AAAAGAAGCT GAAATGAACA AATATATTTC 323
                              1,160               1,180               1,200
gDNA_Solyc11g069220  CGGTGACTTA GAGGGTCATC GCCGGCGACT TTTCACGGCT GACGATGGCG 1200
cDNA_Solyc11g069220  CGGTGACTTA GAGGGTCATC GCCGGCGACT TTTCACGGCT GACGATGGCG 334
        SIMLO8_LEAF  CGGTGACTTA GAGGGTCATC GCCGGCGACT TTTCACGGCT GACGATGGCG 373
                                        1,220               1,240
gDNA_Solyc11g069220  GAGTCCGGCG AGTTTTGGCG GCTGCCGGAA CTGACAAATG TGCAGACAAG 1250
cDNA_Solyc11g069220  GAGTCCGGCG AGTTTTGGCG GCTGCCGGAA CTGACAAATG TGCAGACAAG 384
        SIMLO8_LEAF  GAGTCCGGCG AGTTTTGGCG GCTGCCGGAA CTGACAAATG TGCAGACAAG 423
                              1,260               1,280               1,300
gDNA_Solyc11g069220  GCATGTTATT CGTATTTATT ATTTTTTTCT TAATAAAAAT GATAGTTAAT 1300
cDNA_Solyc11g069220  G--------- ---------- ---------- ---------- ---------- 385
        SIMLO8_LEAF  G--------- ---------- ---------- ---------- ---------- 424
                                        1,320               1,340
gDNA_Solyc11g069220  ATTAAACGAA GAAAATGTTA TATATACTCT AGAATAATAA AATATATTTA 1350
cDNA_Solyc11g069220  ---------- ---------- ---------- ---------- ---------- 385
        SIMLO8_LEAF  ---------- ---------- ---------- ---------- ---------- 424
                              1,360               1,380               1,400
gDNA_Solyc11g069220  TATATTTTAT AAGTAAATAA CATACTATAC TGTAAATTAA AAAAATACTT 1400
cDNA_Solyc11g069220  ---------- ---------- ---------- ---------- ---------- 385
        SIMLO8_LEAF  ---------- ---------- ---------- ---------- ---------- 424
                                        1,420               1,440
gDNA_Solyc11g069220  ATAAGCTAAT TTGATCGACT TAAAATTAGC CAAAACGCAC GTTAACTTTA 1450
cDNA_Solyc11g069220  ---------- ---------- ---------- ---------- ---------- 385
        SIMLO8_LEAF  ---------- ---------- ---------- ---------- ---------- 424
                              1,460               1,480               1,500
gDNA_Solyc11g069220  TAAGTCAATG CCAATGCTTA TAAATCAAAA TATGTCAAAA GTCATAACTT 1500
cDNA_Solyc11g069220  ---------- ---------- ---------- ---------- ---------- 385
        SIMLO8_LEAF  ---------- ---------- ---------- ---------- ---------- 424
                                        1,520               1,540
gDNA_Solyc11g069220  AGTTATTACT TATATATTAC TCCTAATTAA TTTAAGCCTA TAGTATTAAG 1550
cDNA_Solyc11g069220  ---------- ---------- ---------- ---------- ---------- 385
        SIMLO8_LEAF  ---------- ---------- ---------- ---------- ---------- 424
                              1,560               1,580               1,600
gDNA_Solyc11g069220  TATTTTAAGT TTGACTATTT TTATTTTATT ACTTTATCCT TATTTATTTG 1600
cDNA_Solyc11g069220  ---------- ---------- ---------- ---------- ---------- 385
        SIMLO8_LEAF  ---------- ---------- ---------- ---------- ---------- 424
                                        1,620               1,640
gDNA_Solyc11g069220  AAATATTTTT AGCTAAACAA AAATCTTAAC TTTCTCTTCT TTTGTATATA 1650
cDNA_Solyc11g069220  ---------- ---------- ---------- ---------- ---------- 385
        SIMLO8_LEAF  ---------- ---------- ---------- ---------- ---------- 424
                              1,660               1,680               1,700
gDNA_Solyc11g069220  TGTTTTTTGT TTTTTCTAAA ATAAATAATA AAAAAATAAA ATTACCAGTT 1700
cDNA_Solyc11g069220  ---------- ---------- ---------- ---------- ---------- 385
        SIMLO8_LEAF  ---------- ---------- ---------- ---------- ---------- 424
                                        1,720               1,740
gDNA_Solyc11g069220  TAACATAATG ATAAATTGAT ACTTTTAAAT TTATCAAGAT TTTTTCTTAA 1750
cDNA_Solyc11g069220  ---------- ---------- ---------- ---------- ---------- 385
        SIMLO8_LEAF  ---------- ---------- ---------- ---------- ---------- 424
                              1,760               1,780               1,800
gDNA_Solyc11g069220  CAAACATGCC AATTGTTCAT ATTTATTTAT AAAATTAATT TCAACGCTTG 1800
cDNA_Solyc11g069220  ---------- ---------- ---------- ---------- ---------- 385
        SIMLO8_LEAF  ---------- ---------- ---------- ---------- ---------- 424
                                        1,820               1,840
gDNA_Solyc11g069220  GATATATTTT TTTATATTTA AATTTATCAA CTATTTACGA TCATCTAATC 1850
cDNA_Solyc11g069220  ---------- ---------- ---------- ---------- ---------- 385
        SIMLO8_LEAF  ---------- ---------- ---------- ---------- ---------- 424
                              1,860               1,880               1,900
gDNA_Solyc11g069220  AAAACGAGGC GTGAATTAAT TTTTTTTTTT TGGATTTGTA ATTTGCAGGG 1900
cDNA_Solyc11g069220  ---------- ---------- ---------- ---------- ---------G 386
        SIMLO8_LEAF  ---------- ---------- ---------- ---------- ---------G 425
                                        1,920               1,940
gDNA_Solyc11g069220  AAAAGTAGCA TTTGTGTCTG CTGATGGTAT TCATCAATTA CATATTTTCA 1950
cDNA_Solyc11g069220  AAAAGTAGCA TTTGTGTCTG CTGATGGTAT TCATCAATTA CATATTTTCA 436
        SIMLO8_LEAF  AAAAGTAGCA TTTGTGTCTG CTGATGGTAT TCATCAATTA CATATTTTCA 475
                              1,960               1,980               2,000
gDNA_Solyc11g069220  TTTTTGTGCT GGCTATTTTT CATGTATTTT ATTGTGTTAC CACATTGGCA 2000
cDNA_Solyc11g069220  TTTTTGTGCT GGCTATTTTT CATGTATTTT ATTGTGTTAC CACATTGGCA 486
        SIMLO8_LEAF  TTTTTGTGCT GGCTATTTTT CATGTATTTT ATTGTGTTAC CACATTGGCA 525
```

```
                                   2,020                         2,040
gDNA_Solyc11g069220  TTGGGAAGAG CTAAGGTATT TTAATCAAAC AATATATAAA TAAATATTTA 2050
cDNA_Solyc11g069220  TTGGGAAGAG CTAAG----- ---------- ---------- ---------- 501
        SIMLO8_LEAF  TTGGGAAGAG CTAAG----- ---------- ---------- ---------- 540
                          2,060                         2,080                    2,100
gDNA_Solyc11g069220  TTTACTTATT TTTCCTTTTT TTAAAAAAAA ATAGAAAATA CTATAGAAGT 2100
cDNA_Solyc11g069220  ---------- ---------- ---------- ---------- ---------- 501
        SIMLO8_LEAF  ---------- ---------- ---------- ---------- ---------- 540
                                   2,120                         2,140
gDNA_Solyc11g069220  ATTCAAAATA GGTCAAGTTA TTTTTTAATT TATTATTAAT TTGGTTGTAT 2150
cDNA_Solyc11g069220  ---------- ---------- ---------- ---------- ---------- 501
        SIMLO8_LEAF  ---------- ---------- ---------- ---------- ---------- 540
                          2,160                         2,180                    2,200
gDNA_Solyc11g069220  TTTATTAATT TCAGATGAGT CGTTGGAAGA TATGGGAAAA GGAAACAAGA 2200
cDNA_Solyc11g069220  ---------- ----ATGAGT CGTTGGAAGA TATGGGAAAA GGAAACAAGA 537
        SIMLO8_LEAF  ---------- ----ATGAGT CGTTGGAAGA TATGGGAAAA GGAAACAAGA 576
                                   2,220                         2,240
gDNA_Solyc11g069220  ACAGCTGAGT ACCAATTTTC TCATGGTAAA AATCATCCTA AAAATAAATT 2250
cDNA_Solyc11g069220  ACAGCTGAGT ACCAATTTTC TCATG----- ---------- ---------- 562
        SIMLO8_LEAF  ACAGCTGAGT ACCAATTTTC TCATG----- ---------- ---------- 601
                          2,260                         2,280                    2,300
gDNA_Solyc11g069220  TTCGATCGAA AATTATCTCG TTAAATATAG AGCATGTTAT CAATGTCTAA 2300
cDNA_Solyc11g069220  ---------- ---------- ---------- ---------- ---------- 562
        SIMLO8_LEAF  ---------- ---------- ---------- ---------- ---------- 601
                                   2,320                         2,340
gDNA_Solyc11g069220  AATTTTTATT TTTTTAGTAG TGTAAGAAAT TAAGAGTCAT TATATTAACG 2350
cDNA_Solyc11g069220  ---------- ---------- ---------- ---------- ---------- 562
        SIMLO8_LEAF  ---------- ---------- ---------- ---------- ---------- 601
                          2,360                         2,380                    2,400
gDNA_Solyc11g069220  TGTTTAATAA TTTAACAGAT CCAGAGAGAT TTCGATTTGC TAGAGATACA 2400
cDNA_Solyc11g069220  ---------- --------AT CCAGAGAGAT TTCGATTTGC TAGAGATACA 594
        SIMLO8_LEAF  ---------- --------AT CCAGAGAGAT TTCGATTTGC TAGAGATACA 633
                                   2,420                         2,440
gDNA_Solyc11g069220  TCATTTGGAA GAAGACATTT GAGTTTTTGG ACTAAAAATT CAGTTCTTCT 2450
cDNA_Solyc11g069220  TCATTTGGAA GAAGACATTT GAGTTTTTGG ACTAAAAATT CAGTTCTTCT 644
        SIMLO8_LEAF  TCATTTGGAA GAAGACATTT GAGTTTTTGG ACTAAAAATT CAGTTCTTCT 683
                          2,460                         2,480                    2,500
gDNA_Solyc11g069220  ATGGATTGTA AGTATCCTTG ATCTCAAATT TATTATAAAA ATAAATTTTG 2500
cDNA_Solyc11g069220  ATGGATTGT- ---------- ---------- ---------- ---------- 653
        SIMLO8_LEAF  ATGGATTGT- ---------- ---------- ---------- ---------- 692
                                   2,520                         2,540
gDNA_Solyc11g069220  AGTTAAAAAA GTCTATTATA ATTGAAAATA ATATATTATG TTATATTTTT 2550
cDNA_Solyc11g069220  ---------- ---------- ---------- ---------- ---------- 653
        SIMLO8_LEAF  ---------- ---------- ---------- ---------- ---------- 692
                          2,560                         2,580                    2,600
gDNA_Solyc11g069220  ACATTTAACT ATTTTATTTG TAATTTAATT TGCAGGTTTG TTTCTTCAGG 2600
cDNA_Solyc11g069220  ---------- ---------- ---------- -------TTG TTTCTTCAGG 666
        SIMLO8_LEAF  ---------- ---------- ---------- -------TTG TTTCTTCAGG 705
                                   2,620                         2,640
gDNA_Solyc11g069220  CAATTTGTAA GATCTGTTCC AAAAGTTGAT TATTTGACCC TACGACATGG 2650
cDNA_Solyc11g069220  CAATTTGTAA GATCTGTTCC AAAAGTTGAT TATTTGACCC TACGACATGG 716
        SIMLO8_LEAF  CAATTTGTAA GATCTGTTCC AAAAGTTGAT TATTTGACCC TACGACATGG 755
                          2,660                         2,680                    2,700
gDNA_Solyc11g069220  TTTTATCACG GTAAACACAT AATAAATAAT TAATATAAAT GATATAACTA 2700
cDNA_Solyc11g069220  TTTTATCACG ---------- ---------- ---------- ---------- 726
        SIMLO8_LEAF  TTTTATCACG GCACATTTGG CACCTCAGAG CAACATAAAT ---------- 795
                                   2,720                         2,740
gDNA_Solyc11g069220  ATTAATCTTG AAAATTTAAT GATAATTCAG TTCATTAAAT AAATATATGA 2750
cDNA_Solyc11g069220  ---------- ---------- ---------- ---------- ---------- 726
        SIMLO8_LEAF  ---------- ---------- ---------- ---------- ---------- 795
                          2,760                         2,780                    2,800
gDNA_Solyc11g069220  AAATGGAAAA TGACAATTTA TGAGTTTCTT TCAATATATA ATTTTTTTAA 2800
cDNA_Solyc11g069220  ---------- ---------- ---------- ---------- ---------- 726
        SIMLO8_LEAF  ---------- ---------- ---------- ---------- ---------- 795
                                   2,820                         2,840
gDNA_Solyc11g069220  AAAAAATCAT ATATTTTTTA AAGCACAGAT GAATGATGTA CAGACTCAAT 2850
cDNA_Solyc11g069220  ---------- ---------- ---------- ---------- ---------- 726
        SIMLO8_LEAF  ---------- ---------- ---------- ---------- ---------- 795
                          2,860                         2,880                    2,900
gDNA_Solyc11g069220  GAATTCAAAA AAACTGACGC AAGCCATAAA TATATAGATA TAACTAAAAT 2900
cDNA_Solyc11g069220  ---------- ---------- ---------- ---------- ---------- 726
        SIMLO8_LEAF  ---------- ---------- ---------- ---------- ---------- 795
                                   2,920                         2,940
gDNA_Solyc11g069220  TTATAAAGGT CTGAATTCAT TACCTTTAAT CGGAGTATAT TTATTTAAAA 2950
cDNA_Solyc11g069220  ---------- ---------- ---------- ---------- ---------- 726
        SIMLO8_LEAF  ---------- ---------- ---------- ---------- ---------- 795
                          2,960                         2,980                    3,000
gDNA_Solyc11g069220  TTATATATAT ATATATATAT ATATATATAT ATATATATAT ATATATANNN 3000
cDNA_Solyc11g069220  ---------- ---------- ---------- ---------- ---------- 726
        SIMLO8_LEAF  ---------- ---------- ---------- ---------- ---------- 795
```

```
                                       3,020                                   3,040
gDNA_Solyc11g069220  NNNNNNNNNN NNNNNNNNNN NNNNNNNNNN NNNNNNNNNN NNNNNNNNNN 3050
cDNA_Solyc11g069220  ---------- ---------- ---------- ---------- ---------- 726
       SlMLO8_LEAF   ---------- ---------- ---------- ---------- ---------- 795
                          3,060                   3,080                  3,100
gDNA_Solyc11g069220  NNNNNNNNNN NNNNNNNNNN NNNNNNNNNN NNNNNNNNNN NNNNNNNNNN 3100
cDNA_Solyc11g069220  ---------- ---------- ---------- ---------- ---------- 726
       SlMLO8_LEAF   ---------- ---------- ---------- ---------- ---------- 795
                                       3,120                                   3,140
gDNA_Solyc11g069220  NNNNNNNNNN NNNNNNNNNN NNNNNNNNNN NNNNNNNNNN NNNNNNNNNN 3150
cDNA_Solyc11g069220  ---------- ---------- ---------- ---------- ---------- 726
       SlMLO8_LEAF   ---------- ---------- ---------- ---------- ---------- 795
                          3,160                   3,180                  3,200
gDNA_Solyc11g069220  NNNNNNNNNN NNNNNNNNNN NNNNNNNNNN NNNNNNNNNN NNNNNNNNNN 3200
cDNA_Solyc11g069220  ---------- ---------- ---------- ---------- ---------- 726
       SlMLO8_LEAF   ---------- ---------- ---------- ---------- ---------- 795
                                       3,220                                   3,240
gDNA_Solyc11g069220  NNNNNNNNNN NNNNNNNNNN NNNNNNNNNN NNNNNNNNNN NNNNNNNNNN 3250
cDNA_Solyc11g069220  ---------- ---------- ---------- ---------- ---------- 726
       SlMLO8_LEAF   ---------- ---------- ---------- ---------- ---------- 795
                          3,260                   3,280                  3,300
gDNA_Solyc11g069220  NNNNNNNNNN NNNNNNNNNN NNNNNNNNNN NNNNNNNNNN NNNNNNNNNN 3300
cDNA_Solyc11g069220  ---------- ---------- ---------- ---------- ---------- 726
       SlMLO8_LEAF   ---------- ---------- ---------- ---------- ---------- 795
                                       3,320                                   3,340
gDNA_Solyc11g069220  NNNNNNNNNN NNNNNNNNNN NNNNNNNNNN NNNNNNNNNN NNNNNNNNNN 3350
cDNA_Solyc11g069220  ---------- ---------- ---------- ---------- ---------- 726
       SlMLO8_LEAF   ---------- ---------- ---------- ---------- ---------- 795
                          3,360                   3,380                  3,400
gDNA_Solyc11g069220  NNNNNNNNNN NNNNNNNNNN NNNNNNNNNN NNNNNNNNNN NNNNNNNNNN 3400
cDNA_Solyc11g069220  ---------- ---------- ---------- ---------- ---------- 726
       SlMLO8_LEAF   ---------- ---------- ---------- ---------- ---------- 795
                                       3,420                                   3,440
gDNA_Solyc11g069220  NNNNNNNNNN NNNNNNNNNN NNNNNNNNNN NNNNNNNNNN NNNNNNNNNN 3450
cDNA_Solyc11g069220  ---------- ---------- ---------- ---------- ---------- 726
       SlMLO8_LEAF   ---------- ---------- ---------- ---------- ---------- 795
                          3,460                   3,480                  3,500
gDNA_Solyc11g069220  NNNNNNNNNN NNNNNNNNNN NNNNNNNNNN NNNNNNNNNN NNNNNNNNNN 3500
cDNA_Solyc11g069220  ---------- ---------- ---------- ---------- ---------- 726
       SlMLO8_LEAF   ---------- ---------- ---------- ---------- ---------- 795
                                       3,520                                   3,540
gDNA_Solyc11g069220  NNNNNNNNNN NNNNNNNNNN NNNNNNNNNN NNNNNNNNNN NNNNNNNNNN 3550
cDNA_Solyc11g069220  ---------- ---------- ---------- ---------- ---------- 726
       SlMLO8_LEAF   ---------- ---------- ---------- ---------- ---------- 795
                          3,560                   3,580                  3,600
gDNA_Solyc11g069220  NNNNNNNNNN NNNNNNNNNN NNNNNNNNNN NNNNNNNNNN NNNNNNNNNN 3600
cDNA_Solyc11g069220  ---------- ---------- ---------- ---------- ---------- 726
       SlMLO8_LEAF   ---------- ---------- ---------- ---------- ---------- 795
                                       3,620                                   3,640
gDNA_Solyc11g069220  NNNNNNNNNN NNNNNNNNNN NNNNNNNNNN NNNNNNNNNN NNNNNNNNNN 3650
cDNA_Solyc11g069220  ---------- ---------- ---------- ---------- ---------- 726
       SlMLO8_LEAF   ---------- ---------- ---------- ---------- ---------- 795
                          3,660                   3,680                  3,700
gDNA_Solyc11g069220  NNNNNNNNNN NNNNNNNNNN NNNNNNNNNN NNNNATTAAG AGGTCATTAG 3700
cDNA_Solyc11g069220  ---------- ---------- ---------- ---------- ---------- 726
       SlMLO8_LEAF   ---------- ------TTTG ATTTTCAAAA ATATATTAAG AGGTCATTAG 829
                                       3,720                                   3,740
gDNA_Solyc11g069220  AAGAAGACTT CAAAGTAGTT GTTAGCATAA GGTATTTTTT GACCTTCAAA 3750
cDNA_Solyc11g069220  ---------- ---------- ---------- ---------- ---------- 726
       SlMLO8_LEAF   AAGAAGACTT CAAAGTAGTT GTTAGCATAA G--------- ---------- 860
                          3,760                   3,780                  3,800
gDNA_Solyc11g069220  CTTATAATTA TTATTTCTAA ATTTAAATAA TTAAATATAC TTTGATCCTT 3800
cDNA_Solyc11g069220  ---------- ---------- ---------- ---------- ---------- 726
       SlMLO8_LEAF   ---------- ---------- ---------- ---------- ---------- 860
                                       3,820                                   3,840
gDNA_Solyc11g069220  TTTTTTTTTG GCAAAAATAT TGTGTTTATT CCATATATAG CCAAAATTGA 3850
cDNA_Solyc11g069220  ---------- ---------- ---------- ---------- ---------- 726
       SlMLO8_LEAF   ---------- ---------- ---------- ---------- ---------- 860
                          3,860                   3,880                  3,900
gDNA_Solyc11g069220  TCTTCTTTTT TTGTTGTATT TTTTTTGCAG TCCACCAATT TGGTTCCTTG 3900
cDNA_Solyc11g069220  ---------- ---------- ---------- TCCACCAATT TGGTTCCTTG 746
       SlMLO8_LEAF   ---------- ---------- ---------- TCCACCAATT TGGTTCCTTG 880
                                       3,920                                   3,940
gDNA_Solyc11g069220  CTGTATTATT CCTACTCTTC AATACTCATG GTAAATAAAT ACTGAAATTA 3950
cDNA_Solyc11g069220  CTG------- ---------- ---------- ---------- ---------- 749
       SlMLO8_LEAF   CTGTATTATT CCTACTCTTC AATACTCATG G--------- --------- - 911
                          3,960                   3,980                  4,000
gDNA_Solyc11g069220  ACTTTCAATT AATTAAAATA TACGTTTTTG ATATCATATC AATATTATAT 4000
cDNA_Solyc11g069220  ---------- ---------- ---------- ---------- ---------- 749
       SlMLO8_LEAF   ---------- ---------- ---------- ---------- ---------- 911
```

```
                                      4,020                                   4,040
gDNA_Solyc11g069220  TATATTCTAA CTATTTATAA AAGTGTGTCA CACTCAAATA TGAAGTGAAA 4050
cDNA_Solyc11g069220  ---------- ---------- ---------- ---------- ---------- 749
        SlMLO8_LEAF  ---------- ---------- ---------- ---------- ---------- 911

                           4,060                      4,080                4,100
gDNA_Solyc11g069220  ATAAAGTTTA ATTTAGTACA CATATATATT CAAATAGTAA AACGATTGAT 4100
cDNA_Solyc11g069220  ---------- ---------- ---------- ---------- ---------- 749
        SlMLO8_LEAF  ---------- ---------- ---------- ---------- ---------- 911

                                      4,120                                   4,140
gDNA_Solyc11g069220  ACTTCAAGTA AACATTTTTT TTAAATTTTA ATCAAATATT TCGAGTTTAA 4150
cDNA_Solyc11g069220  ---------- ---------- ---------- ---------- ---------- 749
        SlMLO8_LEAF  ---------- ---------- ---------- ---------- ---------- 911

                           4,160                      4,180                4,200
gDNA_Solyc11g069220  ATTTTAAATA TGTAAAATTA ATCAATTTTA TTACGGAATG CTTTGTCTTT 4200
cDNA_Solyc11g069220  ---------- ---------- ---------- ---------- ---------- 749
        SlMLO8_LEAF  ---------- ---------- ---------- ---------- ---------- 911

                                      4,220                                   4,240
gDNA_Solyc11g069220  TGATTTGAAC TTTCCAATAT GAATCTAAAT GTCTTTACCT TTTGGTATAA 4250
cDNA_Solyc11g069220  ---------- ---------- ---------- ---------- ---------- 749
        SlMLO8_LEAF  ---------- ---------- ---------- ---------- ---------- 911

                           4,260                      4,280                4,300
gDNA_Solyc11g069220  ATTTTTTTAA TATAAATTTA AATTTAATCA AATTTTAATA TAATTATCGA 4300
cDNA_Solyc11g069220  ---------- ---------- ---------- ---------- ---------- 749
        SlMLO8_LEAF  ---------- ---------- ---------- ---------- ---------- 911

                                      4,320                                   4,340
gDNA_Solyc11g069220  AATTTATTAA GTAATTTCAT TGTCATTTGT GGACAGGCTG GTATTCTTAT 4350
cDNA_Solyc11g069220  ---------- ---------- ---------- ---------- GTATTCTTAT 759
        SlMLO8_LEAF  ---------- ---------- ---------- -------CTG GTATTCTTAT 924

                           4,360                      4,380                4,400
gDNA_Solyc11g069220  CTGTGGCTAC CGTTCATTCC GTTACTTGTA AGTATTTCCA AAATCATTTC 4400
cDNA_Solyc11g069220  CTGTGGCTAC CGTTCATTCC GTTACTTGT- ---------- ---------- 788
        SlMLO8_LEAF  CTGTGGCTAC CGTTCATTCC GTTACTTGT- ---------- ---------- 953

                                      4,420                                   4,440
gDNA_Solyc11g069220  GCATTTGACT TTTATTCATT TATTTCTATT TTTCATATTT TATATATATA 4450
cDNA_Solyc11g069220  ---------- ---------- ---------- ---------- ---------- 788
        SlMLO8_LEAF  ---------- ---------- ---------- ---------- ---------- 953

                           4,460                      4,480                4,500
gDNA_Solyc11g069220  TCCATCTTCC CATAGTCATA TACAATAATT TTTAATAATT CTTTATGTAC 4500
cDNA_Solyc11g069220  ---------- ---------- ---------- ---------- ---------- 788
        SlMLO8_LEAF  ---------- ---------- ---------- ---------- ---------- 953

                                      4,520                                   4,540
gDNA_Solyc11g069220  TAAAAAATTA TTTATTACAT CTCTCTATTT TAATTTATGT TGTATTTTTT 4550
cDNA_Solyc11g069220  ---------- ---------- ---------- ---------- ---------- 788
        SlMLO8_LEAF  ---------- ---------- ---------- ---------- ---------- 953

                           4,560                      4,580                4,600
gDNA_Solyc11g069220  ATTTTTCGAG AGCTAATAAA TAATTAAAGC ATGACTGAGA ATTTACACAT 4600
cDNA_Solyc11g069220  ---------- ---------- ---------- ---------- ---------- 788
        SlMLO8_LEAF  ---------- ---------- ---------- ---------- ---------- 953

                                      4,620                                   4,640
gDNA_Solyc11g069220  GAATTATAAT ATTTTTTTAA AAATGAAATT TATACATATG TAAACTATGT 4650
cDNA_Solyc11g069220  ---------- ---------- ---------- ---------- ---------- 788
        SlMLO8_LEAF  ---------- ---------- ---------- ---------- ---------- 953

                           4,660                      4,680                4,700
gDNA_Solyc11g069220  AATTAAAGAG TATTATAATT CACAATAATT GACAATTCAA AATATTTAAA 4700
cDNA_Solyc11g069220  ---------- ---------- ---------- ---------- ---------- 788
        SlMLO8_LEAF  ---------- ---------- ---------- ---------- ---------- 953

                                      4,720                                   4,740
gDNA_Solyc11g069220  AGATATACGG AAAAATTTAC AGTCTTGTTT GAATCTCAAA ATTTAAAAAA 4750
cDNA_Solyc11g069220  ---------- ---------- ---------- ---------- ---------- 788
        SlMLO8_LEAF  ---------- ---------- ---------- ---------- ---------- 953

                           4,760                      4,780                4,800
gDNA_Solyc11g069220  TACCACATAA ATTAAGATGG AAAGAATATT AATTACACAG TATTATATAT 4800
cDNA_Solyc11g069220  ---------- ---------- ---------- ---------- ---------- 788
        SlMLO8_LEAF  ---------- ---------- ---------- ---------- ---------- 953

                                      4,820                                   4,840
gDNA_Solyc11g069220  TAACTTGAGA TTGTTAATCG TATAACAACT CATCATAATT TTTGAATTCC 4850
cDNA_Solyc11g069220  ---------- ---------- ---------- ---------- ---------- 788
        SlMLO8_LEAF  ---------- ---------- ---------- ---------- ---------- 953

                           4,860                      4,880                4,900
gDNA_Solyc11g069220  GAATTTTGTC TATGTTTTTT GATGCTTTTA ATATTCGTAA AAATTCTGAC 4900
cDNA_Solyc11g069220  ---------- ---------- ---------- ---------- ---------- 788
        SlMLO8_LEAF  ---------- ---------- ---------- ---------- ---------- 953

                                      4,920                                   4,940
gDNA_Solyc11g069220  TTCGTCATCG ATCGATACAG GTGATATTAT TAGTAGGGAC TAAACTACAA 4950
cDNA_Solyc11g069220  ---------- ---------- --GATATTAT TAGTAGGGAC TAAACTACAA 816
        SlMLO8_LEAF  ---------- ---------- --GATATTAT TAGTAGGGAC TAAACTACAA 981

                           4,960                      4,980                5,000
gDNA_Solyc11g069220  GTGATTATAA CAAAAATGGG ATTAAGAATT CAAGAAAGGG GAGAAGTAGT 5000
cDNA_Solyc11g069220  GTGATTATAA CAAAAATGGG ATTAAGAATT CAAGAAAGGG GAGAAGTAGT 866
        SlMLO8_LEAF  GTGATTATAA CAAAAATGGG ATTAAGAATT CAAGAAAGGG GAGAAGTAGT 1031
```

```
                                   5,020                         5,040
gDNA_Solyc11g069220 GAAAGGGGTA CCTGTGGTTC AACCTGGAGA TGATTTATTT TGGTTTAATC 5050
cDNA_Solyc11g069220 GAAAGGGGTA CCTGTGGTTC AACCTGGAGA TGATTTATTT TGGTTTAATC 916
        SIMLO8_LEAF GAAAGGGGTA CCTGTGGTTC AACCTGGAGA TGATTTATTT TGGTTTAATC 1081

                         5,060                         5,080                         5,100
gDNA_Solyc11g069220 GTCCTCGTCT ACTACTTTTT TTAATTAATT TTGTTCTTTT TCAGGTATTT 5100
cDNA_Solyc11g069220 GTCCTCGTCT ACTACTTTTT TTAATTAATT TTGTTCTTTT TCAG------ 960
        SIMLO8_LEAF GTCCTCGTCT ACTACTTTTT TTAATTAATT TTGTTCTTTT TCAG------ 1125

                                   5,120                         5,140
gDNA_Solyc11g069220 TTTAAATATT TATTTTTAAG AAAAGTCATT TTTTAAAATT TAAATAATTT 5150
cDNA_Solyc11g069220 ---------- ---------- ---------- ---------- ---------- 960
        SIMLO8_LEAF ---------- ---------- ---------- ---------- ---------- 1125

                         5,160                         5,180                         5,200
gDNA_Solyc11g069220 AATTTTTATT TTTGATCTTG TTAATATATA TATATATATA TATATATTAT 5200
cDNA_Solyc11g069220 ---------- ---------- ---------- ---------- ---------- 960
        SIMLO8_LEAF ---------- ---------- ---------- ---------- ---------- 1125

                                   5,220                         5,240
gDNA_Solyc11g069220 TTTTTTTTTT TTGTGATTTA TTGCAGAATG CTTTTCAATT GGCTTTCTTT 5250
cDNA_Solyc11g069220 ---------- ---------- ------AATG CTTTTCAATT GGCTTTCTTT 984
        SIMLO8_LEAF ---------- ---------- ------AATG CTTTTCAATT GGCTTTCTTT 1149

                         5,260                         5,280                         5,300
gDNA_Solyc11g069220 GCTTGGACTT GGGTAAGTCC ACCAATTAAC TCTACTAGTA ATATCAACTT 5300
cDNA_Solyc11g069220 GCTTGGACTT GG-------- ---------- ---------- ---------- 996
        SIMLO8_LEAF GCTTGGACTT GG-------- ---------- ---------- ---------- 1161

                                   5,320                         5,340
gDNA_Solyc11g069220 TCGAAAATCG ATAAAATAGT TATTGGCGTG GAGTTTCGAA TTTCATTTGT 5350
cDNA_Solyc11g069220 ---------- ---------- ---------- ---------- ---------- 996
        SIMLO8_LEAF ---------- ---------- ---------- ---------- ---------- 1161

                         5,360                         5,380                         5,400
gDNA_Solyc11g069220 GAAAATATTT TGGATTCTAT TTGTTATAAT AATAACGGTA TAATGTATTA 5400
cDNA_Solyc11g069220 ---------- ---------- ---------- ---------- ---------- 996
        SIMLO8_LEAF ---------- ---------- ---------- ---------- ---------- 1161

                                   5,420                         5,440
gDNA_Solyc11g069220 AGTATAATTT TATAAGTGAG GTCTGAAGGG TAGTTTGGTC GGTTAGTGGT 5450
cDNA_Solyc11g069220 ---------- ---------- ---------- ---------- ---------- 996
        SIMLO8_LEAF ---------- ---------- ---------- ---------- ---------- 1161

                         5,460                         5,480                         5,500
gDNA_Solyc11g069220 GGAGCCACCT TATATTCAGG TGTTCATCTG AATTTTTTTT TGGCAGAAAA 5500
cDNA_Solyc11g069220 ---------- ---------- ---------- ---------- ---------- 996
        SIMLO8_LEAF ---------- ---------- ---------- ---------- ---------- 1161

                                   5,520                         5,540
gDNA_Solyc11g069220 TTATATTATT TATTCATGCT TAAAATCACT TTTAGGTATA TATAGTAGAT 5550
cDNA_Solyc11g069220 ---------- ---------- ---------- ---------- ---------- 996
        SIMLO8_LEAF ---------- ---------- ---------- ---------- ---------- 1161

                         5,560                         5,580                         5,600
gDNA_Solyc11g069220 GTCGAATTCT CTTCGGCTAT TTCGTGTGTC TATTTCCTTA AATTTTAAAT 5600
cDNA_Solyc11g069220 ---------- ---------- ---------- ---------- ---------- 996
        SIMLO8_LEAF ---------- ---------- ---------- ---------- ---------- 1161

                                   5,620                         5,640
gDNA_Solyc11g069220 TCTTTTATTG AAAATTTTGA CTTAGCCGTT GCAGTCAGTA TACTAAGCTT 5650
cDNA_Solyc11g069220 ---------- ---------- ---------- ---------- ---------- 996
        SIMLO8_LEAF ---------- ---------- ---------- ---------- ---------- 1161

                         5,660                         5,680                         5,700
gDNA_Solyc11g069220 CCGCAATATA GGGTTCTATT ATAAAAACAA TTATCACGTA TCTTTATAAG 5700
cDNA_Solyc11g069220 ---------- ---------- ---------- ---------- ---------- 996
        SIMLO8_LEAF ---------- ---------- ---------- ---------- ---------- 1161

                                   5,720                         5,740
gDNA_Solyc11g069220 AGACTGTTTT CACATTCTGT ATATTACGGG ACAACAATTT ATAAACATAT 5750
cDNA_Solyc11g069220 ---------- ---------- ---------- ---------- ---------- 996
        SIMLO8_LEAF ---------- ---------- ---------- ---------- ---------- 1161

                         5,760                         5,780                         5,800
gDNA_Solyc11g069220 AATAGTATTT TGAAATTTCA TGATAACAAC TATTTGTCAA TTATAAGGAC 5800
cDNA_Solyc11g069220 ---------- ---------- ---------- ---------- ---------- 996
        SIMLO8_LEAF ---------- ---------- ---------- ---------- ---------- 1161

                                   5,820                         5,840
gDNA_Solyc11g069220 CGAAAAGTGA TTATTTGCGA ATTATATCTC ATAATTAATT CTTATTTTGT 5850
cDNA_Solyc11g069220 ---------- ---------- ---------- ---------- ---------- 996
        SIMLO8_LEAF ---------- ---------- ---------- ---------- ---------- 1161

                         5,860                         5,880                         5,900
gDNA_Solyc11g069220 TTGATTTCTC TTGTAATTTA AAAAAAAACA GTATGAATTT GGGCTGAAAT 5900
cDNA_Solyc11g069220 ---------- ---------- ---------- -TATGAATTT GGGCTGAAAT 1015
        SIMLO8_LEAF ---------- ---------- ---------- -TATGAATTT GGGCTGAAAT 1180

                                   5,920                         5,940
gDNA_Solyc11g069220 CTTGTTTCCA TGACCAAACT GAGGACATCG TAATTAGAAT GACAATGGGG 5950
cDNA_Solyc11g069220 CTTGTTTCCA TGACCAAACT GAGGACATCG TAATTAGAAT GACAATGGGG 1065
        SIMLO8_LEAF CTTGTTTCCA TGACCAAACT GAGGACATCG TAATTAGAAT GACAATGGGG 1230

                         5,960                         5,980                         6,000
gDNA_Solyc11g069220 TTAGTATTAA TTATTTTCTA ATTTATAATT AAAATTTTAT TAATTTGATC 6000
cDNA_Solyc11g069220 ---------- ---------- ---------- ---------- ---------- 1065
        SIMLO8_LEAF ---------- ---------- ---------- ---------- ---------- 1230
```

```
                                        6,020                          6,040
gDNA_Solyc11g069220  CAATTTACTT ACATATAAAA ATGGTTTGAT TTAATTTTTG GTGCAGGGTT 6050
cDNA_Solyc11g069220  ---------- ---------- ---------- ---------- -------GTT 1068
        SIMLO8_LEAF  ---------- ---------- ---------- ---------- -------GTT 1233
                          6,060                          6,080                     6,100
gDNA_Solyc11g069220  CTTATTCAGA TTCTTTGCAG CTATGTTACT CTTCCATTAT ATGCTCTTGT 6100
cDNA_Solyc11g069220  CTTATTCAGA TTCTTTGCAG CTATGTTACT CTTCCATTAT ATGCTCTTGT 1118
        SIMLO8_LEAF  CTTATTCAGA TTCTTTGCAG CTATGTTACT CTTCCATTAT ATGCTCTTGT 1283
                                        6,120                          6,140
gDNA_Solyc11g069220  AACACAGGTA AATTAAGTTA TATATATCGT CAACGTAAAT TTTTGTTTTT 6150
cDNA_Solyc11g069220  AACACAG--- ---------- ---------- ---------- ---------- 1125
        SIMLO8_LEAF  AACACAG--- ---------- ---------- ---------- ---------- 1290
                          6,160                          6,180                     6,200
gDNA_Solyc11g069220  ATCTCATAAG ATTACATTTG AATATACATC TCGTTTTATT TTTAAGATTA 6200
cDNA_Solyc11g069220  ---------- ---------- ---------- ---------- ---------- 1125
        SIMLO8_LEAF  ---------- ---------- ---------- ---------- ---------- 1290
                                        6,220                          6,240
gDNA_Solyc11g069220  TTAATCTCGC ATTTTAAGTT TCAAGTCACA AAAACAATAA CATAATAATA 6250
cDNA_Solyc11g069220  ---------- ---------- ---------- ---------- ---------- 1125
        SIMLO8_LEAF  ---------- ---------- ---------- ---------- ---------- 1290
                          6,260                          6,280                     6,300
gDNA_Solyc11g069220  ACGAATATTA ACAACTCCTA GATATTACGT GGGCGTTTGG ACATAAGAAT 6300
cDNA_Solyc11g069220  ---------- ---------- ---------- ---------- ---------- 1125
        SIMLO8_LEAF  ---------- ---------- ---------- ---------- ---------- 1290
                                        6,320                          6,340
gDNA_Solyc11g069220  TTATGTGAAT TTTGGAAGAA AGTAGAATGT TTTATTTTCA AGTTAAAAAT 6350
cDNA_Solyc11g069220  ---------- ---------- ---------- ---------- ---------- 1125
        SIMLO8_LEAF  ---------- ---------- ---------- ---------- ---------- 1290
                          6,360                          6,380                     6,400
gDNA_Solyc11g069220  AATGTTAAGA AATTAAAAAT TGAAATTGTG TTTGGACACG AATACAAATT 6400
cDNA_Solyc11g069220  ---------- ---------- ---------- ---------- ---------- 1125
        SIMLO8_LEAF  ---------- ---------- ---------- ---------- ---------- 1290
                                        6,420                          6,440
gDNA_Solyc11g069220  GAAGTTGTTT CTTTAAACGT TCGCGAATGA TTTGTAGTGA AAAATTGTGA 6450
cDNA_Solyc11g069220  ---------- ---------- ---------- ---------- ---------- 1125
        SIMLO8_LEAF  ---------- ---------- ---------- ---------- ---------- 1290
                          6,460                          6,480                     6,500
gDNA_Solyc11g069220  AAAATGGAGT TTCTTTGGAT TTCGAAAAAT TAAAACAATT CTACGTTCAA 6500
cDNA_Solyc11g069220  ---------- ---------- ---------- ---------- ---------- 1125
        SIMLO8_LEAF  ---------- ---------- ---------- ---------- ---------- 1290
                                        6,520                          6,540
gDNA_Solyc11g069220  ATAATTTTCT TCCGAAAAAA ATGAAAACTA TCCATGGTTA ACAAACGGAC 6550
cDNA_Solyc11g069220  ---------- ---------- ---------- ---------- ---------- 1125
        SIMLO8_LEAF  ---------- ---------- ---------- ---------- ---------- 1290
                          6,560                          6,580                     6,600
gDNA_Solyc11g069220  ACTAAAATTT CTATCCTTCA CTAGAATTTT TTTTCCCCAC ACCTTTATAG 6600
cDNA_Solyc11g069220  ---------- ---------- ---------- ---------- ---------- 1125
        SIMLO8_LEAF  ---------- ---------- ---------- ---------- ---------- 1290
                                        6,620                          6,640
gDNA_Solyc11g069220  GAATGAAAAT ACTGATAGGA TTTTACTTAT ATATACTGTC AGTACTAACT 6650
cDNA_Solyc11g069220  ---------- ---------- ---------- ---------- ---------- 1125
        SIMLO8_LEAF  ---------- ---------- ---------- ---------- ---------- 1290
                          6,660                          6,680                     6,700
gDNA_Solyc11g069220  ATCTTACCTT TCATGTTTTC AATTTCATTT ATTATGAAAA TACTGTAAAT 6700
cDNA_Solyc11g069220  ---------- ---------- ---------- ---------- ---------- 1125
        SIMLO8_LEAF  ---------- ---------- ---------- ---------- ---------- 1290
                                        6,720                          6,740
gDNA_Solyc11g069220  ATTTAAGTCT TGCGATAGCA TAAAAATTCT TTTACGTTGT TAGTTCGTAG 6750
cDNA_Solyc11g069220  ---------- ---------- ---------- ---------- ---------- 1125
        SIMLO8_LEAF  ---------- ---------- ---------- ---------- ---------- 1290
                          6,760                          6,780                     6,800
gDNA_Solyc11g069220  AAATTAAACT CATTCTCTCA AAATGTTTGC ACATTTTTTT TAAAATAGAT 6800
cDNA_Solyc11g069220  ---------- ---------- ---------- ---------- --------AT 1127
        SIMLO8_LEAF  ---------- ---------- ---------- ---------- --------AT 1292
                                        6,820                          6,840
gDNA_Solyc11g069220  GGGATCAACA ATGAAACCAA CAATCTTCAA TGAAAGAGTA GCAATGGCAT 6850
cDNA_Solyc11g069220  GGGATCAACA ATGAAACCAA CAATCTTCAA TGAAAGAGTA GCAATGGCAT 1177
        SIMLO8_LEAF  GGGATCAACA ATGAAACCAA CAATCTTCAA TGAAAGAGTA GCAATGGCAT 1342
                          6,860                          6,880                     6,900
gDNA_Solyc11g069220  TGAGAAAATG GCACCATAGT GCCAAAAAAC ACATCAAAGA GATCAACAAG 6900
cDNA_Solyc11g069220  TGAGAAAATG GCACCATAGT GCCAAAAAAC ACATCAAAGA GATCAACAAG 1227
        SIMLO8_LEAF  TGAGAAAATG GCACCATAGT GCCAAAAAAC ACATCAAAGA GATCAACAAG 1392
                                        6,920                          6,940
gDNA_Solyc11g069220  CAACACTCAA ATCCAACAAC ACCAATGTCA AGTAGGCCAC CAACTCCCTC 6950
cDNA_Solyc11g069220  CAACACTCAA ATCCAACAAC ACCAATGTCA AGTAGGCCAC CAACTCCCTC 1277
        SIMLO8_LEAF  CAACACTCAA ATCCAACAAC ACCAATGTCA AGTAGGCCAC CAACTCCCTC 1442
                          6,960                          6,980                     7,000
gDNA_Solyc11g069220  TCACGGCATG TCACCCGTCC ATCTCCTACG CGGTATCAGG ACGAGTGACA 7000
cDNA_Solyc11g069220  TCACGGCATG TCACCCGTCC ATCTCCTACG CGGTATCAGG ACGAGTGACA 1327
        SIMLO8_LEAF  TCACGGCATG TCACCCGTCC ATCTCCTACG CGGTATCAGG ACGAGTGACA 1492
```

```
                                                   7,020                         7,040
                                                     |                             |
gDNA_Solyc11g069220  TGGACGTGGG TCCACGAAGA TCGAGTTATA ATAATATAGA CCATTGGGAT 7050
cDNA_Solyc11g069220  TGGACGTGGG TCCACGAAGA TCGAGTTATA ATAATATAGA CCATTGGGAT 1377
        SlMLO8_LEAF  TGGACGTGGG TCCACGAAGA TCGAGTTATA ATAATATAGA CCATTGGGAT 1542
                               7,060                         7,080                         7,100
                                 |                             |                             |
gDNA_Solyc11g069220  ATTGAGGGCT CACCATCTCC AAATCGTCAC GACTCTGAAG TTCACGAGCC 7100
cDNA_Solyc11g069220  ATTGAGGGCT CACCATCTCC AAATCGTCAC GACTCTGAAG TTCACGAGCC 1427
        SlMLO8_LEAF  ATTGAGGGCT CACCATCTCC AAATCGTCAC GACTCTGAAG TTCACGAGCC 1592
                                                   7,120                         7,140
                                                     |                             |
gDNA_Solyc11g069220  TAATTTGTCA GAAATTGAGG CTCGTGAACA ATACGAGATT AATATTGCTC 7150
cDNA_Solyc11g069220  TAATTTGTCA GAAATTGAGG CTCGTGAACA ATACGAGATT AATATTGCTC 1477
        SlMLO8_LEAF  TAATTTGTCA GAAATTGAGG CTCGTGAACA ATACGAGATT AATATTGCTC 1642
                               7,160                         7,180
                                 |                             |
gDNA_Solyc11g069220  GTTCAAGGGA TTTTTCTTTT GATAAAAGAA CGACTAGTGT ATAA》7194
cDNA_Solyc11g069220  GTTCAAGGGA TTTTTCTTTT GATAAAAGAA CGACTAGTGT ATAA》1521
        SlMLO8_LEAF  GTTCAAGGGA TTTTTCTTTT GATAAAAGAA CGACTAGTGT ATAA》1686
```

```
                                               20                            40
                                                |                             |
gDNA_Solyc06g082820 《CATTTTTCTC TGTTTCATTT CATTAATTAA TTTTATCATA TTTGCCTAAT 50
cDNA_Solyc06g082820 《---------- ---------- ---------- ---------- ---------- -
        SlMLO9_LEAF 《---------- ---------- ---------- ---------- ---------- -
      SlMLO9_FLOWER 《---------- ---------- ---------- ---------- ---------- -
       SlMLO9_FRUIT 《---------- ---------- ---------- ---------- ---------- -
                                  60                            80                 100
                                   |                             |                   |
gDNA_Solyc06g082820  CCATGAAATA ATTTACCAAA ACTTAAACAG GAGAAAATAA TAATAATAAT 100
cDNA_Solyc06g082820  ---------- ---------- ---------- ---------- ---------- -
        SlMLO9_LEAF  ---------- ---------- ---------- ---------- ---------- -
      SlMLO9_FLOWER  ---------- ---------- ---------- ---------- ---------- -
       SlMLO9_FRUIT  ---------- ---------- ---------- ---------- ---------- -
                                              120                           140
                                                |                             |
gDNA_Solyc06g082820  ATATGGAGGG AGGTGGAGAG GAAGAAGGGT CATTGGAATA TACACCAACA 150
cDNA_Solyc06g082820  --ATGGAGGG AGGTGGAGAG GAAGAAGGGT CATTGGAATA TACACCAACA 48
        SlMLO9_LEAF  --A------- ---------- ---------- ---------- ---------- 1
      SlMLO9_FLOWER  --ATGGAGGG AGGTGGAGAG GAAGAAGGGT CATTGGAATA TACACCAACA 48
       SlMLO9_FRUIT  --ATGGAGGG AGGTGGAGAG GAAGAAGGGT CATTGGAATA TACACCAACA 48
                                 160                           180                 200
                                   |                             |                   |
gDNA_Solyc06g082820  TGGGTGGTTG CTGCTGTATG CACAGTCATT GTTTCTATTT CTCTTCTTGT 200
cDNA_Solyc06g082820  TGGGTGGTTG CTGCTGTATG CACAGTCATT GTTTCTATTT CTCTTCTTGT 98
        SlMLO9_LEAF  ---------- ---------- ---------- ---------- ---------- 1
      SlMLO9_FLOWER  TGGGTGGTTG CTGCTGTATG CACAGTCATT GTTTCTATTT CTCTTCTTGT 98
       SlMLO9_FRUIT  TGGGTGGTTG CTGCTGTATG CACAGTCATT GTTTCTATTT CTCTTCTTGT 98
                                              220                           240
                                                |                             |
gDNA_Solyc06g082820  TGAACGTCTC ATCCATTACG CTGGCAAGGT ATATATATAT ATATATATAT 250
cDNA_Solyc06g082820  TGAACGTCTC ATCCATTACG CTGGCAAG-- ---------- ---------- 126
        SlMLO9_LEAF  ---------- ---------- -------G-- ---------- ---------- 2
      SlMLO9_FLOWER  TGAACGTCTC ATCCATTACG CTGGCAAG-- ---------- ---------- 126
       SlMLO9_FRUIT  TGAACGTCTC ATCCATTACG CTGGCAAG-- ---------- ---------- 126
                                 260                           280                 300
                                   |                             |                   |
gDNA_Solyc06g082820  ATTTTTTTTT TTTTCTGTTC TTTAAAAAAA TTATAATTAT TTATCATATG 300
cDNA_Solyc06g082820  ---------- ---------- ---------- ---------- ---------- 126
        SlMLO9_LEAF  ---------- ---------- ---------- ---------- ---------- 2
      SlMLO9_FLOWER  ---------- ---------- ---------- ---------- ---------- 126
       SlMLO9_FRUIT  ---------- ---------- ---------- ---------- ---------- 126
                                              320                           340
                                                |                             |
gDNA_Solyc06g082820  AATGTGCAAT ATGGTTATAT AAAAGATTTT CTCGAAAATG CAGGATAAGA 350
cDNA_Solyc06g082820  ---------- ---------- ---------- ---------- ---------- 126
        SlMLO9_LEAF  ---------- ---------- ---------- ---------- ---------- 2
      SlMLO9_FLOWER  ---------- ---------- ---------- ---------- ---------- 126
       SlMLO9_FRUIT  ---------- ---------- ---------- ---------- ---------- 126
                                 360                           380                 400
                                   |                             |                   |
gDNA_Solyc06g082820  TTTTGTACTT GTAATGTGCG TAGCTGAAGC TTAGTGCACG TAGTTTTTTT 400
cDNA_Solyc06g082820  ---------- ---------- ---------- ---------- ---------- 126
        SlMLO9_LEAF  ---------- ---------- ---------- ---------- ---------- 2
      SlMLO9_FLOWER  ---------- ---------- ---------- ---------- ---------- 126
       SlMLO9_FRUIT  ---------- ---------- ---------- ---------- ---------- 126
                                              420                           440
                                                |                             |
gDNA_Solyc06g082820  TTCTTAAAAG AGTTTATTGG TATTTCTATG AAAACAACAA ATAAATAACA 450
cDNA_Solyc06g082820  ---------- ---------- ---------- ---------- ---------- 126
        SlMLO9_LEAF  ---------- ---------- ---------- ---------- ---------- 2
      SlMLO9_FLOWER  ---------- ---------- ---------- ---------- ---------- 126
       SlMLO9_FRUIT  ---------- ---------- ---------- ---------- ---------- 126
                                 460                           480                 500
                                   |                             |                   |
gDNA_Solyc06g082820  TGCATTTTAT TGTCATGTTT GAATCTGTCT ATTGTTAAGA AATCAATGTC 500
cDNA_Solyc06g082820  ---------- ---------- ---------- ---------- ---------- 126
        SlMLO9_LEAF  ---------- ---------- ---------- ---------- ---------- 2
      SlMLO9_FLOWER  ---------- ---------- ---------- ---------- ---------- 126
       SlMLO9_FRUIT  ---------- ---------- ---------- ---------- ---------- 126
                                              520                           540
                                                |                             |
gDNA_Solyc06g082820  TTTATGAAAT ATAATGTCTT TGTTTTCTCT CTATTTCTGG ATGACAGTTG 550
cDNA_Solyc06g082820  ---------- ---------- ---------- ---------- ---------- 126
        SlMLO9_LEAF  ---------- ---------- ---------- ---------- ---------- 2
      SlMLO9_FLOWER  ---------- ---------- ---------- ---------- ---------- 126
       SlMLO9_FRUIT  ---------- ---------- ---------- ---------- ---------- 126
                                 560                           580                 600
                                   |                             |                   |
gDNA_Solyc06g082820  GTTTGGAGGA TGGTTATCCA CCTGGGATCG ATCCTTCTCA ATGACTTCTG 600
cDNA_Solyc06g082820  ---------- ---------- ---------- ---------- ---------- 126
        SlMLO9_LEAF  ---------- ---------- ---------- ---------- ---------- 2
      SlMLO9_FLOWER  ---------- ---------- ---------- ---------- ---------- 126
       SlMLO9_FRUIT  ---------- ---------- ---------- ---------- ---------- 126
                                              620                           640
                                                |                             |
gDNA_Solyc06g082820  AGTTGAATTT GTCGCATAGG ACAAGCCTGA TACGGTTTTT TACATATAGT 650
cDNA_Solyc06g082820  ---------- ---------- ---------- ---------- ---------- 126
        SlMLO9_LEAF  ---------- ---------- ---------- ---------- ---------- 2
      SlMLO9_FLOWER  ---------- ---------- ---------- ---------- ---------- 126
       SlMLO9_FRUIT  ---------- ---------- ---------- ---------- ---------- 126
                                 660                           680                 700
                                   |                             |                   |
gDNA_Solyc06g082820  GTTCATCACA AGGGCAGAGA GAAAAAAAAA ATTGATTTTC AACATTTGAC 700
cDNA_Solyc06g082820  ---------- ---------- ---------- ---------- ---------- 126
        SlMLO9_LEAF  ---------- ---------- ---------- ---------- ---------- 2
      SlMLO9_FLOWER  ---------- ---------- ---------- ---------- ---------- 126
       SlMLO9_FRUIT  ---------- ---------- ---------- ---------- ---------- 126
```

```
                          720                    740
gDNA_Solyc06g082820 TAAGATTCCA TCATTTTATT TTTCCTTTTC AATTTCGCCA TGTTATGAAA 750
cDNA_Solyc06g082820 ---------- ---------- ---------- ---------- ---------- 126
        SlMLO9_LEAF ---------- ---------- ---------- ---------- ---------- 2
      SlMLO9_FLOWER ---------- ---------- ---------- ---------- ---------- 126
       SlMLO9_FRUIT ---------- ---------- ---------- ---------- ---------- 126
               760                  780                  800
gDNA_Solyc06g082820 CGGTTTACAA GTGGAATGGT ATAAGGAATT CATATAGCGG ATCTGAACTT 800
cDNA_Solyc06g082820 ---------- ---------- ---------- ---------- ---------- 126
        SlMLO9_LEAF ---------- ---------- ---------- ---------- ---------- 2
      SlMLO9_FLOWER ---------- ---------- ---------- ---------- ---------- 126
       SlMLO9_FRUIT ---------- ---------- ---------- ---------- ---------- 126
                          820                    840
gDNA_Solyc06g082820 GTTATTATTG ATCGTTGTTG GTTTGTTCAA GTTTCATTAT TTTATTATAG 850
cDNA_Solyc06g082820 ---------- ---------- ---------- ---------- ---------- 126
        SlMLO9_LEAF ---------- ---------- ---------- ---------- ---------- 2
      SlMLO9_FLOWER ---------- ---------- ---------- ---------- ---------- 126
       SlMLO9_FRUIT ---------- ---------- ---------- ---------- ---------- 126
               860                  880                  900
gDNA_Solyc06g082820 ATGCAACCAA TACGTAGCTT AGCAACATAG AGTGAAAGGT GGTCCGTTAA 900
cDNA_Solyc06g082820 ---------- ---------- ---------- ---------- ---------- 126
        SlMLO9_LEAF ---------- ---------- ---------- ---------- ---------- 2
      SlMLO9_FLOWER ---------- ---------- ---------- ---------- ---------- 126
       SlMLO9_FRUIT ---------- ---------- ---------- ---------- ---------- 126
                          920                    940
gDNA_Solyc06g082820 ACATACTTCA TAGGAAAATC ATACTATATT AATTAGGTAT ATAGGTCAAT 950
cDNA_Solyc06g082820 ---------- ---------- ---------- ---------- ---------- 126
        SlMLO9_LEAF ---------- ---------- ---------- ---------- ---------- 2
      SlMLO9_FLOWER ---------- ---------- ---------- ---------- ---------- 126
       SlMLO9_FRUIT ---------- ---------- ---------- ---------- ---------- 126
               960                  980                  1,000
gDNA_Solyc06g082820 TATTTTCATA CATGTATATG TATATATATG TATTGAACTT TGAACATTCT 1000
cDNA_Solyc06g082820 ---------- ---------- ---------- ---------- ---------- 126
        SlMLO9_LEAF ---------- ---------- ---------- ---------- ---------- 2
      SlMLO9_FLOWER ---------- ---------- ---------- ---------- ---------- 126
       SlMLO9_FRUIT ---------- ---------- ---------- ---------- ---------- 126
                          1,020                  1,040
gDNA_Solyc06g082820 TAACGAAATC TCTGGCTCTG CTACTGGTTG CAGCGTCTGA AGAAGAAGAA 1050
cDNA_Solyc06g082820 ---------- ---------- ---------- ---CGTCTGA AGAAGAAGAA 143
        SlMLO9_LEAF ---------- ---------- ---------- ---CGTCTGA AGAAGAAGAA 19
      SlMLO9_FLOWER ---------- ---------- ---------- ---CGTCTGA AGAAGAAGAA 143
       SlMLO9_FRUIT ---------- ---------- ---------- ---CGTCTGA AGAAGAAGAA 143
               1,060                1,080                1,100
gDNA_Solyc06g082820 ACAAAAGCAT CTGTATGAAG CCCTACAGAA AGTTAAAGAA GGTTCAAAAA 1100
cDNA_Solyc06g082820 ACAAAAGCAT CTGTATGAAG CCCTACAGAA AGTTAAAGAA G--------- 184
        SlMLO9_LEAF ACAAAAGCAT CTGTATGAAG CCCTACAGAA AGTTAAAGAA G--------- 60
      SlMLO9_FLOWER ACAAAAGCAT CTGTATGAAG CCCTACAGAA AGTTAAAGAA G--------- 184
       SlMLO9_FRUIT ACAAAAGCAT CTGTATGAAG CCCTACAGAA AGTTAAAGAA G--------- 184
                          1,120                  1,140
gDNA_Solyc06g082820 TATACAACAT TGATCAATTA AGGTTGTATT TATATATGAA CTATTTTGTG 1150
cDNA_Solyc06g082820 ---------- ---------- ---------- ---------- ---------- 184
        SlMLO9_LEAF ---------- ---------- ---------- ---------- ---------- 60
      SlMLO9_FLOWER ---------- ---------- ---------- ---------- ---------- 184
       SlMLO9_FRUIT ---------- ---------- ---------- ---------- ---------- 184
               1,160                1,180                1,200
gDNA_Solyc06g082820 AATGTGTATT ATGTGCAGAG TTGATGCTGT TGGGGTTTAT TTCTCTGCTG 1200
cDNA_Solyc06g082820 ---------- --------AG TTGATGCTGT TGGGGTTTAT TTCTCTGCTG 216
        SlMLO9_LEAF ---------- --------AG TTGATGCTGT TGGGGTTTAT TTCTCTGCTG 92
      SlMLO9_FLOWER ---------- --------AG TTGATGCTGT TGGGGTTTAT TTCTCTGCTG 216
       SlMLO9_FRUIT ---------- --------AG TTGATGCTGT TGGGGTTTAT TTCTCTGCTG 216
                          1,220                  1,240
gDNA_Solyc06g082820 TTAACAGTAT TTCAAAGTCG TATTGTTGAA ATCTGTGTGC CTCCTCATGT 1250
cDNA_Solyc06g082820 TTAACAGTAT TTCAAAGTCG TATTGTTGAA ATCTGTGTGC CTCCTCATGT 266
        SlMLO9_LEAF TTAACAGTAT TTCAAAGTCG TATTGTTGAA ATCTGTGTGC CTCCTCATGT 142
      SlMLO9_FLOWER TTAACAGTAT TTCAAAGTCG TATTGTTGAA ATCTGTGTGC CTCCTCATGT 266
       SlMLO9_FRUIT TTAACAGTAT TTCAAAGTCG TATTGTTGAA ATCTGTGTGC CTCCTCATGT 266
               1,260                1,280                1,300
gDNA_Solyc06g082820 TGTAACACAC TTGCTTCCCT GTGCGTTACC GTTGGAGCAT ACTTCATTTT 1300
cDNA_Solyc06g082820 TGTAACACAC TTGCTTCCCT GTGCGTTACC GTTGGAGCAT ACTTCATTTT 316
        SlMLO9_LEAF TGTAACACAC TTGCTTCCCT GTGCGTTACC GTTGGAGCAT ACTTCATTTT 192
      SlMLO9_FLOWER TGTAACACAC TTGCTTCCCT GTGCGTTACC GTTGGAGCAT ACTTCATTTT 316
       SlMLO9_FRUIT TGTAACACAC TTGCTTCCCT GTGCGTTACC GTTGGAGCAT ACTTCATTTT 316
                          1,320                  1,340
gDNA_Solyc06g082820 CACCTCCAAC TCCAACTCCA ACTCTAACTC CACCTCCGCC TCACAAAGAG 1350
cDNA_Solyc06g082820 CACCTCCAAC TCCAACTCCA ACTCTAACTC CACCTCCGCC TCACAAAGAG 366
        SlMLO9_LEAF CACCTCCAAC TCCAACTCCA ACTCTAACTC CACCTCCGCC TCACAAAGAG 242
      SlMLO9_FLOWER CACCTCCAAC TCCAACTCCA ACTCTAACTC CACCTCCGCC TCACAAAGAG 366
       SlMLO9_FRUIT CACCTCCAAC TCCAACTCCA ACTCTAACTC CACCTCCGCC TCACAAAGAG 366
               1,360                1,380                1,400
gDNA_Solyc06g082820 CCTCAGGTTA ACAATCAGGC GGTTCACTAT CATGCTGGTC CTCACCATCA 1400
cDNA_Solyc06g082820 CCTCAGGTTA ACAATCAGGC GGTTCACTAT CATGCTGGTC CTCACCATCA 416
        SlMLO9_LEAF CCTCAGGTTA ACAATCAGGC GGTTCACTAT CATGCTGGTC CTCACCATCA 292
      SlMLO9_FLOWER CCTCAGGTTA ACAATCAGGC GGTTCACTAT CATGCTGGTC CTCACCATCA 416
       SlMLO9_FRUIT CCTCAGGTTA ACAATCAGGC GGTTCACTAT CATGCTGGTC CTCACCATCA 416
```

```
                                            1,420                     1,440
gDNA_Solyc06g082820  ACGGCATTTG CTTGAAGAAG AAACAATGTC AGCTGAGGGT TACTGCCGTC 1450
cDNA_Solyc06g082820  ACGGCATTTG CTTGAAGAAG AAACAATGTC AGCTGAGGGT TACTGCCGTC 466
         SlMLO9_LEAF ACGGCATTTG CTTGAAGAAG AAACAATGTC AGCTGAGGGT TACTGCCGTC 342
       SlMLO9_FLOWER ACGGCATTTG CTTGAAGAAG AAACAATGTC AGCTGAGGGT TACTGCCGTC 466
        SlMLO9_FRUIT ACGGCATTTG CTTGAAGAAG AAACAATGTC AGCTGAGGGT TACTGCCGTC 466
                               1,460                     1,480               1,500
gDNA_Solyc06g082820  ATAAAGTACT TAAGTCATTG CTCTTAACTT CCAGCTATAT ACTTTGTGTT 1500
cDNA_Solyc06g082820  ATAAA----- ---------- ---------- ---------- ---------- 471
         SlMLO9_LEAF ATAAA----- ---------- ---------- ---------- ---------- 347
       SlMLO9_FLOWER ATAAA----- ---------- ---------- ---------- ---------- 471
        SlMLO9_FRUIT ATAAA----- ---------- ---------- ---------- ---------- 471
                                            1,520                     1,540
gDNA_Solyc06g082820  GGCGATGTTC TGTTATATCT GTTTCTTGTT AGTTTGCAAG TTACGAGTTT 1550
cDNA_Solyc06g082820  ---------- ---------- ---------- ---------- ---------- 471
         SlMLO9_LEAF ---------- ---------- ---------- ---------- ---------- 347
       SlMLO9_FLOWER ---------- ---------- ---------- ---------- ---------- 471
        SlMLO9_FRUIT ---------- ---------- ---------- ---------- ---------- 471
                               1,560                     1,580               1,600
gDNA_Solyc06g082820  GGCAGAATCC AGTAACTTTG GCTGCACTTT TTTCCTTTTA TGTTTGACAA 1600
cDNA_Solyc06g082820  ---------- ---------- ---------- ---------- ---------- 471
         SlMLO9_LEAF ---------- ---------- ---------- ---------- ---------- 347
       SlMLO9_FLOWER ---------- ---------- ---------- ---------- ---------- 471
        SlMLO9_FRUIT ---------- ---------- ---------- ---------- ---------- 471
                                            1,620                     1,640
gDNA_Solyc06g082820  TATGACTTCA AACATTGGTG TTTACTTCTT ATATAATACT TTCCGTTGTT 1650
cDNA_Solyc06g082820  ---------- ---------- ---------- ---------- ---------- 471
         SlMLO9_LEAF ---------- ---------- ---------- ---------- ---------- 347
       SlMLO9_FLOWER ---------- ---------- ---------- ---------- ---------- 471
        SlMLO9_FRUIT ---------- ---------- ---------- ---------- ---------- 471
                               1,660                     1,680               1,700
gDNA_Solyc06g082820  TTTACAGAAC AAGGTTCCGT TACTATCTCT TGAGGCGTTG CATCACCTTC 1700
cDNA_Solyc06g082820  -------AAC AAGGTTCCGT TACTATCTCT TGAGGCGTTG CATCACCTTC 514
         SlMLO9_LEAF -------AAC AAGGTTCCGT TACTATCTCT TGAGGCGTTG CATCACCTTC 390
       SlMLO9_FLOWER -------AAC AAGGTTCCGT TACTATCTCT TGAGGCGTTG CATCACCTTC 514
        SlMLO9_FRUIT -------AAC AAGGTTCCGT TACTATCTCT TGAGGCGTTG CATCACCTTC 514
                                            1,720                     1,740
gDNA_Solyc06g082820  ATGTTTTTAT ATTTGTCCTA GCTATTGTGC ATGTGACATT CTCTGTTTTG 1750
cDNA_Solyc06g082820  ATGTTTTTAT ATTTGTCCTA GCTATTGTGC ATGTGACATT CTCTGTTTTG 564
         SlMLO9_LEAF ATGTTTTTAT ATTTGTCCTA GCTATTGTGC ATGTGACATT CTCTGTTTTG 440
       SlMLO9_FLOWER ATGTTTTTAT ATTTGTCCTA GCTATTGTGC ATGTGACATT CTCTGTTTTG 564
        SlMLO9_FRUIT ATGTTTTTAT ATTTGTCCTA GCTATTGTGC ATGTGACATT CTCTGTTTTG 564
                               1,760                     1,780               1,800
gDNA_Solyc06g082820  ACTATTGTAT TTGGAGGAGC AAAGGTGAGA GTTACTAAGC TTTTCTGCTT 1800
cDNA_Solyc06g082820  ACTATTGTAT TTGGAGGAGC AAAG------ ---------- ---------- 588
         SlMLO9_LEAF ACTATTGTAT TTGGAGGAGC AAAG------ ---------- ---------- 464
       SlMLO9_FLOWER ACTATTGTAT TTGGAGGAGC AAAG------ ---------- ---------- 588
        SlMLO9_FRUIT ACTATTGTAT TTGGAGGAGC AAAG------ ---------- ---------- 588
                                            1,820                     1,840
gDNA_Solyc06g082820  GAGTTTCTTT TACTTTGTCT TTCGGGCTGA TTCATTGTTT GTGAATGTTT 1850
cDNA_Solyc06g082820  ---------- ---------- ---------- ---------- ---------- 588
         SlMLO9_LEAF ---------- ---------- ---------- ---------- ---------- 464
       SlMLO9_FLOWER ---------- ---------- ---------- ---------- ---------- 588
        SlMLO9_FRUIT ---------- ---------- ---------- ---------- ---------- 588
                               1,860                     1,880               1,900
gDNA_Solyc06g082820  TAATAATGGC CTAGATACGT CAATGGAAGC AATGGGAGGA TGCAATCGTA 1900
cDNA_Solyc06g082820  ---------- ----ATACGT CAATGGAAGC AATGGGAGGA TGCAATCGTA 624
         SlMLO9_LEAF ---------- ----ATACGT CAATGGAAGC AATGGGAGGA TGCAATCGTA 500
       SlMLO9_FLOWER ---------- ----ATACGT CAATGGAAGC AATGGGAGGA TGCAATCGTA 624
        SlMLO9_FRUIT ---------- ----ATACGT CAATGGAAGC AATGGGAGGA TGCAATCGTA 624
                                            1,920                     1,940
gDNA_Solyc06g082820  AAAGATGATT ATGAATCCGA AGATGGTATG ACTAAATAGG AAAGGAATTA 1950
cDNA_Solyc06g082820  AAAGATGATT ATGAATCCGA AGATG----- ---------- ---------- 649
         SlMLO9_LEAF AAAGATGATT ATGAATCCGA AGATG----- ---------- ---------- 525
       SlMLO9_FLOWER AAAGATGATT ATGAATCCGA AGATG----- ---------- ---------- 649
        SlMLO9_FRUIT AAAGATGATT ATGAATCCGA AGATG----- ---------- ---------- 649
                               1,960                     1,980               2,000
gDNA_Solyc06g082820  AGTAAACGGT TGAAATGTAA AGCTACTAGT AATATTGGTA TTCCATTTTG 2000
cDNA_Solyc06g082820  ---------- ---------- ---------- ---------- ---------- 649
         SlMLO9_LEAF ---------- ---------- ---------- ---------- ---------- 525
       SlMLO9_FLOWER ---------- ---------- ---------- ---------- ---------- 649
        SlMLO9_FRUIT ---------- ---------- ---------- ---------- ---------- 649
                                            2,020                     2,040
gDNA_Solyc06g082820  CAGCTCATTT GAAGCCAACA GTTACTCATG TCTTTGAACA TGATTTCATC 2050
cDNA_Solyc06g082820  ---CTCATTT GAAGCCAACA GTTACTCATG TCTTTGAACA TGATTTCATC 696
         SlMLO9_LEAF ---CTCATTT GAAGCCAACA GTTACTCATG TCTTTGAACA TGATTTCATC 572
       SlMLO9_FLOWER ---CTCATTT GAAGCCAACA GTTACTCATG TCTTTGAACA TGATTTCATC 696
        SlMLO9_FRUIT ---CTCATTT GAAGCCAACA GTTACTCATG TCTTTGAACA TGATTTCATC 696
                               2,060                     2,080               2,100
gDNA_Solyc06g082820  AGGAATCGGT TTCAGGGTAT GGGTACACAA TCAGCCATTT TGGGTTGGGT 2100
cDNA_Solyc06g082820  AGGAATCGGT TTCAGGGTAT GGGTACACAA TCAGCCATTT TGGGTTGGGT 746
         SlMLO9_LEAF AGGAATCGGT TTCAGGGTAT GGGTACACAA TCAGCCATTT TGGGTTGGGT 622
       SlMLO9_FLOWER AGGAATCGGT TTCAGGGTAT GGGTACACAA TCAGCCATTT TGGGTTGGGT 746
        SlMLO9_FRUIT AGGAATCGGT TTCAGGGTAT GGGTACACAA TCAGCCATTT TGGGTTGGGT 746
```

```
                                             2,120                         2,140
gDNA_Solyc06g082820  GGTAAGCACC CCCATTCTCC GCGAACAATA TGGAATCTTT GTATTTCCAT 2150
cDNA_Solyc06g082820  ---------- ---------- ---------- ---------- ---------- 746
        SIMLO9_LEAF  ---------- ---------- ---------- ---------- ---------- 622
      SIMLO9_FLOWER  ---------- ---------- ---------- ---------- ---------- 746
       SIMLO9_FRUIT  ---------- ---------- ---------- ---------- ---------- 746
                              2,160                         2,180                         2,200
gDNA_Solyc06g082820  ATGTGCCAAA AAACAAAAAA ACTTCTGTTG TGTGATTGTT TTGACATATG 2200
cDNA_Solyc06g082820  ---------- ---------- ---------- ---------- ---------- 746
        SIMLO9_LEAF  ---------- ---------- ---------- ---------- ---------- 622
      SIMLO9_FLOWER  ---------- ---------- ---------- ---------- ---------- 746
       SIMLO9_FRUIT  ---------- ---------- ---------- ---------- ---------- 746
                                             2,220                         2,240
gDNA_Solyc06g082820  TTCTCAAATG ACTCGCGATA GACTCATATG ATCACCTTCT TTCTCTGATT 2250
cDNA_Solyc06g082820  ---------- ---------- ---------- ---------- ---------- 746
        SIMLO9_LEAF  ---------- ---------- ---------- ---------- ---------- 622
      SIMLO9_FLOWER  ---------- ---------- ---------- ---------- ---------- 746
       SIMLO9_FRUIT  ---------- ---------- ---------- ---------- ---------- 746
                              2,260                         2,280                         2,300
gDNA_Solyc06g082820  CGTATTGGTA TTAATCAACT AACTTACTGT ATTTTGGTGA AAATTTGCAG 2300
cDNA_Solyc06g082820  ---------- ---------- ---------- ---------- ---------G 747
        SIMLO9_LEAF  ---------- ---------- ---------- ---------- ---------G 623
      SIMLO9_FLOWER  ---------- ---------- ---------- ---------- ---------G 747
       SIMLO9_FRUIT  ---------- ---------- ---------- ---------- ---------G 747
                                             2,320                         2,340
gDNA_Solyc06g082820  CGTTCTTTCT TCAAGCAATT TTATGCTTCT GTCAACGAAT CAGACTACAG 2350
cDNA_Solyc06g082820  CGTTCTTTCT TCAAGCAATT TTATGCTTCT GTCAACGAAT CAGACTACAG 797
        SIMLO9_LEAF  CGTTCTTTCT TCAAGCAATT TTATGCTTCT GTCAACGAAT CAGACTACAG 673
      SIMLO9_FLOWER  CGTTCTTTCT TCAAGCAATT TTATGCTTCT GTCAACGAAT CAGACTACAG 797
       SIMLO9_FRUIT  CGTTCTTTCT TCAAGCAATT TTATGCTTCT GTCAACGAAT CAGACTACAG 797
                              2,360                         2,380                         2,400
gDNA_Solyc06g082820  AGCGCTTCGT TTGGGATTCA TTATGGTAAC TATGTTCATT TGCCATAGTT 2400
cDNA_Solyc06g082820  AGCGCTTCGT TTGGGATTCA TTATG----- ---------- ---------- 822
        SIMLO9_LEAF  AGCGCTTCGT TTGGGATTCA TTATG----- ---------- ---------- 698
      SIMLO9_FLOWER  AGCGCTTCGT TTGGGATTCA TTATG----- ---------- ---------- 822
       SIMLO9_FRUIT  AGCGCTTCGT TTGGGATTCA TTATG----- ---------- ---------- 822
                                             2,420                         2,440
gDNA_Solyc06g082820  GTTAGATAGA AACTGTTTTA CAATGTAGTG ATTTTTACTT GGCTTATACA 2450
cDNA_Solyc06g082820  ---------- ---------- ---------- ---------- ---------- 822
        SIMLO9_LEAF  ---------- ---------- ---------- ---------- ---------- 698
      SIMLO9_FLOWER  ---------- ---------- ---------- ---------- ---------- 822
       SIMLO9_FRUIT  ---------- ---------- ---------- ---------- ---------- 822
                              2,460                         2,480                         2,500
gDNA_Solyc06g082820  TAATATTAGT AAAAGCATAT TCCTTGTGTA GACACATTGC AAGGGAAATC 2500
cDNA_Solyc06g082820  ---------- ---------- ---------- -ACACATTGC AAGGGAAATC 841
        SIMLO9_LEAF  ---------- ---------- ---------- -ACACATTGC AAGGGAAATC 717
      SIMLO9_FLOWER  ---------- ---------- ---------- -ACACATTGC AAGGGAAATC 841
       SIMLO9_FRUIT  ---------- ---------- ---------- -ACACATTGC AAGGGAAATC 841
                                             2,520                         2,540
gDNA_Solyc06g082820  CAAGATTCAA TTTTCATAGG TACATGATAC GTGCACTGGA AGACGATTTT 2550
cDNA_Solyc06g082820  CAAGATTCAA TTTTCATAGG TACATGATAC GTGCACTGGA AGACGATTTT 891
        SIMLO9_LEAF  CAAGATTCAA TTTTCATAGG TACATGATAC GTGCACTGGA AGACGATTTT 767
      SIMLO9_FLOWER  CAAGATTCAA TTTTCATAGG TACATGATAC GTGCACTGGA AGACGATTTT 891
       SIMLO9_FRUIT  CAAGATTCAA TTTTCATAGG TACATGATAC GTGCACTGGA AGACGATTTT 891
                              2,560                         2,580                         2,600
gDNA_Solyc06g082820  AGGACAGTTG TTGGTATCAG GCAAGTGATA GAATGATGCT AAAGCTCTTA 2600
cDNA_Solyc06g082820  AGGACAGTTG TTGGTATCAG ---------- ---------- ---------- 911
        SIMLO9_LEAF  AGGACAGTTG TTGGTATCAG ---------- ---------- ---------- 787
      SIMLO9_FLOWER  AGGACAGTTG TTGGTATCAG ---------- ---------- ---------- 911
       SIMLO9_FRUIT  AGGACAGTTG TTGGTATCAG ---------- ---------- ---------- 911
                                             2,620                         2,640
gDNA_Solyc06g082820  GAATGTAGTT TCTCACTGCT GGTTCTTCAA ATGAACTTGT CGCTCTTTGT 2650
cDNA_Solyc06g082820  ---------- ---------- ---------- ---------- ---------- 911
        SIMLO9_LEAF  ---------- ---------- ---------- ---------- ---------- 787
      SIMLO9_FLOWER  ---------- ---------- ---------- ---------- ---------- 911
       SIMLO9_FRUIT  ---------- ---------- ---------- ---------- ---------- 911
                              2,660                         2,680                         2,700
gDNA_Solyc06g082820  TTTTTGTTTG TTTTGCAGTT GGTATCTCTG GATATTCGTA ATCCTCTTCT 2700
cDNA_Solyc06g082820  ---------- --------TT GGTATCTCTG GATATTCGTA ATCCTCTTCT 943
        SIMLO9_LEAF  ---------- --------TT GGTATCTCTG GATATTCGTA ATCCTCTTCT 819
      SIMLO9_FLOWER  ---------- --------TT GGTATCTCTG GATATTCGTA ATCCTCTTCT 943
       SIMLO9_FRUIT  ---------- --------TT GGTATCTCTG GATATTCGTA ATCCTCTTCT 943
                                             2,720                         2,740
gDNA_Solyc06g082820  TGTTGCTTAA CATTAACGGT ATATCCCAGC TCAAACTTGT TCACCTTGCC 2750
cDNA_Solyc06g082820  TGTTGCTTAA CATTAACGGT ---------- ---------- ---------- 963
        SIMLO9_LEAF  TGTTGCTTAA CATTAACGGT ---------- ---------- ---------- 839
      SIMLO9_FLOWER  TGTTGCTTAA CATTAACGGT ---------- ---------- ---------- 963
       SIMLO9_FRUIT  TGTTGCTTAA CATTAACGGT ---------- ---------- ---------- 963
                              2,760                         2,780                         2,800
gDNA_Solyc06g082820  ACACTGCTAC GATTTCATGC TACAGCCTAC TACAATGCTA TTGTGCTTTG 2800
cDNA_Solyc06g082820  ---------- ---------- ---------- ---------- ---------- 963
        SIMLO9_LEAF  ---------- ---------- ---------- ---------- ---------- 839
      SIMLO9_FLOWER  ---------- ---------- ---------- ---------- ---------- 963
       SIMLO9_FRUIT  ---------- ---------- ---------- ---------- ---------- 963
```

```
                                2,820                         2,840
gDNA_Solyc06g082820  TGCTTTCGTT TAGAGGATAG TTTCGTTTAT TGTGATGTGA AGATTGATAC 2850
cDNA_Solyc06g082820  ---------- ---------- ---------- ---------- ---------- 963
        SlMLO9_LEAF  ---------- ---------- ---------- ---------- ---------- 839
      SlMLO9_FLOWER  ---------- ---------- ---------- ---------- ---------- 963
       SlMLO9_FRUIT  ---------- ---------- ---------- ---------- ---------- 963
                          2,860                         2,880                2,900
gDNA_Solyc06g082820  ATCACTGTGC TTGTGCAGGT TGGCATACAT ATTTCTGGAT TGCTTTCTTT 2900
cDNA_Solyc06g082820  ---------- ---------- TGGCATACAT ATTTCTGGAT TGCTTTCTTT 993
        SlMLO9_LEAF  ---------- ---------- TGGCATACAT ATTTCTGGAT TGCTTTCTTT 869
      SlMLO9_FLOWER  ---------- ---------- TGGCATACAT ATTTCTGGAT TGCTTTCTTT 993
       SlMLO9_FRUIT  ---------- ---------- TGGCATACAT ATTTCTGGAT TGCTTTCTTT 993
                                2,920                         2,940
gDNA_Solyc06g082820  CCTTTTATTG TGAGTGACCC CGTCATGAAT TTCACTCGTA TAGTTTTCAT 2950
cDNA_Solyc06g082820  CCTTTTATT- ---------- ---------- ---------- ---------- 1002
        SlMLO9_LEAF  CCTTTTATT- ---------- ---------- ---------- ---------- 878
      SlMLO9_FLOWER  CCTTTTATT- ---------- ---------- ---------- ---------- 1002
       SlMLO9_FRUIT  CCTTTTATT- ---------- ---------- ---------- ---------- 1002
                          2,960                         2,980                3,000
gDNA_Solyc06g082820  TCTTTCAGTC TCCTATCTAT GAATTAACGT TCAGCACACG ATCACGAGAG 3000
cDNA_Solyc06g082820  ---------- ---------- ---------- ---------- ---------- 1002
        SlMLO9_LEAF  ---------- ---------- ---------- ---------- ---------- 878
      SlMLO9_FLOWER  ---------- ---------- ---------- ---------- ---------- 1002
       SlMLO9_FRUIT  ---------- ---------- ---------- ---------- ---------- 1002
                                3,020                         3,040
gDNA_Solyc06g082820  CTATCAATTT AGGCTCTTTT TTTTGTTAAT AAAAATAGTT CAAGGGTAAT 3050
cDNA_Solyc06g082820  ---------- ---------- ---------- ---------- ---------- 1002
        SlMLO9_LEAF  ---------- ---------- ---------- ---------- ---------- 878
      SlMLO9_FLOWER  ---------- ---------- ---------- ---------- ---------- 1002
       SlMLO9_FRUIT  ---------- ---------- ---------- ---------- ---------- 1002
                          3,060                         3,080                3,100
gDNA_Solyc06g082820  CTGCACTGCT ACTCTACTCA TATTCAAGTC ATAATAAAAT GGTAGCCAAC 3100
cDNA_Solyc06g082820  ---------- ---------- ---------- ---------- ---------- 1002
        SlMLO9_LEAF  ---------- ---------- ---------- ---------- ---------- 878
      SlMLO9_FLOWER  ---------- ---------- ---------- ---------- ---------- 1002
       SlMLO9_FRUIT  ---------- ---------- ---------- ---------- ---------- 1002
                                3,120                         3,140
gDNA_Solyc06g082820  ATGATCAAGG CTTAAAAGCA GCAGGAAGCT AAGTTGATTT TATGTTGCCT 3150
cDNA_Solyc06g082820  ---------- ---------- ---------- ---------- ---------- 1002
        SlMLO9_LEAF  ---------- ---------- ---------- ---------- ---------- 878
      SlMLO9_FLOWER  ---------- ---------- ---------- ---------- ---------- 1002
       SlMLO9_FRUIT  ---------- ---------- ---------- ---------- ---------- 1002
                          3,160                         3,180                3,200
gDNA_Solyc06g082820  TTTTGGTTCT TCATTTTTTC AGCTTCTGCT CTCTGTGGGA ACAAAGTTGG 3200
cDNA_Solyc06g082820  ---------- ---------- --CTTCTGCT CTCTGTGGGA ACAAAGTTGG 1030
        SlMLO9_LEAF  ---------- ---------- --CTTCTGCT CTCTGTGGGA ACAAAGTTGG 906
      SlMLO9_FLOWER  ---------- ---------- --CTTCTGCT CTCTGTGGGA ACAAAGTTGG 1030
       SlMLO9_FRUIT  ---------- ---------- --CTTCTGCT CTCTGTGGGA ACAAAGTTGG 1030
                                3,220                         3,240
gDNA_Solyc06g082820  AGCATGTGAT TCTACAGTTA GCTCATGAAA TTGCTGAGAA ACATGTAGCT 3250
cDNA_Solyc06g082820  AGCATGTGAT TCTACAGTTA GCTCATGAAA TTGCTGAGAA ACATGTAGCT 1080
        SlMLO9_LEAF  AGCATGTGAT TCTACAGTTA GCTCATGAAA TTGCTGAGAA ACATGTAGCT 956
      SlMLO9_FLOWER  AGCATGTGAT TCTACAGTTA GCTCATGAAA TTGCTGAGAA ACATGTAGCT 1080
       SlMLO9_FRUIT  AGCATGTGAT TCTACAGTTA GCTCATGAAA TTGCTGAGAA ACATGTAGCT 1080
                          3,260                         3,280                3,300
gDNA_Solyc06g082820  ATAGAAGGCG AATTGGTTGT AACACCATCT GATAATCACT TTTGGTTCGA 3300
cDNA_Solyc06g082820  ATAGAAGGCG AATTGGTTGT AACACCATCT GATAATCACT TTTGGTTCGA 1130
        SlMLO9_LEAF  ATAGAAGGCG AATTGGTTGT AACACCATCT GATAATCACT TTTGGTTCGA 1006
      SlMLO9_FLOWER  ATAGAAGGCG AATTGGTTGT AACACCATCT GATAATCACT TTTGGTTCGA 1130
       SlMLO9_FRUIT  ATAGAAGGCG AATTGGTTGT AACACCATCT GATAATCACT TTTGGTTCGA 1130
                                3,320                         3,340
gDNA_Solyc06g082820  TAACCCTCAA ATCATCCTCC TCTTGATACA TTTTATTCTC TTCCAAAATG 3350
cDNA_Solyc06g082820  TAACCCTCAA ATCATCCTCC TCTTGATACA TTTTATTCTC TTCCAAAATG 1180
        SlMLO9_LEAF  TAACCCTCAA ATCATCCTCC TCTTGATACA TTTTATTCTC TTCCAAAATG 1056
      SlMLO9_FLOWER  TAACCCTCAA ATCATCCTCC TCTTGATACA TTTTATTCTC TTCCAAAATG 1180
       SlMLO9_FRUIT  TAACCCTCAA ATCATCCTCC TCTTGATACA TTTTATTCTC TTCCAAAATG 1180
                          3,360                         3,380                3,400
gDNA_Solyc06g082820  CTTTTGAAAT AGCATTTTTC TTCTGGATTT TGGTAAGTCA TTAATCAACA 3400
cDNA_Solyc06g082820  CTTTTGAAAT AGCATTTTTC TTCTGGATTT TG-------- ---------- 1212
        SlMLO9_LEAF  CTTTTGAAAT AGCATTTTTC TTCTGGATTT TG-------- ---------- 1088
      SlMLO9_FLOWER  CTTTTGAAAT AGCATTTTTC TTCTGGATTT TG-------- ---------- 1212
       SlMLO9_FRUIT  CTTTTGAAAT AGCATTTTTC TTCTGGATTT TG-------- ---------- 1212
                                3,420                         3,440
gDNA_Solyc06g082820  CCATTCTAAT TCAGTGTTCA ATACAATTTT TTCAATGTTC CTTTCCTAAA 3450
cDNA_Solyc06g082820  ---------- ---------- ---------- ---------- ---------- 1212
        SlMLO9_LEAF  ---------- ---------- ---------- ---------- ---------- 1088
      SlMLO9_FLOWER  ---------- ---------- ---------- ---------- ---------- 1212
       SlMLO9_FRUIT  ---------- ---------- ---------- ---------- ---------- 1212
                          3,460                         3,480                3,500
gDNA_Solyc06g082820  TTTCATGTTT TTTTAAAAAT AATCTTTGTA GTTCCAATAT GGCTTTCACT 3500
cDNA_Solyc06g082820  ---------- ---------- ---------- -TTCCAATAT GGCTTTCACT 1231
        SlMLO9_LEAF  ---------- ---------- ---------- -TTCCAATAT GGCTTTCACT 1107
      SlMLO9_FLOWER  ---------- ---------- ---------- -TTCCAATAT GGCTTTCACT 1231
       SlMLO9_FRUIT  ---------- ---------- ---------- -TTCCAATAT GGCTTTCACT 1231
```

```
                                          3,520                 3,540
gDNA_Solyc06g082820  CCTGCATTAT GGGAAAATAT GTCTTTGTGA TTCCACGGCT CGTCATAGGG 3550
cDNA_Solyc06g082820  CCTGCATTAT GGGAAAATAT GTCTTTGTGA TTCCACGGCT CGTCATAGGG 1281
         SlMLO9_LEAF CCTGCATTAT GGGAAAATAT GTCTTTGTGA TTCCACGGCT CGTCATAGGG 1157
       SlMLO9_FLOWER CCTGCATTAT GGGAAAATAT GTCTTTGTGA TTCCACGGCT CGTCATAGGG 1281
        SlMLO9_FRUIT CCTGCATTAT GGGAAAATAT GTCTTTGTGA TTCCACGGCT CGTCATANGG 1281
                          3,560                 3,580                 3,600
gDNA_Solyc06g082820  TAGAGTTCAC TACTCCTTCC TCTATTCATA ACTTCATATA TATATATATA 3600
cDNA_Solyc06g082820  ---------- ---------- ---------- ---------- ---------- 1281
         SlMLO9_LEAF ---------- ---------- ---------- ---------- ---------- 1157
       SlMLO9_FLOWER ---------- ---------- ---------- ---------- ---------- 1281
        SlMLO9_FRUIT ---------- ---------- ---------- ---------- ---------- 1281
                                          3,620                 3,640
gDNA_Solyc06g082820  TACGGTGTGA TTTTTCAGCG AAAGGAATTC AAATAAACCA ACCCCCTTAT 3650
cDNA_Solyc06g082820  ---------- ---------- ---------- ---------- ---------- 1281
         SlMLO9_LEAF ---------- ---------- ---------- ---------- ---------- 1157
       SlMLO9_FLOWER ---------- ---------- ---------- ---------- ---------- 1281
        SlMLO9_FRUIT ---------- ---------- ---------- ---------- ---------- 1281
                          3,660                 3,680                 3,700
gDNA_Solyc06g082820  TGCAAATTTC TTGACCCTTA ACATTCATAT ATGAAGGTGG TTTTCATCTT 3700
cDNA_Solyc06g082820  ---------- ---------- ---------- ---------- ---------- 1281
         SlMLO9_LEAF ---------- ---------- ---------- ---------- ---------- 1157
       SlMLO9_FLOWER ---------- ---------- ---------- ---------- ---------- 1281
        SlMLO9_FRUIT ---------- ---------- ---------- ---------- ---------- 1281
                                          3,720                 3,740
gDNA_Solyc06g082820  TTTCCAGGGT AATCATTCAA GTTTTATGCA GCTATAGTAC ACTGCCACTT 3750
cDNA_Solyc06g082820  --------GT AATCATTCAA GTTTTATGCA GCTATAGTAC ACTGCCACTT 1323
         SlMLO9_LEAF --------GT AATCATTCAA GTTTTATGCA GCTATAGTAC ACTGCCACTT 1199
       SlMLO9_FLOWER --------GT AATCATTCAA GTTTTATGCA GCTATAGTAC ACTGCCACTT 1323
        SlMLO9_FRUIT --------GT AATCATTCAA GTTTTATGCA GCTATAGTAC ACTGCCACTT 1323
                          3,760                 3,780                 3,800
gDNA_Solyc06g082820  TATGCTCTAG TTACACAGGT GAGTTTTTAC TCAAACCAAT CGATGAAACT 3800
cDNA_Solyc06g082820  TATGCTCTAG TTACACAG-- ---------- ---------- ---------- 1341
         SlMLO9_LEAF TATGCTCTAG TTACACAG-- ---------- ---------- ---------- 1217
       SlMLO9_FLOWER TATGCTCTAG TTACACAG-- ---------- ---------- ---------- 1341
        SlMLO9_FRUIT TATGCTCTAG TTACACAG-- ---------- ---------- ---------- 1341
                                          3,820                 3,840
gDNA_Solyc06g082820  TAAACCTGAA TGTCCTTCTC CTAATATTGT TTTTACATGA AATTATAGAT 3850
cDNA_Solyc06g082820  ---------- ---------- ---------- ---------- --------AT 1343
         SlMLO9_LEAF ---------- ---------- ---------- ---------- --------AT 1219
       SlMLO9_FLOWER ---------- ---------- ---------- ---------- --------AT 1343
        SlMLO9_FRUIT ---------- ---------- ---------- ---------- --------AT 1343
                          3,860                 3,880                 3,900
gDNA_Solyc06g082820  GGGGAGTCAT TATAAGAAAT CGATGTTCGA TAATCATGTT CAAACATGTC 3900
cDNA_Solyc06g082820  GGGGAGTCAT TATAAGAAAT CGATGTTCGA TAATCATGTT CAAACATGTC 1393
         SlMLO9_LEAF GGGGAGTCAT TATAAGAAAT CGATGTTCGA TAATCATGTT CAAACATGTC 1269
       SlMLO9_FLOWER GGGGAGTCAT TATAAGAAAT CGATGTTCGA TAATCATGTT CAAACATGTC 1393
        SlMLO9_FRUIT GGGGAGTCAT TATAAGAAAT CGATGTTCGA TAATCATGTT CAAACATGTC 1393
                                          3,920                 3,940
gDNA_Solyc06g082820  TTGTGGAATG GGCTGAGAAG GTGAAAAAGA AGAAGGGACA TAAGTATGGT 3950
cDNA_Solyc06g082820  TTGTGGAATG GGCTGAGAAG GTGAAAAAGA AGAAGGGACA TAAGTATGGT 1443
         SlMLO9_LEAF TTGTGGAATG GGCTGAGAAG GTGAAAAAGA AGAAGGGACA TAAGTATGGT 1319
       SlMLO9_FLOWER TTGTGGAATG GGCTGAGAAG GTGAAAAAGA AGAAGGGACA TAAGTATGGT 1443
        SlMLO9_FRUIT TTGTGGAATG GGCTGAGAAG GTGAAAAAGA AGAAGGGACA TAAGTATGGT 1443
                          3,960                 3,980                 4,000
gDNA_Solyc06g082820  AGAGACGGAT CTACCCGTTC AAATGACGGT TCTGTTGTTG CAGCATCACT 4000
cDNA_Solyc06g082820  AGAGACGGAT CTACCCGTTC AAATGACGGT TCTGTTGTTG CAGCATCACT 1493
         SlMLO9_LEAF AGAGACGGAT CTACCCGTTC AAATGACGGT TCTGTTGTTG CAGCATCACT 1369
       SlMLO9_FLOWER AGAGACGGAT CTACCCGTTC AAATGACGGT TCTGTTGTTG CAGCATCACT 1493
        SlMLO9_FRUIT AGAGACGGAT CTACCCGTTC AAATGACGGT TCTGTTGTTG CAGCATCACT 1493
                                          4,020                 4,040
gDNA_Solyc06g082820  GTCTGTAAAT GACCACAAAG ATCTCCCTCA AAATGGGGTT TAGCTAACAT 4050
cDNA_Solyc06g082820  GTCTGTAAAT GACCACAAAG ATCTCCCTCA AAATGGGGTT TAG------- 1536
         SlMLO9_LEAF GTCTGTAAAT GACCACAAAG ATCTCCCTCA AAATGGGGTT TAGCTAACAT 1419
       SlMLO9_FLOWER GTCTGTAAAT GACCACAAAG ATCTCCCTCA AAATGGGGTT TAG------- 1536
        SlMLO9_FRUIT GTCTGTAAAT GACCACAAAG ATCTCCCTCA AAATGGGGTT TAG------- 1536
                          4,060                 4,080                 4,100
gDNA_Solyc06g082820  TTTGTTCAAT GCCATTTGAA CAATATTTTG TAAAAGTTAG GTATGATCCA 4100
cDNA_Solyc06g082820  ---------- ---------- ---------- ---------- ---------- 1536
         SlMLO9_LEAF TTTGTTCAAT GCCATTTGAA CAATATTTTG TAAAAGTTAG GTATGATCCA 1469
       SlMLO9_FLOWER ---------- ---------- ---------- ---------- ---------- 1536
        SlMLO9_FRUIT ---------- ---------- ---------- ---------- ---------- 1536
                                          4,120                 4,140
gDNA_Solyc06g082820  GTATACCAGT TACTGAATAT ATATATGTAT GATAAGAGCG CCAAAAAGCT 4150
cDNA_Solyc06g082820  ---------- ---------- ---------- ---------- ---------- 1536
         SlMLO9_LEAF GTATAC---- ---------- ---------- ---------- ---------- 1475
       SlMLO9_FLOWER ---------- ---------- ---------- ---------- ---------- 1536
        SlMLO9_FRUIT ---------- ---------- ---------- ---------- ---------- 1536
                          4,160                 4,180                 4,200
gDNA_Solyc06g082820  TTAGATTTTT ACTGTGCAAG ATGAACTGAA ATGGAGAATG ATGGTTGGTG 4200
cDNA_Solyc06g082820  ---------- ---------- ---------- ---------- ---------- 1536
         SlMLO9_LEAF ---------- ---------- ---------- ---------- ---------- 1475
       SlMLO9_FLOWER ---------- ---------- ---------- ---------- ---------- 1536
        SlMLO9_FRUIT ---------- ---------- ---------- ---------- ---------- 1536
```

```
                                     4,220                          4,240
                                       |                              |
gDNA_Solyc06g082820  GAATTCAAAA CGTTGATCTT GTTTGGGACC GAGGCATGAT TATCTTAGGC 4250
cDNA_Solyc06g082820  ---------- ---------- ---------- ---------- ---------- 1536
       SIMLO9_LEAF   ---------- ---------- ---------- ---------- ---------- 1475
     SIMLO9_FLOWER   ---------- ---------- ---------- ---------- ---------- 1536
      SIMLO9_FRUIT   ---------- ---------- ---------- ---------- ---------- 1536
                          4,260                          4,280                       4,300
                            |                              |                           |
gDNA_Solyc06g082820  TATTGCTGAG GTGAAAGGGG GTTTTGTATA TGCGTTCCCT TGACGGTTTG 4300
cDNA_Solyc06g082820  ---------- ---------- ---------- ---------- ---------- 1536
       SIMLO9_LEAF   ---------- ---------- ---------- ---------- ---------- 1475
     SIMLO9_FLOWER   ---------- ---------- ---------- ---------- ---------- 1536
      SIMLO9_FRUIT   ---------- ---------- ---------- ---------- ---------- 1536
                                     4,320                          4,340
                                       |                              |
gDNA_Solyc06g082820  ATGTAAAATT AACTGGATAT ATTAAGTTTT ACTTGATATA AGTAAAATGT 4350
cDNA_Solyc06g082820  ---------- ---------- ---------- ---------- ---------- 1536
       SIMLO9_LEAF   ---------- ---------- ---------- ---------- ---------- 1475
     SIMLO9_FLOWER   ---------- ---------- ---------- ---------- ---------- 1536
      SIMLO9_FRUIT   ---------- ---------- ---------- ---------- ---------- 1536
                          4,360
                            |
gDNA_Solyc06g082820  ACACCATTTG CCA》4363
cDNA_Solyc06g082820  -------------》1536
       SIMLO9_LEAF   -------------》1475
     SIMLO9_FLOWER   -------------》1536
      SIMLO9_FRUIT   -------------》1536
```

```
                                    20                  40
gDNA_Solyc02g083720  CCAACCAAAT CCAGCTGATG AATACTAATG ATAAGTCATT GCTGCAGTTT 50
cDNA_Solyc02g083720  CCAACCAAAT CCAGCTGATG AATACTAATG ATAAGTCATT GCTGCAGTTT 50
SlMLO10_LEAF         ---------- ---------- ---------- ---------- ---------- -
SlMLO10_ROOTS        ---------- ---------- ---------- ---------- ---------- -
SlMLO10_FLOWER       ---------- ---------- ---------- ---------- ---------- -
SlMLO10_FRUIT        ---------- ---------- ---------- ---------- ---------- -

                          60                  80                  100
gDNA_Solyc02g083720  TTGCACTTGA AAAAAATTCC ATCTTTCTGC AACACAAATT GTTCTGTTTC 100
cDNA_Solyc02g083720  TTGCACTTGA AAAAAATTCC ATCTTTCTGC AACACAAATT GTTCTGTTTC 100
SlMLO10_LEAF         ---------- ---------- ---------- ---------- ---------- -
SlMLO10_ROOTS        ---------- ---------- ---------- ---------- ---------- -
SlMLO10_FLOWER       ---------- ---------- ---------- ---------- ---------- -
SlMLO10_FRUIT        ---------- ---------- ---------- ---------- ---------- -

                                    120                 140
gDNA_Solyc02g083720  AAGAAGATGA TGTAAATAAG GTTTATGTAG TTTTCTAAAG GTTGTTGCAT 150
cDNA_Solyc02g083720  AAGAAGATGA TGTAAATAAG GTTTATGTAG TTTTCTAAAG GTTGTTGCAT 150
SlMLO10_LEAF         ---------- ---------- ---------- ---------- ---------- -
SlMLO10_ROOTS        ---------- ---------- ---------- ---------- ---------- -
SlMLO10_FLOWER       ---------- ---------- ---------- ---------- ---------- -
SlMLO10_FRUIT        ---------- ---------- ---------- ---------- ---------- -

                          160                 180                 200
gDNA_Solyc02g083720  ACTTTACTTA TTTGTCAAGA TGGGTAATTT GGAAGGGGCG TCGTTTGCTG 200
cDNA_Solyc02g083720  ACTTTACTTA TTTGTCAAGA TGGGTAATTT GGAAGGGGCG TCGTTTGCTG 200
SlMLO10_LEAF         ---------- ---------A TGGGTAATTT GGAAGGGGCG TCGTTTGCTG 31
SlMLO10_ROOTS        ---------- ---------A TGGGTAATTT GGAAGGGGCG TCGTTTGCTG 31
SlMLO10_FLOWER       ---------- ---------A TGGGTAATTT GGAAGGGGCG TCGTTTGCTG 31
SlMLO10_FRUIT        ---------- ---------A TGGGTAATTT GGAAGGGGCG TCGTTTGCTG 31

                                    220                 240
gDNA_Solyc02g083720  AAACACCAAC ATGGGCCGTG GCAACAGTCG TAGCTGTTCT GGTGAGCATT 250
cDNA_Solyc02g083720  AAACACCAAC ATGGGCCGTG GCAACAGTCG TAGCTGTTCT GGTGAGCATT 250
SlMLO10_LEAF         AAACACCAAC ATGGGCCGTG GCAACAGTCG TAGCTGTTCT GGTGAGCATT 81
SlMLO10_ROOTS        GAACACCAAC ATGGGCCGTG GCAACAGTCG TAGCTGTTCT GGTGAGCATT 81
SlMLO10_FLOWER       AAACACCAAC ATGGGCCGTG GCAACAGTCG TAGCTGTTCT GGTGAGCATT 81
SlMLO10_FRUIT        AAACACCAAC ATGGGCCGTG GCAACAGTCG TAGCTGTTCT GGTGAGCATT 81

                          260                 280                 300
gDNA_Solyc02g083720  GGTTTCTTGA TCCATGGAAG TTTGAAGAAG TTTGGAAAGG TAAGGAAAAA 300
cDNA_Solyc02g083720  GGTTTCTTGA TCCATGGAAG TTTGAAGAAG TTTGGAAAG- ---------- 289
SlMLO10_LEAF         GGTTTCTTGA TCCATGGAAG TTTGAAGAAG TTTGGAAAG- ---------- 120
SlMLO10_ROOTS        GGTTTCTTGA TCCATGGAAG TTTGAAGAAG TTTGGAAAG- ---------- 120
SlMLO10_FLOWER       GGTTTCTTGA TCCATGGAAG TTTGAAGAAG TTTGGAAAG- ---------- 120
SlMLO10_FRUIT        GGTTTCTTGA TCCATGGAAG TTTGAAGAAG TTTGGAAAG- ---------- 120

                                    320                 340
gDNA_Solyc02g083720  AAGACTATCT TTCTTGGAAG TGAAAAATTT TCTGTTGACT TATGTGTTTT 350
cDNA_Solyc02g083720  ---------- ---------- ---------- ---------- ---------- 289
SlMLO10_LEAF         ---------- ---------- ---------- ---------- ---------- 120
SlMLO10_ROOTS        ---------- ---------- ---------- ---------- ---------- 120
SlMLO10_FLOWER       ---------- ---------- ---------- ---------- ---------- 120
SlMLO10_FRUIT        ---------- ---------- ---------- ---------- ---------- 120

                          360                 380                 400
gDNA_Solyc02g083720  GTTGTTATAG TGGTTGCATA GGACAAAGAG GGAACCTCTG TATGCTGCAC 400
cDNA_Solyc02g083720  ---------- TGGTTGCATA GGACAAAGAG GGAACCTCTG TATGCTGCAC 329
SlMLO10_LEAF         ---------- TGGTTGCATA GGACAAAGAG GGAACCTCTG TATGCTGCAC 160
SlMLO10_ROOTS        ---------- TGGTTGCATA GGACAAAGAG GGAACCTCTG TATGCTGCAC 160
SlMLO10_FLOWER       ---------- TGGTTGCATA GGACAAAGAG GGAACCTCTG TATGCTGCAC 160
SlMLO10_FRUIT        ---------- TGGTTGCATA GGACAAAGAG GGAACCTCTG TATGCTGCAC 160

                                    420                 440
gDNA_Solyc02g083720  TAGAGAAAAT CAAGGAAGGT AAGCCACCTA TTAATCATCT GTATATTGAG 450
cDNA_Solyc02g083720  TAGAGAAAAT CAAGGAAG-- ---------- ---------- ---------- 347
SlMLO10_LEAF         TAGAGAAAAT CAAGGAAG-- ---------- ---------- ---------- 178
SlMLO10_ROOTS        TAGAGAAAAT CAAGGAAG-- ---------- ---------- ---------- 178
SlMLO10_FLOWER       TAGAGAAAAT CAAGGAAG-- ---------- ---------- ---------- 178
SlMLO10_FRUIT        TAGAGAAAAT CAAGGAAG-- ---------- ---------- ---------- 178

                          460                 480                 500
gDNA_Solyc02g083720  TCTTTCTTGA TCACTGCATA TATAGAAGGT TTCTGCAGAT AATTTTTTAC 500
cDNA_Solyc02g083720  ---------- ---------- ---------- ---------- ---------- 347
SlMLO10_LEAF         ---------- ---------- ---------- ---------- ---------- 178
SlMLO10_ROOTS        ---------- ---------- ---------- ---------- ---------- 178
SlMLO10_FLOWER       ---------- ---------- ---------- ---------- ---------- 178
SlMLO10_FRUIT        ---------- ---------- ---------- ---------- ---------- 178

                                    520                 540
gDNA_Solyc02g083720  TTGAACGTCG AATATACTAT ATGGAAATAA GTAGGAATAG AGTTATCGGT 550
cDNA_Solyc02g083720  ---------- ---------- ---------- ---------- ---------- 347
SlMLO10_LEAF         ---------- ---------- ---------- ---------- ---------- 178
SlMLO10_ROOTS        ---------- ---------- ---------- ---------- ---------- 178
SlMLO10_FLOWER       ---------- ---------- ---------- ---------- ---------- 178
SlMLO10_FRUIT        ---------- ---------- ---------- ---------- ---------- 178

                          560                 580                 600
gDNA_Solyc02g083720  TATAGAAAAT TGTACATATT AAGTGTGTTC TTAAACACAA ATATAGAGTT 600
cDNA_Solyc02g083720  ---------- ---------- ---------- ---------- ---------- 347
SlMLO10_LEAF         ---------- ---------- ---------- ---------- ---------- 178
SlMLO10_ROOTS        ---------- ---------- ---------- ---------- ---------- 178
SlMLO10_FLOWER       ---------- ---------- ---------- ---------- ---------- 178
SlMLO10_FRUIT        ---------- ---------- ---------- ---------- ---------- 178
```

```
                                     620                           640
gDNA_Solyc02g083720  TGAGCTAAAG CTGTTGATGT CAGAACTTAC ACCTCCAGTC TAATAAAACC 650
cDNA_Solyc02g083720  ---------- ---------- ---------- ---------- ---------- 347
       SIMLO10_LEAF  ---------- ---------- ---------- ---------- ---------- 178
      SIMLO10_ROOTS  ---------- ---------- ---------- ---------- ---------- 178
     SIMLO10_FLOWER  ---------- ---------- ---------- ---------- ---------- 178
      SIMLO10_FRUIT  ---------- ---------- ---------- ---------- ---------- 178

                          660                           680                700
gDNA_Solyc02g083720  TAAACAGGTG GTGTTGGTTC TTCTAAACGC TACTGCTTTT TTGCGCTAAA 700
cDNA_Solyc02g083720  ---------- ---------- ---------- ---------- ---------- 347
       SIMLO10_LEAF  ---------- ---------- ---------- ---------- ---------- 178
      SIMLO10_ROOTS  ---------- ---------- ---------- ---------- ---------- 178
     SIMLO10_FLOWER  ---------- ---------- ---------- ---------- ---------- 178
      SIMLO10_FRUIT  ---------- ---------- ---------- ---------- ---------- 178

                                     720                           740
gDNA_Solyc02g083720  GTGGAGGGAT CCTATCCATT CCACTACAAT TTATGATTGC TTGATGAACT 750
cDNA_Solyc02g083720  ---------- ---------- ---------- ---------- ---------- 347
       SIMLO10_LEAF  ---------- ---------- ---------- ---------- ---------- 178
      SIMLO10_ROOTS  ---------- ---------- ---------- ---------- ---------- 178
     SIMLO10_FLOWER  ---------- ---------- ---------- ---------- ---------- 178
      SIMLO10_FRUIT  ---------- ---------- ---------- ---------- ---------- 178

                          760                           780                800
gDNA_Solyc02g083720  CATTTGTTTT GCTTTCTCTT CCCTTTTTTT GTGCGGCAAG CAGAGCTTAT 800
cDNA_Solyc02g083720  ---------- ---------- ---------- ---------- ---AGCTTAT 354
       SIMLO10_LEAF  ---------- ---------- ---------- ---------- ---AGCTTAT 185
      SIMLO10_ROOTS  ---------- ---------- ---------- ---------- ---AGCTTAT 185
     SIMLO10_FLOWER  ---------- ---------- ---------- ---------- ---AGCTTAT 185
      SIMLO10_FRUIT  ---------- ---------- ---------- ---------- ---AGCTTAT 185

                                     820                           840
gDNA_Solyc02g083720  GGTTTTTGGA CTGCTCTCAC TGCTTATGGG TCATTGGATC GTTTATATTG 850
cDNA_Solyc02g083720  GGTTTTTGGA CTGCTCTCAC TGCTTATGGG TCATTGGATC GTTTATATTG 404
       SIMLO10_LEAF  GGTTTTTGGA CTGCTCTCAC TGCTTATGGG TCATTGGATC GTTTATATTG 235
      SIMLO10_ROOTS  GGTTTTTGGA CTGCTCTCAC TGCTTATGGG TCATTGGATC GTTTATATTG 235
     SIMLO10_FLOWER  GGTTTTTGGA CTGCTCTCAC TGCTTATGGG TCATTGGATC GTTTATATTG 235
      SIMLO10_FRUIT  GGTTTCTGGA CTGCTCTCAC TGCTTATGGG TCATTGGATC GTTTATATTG 235

                          860                           880                900
gDNA_Solyc02g083720  CAAAGATTTG TGTCAAATCG TCAGCAGTGA GCAGCCACTT TTATCCCTGT 900
cDNA_Solyc02g083720  CAAAGATTTG TGTCAAATCG TCAGCAGTGA GCAGCCACTT TTATCCCTGT 454
       SIMLO10_LEAF  CAAAGATTTG TGTCAAATCG TCAGCAGTGA GCAGCCACTT TTATCCCTGT 285
      SIMLO10_ROOTS  CAAAGATTTG TGTCAAATCG TCAGCAGTGA GCAGCCACTT TTATCCCTGT 285
     SIMLO10_FLOWER  CAAAGATTTG TGTCAAATCG TCAGCAGTGA GCAGCCACTT TTATCCCTGT 285
      SIMLO10_FRUIT  CAAAGATTTG TGTCAAATCG TCAGCAGTGA GCAGCCACTT TTATCCCTGT 285

                                     920                           940
gDNA_Solyc02g083720  TCTCCGCCAA GAAACAAGAT GAAGTCAGCA ATTACAAGAT TTGCTTTATC 950
cDNA_Solyc02g083720  TCTCCGCCAA GAAACAAGAT GAAGTCAGCA ATTACAAGAT TTGCTTTATC 504
       SIMLO10_LEAF  TCTCCGCCAA GAAACAAGAT GAAGTCAGCA ATTACAAGAT TTGCTTTATC 335
      SIMLO10_ROOTS  TCTCCGCCAA GAAACAAGAT GAAGTCAGCA ATTACAAGAT TTGCTTTATC 335
     SIMLO10_FLOWER  TCTCCGCCAA GAAACAAGAT GAAGTCAGCA ATTACAAGAT TTGCTTTATC 335
      SIMLO10_FRUIT  TCTCCGCCAA GAAACAAGAT GAAGTCAGCA ATTACAAGAT TTGCTTTATC 335

                          960                           980              1,000
gDNA_Solyc02g083720  AGGTTCTTCA TACTCGAATT TCTCAACATC AAGGCTACTG TTAAGCAGTG 1000
cDNA_Solyc02g083720  AGGTTCTTCA TACTCGAATT TCTCAACATC AAGGCTACTG TTAAGCAGTG 554
       SIMLO10_LEAF  AGGTTCTTCA TACTCGAATT TCTCAACATC AAGGCTACTG TTAAGCAGTG 385
      SIMLO10_ROOTS  AGGTTCTTCA TACTCGAATT TCTCAACATC AAGGCTACTG TTAAGCAGTG 385
     SIMLO10_FLOWER  AGGTTCTTCA TACTCGAATT TCTCAACATC AAGGCTACTG TTAAGCAGTG 385
      SIMLO10_FRUIT  AGGTTCTTCA TACTCGAATT TCTCAACATC AAGGCTACTG TTAAGCAGTG 385

                                   1,020                         1,040
gDNA_Solyc02g083720  GACATGAAGA TTTTTGTCCT GAGGTAGCCC AGTAATGAAT GTTCCACTCA 1050
cDNA_Solyc02g083720  GACATGAAGA TTTTTGTCCT GAGG------ ---------- ---------- 578
       SIMLO10_LEAF  GACATGAAGA TTTTTGTCCT GAGG------ ---------- ---------- 409
      SIMLO10_ROOTS  GACATGAAGA TTTTTGTCCT GAGG------ ---------- ---------- 409
     SIMLO10_FLOWER  GACATGAAGA TTTTTGTCCT GAGG------ ---------- ---------- 409
      SIMLO10_FRUIT  GACATGAAGA TTTTTGTCCT GAGG------ ---------- ---------- 409

                        1,060                         1,080              1,100
gDNA_Solyc02g083720  AAATTTAATC TGACATTTAC TTTTAACAGT TTTTGGTTGA TCTGTCACAA 1100
cDNA_Solyc02g083720  ---------- ---------- ---------- ---------- ---------- 578
       SIMLO10_LEAF  ---------- ---------- ---------- ---------- ---------- 409
      SIMLO10_ROOTS  ---------- ---------- ---------- ---------- ---------- 409
     SIMLO10_FLOWER  ---------- ---------- ---------- ---------- ---------- 409
      SIMLO10_FRUIT  ---------- ---------- ---------- ---------- ---------- 409

                                   1,120                         1,140
gDNA_Solyc02g083720  GCTATCATCA ACTGAAAATA ACTAATGCTA CCGTGGAATC TCTAGTCTAT 1150
cDNA_Solyc02g083720  ---------- ---------- ---------- ---------- ---------- 578
       SIMLO10_LEAF  ---------- ---------- ---------- ---------- ---------- 409
      SIMLO10_ROOTS  ---------- ---------- ---------- ---------- ---------- 409
     SIMLO10_FLOWER  ---------- ---------- ---------- ---------- ---------- 409
      SIMLO10_FRUIT  ---------- ---------- ---------- ---------- ---------- 409

                        1,160                         1,180              1,200
gDNA_Solyc02g083720  TGTTGTAAGC TAAAATTTGT CACATGAGAT TGTTCTATGT AAATCCTAAA 1200
cDNA_Solyc02g083720  ---------- ---------- ---------- ---------- ---------- 578
       SIMLO10_LEAF  ---------- ---------- ---------- ---------- ---------- 409
      SIMLO10_ROOTS  ---------- ---------- ---------- ---------- ---------- 409
     SIMLO10_FLOWER  ---------- ---------- ---------- ---------- ---------- 409
      SIMLO10_FRUIT  ---------- ---------- ---------- ---------- ---------- 409
```

```
                                                   1,220                1,240
gDNA_Solyc02g083720  TGTAGCATAA TTTATAGTCA AATGTTTTCG TCCAGTGAAG TTCCATGATA 1250
cDNA_Solyc02g083720  ---------- ---------- ---------- ---------- ---------- 578
       SlMLO10_LEAF  ---------- ---------- ---------- ---------- ---------- 409
      SlMLO10_ROOTS  ---------- ---------- ---------- ---------- ---------- 409
     SlMLO10_FLOWER  ---------- ---------- ---------- ---------- ---------- 409
      SlMLO10_FRUIT  ---------- ---------- ---------- ---------- ---------- 409
                              1,260                1,280                1,300
gDNA_Solyc02g083720  TTACTACATC TAATACTCAT CAATTACAAG ATTGGTCGCG CTAAGAAGCA 1300
cDNA_Solyc02g083720  ---------- ---------- ---------- ---------- ---------- 578
       SlMLO10_LEAF  ---------- ---------- ---------- ---------- ---------- 409
      SlMLO10_ROOTS  ---------- ---------- ---------- ---------- ---------- 409
     SlMLO10_FLOWER  ---------- ---------- ---------- ---------- ---------- 409
      SlMLO10_FRUIT  ---------- ---------- ---------- ---------- ---------- 409
                                         1,320                1,340
gDNA_Solyc02g083720  AATTTTTTTG GAAGTTTAAC GTGCCTTGAA CTTGAAAATA GGACTCATTT 1350
cDNA_Solyc02g083720  ---------- ---------- ---------- ---------- ---------- 578
       SlMLO10_LEAF  ---------- ---------- ---------- ---------- ---------- 409
      SlMLO10_ROOTS  ---------- ---------- ---------- ---------- ---------- 409
     SlMLO10_FLOWER  ---------- ---------- ---------- ---------- ---------- 409
      SlMLO10_FRUIT  ---------- ---------- ---------- ---------- ---------- 409
                              1,360                1,380                1,400
gDNA_Solyc02g083720  AGACAAAGAC TCCTCCGGAC TCCCTAGGTC AGGCAACTAG TGAAGCTTGT 1400
cDNA_Solyc02g083720  ---------- ---------- ---------- ---------- ---------- 578
       SlMLO10_LEAF  ---------- ---------- ---------- ---------- ---------- 409
      SlMLO10_ROOTS  ---------- ---------- ---------- ---------- ---------- 409
     SlMLO10_FLOWER  ---------- ---------- ---------- ---------- ---------- 409
      SlMLO10_FRUIT  ---------- ---------- ---------- ---------- ---------- 409
                                         1,420                1,440
gDNA_Solyc02g083720  CTTTAGGAAG AAAAAAGAAA GGAAGTTCTC TCACATTTTC TCATTTCATT 1450
cDNA_Solyc02g083720  ---------- ---------- ---------- ---------- ---------- 578
       SlMLO10_LEAF  ---------- ---------- ---------- ---------- ---------- 409
      SlMLO10_ROOTS  ---------- ---------- ---------- ---------- ---------- 409
     SlMLO10_FLOWER  ---------- ---------- ---------- ---------- ---------- 409
      SlMLO10_FRUIT  ---------- ---------- ---------- ---------- ---------- 409
                              1,460                1,480                1,500
gDNA_Solyc02g083720  TAATTCTCTT TGCAAGTTTC AAATTGCTTT AGTAAAGTGG CCAGGGCTTG 1500
cDNA_Solyc02g083720  ---------- ---------- ---------- ---------- ---------- 578
       SlMLO10_LEAF  ---------- ---------- ---------- ---------- ---------- 409
      SlMLO10_ROOTS  ---------- ---------- ---------- ---------- ---------- 409
     SlMLO10_FLOWER  ---------- ---------- ---------- ---------- ---------- 409
      SlMLO10_FRUIT  ---------- ---------- ---------- ---------- ---------- 409
                                         1,520                1,540
gDNA_Solyc02g083720  TACTGGATTC ATATGAGCAG AGAACACTCA TGTTGATTTT ACCAGGGTCT 1550
cDNA_Solyc02g083720  ---------- ---------- ---------- ---------- ------GTCT 582
       SlMLO10_LEAF  ---------- ---------- ---------- ---------- ------GTCC 413
      SlMLO10_ROOTS  ---------- ---------- ---------- ---------- ------GTCT 413
     SlMLO10_FLOWER  ---------- ---------- ---------- ---------- ------GTCT 413
      SlMLO10_FRUIT  ---------- ---------- ---------- ---------- ------GTCT 413
                              1,560                1,580                1,600
gDNA_Solyc02g083720  CCAATCGTTT GCTTCGAAGG AGAGCCTGGA GCAGCTCCAT CGCTTTTTGC 1600
cDNA_Solyc02g083720  CCAATCGTTT GCTTCGAAGG AGAGCCTGGA GCAGCTCCAT CGCTTTTTGC 632
       SlMLO10_LEAF  CCAATCGTTT GCTTCGAAGG GGAGCCTGGA GCAGCTCCAT CGCTTTTTGC 463
      SlMLO10_ROOTS  CCAATCGTTT GCTTCGAAGG AGAGCCTGGA GCAGCTCCAT CGCTTTTTGC 463
     SlMLO10_FLOWER  CCAATCGTTT GCTTCGAAGG AGAGCCTGGA GCAGCTCCAT CGCTTTTTGC 463
      SlMLO10_FRUIT  CCAATCGTTT GCTTCGAAGG AGAGCCTGGA GCAGCTCCAT CGCTTTTTGC 463
                                         1,620                1,640
gDNA_Solyc02g083720  TTGTCCTCGG TGTTAGCCAT GTATCGTATA GCTTTTTTGC CATTGCCCTG 1650
cDNA_Solyc02g083720  TTGTCCTCGG TGTTAGCCAT GTATCGTATA GCTTTTTTGC CATTGCCCTG 682
       SlMLO10_LEAF  TTGTCCTCGG TGTTAGCCAT GTATCGTATA GCTTTTTTGC CATTGCCCTG 513
      SlMLO10_ROOTS  TTGTCCTCGG TGTTAGCCAT GTATCGTATA GCTTTTTTGC CATTGCCCTG 513
     SlMLO10_FLOWER  TTGTCCTCGG TGTTAGCCAT GTATCGTATA GCTTTTTTGC CATTGCCCTG 513
      SlMLO10_FRUIT  TTGTCCTCGG TGTTAGCCAT GTATCGTATA GCTTTTTTGC CATTGCCCTG 513
                              1,660                1,680                1,700
gDNA_Solyc02g083720  GCAATGATCA AGGTTGGATA TCTTCTTCAT GATTAACCTC TTATTACATC 1700
cDNA_Solyc02g083720  GCAATGATCA AG-------- ---------- ---------- ---------- 694
       SlMLO10_LEAF  GCAATGATCA AG-------- ---------- ---------- ---------- 525
      SlMLO10_ROOTS  GCAATGATCA AG-------- ---------- ---------- ---------- 525
     SlMLO10_FLOWER  GCAATGATCA AG-------- ---------- ---------- ---------- 525
      SlMLO10_FRUIT  GCAATGATCA AG-------- ---------- ---------- ---------- 525
                                         1,720                1,740
gDNA_Solyc02g083720  TCAAAACTTT CTTCTCAAAT CCATCAAGAA ACATGAATTA ACCATGCAGA 1750
cDNA_Solyc02g083720  ---------- ---------- ---------- ---------- ---------A 695
       SlMLO10_LEAF  ---------- ---------- ---------- ---------- ---------A 526
      SlMLO10_ROOTS  ---------- ---------- ---------- ---------- ---------A 526
     SlMLO10_FLOWER  ---------- ---------- ---------- ---------- ---------A 526
      SlMLO10_FRUIT  ---------- ---------- ---------- ---------- ---------A 526
                              1,760                1,780                1,800
gDNA_Solyc02g083720  TATATAGCTG GAGAACATGG GAGAACTATG CCAAGTCGAT TGCTCTTCAA 1800
cDNA_Solyc02g083720  TATATAGCTG GAGAACATGG GAGAACTATG CCAAGTCGAT TGCTCTTCAA 745
       SlMLO10_LEAF  TATATAGCTG GAGAACATGG GAGAACTATG CCAAGTCGAT TGCTCTTCAA 576
      SlMLO10_ROOTS  TATATAGCTG GAGAACATGG GAGAACTATG CCAAGTCGAT TGCTCTTCAA 576
     SlMLO10_FLOWER  TATATAGCTG GAGAACATGG GAGAACTATG CCAAGTCGAT TGCTCTTCAA 576
      SlMLO10_FRUIT  TATATAGCTA GAGAACATGG GAGAACTATG CCAAGTCGAT TGCTCTTCAA 576
```

```
                                           1,820                           1,840
gDNA_Solyc02g083720  AGACTAGAAG GTGCTAAAAT TTCCAGCCAT ACTACTATTC TTCATACTTT 1850
cDNA_Solyc02g083720  AGACTAGAAG GT-------- ---------- ---------- ---------- 757
        SlMLO10_LEAF  AGACTAGAAG GT-------- ---------- ---------- ---------- 588
       SlMLO10_ROOTS  AGACTAGAAG GT-------- ---------- ---------- ---------- 588
      SlMLO10_FLOWER  AGACTAGAAG GT-------- ---------- ---------- ---------- 588
       SlMLO10_FRUIT  AGACTAGAAG GT-------- ---------- ---------- ---------- 588
                               1,860                           1,880                   1,900
gDNA_Solyc02g083720  ATTTATCATT TCCAGGACAC TTACTTGCAA GTTTAACCTT AGGTTCTGAA 1900
cDNA_Solyc02g083720  ---------- ---------- ---------- ---------- ----TCTGAA 763
        SlMLO10_LEAF  ---------- ---------- ---------- ---------- ----TCTGAA 594
       SlMLO10_ROOTS  ---------- ---------- ---------- ---------- ----TCTGAA 594
      SlMLO10_FLOWER  ---------- ---------- ---------- ---------- ----TCTGAA 594
       SlMLO10_FRUIT  ---------- ---------- ---------- ---------- ----TCTGAA 594
                                           1,920                           1,940
gDNA_Solyc02g083720  GAAGCTGTCC CAAACAATAC GAGAATGGGA CGTCTATCAA CTTTTACTTT 1950
cDNA_Solyc02g083720  GAAGCTGTCC CAAACAATAC GAGAATGGGA CGTCTATCAA CTTTTACTTT 813
        SlMLO10_LEAF  GAAGCTGTCC CAAACAATAC GAGAATGGGA CGTCTATCAA CTTTTACTTT 644
       SlMLO10_ROOTS  GAAGCTGTCC CAAACAATAC GAGAATGGGA CGTCTATCAA CTTTTACTTT 644
      SlMLO10_FLOWER  GAAGCTGTCC CAAACAATAC GAGAATGGGA CGTCTATCAA CTTTTACTTT 644
       SlMLO10_FRUIT  GAAGCTGTCC CAAACAATAC GAGAATGGGA CGTCTATCAA CTTTTACTTT 644
                               1,960                           1,980                   2,000
gDNA_Solyc02g083720  TCACCAGACC ACTCATCCAT GGAGCCAGCA CAGAGCTCTT GTTTGGCTGG 2000
cDNA_Solyc02g083720  TCACCAGACC ACTCATCCAT GGAGCCAGCA CAGAGCTCTT GTTTGGCTG- 862
        SlMLO10_LEAF  TCACCAGACC ACTCATCCAT GGAGCCAGCA CAGAGCTCTT GTTTGGCTG- 693
       SlMLO10_ROOTS  TCACCAGACC ACTCATCCAT GGAGCCAGCA CAGAGCTCTT GTTTGGCTG- 693
      SlMLO10_FLOWER  TCACCAGACC ACTCATCCAT GGAGCCAGCA CAGAGCTCTT GTTTGGCTG- 693
       SlMLO10_FRUIT  TCACCAGACC ACTCATCCAT GGAGCCAGCA CAGAGCTCTT GTTTGGCTG- 693
                                           2,020                           2,040
gDNA_Solyc02g083720  TATTTCACGA CGAATCTTGT TTATCTGGTT GAGTGAATCC TTGTTATGTG 2050
cDNA_Solyc02g083720  ---------- ---------- ---------- ---------- ---------- 862
        SlMLO10_LEAF  ---------- ---------- ---------- ---------- ---------- 693
       SlMLO10_ROOTS  ---------- ---------- ---------- ---------- ---------- 693
      SlMLO10_FLOWER  ---------- ---------- ---------- ---------- ---------- 693
       SlMLO10_FRUIT  ---------- ---------- ---------- ---------- ---------- 693
                               2,060                           2,080                   2,100
gDNA_Solyc02g083720  AAATGCTTCT TCTATCTCAA TTTAAGGGGA GTTATCATAG CCCAGTGGTG 2100
cDNA_Solyc02g083720  ---------- ---------- ---------- ---------- ---------- 862
        SlMLO10_LEAF  ---------- ---------- ---------- ---------- ---------- 693
       SlMLO10_ROOTS  ---------- ---------- ---------- ---------- ---------- 693
      SlMLO10_FLOWER  ---------- ---------- ---------- ---------- ---------- 693
       SlMLO10_FRUIT  ---------- ---------- ---------- ---------- ---------- 693
                                           2,120                           2,140
gDNA_Solyc02g083720  GAGGTATCTC AGTCATTGAA AAGGGTTCTG GACAACTGTG TACATTTGTT 2150
cDNA_Solyc02g083720  ---------- ---------- ---------- ---------- ---------- 862
        SlMLO10_LEAF  ---------- ---------- ---------- ---------- ---------- 693
       SlMLO10_ROOTS  ---------- ---------- ---------- ---------- ---------- 693
      SlMLO10_FLOWER  ---------- ---------- ---------- ---------- ---------- 693
       SlMLO10_FRUIT  ---------- ---------- ---------- ---------- ---------- 693
                               2,160                           2,180                   2,200
gDNA_Solyc02g083720  CTGGACAAGT GCGGGGAGAA GTGTTAACAT GTACACAATA AGAAGATGAA 2200
cDNA_Solyc02g083720  ---------- ---------- ---------- ---------- ---------- 862
        SlMLO10_LEAF  ---------- ---------- ---------- ---------- ---------- 693
       SlMLO10_ROOTS  ---------- ---------- ---------- ---------- ---------- 693
      SlMLO10_FLOWER  ---------- ---------- ---------- ---------- ---------- 693
       SlMLO10_FRUIT  ---------- ---------- ---------- ---------- ---------- 693
                                           2,220                           2,240
gDNA_Solyc02g083720  AAACTAAAAA TTAATATAGA ATATCAAATT CAAGGTTGCT TTTGCAGCTA 2250
cDNA_Solyc02g083720  ---------- ---------- ---------- ---------- ---------- 862
        SlMLO10_LEAF  ---------- ---------- ---------- ---------- ---------- 693
       SlMLO10_ROOTS  ---------- ---------- ---------- ---------- ---------- 693
      SlMLO10_FLOWER  ---------- ---------- ---------- ---------- ---------- 693
       SlMLO10_FRUIT  ---------- ---------- ---------- ---------- ---------- 693
                               2,260                           2,280                   2,300
gDNA_Solyc02g083720  AAACTATGAT TTAGGGTGAT ACTAATCGGC AATGTCATTC ACTAATTCTA 2300
cDNA_Solyc02g083720  ---------- ---------- ---------- ---------- ---------- 862
        SlMLO10_LEAF  ---------- ---------- ---------- ---------- ---------- 693
       SlMLO10_ROOTS  ---------- ---------- ---------- ---------- ---------- 693
      SlMLO10_FLOWER  ---------- ---------- ---------- ---------- ---------- 693
       SlMLO10_FRUIT  ---------- ---------- ---------- ---------- ---------- 693
                                           2,320                           2,340
gDNA_Solyc02g083720  TGACTATGCA AACAATCATT GTTCACTCAG GTCCAAGTCA CAAATCTTAA 2350
cDNA_Solyc02g083720  ---------- ---------- ---------- ---------- ---------- 862
        SlMLO10_LEAF  ---------- ---------- ---------- ---------- ---------- 693
       SlMLO10_ROOTS  ---------- ---------- ---------- ---------- ---------- 693
      SlMLO10_FLOWER  ---------- ---------- ---------- ---------- ---------- 693
       SlMLO10_FRUIT  ---------- ---------- ---------- ---------- ---------- 693
                               2,360                           2,380                   2,400
gDNA_Solyc02g083720  CAATACTGTA GAATTATGTT TAGGTCTTAC ATTTGCTGTT ACTTTAATTC 2400
cDNA_Solyc02g083720  ---------- ---------- ---------- ---------- ---------- 862
        SlMLO10_LEAF  ---------- ---------- ---------- ---------- ---------- 693
       SlMLO10_ROOTS  ---------- ---------- ---------- ---------- ---------- 693
      SlMLO10_FLOWER  ---------- ---------- ---------- ---------- ---------- 693
       SlMLO10_FRUIT  ---------- ---------- ---------- ---------- ---------- 693
```

```
                                         2,420                         2,440
                                           |                             |
gDNA_Solyc02g083720  ACGTATTCAG AAAGAATTAT TGTAAACCAG TGCATCAAAG CATATTCATA 2450
cDNA_Solyc02g083720  ---------- ---------- ---------- ---------- ---------- 862
       SIMLO10_LEAF  ---------- ---------- ---------- ---------- ---------- 693
      SIMLO10_ROOTS  ---------- ---------- ---------- ---------- ---------- 693
     SIMLO10_FLOWER  ---------- ---------- ---------- ---------- ---------- 693
      SIMLO10_FRUIT  ---------- ---------- ---------- ---------- ---------- 693

                              2,460                          2,480                       2,500
                                |                              |                           |
gDNA_Solyc02g083720  TGTCCTGCTT GTATTGGCAT CAATCAAATT TAATAATTTT CTCTTATCAT 2500
cDNA_Solyc02g083720  ---------- ---------- ---------- ---------- ---------- 862
       SIMLO10_LEAF  ---------- ---------- ---------- ---------- ---------- 693
      SIMLO10_ROOTS  ---------- ---------- ---------- ---------- ---------- 693
     SIMLO10_FLOWER  ---------- ---------- ---------- ---------- ---------- 693
      SIMLO10_FRUIT  ---------- ---------- ---------- ---------- ---------- 693

                                         2,520                         2,540
                                           |                             |
gDNA_Solyc02g083720  TATGTACGTC TGACGAAAAT TTCACCTTCA TGCTTTAATC TTCACAACCT 2550
cDNA_Solyc02g083720  ---------- ---------- ---------- ---------- ---------- 862
       SIMLO10_LEAF  ---------- ---------- ---------- ---------- ---------- 693
      SIMLO10_ROOTS  ---------- ---------- ---------- ---------- ---------- 693
     SIMLO10_FLOWER  ---------- ---------- ---------- ---------- ---------- 693
      SIMLO10_FRUIT  ---------- ---------- ---------- ---------- ---------- 693

                              2,560                          2,580                       2,600
                                |                              |                           |
gDNA_Solyc02g083720  CTTGAGTCTT GACAAGTGAT GCAATGAGTC CAGTGATTCA AGATATTTAA 2600
cDNA_Solyc02g083720  ---------- ---------- ---------- ---------- ---------- 862
       SIMLO10_LEAF  ---------- ---------- ---------- ---------- ---------- 693
      SIMLO10_ROOTS  ---------- ---------- ---------- ---------- ---------- 693
     SIMLO10_FLOWER  ---------- ---------- ---------- ---------- ---------- 693
      SIMLO10_FRUIT  ---------- ---------- ---------- ---------- ---------- 693

                                         2,620                         2,640
                                           |                             |
gDNA_Solyc02g083720  TTCCATATTC CCTTTTTAGC TTTGTTTCAG CCGCCAGTTC TGGAGTTCTA 2650
cDNA_Solyc02g083720  ---------- ---------C TTTGTTTCAG CCGCCAGTTC TGGAGTTCTA 893
       SIMLO10_LEAF  ---------- ---------C TTTGTTTCAG CCGCCAGTTC TGGAGTTCTA 724
      SIMLO10_ROOTS  ---------- ---------C TTTGTTTCAG CCGCCAGTTC TGGAGTTCTA 724
     SIMLO10_FLOWER  ---------- ---------C TTTGTTTCAG CCGCCAGTTC TGGAGTTCTA 724
      SIMLO10_FRUIT  ---------- ---------C TTTGTTTCAG CCGCCAGTTC TGGAGTTCTA 724

                              2,660                          2,680                       2,700
                                |                              |                           |
gDNA_Solyc02g083720  TAAATGAAGC TGACTACATG GCTTTGCGCT TGGGTTTTAT TACTGTAAGT 2700
cDNA_Solyc02g083720  TAAATGAAGC TGACTACATG GCTTTGCGCT TGGGTTTTAT TACT------ 937
       SIMLO10_LEAF  TAAATGAAGC TGACTACATG GCTTTGCGCT TGGGTTTTAT TACT------ 768
      SIMLO10_ROOTS  TAAATGAAGC TGACTACATG GCTTTGCGCT TGGGTTTTAT TACT------ 768
     SIMLO10_FLOWER  TAAATGAAGC TGACTACATG GCTTTGCGCT TGGGTTTTAT TACT------ 768
      SIMLO10_FRUIT  TAAATGAAGC TGACTACATG GCTTTGCGCT TGGGTTTTAT TACT------ 768

                                         2,720                         2,740
                                           |                             |
gDNA_Solyc02g083720  CATGTATTGT TATACTTCAC AAATGCAAAT ATTTCACTCA ATCGTCATTA 2750
cDNA_Solyc02g083720  ---------- ---------- ---------- ---------- ---------- 937
       SIMLO10_LEAF  ---------- ---------- ---------- ---------- ---------- 768
      SIMLO10_ROOTS  ---------- ---------- ---------- ---------- ---------- 768
     SIMLO10_FLOWER  ---------- ---------- ---------- ---------- ---------- 768
      SIMLO10_FRUIT  ---------- ---------- ---------- ---------- ---------- 768

                              2,760                          2,780                       2,800
                                |                              |                           |
gDNA_Solyc02g083720  AACTTGGAAG TGAAGACTGA CTGACTTTCT ATGGCTGACT TCATGCTGCC 2800
cDNA_Solyc02g083720  ---------- ---------- ---------- ---------- ---------- 937
       SIMLO10_LEAF  ---------- ---------- ---------- ---------- ---------- 768
      SIMLO10_ROOTS  ---------- ---------- ---------- ---------- ---------- 768
     SIMLO10_FLOWER  ---------- ---------- ---------- ---------- ---------- 768
      SIMLO10_FRUIT  ---------- ---------- ---------- ---------- ---------- 768

                                         2,820                         2,840
                                           |                             |
gDNA_Solyc02g083720  CCCGTTTATA ACAATATAAA TCAGTTATTA ATCATATTTG AATTTTGGTT 2850
cDNA_Solyc02g083720  ---------- ---------- ---------- ---------- ---------- 937
       SIMLO10_LEAF  ---------- ---------- ---------- ---------- ---------- 768
      SIMLO10_ROOTS  ---------- ---------- ---------- ---------- ---------- 768
     SIMLO10_FLOWER  ---------- ---------- ---------- ---------- ---------- 768
      SIMLO10_FRUIT  ---------- ---------- ---------- ---------- ---------- 768

                              2,860                          2,880                       2,900
                                |                              |                           |
gDNA_Solyc02g083720  ATTTTATGAA AATGACTCTT TAAGCTAATA ACCACTTAAT ACAACATTCA 2900
cDNA_Solyc02g083720  ---------- ---------- ---------- ---------- ---------- 937
       SIMLO10_LEAF  ---------- ---------- ---------- ---------- ---------- 768
      SIMLO10_ROOTS  ---------- ---------- ---------- ---------- ---------- 768
     SIMLO10_FLOWER  ---------- ---------- ---------- ---------- ---------- 768
      SIMLO10_FRUIT  ---------- ---------- ---------- ---------- ---------- 768

                                         2,920                         2,940
                                           |                             |
gDNA_Solyc02g083720  CTTATGTAGA TGATGTGAAT AAATACCAAG TAGGCCCTAA TTTTGGTACT 2950
cDNA_Solyc02g083720  ---------- ---------- ---------- ---------- ---------- 937
       SIMLO10_LEAF  ---------- ---------- ---------- ---------- ---------- 768
      SIMLO10_ROOTS  ---------- ---------- ---------- ---------- ---------- 768
     SIMLO10_FLOWER  ---------- ---------- ---------- ---------- ---------- 768
      SIMLO10_FRUIT  ---------- ---------- ---------- ---------- ---------- 768

                              2,960                          2,980                       3,000
                                |                              |                           |
gDNA_Solyc02g083720  TCTATGTTGT TCCACTGATT AAGTTCTCTT TATTAATAGA AGCATTTTTA 3000
cDNA_Solyc02g083720  ---------- ---------- ---------- ---------- ---------- 937
       SIMLO10_LEAF  ---------- ---------- ---------- ---------- ---------- 768
      SIMLO10_ROOTS  ---------- ---------- ---------- ---------- ---------- 768
     SIMLO10_FLOWER  ---------- ---------- ---------- ---------- ---------- 768
      SIMLO10_FRUIT  ---------- ---------- ---------- ---------- ---------- 768
```

```
                                         3,020                3,040
gDNA_Solyc02g083720  CTAATCCATG AAACAAAGGG CTCGCTATCT AAAATTACAG TGACTTCCTA 3050
cDNA_Solyc02g083720  ---------- ---------- ---------- ---------- ---------- 937
       SlMLO10_LEAF  ---------- ---------- ---------- ---------- ---------- 768
      SlMLO10_ROOTS  ---------- ---------- ---------- ---------- ---------- 768
     SlMLO10_FLOWER  ---------- ---------- ---------- ---------- ---------- 768
      SlMLO10_FRUIT  ---------- ---------- ---------- ---------- ---------- 768
                              3,060                3,080                3,100
gDNA_Solyc02g083720  CTGCCAAATG TATTTGTTGT TGTTGCGATA TCAGGATTTA CACCTCTATA 3100
cDNA_Solyc02g083720  ---------- ---------- ---------- ---------- ---------- 937
       SlMLO10_LEAF  ---------- ---------- ---------- ---------- ---------- 768
      SlMLO10_ROOTS  ---------- ---------- ---------- ---------- ---------- 768
     SlMLO10_FLOWER  ---------- ---------- ---------- ---------- ---------- 768
      SlMLO10_FRUIT  ---------- ---------- ---------- ---------- ---------- 768
                                         3,120                3,140
gDNA_Solyc02g083720  TATCCCTTTG ACAGAAAAAC AAGTTCGTGT CTTTATATTG TTATTGTGGT 3150
cDNA_Solyc02g083720  ---------- ---------- ---------- ---------- ---------- 937
       SlMLO10_LEAF  ---------- ---------- ---------- ---------- ---------- 768
      SlMLO10_ROOTS  ---------- ---------- ---------- ---------- ---------- 768
     SlMLO10_FLOWER  ---------- ---------- ---------- ---------- ---------- 768
      SlMLO10_FRUIT  ---------- ---------- ---------- ---------- ---------- 768
                              3,160                3,180                3,200
gDNA_Solyc02g083720  CTTGCAGACT CATCAGCTGC CATTAACTTA TGATTTCCAT AAGTATATGC 3200
cDNA_Solyc02g083720  -------ACT CATCAGCTGC CATTAACTTA TGATTTCCAT AAGTATATGC 980
       SlMLO10_LEAF  -------ACT CATCAGCTGC CATTAACTTA TGATTTCCAT AAGTATATGC 811
      SlMLO10_ROOTS  -------ACT CATCAGCTGC CATTAACTTA TGATTTCCAT AAGTATATGC 811
     SlMLO10_FLOWER  -------ACT CATCAGCTGC CATTAACTTA TGATTTCCAT AAGTATATGC 811
      SlMLO10_FRUIT  -------ACT CATCAGCTGC CATTAACTTA TGATTTCCAT AAGTATATGC 811
                                         3,220                3,240
gDNA_Solyc02g083720  TTCGAAGCAT GGAGGAAGAA TTTCGTGATA TTGTTGGCAT AAGGTACAAT 3250
cDNA_Solyc02g083720  TTCGAAGCAT GGAGGAAGAA TTTCGTGATA TTGTTGGCAT AAG------- 1023
       SlMLO10_LEAF  TTCGAAGCAT GGAGGAAGAA TTTCGTGATA TTGTTGGCAT AAG------- 854
      SlMLO10_ROOTS  TTCGAAGCAT GGAGGAAGAA TTTCGTGATA TTGTTGGCAT AAG------- 854
     SlMLO10_FLOWER  TTCGAAGCAT GGAGGAAGAA TTTCGTGATA TTGTTGGCAT AAG------- 854
      SlMLO10_FRUIT  TTCGAAGCAT GGAGGAAGAA TTTCGTGATA TTGTTGGCAT AAG------- 854
                              3,260                3,280                3,300
gDNA_Solyc02g083720  GACATGTCTA TTTGAAGTTT AAGTTTATGT TTTGAACATA ACTTTTGTGT 3300
cDNA_Solyc02g083720  ---------- ---------- ---------- ---------- ---------- 1023
       SlMLO10_LEAF  ---------- ---------- ---------- ---------- ---------- 854
      SlMLO10_ROOTS  ---------- ---------- ---------- ---------- ---------- 854
     SlMLO10_FLOWER  ---------- ---------- ---------- ---------- ---------- 854
      SlMLO10_FRUIT  ---------- ---------- ---------- ---------- ---------- 854
                                         3,320                3,340
gDNA_Solyc02g083720  CACAATGTAA CATTCAGTAC TAGGTCTTCC AACATATGAA TCATCCTACA 3350
cDNA_Solyc02g083720  ---------- ---------- ---------- ---------- ---------- 1023
       SlMLO10_LEAF  ---------- ---------- ---------- ---------- ---------- 854
      SlMLO10_ROOTS  ---------- ---------- ---------- ---------- ---------- 854
     SlMLO10_FLOWER  ---------- ---------- ---------- ---------- ---------- 854
      SlMLO10_FRUIT  ---------- ---------- ---------- ---------- ---------- 854
                              3,360                3,380                3,400
gDNA_Solyc02g083720  TGATGCCAGA GCAATAATAA CTCCATATAA AATATCTGTA GTCATACAGA 3400
cDNA_Solyc02g083720  ---------- ---------- ---------- ---------- ---------- 1023
       SlMLO10_LEAF  ---------- ---------- ---------- ---------- ---------- 854
      SlMLO10_ROOTS  ---------- ---------- ---------- ---------- ---------- 854
     SlMLO10_FLOWER  ---------- ---------- ---------- ---------- ---------- 854
      SlMLO10_FRUIT  ---------- ---------- ---------- ---------- ---------- 854
                                         3,420                3,440
gDNA_Solyc02g083720  TATATGTAAT TGCTAGTTCT GAGGTTACAC AATTTATGTT TTCTTTTTTA 3450
cDNA_Solyc02g083720  ---------- ---------- ---------- ---------- ---------- 1023
       SlMLO10_LEAF  ---------- ---------- ---------- ---------- ---------- 854
      SlMLO10_ROOTS  ---------- ---------- ---------- ---------- ---------- 854
     SlMLO10_FLOWER  ---------- ---------- ---------- ---------- ---------- 854
      SlMLO10_FRUIT  ---------- ---------- ---------- ---------- ---------- 854
                              3,460                3,480                3,500
gDNA_Solyc02g083720  TTACAGTTCC GTGTTTCCAC TTAATAGCTA CAGCTATCAT GTTTGGTTAA 3500
cDNA_Solyc02g083720  ---------- ---------- ---------- ---------- ---------- 1023
       SlMLO10_LEAF  ---------- ---------- ---------- ---------- ---------- 854
      SlMLO10_ROOTS  ---------- ---------- ---------- ---------- ---------- 854
     SlMLO10_FLOWER  ---------- ---------- ---------- ---------- ---------- 854
      SlMLO10_FRUIT  ---------- ---------- ---------- ---------- ---------- 854
                                         3,520                3,540
gDNA_Solyc02g083720  ACCAGTAAAG ATGTTAAGAT TGAATTCTTG ACCCTCCTTG GAGGGAGTAT 3550
cDNA_Solyc02g083720  ---------- ---------- ---------- ---------- ---------- 1023
       SlMLO10_LEAF  ---------- ---------- ---------- ---------- ---------- 854
      SlMLO10_ROOTS  ---------- ---------- ---------- ---------- ---------- 854
     SlMLO10_FLOWER  ---------- ---------- ---------- ---------- ---------- 854
      SlMLO10_FRUIT  ---------- ---------- ---------- ---------- ---------- 854
                              3,560                3,580                3,600
gDNA_Solyc02g083720  AAATATTGTC TTTCTATTTC TCTATTTTAT CTTCACCAAC AAGTTCTACT 3600
cDNA_Solyc02g083720  ---------- ---------- ---------- ---------- ---------- 1023
       SlMLO10_LEAF  ---------- ---------- ---------- ---------- ---------- 854
      SlMLO10_ROOTS  ---------- ---------- ---------- ---------- ---------- 854
     SlMLO10_FLOWER  ---------- ---------- ---------- ---------- ---------- 854
      SlMLO10_FRUIT  ---------- ---------- ---------- ---------- ---------- 854
```

```
                                              3,620                         3,640
                                                |                             |
gDNA_Solyc02g083720  TCTATGTTTT  ATGTTCGCCA  ACAAGAACCT  TTCGTTTTAA  ATCTATAACT  3650
cDNA_Solyc02g083720  ----------  ----------  ----------  ----------  ----------  1023
       SIMLO10_LEAF  ----------  ----------  ----------  ----------  ----------  854
      SIMLO10_ROOTS  ----------  ----------  ----------  ----------  ----------  854
     SIMLO10_FLOWER  ----------  ----------  ----------  ----------  ----------  854
      SIMLO10_FRUIT  ----------  ----------  ----------  ----------  ----------  854

                                3,660                        3,680                        3,700
                                  |                            |                            |
gDNA_Solyc02g083720  TGGCCTTCCT  ATCTTTCCCT  TCAACTAGAA  TTAGTTATTT  TCTATCTCAT  3700
cDNA_Solyc02g083720  ----------  ----------  ----------  ----------  ----------  1023
       SIMLO10_LEAF  ----------  ----------  ----------  ----------  ----------  854
      SIMLO10_ROOTS  ----------  ----------  ----------  ----------  ----------  854
     SIMLO10_FLOWER  ----------  ----------  ----------  ----------  ----------  854
      SIMLO10_FRUIT  ----------  ----------  ----------  ----------  ----------  854

                                              3,720                         3,740
                                                |                             |
gDNA_Solyc02g083720  GGTAAAAAAA  TAGCTGCTCC  ACGTGAACTA  TAGTAAGTAC  TTATAACAGA  3750
cDNA_Solyc02g083720  ----------  ----------  ----------  ----------  ----------  1023
       SIMLO10_LEAF  ----------  ----------  ----------  ----------  ----------  854
      SIMLO10_ROOTS  ----------  ----------  ----------  ----------  ----------  854
     SIMLO10_FLOWER  ----------  ----------  ----------  ----------  ----------  854
      SIMLO10_FRUIT  ----------  ----------  ----------  ----------  ----------  854

                                3,760                        3,780                        3,800
                                  |                            |                            |
gDNA_Solyc02g083720  TTCTATGAGA  ATTCATATAA  CTCATTCCAA  TTTTCTGTTC  CTGACTGTCA  3800
cDNA_Solyc02g083720  ----------  ----------  ----------  ----------  ----------  1023
       SIMLO10_LEAF  ----------  ----------  ----------  ----------  ----------  854
      SIMLO10_ROOTS  ----------  ----------  ----------  ----------  ----------  854
     SIMLO10_FLOWER  ----------  ----------  ----------  ----------  ----------  854
      SIMLO10_FRUIT  ----------  ----------  ----------  ----------  ----------  854

                                              3,820                         3,840
                                                |                             |
gDNA_Solyc02g083720  TGATCAACAG  TTAAAGCTTT  CCGTATCTCT  AGGCATATAC  GATGTGTTGT  3850
cDNA_Solyc02g083720  ----------  ----------  ----------  ----------  ----------  1023
       SIMLO10_LEAF  ----------  ----------  ----------  ----------  ----------  854
      SIMLO10_ROOTS  ----------  ----------  ----------  ----------  ----------  854
     SIMLO10_FLOWER  ----------  ----------  ----------  ----------  ----------  854
      SIMLO10_FRUIT  ----------  ----------  ----------  ----------  ----------  854

                                3,860                        3,880                        3,900
                                  |                            |                            |
gDNA_Solyc02g083720  TTATTTCCAT  TTATATGGTA  CTGTAAGACA  AAAGCTGACA  TATATGTCCC  3900
cDNA_Solyc02g083720  ----------  ----------  ----------  ----------  ----------  1023
       SIMLO10_LEAF  ----------  ----------  ----------  ----------  ----------  854
      SIMLO10_ROOTS  ----------  ----------  ----------  ----------  ----------  854
     SIMLO10_FLOWER  ----------  ----------  ----------  ----------  ----------  854
      SIMLO10_FRUIT  ----------  ----------  ----------  ----------  ----------  854

                                              3,920                         3,940
                                                |                             |
gDNA_Solyc02g083720  TGTTGGAGGA  CTAAATGCAA  TATTTCACAA  ACTGTAAGAT  ACTATGGTTA  3950
cDNA_Solyc02g083720  ----------  ----------  ----------  ----------  ----------  1023
       SIMLO10_LEAF  ----------  ----------  ----------  ----------  ----------  854
      SIMLO10_ROOTS  ----------  ----------  ----------  ----------  ----------  854
     SIMLO10_FLOWER  ----------  ----------  ----------  ----------  ----------  854
      SIMLO10_FRUIT  ----------  ----------  ----------  ----------  ----------  854

                                3,960                        3,980                        4,000
                                  |                            |                            |
gDNA_Solyc02g083720  TGGAAAGACA  TCTAAGATAT  TCTATTTTTT  TGGCTTTACT  GCACAATTTT  4000
cDNA_Solyc02g083720  ----------  ----------  ----------  ----------  ----------  1023
       SIMLO10_LEAF  ----------  ----------  ----------  ----------  ----------  854
      SIMLO10_ROOTS  ----------  ----------  ----------  ----------  ----------  854
     SIMLO10_FLOWER  ----------  ----------  ----------  ----------  ----------  854
      SIMLO10_FRUIT  ----------  ----------  ----------  ----------  ----------  854

                                              4,020                         4,040
                                                |                             |
gDNA_Solyc02g083720  ACTATGGATC  CAGTCATCTA  AGACACTCCT  TTTCTTTTTC  TTGTCTGCAG  4050
cDNA_Solyc02g083720  ----------  ----------  ----------  ----------  ----------  1023
       SIMLO10_LEAF  ----------  ----------  ----------  ----------  ----------  854
      SIMLO10_ROOTS  ----------  ----------  ----------  ----------  ----------  854
     SIMLO10_FLOWER  ----------  ----------  ----------  ----------  ----------  854
      SIMLO10_FRUIT  ----------  ----------  ----------  ----------  ----------  854

                                4,060                        4,080                        4,100
                                  |                            |                            |
gDNA_Solyc02g083720  TGTTCCACTA  TGGATTTTCG  TCATAGTTTG  TGTATTTTTA  AGCTTTCATG  4100
cDNA_Solyc02g083720  TGTTCCACTA  TGGATTTTCG  TCATAGTTTG  TGTATTTTTA  AGCTTTCATG  1073
       SIMLO10_LEAF  TGTTCCACTA  TGGATTTTCG  TCATAGTTTG  TGTATTTTTA  AGCTTTCATG  904
      SIMLO10_ROOTS  TGTTCCACTA  TGGATTTTCG  TCATAGTTTG  TGTATTTTTA  AGCTTTCATG  904
     SIMLO10_FLOWER  TGTTCCACTA  TGGATTTTCG  TCATAGTTTG  TGTATTTTTA  AGCTTTCATG  904
      SIMLO10_FRUIT  TGTTCCACTA  TGGATTTTCG  TCATAGTTTG  TGTATTTTTA  AGCTTTCATG  904

                                              4,120                         4,140
                                                |                             |
gDNA_Solyc02g083720  GTAACTTTTA  TCTACTAGAT  ATTATCAGGA  ATTTCTGCCT  CGGAATGCTT  4150
cDNA_Solyc02g083720  G---------  ----------  ----------  ----------  ----------  1074
       SIMLO10_LEAF  G---------  ----------  ----------  ----------  ----------  905
      SIMLO10_ROOTS  G---------  ----------  ----------  ----------  ----------  905
     SIMLO10_FLOWER  G---------  ----------  ----------  ----------  ----------  905
      SIMLO10_FRUIT  G---------  ----------  ----------  ----------  ----------  905

                                4,160                        4,180                        4,200
                                  |                            |                            |
gDNA_Solyc02g083720  TTTTCAGTAC  AATTTTAATG  ATCATTGTCC  AGGCACAAAT  ATCTACTTTT  4200
cDNA_Solyc02g083720  ----------  ----------  ----------  ---CACAAAT  ATCTACTTTT  1091
       SIMLO10_LEAF  ----------  ----------  ----------  ---CACAAAT  ATCTACTTTT  922
      SIMLO10_ROOTS  ----------  ----------  ----------  ---CACAAAT  ATCTACTTTT  922
     SIMLO10_FLOWER  ----------  ----------  ----------  ---CACAAAT  ATCTACTTTT  922
      SIMLO10_FRUIT  ----------  ----------  ----------  ---CACAAAT  ATCTACTTTT  922
```

```
                       4,220                 4,240
gDNA_Solyc02g083720  GGATCTCCTT CTTTCCAGCT ATCGTAAGTC CTTGTTATTT GCTGTTCCAT 4250
cDNA_Solyc02g083720  GGATCTCCTT CTTTCCAGCT ATC------- ---------- ---------- 1114
SlMLO10_LEAF         GGATCTCCTT CTTTCCAGCT ATC------- ---------- ---------- 945
SlMLO10_ROOTS        GGATCTCCTT CTTTCCAGCT ATC------- ---------- ---------- 945
SlMLO10_FLOWER       GGATCTCCTT CTTTCCAGCT ATC------- ---------- ---------- 945
SlMLO10_FRUIT        GGATCTCCTT CTTTCCAGCT ATC------- ---------- ---------- 945

             4,260                 4,280                 4,300
gDNA_Solyc02g083720  TTTTCATTTT GCTTCTGAAG AATAATTTGT TCTCATATTT CTGACTTTGT 4300
cDNA_Solyc02g083720  ---------- ---------- ---------- ---------- ---------- 1114
SlMLO10_LEAF         ---------- ---------- ---------- ---------- ---------- 945
SlMLO10_ROOTS        ---------- ---------- ---------- ---------- ---------- 945
SlMLO10_FLOWER       ---------- ---------- ---------- ---------- ---------- 945
SlMLO10_FRUIT        ---------- ---------- ---------- ---------- ---------- 945

                       4,320                 4,340
gDNA_Solyc02g083720  CAAAAATTGT TATTGGAAGT TAATCCTTTT GGTTGGTACC AAGCTTCATC 4350
cDNA_Solyc02g083720  ---------- ---------T TAATCCTTTT GGTTGGTACC AAGCTTCATC 1145
SlMLO10_LEAF         ---------- ---------T TAATCCTTTT GGTTGGTACC AAGCTTCATC 976
SlMLO10_ROOTS        ---------- ---------T TAATCCTTTT GGTTGGTACC AAGCTTCATC 976
SlMLO10_FLOWER       ---------- ---------T TAATCCTTTT GGTTGGTACC AAGCTTCATC 976
SlMLO10_FRUIT        ---------- ---------T TAATCCTTTT GGTTGGTACC AAGCTTCATC 976

             4,360                 4,380                 4,400
gDNA_Solyc02g083720  GCGTGGTGGT CAAGCTGGCA GTTGAAATCA TTGACAGTAG TCCATTGGAA 4400
cDNA_Solyc02g083720  GCGTGGTGGT CAAGCTGGCA GTTGAAATCA TTGACAGTAG TCCATTGGAA 1195
SlMLO10_LEAF         GCGTGGTGGT CAAGCTGGCA GTTGAAATCA TTGACAGTAG TCCATTGGAA 1026
SlMLO10_ROOTS        GCGTGGTGGT CAAGCTGGCA GTTGAAATCA TTGACAGTAG TCCACTGGAA 1026
SlMLO10_FLOWER       GCGTGGTGGT CAAGCTGGCA GTTGAAATCA TTGACAGTAG TCCATTGGAA 1026
SlMLO10_FRUIT        GCGTGGTGGT CAAGCTGGCA GTTGAAATCA TTGACAGTAG TCCATTGGAA 1026

                       4,420                 4,440
gDNA_Solyc02g083720  GGATTTCATC AGTTCAACCT TAGAGATGAG CTCTTCTGGT TTGGGAAGCC 4450
cDNA_Solyc02g083720  GGATTTCATC AGTTCAACCT TAGAGATGAG CTCTTCTGGT TTGGGAAGCC 1245
SlMLO10_LEAF         GGATTTCATC AGTTCAACCT TAGAGATGAG CTCTTCTGGT TTGGGAAGCC 1076
SlMLO10_ROOTS        GGATTTCATC AGTTCAACCT TAGAGATGAG CTCTTCTGGT TTGGGAAGCC 1076
SlMLO10_FLOWER       GGATTTCATC AGTTCAACCT TAGAGATGAG CTCTTCTGGT TTGGGAAGCC 1076
SlMLO10_FRUIT        GGATTTCATC AGTTCAACCT TAGAGATGAG CTCTTCTGGT TTGGGAAGCC 1076

             4,460                 4,480                 4,500
gDNA_Solyc02g083720  TAGATTTCTG CTGCGGATAA TACAATTTGT ATCATTCCAG GTATGACCAA 4500
cDNA_Solyc02g083720  TAGATTTCTG CTGCGGATAA TACAATTTGT ATCATTCCAG ---------- 1285
SlMLO10_LEAF         TAGATTTCTG CTGCGGATAA TACAATTTGT ATCATTCCAG ---------- 1116
SlMLO10_ROOTS        TAGATTTCTG CTGCGGATAA TACAATTTGT ATCACTCCAG ---------- 1116
SlMLO10_FLOWER       TAGATTTCTG CTGCGGATAA TACAATTTGT ATCATTCCAG ---------- 1116
SlMLO10_FRUIT        TAGATTTCTG CTGCGGATAA TACAATTTGT ATCATTCCAG ---------- 1116

                       4,520                 4,540
gDNA_Solyc02g083720  TAACATCTTT TTATCCTCGA ATCCAAATGG TAACAAGAAG AATATTACAA 4550
cDNA_Solyc02g083720  ---------- ---------- ---------- ---------- ---------- 1285
SlMLO10_LEAF         ---------- ---------- ---------- ---------- ---------- 1116
SlMLO10_ROOTS        ---------- ---------- ---------- ---------- ---------- 1116
SlMLO10_FLOWER       ---------- ---------- ---------- ---------- ---------- 1116
SlMLO10_FRUIT        ---------- ---------- ---------- ---------- ---------- 1116

             4,560                 4,580                 4,600
gDNA_Solyc02g083720  CTAATTTTTA GCTCAATGAT ATAGCGTCCT TGGATATGCT TTACTGATTG 4600
cDNA_Solyc02g083720  ---------- ---------- ---------- ---------- ---------- 1285
SlMLO10_LEAF         ---------- ---------- ---------- ---------- ---------- 1116
SlMLO10_ROOTS        ---------- ---------- ---------- ---------- ---------- 1116
SlMLO10_FLOWER       ---------- ---------- ---------- ---------- ---------- 1116
SlMLO10_FRUIT        ---------- ---------- ---------- ---------- ---------- 1116

                       4,620                 4,640
gDNA_Solyc02g083720  CTCTTCTTTC AATCATATGC AGAATGCATT TGAGATGGCA ACATACATAT 4650
cDNA_Solyc02g083720  ---------- ---------- --AATGCATT TGAGATGGCA ACATACATAT 1313
SlMLO10_LEAF         ---------- ---------- --AATGCATT TGAGATGGCA ACATACATAT 1144
SlMLO10_ROOTS        ---------- ---------- --AATGCATT TGAGATGGCA ACATACATAT 1144
SlMLO10_FLOWER       ---------- ---------- --AATGCATT TGAGATGGCA ACATACATAT 1144
SlMLO10_FRUIT        ---------- ---------- --AATGCATT TGAGATGGCA ACATACATAT 1144

             4,660                 4,680                 4,700
gDNA_Solyc02g083720  GGTCACTGGT AAGAACTTGA GAAACTAGTG TTCTGATAGA AGTTTTGCTT 4700
cDNA_Solyc02g083720  GGTCACTG-- ---------- ---------- ---------- ---------- 1321
SlMLO10_LEAF         GGTCACTG-- ---------- ---------- ---------- ---------- 1152
SlMLO10_ROOTS        GGTCACTG-- ---------- ---------- ---------- ---------- 1152
SlMLO10_FLOWER       GGTCACTG-- ---------- ---------- ---------- ---------- 1152
SlMLO10_FRUIT        GGTCACTG-- ---------- ---------- ---------- ---------- 1152

                       4,720                 4,740
gDNA_Solyc02g083720  TACTGTAAAT TAATTCTTCA CCCAATACAT CCCTTCTGTT AGTCGGTAGA 4750
cDNA_Solyc02g083720  ---------- ---------- ---------- ---------- ---------- 1321
SlMLO10_LEAF         ---------- ---------- ---------- ---------- ---------- 1152
SlMLO10_ROOTS        ---------- ---------- ---------- ---------- ---------- 1152
SlMLO10_FLOWER       ---------- ---------- ---------- ---------- ---------- 1152
SlMLO10_FRUIT        ---------- ---------- ---------- ---------- ---------- 1152

             4,760                 4,780                 4,800
gDNA_Solyc02g083720  TGCAAGTTTC AAACATTGAT TATTCACATA CTCTCCATTT TCTCTGCACC 4800
cDNA_Solyc02g083720  ---------- ---------- ---------- ---------- ---------- 1321
SlMLO10_LEAF         ---------- ---------- ---------- ---------- ---------- 1152
SlMLO10_ROOTS        ---------- ---------- ---------- ---------- ---------- 1152
SlMLO10_FLOWER       ---------- ---------- ---------- ---------- ---------- 1152
SlMLO10_FRUIT        ---------- ---------- ---------- ---------- ---------- 1152
```

```
                                              4,820                         4,840
gDNA_Solyc02g083720  CTTTTGTGAT CAAAGCAAGG GACATAGATT CTTCTGCCTA GTGTTTTTCT 4850
cDNA_Solyc02g083720  ---------- ---------- ---------- ---------- ---------- 1321
        SIMLO10_LEAF ---------- ---------- ---------- ---------- ---------- 1152
       SIMLO10_ROOTS ---------- ---------- ---------- ---------- ---------- 1152
      SIMLO10_FLOWER ---------- ---------- ---------- ---------- ---------- 1152
       SIMLO10_FRUIT ---------- ---------- ---------- ---------- ---------- 1152
                              4,860                         4,880                  4,900
gDNA_Solyc02g083720  CGTTTGACGC TCTTGATTAT TTTGCCTTTT TGGGAGCCTC TACAATATAA 4900
cDNA_Solyc02g083720  ---------- ---------- ---------- ---------- ---------- 1321
        SIMLO10_LEAF ---------- ---------- ---------- ---------- ---------- 1152
       SIMLO10_ROOTS ---------- ---------- ---------- ---------- ---------- 1152
      SIMLO10_FLOWER ---------- ---------- ---------- ---------- ---------- 1152
       SIMLO10_FRUIT ---------- ---------- ---------- ---------- ---------- 1152
                                              4,920                         4,940
gDNA_Solyc02g083720  CTTGACCAAA ATTTTCAACT GGAGAAGCAA TTAGTTTTGG TTGGAAACAG 4950
cDNA_Solyc02g083720  ---------- ---------- ---------- ---------- ---------- 1321
        SIMLO10_LEAF ---------- ---------- ---------- ---------- ---------- 1152
       SIMLO10_ROOTS ---------- ---------- ---------- ---------- ---------- 1152
      SIMLO10_FLOWER ---------- ---------- ---------- ---------- ---------- 1152
       SIMLO10_FRUIT ---------- ---------- ---------- ---------- ---------- 1152
                              4,960                         4,980                  5,000
gDNA_Solyc02g083720  AAATTGTACA ACTTTAAAGA ACATATATAT AACTTTTCAA CTCTTATAAC 5000
cDNA_Solyc02g083720  ---------- ---------- ---------- ---------- ---------- 1321
        SIMLO10_LEAF ---------- ---------- ---------- ---------- ---------- 1152
       SIMLO10_ROOTS ---------- ---------- ---------- ---------- ---------- 1152
      SIMLO10_FLOWER ---------- ---------- ---------- ---------- ---------- 1152
       SIMLO10_FRUIT ---------- ---------- ---------- ---------- ---------- 1152
                                              5,020                         5,040
gDNA_Solyc02g083720  AAACTGCAAA GTACTATTGA ACTATGGGCT AAACAACACA GAGAGTTAAA 5050
cDNA_Solyc02g083720  ---------- ---------- ---------- ---------- ---------- 1321
        SIMLO10_LEAF ---------- ---------- ---------- ---------- ---------- 1152
       SIMLO10_ROOTS ---------- ---------- ---------- ---------- ---------- 1152
      SIMLO10_FLOWER ---------- ---------- ---------- ---------- ---------- 1152
       SIMLO10_FRUIT ---------- ---------- ---------- ---------- ---------- 1152
                              5,060                         5,080                  5,100
gDNA_Solyc02g083720  CAGACAATGT TTTCATATGT AGTTTGTGTC TGTTTTATGT TTATACCTAA 5100
cDNA_Solyc02g083720  ---------- ---------- ---------- ---------- ---------- 1321
        SIMLO10_LEAF ---------- ---------- ---------- ---------- ---------- 1152
       SIMLO10_ROOTS ---------- ---------- ---------- ---------- ---------- 1152
      SIMLO10_FLOWER ---------- ---------- ---------- ---------- ---------- 1152
       SIMLO10_FRUIT ---------- ---------- ---------- ---------- ---------- 1152
                                              5,120                         5,140
gDNA_Solyc02g083720  TCCTTAGGCG AGGCAACACA GACATTCGTG TCACTGCATG AATACAAATT 5150
cDNA_Solyc02g083720  ---------- ---------- ---------- ---------- ---------- 1321
        SIMLO10_LEAF ---------- ---------- ---------- ---------- ---------- 1152
       SIMLO10_ROOTS ---------- ---------- ---------- ---------- ---------- 1152
      SIMLO10_FLOWER ---------- ---------- ---------- ---------- ---------- 1152
       SIMLO10_FRUIT ---------- ---------- ---------- ---------- ---------- 1152
                              5,160                         5,180                  5,200
gDNA_Solyc02g083720  TTTTCCATAT GTACATGGGG ATGACAACTA ATTTCCTTTT CTTGGTGCAG 5200
cDNA_Solyc02g083720  ---------- ---------- ---------- ---------- ---------- 1321
        SIMLO10_LEAF ---------- ---------- ---------- ---------- ---------- 1152
       SIMLO10_ROOTS ---------- ---------- ---------- ---------- ---------- 1152
      SIMLO10_FLOWER ---------- ---------- ---------- ---------- ---------- 1152
       SIMLO10_FRUIT ---------- ---------- ---------- ---------- ---------- 1152
                                              5,220                         5,240
gDNA_Solyc02g083720  TGGGAAATTA AGGGATCTTC ATGTTTCACA GACAATCACA CATTTCTTGT 5250
cDNA_Solyc02g083720  TGGGAAATTA AGGGATCTTC ATGTTTCACA GACAATCACA CATTTCTTGT 1371
        SIMLO10_LEAF TGGGAAATTA AGGGATCTTC ATGTTTCACA GACAATCACA CATTTCTTGT 1202
       SIMLO10_ROOTS TGGGAAATTA AGGGGTCTTC ATGTTTCACA GACAATCACA CATTTCTTGT 1202
      SIMLO10_FLOWER TGGGAAATTA AGGGATCTTC ATGTTTCACA GACAATCACA CATTTCTTGT 1202
       SIMLO10_FRUIT TGGGAAATTA AGGGATCTTC ATGTTTCACA GACAATCACA CATTTCTTGT 1202
                              5,260                         5,280                  5,300
gDNA_Solyc02g083720  GATTCGCTTG TCATTTGGGT AACTATCTTG TGTCTCACTT CCTTGTTAAC 5300
cDNA_Solyc02g083720  GATTCGCTTG TCATTTGGG- ---------- ---------- ---------- 1390
        SIMLO10_LEAF GATTCGCTTG TCATTTGGG- ---------- ---------- ---------- 1221
       SIMLO10_ROOTS GATTCGCTTG TCATTTGGG- ---------- ---------- ---------- 1221
      SIMLO10_FLOWER GATTCGCTTG TCATTTGGG- ---------- ---------- ---------- 1221
       SIMLO10_FRUIT GATTCGCTTG TCATTTGGG- ---------- ---------- ---------- 1221
                                              5,320                         5,340
gDNA_Solyc02g083720  TTGTTTCTGA ATACACTGAT TTTCGCATGA TCCTTGTGAC TTGTTGCTGC 5350
cDNA_Solyc02g083720  ---------- ---------- ---------- ---------- ---------- 1390
        SIMLO10_LEAF ---------- ---------- ---------- ---------- ---------- 1221
       SIMLO10_ROOTS ---------- ---------- ---------- ---------- ---------- 1221
      SIMLO10_FLOWER ---------- ---------- ---------- ---------- ---------- 1221
       SIMLO10_FRUIT ---------- ---------- ---------- ---------- ---------- 1221
                              5,360                         5,380                  5,400
gDNA_Solyc02g083720  AGGGTTGTTT CTCAATTCTG GTGTAGCTTT GTCACATTTC CGCTCTATGT 5400
cDNA_Solyc02g083720  ---GTTGTTT CTCAATTCTG GTGTAGCTTT GTCACATTTC CGCTCTATGT 1437
        SIMLO10_LEAF ---GTTGTTT CTCAATTCTG GTGTAGCTTT GTCACATTTC CGCTCTATGT 1268
       SIMLO10_ROOTS ---GTTGTTT CTCAATTCTG GTGTAGCTTT GTCACATTTC CGCTCTATGT 1268
      SIMLO10_FLOWER ---GTTGTTT CTCAATTCTG GTGTAGCTTT GTCACATTTC CGCTCTATGT 1268
       SIMLO10_FRUIT ---GTTGTTT CTCAATTCTG GTGTAGCTTT GTCACATTTC CGCTCTATGT 1268
```

```
                                                   5,420                                   5,440
gDNA_Solyc02g083720  TATTGTTGCT CAGGTAACTA ACAAACACAA TGTAATCTCT TTTTTTTGTC 5450
cDNA_Solyc02g083720  TATTGTTGCT CAG------- ---------- ---------- ---------- 1450
       SIMLO10_LEAF  TATTGTTGCT CAG------- ---------- ---------- ---------- 1281
      SIMLO10_ROOTS  TATTGTTGCT CAG------- ---------- ---------- ---------- 1281
     SIMLO10_FLOWER  TATTGTTGCT CAG------- ---------- ---------- ---------- 1281
      SIMLO10_FRUIT  TATTGTTGCT CAG------- ---------- ---------- ---------- 1281
                                 5,460                                   5,480                                   5,500
gDNA_Solyc02g083720  CTTAGTAATC ATTCTACACA TTTTATGTCA AGTCAGTGAT CCATGTTTGA 5500
cDNA_Solyc02g083720  ---------- ---------- ---------- ---------- ---------- 1450
       SIMLO10_LEAF  ---------- ---------- ---------- ---------- ---------- 1281
      SIMLO10_ROOTS  ---------- ---------- ---------- ---------- ---------- 1281
     SIMLO10_FLOWER  ---------- ---------- ---------- ---------- ---------- 1281
      SIMLO10_FRUIT  ---------- ---------- ---------- ---------- ---------- 1281
                                                   5,520                                   5,540
gDNA_Solyc02g083720  GCAGATGGGT TCGAGATACA AGAAGACCAT TGTTTCAGAG AATGTTAGGA 5550
cDNA_Solyc02g083720  ----ATGGGT TCGAGATACA AGAAGACCAT TGTTTCAGAG AATGTTAGGA 1496
       SIMLO10_LEAF  ----ATGGGT TCGAGATACA AGAAGACCAT TGTTTCAGAG AATGTTAGGA 1327
      SIMLO10_ROOTS  ----ATGGGT TCGAGATACA AGAAGACCAT TGTTTCAGAG AATGTTAGGA 1327
     SIMLO10_FLOWER  ----ATGGGT TCGAGATACA AGAAGACCAT TGTTTCAGAG AATGTTAGGA 1327
      SIMLO10_FRUIT  ----ATGGGT TCGAGATACA AGAAGACCAT TGTTTCAGAG AATGTTAGGA 1327
                                 5,560                                   5,580                                   5,600
gDNA_Solyc02g083720  CTTCGCTACA TGGATGGCGA CACAAGGTGA AAACTAGACT AGAAGGTTCT 5600
cDNA_Solyc02g083720  CTTCGCTACA TGGATGGCGA CACAAGGTGA AAACTAGACT AGAAGGTTCT 1546
       SIMLO10_LEAF  CTTCGCTACA TGGATGGCGA CACAAGGTGA AAACTAGACT AGAAGGTTCT 1377
      SIMLO10_ROOTS  CTTCGCTACA TGGATGGCGA CACAAGGTGA AAACTAGACT AGAAGGTTCT 1377
     SIMLO10_FLOWER  CTTCGCTACA TGGATGGCGA CACAAGGTGA AAACTAGACT AGAAGGTTCT 1377
      SIMLO10_FRUIT  CTTCGCTACA TGGATGGCGA CACAAGGTGA AAACTAGACT AGAAGGTTCT 1377
                                                   5,620                                   5,640
gDNA_Solyc02g083720  GTCGTTTCTC CAGAAACATT ATTAGCAACC ACATCATTAG ACTCCATGGC 5650
cDNA_Solyc02g083720  GTCGTTTCTC CAGAAACATT ATTAGCAACC ACATCATTAG ACTCCATGGC 1596
       SIMLO10_LEAF  GTCGTTTCTC CAGAAACATT ATTAGCAACC ACATCATTAG ACTCCATGGC 1427
      SIMLO10_ROOTS  GTCGTTTCTC CAGAAACATT ATTAGCAACC ACATCATTAG ACTCCATGGC 1427
     SIMLO10_FLOWER  GTCGTTTCTC CAGAAACATT ATTAGCAACC ACATCATTAG ACTCCATGGC 1427
      SIMLO10_FRUIT  GTCGTTTCTC CAGAAACATT ATTAGCAACC ACATCATTAG ACTCCATGGC 1427
                                 5,660                                   5,680                                   5,700
gDNA_Solyc02g083720  GGAGGATGAG GTAGATCAAA TTCATAGCGT TGCTACTATC AGCACAGAGG 5700
cDNA_Solyc02g083720  GGAGGATGAG GTAGATCAAA TTCATAGCGT TGCTACTATC AGCACAGAGG 1646
       SIMLO10_LEAF  GGAGGATGAG GTAGATCAAA TTCATAGCGT TGCTACTATC AGCACAGAGG 1477
      SIMLO10_ROOTS  GGAGGATGAG GTAGATCAAA TTCATAGCGT TGCTACTATC AGCACAGAGG 1477
     SIMLO10_FLOWER  GGAGGATGAG GTAGATCAAA TTCATAGCGT TGCTACTATC AGCACAGAGG 1477
      SIMLO10_FRUIT  GGAGGATGAG GTAGATCAAA TTCATAGCGT TGCTACTATC AGCACAGAGG 1477
                                                   5,720                                   5,740
gDNA_Solyc02g083720  TGTTTGATGA GAGTACAGTT GAGTCGGAAC AACGAGTCCC TATTGAGCAA 5750
cDNA_Solyc02g083720  TGTTTGATGA GAGTACAGTT GAGTCGGAAC AACGAGTCCC TATTGAGCAA 1696
       SIMLO10_LEAF  TGTTTGATGA GAGTACAGTT GAGTCGGAAC AACGAGTCCC TATTGAGCAA 1527
      SIMLO10_ROOTS  TGTTTGATGA GAGTACAGTT GAGTCGGAAC AACGAGTCCC TATTGAGCAA 1527
     SIMLO10_FLOWER  TGTTTGATGA GAGTACAGTT GAGTCGGAAC AACGAGTCCC TATTGAGCAA 1527
      SIMLO10_FRUIT  TGTTTGATGA GAGTACAGTT GAGTCGGAAC AACGAGTCCC TATTGAGCAA 1527
                                 5,760                                   5,780                                   5,800
gDNA_Solyc02g083720  AGTTGTACCA ATGAGATCTC AGAATGTGAT GAGTTACATA TTCCACTTTC 5800
cDNA_Solyc02g083720  AGTTGTACCA ATGAGATCTC AGAATGTGAT GAGTTACATA TTCCACTTTC 1746
       SIMLO10_LEAF  AGTTGTACCA ATGAGATCTC AGAATGTGAT GAGTTACATA TTCCACTTTC 1577
      SIMLO10_ROOTS  AGTTGTACCA ATGAGATCTC AGAATGTGAT GAGTTACATA TTCCACTTTC 1577
     SIMLO10_FLOWER  AGTTGTACCA ATGAGATCTC AGAATGTGAT GAGTTACATA TTCCACTTTC 1577
      SIMLO10_FRUIT  AGTTGTACCA ATGAGATCTC AGAATGTGAT GAGTTACATA TTCCACTTTC 1577
                                                   5,820                                   5,840
gDNA_Solyc02g083720  TCCTAGAAAT CATGGAGAGG TTTGATCAAA ACAATGATGA GATGATATAG 5850
cDNA_Solyc02g083720  TCCTAGAAAT CATGGAGAGG TTTGATCAAA ACAATGATGA GATGATATAG 1796
       SIMLO10_LEAF  TCCTAGAAAT CATGGAGAGG TTTGA----- ---------- ---------- 1602
      SIMLO10_ROOTS  TCCTAGAAAT CATGGAGAGG TTTGA----- ---------- ---------- 1602
     SIMLO10_FLOWER  TCCTAGAAAT CATGGAGAGG TTTGA----- ---------- ---------- 1602
      SIMLO10_FRUIT  TCCTAGAAAT CATGGAGAGG TTTGA----- ---------- ---------- 1602
                                 5,860                                   5,880                                   5,900
gDNA_Solyc02g083720  TTTCGCTCCT TATATTACAG AATCCACAAC CTTAGACATA CTAATCAACT 5900
cDNA_Solyc02g083720  TTTCGCTCCT TATATTACAG AATCCACAAC CTTAGACATA CTAATCAACT 1846
       SIMLO10_LEAF  ---------- ---------- ---------- ---------- ---------- 1602
      SIMLO10_ROOTS  ---------- ---------- ---------- ---------- ---------- 1602
     SIMLO10_FLOWER  ---------- ---------- ---------- ---------- ---------- 1602
      SIMLO10_FRUIT  ---------- ---------- ---------- ---------- ---------- 1602
                                                   5,920                                5,940
gDNA_Solyc02g083720  TGTTCATCTT GCTTTTGTCC AAATTTTCCA GGCTATATAG » 5940
cDNA_Solyc02g083720  TGTTCATCTT GCTTTTGTCC AAATTTTCCA GGCTATATAG » 1886
       SIMLO10_LEAF  ---------- ---------- ---------- ---------- 1602
      SIMLO10_ROOTS  ---------- ---------- ---------- ---------- 1602
     SIMLO10_FLOWER  ---------- ---------- ---------- ---------- 1602
      SIMLO10_FRUIT  ---------- ---------- ---------- ---------- 1602
```

```
                                        20                            40
                                         |                             |
gDNA_Solyc01g102520 ‹TCATCAAGAC ATGCATTAAG GTTAAATATG GCGGAAAAGT CTGAGTCGTC 50
cDNA_Solyc01g102520 ‹TCATCAAGAC ATGCATTAAG GTTAAATATG GCGGAAAAGT CTGAGTCGTC 50
       SIMLO11_LEAF  ---------- ---------- -------ATG GCGGAAAAGT CTGAGTCGTC 23
       SIMLO11_ROOT  ---------- ---------- -------ATG GCGGAAAAGT CTGAGTCGTC 23
    SIMILO11_FLOWER  ---------- ---------- -------ATG GCGGAAAAGT CTGAGTCGTC 23
      SIMLO11_ FRUIT ---------- ---------- -------ATG GCGGAAAAGT CTGAGTCGTC 23
                              60                            80                 100
                               |                             |                   |
gDNA_Solyc01g102520  TCTGGAGTAT ACACCAACAT GGGTCGTTGC CGTTGTCTGT TTCATCATTG 100
cDNA_Solyc01g102520  TCTGGAGTAT ACACCAACAT GGGTCGTTGC CGTTGTCTGT TTCATCATTG 100
       SIMLO11_LEAF  TCTGGAGTAT ACACCAACAT GGGTCGTTGC CGTTGTCTGT TTCATCATTG 73
       SIMLO11_ROOT  TCTGGAGTAT ACACCAACAT GGGTCGTTGC CGTTGTCTGT TTCATCATTG 73
    SIMILO11_FLOWER  TCTGGAGTAT ACACCAACAT GGGTCGTTGC CGTTGTCTGT TTCATCATTG 73
      SIMLO11_ FRUIT TCTGGAGTAT ACACCAACAT GGGTCGTTGC CGTTGTCTGT TTCATCATTG 73
                                        120                           140
                                         |                             |
gDNA_Solyc01g102520  TTCTCATTTC ACTTGCTGCT GAACGTGGCC TTCATCGCCT TGGAAAGGTT 150
cDNA_Solyc01g102520  TTCTCATTTC ACTTGCTGCT GAACGTGGCC TTCATCGCCT TGGAAAG--- 147
       SIMLO11_LEAF  TTCTCATTTC ACTTGCTGCT GAACGTGGCC TTCATCGCCT TGGAAAG--- 120
       SIMLO11_ROOT  TTCTCATTTC ACTTGCTGCT GAACGTGGCC TTCATCGCCT TGGAAAG--- 120
    SIMILO11_FLOWER  TTCTCATTTC ACTTGCTGCT GAACGTGGCC TTCATCGCCT TGGAAAG--- 120
      SIMLO11_ FRUIT TTCTCATTTC ACTTGCTGCT GAACGTGGCC TTCATCGCCT TGGAAAG--- 120
                              160                           180                 200
                               |                             |                   |
gDNA_Solyc01g102520  GGTCTCTACT AGTAAAGTGA TGGTACGTTA CTCTATATAC GTTCAATAGT 200
cDNA_Solyc01g102520  ---------- ---------- ---------- ---------- ---------- 147
       SIMLO11_LEAF  ---------- ---------- ---------- ---------- ---------- 120
       SIMLO11_ROOT  ---------- ---------- ---------- ---------- ---------- 120
    SIMILO11_FLOWER  ---------- ---------- ---------- ---------- ---------- 120
      SIMLO11_ FRUIT ---------- ---------- ---------- ---------- ---------- 120
                                        220                           240
                                         |                             |
gDNA_Solyc01g102520  TGATCGATGA TGTTGTTGTG TTACAGTTCT TCTTGCAAAA GAATCAAGAT 250
cDNA_Solyc01g102520  ---------- ---------- ------TTCT TCTTGCAAAA GAATCAAGAT 171
       SIMLO11_LEAF  ---------- ---------- ------TTCT TCTTGCAAAA GAATCAAGAT 144
       SIMLO11_ROOT  ---------- ---------- ------TTCT TCTTGCAAAA GAATCAAGAT 144
    SIMILO11_FLOWER  ---------- ---------- ------TTCT TCTTGCAAAA GAATCAAGAT 144
      SIMLO11_ FRUIT ---------- ---------- ------TTCT TCTTGCAAAA GAATCAAGAT 144
                              260                           280                 300
                               |                             |                   |
gDNA_Solyc01g102520  GCCCTATTCA AGGCACTCCA GAAATTAAAA GAAGGTTTGA CGACACATGG 300
cDNA_Solyc01g102520  GCCCTATTCA AGGCACTCCA GAAATTAAAA GAAG------ ---------- 205
       SIMLO11_LEAF  GCCCTATTCA AGGCACTCCA GAAATTAAAA GAAG------ ---------- 178
       SIMLO11_ROOT  GCCCTATTCA AGGCACTCCA GAAATTAAAA GAAGGTTTGA CGACACATGG 194
    SIMILO11_FLOWER  GCCCTATTCA AGGCACTCCA GAAATTAAAA GAAG------ ---------- 178
      SIMLO11_ FRUIT GCCCTATTCA AGGCACTCCA GAAATTAAAA GAAG------ ---------- 178
                                        320                           340
                                         |                             |
gDNA_Solyc01g102520  ATTGATTCTG TATTTGTAGT TACATTATTT TTTTTTTGGT TAATAAATTG 350
cDNA_Solyc01g102520  ---------- ---------- ---------- ---------- ---------- 205
       SIMLO11_LEAF  ---------- ---------- ---------- ---------- ---------- 178
       SIMLO11_ROOT  ATTGATTCTG TATTTGTAGT TACATTATTT TTTTTT-GGT TAATAAATTG 243
    SIMILO11_FLOWER  ---------- ---------- ---------- ---------- ---------- 178
      SIMLO11_ FRUIT ---------- ---------- ---------- ---------- ---------- 178
                              360                           380                 400
                               |                             |                   |
gDNA_Solyc01g102520  AATTTGCAGA ATTGATGCTT TTGGGATTTA TTTCTCTGCT ATTGACGGTG 400
cDNA_Solyc01g102520  ---------A ATTGATGCTT TTGGGATTTA TTTCTCTGCT ATTGACGGTG 246
       SIMLO11_LEAF  ---------A ATTGATGCTT TTGGGATTTA TTTCTCTGCT ATTGACGGTG 219
       SIMLO11_ROOT  AATTTGCAGA ATTGATGCTT TTGGGATTTA TTTCTCTGCT ATTGACGGTG 293
    SIMILO11_FLOWER  ---------A ATTGATGCTT TTGGGATTTA TTTCTCTGCT ATTGACGGTG 219
      SIMLO11_ FRUIT ---------A ATTGATGCTT TTGGGATTTA TTTCTCTGCT ATTGACGGTG 219
                                        420                           440
                                         |                             |
gDNA_Solyc01g102520  TCTCAAGGGG CAATAAGCCA AATATGTGTT CCTGAAAATA TTTCAAAAGT 450
cDNA_Solyc01g102520  TCTCAAGGGG CAATAAGCCA AATATGTGTT CCTGAAAATA TTTCAAAAGT 296
       SIMLO11_LEAF  TCTCAAGGGG CAATAAGCCA AATATGTGTT CCTGAAAATA TTTCAAAAGT 269
       SIMLO11_ROOT  TCTCAAGGGG CAATAAGCCA AATATGTGTT CCTGAAAATA TTTCAAAAGT 343
    SIMILO11_FLOWER  TCTCAAGGGG CAATAAGCCA AATATGTGTT CCTGAAAATA TTTCAAAAGT 269
      SIMLO11_ FRUIT TCTCAAGGGG CAATAAGCCA AATATGTGTT CCTGAAAATA TTTCAAAAGT 269
                              460                           480                 500
                               |                             |                   |
gDNA_Solyc01g102520  AATGCTTCCA TGCAAGCTTA AAGAAGCTTC AACTTCTAAA CATCTCATCA 500
cDNA_Solyc01g102520  AATGCTTCCA TGCAAGCTTA AAGAAGCTTC AACTTCTAAA CATCTCATCA 346
       SIMLO11_LEAF  AATGCTTCCA TGCAAGCTTA AAGAAGCTTC AACTTCTAAA CATCTCATCA 319
       SIMLO11_ROOT  AATGCTTCCA TGCAAGCTTA AAGAAGCTTC AACTTCTAAA CATCTCATCA 393
    SIMILO11_FLOWER  AATGCTTCCA TGCAAGCTTA AAGAAGCTTC AACTTCTAAA CATCTCATCA 319
      SIMLO11_ FRUIT AATGCTTCCA TGCAAGCTTA AAGAAGCTTC AACTTCTAAA CATCTCATCA 319
                                        520                           540
                                         |                             |
gDNA_Solyc01g102520  CTGGGAGACA TCTTTTAGCT GGATCTAGCG GTGAACAACA TTGTAATCAT 550
cDNA_Solyc01g102520  CTGGGAGACA TCTTTTAGCT GGATCTAGCG GTGAACAACA TTGTAATCAT 396
       SIMLO11_LEAF  CTGGGAGACA TCTTTTAGCT GGATCTAGCG GTGAACAACA TTGTAATCAT 369
       SIMLO11_ROOT  CTGGGAGACA TCTTTTAGCT GGATCTAGCG GTGAACAACA TTGTAATCAT 443
    SIMILO11_FLOWER  CTGGGAGACA TCTTTTAGCT GGATCTAGCG GTGAACAACA TTGTAATCAT 369
      SIMLO11_ FRUIT CTGGGAGACA TCTTTTAGCT GGATCTAGCG GTGAACAACA TTGTAATCAT 369
                              560                           580                 600
                               |                             |                   |
gDNA_Solyc01g102520  CACGAGGTGA ATCATTATTT TCCTTTGTCT AATTACTGTT ATTTCAATTA 600
cDNA_Solyc01g102520  CACGAGG--- ---------- ---------- ---------- ---------- 403
       SIMLO11_LEAF  CACGAGG--- ---------- ---------- ---------- ---------- 376
       SIMLO11_ROOT  CACGAGG--- ---------- ---------- ---------- ---------- 450
    SIMILO11_FLOWER  CACGAGG--- ---------- ---------- ---------- ---------- 376
      SIMLO11_ FRUIT CACGAGG--- ---------- ---------- ---------- ---------- 376
```

```
                                           620                            640
                                             |                              |
gDNA_Solyc01g102520 TAAGATTTAA ATTTATTAAT TTGTGTAGGG AAAAGTTCCA CTGTTATCAC 650
cDNA_Solyc01g102520 ---------- ---------- ---------G AAAAGTTCCA CTGTTATCAC 424
       SIMLO11_LEAF ---------- ---------- ---------G AAAAGTTCCA CTGTTATCAC 397
       SIMLO11_ROOT ---------- ---------- ---------G AAAAGTTCCA CTGTTATCAC 471
    SIMILO11_FLOWER ---------- ---------- ---------G AAAAGTTCCA CTGTTATCAC 397
     SIMLO11_ FRUIT ---------- ---------- ---------G AAAAGTTCCA CTGTTATCAC 397
                                660                            680                            700
                                  |                              |                              |
gDNA_Solyc01g102520 TGGAGGCATT GCATCAACTA CACATTTTCA TATTTGTATT GGCAGTCACA 700
cDNA_Solyc01g102520 TGGAGGCATT GCATCAACTA CACATTTTCA TATTTGTATT GGCAGTCACA 474
       SIMLO11_LEAF TGGAGGCATT GCATCAACTA CACATTTTCA TATTTGTATT GGCAGTCACA 447
       SIMLO11_ROOT TGGAGGCATT GCATCAACTA CACATTTTCA TATTTGTATT GGCAGTCACA 521
    SIMILO11_FLOWER TGGAGGCATT GCATCAACTA CACATTTTCA TATTTGTATT GGCAGTCACA 447
     SIMLO11_ FRUIT TGGAGGCATT GCATCAACTA CACATTTTCA TATTTGTATT GGCAGTCACA 447
                                           720                            740
                                             |                              |
gDNA_Solyc01g102520 CATGTTATCT TCTGTGCCAC CACCATGGTT CTTGGAGGGG CTAAGGTAAA 750
cDNA_Solyc01g102520 CATGTTATCT TCTGTGCCAC CACCATGGTT CTTGGAGGGG CTAAG----- 519
       SIMLO11_LEAF CATGTTATCT TCTGTGCCAC CACCATGGTT CTTGGAGGGG CTAAG----- 492
       SIMLO11_ROOT CATGTTATCT TCTGTGCCAC CACCATGGTT CTTGGAGGGG CTAAG----- 566
    SIMILO11_FLOWER CATGTTATCT TCTGTGCCAC CACCATGGTT CTTGGAGGGG CTAAG----- 492
     SIMLO11_ FRUIT CATGTTATCT TCTGTGCCAC CACCATGGTT CTTGGAGGGG CTAAG----- 492
                                760                            780                            800
                                  |                              |                              |
gDNA_Solyc01g102520 ATATTTAACA ATTAATTCAT TTTCAATTTC TTTCCCAATT TGTAAATTAA 800
cDNA_Solyc01g102520 ---------- ---------- ---------- ---------- ---------- 519
       SIMLO11_LEAF ---------- ---------- ---------- ---------- ---------- 492
       SIMLO11_ROOT ---------- ---------- ---------- ---------- ---------- 566
    SIMILO11_FLOWER ---------- ---------- ---------- ---------- ---------- 492
     SIMLO11_ FRUIT ---------- ---------- ---------- ---------- ---------- 492
                                           820                            840
                                             |                              |
gDNA_Solyc01g102520 AAAAAAACAT ATAAATATTA CACATAAAAA ATACACGAGT AAATATTATA 850
cDNA_Solyc01g102520 ---------- ---------- ---------- ---------- ---------- 519
       SIMLO11_LEAF ---------- ---------- ---------- ---------- ---------- 492
       SIMLO11_ROOT ---------- ---------- ---------- ---------- ---------- 566
    SIMILO11_FLOWER ---------- ---------- ---------- ---------- ---------- 492
     SIMLO11_ FRUIT ---------- ---------- ---------- ---------- ---------- 492
                                860                            880                            900
                                  |                              |                              |
gDNA_Solyc01g102520 GTGTGTAGTG AGTAAGTGAA GTGAAAGAGA GAGTTGAGAC AAAGAAAACA 900
cDNA_Solyc01g102520 ---------- ---------- ---------- ---------- ---------- 519
       SIMLO11_LEAF ---------- ---------- ---------- ---------- ---------- 492
       SIMLO11_ROOT ---------- ---------- ---------- ---------- ---------- 566
    SIMILO11_FLOWER ---------- ---------- ---------- ---------- ---------- 492
     SIMLO11_ FRUIT ---------- ---------- ---------- ---------- ---------- 492
                                           920                            940
                                             |                              |
gDNA_Solyc01g102520 CGCTATGCAT TTATTCGTTT AGTATAAAGA GCCGATTTAC GTAAAATTAT 950
cDNA_Solyc01g102520 ---------- ---------- ---------- ---------- ---------- 519
       SIMLO11_LEAF ---------- ---------- ---------- ---------- ---------- 492
       SIMLO11_ROOT ---------- ---------- ---------- ---------- ---------- 566
    SIMILO11_FLOWER ---------- ---------- ---------- ---------- ---------- 492
     SIMLO11_ FRUIT ---------- ---------- ---------- ---------- ---------- 492
                                960                            980                          1,000
                                  |                              |                              |
gDNA_Solyc01g102520 ATTCAAATAA CTAAGATAAA ATACATCTAT TTGTTTTATT CAACAAAAAA 1000
cDNA_Solyc01g102520 ---------- ---------- ---------- ---------- ---------- 519
       SIMLO11_LEAF ---------- ---------- ---------- ---------- ---------- 492
       SIMLO11_ROOT ---------- ---------- ---------- ---------- ---------- 566
    SIMILO11_FLOWER ---------- ---------- ---------- ---------- ---------- 492
     SIMLO11_ FRUIT ---------- ---------- ---------- ---------- ---------- 492
                                         1,020                          1,040
                                             |                              |
gDNA_Solyc01g102520 AAGGGGGGGG CCATTACTTT ACGTACTTTT CAAAAACACA ACTCATAATT 1050
cDNA_Solyc01g102520 ---------- ---------- ---------- ---------- ---------- 519
       SIMLO11_LEAF ---------- ---------- ---------- ---------- ---------- 492
       SIMLO11_ROOT ---------- ---------- ---------- ---------- ---------- 566
    SIMILO11_FLOWER ---------- ---------- ---------- ---------- ---------- 492
     SIMLO11_ FRUIT ---------- ---------- ---------- ---------- ---------- 492
                              1,060                          1,080                          1,100
                                  |                              |                              |
gDNA_Solyc01g102520 GCATTTAAAC GTATACTTAT GGACTTATCC TTCAAGCCCA AGATCGATAT 1100
cDNA_Solyc01g102520 ---------- ---------- ---------- ---------- ---------- 519
       SIMLO11_LEAF ---------- ---------- ---------- ---------- ---------- 492
       SIMLO11_ROOT ---------- ---------- ---------- ---------- ---------- 566
    SIMILO11_FLOWER ---------- ---------- ---------- ---------- ---------- 492
     SIMLO11_ FRUIT ---------- ---------- ---------- ---------- ---------- 492
                                         1,120                          1,140
                                             |                              |
gDNA_Solyc01g102520 ACTTAACAAT TGTAATTATT GCTAGCATTT TTCCCTAGTC GTTAACTATT 1150
cDNA_Solyc01g102520 ---------- ---------- ---------- ---------- ---------- 519
       SIMLO11_LEAF ---------- ---------- ---------- ---------- ---------- 492
       SIMLO11_ROOT ---------- ---------- ---------- ---------- ---------- 566
    SIMILO11_FLOWER ---------- ---------- ---------- ---------- ---------- 492
     SIMLO11_ FRUIT ---------- ---------- ---------- ---------- ---------- 492
                              1,160                          1,180                          1,200
                                  |                              |                              |
gDNA_Solyc01g102520 ATATTAATAT TCGATTGTGT ATTGTAAAAT TAACCATTTA ACATTTTTTT 1200
cDNA_Solyc01g102520 ---------- ---------- ---------- ---------- ---------- 519
       SIMLO11_LEAF ---------- ---------- ---------- ---------- ---------- 492
       SIMLO11_ROOT ---------- ---------- ---------- ---------- ---------- 566
    SIMILO11_FLOWER ---------- ---------- ---------- ---------- ---------- 492
     SIMLO11_ FRUIT ---------- ---------- ---------- ---------- ---------- 492
```

```
                                  1,220                 1,240
gDNA_Solyc01g102520  AAGAGTAATT TTATCAGTAG ACAGAGTGTC TAGCGGTCTT TTATTGGAAA 1250
cDNA_Solyc01g102520  ---------- ---------- ---------- ---------- ---------- 519
      SIMLO11_LEAF   ---------- ---------- ---------- ---------- ---------- 492
      SIMLO11_ROOT   ---------- ---------- ---------- ---------- ---------- 566
    SIMILO11_FLOWER  ---------- ---------- ---------- ---------- ---------- 492
     SIMLO11_ FRUIT  ---------- ---------- ---------- ---------- ---------- 492

                        1,260                 1,280                 1,300
gDNA_Solyc01g102520  ATAAAAATAT ATCAATAGGA ATAAGTACAA GCAAGGAGGT TAGGCCGTAT 1300
cDNA_Solyc01g102520  ---------- ---------- ---------- ---------- ---------- 519
      SIMLO11_LEAF   ---------- ---------- ---------- ---------- ---------- 492
      SIMLO11_ROOT   ---------- ---------- ---------- ---------- ---------- 566
    SIMILO11_FLOWER  ---------- ---------- ---------- ---------- ---------- 492
     SIMLO11_ FRUIT  ---------- ---------- ---------- ---------- ---------- 492

                                  1,320                 1,340
gDNA_Solyc01g102520  CTTACTATTC CTAATAAGAT AACAAATCTC TACTAATTAA CAAGCATCGA 1350
cDNA_Solyc01g102520  ---------- ---------- ---------- ---------- ---------- 519
      SIMLO11_LEAF   ---------- ---------- ---------- ---------- ---------- 492
      SIMLO11_ROOT   ---------- ---------- ---------- ---------- ---------- 566
    SIMILO11_FLOWER  ---------- ---------- ---------- ---------- ---------- 492
     SIMLO11_ FRUIT  ---------- ---------- ---------- ---------- ---------- 492

                        1,360                 1,380                 1,400
gDNA_Solyc01g102520  AAAAATAAGA ACTAGAGAAA ACTACGTATA TGTATGATTG TCACGAAGTT 1400
cDNA_Solyc01g102520  ---------- ---------- ---------- ---------- ---------- 519
      SIMLO11_LEAF   ---------- ---------- ---------- ---------- ---------- 492
      SIMLO11_ROOT   ---------- ---------- ---------- ---------- ---------- 566
    SIMILO11_FLOWER  ---------- ---------- ---------- ---------- ---------- 492
     SIMLO11_ FRUIT  ---------- ---------- ---------- ---------- ---------- 492

                                  1,420                 1,440
gDNA_Solyc01g102520  TATAACCCTC AACAAATGAG CAATACCTAA TTAGTTCTCT TTTATTTTTT 1450
cDNA_Solyc01g102520  ---------- ---------- ---------- ---------- ---------- 519
      SIMLO11_LEAF   ---------- ---------- ---------- ---------- ---------- 492
      SIMLO11_ROOT   ---------- ---------- ---------- ---------- ---------- 566
    SIMILO11_FLOWER  ---------- ---------- ---------- ---------- ---------- 492
     SIMLO11_ FRUIT  ---------- ---------- ---------- ---------- ---------- 492

                        1,460                 1,480                 1,500
gDNA_Solyc01g102520  TGGGCCTCAA ATAGATACAA CAATGGAGAC ACTGGGAGAA CTCAATTCAA 1500
cDNA_Solyc01g102520  ---------- ----ATACAA CAATGGAGAC ACTGGGAGAA CTCAATTCAA 555
      SIMLO11_LEAF   ---------- ----ATACAA CAATGGAGAC ACTGGGAGAA CTCAATTCAA 528
      SIMLO11_ROOT   ---------- ----ATACAA CAATGGAGAC ACTGGGAGAA CTCAATTCAA 602
    SIMILO11_FLOWER  ---------- ----ATACAA CAATGGAGAC ACTGGGAGAA CTCAATTCAA 528
     SIMLO11_ FRUIT  ---------- ----ATACAA CAATGGAGAC ACTGGGAGAA CTCAATTCAA 528

                                  1,520                 1,540
gDNA_Solyc01g102520  AAGCAATCAA AACCACATCA TGGTATAATT ATATTAGTTA ATTATTGTTT 1550
cDNA_Solyc01g102520  AAGCAATCAA AACCACATCA TG-------- ---------- ---------- 577
      SIMLO11_LEAF   AAGCAATCAA AGCCACATCA TG-------- ---------- ---------- 550
      SIMLO11_ROOT   AAGCAATCAA AACCGCATCA TG-------- ---------- ---------- 624
    SIMILO11_FLOWER  AAGCAATCAA AACCACATCA TG-------- ---------- ---------- 550
     SIMLO11_ FRUIT  AAGCAATCAA AACCACATCA TG-------- ---------- ---------- 550

                        1,560                 1,580                 1,600
gDNA_Solyc01g102520  ATACAATTTT GTCTGTAATT TTTAACGAAT TATTACCATG ATATTTTCTA 1600
cDNA_Solyc01g102520  ---------- ---------- ---------- ---------- ---------- 577
      SIMLO11_LEAF   ---------- ---------- ---------- ---------- ---------- 550
      SIMLO11_ROOT   ---------- ---------- ---------- ---------- ---------- 624
    SIMILO11_FLOWER  ---------- ---------- ---------- ---------- ---------- 550
     SIMLO11_ FRUIT  ---------- ---------- ---------- ---------- ---------- 550

                                  1,620                 1,640
gDNA_Solyc01g102520  TGTGTAGTGC ATATTCTCCA TATTCAATCA TTTGTGGATA GAGCTGGTAA 1650
cDNA_Solyc01g102520  -------TGC ATATTCTCCA TATTCAATCA TTTGTGGATA GAGCTGGTAA 620
      SIMLO11_LEAF   -------TGC ATATTCTCCA TATTCAATCA TTTGTGGATA GAGCTGGTAA 593
      SIMLO11_ROOT   -------TGC ATATTCTCCA TATTCAATCA TTTGTGGATA GAGCTGGTAA 667
    SIMILO11_FLOWER  -------TGC ATATTCTCCA TATTCAATCA TTTGTGGATA GAGCTGGTAA 593
     SIMLO11_ FRUIT  -------TGC ATATTCTCCA TATTCAATCA TTTGTGGATA GAGCTGGTAA 593

                        1,660                 1,680                 1,700
gDNA_Solyc01g102520  ACGTTGGAGA AAGTACGCTC TCATTAGTTG GACGGTACGA TGTTTTATTT 1700
cDNA_Solyc01g102520  ACGTTGGAGA AAGTACGCTC TCATTAGTTG GACGGT---- ---------- 656
      SIMLO11_LEAF   ACGTTGGAGA AAGTACGCTC TCATTAGTTG GACGGT---- ---------- 629
      SIMLO11_ROOT   ACGTTGGAGA AAGTACGCTC TCATTAGTTG GACGGT---- ---------- 703
    SIMILO11_FLOWER  ACGTTGGAGA AAGTACGCTC TCATTAGTTG GACGGT---- ---------- 629
     SIMLO11_ FRUIT  ACGTTGGAGA AAGTACGCTC TCATTAGTTG GACGGT---- ---------- 629

                                  1,720                 1,740
gDNA_Solyc01g102520  ATTAAAGATC GATAATAATT ATGTATTTTA CTCAAAGAAA AATGACGATT 1750
cDNA_Solyc01g102520  ---------- ---------- ---------- ---------- ---------- 656
      SIMLO11_LEAF   ---------- ---------- ---------- ---------- ---------- 629
      SIMLO11_ROOT   ---------- ---------- ---------- ---------- ---------- 703
    SIMILO11_FLOWER  ---------- ---------- ---------- ---------- ---------- 629
     SIMLO11_ FRUIT  ---------- ---------- ---------- ---------- ---------- 629

                        1,760                 1,780                 1,800
gDNA_Solyc01g102520  TTTCATGTGT ATGAATATAA AAAAAAAATA TATATAGGTA GCATTTTTCA 1800
cDNA_Solyc01g102520  ---------- ---------- ---------- ---------A GCATTTTTCA 667
      SIMLO11_LEAF   ---------- ---------- ---------- ---------A GCATTTTTCA 640
      SIMLO11_ROOT   ---------- ---------- ---------- ---------A GCATTTTTCA 714
    SIMILO11_FLOWER  ---------- ---------- ---------- ---------A GCATTTTTCA 640
     SIMLO11_ FRUIT  ---------- ---------- ---------- ---------A GCATTTTTCA 640
```

```
                                    1,820                          1,840
gDNA_Solyc01g102520  AACAATTTTA TGGTTCAGTA ACAAAGTCAG ACTATATTGT CTTGCGGACT 1850
cDNA_Solyc01g102520  AACAATTTTA TGGTTCAGTA ACAAAGTCAG ACTATATTGT CTTGCGGACT 717
       SIMLO11_LEAF  AACAATTTTA TGGTTCAGTA ACAAAGTCAG ACTATATTGT CTTGCGGACT 690
       SIMLO11_ROOT  AACAATTTTA TGGTTCAGTA ACAAAGTCAG ACTATATTGT CTTGCGGACT 764
    SIMILO11_FLOWER  AACAATTTTA TGGTTCAGTA ACAAAGTCAG ACTATATTGT CTTGCGGACT 690
     SIMLO11_ FRUIT  AACAATTTTA TGGTTCAGTA ACAAAGTCAG ACTATATTGT CTTGCGGACT 690

                          1,860                          1,880                    1,900
gDNA_Solyc01g102520  GGATTTATCA AGGTAAGCAA TACTTAATTC TTTCTATTTT ATCTTTCATT 1900
cDNA_Solyc01g102520  GGATTTATCA AG-------- ---------- ---------- ---------- 729
       SIMLO11_LEAF  GGATTTATCA AG-------- ---------- ---------- ---------- 702
       SIMLO11_ROOT  GGATTTATCA AG-------- ---------- ---------- ---------- 776
    SIMILO11_FLOWER  GGATTTATCA AG-------- ---------- ---------- ---------- 702
     SIMLO11_ FRUIT  GGATTTATCA AG-------- ---------- ---------- ---------- 702

                                    1,920                          1,940
gDNA_Solyc01g102520  TTTATAATTA CTTTGTGTCA ATTCTATGAT TTTAATTAGT CGCAATTATA 1950
cDNA_Solyc01g102520  ---------- ---------- ---------- ---------- ---------- 729
       SIMLO11_LEAF  ---------- ---------- ---------- ---------- ---------- 702
       SIMLO11_ROOT  ---------- ---------- ---------- ---------- ---------- 776
    SIMILO11_FLOWER  ---------- ---------- ---------- ---------- ---------- 702
     SIMLO11_ FRUIT  ---------- ---------- ---------- ---------- ---------- 702

                          1,960                          1,980                    2,000
gDNA_Solyc01g102520  ATGCATTTTA TCGTGTGATA ATAGAAACAT TGTCCATCCA ATCCTACGTA 2000
cDNA_Solyc01g102520  ---------- ---------- ----AAACAT TGTCCATCCA ATCCTACGTA 755
       SIMLO11_LEAF  ---------- ---------- ----AAACAT TGTCCATCCA ATCCTACGTA 728
       SIMLO11_ROOT  ---------- ---------- ----AAACAT TGTCCATCCA ATCCTACGTA 802
    SIMILO11_FLOWER  ---------- ---------- ----AAACAT TGTCCATCCA ATCCTACGTA 728
     SIMLO11_ FRUIT  ---------- ---------- ----AAACAT TGTCCATCCA ATCCTACGTA 728

                                    2,020                          2,040
gDNA_Solyc01g102520  TAATTTTCAC CGATATATTT TGCGGACACT GGAGCATGAC TTCAAAAAGA 2050
cDNA_Solyc01g102520  TAATTTTCAC CGATATATTT TGCGGACACT GGAGCATGAC TTCAAAAAGA 805
       SIMLO11_LEAF  TAATTTTCAC CGATATATTT TGCGGACACT GGAGCATGAC TTCAAAAAGA 778
       SIMLO11_ROOT  TAATTTTCAC CGATATATTT TGCGGACACT GGAGCATGAC TTCAAAAAGA 852
    SIMILO11_FLOWER  TAATTTTCAC CGATATATTT TGCGGACACT GGAGCATGAC TTCAAAAAGA 778
     SIMLO11_ FRUIT  TAATTTTCAC CGATATATTT TGCGGACACT GGAGCATGAC TTCAAAAAGA 778

                          2,060                          2,080                    2,100
gDNA_Solyc01g102520  TTGTTGGAAT CAGGTAAATT AACACTACAT ATACTAATTA TGTATAGGAT 2100
cDNA_Solyc01g102520  TTGTTGGAAT CAG------- ---------- ---------- ---------- 818
       SIMLO11_LEAF  TTGTTGGAAT CAG------- ---------- ---------- ---------- 791
       SIMLO11_ROOT  TTGTTGGAAT CAG------- ---------- ---------- ---------- 865
    SIMILO11_FLOWER  TTGTTGGAAT CAG------- ---------- ---------- ---------- 791
     SIMLO11_ FRUIT  TTGTTGGAAT CAG------- ---------- ---------- ---------- 791

                                    2,120                          2,140
gDNA_Solyc01g102520  AATTACTTGG TAATTTATAT TTAATTATGT TTAATCGAGC TAATTTATGC 2150
cDNA_Solyc01g102520  ---------- ---------- ---------- ---------- ---------- 818
       SIMLO11_LEAF  ---------- ---------- ---------- ---------- ---------- 791
       SIMLO11_ROOT  ---------- ---------- ---------- ---------- ---------- 865
    SIMILO11_FLOWER  ---------- ---------- ---------- ---------- ---------- 791
     SIMLO11_ FRUIT  ---------- ---------- ---------- ---------- ---------- 791

                          2,160                          2,180                    2,200
gDNA_Solyc01g102520  TTTTTGGAAA TTGATCTTGA ACAGCTGGTA CTTGTGGCTT TTTGTTGTTC 2200
cDNA_Solyc01g102520  ---------- ---------- ----CTGGTA CTTGTGGCTT TTTGTTGTTC 844
       SIMLO11_LEAF  ---------- ---------- ----CTGGTA CTTGTGGCTT TTTGTTGTTC 817
       SIMLO11_ROOT  ---------- ---------- ----CTGGTA CTTGTGGCTT TTTGTTGTTC 891
    SIMILO11_FLOWER  ---------- ---------- ----CTGGTA CTTGTGGCTT TTTGTTGTTC 817
     SIMLO11_ FRUIT  ---------- ---------- ----CTGGTA CTTGTGGCTT TTTGTTGTTC 817

                                    2,220                          2,240
gDNA_Solyc01g102520  TGTTTTTGTT GATCAACATT GCAGGTATTA ATTTTTTATC CTAAGTATTA 2250
cDNA_Solyc01g102520  TGTTTTTGTT GATCAACATT GCAGG----- ---------- ---------- 869
       SIMLO11_LEAF  TGTTTTTGTT GATCAACATT GCAGG----- ---------- ---------- 842
       SIMLO11_ROOT  TGTTTTTGTT GATCAACATT GCAGG----- ---------- ---------- 916
    SIMILO11_FLOWER  TGTTTTTGTT GATCAACATT GCAGG----- ---------- ---------- 842
     SIMLO11_ FRUIT  TGTTTTTGTT GATCAACATT GCAGG----- ---------- ---------- 842

                          2,260                          2,280                    2,300
gDNA_Solyc01g102520  CTAAATCTTA CTCCTAATTT TGCTACTTTT TTTTTTTTAA TATCTTATTA 2300
cDNA_Solyc01g102520  ---------- ---------- ---------- ---------- ---------- 869
       SIMLO11_LEAF  ---------- ---------- ---------- ---------- ---------- 842
       SIMLO11_ROOT  ---------- ---------- ---------- ---------- ---------- 916
    SIMILO11_FLOWER  ---------- ---------- ---------- ---------- ---------- 842
     SIMLO11_ FRUIT  ---------- ---------- ---------- ---------- ---------- 842

                                    2,320                          2,340
gDNA_Solyc01g102520  TATATGTTTC GCTTAAACTA ATGTATGGTT TAAAATATGC AGGATGGCAC 2350
cDNA_Solyc01g102520  ---------- ---------- ---------- ---------- ---ATGGCAC 876
       SIMLO11_LEAF  ---------- ---------- ---------- ---------- ---ATGGCAC 849
       SIMLO11_ROOT  ---------- ---------- ---------- ---------- ---ATGGCAC 923
    SIMILO11_FLOWER  ---------- ---------- ---------- ---------- ---ATGGCAC 849
     SIMLO11_ FRUIT  ---------- ---------- ---------- ---------- ---ATGGCAC 849

                          2,360                          2,380                    2,400
gDNA_Solyc01g102520  TCTTACTTTT GGCTATCATT TTTACCTCTT GTTGTAAGTG ACCATTGAAA 2400
cDNA_Solyc01g102520  TCTTACTTTT GGCTATCATT TTTACCTCTT GTT------- ---------- 909
       SIMLO11_LEAF  TCTTACTTTT GGCTATCATT TTTACCTCTT GTT------- ---------- 882
       SIMLO11_ROOT  TCTTACTTTT GGCTATCATT TTTACCTCTT GTT------- ---------- 956
    SIMILO11_FLOWER  TCTTACTTTT GGCTATCATT TTTACCTCTT GTT------- ---------- 882
     SIMLO11_ FRUIT  TCTTACTTTT GGCTATCATT TTTACCTCTT GTT------- ---------- 882
```

```
                                               2,420                         2,440
gDNA_Solyc01g102520  AACTGGTTTG CTGCATCTGT TTGAATAAAT TGGGATAAGA CTATATGAAT 2450
cDNA_Solyc01g102520  ---------- ---------- ---------- ---------- ---------- 909
       SIMLO11_LEAF  ---------- ---------- ---------- ---------- ---------- 882
       SIMLO11_ROOT  ---------- ---------- ---------- ---------- ---------- 956
    SIMILO11_FLOWER  ---------- ---------- ---------- ---------- ---------- 882
      SIMLO11_ FRUIT ---------- ---------- ---------- ---------- ---------- 882
                                2,460                         2,480                         2,500
gDNA_Solyc01g102520  CACCACTGTC CATATTTGAC GATGTGTATA TTTTTGATAA AATTTATGCA 2500
cDNA_Solyc01g102520  ---------- ---------- ---------- ---------- ---------- 909
       SIMLO11_LEAF  ---------- ---------- ---------- ---------- ---------- 882
       SIMLO11_ROOT  ---------- ---------- ---------- ---------- ---------- 956
    SIMILO11_FLOWER  ---------- ---------- ---------- ---------- ---------- 882
      SIMLO11_ FRUIT ---------- ---------- ---------- ---------- ---------- 882
                                               2,520                         2,540
gDNA_Solyc01g102520  GCTACTACTT CTAGTAGGAA CAAAATTGGA GCACATAATA ACAGAATTGG 2550
cDNA_Solyc01g102520  -CTACTACTT CTAGTAGGAA CAAAATTGGA GCACATAATA ACAGAATTGG 958
       SIMLO11_LEAF  -CTACTACTT CTAGTAGGAA CAAAATTGGA GCACATAATA ACAGAATTGG 931
       SIMLO11_ROOT  -CTACTACTT CTAGTAGGAA CAAAATTGGA GCACATAATA ACAGAATTGG 1005
    SIMILO11_FLOWER  -CTACTACTT CTAGCAGGAA CAAAATTGGA GCACATAATA ACAGAATTGG 931
      SIMLO11_ FRUIT -CTACTACTT CTAGTAGGAA CAAAATTGGA GCACATAATA ACAGAATTGG 931
                                2,560                         2,580                         2,600
gDNA_Solyc01g102520  CTCAAGAGGT TTCAGAGAGG TCATCAGTAG TAGATGAAAC CACACCAATT 2600
cDNA_Solyc01g102520  CTCAAGAGGT TTCAGAGAGG TCATCAGTAG TAGATGAAAC CACACCAATT 1008
       SIMLO11_LEAF  CTCAAGAGGT TTCAGAGAGG TCATCAGTAG TAGATGAAAC CACACCAATT 981
       SIMLO11_ROOT  CTCAAGAGGT TTCAGAGAGG TCATCAGTAG TAGATGAAAC CACACCAATT 1055
    SIMILO11_FLOWER  CTCAAGAGGT TTCAGAGAGG TCATCAGTAG TAGATGAAAC CACACCAATT 981
      SIMLO11_ FRUIT CTCAAGAGGT TTCAGAGAGG TCATCAGTAG TAGATGAAAC CACACCAATT 981
                                               2,620                         2,640
gDNA_Solyc01g102520  AAACCTTCTG ATGAGTTGTT TTGGTTTGAC AGTCCAAATC TTGTCCTTTA 2650
cDNA_Solyc01g102520  AAACCTTCTG ATGAGTTGTT TTGGTTTGAC AGTCCAAATC TTGTCCTTTA 1058
       SIMLO11_LEAF  AAACCTTCTG ATGAGTTGTT TTGGTTTGAC AGTCCAAACC TTGTCCTTTA 1031
       SIMLO11_ROOT  AAACCTTCTG ATGAGTTGTT TTGGTTTGAC AGTCCAAATC TTGTCCTTTA 1105
    SIMILO11_FLOWER  AAACCTTCTG ATGAGTTGTT TTGGTTTGAC AGTCCAAATC TTGTCCTTTA 1031
      SIMLO11_ FRUIT AAACCTTCTG ATGAGTTGTT TTGGTTTGAC AGTCCAAATC TTGTCCTTTA 1031
                                2,660                         2,680                         2,700
gDNA_Solyc01g102520  CCTAATCCAC TTCATTTTGT TCCAAAACTC CTTTGAGATT GCTTTCTTTG 2700
cDNA_Solyc01g102520  CCTAATCCAC TTCATTTTGT TCCAAAACTC CTTTGAGATT GCTTTCTTTG 1108
       SIMLO11_LEAF  CCTAATCCAC TTCATTTTGT TCCAAGACTC CTTTGAGATT GCTTTCTTTG 1081
       SIMLO11_ROOT  CCTAATCCAC TTCATTTTGT TCCAAAACTC CTTTGAGATT GCTTTCTTTG 1155
    SIMILO11_FLOWER  CCTAATCCAC TTCATTTTGT TCCAAAACTC CTTTGAGATT GCTTTCTTTG 1081
      SIMLO11_ FRUIT CCTAATCCAC TTCATTTTGT TCCAAAACTC CTTTGAGATT GCTTTCTTTG 1081
                                               2,720                         2,740
gDNA_Solyc01g102520  TCTGGATTTG GGTAATAACT TTTAACTTCT TTTATTTCAA CTTAACTTCA 2750
cDNA_Solyc01g102520  TCTGGATTTG G--------- ---------- ---------- ---------- 1119
       SIMLO11_LEAF  TCTGGATTTG G--------- ---------- ---------- ---------- 1092
       SIMLO11_ROOT  TCTGGATTTG G--------- ---------- ---------- ---------- 1166
    SIMILO11_FLOWER  TCTGGATTTG G--------- ---------- ---------- ---------- 1092
      SIMLO11_ FRUIT TCTGGATTTG G--------- ---------- ---------- ---------- 1092
                                2,760                         2,780                         2,800
gDNA_Solyc01g102520  ATCACTTTTC TTCTTTTCTA ACATGAAAAA AACTGTCATT TGACAATTCT 2800
cDNA_Solyc01g102520  ---------- ---------- ---------- ---------- ---------- 1119
       SIMLO11_LEAF  ---------- ---------- ---------- ---------- ---------- 1092
       SIMLO11_ROOT  ---------- ---------- ---------- ---------- ---------- 1166
    SIMILO11_FLOWER  ---------- ---------- ---------- ---------- ---------- 1092
      SIMLO11_ FRUIT ---------- ---------- ---------- ---------- ---------- 1092
                                               2,820                         2,840
gDNA_Solyc01g102520  CCAGTGTACT TATGGATTCA AATCATGCAT CATGGAAGAT TTGGGTTTCA 2850
cDNA_Solyc01g102520  ----TGTACT TATGGATTCA AATCATGCAT CATGGAAGAT TTGGGTTTCA 1165
       SIMLO11_LEAF  ----TGTACT TATGGATTCA AATCATGCAT CATGGAAGAT TTGGGTTTCA 1138
       SIMLO11_ROOT  ----TGTACT TATGGATTCA AATCATGCAT CATGGAAGAT TTGGGTTTCA 1212
    SIMILO11_FLOWER  ----TGTACT TATGGATTCA AATCATGCAT CATGGAAGAT TTGGGTTTCA 1138
      SIMLO11_ FRUIT ----TGTACT TATGGATTCA AATCATGCAT CATGGAAGAT TTGGGTTTCA 1138
                                2,860                         2,880                         2,900
gDNA_Solyc01g102520  TTATTCCAAG ACTTATTATT GGGTAAGCTA CTCAAATCCC ATATAGTAAT 2900
cDNA_Solyc01g102520  TTATTCCAAG ACTTATTATT GGG------- ---------- ---------- 1188
       SIMLO11_LEAF  TTATTCCAAG ACTTATTATT GGG------- ---------- ---------- 1161
       SIMLO11_ROOT  TTATTCCAAG ACTTATTATT GGG------- ---------- ---------- 1235
    SIMILO11_FLOWER  TTATTCCAAG ACTTATTATT GGG------- ---------- ---------- 1161
      SIMLO11_ FRUIT TTATTCCAAG ACTTATTATT GGG------- ---------- ---------- 1161
                                               2,920                         2,940
gDNA_Solyc01g102520  ATGTTTCTGA CAGAAAGTGA CCATGTAGAT TTTGTGGTGC CACAGGGTGA 2950
cDNA_Solyc01g102520  ---------- ---------- ---------- ---------- ------GTGA 1192
       SIMLO11_LEAF  ---------- ---------- ---------- ---------- ------GTGA 1165
       SIMLO11_ROOT  ---------- ---------- ---------- ---------- ------GTGA 1239
    SIMILO11_FLOWER  ---------- ---------- ---------- ---------- ------GTGA 1165
      SIMLO11_ FRUIT ---------- ---------- ---------- ---------- ------GTGA 1165
                                2,960                         2,980                         3,000
gDNA_Solyc01g102520  TTGTTCAAGT TCTTTGCAGT TACAGTACTC TGCCCTTATA TGCTCTTGTC 3000
cDNA_Solyc01g102520  TTGTTCAAGT TCTTTGCAGT TACAGTACTC TGCCCTTATA TGCTCTTGTC 1242
       SIMLO11_LEAF  TTGTTCAAGT TCTTTGCAGT TACAGTACTC TGCCCTTATA TGCTCTTGTC 1215
       SIMLO11_ROOT  TTGTTCAAGT TCTTTGCAGT TACAGTACTC TGCCCTTATA TGCTCTTGTC 1289
    SIMILO11_FLOWER  TTGTTCAAGT TCTTTGCAGT TACAGTACTC TGCCCTTATA TGCTCTTGTC 1215
      SIMLO11_ FRUIT TTGTTCAAGT TCTTTGCAGT TACAGTACTC TGCCCTTATA TGCTCTTGTC 1215
```

```
                                      3,020                         3,040
gDNA_Solyc01g102520  ACACAGGTAA ATAAATACCT GTCTCATTCT GTTTACTCAG ACGCCTCTTT 3050
cDNA_Solyc01g102520  ACACAG---- ---------- ---------- ---------- ---------- 1248
       SIMLO11_LEAF  ACACAG---- ---------- ---------- ---------- ---------- 1221
       SIMLO11_ROOT  ACACAG---- ---------- ---------- ---------- ---------- 1295
    SIMILO11_FLOWER  ACACAG---- ---------- ---------- ---------- ---------- 1221
      SIMLO11_ FRUIT ACACAG---- ---------- ---------- ---------- ---------- 1221

                          3,060                         3,080                 3,100
gDNA_Solyc01g102520  CTTATTGAGG ACTCTTTTTT TTTCCACAAC AGATGGGAAC CACATATAAG 3100
cDNA_Solyc01g102520  ---------- ---------- ---------- --ATGGGAAC CACATATAAG 1266
       SIMLO11_LEAF  ---------- ---------- ---------- --ATGGGAAC CACATATAAG 1239
       SIMLO11_ROOT  ---------- ---------- ---------- --ATGGGAAC CACATATAAG 1313
    SIMILO11_FLOWER  ---------- ---------- ---------- --ATGGGAAC CACATATAAG 1239
      SIMLO11_ FRUIT ---------- ---------- ---------- --ATGGGAAC CACATATAAG 1239

                                      3,120                         3,140
gDNA_Solyc01g102520  AGAGGAATAT TTGGTGAGCA GACAGAAGAT TCTCTGAGGA TATGGGCTGG 3150
cDNA_Solyc01g102520  AGAGGAATAT TTGGTGAGCA GACAGAAGAT TCTCTGAGGA TATGGGCTGG 1316
       SIMLO11_LEAF  AGAGGAATAT TTGGTGAGCA GACAGAAGAT TCTCTGAGGA TATGGGCTGG 1289
       SIMLO11_ROOT  AGAGGAATAT TTGGTGAGCA GACAGAAGAT TCTCTGAGGA TATGGGCTGG 1363
    SIMILO11_FLOWER  AGAGGAATAT TTGGTGAGCA GACAGAAGAT TCTCTGAGGA TATGGGCTGG 1289
      SIMLO11_ FRUIT AGAGGAATAT TTGGTGAGCA GACAGAAGAT TCTCTGAGGA TATGGGCTGG 1289

                          3,160                         3,180                 3,200
gDNA_Solyc01g102520  TACATCAAGC CACAATAAGA TGGTCACAGA AACACAAGAA AGCATCACTG 3200
cDNA_Solyc01g102520  TACATCAAGC CACAATAAGA TGGTCACAGA AACACAAGAA AGCATCACTG 1366
       SIMLO11_LEAF  TACATCAAGC CACAATAAGA TGGTCACAGA AACACAAGAA AGCATCACTG 1339
       SIMLO11_ROOT  TACATCAAGC CACAATAAGA TGGTCACAGA AACACAAGAA AGCATCACTG 1413
    SIMILO11_FLOWER  TACATCAAGC CACAATAAGA TGGTCACAGA AACACAAGAA AGCATCACTG 1339
      SIMLO11_ FRUIT TACATCAAGC CACAATAAGA TGGTCACAGA AACACAAGAA AGCATCACTG 1339

                                      3,220                         3,240
gDNA_Solyc01g102520  TTTCTAACAG ACCTGCATGC AATGAAATTC AAGACATTTC ATCAACTGTT 3250
cDNA_Solyc01g102520  TTTCTAACAG ACCTGCATGC AATGAAATTC AAGACATTTC ATCAACTGTT 1416
       SIMLO11_LEAF  TTTCTAACAG ACCTGCATGC AATGAAATTC AAGACATTTC ATCAACTGTT 1389
       SIMLO11_ROOT  TTTCTAACAG ACCTGCATGC AATGAAATTC AAGACATTTC ATCAACTGTT 1463
    SIMILO11_FLOWER  TTTCTAACAG ACCTGCATGC AATGAAATTC AAGACATTTC ATCAACTGTT 1389
      SIMLO11_ FRUIT TTTCTAACAG ACCTGCATGC AATGAAATTC AAGACATTTC ATCAACTGTT 1389

                          3,260                         3,280                 3,300
gDNA_Solyc01g102520  GAGCTTTCTT ATCCAAACAA GCCTCACACT ACTCCTTAGT TGTACAGTTA 3300
cDNA_Solyc01g102520  GAGCTTTCTT ATCCAAACAA GCCTCACACT ACTCCTTAGT TGTACAGTTA 1466
       SIMLO11_LEAF  GAGCTTTCTT ATCCAAACAA GCCTCACACT ACTCCTTAG- ---------- 1428
       SIMLO11_ROOT  GAGCTTTCTT ATCCAAACAA GCCTCACACT ACTCCTTAG- ---------- 1502
    SIMILO11_FLOWER  GAGCTTTCTT ATCCAAACAA GCCTCACACT ACTCCTTAG- ---------- 1428
      SIMLO11_ FRUIT GAGCTTTCTT ATCCAAACAA GCCTCACACT ACTCCTTAG- ---------- 1428

                                      3,320                         3,340
gDNA_Solyc01g102520  CTGGTTTTTC TTGAGTGTAA AAAATAAGGA TGAACAATAC ATTATTCTTT 3350
cDNA_Solyc01g102520  CTGGTTTTTC TTGAGTGTAA AAAATAAGGA TGAACAATAC ATTATTCTTT 1516
       SIMLO11_LEAF  ---------- ---------- ---------- ---------- ---------- 1428
       SIMLO11_ROOT  ---------- ---------- ---------- ---------- ---------- 1502
    SIMILO11_FLOWER  ---------- ---------- ---------- ---------- ---------- 1428
      SIMLO11_ FRUIT ---------- ---------- ---------- ---------- ---------- 1428

                          3,360                         3,380                 3,400
gDNA_Solyc01g102520  AAAGAGTAAA CATTGTTAAC TTTTCACTGA GAGGGAGAGA TGTTCATGCT 3400
cDNA_Solyc01g102520  AAAGAGTAAA CATTGTTAAC TTTTCACTGA GAGGGAGAGA TGTTCATGCT 1566
       SIMLO11_LEAF  ---------- ---------- ---------- ---------- ---------- 1428
       SIMLO11_ROOT  ---------- ---------- ---------- ---------- ---------- 1502
    SIMILO11_FLOWER  ---------- ---------- ---------- ---------- ---------- 1428
      SIMLO11_ FRUIT ---------- ---------- ---------- ---------- ---------- 1428

                                      3,420                         3,440
gDNA_Solyc01g102520  GCCTAATCAA ATGTTGTCAT CATTTTCCAA TATCAATTAG CCCAGGCATC 3450
cDNA_Solyc01g102520  GCCTAATCAA ATGTTGTCAT CATTTTCCAA TATCAATTAG CCCAGGCATC 1616
       SIMLO11_LEAF  ---------- ---------- ---------- ---------- ---------- 1428
       SIMLO11_ROOT  ---------- ---------- ---------- ---------- ---------- 1502
    SIMILO11_FLOWER  ---------- ---------- ---------- ---------- ---------- 1428
      SIMLO11_ FRUIT ---------- ---------- ---------- ---------- ---------- 1428

                          3,460                         3,480                 3,500
gDNA_Solyc01g102520  ATCTCTAGTT ACACAACACC TATTTTTTTT TCTTCTGCGA AAAGCATCTC 3500
cDNA_Solyc01g102520  ATCTCTAGTT ACACAACACC TATTTTTTTT TCTTCTGCGA AAAGCATCTC 1666
       SIMLO11_LEAF  ---------- ---------- ---------- ---------- ---------- 1428
       SIMLO11_ROOT  ---------- ---------- ---------- ---------- ---------- 1502
    SIMILO11_FLOWER  ---------- ---------- ---------- ---------- ---------- 1428
      SIMLO11_ FRUIT ---------- ---------- ---------- ---------- ---------- 1428

                                      3,520                         3,540
gDNA_Solyc01g102520  CCAATTTCCT AGTTACAAAT ATGGAGGGAC GCATAAAACA TTTCCCTCTT 3550
cDNA_Solyc01g102520  CCAATTTCCT AGTTACAAAT ATGGAGGGAC GCATAAAACA TTTCCCTCTT 1716
       SIMLO11_LEAF  ---------- ---------- ---------- ---------- ---------- 1428
       SIMLO11_ROOT  ---------- ---------- ---------- ---------- ---------- 1502
    SIMILO11_FLOWER  ---------- ---------- ---------- ---------- ---------- 1428
      SIMLO11_ FRUIT ---------- ---------- ---------- ---------- ---------- 1428

                          3,560                       3,580
gDNA_Solyc01g102520  TAGCTCAAAT TATTTACACA CTAGTTATCT » 3580
cDNA_Solyc01g102520  TAGCTCAAAT TATTTACACA CTAGTTATCT » 1746
       SIMLO11_LEAF  ---------- ---------- ---------- 1428
       SIMLO11_ROOT  ---------- ---------- ---------- 1502
    SIMILO11_FLOWER  ---------- ---------- ---------- 1428
      SIMLO11_ FRUIT ---------- ---------- ---------- 1428
```

```
                                       20                            40
gDNA_Solyc10g044510  ATGGAAGGAG CTTTTAGGGA AGGAAGGTCA TTGAAGGAAA CACCAACATG 50
cDNASolyc10g044510   ATGGAAGGAG CTTTTAGGGA AGGAAGGTCA TTGAAGGAAA CACCAACATG 50
SlMLO13_LEAF         ATGGAAGGAG CTTTTAGGGA AGGAAGGTCA TTGAAGGAAA CACCAACATG 50
SlMLO13_ROOT         ATGGAAGGAG CTTTTAGGGA AGGAAGGTCA TTGAAGGAAA CACCAACACG 50
SlMLO13_FLOWER       ATGGAAGGAG CTTTTAGGGA AGGAAGGTCA TTGAAGGAAA CACCAACATG 50
SlMLO13_FRUIT        ATGGAAGGAG CTTTTAGGGA AGGAAGGTCA TTGAAGGAAA CACCAACAYG 50
                            60                            80                           100
gDNA_Solyc10g044510  GTCAGTTGCA ACAGTCACCA CTGTTATGGT TTTTGTTTGC TTGTTTGCCC 100
cDNASolyc10g044510   GTCAGTTGCA ACAGTCACCA CTGTTATGGT TTTTGTTTGC TTGTTTGCCC 100
SlMLO13_LEAF         GTCAGTTGCA ACAGTCACCA CTGTTATGGT TTTTGTTTGC TTGTTTGCCC 100
SlMLO13_ROOT         GTCAGTTGCA ACAGTCACCA CTGTTATGGT TTTTGTTTGC TTGTTTGCCC 100
SlMLO13_FLOWER       GTCAGTTGCA ACAGTCACCA CTGTTATGGT TTTTGTTTGC TTGTTTGCCC 100
SlMLO13_FRUIT        GTCAGTTGCA ACAGTCACCA CTGTTATGGT TTTTGTTTGC TTGTTTGCCC 100
                                       120                           140
gDNA_Solyc10g044510  AACGCTCCAT TTATCGATTC GGAAGGGTGA GTAGTAGTCA CTATTGGACT 150
cDNASolyc10g044510   AACGCTCCAT TTATCGATTC GGAAGG---- ---------- ---------- 126
SlMLO13_LEAF         AACGCTCCAT TTATCGATTC GGAAGG---- ---------- ---------- 126
SlMLO13_ROOT         AACGCTCCAT TTATCGATTC GGAAGG---- ---------- ---------- 126
SlMLO13_FLOWER       AACGCTCCAT TTATCGATTC GGAAGG---- ---------- ---------- 126
SlMLO13_FRUIT        AACGCTCCAT TTATCGATTC GGAAGG---- ---------- ---------- 126
                            160                           180                          200
gDNA_Solyc10g044510  TTTTATTTGT CAACACTAGT CTTTTCTTTT CTCAATGAGC TCTATCTACT 200
cDNASolyc10g044510   ---------- ---------- ---------- ---------- ---------- 126
SlMLO13_LEAF         ---------- ---------- ---------- ---------- ---------- 126
SlMLO13_ROOT         ---------- ---------- ---------- ---------- ---------- 126
SlMLO13_FLOWER       ---------- ---------- ---------- ---------- ---------- 126
SlMLO13_FRUIT        ---------- ---------- ---------- ---------- ---------- 126
                                       220                           240
gDNA_Solyc10g044510  TGGCTTTGTG CAGTGGTTGA AAAAGACTAG AAGAAAAGCT CTTTTTGCTT 250
cDNASolyc10g044510   ---------- ---TGGTTGA AAAAGACTAG AAGAAAAGCT CTTTTTGCTT 163
SlMLO13_LEAF         ---------- ---TGGTTGA AAAAGACTAG AAGAAAAGCT CTTTTTGCTT 163
SlMLO13_ROOT         ---------- ---TGGTTGA AAAAGACTAG AAGAAAAGCT CTTTTTGCTT 163
SlMLO13_FLOWER       ---------- ---TGGTTGA AAAAGACTAG AAGAAAAGCT CTTTTTGCTT 163
SlMLO13_FRUIT        ---------- ---TGGTTGA AAAAGACTAG AAGAAAAGCT CTTTTTGCTT 163
                            260                           280                          300
gDNA_Solyc10g044510  CTGTGGAGAA GATAAAGGAA GGTGACCAAT CATTCATGAA CTAGTTAACA 300
cDNASolyc10g044510   CTGTGGAGAA GATAAAGGAA G--------- ---------- ---------- 184
SlMLO13_LEAF         CTGTGGAGAA GATAAAGGAA G--------- ---------- ---------- 184
SlMLO13_ROOT         CTGTGGAGAA GATAAAGGAA G--------- ---------- ---------- 184
SlMLO13_FLOWER       CTGTGGAGAA GATAAAGGAA G--------- ---------- ---------- 184
SlMLO13_FRUIT        CTGTGGAGAA GATAAAGGAA G--------- ---------- ---------- 184
                                       320                           340
gDNA_Solyc10g044510  ATCTAAAAAT ACAAAATCTG ATTTTTTTTC TTCTCAAAAC AGAGTTGATG 350
cDNASolyc10g044510   ---------- ---------- ---------- ---------- AG--TTGATG 192
SlMLO13_LEAF         ---------- ---------- ---------- ---------- AGAGTTGATG 194
SlMLO13_ROOT         ---------- ---------- ---------- ---------- AG--TTGATG 192
SlMLO13_FLOWER       ---------- ---------- ---------- ---------- AG--TTGATG 192
SlMLO13_FRUIT        ---------- ---------- ---------- ---------- AG--TTGATG 192
                            360                           380                          400
gDNA_Solyc10g044510  CTGCTTGGCC TAATTTCTTT GTTGTTGGGA CAATGTGCAA GACAGATTTC 400
cDNASolyc10g044510   CTGCTTGGCC TAATTTCTTT GTTGTTGGGA CAATGTGCAA GACAGATTTC 242
SlMLO13_LEAF         CTGCTTGGCC TAATTTCTTT GTTGTTGGGA CAATGTGCAA GACAGATTTC 244
SlMLO13_ROOT         CTGCTTGGCC TAATTTCTTT GTTGTTGGGA CAATGTGCAA GACAGATTTC 242
SlMLO13_FLOWER       CTGCTTGGCC TAATTTCTTT GTTGTTGGGA CAATGTGCAA GACAGATTTC 242
SlMLO13_FRUIT        CTGCTTGGCC TAATTTCTTT GTTGTTGGGA CAATGTGCAA GACAGATTTC 242
                                       420                           440
gDNA_Solyc10g044510  TCAAATTTGT GTTAATTCAT CCCTTTTCAC TAGTAGATTC TACCTTTGTT 450
cDNASolyc10g044510   TCAAATTTGT GTTAATTCAT CCCTTTTCAC TAGTAGATTC TACCTTTGTT 292
SlMLO13_LEAF         TCAAATTTGT GTTAATTCAT CCCTTTTCAC TAGTAGATTC TACCTTTGTT 294
SlMLO13_ROOT         TCAAATTTGT GTTAATTCAT CCCTTTTCAC TAGTAGATTC TACCTTTGTT 292
SlMLO13_FLOWER       TCAAATTTGT GTTAATTCAT CCCTTTTCAC TAGTAGATTC TACCTTTGTT 292
SlMLO13_FRUIT        TCAAATTTGT GTTAATTCAT CCCTTTTCAC TAGTAGATTC TACCTTTGTT 292
                            460                           480                          500
gDNA_Solyc10g044510  CTCAAGAAGA TTATGACAGT AGTAGTAGTA GTACTAGTGA CACAATCCAT 500
cDNASolyc10g044510   CTCAAGAAGA TTATGACAGT AGTAGTAGTA GTACTAGTGA CACAATCCAT 342
SlMLO13_LEAF         CTCAAGAAGA TTATGACAGT AGTAGTAGTA GTACTAGTGA CACAATCCAT 344
SlMLO13_ROOT         CTCAAGAAGA TTATGACAGT AGTAGTAGTA GTACTAGTGA CACAATCCAT 342
SlMLO13_FLOWER       CTCAAGAAGA TTATGACAGT AGTAGTAGTA GTACTAGTGA CACAATCCAT 342
SlMLO13_FRUIT        CTCAAGAAGA TTATGACAGT AGTAGTAGTA GTACTAGTGA CACAATCCAT 342
                                       520                           540
gDNA_Solyc10g044510  TCCTCCATTC TCTCTAATCA TACTACTCTT ATTCCTCCCA AGGGAATTTA 550
cDNASolyc10g044510   TCCTCCATTC TCTCTAATCA TACTACTCTT ATTCCTCCCA AGGGAATTTA 392
SlMLO13_LEAF         TCCTCCATTC TCTCTAATCA TACTACTCTT ATTCCTCCCA AGGGAATTTA 394
SlMLO13_ROOT         TCCTCCATTC TCTCTAATCA TACTACTCTT ATTCCTCCCA AGGGAATTTA 392
SlMLO13_FLOWER       TCCTCCATTC TCTCTAATCA TACTACTCTT ATTCCTCCCA AGGGAATTTA 392
SlMLO13_FRUIT        TCCTCCATTC TCTCTAATCA TACTACTCTT ATTCCTCCCA AGGGAATTTA 392
                            560                           580                          600
gDNA_Solyc10g044510  TCAACAATCG CATCAATGTG GTGAGGTAAA TTGTTTTCTT CATTCATCTA 600
cDNASolyc10g044510   TCAACAATCG CATCAATGTG GTGAGG---- ---------- ---------- 418
SlMLO13_LEAF         TCAACAATCG CATCAATGTG GTGAGG---- ---------- ---------- 420
SlMLO13_ROOT         TCAACAATCG CATCAATGTG GTGAGG---- ---------- ---------- 418
SlMLO13_FLOWER       TCAACAATCG CATCAATGTG GTGAGG---- ---------- ---------- 418
SlMLO13_FRUIT        TCAACAATCG CATCAATGTG GTGAGG---- ---------- ---------- 418
```

```
                                                  620                           640
gDNA_Solyc10g044510  AATTTGAAAT TATAGTAAAT CATGTTTTCT ACTAATTAAC CACAAATCTT 650
cDNASolyc10g044510   ---------- ---------- ---------- ---------- ---------- 418
SlMLO13_LEAF         ---------- ---------- ---------- ---------- ---------- 420
SlMLO13_ROOT         ---------- ---------- ---------- ---------- ---------- 418
SlMLO13_FLOWER       ---------- ---------- ---------- ---------- ---------- 418
SlMLO13_FRUIT        ---------- ---------- ---------- ---------- ---------- 418
                                660                           680                700
gDNA_Solyc10g044510  TAACAGGGAC GTGAACCTTT TGTTTCCTAT GAAGGTCTTG AACAGCTTCA 700
cDNASolyc10g044510   -------GAC GTGAACCTTT TGTTTCCTAT GAAGGTCTTG AACAGCTTCA 461
SlMLO13_LEAF         ----AGGGAC GTGAACCTTT TGTTTCCTAT GAAGGTCTTG AACAGCTTCA 466
SlMLO13_ROOT         -------GAC GTGAACCTTT TGTTTCCTAT GAAGGTCTTG AACAGCTTCA 461
SlMLO13_FLOWER       -------GAC GTGAACCTTT TGTTTCCTAT GAAGGTCTTG AACAGCTTCA 461
SlMLO13_FRUIT        -------GAC GTGAACCTTT TGTTTCCTAT GAAGGTCTTG AACAGCTTCA 461
                                                  720                           740
gDNA_Solyc10g044510  CCGCTTCCTT TTTGTTCTTG GTATCACTCA TGTTCTCTAC AGTTGTATTG 750
cDNASolyc10g044510   CCGCTTCCTT TTTGTTCTTG GTATCACTCA TGTTCTCTAC AGTTGTATTG 511
SlMLO13_LEAF         CCGCTTCCTT TTTGTTCTTG GTATCACTCA TGTTCTCTAC AGTTGTATTG 516
SlMLO13_ROOT         CCGCTTCCTT TTTGTTCTTG GTATCACTCA TGTTCTCTAC AGTTGTATTG 511
SlMLO13_FLOWER       CCGCTTCCTT TTTGTTCTTG GTATCACTCA TGTTCTCTAC AGTTGTATTG 511
SlMLO13_FRUIT        CCGCTTCCTT TTTGTTCTTG GTATCGCCCA TGTTCTCTAC AGTTGTATTG 511
                                760                           780                800
gDNA_Solyc10g044510  TCGTTGGCTT GGCCATGACC AAGGTACACT TTCCATATCA AAAATATTTA 800
cDNASolyc10g044510   TCGTTGGCTT GGCCATGACC AAG------- ---------- ---------- 534
SlMLO13_LEAF         TCGTTGGCTT GGCCATGACC AAG------- ---------- ---------- 539
SlMLO13_ROOT         TCGTTGGCTT GGCCATGACC AAG------- ---------- ---------- 534
SlMLO13_FLOWER       TCGTTGGCTT GGCCATGACC AAG------- ---------- ---------- 534
SlMLO13_FRUIT        TCGTTGGCTT GGCCATGACC AAG------- ---------- ---------- 534
                                                  820                           840
gDNA_Solyc10g044510  AATCATGTGC CCTAGTCTAT TTTATGCTAG TGCTTGTATG TGAGTTTAAT 850
cDNASolyc10g044510   ---------- ---------- ---------- ---------- ---------- 534
SlMLO13_LEAF         ---------- ---------- ---------- ---------- ---------- 539
SlMLO13_ROOT         ---------- ---------- ---------- ---------- ---------- 534
SlMLO13_FLOWER       ---------- ---------- ---------- ---------- ---------- 534
SlMLO13_FRUIT        ---------- ---------- ---------- ---------- ---------- 534
                                860                           880                900
gDNA_Solyc10g044510  TTTACGGAGG TTGACTAGAC ACTAGCTACA CTCATGTCAA GATGTTCAAT 900
cDNASolyc10g044510   ---------- ---------- ---------- ---------- ---------- 534
SlMLO13_LEAF         ---------- ---------- ---------- ---------- ---------- 539
SlMLO13_ROOT         ---------- ---------- ---------- ---------- ---------- 534
SlMLO13_FLOWER       ---------- ---------- ---------- ---------- ---------- 534
SlMLO13_FRUIT        ---------- ---------- ---------- ---------- ---------- 534
                                                  920                           940
gDNA_Solyc10g044510  TGGACACCTT TTGTCAAAAA ATTATATTGC ATATTCAAAT TTAATAATTA 950
cDNASolyc10g044510   ---------- ---------- ---------- ---------- ---------- 534
SlMLO13_LEAF         ---------- ---------- ---------- ---------- ---------- 539
SlMLO13_ROOT         ---------- ---------- ---------- ---------- ---------- 534
SlMLO13_FLOWER       ---------- ---------- ---------- ---------- ---------- 534
SlMLO13_FRUIT        ---------- ---------- ---------- ---------- ---------- 534
                                960                           980              1,000
gDNA_Solyc10g044510  ACAAAGTGTT CGTATGTTCA AATCTCACTA GTAGCAACTC TTTTTTATCA 1000
cDNASolyc10g044510   ---------- ---------- ---------- ---------- ---------- 534
SlMLO13_LEAF         ---------- ---------- ---------- ---------- ---------- 539
SlMLO13_ROOT         ---------- ---------- ---------- ---------- ---------- 534
SlMLO13_FLOWER       ---------- ---------- ---------- ---------- ---------- 534
SlMLO13_FRUIT        ---------- ---------- ---------- ---------- ---------- 534
                                                1,020                         1,040
gDNA_Solyc10g044510  CTTTTTAAGA AGAACAGACT CACTTCAGCA CAGCACCTGG ACACTCTTCG 1050
cDNASolyc10g044510   ---------- ---------- ---------- ---------- ---------- 534
SlMLO13_LEAF         ---------- ---------- ---------- ---------- ---------- 539
SlMLO13_ROOT         ---------- ---------- ---------- ---------- ---------- 534
SlMLO13_FLOWER       ---------- ---------- ---------- ---------- ---------- 534
SlMLO13_FRUIT        ---------- ---------- ---------- ---------- ---------- 534
                              1,060                         1,080              1,100
gDNA_Solyc10g044510  TGAAATTTCT CGCGCCACTA CATGTCGATG TTCTAAGGAT TTACTTACTA 1100
cDNASolyc10g044510   ---------- ---------- ---------- ---------- ---------- 534
SlMLO13_LEAF         ---------- ---------- ---------- ---------- ---------- 539
SlMLO13_ROOT         ---------- ---------- ---------- ---------- ---------- 534
SlMLO13_FLOWER       ---------- ---------- ---------- ---------- ---------- 534
SlMLO13_FRUIT        ---------- ---------- ---------- ---------- ---------- 534
                                                1,120                         1,140
gDNA_Solyc10g044510  TATCAGATTG TAGTATTCCT TGTTTGTGGT TTTAAATGAA TTTGATTGTA 1150
cDNASolyc10g044510   ---------- ---------- ---------- ---------- ---------A 535
SlMLO13_LEAF         ---------- ---------- ---------- ---------- ---------A 540
SlMLO13_ROOT         ---------- ---------- ---------- ---------- ---------A 535
SlMLO13_FLOWER       ---------- ---------- ---------- ---------- ---------A 535
SlMLO13_FRUIT        ---------- ---------- ---------- ---------- ---------A 535
                              1,160                         1,180              1,200
gDNA_Solyc10g044510  GATATACAGT TGGAGGAAGT GGGAAAACCA AGTGAGCTCT GGGGAACAGA 1200
cDNASolyc10g044510   --TATACAGT TGGAGGAAGT GGGAAAACCA AGTGAGCTCT GGGGAACAGA 583
SlMLO13_LEAF         GATATACAGT TGGAGGAAGT GGGAAAACCA AGTGAGCTCT GGGGAACAGA 590
SlMLO13_ROOT         --TATACAGT TGGAGGAAGT GGGAAAACCA AGTGAGCTCT GGGGAACAGA 583
SlMLO13_FLOWER       --TATACAGT TGGAGGAAGT GGGAAAACCA AGTGAGCTCT GGGGAACAGA 583
SlMLO13_FRUIT        --TATACAGT TGGAGGAAGT GGGAAAACCA AGTGAGCTCT GGGGAACAGA 583
```

```
                              1,220               1,240
gDNA_Solyc10g044510  ACAACTTACA AGGTGTGTGG AATTAATAAT TATTAACTAT GTCCTATCAA 1250
cDNASolyc10g044510   ACAACTTACA AG-------- ---------- ---------- ---------- 595
SlMLO13_LEAF         ACAACTTACA AG-------- ---------- ---------- ---------- 602
SlMLO13_ROOT         ACAACTTACA AG-------- ---------- ---------- ---------- 595
SlMLO13_FLOWER       ACAACTTACA AG-------- ---------- ---------- ---------- 595
SlMLO13_FRUIT        ACAACTTACA AG-------- ---------- ---------- ---------- 595

                     1,260               1,280               1,300
gDNA_Solyc10g044510  TATAAATTCA CCTTCTTCCA TATAAGTAAT AGTTGCATAA AATACAAATT 1300
cDNASolyc10g044510   ---------- ---------- ---------- ---------- ---------- 595
SlMLO13_LEAF         ---------- ---------- ---------- ---------- ---------- 602
SlMLO13_ROOT         ---------- ---------- ---------- ---------- ---------- 595
SlMLO13_FLOWER       ---------- ---------- ---------- ---------- ---------- 595
SlMLO13_FRUIT        ---------- ---------- ---------- ---------- ---------- 595

                              1,320               1,340
gDNA_Solyc10g044510  TAAGGAAACG TATCATAGAT TATTCAACAA GTATGTAATA TTTTCTTAAA 1350
cDNASolyc10g044510   ---------- ---------- ---------- ---------- ---------- 595
SlMLO13_LEAF         ---------- ---------- ---------- ---------- ---------- 602
SlMLO13_ROOT         ---------- ---------- ---------- ---------- ---------- 595
SlMLO13_FLOWER       ---------- ---------- ---------- ---------- ---------- 595
SlMLO13_FRUIT        ---------- ---------- ---------- ---------- ---------- 595

                     1,360               1,380               1,400
gDNA_Solyc10g044510  GATTAAAAAA TGTGTTCTCA TCTTTTATCA TAGAGCTGAG CTTCTAAATA 1400
cDNASolyc10g044510   ---------- ---------- ---------- ---------- ---------- 595
SlMLO13_LEAF         ---------- ---------- ---------- ---------- ---------- 602
SlMLO13_ROOT         ---------- ---------- ---------- ---------- ---------- 595
SlMLO13_FLOWER       ---------- ---------- ---------- ---------- ---------- 595
SlMLO13_FRUIT        ---------- ---------- ---------- ---------- ---------- 595

                              1,420               1,440
gDNA_Solyc10g044510  TCCCTTTCTA TTGTTTTATT TAGTGCCAAA GAATAAGGAG ATGAGACGAC 1450
cDNASolyc10g044510   ---------- ---------- ---TGCCAAA GAATAAGGAG ATGAGACGAC 622
SlMLO13_LEAF         ---------- ---------- ---AGC---- ---------- ---------- 605
SlMLO13_ROOT         ---------- ---------- ---TGCCAAA GAATAAGGAG ATGAGACGAC 622
SlMLO13_FLOWER       ---------- ---------- ---TGCCAAA GAATAAGGAG ATGAGACGAC 622
SlMLO13_FRUIT        ---------- ---------- ---TGCCAAA GAATAAGGAG ATGAGACGAC 622

                     1,460               1,480               1,500
gDNA_Solyc10g044510  AGTCCACCTT TGCCCTGCAT CATGCTTCCC ATCCATGGAG TAGGAGTCGG 1500
cDNASolyc10g044510   AGTCCACCTT TGCCCTGCAT CATGCTTCCC ATCCATGGAG TAGGAGTCGG 672
SlMLO13_LEAF         ---------- ---------- ---------- ---------- ---------- 605
SlMLO13_ROOT         AGTCCACCTT TGCCCTGCAT CATGCTTCCC ATCCATGGAG TAGGAGTCGG 672
SlMLO13_FLOWER       AGTCCACCTT TGCCCTGCAT CATGCTTCCC ATCCATGGAG TAGGAGTCGG 672
SlMLO13_FRUIT        AGTCCACCTT TGCCCTGCAT CATGCTTCCC ATCCATGGAG TAGGAGTCGG 672

                              1,520               1,540
gDNA_Solyc10g044510  ATTCTTATAT GGATGGTAAT AGTTATTGCC TTAATCTAAC AACATCATAC 1550
cDNASolyc10g044510   ATTCTTATAT GGATG----- ---------- ---------- ---------- 687
SlMLO13_LEAF         ---------- ---------- ---------- ---------- ---------- 605
SlMLO13_ROOT         ATTCTTATAT GGATG----- ---------- ---------- ---------- 687
SlMLO13_FLOWER       ATTCTTATAT GGATG----- ---------- ---------- ---------- 687
SlMLO13_FRUIT        ATTCTTATAT GGATG----- ---------- ---------- ---------- 687

                     1,560               1,580               1,600
gDNA_Solyc10g044510  ACTTCTCTAT AAATTAGGAT GCATTCAAGT AATTTTTATG TATCATGGAT 1600
cDNASolyc10g044510   ---------- ---------- ---------- ---------- ---------- 687
SlMLO13_LEAF         ---------- ---------- ---------- ---------- ---------- 605
SlMLO13_ROOT         ---------- ---------- ---------- ---------- ---------- 687
SlMLO13_FLOWER       ---------- ---------- ---------- ---------- ---------- 687
SlMLO13_FRUIT        ---------- ---------- ---------- ---------- ---------- 687

                              1,620               1,640
gDNA_Solyc10g044510  ATTTATTCCA TAGTCGTCTC CTGAGATTAT GTAATAGGAA AATTTTTCCA 1650
cDNASolyc10g044510   ---------- ---------- ---------- ---------- ---------- 687
SlMLO13_LEAF         ---------- ---------- ---------- ---------- ---------- 605
SlMLO13_ROOT         ---------- ---------- ---------- ---------- ---------- 687
SlMLO13_FLOWER       ---------- ---------- ---------- ---------- ---------- 687
SlMLO13_FRUIT        ---------- ---------- ---------- ---------- ---------- 687

                     1,660               1,680               1,700
gDNA_Solyc10g044510  TCTAACTTGG TGTTAAAAAC CAAACACTTC TTTAAAATTT TGGACTTTAG 1700
cDNASolyc10g044510   ---------- ---------- ---------- ---------- ---------- 687
SlMLO13_LEAF         ---------- ---------- ---------- ---------- ---------- 605
SlMLO13_ROOT         ---------- ---------- ---------- ---------- ---------- 687
SlMLO13_FLOWER       ---------- ---------- ---------- ---------- ---------- 687
SlMLO13_FRUIT        ---------- ---------- ---------- ---------- ---------- 687

                              1,720               1,740
gDNA_Solyc10g044510  ACCTTTGTGG AAGAACTAGT ACCGATTACT CCTACAAGGA ACCAACTTCC 1750
cDNASolyc10g044510   ---------- ---------- ---------- ---------- ---------- 687
SlMLO13_LEAF         ---------- ---------- ---------- ---------- ---------- 605
SlMLO13_ROOT         ---------- ---------- ---------- ---------- ---------- 687
SlMLO13_FLOWER       ---------- ---------- ---------- ---------- ---------- 687
SlMLO13_FRUIT        ---------- ---------- ---------- ---------- ---------- 687

                     1,760               1,780               1,800
gDNA_Solyc10g044510  TTTAGACGGA ACAAGAAAGT AGTATAGGAA CACAAGTTTT ACCATGGAAA 1800
cDNASolyc10g044510   ---------- ---------- ---------- ---------- ---------- 687
SlMLO13_LEAF         ---------- ---------- ---------- ---------- ---------- 605
SlMLO13_ROOT         ---------- ---------- ---------- ---------- ---------- 687
SlMLO13_FLOWER       ---------- ---------- ---------- ---------- ---------- 687
SlMLO13_FRUIT        ---------- ---------- ---------- ---------- ---------- 687
```

```
                                        1,820                 1,840
gDNA_Solyc10g044510  ACCTCCTTGC TCAAGGAGTA AAAAGCTACA GTGAGTAGGA TTTTAAGAAC 1850
cDNASolyc10g044510   ---------- ---------- ---------- ---------- ---------- 687
SlMLO13_LEAF         ---------- ---------- ---------- ---------- ---------- 605
SlMLO13_ROOT         ---------- ---------- ---------- ---------- ---------- 687
SlMLO13_FLOWER       ---------- ---------- ---------- ---------- ---------- 687
SlMLO13_FRUIT        ---------- ---------- ---------- ---------- ---------- 687
                             1,860                 1,880                 1,900
gDNA_Solyc10g044510  CAATCTTCAC TAACTTCAAA GAATAGTGTC AGATTACATC TCTATAATCT 1900
cDNASolyc10g044510   ---------- ---------- ---------- ---------- ---------- 687
SlMLO13_LEAF         ---------- ---------- ---------- ---------- ---------- 605
SlMLO13_ROOT         ---------- ---------- ---------- ---------- ---------- 687
SlMLO13_FLOWER       ---------- ---------- ---------- ---------- ---------- 687
SlMLO13_FRUIT        ---------- ---------- ---------- ---------- ---------- 687
                                        1,920                 1,940
gDNA_Solyc10g044510  AAGGTACTAA ATCTAGTATC TGTACTCAAC AGTTAACTCT AACTGCTCTA 1950
cDNASolyc10g044510   ---------- ---------- ---------- ---------- ---------- 687
SlMLO13_LEAF         ---------- ---------- ---------- ---------- ---------- 605
SlMLO13_ROOT         ---------- ---------- ---------- ---------- ---------- 687
SlMLO13_FLOWER       ---------- ---------- ---------- ---------- ---------- 687
SlMLO13_FRUIT        ---------- ---------- ---------- ---------- ---------- 687
                             1,960                 1,980                 2,000
gDNA_Solyc10g044510  GTCTCTTCTT GTTACAAACC TCACAACTAT CACAGATAAA CCAAACAAGA 2000
cDNASolyc10g044510   ---------- ---------- ---------- ---------- ---------- 687
SlMLO13_LEAF         ---------- ---------- ---------- ---------- ---------- 605
SlMLO13_ROOT         ---------- ---------- ---------- ---------- ---------- 687
SlMLO13_FLOWER       ---------- ---------- ---------- ---------- ---------- 687
SlMLO13_FRUIT        ---------- ---------- ---------- ---------- ---------- 687
                                        2,020                 2,040
gDNA_Solyc10g044510  AATTATTACA ACTCAATAAT ATTCATGAAA CAACTTCAAC TAAGAAAGCA 2050
cDNASolyc10g044510   ---------- ---------- ---------- ---------- ---------- 687
SlMLO13_LEAF         ---------- ---------- ---------- ---------- ---------- 605
SlMLO13_ROOT         ---------- ---------- ---------- ---------- ---------- 687
SlMLO13_FLOWER       ---------- ---------- ---------- ---------- ---------- 687
SlMLO13_FRUIT        ---------- ---------- ---------- ---------- ---------- 687
                             2,060                 2,080                 2,100
gDNA_Solyc10g044510  TGAACACACC CTAAACACTA TTCTTCTTTC TTCCTTGTTC CTCTGCTTGG 2100
cDNASolyc10g044510   ---------- ---------- ---------- ---------- ---------- 687
SlMLO13_LEAF         ---------- ---------- ---------- ---------- ---------- 605
SlMLO13_ROOT         ---------- ---------- ---------- ---------- ---------- 687
SlMLO13_FLOWER       ---------- ---------- ---------- ---------- ---------- 687
SlMLO13_FRUIT        ---------- ---------- ---------- ---------- ---------- 687
                                        2,120                 2,140
gDNA_Solyc10g044510  ATGCAATGTA ATAGACTTTG TTGGAATACA TTTTCTGCTT GTGCAATAGC 2150
cDNASolyc10g044510   ---------- ---------- ---------- ---------- ---------- 687
SlMLO13_LEAF         ---------- ---------- ---------- ---------- ---------- 605
SlMLO13_ROOT         ---------- ---------- ---------- ---------- ---------- 687
SlMLO13_FLOWER       ---------- ---------- ---------- ---------- ---------- 687
SlMLO13_FRUIT        ---------- ---------- ---------- ---------- ---------- 687
                             2,160                 2,180                 2,200
gDNA_Solyc10g044510  TGTCTTCGAT GAGCCTAAAA TTTTGCCTTA TATAGTTAGG ACACCTTGTG 2200
cDNASolyc10g044510   ---------- ---------- ---------- ---------- ---------- 687
SlMLO13_LEAF         ---------- ---------- ---------- ---------- ---------- 605
SlMLO13_ROOT         ---------- ---------- ---------- ---------- ---------- 687
SlMLO13_FLOWER       ---------- ---------- ---------- ---------- ---------- 687
SlMLO13_FRUIT        ---------- ---------- ---------- ---------- ---------- 687
                                        2,220                 2,240
gDNA_Solyc10g044510  CTGTGCAACA ACCCTGATTG ACTCAGTGCA AAACTATGGT TACACAATTC 2250
cDNASolyc10g044510   ---------- ---------- ---------- ---------- ---------- 687
SlMLO13_LEAF         ---------- ---------- ---------- ---------- ---------- 605
SlMLO13_ROOT         ---------- ---------- ---------- ---------- ---------- 687
SlMLO13_FLOWER       ---------- ---------- ---------- ---------- ---------- 687
SlMLO13_FRUIT        ---------- ---------- ---------- ---------- ---------- 687
                             2,260                 2,280                 2,300
gDNA_Solyc10g044510  AATAATATTT TTCTCCTTTA TTTCTCACAG TTCTTAACTA GAGGCAAAAT 2300
cDNASolyc10g044510   ---------- ---------- ---------- ---------- ---------- 687
SlMLO13_LEAF         ---------- ---------- ---------- ---------- ---------- 605
SlMLO13_ROOT         ---------- ---------- ---------- ---------- ---------- 687
SlMLO13_FLOWER       ---------- ---------- ---------- ---------- ---------- 687
SlMLO13_FRUIT        ---------- ---------- ---------- ---------- ---------- 687
                                        2,320                 2,340
gDNA_Solyc10g044510  GTATGATTAT GTATAGGTTA TCTATGTGTC TTTTACTGAA CGGACTGAAA 2350
cDNASolyc10g044510   ---------- ---------- ---------- ---------- ---------- 687
SlMLO13_LEAF         ---------- ---------- ---------- ---------- ---------- 605
SlMLO13_ROOT         ---------- ---------- ---------- ---------- ---------- 687
SlMLO13_FLOWER       ---------- ---------- ---------- ---------- ---------- 687
SlMLO13_FRUIT        ---------- ---------- ---------- ---------- ---------- 687
                             2,360                 2,380                 2,400
gDNA_Solyc10g044510  TGTTCTATAT CTTCTGTCTT TTTTAAATTG ATAAATTCAA ACTATTAGGG 2400
cDNASolyc10g044510   ---------- ---------- ---------- ---------- ---------- 687
SlMLO13_LEAF         ---------- ---------- ---------- ---------- ---------- 605
SlMLO13_ROOT         ---------- ---------- ---------- ---------- ---------- 687
SlMLO13_FLOWER       ---------- ---------- ---------- ---------- ---------- 687
SlMLO13_FRUIT        ---------- ---------- ---------- ---------- ---------- 687
```

```
                                               2,420                                       2,440
gDNA_Solyc10g044510  GTTGTGTGGT AGAGTAATGT AAATACTCTA GAATTAAAAA TCTAAATAGA 2450
cDNASolyc10g044510   ---------- ---------- ---------- ---------- ---------- 687
SlMLO13_LEAF         ---------- ---------- ---------- ---------- ---------- 605
SlMLO13_ROOT         ---------- ---------- ---------- ---------- ---------- 687
SlMLO13_FLOWER       ---------- ---------- ---------- ---------- ---------- 687
SlMLO13_FRUIT        ---------- ---------- ---------- ---------- ---------- 687
                               2,460                  2,480                  2,500
gDNA_Solyc10g044510  AATTAAACAT TTGTGTAGAA ATTAGTTGTT CCATTTATTT AATCTGAGTT 2500
cDNASolyc10g044510   ---------- ---------- ---------- ---------- ---------- 687
SlMLO13_LEAF         ---------- ---------- ---------- ---------- ---------- 605
SlMLO13_ROOT         ---------- ---------- ---------- ---------- ---------- 687
SlMLO13_FLOWER       ---------- ---------- ---------- ---------- ---------- 687
SlMLO13_FRUIT        ---------- ---------- ---------- ---------- ---------- 687
                                          2,520                  2,540
gDNA_Solyc10g044510  GCCAAGTTCT AAGTGAATGT ATCTTTTGTT GTAACATAAT CAATAACATG 2550
cDNASolyc10g044510   ---------- ---------- ---------- ---------- ---------- 687
SlMLO13_LEAF         ---------- ---------- ---------- ---------- ---------- 605
SlMLO13_ROOT         ---------- ---------- ---------- ---------- ---------- 687
SlMLO13_FLOWER       ---------- ---------- ---------- ---------- ---------- 687
SlMLO13_FRUIT        ---------- ---------- ---------- ---------- ---------- 687
                               2,560                  2,580                  2,600
gDNA_Solyc10g044510  CATAGCAAAT CGTAGCACCT TTGACCACAC TTTTCAGCTT TCCTTGCAAA 2600
cDNASolyc10g044510   ---------- ---------- ---------- ---------- ---------- 687
SlMLO13_LEAF         ---------- ---------- ---------- ---------- ---------- 605
SlMLO13_ROOT         ---------- ---------- ---------- ---------- ---------- 687
SlMLO13_FLOWER       ---------- ---------- ---------- ---------- ---------- 687
SlMLO13_FRUIT        ---------- ---------- ---------- ---------- ---------- 687
                                          2,620                  2,640
gDNA_Solyc10g044510  TATGACGAAA TTCCTATACA TTATTGTCTT GAATTTTATC TCAGCAATCA 2650
cDNASolyc10g044510   ---------- ---------- ---------- ---------- ---------- 687
SlMLO13_LEAF         ---------- ---------- ---------- ---------- ---------- 605
SlMLO13_ROOT         ---------- ---------- ---------- ---------- ---------- 687
SlMLO13_FLOWER       ---------- ---------- ---------- ---------- ---------- 687
SlMLO13_FRUIT        ---------- ---------- ---------- ---------- ---------- 687
                               2,660                  2,680                  2,700
gDNA_Solyc10g044510  TGTGTTTCTG CATTATCCAT GCATGGATAA AATCCGTGAC ATAGATTTGT 2700
cDNASolyc10g044510   ---------- ---------- ---------- ---------- ---------- 687
SlMLO13_LEAF         ---------- ---------- ---------- ---------- ---------- 605
SlMLO13_ROOT         ---------- ---------- ---------- ---------- ---------- 687
SlMLO13_FLOWER       ---------- ---------- ---------- ---------- ---------- 687
SlMLO13_FRUIT        ---------- ---------- ---------- ---------- ---------- 687
                                          2,720                  2,740
gDNA_Solyc10g044510  TTTGTAATCT TGCAGCTTTG CTTTCTTAGG CAGTTCAGGA CTTCAATTCA 2750
cDNASolyc10g044510   ---------- -----CTTTG CTTTCTTAGG CAGTTCAGGA CTTCAATTCA 722
SlMLO13_LEAF         ---------- ------TTTG CTTTCTTAGG CAGTTCAGGA CTTCAATTCA 639
SlMLO13_ROOT         ---------- -----CTTTG CTTTCTTAGG CAGTTCAGGA CTTCAATTCA 722
SlMLO13_FLOWER       ---------- -----CTTTG CTTTCTTAGG CAGTTCAGGA CTTCAATTCA 722
SlMLO13_FRUIT        ---------- -----CTTTG CTTTCTTAGG CAGTTCAGGA CTTCAATTCA 722
                               2,760                  2,780                  2,800
gDNA_Solyc10g044510  CAAGTCAGAT TACTTGGCAC TGAGATTGGG GTTTATCACT GTAGGTATTC 2800
cDNASolyc10g044510   CAAGTCAGAT TACTTGGCAC TGAGATTGGG GTTTATCACT ---------- 762
SlMLO13_LEAF         CAAGTCAGAT TACTTGGCAC TGAGATTGGG GTTTATCACT ---------- 679
SlMLO13_ROOT         CAAGTCAGAT TACTTGGCAC TGAGATTGGG GTTTATCACT ---------- 762
SlMLO13_FLOWER       CAAGTCAGAT TACTTGGCAC TGAGATTGGG GTTTATCACT ---------- 762
SlMLO13_FRUIT        CAAGTCAGAT TACTTGGCAC TGAGATTGGG GTTTATCACT ---------- 762
                                          2,820                  2,840
gDNA_Solyc10g044510  AGTTTCTTTT GGAACTTCAT TTATATTGTT CCACTTAGAA ATGATGTCTC 2850
cDNASolyc10g044510   ---------- ---------- ---------- ---------- ---------- 762
SlMLO13_LEAF         ---------- ---------- ---------- ---------- ---------- 679
SlMLO13_ROOT         ---------- ---------- ---------- ---------- ---------- 762
SlMLO13_FLOWER       ---------- ---------- ---------- ---------- ---------- 762
SlMLO13_FRUIT        ---------- ---------- ---------- ---------- ---------- 762
                               2,860                  2,880                  2,900
gDNA_Solyc10g044510  AACTCCTCTG TTAGTGTGGG AAAGTAAGGG AAGCGGTGGA AATGCTCAGT 2900
cDNASolyc10g044510   ---------- ---------- ---------- ---------- ---------- 762
SlMLO13_LEAF         ---------- ---------- ---------- ---------- ---------- 679
SlMLO13_ROOT         ---------- ---------- ---------- ---------- ---------- 762
SlMLO13_FLOWER       ---------- ---------- ---------- ---------- ---------- 762
SlMLO13_FRUIT        ---------- ---------- ---------- ---------- ---------- 762
                                          2,920                  2,940
gDNA_Solyc10g044510  ATTCTTTTCC ACGGTTAAAA TTCGTATGCA TCATTCTCGA CATGCTTAAG 2950
cDNASolyc10g044510   ---------- ---------- ---------- ---------- ---------- 762
SlMLO13_LEAF         ---------- ---------- ---------- ---------- ---------- 679
SlMLO13_ROOT         ---------- ---------- ---------- ---------- ---------- 762
SlMLO13_FLOWER       ---------- ---------- ---------- ---------- ---------- 762
SlMLO13_FRUIT        ---------- ---------- ---------- ---------- ---------- 762
                               2,960                  2,980                  3,000
gDNA_Solyc10g044510  CAGTTGCATA TTTAATTGAA AGGTGTCCTA TGAGGAATAC ATTATACAAG 3000
cDNASolyc10g044510   ---------- ---------- ---------- ---------- ---------- 762
SlMLO13_LEAF         ---------- ---------- ---------- ---------- ---------- 679
SlMLO13_ROOT         ---------- ---------- ---------- ---------- ---------- 762
SlMLO13_FLOWER       ---------- ---------- ---------- ---------- ---------- 762
SlMLO13_FRUIT        ---------- ---------- ---------- ---------- ---------- 762
```

```
                                              3,020                         3,040
gDNA_Solyc10g044510  AGGGAAGGAT ATTTTAAAGA TGTTGCTGTT AAGTTATAAT ATATATCTGA 3050
 cDNASolyc10g044510  ---------- ---------- ---------- ---------- ---------- 762
       SlMLO13_LEAF  ---------- ---------- ---------- ---------- ---------- 679
       SlMLO13_ROOT  ---------- ---------- ---------- ---------- ---------- 762
     SlMLO13_FLOWER  ---------- ---------- ---------- ---------- ---------- 762
      SlMLO13_FRUIT  ---------- ---------- ---------- ---------- ---------- 762
                               3,060                         3,080                         3,100
gDNA_Solyc10g044510  ATGTAATTGC TGAAGCACTT AAGTTGAATG TTTTAATTCT GTTTAATTTG 3100
 cDNASolyc10g044510  ---------- ---------- ---------- ---------- ---------- 762
       SlMLO13_LEAF  ---------- ---------- ---------- ---------- ---------- 679
       SlMLO13_ROOT  ---------- ---------- ---------- ---------- ---------- 762
     SlMLO13_FLOWER  ---------- ---------- ---------- ---------- ---------- 762
      SlMLO13_FRUIT  ---------- ---------- ---------- ---------- ---------- 762
                                              3,120                         3,140
gDNA_Solyc10g044510  TTCCCGAAAT TCAGTATCCT TGTAATAATT TTGTTGCTCG GAGGTATTTA 3150
 cDNASolyc10g044510  ---------- ---------- ---------- ---------- ---------- 762
       SlMLO13_LEAF  ---------- ---------- ---------- ---------- ---------- 679
       SlMLO13_ROOT  ---------- ---------- ---------- ---------- ---------- 762
     SlMLO13_FLOWER  ---------- ---------- ---------- ---------- ---------- 762
      SlMLO13_FRUIT  ---------- ---------- ---------- ---------- ---------- 762
                               3,160                         3,180                         3,200
gDNA_Solyc10g044510  TAGAGTAGGG TGTTTTATTT TGTTCGTCAT ACTAAGTACT CCATTGCAAG 3200
 cDNASolyc10g044510  ---------- ---------- ---------- ---------- ---------- 762
       SlMLO13_LEAF  ---------- ---------- ---------- ---------- ---------- 679
       SlMLO13_ROOT  ---------- ---------- ---------- ---------- ---------- 762
     SlMLO13_FLOWER  ---------- ---------- ---------- ---------- ---------- 762
      SlMLO13_FRUIT  ---------- ---------- ---------- ---------- ---------- 762
                                              3,220                         3,240
gDNA_Solyc10g044510  TAATGCCAGG GATAAGACTT AAGAGGACAT ATTTGTAGCT AATTTATTAC 3250
 cDNASolyc10g044510  ---------- ---------- ---------- ---------- ---------- 762
       SlMLO13_LEAF  ---------- ---------- ---------- ---------- ---------- 679
       SlMLO13_ROOT  ---------- ---------- ---------- ---------- ---------- 762
     SlMLO13_FLOWER  ---------- ---------- ---------- ---------- ---------- 762
      SlMLO13_FRUIT  ---------- ---------- ---------- ---------- ---------- 762
                               3,260                         3,280                         3,300
gDNA_Solyc10g044510  TTGAACCAAA TCCAGAAGCA GTAACTGTTG TTCCCAGAAT TCACTTATTT 3300
 cDNASolyc10g044510  ---------- ---------- ---------- ---------- ---------- 762
       SlMLO13_LEAF  ---------- ---------- ---------- ---------- ---------- 679
       SlMLO13_ROOT  ---------- ---------- ---------- ---------- ---------- 762
     SlMLO13_FLOWER  ---------- ---------- ---------- ---------- ---------- 762
      SlMLO13_FRUIT  ---------- ---------- ---------- ---------- ---------- 762
                                              3,320                         3,340
gDNA_Solyc10g044510  ATTTTCATTG TTTGACCGTT GCTTCAATAA CATGCAGAAT CACAAGCTTC 3350
 cDNASolyc10g044510  ---------- ---------- ---------- -------AAT CACAAGCTTC 775
       SlMLO13_LEAF  ---------- ---------- ---------- -------AAT CACAAGCTTC 692
       SlMLO13_ROOT  ---------- ---------- ---------- -------AAT CACAAGCTTC 775
     SlMLO13_FLOWER  ---------- ---------- ---------- -------AAT CACAAGCTTC 775
      SlMLO13_FRUIT  ---------- ---------- ---------- -------AAT CACAAGCTTC 775
                               3,360                         3,380                         3,400
gDNA_Solyc10g044510  CACTTACTTA CAATTTCCAC AAGTACATGG TTCGTAGCAT GGAAGATGAG 3400
 cDNASolyc10g044510  CACTTACTTA CAATTTCCAC AAGTACATGG TTCGTAGCAT GGAAGATGAG 825
       SlMLO13_LEAF  CACTTACTTA CAATTTCCAC AAGTACATGG TTCGTAGCAT GGAAGATGAG 742
       SlMLO13_ROOT  CACTTACTTA CAATTTCCAC AAGTACATGG TTCGTAGCAT GGAAGATGAG 825
     SlMLO13_FLOWER  CACTTACTTA CAATTTCCAC AAGTACATGG TTCGTAGCAT GGAAGATGAG 825
      SlMLO13_FRUIT  CACTTACTTA CAATTTCCAC AAGTACATGG TTCGTAGCAT GGAAGATGAG 825
                                              3,420                         3,440
gDNA_Solyc10g044510  TTTTATGAAA TTGTAGGCAT CAGGTATGTT GTTATTGGTT TAGATCATTT 3450
 cDNASolyc10g044510  TTTTATGAAA TTGTAGGCAT CA-------- ---------- ---------- 847
       SlMLO13_LEAF  TTTTATGAAA TTGTAGGCAT CA-------- ---------- ---------- 764
       SlMLO13_ROOT  TTTTATGAAA TTGTAGGCAT CA-------- ---------- ---------- 847
     SlMLO13_FLOWER  TTTTATGAAA TTGTAGGCAT CA-------- ---------- ---------- 847
      SlMLO13_FRUIT  TTTTATGAAA TTGTAGGCAT CA-------- ---------- ---------- 847
                               3,460                         3,480                         3,500
gDNA_Solyc10g044510  TAATTCACAG TATAGGGGTG AGAATATTTT TGTTCAGTAG TAATTATACT 3500
 cDNASolyc10g044510  ---------- ---------- ---------- ---------- ---------- 847
       SlMLO13_LEAF  ---------- ---------- ---------- ---------- ---------- 764
       SlMLO13_ROOT  ---------- ---------- ---------- ---------- ---------- 847
     SlMLO13_FLOWER  ---------- ---------- ---------- ---------- ---------- 847
      SlMLO13_FRUIT  ---------- ---------- ---------- ---------- ---------- 847
                                              3,520                         3,540
gDNA_Solyc10g044510  TTCTGTTTGT GACATACCCA TCAATCAAAG ACTTGAGCTC CTCCTTGAGT 3550
 cDNASolyc10g044510  ---------- ---------- ---------- ---------- ---------- 847
       SlMLO13_LEAF  ---------- ---------- ---------- ---------- ---------- 764
       SlMLO13_ROOT  ---------- ---------- ---------- ---------- ---------- 847
     SlMLO13_FLOWER  ---------- ---------- ---------- ---------- ---------- 847
      SlMLO13_FRUIT  ---------- ---------- ---------- ---------- ---------- 847
                               3,560                         3,580                         3,600
gDNA_Solyc10g044510  TTTTATTGGT GTTTATGATG AATGCTTCTT CTAGGTGCTT CCGCATAAAT 3600
 cDNASolyc10g044510  ---------- ---------- ---------- ---------- ---------- 847
       SlMLO13_LEAF  ---------- ---------- ---------- ---------- ---------- 764
       SlMLO13_ROOT  ---------- ---------- ---------- ---------- ---------- 847
     SlMLO13_FLOWER  ---------- ---------- ---------- ---------- ---------- 847
      SlMLO13_FRUIT  ---------- ---------- ---------- ---------- ---------- 847
```

```
                                            3,620                           3,640
gDNA_Solyc10g044510  TTTGGTGAAA ATACGTGAAA AGTTTGGAAT ATGTTGGGTT GTTTTTTTCG 3650
cDNASolyc10g044510   ---------- ---------- ---------- ---------- ---------- 847
SlMLO13_LEAF         ---------- ---------- ---------- ---------- ---------- 764
SlMLO13_ROOT         ---------- ---------- ---------- ---------- ---------- 847
SlMLO13_FLOWER       ---------- ---------- ---------- ---------- ---------- 847
SlMLO13_FRUIT        ---------- ---------- ---------- ---------- ---------- 847
                                3,660                           3,680                      3,700
gDNA_Solyc10g044510  GCATCAAATA AAGCGTAGAG GAAAAAACTT ATTTTTATAG CTAATGAGCT 3700
cDNASolyc10g044510   ---------- ---------- ---------- ---------- ---------- 847
SlMLO13_LEAF         ---------- ---------- ---------- ---------- ---------- 764
SlMLO13_ROOT         ---------- ---------- ---------- ---------- ---------- 847
SlMLO13_FLOWER       ---------- ---------- ---------- ---------- ---------- 847
SlMLO13_FRUIT        ---------- ---------- ---------- ---------- ---------- 847
                                            3,720                           3,740
gDNA_Solyc10g044510  GTAGTTCTAT TACAATTTGG AAATTTGTCC AATTCTTTTC TTAGTTTCTT 3750
cDNASolyc10g044510   ---------- ---------- ---------- ---------- ---------- 847
SlMLO13_LEAF         ---------- ---------- ---------- ---------- ---------- 764
SlMLO13_ROOT         ---------- ---------- ---------- ---------- ---------- 847
SlMLO13_FLOWER       ---------- ---------- ---------- ---------- ---------- 847
SlMLO13_FRUIT        ---------- ---------- ---------- ---------- ---------- 847
                                3,760                           3,780                      3,800
gDNA_Solyc10g044510  GTAGAAATAA ATGTCTTGAT AGTTGTTTAT TTGTGCTGAC TTATGCTGGG 3800
cDNASolyc10g044510   ---------- ---------- ---------- ---------- ---------- 847
SlMLO13_LEAF         ---------- ---------- ---------- ---------- ---------- 764
SlMLO13_ROOT         ---------- ---------- ---------- ---------- ---------- 847
SlMLO13_FLOWER       ---------- ---------- ---------- ---------- ---------- 847
SlMLO13_FRUIT        ---------- ---------- ---------- ---------- ---------- 847
                                            3,820                           3,840
gDNA_Solyc10g044510  ACTTTGCAGC TGGCTACTTT GGGGTTATGC CATCATATGC ATCTTCATCA 3850
cDNASolyc10g044510   -----GC--- TGGCTACTTT GGGGTTATGC CATCATATGC ATCTTCATCA 889
SlMLO13_LEAF         -----GCAGC TGGCTACTTT GGGGTTATGC CATCATATGC ATCTTCATCA 809
SlMLO13_ROOT         -----GC--- TGGCTACTTT GGGGTTATGC CATCATATGC ATCTTCATCA 889
SlMLO13_FLOWER       -----GC--- TGGCTACTTT GGGGTTATGC CATCATATGC ATCTTCATCA 889
SlMLO13_FRUIT        -----GC--- TGGCTACTTT GGGGTTATGC CATCATATGC ATCTTCATCA 889
                                3,860                           3,880                      3,900
gDNA_Solyc10g044510  ACATACATGG TAATGAAATT TTTGTAACAT GAAACATCAT TTTTTTTGCT 3900
cDNASolyc10g044510   ACATACATGG T--------- ---------- ---------- ---------- 900
SlMLO13_LEAF         ACATACATGG T--------- ---------- ---------- ---------- 820
SlMLO13_ROOT         ACATACATGG T--------- ---------- ---------- ---------- 900
SlMLO13_FLOWER       ACATACATGG T--------- ---------- ---------- ---------- 900
SlMLO13_FRUIT        ACATACATGG T--------- ---------- ---------- ---------- 900
                                            3,920                           3,940
gDNA_Solyc10g044510  TGTTAAAGTT TTGAAATGAT GTTTCTTATA TTGCAGGTCT TAATATCTAC 3950
cDNASolyc10g044510   ---------- ---------- ---------- --------CT TAATATCTAC 912
SlMLO13_LEAF         ---------- ---------- ---------- -----GGTCT TAATATCTAC 835
SlMLO13_ROOT         ---------- ---------- ---------- --------CT TAATATCTAC 912
SlMLO13_FLOWER       ---------- ---------- ---------- --------CT TAATATCTAC 912
SlMLO13_FRUIT        ---------- ---------- ---------- --------CT TAATATCTAC 912
                                3,960                           3,980                      4,000
gDNA_Solyc10g044510  TTCTGGCTCT CTTTTATTCC TGCCATCGTA AGTCCGCTTA CTTTCTTTTG 4000
cDNASolyc10g044510   TTCTGGCTCT CTTTTATTCC TGCCATC--- ---------- ---------- 939
SlMLO13_LEAF         TTCTGGCTCT CTTTTATTCC TGCCATC--- ---------- ---------- 862
SlMLO13_ROOT         TTCTGGCTCT CTTTTATTCC TGCCATC--- ---------- ---------- 939
SlMLO13_FLOWER       TTCTGGCTCT CTTTTATTCC TGCCATC--- ---------- ---------- 939
SlMLO13_FRUIT        TTCTGGCTCT CTTTTATTCC TGCCATC--- ---------- ---------- 939
                                            4,020                           4,040
gDNA_Solyc10g044510  TCCATTACCT GAGTGAAATG ATCTATGTAT CTGATGCTCA ACAATAACTT 4050
cDNASolyc10g044510   ---------- ---------- ---------- ---------- ---------- 939
SlMLO13_LEAF         ---------- ---------- ---------- ---------- ---------- 862
SlMLO13_ROOT         ---------- ---------- ---------- ---------- ---------- 939
SlMLO13_FLOWER       ---------- ---------- ---------- ---------- ---------- 939
SlMLO13_FRUIT        ---------- ---------- ---------- ---------- ---------- 939
                                4,060                           4,080                      4,100
gDNA_Solyc10g044510  GTGCCAAAGC TTGTCGTGGT GGTTGGAACA AAACTACAGC ATGTAGTCTC 4100
cDNASolyc10g044510   ---------C TTGTCGTGGT GGTTGGAACA AAACTACAGC ATGTAGTCTC 980
SlMLO13_LEAF         ---------C TTGTCGTGGT GGTTGGAACA AAACTACAGC ATGTAGTCTC 903
SlMLO13_ROOT         ---------C TTGTCGTGGT GGTTGGAACA AAACTACAGC ATGTAGTCTC 980
SlMLO13_FLOWER       ---------C TTGTCGTGGT GGTTGGAACA AAACTACAGC ATGTAGTCTC 980
SlMLO13_FRUIT        ---------C TTGTCGTGGT GGTTGGAACA AAACTACAGC ATGTAGTCTC 980
                                            4,120                           4,140
gDNA_Solyc10g044510  CTCGTTGGCA CTTGAAATTG CAGAGCCGAA GGGTCCACTT ATTGGATTAC 4150
cDNASolyc10g044510   CTCGTTGGCA CTTGAAATTG CAGAGCCGAA GGGTCCACTT ATTGGATTAC 1030
SlMLO13_LEAF         CTCGTTGGCA CTTGAAATTG CAGAGCCGAA GGGTCCACTT ATTGGATTAC 953
SlMLO13_ROOT         CTCGTTGGCA CTTGAAATTG CAGAGCCGAA GGGTCCACTT ATTGGATTAC 1030
SlMLO13_FLOWER       CTCGTTGGCA CTTGAAATTG CAGAGCCGAA GGGTCCACTT ATTGGATTAC 1030
SlMLO13_FRUIT        CTCGTTGGCA CTTGAAATTG CAGAGCCGAA GGGTCCACTT ATTGGATTAC 1030
                                4,160                           4,180                      4,200
gDNA_Solyc10g044510  AAGTAAAGCC ACGCGATGAA TTGTTTTGGT TTGGAAAACC GAAGATACTA 4200
cDNASolyc10g044510   AAGTAAAGCC ACGCGATGAA TTGTTTTGGT TTGGAAAACC GAAGATACTA 1080
SlMLO13_LEAF         AAGTAAAGCC ACGCGATGAA TTGTTTTGGT TTGGAAAACC GAAGATACTA 1003
SlMLO13_ROOT         AAGTAAAGCC ACGCGATGAA TTGTTTTGGT TTGGAAAACC GAAGATACTA 1080
SlMLO13_FLOWER       AAGTAAAGCC ACGCGATGAA TTGTTTTGGT TTGGAAAACC GAAGATACTA 1080
SlMLO13_FRUIT        AAGTAAAGCC ACGCGATGAA TTGTTTTGGT TTGGAAAACC GAAGATACTA 1080
```

```
                                      4,220                    4,240
gDNA_Solyc10g044510  TTACGACTGA TACAGTTTAT ATCATTTCAG GTCAAATTCC AAAAGCTCTG 4250
cDNASolyc10g044510   TTACGACTGA TACAGTTTAT ATCATTTCAG ---------- ---------- 1110
SlMLO13_LEAF         TTACGACTGA TACAGTTTAT ATCATTTCAG ---------- ---------- 1033
SlMLO13_ROOT         TTACGACTGA TACAGTTTAT ATCATTTCAG ---------- ---------- 1110
SlMLO13_FLOWER       TTACGACTGA TACAGTTTAT ATCATTTCAG ---------- ---------- 1110
SlMLO13_FRUIT        TTACGACTGA TACAGTTTAT ATCATTTCAG ---------- ---------- 1110
                              4,260                    4,280                  4,300
gDNA_Solyc10g044510  ATGCTCAATT CATTGTTCAA TGTTCATTTC AAGCAAAACA AGTGAAAAGA 4300
cDNASolyc10g044510   ---------- ---------- ---------- ---------- ---------- 1110
SlMLO13_LEAF         ---------- ---------- ---------- ---------- ---------- 1033
SlMLO13_ROOT         ---------- ---------- ---------- ---------- ---------- 1110
SlMLO13_FLOWER       ---------- ---------- ---------- ---------- ---------- 1110
SlMLO13_FRUIT        ---------- ---------- ---------- ---------- ---------- 1110
                                      4,320                    4,340
gDNA_Solyc10g044510  TAAAAAGATA TGCATGGTTA AATGTCCTAA CATCGATATT TTTGTGTGCA 4350
cDNASolyc10g044510   ---------- ---------- ---------- ---------- ---------- 1110
SlMLO13_LEAF         ---------- ---------- ---------- ---------- ---------- 1033
SlMLO13_ROOT         ---------- ---------- ---------- ---------- ---------- 1110
SlMLO13_FLOWER       ---------- ---------- ---------- ---------- ---------- 1110
SlMLO13_FRUIT        ---------- ---------- ---------- ---------- ---------- 1110
                              4,360                    4,380                  4,400
gDNA_Solyc10g044510  CAGAATGCTT TTGAAATGGC AACCTTTATC TGGTCCTTGG TGAGATTCGT 4400
cDNASolyc10g044510   ---AATGCTT TTGAAATGGC AACCTTTATC TGGTCCTTG- ---------- 1146
SlMLO13_LEAF         ---AATGCTT TTGAAATGGC AACCTTTATC TGGTCCTTG- ---------- 1069
SlMLO13_ROOT         ---AATGCTT TTGAAATGGC AACCTTTATC TGGTCCTTG- ---------- 1146
SlMLO13_FLOWER       ---AATGCTT TTGAAATGGC AACCTTTATC TGGTCCTTG- ---------- 1146
SlMLO13_FRUIT        ---AATGCTT TTGAAATGGC AACCTTTATC TGGTCCTTG- ---------- 1146
                                      4,420                    4,440
gDNA_Solyc10g044510  CGCCTTTCTT CTTTACTATA ATTCATGGAG CATCTTGTTT AATGATTACT 4450
cDNASolyc10g044510   ---------- ---------- ---------- ---------- ---------- 1146
SlMLO13_LEAF         ---------- ---------- ---------- ---------- ---------- 1069
SlMLO13_ROOT         ---------- ---------- ---------- ---------- ---------- 1146
SlMLO13_FLOWER       ---------- ---------- ---------- ---------- ---------- 1146
SlMLO13_FRUIT        ---------- ---------- ---------- ---------- ---------- 1146
                              4,460                    4,480                  4,500
gDNA_Solyc10g044510  ACTTTTTCTC AAGTCAATAT GTAAATAAAT TTCTCTTCTG GCTGAAAATG 4500
cDNASolyc10g044510   ---------- ---------- ---------- ---------- ---------- 1146
SlMLO13_LEAF         ---------- ---------- ---------- ---------- ---------- 1069
SlMLO13_ROOT         ---------- ---------- ---------- ---------- ---------- 1146
SlMLO13_FLOWER       ---------- ---------- ---------- ---------- ---------- 1146
SlMLO13_FRUIT        ---------- ---------- ---------- ---------- ---------- 1146
                                      4,520                    4,540
gDNA_Solyc10g044510  AATGAATGTG GAAGGAAATT GTTCTCCAAA ATGTCATTGC TAGGAAATTA 4550
cDNASolyc10g044510   ---------- ---------- ---------- ---------- ---------- 1146
SlMLO13_LEAF         ---------- ---------- ---------- ---------- ---------- 1069
SlMLO13_ROOT         ---------- ---------- ---------- ---------- ---------- 1146
SlMLO13_FLOWER       ---------- ---------- ---------- ---------- ---------- 1146
SlMLO13_FRUIT        ---------- ---------- ---------- ---------- ---------- 1146
                              4,560                    4,580                  4,600
gDNA_Solyc10g044510  AGAGCGAGGG GGGTGAATTA AAATTCCATC CCTAGTCGAC TTTCCTGTGG 4600
cDNASolyc10g044510   ---------- ---------- ---------- ---------- ---------- 1146
SlMLO13_LEAF         ---------- ---------- ---------- ---------- ---------- 1069
SlMLO13_ROOT         ---------- ---------- ---------- ---------- ---------- 1146
SlMLO13_FLOWER       ---------- ---------- ---------- ---------- ---------- 1146
SlMLO13_FRUIT        ---------- ---------- ---------- ---------- ---------- 1146
                                      4,620                    4,640
gDNA_Solyc10g044510  GTTAGTATAA CGGATAATAG ATAAATTTAA AGAGACAACA ACAAGAATAT 4650
cDNASolyc10g044510   ---------- ---------- ---------- ---------- ---------- 1146
SlMLO13_LEAF         ---------- ---------- ---------- ---------- ---------- 1069
SlMLO13_ROOT         ---------- ---------- ---------- ---------- ---------- 1146
SlMLO13_FLOWER       ---------- ---------- ---------- ---------- ---------- 1146
SlMLO13_FRUIT        ---------- ---------- ---------- ---------- ---------- 1146
                              4,660                    4,680                  4,700
gDNA_Solyc10g044510  GACAAACTAC TCCTATGAAA GAGGATCTTT GGTCTAACTC AATCCTGCAA 4700
cDNASolyc10g044510   ---------- ---------- ---------- ---------- ---------- 1146
SlMLO13_LEAF         ---------- ---------- ---------- ---------- ---------- 1069
SlMLO13_ROOT         ---------- ---------- ---------- ---------- ---------- 1146
SlMLO13_FLOWER       ---------- ---------- ---------- ---------- ---------- 1146
SlMLO13_FRUIT        ---------- ---------- ---------- ---------- ---------- 1146
                                      4,720                    4,740
gDNA_Solyc10g044510  GATAATTCAT GAAGTAAGGA CTCCTAAGAT CATATAATGA GACAACAACA 4750
cDNASolyc10g044510   ---------- ---------- ---------- ---------- ---------- 1146
SlMLO13_LEAF         ---------- ---------- ---------- ---------- ---------- 1069
SlMLO13_ROOT         ---------- ---------- ---------- ---------- ---------- 1146
SlMLO13_FLOWER       ---------- ---------- ---------- ---------- ---------- 1146
SlMLO13_FRUIT        ---------- ---------- ---------- ---------- ---------- 1146
                              4,760                    4,780                  4,800
gDNA_Solyc10g044510  AAAGCTCAAC CTATAGCGGA GGGTTAGGCT CCTGGAGTAT GAACAATATG 4800
cDNASolyc10g044510   ---------- ---------- ---------- ---------- ---------- 1146
SlMLO13_LEAF         ---------- ---------- ---------- ---------- ---------- 1069
SlMLO13_ROOT         ---------- ---------- ---------- ---------- ---------- 1146
SlMLO13_FLOWER       ---------- ---------- ---------- ---------- ---------- 1146
SlMLO13_FRUIT        ---------- ---------- ---------- ---------- ---------- 1146
```

```
                                        4,820                          4,840
gDNA_Solyc10g044510  ACATGGGGGG CCAACATTGG GTAAATCAAG AATAGGAATG AGTGCCGCTC 4850
 cDNASolyc10g044510  ---------- ---------- ---------- ---------- ---------- 1146
       SlMLO13_LEAF  ---------- ---------- ---------- ---------- ---------- 1069
       SlMLO13_ROOT  ---------- ---------- ---------- ---------- ---------- 1146
     SlMLO13_FLOWER  ---------- ---------- ---------- ---------- ---------- 1146
      SlMLO13_FRUIT  ---------- ---------- ---------- ---------- ---------- 1146

                          4,860                          4,880                4,900
gDNA_Solyc10g044510  TGAGTTCGAT ACCATATTAA GATAGACGTT GAGCCTAATT TAATCCCAAA 4900
 cDNASolyc10g044510  ---------- ---------- ---------- ---------- ---------- 1146
       SlMLO13_LEAF  ---------- ---------- ---------- ---------- ---------- 1069
       SlMLO13_ROOT  ---------- ---------- ---------- ---------- ---------- 1146
     SlMLO13_FLOWER  ---------- ---------- ---------- ---------- ---------- 1146
      SlMLO13_FRUIT  ---------- ---------- ---------- ---------- ---------- 1146

                                        4,920                          4,940
gDNA_Solyc10g044510  AGCTAACAGA TGAGGTGAGG ATCACGAAGA TCATATGAGG AACAACAATA 4950
 cDNASolyc10g044510  ---------- ---------- ---------- ---------- ---------- 1146
       SlMLO13_LEAF  ---------- ---------- ---------- ---------- ---------- 1069
       SlMLO13_ROOT  ---------- ---------- ---------- ---------- ---------- 1146
     SlMLO13_FLOWER  ---------- ---------- ---------- ---------- ---------- 1146
      SlMLO13_FRUIT  ---------- ---------- ---------- ---------- ---------- 1146

                          4,960                          4,980                5,000
gDNA_Solyc10g044510  CATCACTTCA ACCAACAGCA GACACTTAAC AAGTGCAATA CAAGTGACCT 5000
 cDNASolyc10g044510  ---------- ---------- ---------- ---------- ---------- 1146
       SlMLO13_LEAF  ---------- ---------- ---------- ---------- ---------- 1069
       SlMLO13_ROOT  ---------- ---------- ---------- ---------- ---------- 1146
     SlMLO13_FLOWER  ---------- ---------- ---------- ---------- ---------- 1146
      SlMLO13_FRUIT  ---------- ---------- ---------- ---------- ---------- 1146

                                        5,020                          5,040
gDNA_Solyc10g044510  TTTGGAGAGC TAGCTACTCG TTTTGGCTGT TTTCATTCTC AAACCATGTT 5050
 cDNASolyc10g044510  ---------- ---------- ---------- ---------- ---------- 1146
       SlMLO13_LEAF  ---------- ---------- ---------- ---------- ---------- 1069
       SlMLO13_ROOT  ---------- ---------- ---------- ---------- ---------- 1146
     SlMLO13_FLOWER  ---------- ---------- ---------- ---------- ---------- 1146
      SlMLO13_FRUIT  ---------- ---------- ---------- ---------- ---------- 1146

                          5,060                          5,080                5,100
gDNA_Solyc10g044510  GTTGGTATTG CCTTAGAATT TCTTTTCTCT GAAGATGCGG TTTGTTCTTT 5100
 cDNASolyc10g044510  ---------- ---------- ---------- ---------- ---------- 1146
       SlMLO13_LEAF  ---------- ---------- ---------- ---------- ---------- 1069
       SlMLO13_ROOT  ---------- ---------- ---------- ---------- ---------- 1146
     SlMLO13_FLOWER  ---------- ---------- ---------- ---------- ---------- 1146
      SlMLO13_FRUIT  ---------- ---------- ---------- ---------- ---------- 1146

                                        5,120                          5,140
gDNA_Solyc10g044510  TGGATCATTG ATTCACCTCA TATCAATATT AATCCATTTG TGCAGTGGGG 5150
 cDNASolyc10g044510  ---------- ---------- ---------- ---------- -----TGGGG 1151
       SlMLO13_LEAF  ---------- ---------- ---------- ---------- ---------- 1069
       SlMLO13_ROOT  ---------- ---------- ---------- ---------- -----TGGGG 1151
     SlMLO13_FLOWER  ---------- ---------- ---------- ---------- -----TGGGG 1151
      SlMLO13_FRUIT  ---------- ---------- ---------- ---------- -----TGGGG 1151

                          5,160                          5,180                5,200
gDNA_Solyc10g044510  ATTGAAGCAA CGATCGTGCT TCATGAAGAA CCATGCAATG GTTATGATCA 5200
 cDNASolyc10g044510  ATTGAAGCAA CGATCGTGCT TCATGAAGAA CCATGCAATG GTTATGATCA 1201
       SlMLO13_LEAF  ---------- ---------- ---------- ---------- ---------- 1069
       SlMLO13_ROOT  ATTGAAGCAA CGATCGTGCT TCATGAAGAA CCATGCAATG GTTATGATCA 1201
     SlMLO13_FLOWER  ATTGAAGCAA CGATCGTGCT TCATGAAGAA CCATGCAATG GTTATGATCA 1201
      SlMLO13_FRUIT  ATTGAAGCAA CGATCGTGCT TCATGAAGAA CCATGCAATG GTTATGATCA 1201

                                        5,220                          5,240
gDNA_Solyc10g044510  GATTGATTTC AGGGTATGTA TATTACGTCT TGACCACCTA CTCCATCCCC 5250
 cDNASolyc10g044510  GATTGATTTC AGGG------ ---------- ---------- ---------- 1215
       SlMLO13_LEAF  ---------- -GGG------ ---------- ---------- ---------- 1072
       SlMLO13_ROOT  GATTGATTTC AGGG------ ---------- ---------- ---------- 1215
     SlMLO13_FLOWER  GATTGATTTC AGGG------ ---------- ---------- ---------- 1215
      SlMLO13_FRUIT  GATTGATTTC AGGG------ ---------- ---------- ---------- 1215

                          5,260                          5,280                5,300
gDNA_Solyc10g044510  CTTGAATTTA AAGTTGGTAT GCCAAAACCA TGAAATCAAT CCATATTAAG 5300
 cDNASolyc10g044510  ---------- ---------- ---------- ---------- ---------- 1215
       SlMLO13_LEAF  ---------- ---------- ---------- ---------- ---------- 1072
       SlMLO13_ROOT  ---------- ---------- ---------- ---------- ---------- 1215
     SlMLO13_FLOWER  ---------- ---------- ---------- ---------- ---------- 1215
      SlMLO13_FRUIT  ---------- ---------- ---------- ---------- ---------- 1215

                                        5,320                          5,340
gDNA_Solyc10g044510  TTGTTCCAGG GAACTTCTCT TAAAAGAAAA CACCCTCCTT ATGCCCTTAA 5350
 cDNASolyc10g044510  ---------- ---------- ---------- ---------- ---------- 1215
       SlMLO13_LEAF  ---------- ---------- ---------- ---------- ---------- 1072
       SlMLO13_ROOT  ---------- ---------- ---------- ---------- ---------- 1215
     SlMLO13_FLOWER  ---------- ---------- ---------- ---------- ---------- 1215
      SlMLO13_FRUIT  ---------- ---------- ---------- ---------- ---------- 1215

                          5,360                          5,380                5,400
gDNA_Solyc10g044510  GTACGTCTCT ATCCTTCATT ATGAAAGTAC CATCATGAAA TCACTTCCGC 5400
 cDNASolyc10g044510  ---------- ---------- ---------- ---------- ---------- 1215
       SlMLO13_LEAF  ---------- ---------- ---------- ---------- ---------- 1072
       SlMLO13_ROOT  ---------- ---------- ---------- ---------- ---------- 1215
     SlMLO13_FLOWER  ---------- ---------- ---------- ---------- ---------- 1215
      SlMLO13_FRUIT  ---------- ---------- ---------- ---------- ---------- 1215
```

```
                                      5,420                          5,440
gDNA_Solyc10g044510  ATTTACTATA GTCCAAGTTC TGAAGAACTT TACTTTTACG TCCACTTCTT 5450
 cDNASolyc10g044510  ---------- ---------- ---------- ---------- ---------- 1215
        SlMLO13_LEAF ---------- ---------- ---------- ---------- ---------- 1072
        SlMLO13_ROOT ---------- ---------- ---------- ---------- ---------- 1215
      SlMLO13_FLOWER ---------- ---------- ---------- ---------- ---------- 1215
       SlMLO13_FRUIT ---------- ---------- ---------- ---------- ---------- 1215
                           5,460                         5,480                5,500
gDNA_Solyc10g044510  GTTCATTCTT TCCTTTTTAC TTTGTTTAGT ATATTCCTAT CAGATAGTGC 5500
 cDNASolyc10g044510  ---------- ---------- ---------- ---------- ---------- 1215
        SlMLO13_LEAF ---------- ---------- ---------- ---------- ---------- 1072
        SlMLO13_ROOT ---------- ---------- ---------- ---------- ---------- 1215
      SlMLO13_FLOWER ---------- ---------- ---------- ---------- ---------- 1215
       SlMLO13_FRUIT ---------- ---------- ---------- ---------- ---------- 1215
                                      5,520                          5,540
gDNA_Solyc10g044510  TCCTGTAGTA ACCTATTTTA GGTCATCCCC ATCTTAGCTC CATTGTTACA 5550
 cDNASolyc10g044510  ---------- ---------- ---------- ---------- ---------- 1215
        SlMLO13_LEAF ---------- ---------- ---------- ---------- ---------- 1072
        SlMLO13_ROOT ---------- ---------- ---------- ---------- ---------- 1215
      SlMLO13_FLOWER ---------- ---------- ---------- ---------- ---------- 1215
       SlMLO13_FRUIT ---------- ---------- ---------- ---------- ---------- 1215
                           5,560                         5,580                5,600
gDNA_Solyc10g044510  TCTTCATTGA TCATGTAGTT CTCCTATAAG ACTGAGCCTA GTTATATGAG 5600
 cDNASolyc10g044510  ---------- ---------- ---------- ---------- ---------- 1215
        SlMLO13_LEAF ---------- ---------- ---------- ---------- ---------- 1072
        SlMLO13_ROOT ---------- ---------- ---------- ---------- ---------- 1215
      SlMLO13_FLOWER ---------- ---------- ---------- ---------- ---------- 1215
       SlMLO13_FRUIT ---------- ---------- ---------- ---------- ---------- 1215
                                      5,620                          5,640
gDNA_Solyc10g044510  CTGTTTTCAC ATAGAACCGT CTAGTCTCAC TTTTCCATCG TTTCTTATGG 5650
 cDNASolyc10g044510  ---------- ---------- ---------- ---------- ---------- 1215
        SlMLO13_LEAF ---------- ---------- ---------- ---------- ---------- 1072
        SlMLO13_ROOT ---------- ---------- ---------- ---------- ---------- 1215
      SlMLO13_FLOWER ---------- ---------- ---------- ---------- ---------- 1215
       SlMLO13_FRUIT ---------- ---------- ---------- ---------- ---------- 1215
                           5,660                         5,680                5,700
gDNA_Solyc10g044510  GGTTAAACAT CAGTGCCTTT TACTTATCTT AAACAATTGC GGAAGTTCCT 5700
 cDNASolyc10g044510  ---------- ---------- ---------- ---------- ---------- 1215
        SlMLO13_LEAF ---------- ---------- ---------- ---------- ---------- 1072
        SlMLO13_ROOT ---------- ---------- ---------- ---------- ---------- 1215
      SlMLO13_FLOWER ---------- ---------- ---------- ---------- ---------- 1215
       SlMLO13_FRUIT ---------- ---------- ---------- ---------- ---------- 1215
                                      5,720                          5,740
gDNA_Solyc10g044510  CTTAATATCA CTGTGTTATA GTTTAGTATA AACACTGTAG TGGGTCAAGT 5750
 cDNASolyc10g044510  ---------- ---------- ---------- ---------- ---------- 1215
        SlMLO13_LEAF ---------- ---------- ---------- ---------- ---------- 1072
        SlMLO13_ROOT ---------- ---------- ---------- ---------- ---------- 1215
      SlMLO13_FLOWER ---------- ---------- ---------- ---------- ---------- 1215
       SlMLO13_FRUIT ---------- ---------- ---------- ---------- ---------- 1215
                           5,760                         5,780                5,800
gDNA_Solyc10g044510  GACTCCCTTT GTTGCCAAGG TATCTCCTTC ATTGTTTATC AGTCTTGCTT 5800
 cDNASolyc10g044510  ---------- ---------- ---------- ---------- ---------- 1215
        SlMLO13_LEAF ---------- ---------- ---------- ---------- ---------- 1072
        SlMLO13_ROOT ---------- ---------- ---------- ---------- ---------- 1215
      SlMLO13_FLOWER ---------- ---------- ---------- ---------- ---------- 1215
       SlMLO13_FRUIT ---------- ---------- ---------- ---------- ---------- 1215
                                      5,820                          5,840
gDNA_Solyc10g044510  TGAAGGTTGC CTTGCATTAT CTATTTGTTT TCAGTCCTCT TTCAACTACA 5850
 cDNASolyc10g044510  ---------- ---------- ---------- ---------- ---------- 1215
        SlMLO13_LEAF ---------- ---------- ---------- ---------- ---------- 1072
        SlMLO13_ROOT ---------- ---------- ---------- ---------- ---------- 1215
      SlMLO13_FLOWER ---------- ---------- ---------- ---------- ---------- 1215
       SlMLO13_FRUIT ---------- ---------- ---------- ---------- ---------- 1215
                           5,860                         5,880                5,900
gDNA_Solyc10g044510  TACTTATACC TCTAATTTTC TATAACTAAA GCTTATCCAT TGATCTTACA 5900
 cDNASolyc10g044510  ---------- ---------- ---------- ---------- ---------- 1215
        SlMLO13_LEAF ---------- ---------- ---------- ---------- ---------- 1072
        SlMLO13_ROOT ---------- ---------- ---------- ---------- ---------- 1215
      SlMLO13_FLOWER ---------- ---------- ---------- ---------- ---------- 1215
       SlMLO13_FRUIT ---------- ---------- ---------- ---------- ---------- 1215
                                      5,920                          5,940
gDNA_Solyc10g044510  TTCTAGTATG TAAGTTCACC CCTCAATCTT CTTTGATCAG ATCTTATTCA 5950
 cDNASolyc10g044510  ---------- ---------- ---------- ---------- ---------- 1215
        SlMLO13_LEAF ---------- ---------- ---------- ---------- ---------- 1072
        SlMLO13_ROOT ---------- ---------- ---------- ---------- ---------- 1215
      SlMLO13_FLOWER ---------- ---------- ---------- ---------- ---------- 1215
       SlMLO13_FRUIT ---------- ---------- ---------- ---------- ---------- 1215
                           5,960                         5,980                6,000
gDNA_Solyc10g044510  ATGAAACTAG TTCACAGATA ATGCATTAAC GATCAGTTGT TGACATTGAG 6000
 cDNASolyc10g044510  ---------- ---------- ---------- ---------- ---------- 1215
        SlMLO13_LEAF ---------- ---------- ---------- ---------- ---------- 1072
        SlMLO13_ROOT ---------- ---------- ---------- ---------- ---------- 1215
      SlMLO13_FLOWER ---------- ---------- ---------- ---------- ---------- 1215
       SlMLO13_FRUIT ---------- ---------- ---------- ---------- ---------- 1215
```

```
                                           6,020                         6,040
gDNA_Solyc10g044510  AAAATGCTTC AAGCTGTTAA CTCTGTTATT TATACAAGTT TTGTCGGATT 6050
 cDNASolyc10g044510  ---------- ---------- ---------- ---------- ---------- 1215
       SIMLO13_LEAF  ---------- ---------- ---------- ---------- ---------- 1072
       SIMLO13_ROOT  ---------- ---------- ---------- ---------- ---------- 1215
     SIMLO13_FLOWER  ---------- ---------- ---------- ---------- ---------- 1215
      SIMLO13_FRUIT  ---------- ---------- ---------- ---------- ---------- 1215
                               6,060                         6,080                6,100
gDNA_Solyc10g044510  TGTCCATTTT CACCTTGTAT GTTAAGCTAA AAACTTCAAG TTTCAGTCCT 6100
 cDNASolyc10g044510  ---------- ---------- ---------- ---------- ---------- 1215
       SIMLO13_LEAF  ---------- ---------- ---------- ---------- ---------- 1072
       SIMLO13_ROOT  ---------- ---------- ---------- ---------- ---------- 1215
     SIMLO13_FLOWER  ---------- ---------- ---------- ---------- ---------- 1215
      SIMLO13_FRUIT  ---------- ---------- ---------- ---------- ---------- 1215
                                           6,120                         6,140
gDNA_Solyc10g044510  GTTAGTTTCT TCATTAATCC ATCATTACCA AGTCCAGTAC TTTCAAAATA 6150
 cDNASolyc10g044510  ---------- ---------- ---------- ---------- ---------- 1215
       SIMLO13_LEAF  ---------- ---------- ---------- ---------- ---------- 1072
       SIMLO13_ROOT  ---------- ---------- ---------- ---------- ---------- 1215
     SIMLO13_FLOWER  ---------- ---------- ---------- ---------- ---------- 1215
      SIMLO13_FRUIT  ---------- ---------- ---------- ---------- ---------- 1215
                               6,160                         6,180                6,200
gDNA_Solyc10g044510  AAATGTCAAT GGCCAACGAA ACCAAAAGAT ATAATTGCTG AGTGTTACAT 6200
 cDNASolyc10g044510  ---------- ---------- ---------- ---------- ---------- 1215
       SIMLO13_LEAF  ---------- ---------- ---------- ---------- ---------- 1072
       SIMLO13_ROOT  ---------- ---------- ---------- ---------- ---------- 1215
     SIMLO13_FLOWER  ---------- ---------- ---------- ---------- ---------- 1215
      SIMLO13_FRUIT  ---------- ---------- ---------- ---------- ---------- 1215
                                           6,220                         6,240
gDNA_Solyc10g044510  CAGAATCTGG CTCCTTAAAT TAATGGCTCT TATGACTCTT CTAAGTAGTT 6250
 cDNASolyc10g044510  ---------- ---------- ---------- ---------- ---------- 1215
       SIMLO13_LEAF  ---------- ---------- ---------- ---------- ---------- 1072
       SIMLO13_ROOT  ---------- ---------- ---------- ---------- ---------- 1215
     SIMLO13_FLOWER  ---------- ---------- ---------- ---------- ---------- 1215
      SIMLO13_FRUIT  ---------- ---------- ---------- ---------- ---------- 1215
                               6,260                         6,280                6,300
gDNA_Solyc10g044510  ATTGAACTAA AGTAGAGCTA TTAAGACTGT CTTTCTTGAA TAAAGCATGG 6300
 cDNASolyc10g044510  ---------- ---------- ---------- ---------- ---------- 1215
       SIMLO13_LEAF  ---------- ---------- ---------- ---------- ---------- 1072
       SIMLO13_ROOT  ---------- ---------- ---------- ---------- ---------- 1215
     SIMLO13_FLOWER  ---------- ---------- ---------- ---------- ---------- 1215
      SIMLO13_FRUIT  ---------- ---------- ---------- ---------- ---------- 1215
                                           6,320                         6,340
gDNA_Solyc10g044510  AACGGAAATC AAGTAAAAGA CTAACTACCA ACACATATAT CATGGTTTTC 6350
 cDNASolyc10g044510  ---------- ---------- ---------- ---------- ---------- 1215
       SIMLO13_LEAF  ---------- ---------- ---------- ---------- ---------- 1072
       SIMLO13_ROOT  ---------- ---------- ---------- ---------- ---------- 1215
     SIMLO13_FLOWER  ---------- ---------- ---------- ---------- ---------- 1215
      SIMLO13_FRUIT  ---------- ---------- ---------- ---------- ---------- 1215
                               6,360                         6,380                6,400
gDNA_Solyc10g044510  TTCTTGTTAA TTAAAAAATA TATTCATTGA GAATGATTCT TGCTTTTTCA 6400
 cDNASolyc10g044510  ---------- ---------- ---------- ---------- ---------- 1215
       SIMLO13_LEAF  ---------- ---------- ---------- ---------- ---------- 1072
       SIMLO13_ROOT  ---------- ---------- ---------- ---------- ---------- 1215
     SIMLO13_FLOWER  ---------- ---------- ---------- ---------- ---------- 1215
      SIMLO13_FRUIT  ---------- ---------- ---------- ---------- ---------- 1215
                                           6,420                         6,440
gDNA_Solyc10g044510  GATGCTACAT TATTTTTTAG ATGCATAATA TTTTATATAA TTAGTCTTAT 6450
 cDNASolyc10g044510  ---------- ---------- ---------- ---------- ---------- 1215
       SIMLO13_LEAF  ---------- ---------- ---------- ---------- ---------- 1072
       SIMLO13_ROOT  ---------- ---------- ---------- ---------- ---------- 1215
     SIMLO13_FLOWER  ---------- ---------- ---------- ---------- ---------- 1215
      SIMLO13_FRUIT  ---------- ---------- ---------- ---------- ---------- 1215
                               6,460                         6,480                6,500
gDNA_Solyc10g044510  ATTTATTTAT AAATGGACCA ATTTGGCACT CACTCTATGT ACAGGGTTCT 6500
 cDNASolyc10g044510  ---------- ---------- ---------- ---------- -----GTTCT 1220
       SIMLO13_LEAF  ---------- ---------- ---------- ---------- -----GTTCT 1077
       SIMLO13_ROOT  ---------- ---------- ---------- ---------- -----GTTCT 1220
     SIMLO13_FLOWER  ---------- ---------- ---------- ---------- -----GTTCT 1220
      SIMLO13_FRUIT  ---------- ---------- ---------- ---------- -----GTTCT 1220
                                           6,520                         6,540
gDNA_Solyc10g044510  TGTGCAGTTC TGGTGTAGCC ACAGTACTGT TCCTTTAAAT GTGATTATCT 6550
 cDNASolyc10g044510  TGTGCAGTTC TGGTGTAGCC ACAGTACTGT TCCTTTAAAT GTGATTATCT 1270
       SIMLO13_LEAF  TGTGCAGTTC TGGTGTAGCC ACAGTACTGT TCCTTTAAAT GTGATTATCT 1127
       SIMLO13_ROOT  TGTGCAGTTC TGGTGTAGCC ACAGTACTGT TCCTTTAAAT GTGATTATCT 1270
     SIMLO13_FLOWER  TGTGCAGTTC TGGTGTAGCC ACAGTACTGT TCCTTTAAAT GTGATTATCT 1270
      SIMLO13_FRUIT  TGTGCAGTTC TGGTGTAGCC ACAGTACTGT TCCTTTAAAT GTGATTATCT 1270
                               6,560                         6,580                6,600
gDNA_Solyc10g044510  CGCAGGTGAT CCTTCTTTCT TGTTTATATC TCTAGCATAA TGCATAGTAA 6600
 cDNASolyc10g044510  CGCAG----- ---------- ---------- ---------- ---------- 1275
       SIMLO13_LEAF  CGCAG----- ---------- ---------- ---------- ---------- 1132
       SIMLO13_ROOT  CGCAG----- ---------- ---------- ---------- ---------- 1275
     SIMLO13_FLOWER  CGCAG----- ---------- ---------- ---------- ---------- 1275
      SIMLO13_FRUIT  CGCAG----- ---------- ---------- ---------- ---------- 1275
```

```
                                       6,620                         6,640
                                         |                             |
gDNA_Solyc10g044510  GTATTGATAT GCCAATTGAA CTAGTCACAT AGAGACAGAT ATCAGCTTTC 6650
cDNASolyc10g044510   ---------- ---------- ---------- ---------- ---------- 1275
     SlMLO13_LEAF    ---------- ---------- ---------- ---------- ---------- 1132
     SlMLO13_ROOT    ---------- ---------- ---------- ---------- ---------- 1275
   SlMLO13_FLOWER    ---------- ---------- ---------- ---------- ---------- 1275
    SlMLO13_FRUIT    ---------- ---------- ---------- ---------- ---------- 1275

                           6,660                         6,680                        6,700
                             |                             |                            |
gDNA_Solyc10g044510  CAATAATTTT TTGGTTTACA GATGGGTTCA CGGTGTGGAA AGGCTCTCGT 6700
cDNASolyc10g044510   ---------- ---------- -ATGGGTTCA CGGTGTGGAA AGGCTCTCGT 1304
     SlMLO13_LEAF    ---------- --------CA GATGGGTTCA CGGTGTGGAA AGGCTCTCGT 1164
     SlMLO13_ROOT    ---------- ---------- -ATGGGTTCA CGGTGTGGAA AGGCTCTCGT 1304
   SlMLO13_FLOWER    ---------- ---------- -ATGGGTTCA CGGTGTGGAA AGGCTCTCGT 1304
    SlMLO13_FRUIT    ---------- ---------- -ATGGGTTCA CGGTGTGGAA AGGCTCTCGT 1304

                                       6,720                         6,740
                                         |                             |
gDNA_Solyc10g044510  AGCTGAAAGT GTTCGAGACT CACTCCACAG TTGGTGTAAA AGAGTAAAGG 6750
cDNASolyc10g044510   AGCTGAAAGT GTTCGAGACT CACTCCACAG TTGGTGTAAA AGAGTAAAGG 1354
     SlMLO13_LEAF    AGCTGAAAGT GTTCGAGACT CACTCCACAG TTGGTGTAAA AGAGTAAAGG 1214
     SlMLO13_ROOT    AGCTGAAAGT GTTCGAGACT CACTCCACAG TTGGTGTAAA AGAGTAAAGG 1354
   SlMLO13_FLOWER    AGCTGAAAGT GTTCGAGACT CACTCCACAG TTGGTGTAAA AGAGTAAAGG 1354
    SlMLO13_FRUIT    AGCTGAAAGT GTTCGAGACT CACTCCACAG TTGGTGTAAA AGAGTAAAGG 1354

                           6,760                         6,780                        6,800
                             |                             |                            |
gDNA_Solyc10g044510  ACAGATCGAA ACACGATGCT CTACGATCTA TAACTACAAG ATCAACATGT 6800
cDNASolyc10g044510   ACAGATCGAA ACACGATGCT CTACGATCTA TAACTACAAG ATCAACATGT 1404
     SlMLO13_LEAF    ACAGATCGAA ACACGATGCT CTACGATCTA TAACTACAAG ATCAACATGT 1264
     SlMLO13_ROOT    ACAGATCGAA ACACGATGCT CTACGATCTA TAACTACAAG ATCAACATGT 1404
   SlMLO13_FLOWER    ACAGATCGAA ACACGATGCT CTACGATCTA TAACTACAAG ATCAACATGT 1404
    SlMLO13_FRUIT    ACAGATCGAA ACACGATGCT CTACGATCTA TAACTACAAG ATCAACATGT 1404

                                       6,820                         6,840
                                         |                             |
gDNA_Solyc10g044510  TCCCTTGGAT CAACAATTGA TGAGGGGGAT GAGATAGCAA CAGTGGCATC 6850
cDNASolyc10g044510   TCCCTTGGAT CAACAATTGA TGAGGGGGAT GAGATAGCAA CAGTGGCATC 1454
     SlMLO13_LEAF    TCCCTTGGAT CAACAATTGA TGAGGGGGAT GAGATAGCAA CAGTGGCATC 1314
     SlMLO13_ROOT    TCCCTTGGAT CAACAATTGA TGAGGGGGAT GAGATAGCAA CAGTGGCATC 1454
   SlMLO13_FLOWER    TCCCTTGGAT CAACAATTGA TGAGGGGGAT GAGATAGCAA CAGTGGCATC 1454
    SlMLO13_FRUIT    TCCCTTGGAT CAACAATTGA TGAGGGGGAT GAGATAGCAA CAGTGGCATC 1454

                           6,860                         6,880                        6,900
                             |                             |                            |
gDNA_Solyc10g044510  AGTAACTTTA TCTCCATGTT CCTCTAGAGG CTCATTTAAC CACCTAGATG 6900
cDNASolyc10g044510   AGTAACTTTA TCTCCATGTT CCTCTAGAGG CTCATTTAAC CACCTAGATG 1504
     SlMLO13_LEAF    AGTAACTTTA TCTCCATGTT CCTCTAGAGG CTCATTTAAC CACCTAGATG 1364
     SlMLO13_ROOT    AGTAACTTTA TCTCCATGTT CCTCTAGAGG CTCATTTAAC CACCTAGATG 1504
   SlMLO13_FLOWER    AGTAACTTTA TCTCCATGTT CCTCTAGAGG CTCATTTAAC CACCTAGATG 1504
    SlMLO13_FRUIT    AGTAACTTTA TCTCCATGTT CCTCTAGAGG CTCATTTAAC CACCTAGATG 1504

                                       6,920                         6,940
                                         |                             |
gDNA_Solyc10g044510  AGAAGGTTCT GTCTAATGAT CACCAAGAAG ATTGCATTGT TGAAACTACA 6950
cDNASolyc10g044510   AGAAGGTTCT GTCTAATGAT CACCAAGAAG ATTGCATTGT TGAAACTACA 1554
     SlMLO13_LEAF    AGAAGGTTCT GTCTAATGAT CACCAAGAAG ATTGCATTGT TGAAACTACA 1414
     SlMLO13_ROOT    AGAAGGTTCT GTCTAATGAT CACCAAGAAG ATTGCATTGT TGAAACTACA 1554
   SlMLO13_FLOWER    AGAAGGTTCT GTCTAATGAT CACCAAGAAG ATTGCATTGT TGAAACTACA 1554
    SlMLO13_FRUIT    AGAAGGTTCT GTCTAATGAT CACCAAGAAG ATTGCATTGT TGAAACTACA 1554

                           6,960                         6,980                        7,000
                             |                             |                            |
gDNA_Solyc10g044510  AACCAACCAG GTCATGAATT ATCCTTCAGA AACAGTGAAG TTCTGGTCAC 7000
cDNASolyc10g044510   AACCAACCAG GTCATGAATT ATCCTTCAGA AACAGTGAAG TTCTGGTCAC 1604
     SlMLO13_LEAF    AACCAACCAG GTCATGAATT ATCCTTCAGA AACAGTGAAG TTCTGGTCAC 1464
     SlMLO13_ROOT    AACCAACCAG GTCATGAATT ATCCTTCAGA AACAGTGAAG TTCTGGTCAC 1604
   SlMLO13_FLOWER    AACCAACCAG GTCATGAATT ATCCTTCAGA AACAGTGAAG TTCTGGTCAC 1604
    SlMLO13_FRUIT    AACCAACCAG GTCATGAATT ATCCTTCAGA AACAGTGAAG TTCTGGTCAC 1604

                                       7,020                         7,040
                                         |                             |
gDNA_Solyc10g044510  TGATGCAGAG GAGATTGTGG ATGATGAAGC AGACAAGATA GAAACTCTCT 7050
cDNASolyc10g044510   TGATGCAGAG GAGATTGTGG ATGATGAAGC AGACAAGATA GAAACTCTCT 1654
     SlMLO13_LEAF    TGATGCAGAG GAGATTGTGG ATGATGAAGC AGACAAGATA GAAACTCTCT 1514
     SlMLO13_ROOT    TGATGCAGAG GAGATTGTGG ATGATGAAGC AGACAAGATA GAAACTCTCT 1654
   SlMLO13_FLOWER    TGATGCAGAG GAGATTGTGG ATGATGAAGC AGACAAGATA GAAACTCTCT 1654
    SlMLO13_FRUIT    TGATGCAGAG GAGATTGTGG ATGATGAAGC AGACAAGATA GAAACTCTCT 1654

                           7,060
                             |
gDNA_Solyc10g044510  TCGAATTGTT CCAGAAGACG TAA 7073
cDNASolyc10g044510   TCGAATTGTT CCAGAAGACG TAA 1677
     SlMLO13_LEAF    TCGAATTGTT CCAGAAGACG TAA 1537
     SlMLO13_ROOT    TCGAATTGTT CCAGAAGACG TAA 1677
   SlMLO13_FLOWER    TCGAATTGTT CCAGAAGACG TAA 1677
    SlMLO13_FRUIT    TCGAATTGTT CCAGAAGACG TAA 1677
```

```
                                                20                            40
                                                 |                             |
gDNA_Solyc02g077570  ATGGCAGGTT CAAACAACAG TAGAGTCATA ACTCTTGTGA CAACACCAAC  50
cDNA_Solyc02g077570  ATGGCAGGTT CAAACAACAG TAGAGTCATA ACTCTTGTGA CAACACCAAC  50
       SIMLO15_LEAF  ATGGCAGGTT CAAACAACAG TAGAGTCATA ACTCTTGTGA CAACACCAAC  50
       SIMLO15_ROOT  ATGGCAGGTT CAAACAACAG TAGAGTCATA ACTCTTGTGA CAACACCAAC  50
     SIMLO15_FLOWER  ATGGCAGGTT CAAACAACAG TAGAGTCATA ACTCTTGTGA CAACACCAAC  50
      SIMLO15_FRUIT  ---------- ---------- ---------- ---------- ----------  -
                              60                            80                          100
                               |                             |                            |
gDNA_Solyc02g077570  ATGGGCTATT GCTGTTGTGT GTTTCATTTT AATTACTATT TCCATTCTTA 100
cDNA_Solyc02g077570  ATGGGCTATT GCTGTTGTGT GTTTCATTTT AATTACTATT TCCATTCTTA 100
       SIMLO15_LEAF  ATGGGCTATT GCTGTTGTGT GTTTCATTTT AATTACTATT TCCATTCTTA 100
       SIMLO15_ROOT  ATGGGCTATT GCTGTTGTGT GTTTCATTTT AATTACTATT TCCATTCTTA 100
     SIMLO15_FLOWER  ATGGGCTATT GCTGTTGTGT GTTTCATTTT AATTACTATT TCCATTCTTA 100
      SIMLO15_FRUIT  ---------- ---------- ---------- ---------- ----------  -
                                               120                           140
                                                 |                             |
gDNA_Solyc02g077570  TAGAACATGT CCTTCATCTC TTAGCTAAGG TATGTTTGTA TTTTTGCATG 150
cDNA_Solyc02g077570  TAGAACATGT CCTTCATCTC TTAGCTAAG- ---------- ---------- 129
       SIMLO15_LEAF  TAGAACATGT CCTTCATCTC TTAGCTAAG- ---------- ---------- 129
       SIMLO15_ROOT  TAGAACATGT CCTTCATCTC TTAGCTAAGG TATGTTTGTA TTTTTGCATG 150
     SIMLO15_FLOWER  TAGAACATGT CCTTCATCTC TTAGCTAAG- ---------- ---------- 129
      SIMLO15_FRUIT  ---------- ---------- ---------- ---------- ----------  -
                              160                           180                         200
                               |                             |                            |
gDNA_Solyc02g077570  AGACTGTTTT CGGATTTATA ACCCGACAAC AACACTTACT ATTCCTGACA 200
cDNA_Solyc02g077570  ---------- ---------- ---------- ---------- ---------- 129
       SIMLO15_LEAF  ---------- ---------- ---------- ---------- ---------- 129
       SIMLO15_ROOT  AGACTGTTTT CGGATTTATA ACCCGACAAC AACACTTACT ATTCCTGACA 200
     SIMLO15_FLOWER  ---------- ---------- ---------- ---------- ---------- 129
      SIMLO15_FRUIT  ---------- ---------- ---------- ---------- ----------  -
                                               220                           240
                                                 |                             |
gDNA_Solyc02g077570  AACTTCAAAT TTTGATTGTT AACCTTGTAC TACTTCAACA AGAAGAGGAG 250
cDNA_Solyc02g077570  ---------- ---------- ---------- ---------- ---------- 129
       SIMLO15_LEAF  ---------- ---------- ---------- ---------- ---------- 129
       SIMLO15_ROOT  AACTTCAAAT TTTGATTGTT AACCTTGTAC TACTTCAACA AGAAGAGGAG 250
     SIMLO15_FLOWER  ---------- ---------- ---------- ---------- ---------- 129
      SIMLO15_FRUIT  ---------- ---------- ---------- ---------- ----------  -
                              260                           280                         300
                               |                             |                            |
gDNA_Solyc02g077570  GAAGTCTTTG ATTCAAGCAC TAAACAATAT CAAATCAGGT ACTACTAATT 300
cDNA_Solyc02g077570  ---------- ---------- ---------- ---------- ---------- 129
       SIMLO15_LEAF  ---------- ---------- ---------- ---------- ---------- 129
       SIMLO15_ROOT  GAAGTCTTTG ATTCAAGCAC TAAACAATAT CAAATCAGGT ACTACTAATT 300
     SIMLO15_FLOWER  ---------- ---------- ---------- ---------- ---------- 129
      SIMLO15_FRUIT  ---------- ---------- ---------- ---------- ----------  -
                                               320                           340
                                                 |                             |
gDNA_Solyc02g077570  GAATATATAA TTGGTTTTAG CTAGCTAGCT GGTTGCTTGA CTTTTGTATA 350
cDNA_Solyc02g077570  ---------- ---------- ---------- ---------- ---------- 129
       SIMLO15_LEAF  ---------- ---------- ---------- ---------- ---------- 129
       SIMLO15_ROOT  GAATATATAA TTGGTTTTAG CTAGCTAGCT GGTTGCTTGA CTTTTGTATA 350
     SIMLO15_FLOWER  ---------- ---------- ---------- ---------- ---------- 129
      SIMLO15_FRUIT  ---------- ---------- ---------- ---------- ----------  -
                              360                           380                         400
                               |                             |                            |
gDNA_Solyc02g077570  TATAAAATTT TTGTTTCAAG AGTTGATGCT TTTGGGATTT ATCTCTTTAT 400
cDNA_Solyc02g077570  ---------- ---------- -----ATGCT TTTGGGATTT ATCTCTTTAT 154
       SIMLO15_LEAF  ---------- ---------- --TTGATGCT TTTGGGATTT ATCTCTTTAT 157
       SIMLO15_ROOT  TATAAAATTT TTGTTTCAAG AGTTGATGCT TTTGGGATTT ATCTCTTTAT 400
     SIMLO15_FLOWER  ---------- ---------- --AGGAGG-- ---------- ---------- 135
      SIMLO15_FRUIT  ---------- ---------- ----GATGCT TTTGGGATTT ATCTCTTTAT  26
                                               420                           440
                                                 |                             |
gDNA_Solyc02g077570  TGCTGAATGT TCTCCAAAAG CCTATTGCCA AAATTTGTAT TCCAAAGGGT 450
cDNA_Solyc02g077570  TGCTGAATGT TCTCCAAAAG CCTATTGCCA AAATTTGTAT TCCAAAGGGT 204
       SIMLO15_LEAF  TGCTGAATGT TCTCCAAAAG CCTATTGCCA AAATTTGTAT TCCAAAGGGT 207
       SIMLO15_ROOT  TGCTGAATGT TCTCCAAAAG CCTATTGCCA AAATTTGTAT TCCAAAGGGT 450
     SIMLO15_FLOWER  ---------- ---------- ---------- AAGTCTTTGA TTCAAG---- 151
      SIMLO15_FRUIT  TGCTGAATGT TCTCCAAAAG CCTATTGCCA AAATTTGTAT TCCAAAGGGT  76
                              460                           480                         500
                               |                             |                            |
gDNA_Solyc02g077570  GCTGCTCAGA CTTTTCTTCC TTGCCAAAGC TTCACAACTG ATGATGTTGA 500
cDNA_Solyc02g077570  GCTGCTCAGA CTTTTCTTCC TTGCCAAAGC TTCACAACTG ATGATGTTGA 254
       SIMLO15_LEAF  GCTGCTCAGA CTTTTCTTCC TTGCCAAAGC TTCACAACTG ATGATGTTGA 257
       SIMLO15_ROOT  GCTGCTCAGA CTTTTCTTCC TTGCCAAAGC CTCACAACTG ATGATGTTGA 500
     SIMLO15_FLOWER  ---------- ---------- ---------- ----CACTAA ACAATATCAA 167
      SIMLO15_FRUIT  GCTGCTCAGA CTTTTCTTCC TTGCCAAAGC TTCACAACTG ATGATGTTGA 126
                                               520                           540
                                                 |                             |
gDNA_Solyc02g077570  GGAGGAGTCA AACTGTGAAC AACAGGTCTT TTTTTTTATT AATTAGCTGC 550
cDNA_Solyc02g077570  GGAGGAGTCA AACTGTGAAC AACAGG---- ---------- ---------- 280
       SIMLO15_LEAF  GGAGGAGTCA AACTGTGAAC AACAGG---- ---------- ---------- 283
       SIMLO15_ROOT  GGAGGAGTCA AACTGTGAAC AACAGG---- ---------- ---------- 526
     SIMLO15_FLOWER  ---------- ---------- ATCAGG---- ---------- ---------- 173
      SIMLO15_FRUIT  GGAGGAGTCA AACTGTGAAC AACAGG---- ---------- ---------- 152
                              560                           580                         600
                               |                             |                            |
gDNA_Solyc02g077570  TTTTCTCATC GCGAATCAAC AGGAAATTTT TGTTGTGAAA CATGATTTTC 600
cDNA_Solyc02g077570  ---------- ---------- ---------- ---------- ---------- 280
       SIMLO15_LEAF  ---------- ---------- ---------- ---------- ---------- 283
       SIMLO15_ROOT  ---------- ---------- ---------- ---------- ---------- 526
     SIMLO15_FLOWER  ---------- ---------- ---------- ---------- ---------- 173
      SIMLO15_FRUIT  ---------- ---------- ---------- ---------- ---------- 152
```

```
                                        620                 640
gDNA_Solyc02g077570  CTACACTATT TCCACCGAAC TAGCTCACTT GAAAAACAAA TTCTTATTGA 650
cDNA_Solyc02g077570  ---------- ---------- ---------- ---------- ---------- 280
       SIMLO15_LEAF  ---------- ---------- ---------- ---------- ---------- 283
       SIMLO15_ROOT  ---------- ---------- ---------- ---------- ---------- 526
     SIMLO15_FLOWER  ---------- ---------- ---------- ---------- ---------- 173
      SIMLO15_FRUIT  ---------- ---------- ---------- ---------- ---------- 152
                              660                 680                 700
gDNA_Solyc02g077570  AATATTTGTG TAAACATTTT TCAAAGGGAA AATAGTGATT ATTTTAGTAA 700
cDNA_Solyc02g077570  ---------- ---------- ---------- ---------- ---------- 280
       SIMLO15_LEAF  ---------- ---------- ---------- ---------- ---------- 283
       SIMLO15_ROOT  ---------- ---------- ---------- ---------- ---------- 526
     SIMLO15_FLOWER  ---------- ---------- ---------- ---------- ---------- 173
      SIMLO15_FRUIT  ---------- ---------- ---------- ---------- ---------- 152
                                        720                 740
gDNA_Solyc02g077570  TATTTTATGT GTTTTCAGGG AAAGAAATCT TTAATGTCAA GAGCAGCTTC 750
cDNA_Solyc02g077570  ---------- ---------G AAAGAAATCT TTAATGTCAA GAGCAGCTTC 311
       SIMLO15_LEAF  ---------- ---------G AAAGAAATCT TTAATGTCAA GAGCAGCTTC 314
       SIMLO15_ROOT  ---------- ---------G AAAGAAATCT TTAATGTCAA GAGCAGCTTC 557
     SIMLO15_FLOWER  ---------- ---------G GAAGAAATCT TTAATGTCAA GAGCAGCTTC 204
      SIMLO15_FRUIT  ---------- ---------G AAAGAAATCT TTAATGTCAA GAGCAGCTTC 183
                              760                 780                 800
gDNA_Solyc02g077570  AGCTTTTAAT ATTTGCTCTT GCTTTCTTTC ATATTCTTTC TTGCATCCTC 800
cDNA_Solyc02g077570  AGCTTTTAAT ATTTGCTCTT GCTTTCTTTC ATATTCTTTC TTGCATCCTC 361
       SIMLO15_LEAF  AGCTTTTAAT ATTTGCTCTT GCTTTCTTTC ATATTCTTTC TTGCATCCTC 364
       SIMLO15_ROOT  AGCTTTTAAT ATTTGCTCTT GCTTTCTCTC ATATTCTTTC TCGCATCCTC 607
     SIMLO15_FLOWER  AGCTTTTAAT ATTTGCTCTT GCTTTCTTTC ATATTCTTTC TTGCATCCTC 254
      SIMLO15_FRUIT  AGCTTTTAAT ATTTGCTCTT GCTTTCTTTC ATATTCTTTC TTGCATCCTC 233
                                        820                 840
gDNA_Solyc02g077570  ACCTTCAGCC TTGGAACACG TAAGGTAATT AATTAAACTT TAGTTGTGTA 850
cDNA_Solyc02g077570  ACCTTCAGCC TTGGAACAC- ---------- ---------- ---------- 380
       SIMLO15_LEAF  ACCTTCAGCC TTGGAACAC- ---------- ---------- ---------- 383
       SIMLO15_ROOT  ACCTTCAGCC TTGGAACACG TAAG------ ---------- ---------- 631
     SIMLO15_FLOWER  ACCTTCAGCC TTGGAACACG TAAG------ ---------- ---------- 278
      SIMLO15_FRUIT  ACCTTCAGCC TTGGAACACG TAAG------ ---------- ---------- 257
                              860                 880                 900
gDNA_Solyc02g077570  GTAAAATCAC CTTTACTTAT TTTGTGATTG TTTAAAGTGA TTTTTCATTG 900
cDNA_Solyc02g077570  ---------- ---------- ---------- ---------- ---------- 380
       SIMLO15_LEAF  ---------- ---------- ---------- ---------- ---------- 383
       SIMLO15_ROOT  ---------- ---------- ---------- ---------- ---------- 631
     SIMLO15_FLOWER  ---------- ---------- ---------- ---------- ---------- 278
      SIMLO15_FRUIT  ---------- ---------- ---------- ---------- ---------- 257
                                        920                 940
gDNA_Solyc02g077570  CAAAAATTTC TAAAATACAC TACTACATAT ATTATCACCA TTTTGATAAC 950
cDNA_Solyc02g077570  ---------- ---------- ---------- ---------- ---------- 380
       SIMLO15_LEAF  ---------- ---------- ---------- ---------- ---------- 383
       SIMLO15_ROOT  ---------- ---------- ---------- ---------- ---------- 631
     SIMLO15_FLOWER  ---------- ---------- ---------- ---------- ---------- 278
      SIMLO15_FRUIT  ---------- ---------- ---------- ---------- ---------- 257
                              960                 980               1,000
gDNA_Solyc02g077570  TATGTAGAGA ATAATCAAAT CCCAAACCAA CAGTAACAAA GAAGTAGGGA 1000
cDNA_Solyc02g077570  ---------- ---------- ---------- ---------- ---------- 380
       SIMLO15_LEAF  ---------- ---------- ---------- ---------- ---------- 383
       SIMLO15_ROOT  ---------- ---------- ---------- ---------- ---------- 631
     SIMLO15_FLOWER  ---------- ---------- ---------- ---------- ---------- 278
      SIMLO15_FRUIT  ---------- ---------- ---------- ---------- ---------- 257
                                      1,020               1,040
gDNA_Solyc02g077570  AATCGAAATC GAAGTCTTAT ATTTGAAACA TTATTATTTT TGCTAGTGAT 1050
cDNA_Solyc02g077570  ---------- ---------- ---------- ---------- ---------- 380
       SIMLO15_LEAF  ---------- ---------- ---------- ---------- ---------- 383
       SIMLO15_ROOT  ---------- ---------- ---------- ---------- ---------- 631
     SIMLO15_FLOWER  ---------- ---------- ---------- ---------- ---------- 278
      SIMLO15_FRUIT  ---------- ---------- ---------- ---------- ---------- 257
                            1,060               1,080               1,100
gDNA_Solyc02g077570  TGTCAGTCTT GTAGACAATT TGTCAACTAT TGAATACGTA GCTTGAGTTG 1100
cDNA_Solyc02g077570  ---------- ---------- ---------- ---------- ---------- 380
       SIMLO15_LEAF  ---------- ---------- ---------- ---------- ---------- 383
       SIMLO15_ROOT  ---------- ---------- ---------- ---------- ---------- 631
     SIMLO15_FLOWER  ---------- ---------- ---------- ---------- ---------- 278
      SIMLO15_FRUIT  ---------- ---------- ---------- ---------- ---------- 257
                                      1,120               1,140
gDNA_Solyc02g077570  GACATTGATC ATATGTATGT TAATTTTTAC TAAATTTTTT TTGGTAAATG 1150
cDNA_Solyc02g077570  ---------- ---------- ---------- ---------- ---------- 380
       SIMLO15_LEAF  ---------- ---------- ---------- ---------- ---------- 383
       SIMLO15_ROOT  ---------- ---------- ---------- ---------- ---------- 631
     SIMLO15_FLOWER  ---------- ---------- ---------- ---------- ---------- 278
      SIMLO15_FRUIT  ---------- ---------- ---------- ---------- ---------- 257
                            1,160               1,180               1,200
gDNA_Solyc02g077570  GTCAGATGGA AATTTTGGGA GGCAGAAACA ACAACCTTAG ATTATCAATT 1200
cDNA_Solyc02g077570  -----ATGGA AATTTTGGGA GGCAGAAACA ACAACCTTAG ATTATCAATT 425
       SIMLO15_LEAF  -----ATGGA AATTTTGGGA GGCAGAAACA ACAACCTTAG ATTATCAATT 428
       SIMLO15_ROOT  -----ATGGA AATTTTGGGA GGCAGAAACA ACAACCTTAG ATTATCAATT 676
     SIMLO15_FLOWER  -----ATGGA AATTTTGGGA GGCAGAAACA ACAACCTTAG ATTATCAATT 323
      SIMLO15_FRUIT  -----ATGGA AATTTTGGGA GGCAGAAACA ACAACCTTAG ATTATCAATT 302
```

```
                                        1,220                        1,240
                                          |                            |
gDNA_Solyc02g077570  TTCACATGGT AAGCTGTCAC ATCCTCCTGT GGTAAATAAT TAATATTTTA 1250
cDNA_Solyc02g077570  TTCACAT--- ---------- ---------- ---------- ---------- 432
       SlMLO15_LEAF  TTCACAT--- ---------- ---------- ---------- ---------- 435
       SlMLO15_ROOT  TTCACAT--- ---------- ---------- ---------- ---------- 683
     SlMLO15_FLOWER  TTCACAT--- ---------- ---------- ---------- ---------- 330
      SlMLO15_FRUIT  TTCACAT--- ---------- ---------- ---------- ---------- 309
                              1,260                        1,280                        1,300
                                |                            |                            |
gDNA_Solyc02g077570  GGTTTGGACA AGGAAAACAC TTATTCTAAA AAATTAACAG GGGTTATGAA 1300
cDNA_Solyc02g077570  ---------- ---------- ---------- ---------- ---------- 432
       SlMLO15_LEAF  ---------- ---------- ---------- ---------- ---------- 435
       SlMLO15_ROOT  ---------- ---------- ---------- ---------- ---------- 683
     SlMLO15_FLOWER  ---------- ---------- ---------- ---------- ---------- 330
      SlMLO15_FRUIT  ---------- ---------- ---------- ---------- ---------- 309
                                        1,320                        1,340
                                          |                            |
gDNA_Solyc02g077570  ATGTGGCAAG GGCAATTTAG AAATAAAGAA TTAAATATCT TTTTTACGTA 1350
cDNA_Solyc02g077570  ---------- ---------- ---------- ---------- ---------- 432
       SlMLO15_LEAF  ---------- ---------- ---------- ---------- ---------- 435
       SlMLO15_ROOT  ---------- ---------- ---------- ---------- ---------- 683
     SlMLO15_FLOWER  ---------- ---------- ---------- ---------- ---------- 330
      SlMLO15_FRUIT  ---------- ---------- ---------- ---------- ---------- 309
                              1,360                        1,380                        1,400
                                |                            |                            |
gDNA_Solyc02g077570  AAATATTAAT TATTTAGAAT TATTCCTTTC ATTCCTATTT ACTTGTCGTT 1400
cDNA_Solyc02g077570  ---------- ---------- ---------- ---------- ---------- 432
       SlMLO15_LEAF  ---------- ---------- ---------- ---------- ---------- 435
       SlMLO15_ROOT  ---------- ---------- ---------- ---------- ---------- 683
     SlMLO15_FLOWER  ---------- ---------- ---------- ---------- ---------- 330
      SlMLO15_FRUIT  ---------- ---------- ---------- ---------- ---------- 309
                                        1,420                        1,440
                                          |                            |
gDNA_Solyc02g077570  TTTATCAAAA ATTGTCATTT CACATAATCA ATGCATAAAT GACATTAAGC 1450
cDNA_Solyc02g077570  ---------- ---------- ---------- ---------- ---------- 432
       SlMLO15_LEAF  ---------- ---------- ---------- ---------- ---------- 435
       SlMLO15_ROOT  ---------- ---------- ---------- ---------- ---------- 683
     SlMLO15_FLOWER  ---------- ---------- ---------- ---------- ---------- 330
      SlMLO15_FRUIT  ---------- ---------- ---------- ---------- ---------- 309
                              1,460                        1,480                        1,500
                                |                            |                            |
gDNA_Solyc02g077570  ATTCCATTTT ACCTATTAGC CTTGAAAGTA TATACCGTTG ACCAATATAA 1500
cDNA_Solyc02g077570  ---------- ---------- ---------- ---------- ---------- 432
       SlMLO15_LEAF  ---------- ---------- ---------- ---------- ---------- 435
       SlMLO15_ROOT  ---------- ---------- ---------- ---------- ---------- 683
     SlMLO15_FLOWER  ---------- ---------- ---------- ---------- ---------- 330
      SlMLO15_FRUIT  ---------- ---------- ---------- ---------- ---------- 309
                                        1,520                        1,540
                                          |                            |
gDNA_Solyc02g077570  AAAGACTTAT TTAGCAAGTA ATTCATGTTC ATTTGTTAAA TTAATTAATC 1550
cDNA_Solyc02g077570  ---------- ---------- ---------- ---------- ---------- 432
       SlMLO15_LEAF  ---------- ---------- ---------- ---------- ---------- 435
       SlMLO15_ROOT  ---------- ---------- ---------- ---------- ---------- 683
     SlMLO15_FLOWER  ---------- ---------- ---------- ---------- ---------- 330
      SlMLO15_FRUIT  ---------- ---------- ---------- ---------- ---------- 309
                              1,560                        1,580                        1,600
                                |                            |                            |
gDNA_Solyc02g077570  AAAGTAAATT AATTCATGTT TATTTATTGA AAACTTGAAT TCAAATGAAT 1600
cDNA_Solyc02g077570  ---------- ---------- ---------- ---------- ---------- 432
       SlMLO15_LEAF  ---------- ---------- ---------- ---------- ---------- 435
       SlMLO15_ROOT  ---------- ---------- ---------- ---------- ---------- 683
     SlMLO15_FLOWER  ---------- ---------- ---------- ---------- ---------- 330
      SlMLO15_FRUIT  ---------- ---------- ---------- ---------- ---------- 309
                                        1,620                        1,640
                                          |                            |
gDNA_Solyc02g077570  ACTACTAAAT AAGGATATAA TGATAAACTA ATATGGTTAC TTAATTGAGA 1650
cDNA_Solyc02g077570  ---------- ---------- ---------- ---------- ---------- 432
       SlMLO15_LEAF  ---------- ---------- ---------- ---------- ---------- 435
       SlMLO15_ROOT  ---------- ---------- ---------- ---------- ---------- 683
     SlMLO15_FLOWER  ---------- ---------- ---------- ---------- ---------- 330
      SlMLO15_FRUIT  ---------- ---------- ---------- ---------- ---------- 309
                              1,660                        1,680                        1,700
                                |                            |                            |
gDNA_Solyc02g077570  GGGGTGAAGC CTAAATTATT ATATATCAAG TATTAAAATC ATTATAATCT 1700
cDNA_Solyc02g077570  ---------- ---------- ---------- ---------- ---------- 432
       SlMLO15_LEAF  ---------- ---------- ---------- ---------- ---------- 435
       SlMLO15_ROOT  ---------- ---------- ---------- ---------- ---------- 683
     SlMLO15_FLOWER  ---------- ---------- ---------- ---------- ---------- 330
      SlMLO15_FRUIT  ---------- ---------- ---------- ---------- ---------- 309
                                        1,720                        1,740
                                          |                            |
gDNA_Solyc02g077570  CACAATCTTA GCAACATATT ATCTTGTTCA TAAATATAGA AAAAATAAGT 1750
cDNA_Solyc02g077570  ---------- ---------- ---------- ---------- ---------- 432
       SlMLO15_LEAF  ---------- ---------- ---------- ---------- ---------- 435
       SlMLO15_ROOT  ---------- ---------- ---------- ---------- ---------- 683
     SlMLO15_FLOWER  ---------- ---------- ---------- ---------- ---------- 330
      SlMLO15_FRUIT  ---------- ---------- ---------- ---------- ---------- 309
                              1,760                        1,780                        1,800
                                |                            |                            |
gDNA_Solyc02g077570  TTTTTTTCTA TTTATTTTTT TACGTAGGAT CTTCTCTTTT TTTCCGTTTA 1800
cDNA_Solyc02g077570  ---------- ---------- ---------- ---------- ---------- 432
       SlMLO15_LEAF  ---------- ---------- ---------- ---------- ---------- 435
       SlMLO15_ROOT  ---------- ---------- ---------- ---------- ---------- 683
     SlMLO15_FLOWER  ---------- ---------- ---------- ---------- ---------- 330
      SlMLO15_FRUIT  ---------- ---------- ---------- ---------- ---------- 309
```

```
                                                 1,820                         1,840
gDNA_Solyc02g077570  TATAATATTT TTATTGTAAT CTTTTGTTAA CAACATATTA ATCCGTGATT 1850
cDNA_Solyc02g077570  ---------- ---------- ---------- ---------- ---------- 432
        SlMLO15_LEAF ---------- ---------- ---------- ---------- ---------- 435
        SlMLO15_ROOT ---------- ---------- ---------- ---------- ---------- 683
      SlMLO15_FLOWER ---------- ---------- ---------- ---------- ---------- 330
       SlMLO15_FRUIT ---------- ---------- ---------- ---------- ---------- 309
                                1,860                         1,880                        1,900
gDNA_Solyc02g077570  AATAAATTAC CTAGAGATAA TCAAACTATT TGAACAAACT AATTTCAATT 1900
cDNA_Solyc02g077570  ---------- ---------- ---------- ---------- ---------- 432
        SlMLO15_LEAF ---------- ---------- ---------- ---------- ---------- 435
        SlMLO15_ROOT ---------- ---------- ---------- ---------- ---------- 683
      SlMLO15_FLOWER ---------- ---------- ---------- ---------- ---------- 330
       SlMLO15_FRUIT ---------- ---------- ---------- ---------- ---------- 309
                                                 1,920                         1,940
gDNA_Solyc02g077570  TTATAATAAG ATTACGAATA TGGTGATACC AATACATACG GTAGATTAAT 1950
cDNA_Solyc02g077570  ---------- ---------- ---------- ---------- ---------- 432
        SlMLO15_LEAF ---------- ---------- ---------- ---------- ---------- 435
        SlMLO15_ROOT ---------- ---------- ---------- ---------- ---------- 683
      SlMLO15_FLOWER ---------- ---------- ---------- ---------- ---------- 330
       SlMLO15_FRUIT ---------- ---------- ---------- ---------- ---------- 309
                                1,960                         1,980                        2,000
gDNA_Solyc02g077570  TATAGTTTCT ATACTTCTAT TCAGTTAATT ATTCTTTTAA ATTAGATAAA 2000
cDNA_Solyc02g077570  ---------- ---------- ---------- ---------- ---------- 432
        SlMLO15_LEAF ---------- ---------- ---------- ---------- ---------- 435
        SlMLO15_ROOT ---------- ---------- ---------- ---------- ---------- 683
      SlMLO15_FLOWER ---------- ---------- ---------- ---------- ---------- 330
       SlMLO15_FRUIT ---------- ---------- ---------- ---------- ---------- 309
                                                 2,020                         2,040
gDNA_Solyc02g077570  AATGAGTTAA TTATTCTTTT AAATTAGATA AAAAAAATTA TTTAAAAGAA 2050
cDNA_Solyc02g077570  ---------- ---------- ---------- ---------- ---------- 432
        SlMLO15_LEAF ---------- ---------- ---------- ---------- ---------- 435
        SlMLO15_ROOT ---------- ---------- ---------- ---------- ---------- 683
      SlMLO15_FLOWER ---------- ---------- ---------- ---------- ---------- 330
       SlMLO15_FRUIT ---------- ---------- ---------- ---------- ---------- 309
                                2,060                         2,080                        2,100
gDNA_Solyc02g077570  AATTAAAAAT AACCTTACGT ATAAAAGAAA TTTAGAAAGA GATTTTCCCC 2100
cDNA_Solyc02g077570  ---------- ---------- ---------- ---------- ---------- 432
        SlMLO15_LEAF ---------- ---------- ---------- ---------- ---------- 435
        SlMLO15_ROOT ---------- ---------- ---------- ---------- ---------- 683
      SlMLO15_FLOWER ---------- ---------- ---------- ---------- ---------- 330
       SlMLO15_FRUIT ---------- ---------- ---------- ---------- ---------- 309
                                                 2,120                         2,140
gDNA_Solyc02g077570  CCTATATTTA TGGACAAGAT AATATGTTGC TAAGAAGGTA ATATTAAAAT 2150
cDNA_Solyc02g077570  ---------- ---------- ---------- ---------- ---------- 432
        SlMLO15_LEAF ---------- ---------- ---------- ---------- ---------- 435
        SlMLO15_ROOT ---------- ---------- ---------- ---------- ---------- 683
      SlMLO15_FLOWER ---------- ---------- ---------- ---------- ---------- 330
       SlMLO15_FRUIT ---------- ---------- ---------- ---------- ---------- 309
                                2,160                         2,180                        2,200
gDNA_Solyc02g077570  AATTTTTAAT ATTTAATATA TAATGTATTT ATTAATTAAA TCATGGTTAG 2200
cDNA_Solyc02g077570  ---------- ---------- ---------- ---------- ---------- 432
        SlMLO15_LEAF ---------- ---------- ---------- ---------- ---------- 435
        SlMLO15_ROOT ---------- ---------- ---------- ---------- ---------- 683
      SlMLO15_FLOWER ---------- ---------- ---------- ---------- ---------- 330
       SlMLO15_FRUIT ---------- ---------- ---------- ---------- ---------- 309
                                                 2,220                         2,240
gDNA_Solyc02g077570  AGATTTACTT TGAACATTAA ATCTTAACTA TTTATTTTAA CTTATGCCAT 2250
cDNA_Solyc02g077570  ---------- ---------- ---------- ---------- ---------- 432
        SlMLO15_LEAF ---------- ---------- ---------- ---------- ---------- 435
        SlMLO15_ROOT ---------- ---------- ---------- ---------- ---------- 683
      SlMLO15_FLOWER ---------- ---------- ---------- ---------- ---------- 330
       SlMLO15_FRUIT ---------- ---------- ---------- ---------- ---------- 309
                                2,260                         2,280                        2,300
gDNA_Solyc02g077570  GTGTCATTAA TCCAAATAGG AACTTAGAGA AAAAAATAGA GAGGAATCAA 2300
cDNA_Solyc02g077570  ---------- ---------- ---------- ---------- ---------- 432
        SlMLO15_LEAF ---------- ---------- ---------- ---------- ---------- 435
        SlMLO15_ROOT ---------- ---------- ---------- ---------- ---------- 683
      SlMLO15_FLOWER ---------- ---------- ---------- ---------- ---------- 330
       SlMLO15_FRUIT ---------- ---------- ---------- ---------- ---------- 309
                                                 2,320                         2,340
gDNA_Solyc02g077570  ATCCATTTAT AAGTGATTAG AAATAATTAA AAAAGAAAAA GTACACGAAT 2350
cDNA_Solyc02g077570  ---------- ---------- ---------- ---------- ---------- 432
        SlMLO15_LEAF ---------- ---------- ---------- ---------- ---------- 435
        SlMLO15_ROOT ---------- ---------- ---------- ---------- ---------- 683
      SlMLO15_FLOWER ---------- ---------- ---------- ---------- ---------- 330
       SlMLO15_FRUIT ---------- ---------- ---------- ---------- ---------- 309
                                2,360                         2,380                        2,400
gDNA_Solyc02g077570  AATTAGAAAC CGGAGAGTAA CAACTAAACA TCAATTATTC ATGTCGTCTA 2400
cDNA_Solyc02g077570  ---------- ---------- ---------- ---------- ---------- 432
        SlMLO15_LEAF ---------- ---------- ---------- ---------- ---------- 435
        SlMLO15_ROOT ---------- ---------- ---------- ---------- ---------- 683
      SlMLO15_FLOWER ---------- ---------- ---------- ---------- ---------- 330
       SlMLO15_FRUIT ---------- ---------- ---------- ---------- ---------- 309
```

```
                                              2,420                          2,440
gDNA_Solyc02g077570  TCTATACTGT AGGGACATTT ATTTCTTGTC TCAACTAAAC ACATTATTAT 2450
cDNA_Solyc02g077570  ---------- ---------- ---------- ---------- ---------- 432
      SlMLO15_LEAF  ---------- ---------- ---------- ---------- ---------- 435
      SlMLO15_ROOT  ---------- ---------- ---------- ---------- ---------- 683
    SlMLO15_FLOWER  ---------- ---------- ---------- ---------- ---------- 330
     SlMLO15_FRUIT  ---------- ---------- ---------- ---------- ---------- 309
                               2,460                          2,480                      2,500
gDNA_Solyc02g077570  ATGAACAATT ATTATGTCCC AAGATATAGG CTTAATAAGG AAAATGTTTC 2500
cDNA_Solyc02g077570  ---------- ---------- ---------- ---------- ---------- 432
      SlMLO15_LEAF  ---------- ---------- ---------- ---------- ---------- 435
      SlMLO15_ROOT  ---------- ---------- ---------- ---------- ---------- 683
    SlMLO15_FLOWER  ---------- ---------- ---------- ---------- ---------- 330
     SlMLO15_FRUIT  ---------- ---------- ---------- ---------- ---------- 309
                                              2,520                          2,540
gDNA_Solyc02g077570  ATGAAAAAAA AAGATGCAAG TTAGCTGTAC CATAAACGAA TATTTCTTTT 2550
cDNA_Solyc02g077570  ---------- ---------- ---------- ---------- ---------- 432
      SlMLO15_LEAF  ---------- ---------- ---------- ---------- ---------- 435
      SlMLO15_ROOT  ---------- ---------- ---------- ---------- ---------- 683
    SlMLO15_FLOWER  ---------- ---------- ---------- ---------- ---------- 330
     SlMLO15_FRUIT  ---------- ---------- ---------- ---------- ---------- 309
                               2,560                          2,580                      2,600
gDNA_Solyc02g077570  TATATGTCCA CGTTTATTTG ATATTAATTT TTATCATATT GATATGCAAA 2600
cDNA_Solyc02g077570  ---------- ---------- ---------- ---------- ---------- 432
      SlMLO15_LEAF  ---------- ---------- ---------- ---------- ---------- 435
      SlMLO15_ROOT  ---------- ---------- ---------- ---------- ---------- 683
    SlMLO15_FLOWER  ---------- ---------- ---------- ---------- ---------- 330
     SlMLO15_FRUIT  ---------- ---------- ---------- ---------- ---------- 309
                                              2,620                          2,640
gDNA_Solyc02g077570  AAATTATAGT TATAGTACTT TTTGTATATA GTTTTTAAAT GTCTAAATTT 2650
cDNA_Solyc02g077570  ---------- ---------- ---------- ---------- ---------- 432
      SlMLO15_LEAF  ---------- ---------- ---------- ---------- ---------- 435
      SlMLO15_ROOT  ---------- ---------- ---------- ---------- ---------- 683
    SlMLO15_FLOWER  ---------- ---------- ---------- ---------- ---------- 330
     SlMLO15_FRUIT  ---------- ---------- ---------- ---------- ---------- 309
                               2,660                          2,680                      2,700
gDNA_Solyc02g077570  TTTGTTTAAA ATATCGAATT AATGTAATTT AATTTAACTT TGAAAATTAC 2700
cDNA_Solyc02g077570  ---------- ---------- ---------- ---------- ---------- 432
      SlMLO15_LEAF  ---------- ---------- ---------- ---------- ---------- 435
      SlMLO15_ROOT  ---------- ---------- ---------- ---------- ---------- 683
    SlMLO15_FLOWER  ---------- ---------- ---------- ---------- ---------- 330
     SlMLO15_FRUIT  ---------- ---------- ---------- ---------- ---------- 309
                                              2,720                          2,740
gDNA_Solyc02g077570  TTAACTATTT AACTTTCAAA AAGTACAACA TGACAATTAA GAACGGACAG 2750
cDNA_Solyc02g077570  ---------- ---------- ---------- ---------- ---------- 432
      SlMLO15_LEAF  ---------- ---------- ---------- ---------- ---------- 435
      SlMLO15_ROOT  ---------- ---------- ---------- ---------- ---------- 683
    SlMLO15_FLOWER  ---------- ---------- ---------- ---------- ---------- 330
     SlMLO15_FRUIT  ---------- ---------- ---------- ---------- ---------- 309
                               2,760                          2,780                      2,800
gDNA_Solyc02g077570  AAAGGGTTTT ACTAAACAAA TTTTAATCTA TTATAGTATG TTTCTTCATA 2800
cDNA_Solyc02g077570  ---------- ---------- ---------- ---------- ---------- 432
      SlMLO15_LEAF  ---------- ---------- ---------- ---------- ---------- 435
      SlMLO15_ROOT  ---------- ---------- ---------- ---------- ---------- 683
    SlMLO15_FLOWER  ---------- ---------- ---------- ---------- ---------- 330
     SlMLO15_FRUIT  ---------- ---------- ---------- ---------- ---------- 309
                                              2,820                          2,840
gDNA_Solyc02g077570  ATTTTTTTAA TCGTACATAT TATATAGAGA GTAGACTTAT TTATAAGGTA 2850
cDNA_Solyc02g077570  ---------- ---------- ---------- ---------- ---------- 432
      SlMLO15_LEAF  ---------- ---------- ---------- ---------- ---------- 435
      SlMLO15_ROOT  ---------- ---------- ---------- ---------- ---------- 683
    SlMLO15_FLOWER  ---------- ---------- ---------- ---------- ---------- 330
     SlMLO15_FRUIT  ---------- ---------- ---------- ---------- ---------- 309
                               2,860                          2,880                      2,900
gDNA_Solyc02g077570  ATTTGTAATT AAATTTCAAT GATATGATTA TCTAAATATG TACATAGAAA 2900
cDNA_Solyc02g077570  ---------- ---------- ---------- ---------- ---------- 432
      SlMLO15_LEAF  ---------- ---------- ---------- ---------- ---------- 435
      SlMLO15_ROOT  ---------- ---------- ---------- ---------- ---------- 683
    SlMLO15_FLOWER  ---------- ---------- ---------- ---------- ---------- 330
     SlMLO15_FRUIT  ---------- ---------- ---------- ---------- ---------- 309
                                              2,920                          2,940
gDNA_Solyc02g077570  TTTAACACTC TGAACTTTCA ACATATATAA TAATGACAAT TTCAAACAAA 2950
cDNA_Solyc02g077570  ---------- ---------- ---------- ---------- ---------- 432
      SlMLO15_LEAF  ---------- ---------- ---------- ---------- ---------- 435
      SlMLO15_ROOT  ---------- ---------- ---------- ---------- ---------- 683
    SlMLO15_FLOWER  ---------- ---------- ---------- ---------- ---------- 330
     SlMLO15_FRUIT  ---------- ---------- ---------- ---------- ---------- 309
                               2,960                          2,980                      3,000
gDNA_Solyc02g077570  GACGTGAGGG TGTTTTTTTT CTTCTTTCAT GTTTGGTTTA GTTCGTTTAA 3000
cDNA_Solyc02g077570  ---------- ---------- ---------- ---------- ---------- 432
      SlMLO15_LEAF  ---------- ---------- ---------- ---------- ---------- 435
      SlMLO15_ROOT  ---------- ---------- ---------- ---------- ---------- 683
    SlMLO15_FLOWER  ---------- ---------- ---------- ---------- ---------- 330
     SlMLO15_FRUIT  ---------- ---------- ---------- ---------- ---------- 309
```

```
                                       3,020                         3,040
gDNA_Solyc02g077570  ACATTTCTTT TCATTTTGAT TATTTTTTTA ATTTCAACTT TTTATATATG 3050
cDNA_Solyc02g077570  ---------- ---------- ---------- ---------- ---------- 432
       SIMLO15_LEAF  ---------- ---------- ---------- ---------- ---------- 435
       SIMLO15_ROOT  ---------- ---------- ---------- ---------- ---------- 683
     SIMLO15_FLOWER  ---------- ---------- ---------- ---------- ---------- 330
      SIMLO15_FRUIT  ---------- ---------- ---------- ---------- ---------- 309
                            3,060                         3,080                         3,100
gDNA_Solyc02g077570  TTTAGGATTT TAGCATATTT GAATATATCT TTAATTTATG ATTATAAATT 3100
cDNA_Solyc02g077570  ---------- ---------- ---------- ---------- ---------- 432
       SIMLO15_LEAF  ---------- ---------- ---------- ---------- ---------- 435
       SIMLO15_ROOT  ---------- ---------- ---------- ---------- ---------- 683
     SIMLO15_FLOWER  ---------- ---------- ---------- ---------- ---------- 330
      SIMLO15_FRUIT  ---------- ---------- ---------- ---------- ---------- 309
                                       3,120                         3,140
gDNA_Solyc02g077570  CGAAAAATCT CTTTTCTTTC ATAAACTCCA TATCAAAATC ATAATATACA 3150
cDNA_Solyc02g077570  ---------- ---------- ---------- ---------- ---------- 432
       SIMLO15_LEAF  ---------- ---------- ---------- ---------- ---------- 435
       SIMLO15_ROOT  ---------- ---------- ---------- ---------- ---------- 683
     SIMLO15_FLOWER  ---------- ---------- ---------- ---------- ---------- 330
      SIMLO15_FRUIT  ---------- ---------- ---------- ---------- ---------- 309
                            3,160                         3,180                         3,200
gDNA_Solyc02g077570  AATTGAAACA AAAGAAATCA CAATTTTCTA TATTGATAAT AAACAAAATC 3200
cDNA_Solyc02g077570  ---------- ---------- ---------- ---------- ---------- 432
       SIMLO15_LEAF  ---------- ---------- ---------- ---------- ---------- 435
       SIMLO15_ROOT  ---------- ---------- ---------- ---------- ---------- 683
     SIMLO15_FLOWER  ---------- ---------- ---------- ---------- ---------- 330
      SIMLO15_FRUIT  ---------- ---------- ---------- ---------- ---------- 309
                                       3,220                         3,240
gDNA_Solyc02g077570  TTCCACTTTT TAATTGGTGT CTAATTATTT AATGTTTTTT TATAGATCCA 3250
cDNA_Solyc02g077570  ---------- ---------- ---------- ---------- ----GATCCA 438
       SIMLO15_LEAF  ---------- ---------- ---------- ---------- ----GATCCA 441
       SIMLO15_ROOT  ---------- ---------- ---------- ---------- ----GATCCA 689
     SIMLO15_FLOWER  ---------- ---------- ---------- ---------- ----GATCCA 336
      SIMLO15_FRUIT  ---------- ---------- ---------- ---------- ----GATCCA 315
                            3,260                         3,280                         3,300
gDNA_Solyc02g077570  AGGAGGTTTC AACTAATTCA TCAAACATCA TTCGGAAAGA GGCATCTCAA 3300
cDNA_Solyc02g077570  AGGAGGTTTC AACTAATTCA TCAAACATCA TTCGGAAAGA GGCATCTCAA 488
       SIMLO15_LEAF  AGGAGGTTTC AACTAATTCA TCAAACATCA TTCGGAAAGA GGCATCTCAA 491
       SIMLO15_ROOT  AGGAGGTTTC AACTAATTCA TCAAACATCA TTCGGAAAGA GGCATCTCAA 739
     SIMLO15_FLOWER  AGGAGGTTTC AACTAATTCA TCAAACATCA TTCGGAAAGA GGCATCTCAA 386
      SIMLO15_FRUIT  AGGAGGTTTC AACTAATTCA TCAAACATCA TTCGGAAAGA GGCATCTCAA 365
                                       3,320                         3,340
gDNA_Solyc02g077570  TTTTTGGAGT GAACACCGCT TTCTACGTTT CCCAGTACGT AACTAACTTA 3350
cDNA_Solyc02g077570  TTTTTGGAGT GAACACCGCT TTCTACGTTT CCCAGT---- ---------- 524
       SIMLO15_LEAF  TTTTTGGAGT GAACACCGCT TTCTACGTTT CCCAGT---- ---------- 527
       SIMLO15_ROOT  TTTTTGGAGT GAACACCGCT TTCTACGTTT CCCAGT---- ---------- 775
     SIMLO15_FLOWER  TTTTTGGAGT GAACACCGCT TTCTACGTTT CCCAGT---- ---------- 422
      SIMLO15_FRUIT  TTTTTGGAGT GAACACCGCT TTCTACGTTT CCCANN---- ---------- 401
                            3,360                         3,380                         3,400
gDNA_Solyc02g077570  ATTTTGTCTG CTCTGATATT ATGTTAAAGT GTGTAATTAT CTTATTTAAA 3400
cDNA_Solyc02g077570  ---------- ---------- ---------- ---------- ---------- 524
       SIMLO15_LEAF  ---------- ---------- ---------- ---------- ---------- 527
       SIMLO15_ROOT  ---------- ---------- ---------- ---------- ---------- 775
     SIMLO15_FLOWER  ---------- ---------- ---------- ---------- ---------- 422
      SIMLO15_FRUIT  ---------- ---------- ---------- ---------- ---------- 401
                                       3,420                         3,440
gDNA_Solyc02g077570  AATTTAAATA TTTAAAAATA ACATTTTAAT TATTTAATTA TATTTTCAAC 3450
cDNA_Solyc02g077570  ---------- ---------- ---------- ---------- ---------- 524
       SIMLO15_LEAF  ---------- ---------- ---------- ---------- ---------- 527
       SIMLO15_ROOT  ---------- ---------- ---------- ---------- ---------- 775
     SIMLO15_FLOWER  ---------- ---------- ---------- ---------- ---------- 422
      SIMLO15_FRUIT  ---------- ---------- ---------- ---------- ---------- 401
                            3,460                         3,480                         3,500
gDNA_Solyc02g077570  CGTTCTTTTT ACTTTTTTGT GCTAGCCAGG TTTGTTTGTT GAGGCAATTT 3500
cDNA_Solyc02g077570  ---------- ---------- ---------- TT-GTTTGTT GAGGCAATTT 543
       SIMLO15_LEAF  ---------- ---------- ---------- TT-GTTTGTT GAGGCAATTT 546
       SIMLO15_ROOT  ---------- ---------- ---------- TTTGTTTGTT GAGGCAATTT 795
     SIMLO15_FLOWER  ---------- ---------- ---------- TT-GTTTGTT GAGGCAATTT 441
      SIMLO15_FRUIT  ---------- ---------- ---------- TTTGTTTGTT GAGGCAANTT 421
                                       3,520                         3,540
gDNA_Solyc02g077570  TATGGATCAG TATATAAAGT AGACTACTTA ACTCTCCGGC ATGGATTCAT 3550
cDNA_Solyc02g077570  TATGGATCAG TATATAAAGT AGACTACTTA ACTCTCCGGC ATGGATTCAT 593
       SIMLO15_LEAF  TATGGATCAG TATATAAAGT AGACTACTTA ACTCTCCGGC ATGGATTCAT 596
       SIMLO15_ROOT  TATGGATCAG TATATAAAGT AGACTACTTA ACTCTCCGGC ATGGATTCAT 845
     SIMLO15_FLOWER  TATGGATCAG TATATAAAGT AGACTACTTA ACTCTCCGGC ATGGATTCAT 491
      SIMLO15_FRUIT  TATGGATCAG TATATAAAGT AGACTACTTA ACTCTCCGGC ATG-ATTCAT 470
                            3,560                         3,580                         3,600
gDNA_Solyc02g077570  AATGGTATTT ACAAAACCTT TTTTTTCTTT TTTAATGTAT GATGAATTAA 3600
cDNA_Solyc02g077570  AATGG----- ---------- ---------- ---------- ---------- 598
       SIMLO15_LEAF  AATGG----- ---------- ---------- ---------- ---------- 601
       SIMLO15_ROOT  AATGG----- ---------- ---------- ---------- ---------- 850
     SIMLO15_FLOWER  AATGG----- ---------- ---------- ---------- ---------- 496
      SIMLO15_FRUIT  AATGG----- ---------- ---------- ---------- ---------- 475
```

```
                                      3,620                         3,640
gDNA_Solyc02g077570  TTACAATATG TGAGATTAAG GCATATACTT ACTTCTCTTT AATTTTTATT 3650
cDNA_Solyc02g077570  ---------- ---------- ---------- ---------- ---------- 598
       SlMLO15_LEAF  ---------- ---------- ---------- ---------- ---------- 601
       SlMLO15_ROOT  ---------- ---------- ---------- ---------- ---------- 850
     SlMLO15_FLOWER  ---------- ---------- ---------- ---------- ---------- 496
      SlMLO15_FRUIT  ---------- ---------- ---------- ---------- ---------- 475

                           3,660                         3,680                   3,700
gDNA_Solyc02g077570  TATTAGGCAC ATTTTGCTGA AGGAACAGAG TTTGATTTTC -ACAAGTATA 3699
cDNA_Solyc02g077570  -------CAC ATTTTGCTGA AGGAACAGAG TTTGATTTTC -ACAAGTATA 640
       SlMLO15_LEAF  -------CAC ATTTTGCTGA AGGAACAGAG TTTGATTTTC -ACAAGTATA 643
       SlMLO15_ROOT  -------CAC ATTTTGCTGA AGGAACAGAG TTTGATTTTC -ACAAGTATA 892
     SlMLO15_FLOWER  -------CAC CTTTTGCTGA AGGAACAGAG TTGTTTTTTC -ACAAGTATA 538
      SlMLO15_FRUIT  -------CAC ATTTTGCTGA AGGAACAGAT TTTAATTTTC CACAAGTATA 518

                                      3,720                         3,740
gDNA_Solyc02g077570  TAAGAAGAGC TTTAGATAAA G-ATTTTCAA GTTGTCGTGG CAATTAGGTA 3748
cDNA_Solyc02g077570  TAAGAAGAGC TTTAGATAAA G-ATTTTCAA GTTGTCGTGG CAATTAG--- 686
       SlMLO15_LEAF  TAAGAAGAGC TTTAGATAAA G-ATTTTCAA GTTGTCGTGG CAATTAG--- 689
       SlMLO15_ROOT  TAAGAAGAGC TTTAGATAAA G-ATTTTCAA GTTGTCGTGG CAATTAG--- 938
     SlMLO15_FLOWER  TAAGAAGAGC TTTAGATAAA G-ATTTTCAA GTTGTCGTGG CAATTAG--- 584
      SlMLO15_FRUIT  TAAGAAGAGC TTTAGATAAA GGATTTCCAA GTTGTTGGGG CAATTGA--- 565

                           3,760                         3,780                   3,800
gDNA_Solyc02g077570  ACGTTATTAT ATATGTGGAA AAATGCATTT TTCATAATTT AATTAAGTAG 3798
cDNA_Solyc02g077570  ---------- ---------- ---------- ---------- ---------- 686
       SlMLO15_LEAF  ---------- ---------- ---------- ---------- ---------- 689
       SlMLO15_ROOT  ---------- ---------- ---------- ---------- ---------- 938
     SlMLO15_FLOWER  ---------- ---------- ---------- ---------- ---------- 584
      SlMLO15_FRUIT  ---------- ---------- ---------- ---------- ---------- 565

                                      3,820                         3,840
gDNA_Solyc02g077570  CTAATTACAT TTTTTTTGTT GTTGTTGATG CAGCCCATGG ATTTGGACCT 3848
cDNA_Solyc02g077570  ---------- ---------- ---------- ---CCCATGG ATTTGGACCT 703
       SlMLO15_LEAF  ---------- ---------- ---------- ---CCCATGG ATTTGGACCT 706
       SlMLO15_ROOT  ---------- ---------- ---------- ---CCCATGG ATTTGGACCT 955
     SlMLO15_FLOWER  ---------- ---------- ---------- ---CCCATGG ATTTTGACCT 601
      SlMLO15_FRUIT  ---------- ---------- ---------- ---CCCATGG ATTAGGACCT 582

                           3,860                         3,880                   3,900
gDNA_Solyc02g077570  TTTCTATGCT CTTCATATTC TTCAATGCAA ATGGTAAATA TTGTAACAAC 3898
cDNA_Solyc02g077570  TTTCTATGCT CTTCATATTC TTCAATGCAA ATG------- -TGT------ 739
       SlMLO15_LEAF  TTTCTATGCT CTTCATATTC TTCAATGCAA ATG------- -TGT------ 742
       SlMLO15_ROOT  TTTCTATGCT CTTCATATTC TTCAATGCAA ATG------- -TGT------ 991
     SlMLO15_FLOWER  TTTCTATGCT CTTCATATTC TTCAATGCAA ATG------- -TGT------ 637
      SlMLO15_FRUIT  TTTTTATGCT ATTCATATTC TTCAAAGCAA AAA------- -GGT------ 618

                                      3,920                         3,940
gDNA_Solyc02g077570  TGTCACAAAA TTGTCACCAA AATTTATTAG TATATTATTA ATTTTCATTT 3948
cDNA_Solyc02g077570  ---------- ---------- ---------- ---------- ---------- 739
       SlMLO15_LEAF  ---------- ---------- ---------- ---------- ---------- 742
       SlMLO15_ROOT  ---------- ---------- ---------- ---------- ---------- 991
     SlMLO15_FLOWER  ---------- ---------- ---------- ---------- ---------- 637
      SlMLO15_FRUIT  ---------- ---------- ---------- ---------- ---------- 618

                           3,960                         3,980                   4,000
gDNA_Solyc02g077570  GTTTATTTTT GTAGTGTTCC ATAGCACCTA TTGGCTTCCT T-TCATTCCT 3997
cDNA_Solyc02g077570  ---------- ------T-CC ATAGCACCTA TTGGCTTCCT T-TCATTCCT 771
       SlMLO15_LEAF  ---------- ------T-CC ATAGCACCTA TTGGCTTCCT T-TCATTCCT 774
       SlMLO15_ROOT  ---------- ------T-CC ATAGCACCTA TTGGCTTCCT T-TCATTCCT 1023
     SlMLO15_FLOWER  ---------- ------T-CC ATAGCACCTA TTGGCTTCCT T-TCATTCCT 669
      SlMLO15_FRUIT  ---------- ------TTCC ATAGCACGTA TAGGCTTTCA TATCATTCCT 652

                                      4,020                         4,040
gDNA_Solyc02g077570  -CTAGCGGTA AGCAAATTTT ATTCAAAGGA AAAAAAAATA TTTATAAATG 4046
cDNA_Solyc02g077570  -CTAGCG--- ---------- ---------- ---------- ---------- 777
       SlMLO15_LEAF  -CTAGCG--- ---------- ---------- ---------- ---------- 780
       SlMLO15_ROOT  -CTAGCG--- ---------- ---------- ---------- ---------- 1029
     SlMLO15_FLOWER  -CTAGCG--- ---------- ---------- ---------- ---------- 675
      SlMLO15_FRUIT  TCTAGAG--- ---------- ---------- ---------- ---------- 659

                           4,060                         4,080                   4,100
gDNA_Solyc02g077570  AAAATTATCA GTGTCGAGAT CTTAGTAAGC TCAATTGATT GACTATCTGA 4096
cDNA_Solyc02g077570  ---------- ---------- ---------- ---------- ---------- 777
       SlMLO15_LEAF  ---------- ---------- ---------- ---------- ---------- 780
       SlMLO15_ROOT  ---------- ---------- ---------- ---------- ---------- 1029
     SlMLO15_FLOWER  ---------- ---------- ---------- ---------- ---------- 675
      SlMLO15_FRUIT  ---------- ---------- ---------- ---------- ---------- 659

                                      4,120                         4,140
gDNA_Solyc02g077570  ATTTCACATT ATTTAAAGAT TTGACTCTCC ACCTTGTAAT TTGCTTCCCT 4146
cDNA_Solyc02g077570  ---------- ---------- ---------- ---------- ---------- 777
       SlMLO15_LEAF  ---------- ---------- ---------- ---------- ---------- 780
       SlMLO15_ROOT  ---------- ---------- ---------- ---------- ---------- 1029
     SlMLO15_FLOWER  ---------- ---------- ---------- ---------- ---------- 675
      SlMLO15_FRUIT  ---------- ---------- ---------- ---------- ---------- 659

                           4,160                         4,180                   4,200
gDNA_Solyc02g077570  AATAAATACC CTTCCGATCC CTACTTACCC TTTATGTAAT TAAAAATTAG 4196
cDNA_Solyc02g077570  ---------- ---------- ---------- ---------- ---------- 777
       SlMLO15_LEAF  ---------- ---------- ---------- ---------- ---------- 780
       SlMLO15_ROOT  ---------- ---------- ---------- ---------- ---------- 1029
     SlMLO15_FLOWER  ---------- ---------- ---------- ---------- ---------- 675
      SlMLO15_FRUIT  ---------- ---------- ---------- ---------- ---------- 659
```

```
                                      4,220                         4,240
gDNA_Solyc02g077570  TGATTAGATA TATATAGGGA TAAGGCATTA GTACAAGTAA ACTATGATTG 4246
cDNA_Solyc02g077570  ---------- ---------- ---------- ---------- ---------- 777
       SlMLO15_LEAF  ---------- ---------- ---------- ---------- ---------- 780
       SlMLO15_ROOT  ---------- ---------- ---------- ---------- ---------- 1029
     SlMLO15_FLOWER  ---------- ---------- ---------- ---------- ---------- 675
      SlMLO15_FRUIT  ---------- ---------- ---------- ---------- ---------- 659

                          4,260                         4,280                     4,300
gDNA_Solyc02g077570  ATATCTTAAC TTAGTTCGGA TTCTATTATC TTTTGAACTC AATTTTTCTA 4296
cDNA_Solyc02g077570  ---------- ---------- ---------- ---------- ---------- 777
       SlMLO15_LEAF  ---------- ---------- ---------- ---------- ---------- 780
       SlMLO15_ROOT  ---------- ---------- ---------- ---------- ---------- 1029
     SlMLO15_FLOWER  ---------- ---------- ---------- ---------- ---------- 675
      SlMLO15_FRUIT  ---------- ---------- ---------- ---------- ---------- 659

                                      4,320                         4,340
gDNA_Solyc02g077570  TATTTGTATA CACCTTTTGT GCTGACGTGA CATATTTAAT CAGATAAAGT 4346
cDNA_Solyc02g077570  ---------- ---------- ---------- ---------- ---------- 777
       SlMLO15_LEAF  ---------- ---------- ---------- ---------- ---------- 780
       SlMLO15_ROOT  ---------- ---------- ---------- ---------- ---------- 1029
     SlMLO15_FLOWER  ---------- ---------- ---------- ---------- ---------- 675
      SlMLO15_FRUIT  ---------- ---------- ---------- ---------- ---------- 659

                          4,360                         4,380                     4,400
gDNA_Solyc02g077570  TGGCTTATCC CTTAACATGT ATTGTCTTTT TCCTATGAAA TAAACACAGA 4396
cDNA_Solyc02g077570  ---------- ---------- ---------- ---------- ---------A 778
       SlMLO15_LEAF  ---------- ---------- ---------- ---------- ---------A 781
       SlMLO15_ROOT  ---------- ---------- ---------- ---------- ---------A 1030
     SlMLO15_FLOWER  ---------- ---------- ---------- ---------- ---------A 676
      SlMLO15_FRUIT  ---------- ---------- ---------- ---------- ---------A 660

                                      4,420                         4,440
gDNA_Solyc02g077570  TGTTAGTGGC TGTTGGAACA AAATTACAAG GCATAATCAC AAAAATGTGT 4446
cDNA_Solyc02g077570  TGTTAGTGGC TGTTGGAACA AAATTACAAG GCATAATCAC AAAAATGTGT 828
       SlMLO15_LEAF  TGTTAGTGGC TGTTGGAACA AAATTACAAG GCATAATCAC AAAAATGTGT 831
       SlMLO15_ROOT  TGTTAGTGGC CGTTGGAACA AAATTACAAG GCATAATCAC AAAAATGTGT 1080
     SlMLO15_FLOWER  TGTTAGTGGC TGTTGGAACA AAATTACAAG GCATAATCAC AAAA-TGTGT 725
      SlMLO15_FRUIT  TGTTAGTGGC TTTTGGAACA AAATTACAAG GCATAATCAC AAAA-TGTGT 709

                          4,460                         4,480                     4,500
gDNA_Solyc02g077570  TTGGATAGCA ATTATAAGTC AAGTGTTATT AGAGGAAATT TAGTGGTTAA 4496
cDNA_Solyc02g077570  TTGGATAGCA ATTATAAGTC AAGTGTTATT AGAGGAAATT TAGTGGTTAA 878
       SlMLO15_LEAF  TTGGATAGCA ATTATAAGTC AAGTGTTATT AGAGGAAATT TAGTGGTTAA 881
       SlMLO15_ROOT  TTGGATAGCA ATTATAAGTC AAGTGTTATT AGAGGAAATT TAGTGGTTAA 1130
     SlMLO15_FLOWER  TTGGATAGCA ATTATAAGTC AAGTGTTATT AGAGGAAATT TAGTGGTTAA 775
      SlMLO15_FRUIT  TTGGATAGCA ATTATAAGTC AAGTGTTATT AGAGGAAATT TAGTGGTTAA 759

                                      4,520                         4,540
gDNA_Solyc02g077570  ACCTGAAGAT CAATTCTTTT GGTTTGGAAA ACCCAAACTT CTTCTTCTTC 4546
cDNA_Solyc02g077570  ACCTGAAGAT CAATTCTTTT GGTTTGGAAA ACCCAAACTT CTTCTTCTTC 928
       SlMLO15_LEAF  ACCTGAAGAT CAATTCTTTT GGTTTGGAAA ACCCAAACTT CTTCTTCTTC 931
       SlMLO15_ROOT  ACCTGAAGAT CAATTCTTTT GGTTTGGAAA ACCCAAACTT CTTCTTCTTC 1180
     SlMLO15_FLOWER  ACCTGAAGAT CAATTCTTTT GGTTTGGAAA ACCCAAACTT CTTCTTCTTC 825
      SlMLO15_FRUIT  ACCTGAAGAT CAATTCTTTT GGTTTGGAAA ACCCAAACTT CTTCTTCTTC 809

                          4,560                         4,580                     4,600
gDNA_Solyc02g077570  TCATGCATTT CATCCTCTTT CAGGTACTAC TATTTTAATT TTCACCTCTT 4596
cDNA_Solyc02g077570  TCATGCATTT CATCCTCTTT CAG------- ---------- ---------- 951
       SlMLO15_LEAF  TCATGCATTT CATCCTCTTT CAG------- ---------- ---------- 954
       SlMLO15_ROOT  TCATGCATTT CATCCTCTTT CAG------- ---------- ---------- 1203
     SlMLO15_FLOWER  TCATGCATTT CATCCTCTTT CAGGTACTAC TATTTTAATT TTCACCTCTT 875
      SlMLO15_FRUIT  TCATGCATTT CATCCTCTTT CAGGTACTAC TATTTTAATT TTCACCTCTT 859

                                      4,620                         4,640
gDNA_Solyc02g077570  TATTATTCAA TTCTATATTT ATATACTTAT TCATTTCAAT TCGTTATTTT 4646
cDNA_Solyc02g077570  ---------- ---------- ---------- ---------- ---------- 951
       SlMLO15_LEAF  ---------- ---------- ---------- ---------- ---------- 954
       SlMLO15_ROOT  ---------- ---------- ---------- ---------- ---------- 1203
     SlMLO15_FLOWER  TATTATTCAA TTCTATATTT ATATACTTAT TCATTTCAAT TCGTTATTTT 925
      SlMLO15_FRUIT  TATTATTCAA TTCTATATTT ATATACTTAT TCATTTCAAT TCGTTATTTT 909

                          4,660                         4,680                     4,700
gDNA_Solyc02g077570  TCGAAAAAAA AAATCTTCAG AACTCGTTTC AGTTGGCATT TTTTGCATGG 4696
cDNA_Solyc02g077570  ---------- ---------- AACTCGTTTC AGTTGGCATT TTTTGCATGG 981
       SlMLO15_LEAF  ---------- ---------- TACAATTATG GGTTA--AAT TCTTGCTTCC 982
       SlMLO15_ROOT  ---------- ---------- ---------- ---------- ---------- 1203
     SlMLO15_FLOWER  TCGAAAAAAA AAATCTTCAG AACTCGTTTC AGTTGGCATT TTTTGCATGG 975
      SlMLO15_FRUIT  TCGAAAAAAA AAATCTTCAG AACTCGTTTC AGTTGGCATT TTTTGCATGG 959

                                      4,720                         4,740
gDNA_Solyc02g077570  AC-----AAC GGTAAGCGTT ATGAT----- ---------- --------TT 4718
cDNA_Solyc02g077570  AC-----AAC GGTAAGCGTT ATGAT----- ---------- --------TT 1003
       SlMLO15_LEAF  ACCATAAAAC AGTAGATATT GTGACAAGGC TTGTTATGGG AGTGCTTGTT 1032
       SlMLO15_ROOT  ---------- ---------- ---------- ---------- ---------- 1203
     SlMLO15_FLOWER  AC-----AAC GGTAAGCGTT ATGAT----- ---------- --------TT 997
      SlMLO15_FRUIT  AC-----AAC GGTAAGCGTT ATGAT----- ---------- --------TT 981

                          4,760                         4,780                     4,800
gDNA_Solyc02g077570  TATGTTATTT ATATAAGCTA TTGAATCCCC TTGATATAT- ---------- 4757
cDNA_Solyc02g077570  TATGTTATTT ATATAAGCTA TTGAATCCCC TTGATATAT- ---------- 1042
       SlMLO15_LEAF  CATTTTCTTT GTGGATA-TG TCATATTGCC TTTATATGCT CTCCTTACTC 1081
       SlMLO15_ROOT  ---------- ---------- ---------- ---------- ---------- 1203
     SlMLO15_FLOWER  TATGTTATTT ATATAAGCTA TTGAATCCCC TTGATATAT- ---------- 1036
      SlMLO15_FRUIT  TATGTTATTT ATATAAGCTA TTGAATCCCC TTGATATAT- ---------- 1020
```

```
                                                    4,820                          4,840
gDNA_Solyc02g077570   ---------- ---------- ---------- ------ATGA ATTGA-----  4766
cDNA_Solyc02g077570   ---------- ---------- ---------- ------ATGA ATTGA-----  1051
        SIMLO15_LEAF  AGATGGGAAC CAAAATTAAA AGTAGTGTTT TGACTGATGA AATGATTAGT  1131
        SIMLO15_ROOT  ---------- ---------- ---------- ---------- ----------  1203
      SIMLO15_FLOWER  ---------- ---------- ---------- ------ATGA ATTGA-----  1045
       SIMLO15_FRUIT  ---------- ---------- ---------- ------ATGA ATTGA-----  1029
                                4,860                          4,880                 4,900
gDNA_Solyc02g077570   ---------- ---------- ---------- ---AGATCGA ----------  4773
cDNA_Solyc02g077570   ---------- ---------- ---------- ---AGATCGA ----------  1058
        SIMLO15_LEAF  AGGCTAAAAA GATGGCAAGA AAAAGCAAAG AGGAAATTGG CTAAAAGAAG  1181
        SIMLO15_ROOT  ---------- ---------- ---------- ---------- ----------  1203
      SIMLO15_FLOWER  ---------- ---------- ---------- ---AGATCGA ----------  1052
       SIMLO15_FRUIT  ---------- ---------- ---------- ---AGATCGA ----------  1036
                                                    4,920                          4,940
gDNA_Solyc02g077570   -ACTTACTTA TCAAA----- ---TATTATT TCAACAAATA TCA-------  4807
cDNA_Solyc02g077570   -ACTTACTTA TCAAA----- ---TATTATT TCAACAAATA TCA-------  1092
        SIMLO15_LEAF  CAATTACTTG TTAGCACAAA ATTTATTATC TTTAAATATT TCACCATCTT  1231
        SIMLO15_ROOT  ---------- ---------- ---------- ---------- ----------  1203
      SIMLO15_FLOWER  -ACTTACTTA TCAAA----- ---TATTATT TCAACAAATA TCA-------  1086
       SIMLO15_FRUIT  -ACTTACTTA TCAAA----- ---TATTATT TCAACAAATA TCA-------  1070
                                4,960                          4,980                 5,000
gDNA_Solyc02g077570   ----AACTTA ATTAGC---- ---------- ---------- ----------  4819
cDNA_Solyc02g077570   ----AACTTA ATTAGC---- ---------- ---------- ----------  1104
        SIMLO15_LEAF  TTGAAACTTC ATTGGATGTT ACATATTTAT CAACCGATAC TGAAAATGAT  1281
        SIMLO15_ROOT  ---------- ---------- ---------- ---------- ----------  1203
      SIMLO15_FLOWER  ----AACTTA ATTAGC---- ---------- ---------- ----------  1098
       SIMLO15_FRUIT  ----AACTTA ATTAGC---- ---------- ---------- ----------  1082
                                                    5,020                          5,040
gDNA_Solyc02g077570   ------ATTT TAAATAA--- ---------- ---------- ---------G  4831
cDNA_Solyc02g077570   ------ATTT TAAATAA--- ---------- ---------- ---------G  1116
        SIMLO15_LEAF  GGAGAAATTG TAGATGATCA GAGACAAATT CAGCAACACA CAGAATTTGG  1331
        SIMLO15_ROOT  ---------- ---------- ---------- ---------- ----------  1203
      SIMLO15_FLOWER  ------ATTT TAAATAA--- ---------- ---------- ---------G  1110
       SIMLO15_FRUIT  ------ATTT TAAATAA--- ---------- ---------- ---------G  1094
                                5,060                          5,080
gDNA_Solyc02g077570   ATTCTTA--- ---------- ---------- ---------- ----ACTAA  4843
cDNA_Solyc02g077570   ATTCTTA--- ---------- ---------- ---------- ----ACTAA  1128
        SIMLO15_LEAF  ATCTTTTGGT GGATTTCACT TGAGTAAATC TAGAGCAGCA CAACATTAG  1380
        SIMLO15_ROOT  ---------- ---------- ---------- ---------- ---------  1203
      SIMLO15_FLOWER  ATTCTTA--- ---------- ---------- ---------- ----ACTAA  1122
       SIMLO15_FRUIT  ATTCTTA--- ---------- ---------- ---------- ----ACTAA  1106
```
