# Supplementary material for: Genome-Wide Study of the Tomato SlMLO Gene Family and Its Functional Characterization in Response to the Powdery Mildew Fungus Oidium neolycopersici
Source: Front Plant Sci. 2016 Apr 6;7:380. doi: 10.3389/fpls.2016.00380 (PMC4986958; doi:10.3389/fpls.2016.00380)
Supplement: Supplementary file 2 [file DataSheet2.PDF]

```
                                                     TM1
SlMLO1_LEAF       1 M--------------EATPTWAIAVVCFILLAISIFIEQIIHHIGEWLLEKRKKSLYEAL
SlMLO1_ROOT       1 M--------------EATPTWAIAVVCFILLAISIFIEQIIHHIGEWLLEKRKKSLYEAL
SlMLO1_FLOWER     1 M--------------EATPTWAIAAVCFILLAISIFIEQIIHHIGEWLLEKRKKSLYEAL
SlMLO2_LEAF       1 MSGGG---GDEGTSLEFTPTWIVALVCTVIVAISLLVERIIHYGGKYLLKKNQKPLYEAL
SlMLO3_LEAF       1 MAGGGG----GGRSLEQTPTWAVAVVCFVLVAISIVIEHIIHLIGKWLKSKNKSALYEAL
SlMLO4_LEAF       1 MAGGG---DGTSRQLDQTPTWAVAGVCAVIILISIALEKIIHKLGTWLTERHKKALFEAL
SlMLO5_LEAF       1 MASTGCIRTCDERPLDETPTWAVAMVCFVLVVISLFIEQLIHHLGEWLWKKQKRPLYEAL
SlMLO5_FLOWER     1 MASTGCIRTCDERPLDETPTWAVAMVCFVLVVISLFIEQLIHHLGEWLWKKQKRPLYEAL
SlMLO5_FRUIT      1 MASTGCIRTCDERPLDETPTWAVAMVCFVLVVISLFIEQLIHHLGEWLWKKQKRPLYEAL
SlMLO6_LEAF       1 MAGGG---DGTSRQLDQTPTWAVAGVCAVIILISIALEKILHKLGTWLTDRHKKALFEAL
SlMLO6_ROOT       1 MAGGG---DGTSRQLDQTPTWAVAGVCAVIILISIALEKILHKLGTWLTDRHKKALFEAL
SlMLO6_FLOWER     1 MAGGG---DGTSRQLDQTPTWAVAGVCAVIILISIALEKILHKLGTWLTDRHKKALFEAL
SlMLO6_FRUIT      1 MAGGG---DGTSRQLDQTPTWAVAGVCAVIILISIALEKILHKLGTWLTDRHKKALFEAL
SlMLO7_LEAF       1 MSGGEG--EAEGSTLEFTPTWVVATVCTVIAAISLAVERLVHYTGKYLKKKNQKPLYEAL
SlMLO8_LEAF       1 MAGGGG----G-RSLEQTPTWAVALVCFALVAISIVIELIIHLIGKWLKSKHKRALYEAL
SlMLO9_LEAF       1 -----------------------------------------------------------
SlMLO9_FLOWER     1 MEGGG---EEEG-SLEYTPTWVVAAVCTVIVSISLLVERLIHYAGKRLKKKKQKHLYEAL
SlMLO9_FRUIT      1 MEGGG---EEEG-SLEYTPTWVVAAVCTVIVSISLLVERLIHYAGKRLKKKKQKHLYEAL
SlMLO10_LEAF      1 M---GNL---EGASFAETPTWAVATVVAVLVSIGFLIHGSLKKFGKWLHRTKREPLYAAL
SlMLO10_ROOT      1 M---GNL---EGASFAGTPTWAVATVVAVLVSIGFLIHGSLKKFGKWLHRTKREPLYAAL
SlMLO10_FLOWER    1 M---GNL---EGASFAETPTWAVATVVAVLVSIGFLIHGSLKKFGKWLHRTKREPLYAAL
SlMLO10_FRUIT     1 M---GNL---EGASFAETPTWAVATVVAVLVSIGFLIHGSLKKFGKWLHRTKREPLYAAL
SlMLO11_LEAF      1 M---A---EKSESSLEYTPTWVVAVVCFIIVLISLAAERGLHRLGKFFLQKNQDALFKAL
SlMLO11_ROOT      1 MA------EKSESSLEYTPTWVVAVVCFIIVLISLAAERGLHRLGKFFLQKNQDALFKAL
SlMLO11_FLOWER    1 M---A---EKSESSLEYTPTWVVAVVCFIIVLISLAAERGLHRLGKFFLQKNQDALFKAL
SlMLO11_FRUIT     1 M---A---EKSESSLEYTPTWVVAVVCFIIVLISLAAERGLHRLGKFFLQKNQDALFKAL
SlMLO12_FLOWER    1 MAGGG---GDGSRELDQTPTWAVALVCAVIVLISILLEKVLHKVGQTFERRRKSALVEAL
SlMLO13_LEAF      1 MEGA----FREGRSLKETPTWSVATVTTVMVFVCLFAQRSIYRFGRWLKKTRRKALFASV
SlMLO13_ROOT      1 M---EGA-FREGRSLKETPTRSVATVTTVMVFVCLFAQRSIYRFGRWLKKTRRKALFASV
SlMLO13_FLOWER    1 M---EGA-FREGRSLKETPTWSVATVTTVMVFVCLFAQRSIYRFGRWLKKTRRKALFASV
SlMLO13_FRUIT     1 M---EGA-FREGRSLKETPTXSVATVTTVMVFVCLFAQRSIYRFGRWLKKTRRKALFASV
SlMLO14_LEAF      1 M---SGEDTQELRSLALTPTWSVASVLTIFVAVSLLVERSIHRLSNWLRKTNRKPLLAAV
SlMLO15_LEAF      1 MAGSNNSRVIT---LVTTPTWAIAVVCFILITISILIEHVLHLL----------------
SlMLO15_ROOT      1 MAGSNNSRVI---TLVTTPTWAIAVVCFILITISILIEHVLHLL----------------
SlMLO15_FLOWER    1 MAGSNNSRVI---TLVTTPTWAIAVVCFILITISILIEHVLH-----LLAKRRKSLIQAL
SlMLO15_FRUIT     1 M-----------------------------------------------------------
SlMLO16_LEAF      1 MAGGGG---GGSPTLLNTPTWALA-TC--------------------LKKHRKTALFEAV
```

```
                                TM2
SlMLO1_LEAF       47 EKIKAELMLLGFLSLLLTVLQDPVSNLCVPKS-VGYSWHPCMAKEDAKS-----------
SlMLO1_ROOT       47 EKIKAELMLLGFLSLLLTVLQDPVSNLCVPKS-VGYSWHPCMAKEDAKS-----------
SlMLO1_FLOWER     47 EKIKAELMLLGFLSLLLTVLQDPVSNLCVPKS-VGYSWHPCMAKEDAKS-----------
SlMLO2_LEAF       58 QKIKEELMLLGFISLLLTVLQARILKICIPKH-LTNHWLPCK------------------
SlMLO3_LEAF       57 EKIKAELMLLGFISLLLTVGQSPISNICVSEK-LGNSWHPCSKKEED-------------
SlMLO4_LEAF       58 EKIKAELMVLGFISLTLVFSQYYIAGICIPTD-VANTLLPCPA-----------------
SlMLO5_LEAF       61 EKIKSELMLLGFISLFLTVVQDPMSKICIPRS-VGRSWHPCDINKHIDD-----------
SlMLO5_FLOWER     61 EKIKSELMLLGFISLFLTVVQDPMSKICIPRS-VGRSWHPCDINKHIDD-----------
SlMLO5_FRUIT      61 EKIKSELMLLGFISLFLTVVQDPMSKICIPRS-VGRSWHPCDINKHIDD-----------
SlMLO6_LEAF       58 EKVKAELMILGFISLTLVFSQYYIAGICIPPS-VADTMLPCPA-----------------
SlMLO6_ROOT       58 EKVKAELMILGFISLTLVFSQYYIAGICIPPS-VADTMLPCPA-----------------
SlMLO6_FLOWER     58 EKVKAELMILGFISLTLVFSQYYIAGICIPPS-VADTMLPCPA-----------------
SlMLO6_FRUIT      58 EKVKAELMILGFISLTLVFSQYYIAGICIPPS-VADTMLPCPA-----------------
SlMLO7_LEAF       59 QK----------------------------------------------------------
SlMLO8_LEAF       56 EKIKAELMLLGFISLLLTVGQDPISNICVSEK-IASTWHPCSKQKEAEM-----------
SlMLO9_LEAF        1 -------MLLGFISLLLTVFQSRIVEICVPPH-VVTHLLPCALPLEHTSFSPPTPTPTLT
SlMLO9_FLOWER     57 QKVKEELMLLGFISLLLTVFQSRIVEICVPPH-VVTHLLPCALPLEHTSFSPPTPTPTLT
SlMLO9_FRUIT      57 QKVKEELMLLGFISLLLTVFQSRIVEICVPPH-VVTHLLPCALPLEHTSFSPPTPTPTLT
SlMLO10_LEAF      55 EKIKEELMVFGLLSLLMGHWIVYIAKICVKSSAVSSHFYPCSPPRNKMKSAITRFALSGS
SlMLO10_ROOT      55 EKIKEELMVFGLLSLLMGHWIVYIAKICVKSSAVSSHFYPCSPPRNKMKSAITRFALSGS
SlMLO10_FLOWER    55 EKIKEELMVFGLLSLLMGHWIVYIAKICVKSSAVSSHFYPCSPPRNKMKSAITRFALSGS
SlMLO10_FRUIT     55 EKIKEELMVSGLLSLLMGHWIVYIAKICVKSSAVSSHFYPCSPPRNKMKSAITRFALSGS
SlMLO11_LEAF      55 QKLKEELMLLGFISLLLTVSQGAISQICVPEN-ISKVMLPCKL-----------------
SlMLO11_ROOT      55 QKLKEGLTTHGLILYL--------------------------------------------
SlMLO11_FLOWER    55 QKLKEELMLLGFISLLLTVSQGAISQICVPEN-ISKVMLPCKL-----------------
SlMLO11_FRUIT     55 QKLKEELMLLGFISLLLTVSQGAISQICVPEN-ISKVMLPCKL-----------------
SlMLO12_FLOWER    58 EKIKAELMVLGFISLLLTFGQNFISQICIPER-VADTMLPCPAQQHSRL-----------
SlMLO13_LEAF      57 EKIKEESX----------------------------------------------------
SlMLO13_ROOT      57 EKIKEELMLLGLISLLLGQCARQISQICVNSSLFTSRFYLCSQEDYDSSSSSTSDTIHSS
SlMLO13_FLOWER    57 EKIKEELMLLGLISLLLGQCARQISQICVNSSLFTSRFYLCSQEDYDSSSSSTSDTIHSS
SlMLO13_FRUIT     57 EKIKEELMLLGLISLLLGQCARQISQICVNSSLFTSRFYLCSQEDYDSSSSSTSDTIHSS
SlMLO14_LEAF      58 EKMKEELMLLGFISLLLTATSSIISNICIPSKFYNNAFAPCTKSEVDEEME-NKDLKERK
SlMLO15_LEAF      42 ----AKLMLLGFISLLLNVLQKPIAKICIPKG-AAQTFLPCQSFTTDD------------
SlMLO15_ROOT      42 ------------------------AKVCL-------------------------------
SlMLO15_FLOWER    53 NNIKS-------------------------------------------------------
SlMLO15_FRUIT      2 --------LLGFISLLLNVLQKPIAKICIPKG-AAQTFLPCQSFTTDD------------
SlMLO16_LEAF      37 EKLKSVLMQLGFLSLLLAVIQRPISKICIPNR-ITNSMLPCN------------------
```

```
SlMLO1_LEAF       95 -----EY-------------------------------------------DDPCLP-KGK
SlMLO1_ROOT       95 -----EY-------------------------------------------DDPCLP-KGK
SlMLO1_FLOWER     95 -----EY-------------------------------------------DDPCLP-KGK
SlMLO2_LEAF       99 -----KDDDVND-SVHFQTNFFSLIPGGRR-LLSGSANS------------GYCEA-KDK
SlMLO3_LEAF      103 -----SS----------IISEDSLSEQHRRRLLMDAAGGGVRRILAGGGGDDKCAA-KGK
SlMLO4_LEAF      100 -----------------VHKED-KTEEHRRRLLWHE-----RRILAGAEP--KCD--DGR
SlMLO5_LEAF      109 -----QY-------------------------------------------LDPCRI-KGK
SlMLO5_FLOWER    109 -----QY-------------------------------------------LDPCRI-KGK
SlMLO5_FRUIT     109 -----QY-------------------------------------------LDPCRI-KGK
SlMLO6_LEAF      100 -----------------NNKDAAKEEEHRRKLLWYE-----RRILAGAEP--KCK--EGR
SlMLO6_ROOT      100 -----------------NNKDAAKEEEHRRKLLWYE-----RRILAGAEP--KCK--EGR
SlMLO6_FLOWER    100 -----------------NNKDAAKEEEHRRKLLWYE-----RRILAGAEP--KCK--EGR
SlMLO6_FRUIT     100 -----------------NNKDAAKEEEHRRKLLWYE-----RRILAGAEP--KCK--EGR
SlMLO7_LEAF       61 ------------------------------------------------------------
SlMLO8_LEAF      104 -----NK----------YISGDL--EGHRRRLFT-ADDGGVRRVLAAAG-TDKCAD-KGK
SlMLO9_LEAF       53 PPPPHKEPQVNNQAVHYHAG-----PHHQRHLLEEETMSAE----------GYCRH-KNK
SlMLO9_FLOWER    116 PPPPHKEPQVNNQAVHYHAG-----PHHQRHLLEEETMSAE----------GYCRH-KNK
SlMLO9_FRUIT     116 PPPPHKEPQVNNQAVHYHAG-----PHHQRHLLEEETMSAE----------GYCRH-KNK
SlMLO10_LEAF     115 SYSNF-------------------------------ST---SRLLLSSGHEDFC--PEGP
SlMLO10_ROOT     115 SYSNF-------------------------------ST---SRLLLSSGHEDFC--PEGL
SlMLO10_FLOWER   115 SYSNF-------------------------------ST---SRLLLSSGHEDFC--PEGL
SlMLO10_FRUIT    115 SYSNF-------------------------------ST---SRLLLSSGHEDFC--PEGL
SlMLO11_LEAF      97 -----KEASTSK---HLITG---------RHLLAGS--SGE----------QHCNHHEGK
SlMLO11_ROOT         ------------------------------------------------------------
SlMLO11_FLOWER    97 -----KEASTSK---HLITG---------RHLLAGS--SGE----------QHCNHHEGK
SlMLO11_FRUIT     97 -----KEASTSK---HLITG---------RHLLAGS--SGE----------QHCNHHEGK
SlMLO12_FLOWER   106 -----GYGGGHHGPGQGHHKTPEAGEAHRRLL--SD-----HRILAADSPGDTCR--PGQ
SlMLO13_LEAF         ------------------------------------------------------------
SlMLO13_ROOT     117 ILSNH-------------------------------TTLIPPKGIYQQSHQ--C--GEGR
SlMLO13_FLOWER   117 ILSNH-------------------------------TTLIPPKGIYQQSHQ--C--GEGR
SlMLO13_FRUIT    117 ILSNH-------------------------------TTLIPPKGIYQQSHQ--C--GEGR
SlMLO14_LEAF     117 LLMAF-------------------------------GL---HRRVLNSFKQNTC--GENH
SlMLO15_LEAF      85 -----------------------------------------------VEEESNCE-QQGK
SlMLO15_ROOT      47 ------------------------------------------------------------
SlMLO15_FLOWER    58 ------------------------------------------------------------
SlMLO15_FRUIT     41 -----------------------------------------------VEEESNCE-QQGK
SlMLO16_LEAF      78 -----------------------------RVLLDSTKTIKERELASVQNSQDHCAS-RGM
```

```
                                       TM3
SlMLO1_LEAF      106 VQFASSYAIHQLHIFIFVLAVAHVLYCIATFALGRLKMRKWRAWEDETKTMEYQFYN---
SlMLO1_ROOT      106 VQFASSYAIHQLHIFIFALAVAHVLYCIATLALGRLKMRKWRAWEDETKTMEYQFYN---
SlMLO1_FLOWER    106 VQFASSYAIHQLHIFIFVLAVAHVLYCIATFALGRLKMRKWRAWEDETKTMEYQFYN---
SlMLO2_LEAF      139 APLLSLTALHHLHTFIFVLAVSHVTFSALTILFGGIKIRQWKSWEDSIKKEEYNPEEV-L
SlMLO3_LEAF      147 VPFVSADGIHQLHIFIFVLAVFHVLYCVTTLALGRAKMRSWKSWENETKTAEYEFSH---
SlMLO4_LEAF      133 VSLISIDALHQIHILIFFLAVLHVIYSAITMWLGKLKIRGWKCWEQETATHDYEFSN---
SlMLO5_LEAF      120 LQFASKYAIHQLHIFIFVLXVAHVLYCITTLGIGKLRMRTWRAWEDESKTIEYQFYN---
SlMLO5_FLOWER    120 LQFASKYAIHQLHIFIFVLAVAHVLYCITTLGIGKLRMRTWRAWEDESKTIEYQFYN---
SlMLO5_FRUIT     120 LQFASKYAIHQLHIFIFVLAVAHVLYCITTLGIGKLRMRTWRAWEDESKTIEYQFYN---
SlMLO6_LEAF      134 VPLVTVEALHQIHILIFFLAVLHVLYSAITMWLGRLKIRGWKGWEQETSTHSYEFTN---
SlMLO6_ROOT      134 VPLVTVEALHQIHILIFFLAVLHVLYSAITMWLGRLKIRGWKGWEQETSTHSYEFTN---
SlMLO6_FLOWER    134 VPLVTVEALHQIHILIFFLAVLHVLYSAITMWLGRLKIRGWKGWEQETSTHSYEFTN---
SlMLO6_FRUIT     134 VPLVTVEALHQIHILIFFLAVLHVLYSAITMWLGRLKIRGWKGWEQETSTHSYEFTN---
SlMLO7_LEAF       61 ------------------------------------------------------------
SlMLO8_LEAF      144 VAFVSADGIHQLHIFIFVLAIFHVFYCVTTLALGRAKMSRWKIWEKETRTAEYQFSH---
SlMLO9_LEAF       97 VPLLSLEALHHLHVFIFVLAIVHVTFSVLTIVFGGAKIRQWKQWEDAIVKDDYESEDAHL
SlMLO9_FLOWER    160 VPLLSLEALHHLHVFIFVLAIVHVTFSVLTIVFGGAKIRQWKQWEDAIVKDDYESEDAHL
SlMLO9_FRUIT     160 VPLLSLEALHHLHVFIFVLAIVHVTFSVLTIVFGGAKIRQWKQWEDAIVKDDYESEDAHL
SlMLO10_LEAF     139 QSFASKGSLEQLHRFLLVLGVSHVSYSFFAIALAMIKIYSWRTWENYAKSIALQRLEGSE
SlMLO10_ROOT     139 QSFASKESLEQLHRFLLVLGVSHVSYSFFAIALAMIKIYSWRTWENYAKSIALQRLEGSE
SlMLO10_FLOWER   139 QSFASKESLEQLHRFLLVLGVSHVSYSFFAIALAMIKIYSWRTWENYAKSIALQRLEGSE
SlMLO10_FRUIT    139 QSFASKESLEQLHRFLLVLGVSHVSYSFFAIALAMIKIYSX-------------------
SlMLO11_LEAF     128 VPLLSLEALHQLHIFIFVLAVTHVIFCATTMVLGGAKIQQWRHWENSIQKQS--------
SlMLO11_ROOT         ------------------------------------------------------------
SlMLO11_FLOWER   128 VPLLSLEALHQLHIFIFVLAVTHVIFCATTMVLGGAKIQQWRHWENSIQKQS--------
SlMLO11_FRUIT    128 VPLLSLEALHQLHIFIFVLAVTHVIFCATTMVLGGAKIQQWRHWENSIQKQS--------
SlMLO12_FLOWER   152 VPLISIHGLHQLHIFIFFLAVFHVIYSAITMLLGRLKIREWKEWERQVX-HEYEASN---
SlMLO13_LEAF         ------------------------------------------------------------
SlMLO13_ROOT     142 EPFVSYEGLEQLHRFLFVLGITHVLYSCIVVGLAMTKIYSWRKWENQVSSGEQNNLQ---
SlMLO13_FLOWER   142 EPFVSYEGLEQLHRFLFVLGITHVLYSCIVVGLAMTKIYSWRKWENQVSSGEQNNLQ---
SlMLO13_FRUIT    142 EPFVSYEGLEQLHRFLFVLGIAHVLYSCIVVGLAMTKIYSWRKWENQVSSGEQNNLQ---
SlMLO14_LEAF     141 EPFVSYEGLEQLHRFIFVMAITHISYSCLTMLLAIVKVHSWRVWEDQAQNDRHDVLT---
SlMLO15_LEAF      97 KSLMSRAAS------AFNICSCFLSYSFLHPHL--QPWNTWKFWEAETTTLDYQFSH---
SlMLO15_ROOT      47 ------------------------------------------------------------
SlMLO15_FLOWER    58 ------------------------------------------------------------
SlMLO15_FRUIT     53 KSLMSRAA----------------------------------------------------
SlMLO16_LEAF     108 TSFMSQIGINQLNNFIFVLAVMQIVYSVVTMALGRAKMKQWKKWEDETKTIEYMAAN---
```

```
SlMLO1_LEAF      163 D--PERFRFARETSFGRRHLHF-WSKSPVLLSIVCFFRQFFSSVAKVDYLTLRHGFMMAH
SlMLO1_ROOT      163 D--PERFRFARETSFGRRHLHF-WSKSPVLLSIVCFFRQFFSSVAKVDYLTLRHGFMMAH
SlMLO1_FLOWER    163 D--PERFRFARETSFGRRHLHF-WSKSPVLLSIVCFFRQFFSSVAKVDYLTLRHGFMMAH
SlMLO2_LEAF      198 R--SKVTHVHDHDFIKGRFLGF-GKRSTLLGWLHSFVKQFFGSVTKLDYTTLRLGFIMTH
SlMLO3_LEAF      204 D--PERFRFTRETSFGRRHLSF-WTKNPVLLWIVCFFRQFVRSVPKVDYLTLRHGFITAH
SlMLO4_LEAF      190 D--PSRFRLTHETSFVRAHTSF-WTNIPIFFYIGCFFRQFFRSVNKSDYLTLRNGFISVH
SlMLO5_LEAF      177 D--PERFRFARETSFGRKHLHF-WSNSPILLWIVCFFRQFYASVEKVDYLTLRHGFAMAH
SlMLO5_FLOWER    177 D--PERFRFARETSFGRKHLHF-WSNSPILLWIVCFFRQFYASVEKVDYLTLRHGFAMAH
SlMLO5_FRUIT     177 D--PERFRFARETSFGRKHLHF-WSNSPILLWIVCFFRQFYASVEKVDYLTLRHGFAMAH
SlMLO6_LEAF      191 D--PSRFRLTHETSFVRAHTSF-WTRIPIFF--GCFFRQFFKSVSKSDYLALRNGFISVH
SlMLO6_ROOT      191 D--PSRFRLTHETSFVRAHTSF-WTRIPIFFYIGCFFRQFFKSVSKSDYLALRNGFISVH
SlMLO6_FLOWER    191 D--PSRFRLTHETSFVRAHTSF-WTRIPIFFYIGCFFRQFFKSVSKSDYLALRNGFISVH
SlMLO6_FRUIT     191 D--PSRFRLTHETSFVRAHTSF-WTRIPIFFYIGCFFRQFFKSVSKSDYLALRNGFISVH
SlMLO7_LEAF       61 ------------------------------------------------------------
SlMLO8_LEAF      201 D--PERFRFARDTSFGRRHLSF-WTKNSVLLWIVCFFRQFVRSVPKVDYLTLRHGFITAH
SlMLO9_LEAF      157 K--PTVTHVFEHDFIRNRFQGM-GTQSAILGWVRSFFKQFYASVNESDYRALRLGFIMTH
SlMLO9_FLOWER    220 K--PTVTHVFEHDFIRNRFQGM-GTQSAILGWVRSFFKQFYASVNESDYRALRLGFIMTH
SlMLO9_FRUIT     220 K--PTVTHVFEHDFIRNRFQGM-GTQSAILGWVRSFFKQFYASVNESDYRALRLGFIMTH
SlMLO10_LEAF     199 EAVPNNTRMGRLSTFTFHQTTHPWSQHRALVWLLCFSRQFWSSINEADYMALRLGFITTH
SlMLO10_ROOT     199 EAVPNNTRMGRLSTFTFHQTTHPWSQHRALVWLLCFSRQFWSSINEADYMALRLGFITTH
SlMLO10_FLOWER   199 EAVPNNTRMGRLSTFTFHQTTHPWSQHRALVWLLCFSRQFWSSINEADYMALRLGFITTH
SlMLO10_FRUIT        ------------------------------------------------------------
SlMLO11_LEAF     180 K--PHHVHILHIQSFVDRAGKR-WRKYALISWTVAFFKQFYGSVTKSDYIVLRTGFIKKH
SlMLO11_ROOT         ------------------------------------------------------------
SlMLO11_FLOWER   180 K--PHHVHILHIQSFVDRAGKR-WRKYALISWTVAFFKQFYGSVTKSDYIVLRTGFIKKH
SlMLO11_FRUIT    180 K--PHHVHILHIQSFVDRAGKR-WRKYALISWTVAFFKQFYGSVTKSDYIVLRTGFIKKH
SlMLO12_FLOWER   208 D--ASRFRLTKETSFVXDNTSF-GTT-PVVFYSVCFFRQFFRSVRKADYLTMRHGFISVH
SlMLO13_LEAF         ------------------------------------------------------------
SlMLO13_ROOT     199 --VPKNKEMRRQSTFALHHASHPWSRSRILIWMLCFLRQFRTSIHKSDYLALRLGFITNH
SlMLO13_FLOWER   199 --VPKNKEMRRQSTFALHHASHPWSRSRILIWMLCFLRQFRTSIHKSDYLALRLGFITNH
SlMLO13_FRUIT    199 --VPKNKEMRRQSTFALHHASHPWSRSRILIWMLCFLRQFRTSIHKSDYLALRLGFITNH
SlMLO14_LEAF     198 -EISRAKTFRRQSTFVRVHTSSPLARNHFLVWVTCFFRQFGRTVVRADYLTLRKGFIMNH
SlMLO15_LEAF     146 D--PRRFQLIHQTSFGKRHLNF-WSEHRFLRFPVCLLRQFYGSVYKVDYLTLRHGFIMAH
SlMLO15_ROOT      47 ------------------------------------------------YFCMR-------
SlMLO15_FLOWER    58 ------------------------------------------------------------
SlMLO15_FRUIT     61 ------------------------------------------------------------
SlMLO16_LEAF     165 D--PNRFRFTRETTFGRRHINK-FTNMIPQLWIKCFLRQFFHSVAKTDYLTLRHGFITAH
```

```
                                                          TM4
SlMlo1_LEAF       220 LTPQNQNNFDFQLYINRAVDKDFKVVVGISP--ALWL----FTVLYF-------------
SlMLO1_ROOT       220 LTPQNQNNFDFQLYINRAVDRDFKVVVGISP--ALWL----FTVLYF-------------
SlMLO1_FLOWER     220 LTPQNQNNFDFQLYINRAVDKDFKVVVGISP--ALWL----FTVLYF-------------
SlMLO2_LEAF       255 C--KKNPKFDFHKYMTRVLEADFKKVVGIS--WYLWVFVVLFLLL---------------
SlMLO3_LEAF       261 LAPQSHQKFDFRKYIKRSLEEDFKVVVGISPP--IWF----LAVLFL-------------
SlMLO4_LEAF       247 LAPGSK--FNFQKYIKRSLEDDFKVVVGVS-P-VLWGSFVVFLLL---------------
SlMLO5_LEAF       234 LAPQQEKNFDFQLYINRALEEDFKDVVGISP--LLWM----FAALYF-------------
SlMLO5_FLOWER     234 LAPQQEKNFDFQLYINRALEEDFKDVVGISP--LLWM----FAXLYF-------------
SlMLO5_FRUIT      234 LAPQQEKNFDFQLYINRALEEDFKDVVGISKKNVVYIIHENFTLLYVAVHCYGCLQXSTF
SlMLO6_LEAF       246 LAPGSK--FNFQKYIKRSLEDDFKVVVGVSSP-VLWASFVLFLLL---------------
SlMLO6_ROOT       248 LAPGSK--FNFQKYIKRTLEDDFKVVVGVS-P-VLWASFVLFLLL---------------
SlMLO6_FLOWER     248 LAPGSK--FNFQKYIKRTLEDDFKVVVGVS-P-VLWASFVLFLLL---------------
SlMLO6_FRUIT      248 LAPGSK--FNFQKYIKRTLEDDFKVVVGVS-P-VLWASFVLFLLL---------------
SlMLO7_LEAF        61 ------------------------------------------------------------
SlMLO8_LEAF       258 LAPQSNINFDFQKYIKRSLEEDFKVVVSISPP--IWF----LAVLFL-------------
SlMLO9_LEAF       214 C--KGNPRFNFHRYMIRALEDDFRTVVGIS--WYLWIFVILFLLL---------------
SlMLO9_FLOWER     277 C--KGNPRFNFHRYMIRALEDDFRTVVGIS--WYLWIFVILFLLL---------------
SlMLO9_FRUIT      277 C--KGNPRFNFHRYMIRALEDDFRTVVGIS--WYLWIFVILFLLL---------------
SlMLO10_LEAF      259 QLPLT---YDFHKYMLRSMEEEFRDIVGIS--VPLWIFVIVCVFL---------------
SlMLO10_ROOT      259 QLPLT---YDFHKYMLRSMEEEFRDIVGIS--VPLWIFVIVCVFL---------------
SlMLO10_FLOWER    259 QLPLT---YDFHKYMLRSMEEEFRDIVGIS--VPLWIFVIVCVFL---------------
SlMLO10_FRUIT         ------------------------------------------------------------
SlMLO11_LEAF      237 C--PSNPTYNFHRYILRTLEHDFKKIVGIS--WYLWLFVVLFLLI---------------
SlMLO11_ROOT          ------------------------------------------------------------
SlMLO11_FLOWER    237 C--PSNPTYNFHRYILRTLEHDFKKIVGIS--WYLWLFVVLFLLI---------------
SlMLO11_FRUIT     237 C--PSNPTYNFHRYILRTLEHDFKKIVGIS--WYLWLFVVLFLLI---------------
SlMLO12_FLOWER    264 LAPGSK--FNFQKYIKRSLEDDFKVIVGINS--LLWFSAVVYLLL---------------
SlMLO13_LEAF          ------------------------------------------------------------
SlMLO13_ROOT      257 KLPLT---YNFHKYMVRSMEDEFYEIVGIS--WLLWGYAIICIFI---------------
SlMLO13_FLOWER    257 KLPLT---YNFHKYMVRSMEDEFYEIVGIS--WLLWGYAIICIFI---------------
SlMLO13_FRUIT     257 KLPLT---YNFHKYMVRSMEDEFYEIVGIS--WLLWGYAIICIFI---------------
SlMLO14_LEAF      257 NLTSN---YDFHSYMIRSMEEEFQRIVGVS--GPLWGFVVGFMLF---------------
SlMLO15_LEAF      203 FAEGTE--FDFHKYIRRALDKDFQVVVAISP--WIWTFSMLFIFF---------------
SlMLO15_ROOT       52 ------------------------------------------------------------
SlMLO15_FLOWER     58 ------------------------------------------------------------
SlMLO15_FRUIT      61 ------------------------------------------------------------
SlMLO16_LEAF      222 LS--STNVLNFQKYIERSLEDDFKVVVGISP--FMWLIVVIFLLV---------------
```

```
                                 TM5
SlMLO1_LEAF      261 ----LTTTDRLYSYLWVPFIPLVIILLVGTKLQMIITEMGVRISERGDIVKGVPVVETGD
SlMLO1_ROOT      261 ----LTTTDRLYSYLWVPFIPLVIILLVGTKLQMIITEMGVRISERGDIVKGVPVVETGD
SlMLO1_FLOWER    261 ----LTTTDRLYSYLWVPFIPLVIILLVGTKLQMIITEMGVRISERGDIVKGVPVVETGD
SlMLO2_LEAF      296 ------NVHGWHTYFWIAFVPFGLLLAVGTKLEHVITQLAGEVAAKHIAVEGDLVVKPSD
SlMLO3_LEAF      302 ----LFNTHGWYSYLWLPFIPLIVILLVGTKLQVIITKMGLRIHERGEVVKGVPVVQPGD
SlMLO4_LEAF      288 ------NVSGWHALFWASLIPLIIILAVGTQLQAVLTRMALDISERHAVVQGIPLVQGSD
SlMLO5_LEAF      275 ----LTTTNGWYSYYWLPFIPLIIILLVGTKLQVIITKMGLRIKERGDIVKGTPLVEPGD
SlMLO5_FLOWER    275 ----LTTTNGWYSYYWLPFIPLIIILLVGTKLQVIITKMGLRIKERGDIVKGTPLVEPGD
SlMLO5_FRUIT     294 SLLPMICASGWYSYYWLPFIPLIIILLVGTKLQVIITKMGLRIKERGDIVKGTPLVEPGD
SlMLO6_LEAF      288 ------NVSGWQALFWASLIPLIIILAVGTKLQAVLTRMALDIKERHAVVQGIPLVQASD
SlMLO6_ROOT      289 ------NVSGWQALFWASLIPLIIILAVGTKLQAVLTRMALDIKERHAVVQGIPLVQASD
SlMLO6_FLOWER    289 ------NVSGWQALFWASLIPLIIILAVGTKLQAVLTRMALDIKERHAVVQGIPLVQASD
SlMLO6_FRUIT     289 ------NVSGWQALFWASLIPLIIILAVGTKLQAVLTRMALDIKERHAVVQGIPLVQASD
SlMLO7_LEAF       61 ------------------------------------------------------------
SlMLO8_LEAF      299 ----LFNTHGWYSYLWLPFIPLLVILLVGTKLQVIITKMGLRIQERGEVVKGVPVVQPGD
SlMLO9_LEAF      255 ------NINGWHTYFWIAFFPFILLLSVGTKLEHVILQLAHEIAEKHVAIEGELVVTPSD
SlMLO9_FLOWER    318 ------NINGWHTYFWIAFFPFILLLSVGTKLEHVILQLAHEIAEKHVAIEGELVVTPSD
SlMLO9_FRUIT     318 ------NINGWHTYFWIAFFPFILLLSVGTKLEHVILQLAHEIAEKHVAIEGELVVTPSD
SlMLO10_LEAF     299 ------SFHGTNIYFWISFFPAILILLVGTKLHRVVVKLAVEIID-SSPLEGFHQFNLRD
SlMLO10_ROOT     299 ------SFHGTNIYFWISFFPAILILLVGTKLHRVVVKLAVEIID-SSPLEGFHQFNLRD
SlMLO10_FLOWER   299 ------SFHGTNIYFWISFFPAILILLVGTKLHRVVVKLAVEIID-SSPLEGFHQFNLRD
SlMLO10_FRUIT        ------------------------------------------------------------
SlMLO11_LEAF     278 ------NIAGWHSYFWLSFLPLVLLLLVGTKLEHIITELAQEVSERSSVVDETTPIKPSD
SlMLO11_ROOT         ------------------------------------------------------------
SlMLO11_FLOWER   278 ------NIAGWHSYFWLSFLPLVLLLLAGTKLEHIITELAQEVSERSSVVDETTPIKPSD
SlMLO11_FRUIT    278 ------NIAGWHSYFWLSFLPLVLLLLVGTKLEHIITELAQEVSERSSVVDETTPIKPSD
SlMLO12_FLOWER   305 ------NVHGWQAMFWLSIMPLVIILAVGTKLQAIIAQMALEIQERHAVVQGIPLVQISD
SlMLO13_LEAF         ------------------------------------------------------------
SlMLO13_ROOT     297 ------NIHGLNIYFWLSFIPAILVVVVGTKLQHVVSSLALEIAEPKGPLIGL-QVKPRD
SlMLO13_FLOWER   297 ------NIHGLNIYFWLSFIPAILVVVVGTKLQHVVSSLALEIAEPKGPLIGL-QVKPRD
SlMLO13_FRUIT    297 ------NIHGLNIYFWLSFIPAILVVVVGTKLQHVVSSLALEIAEPKGPLIGL-QVKPRD
SlMLO14_LEAF     297 ------NVKGSNLYFWIALIPIILVLLVGTKLQHVIATLALESAGITGSFSRV-KLKPRD
SlMLO15_LEAF     244 ------NANVFHSTYWLPFIPLAMLVAVGTKLQGIITKMCLDSNYKSSVIRGNLVVKPED
SlMLO15_ROOT      52 ------------------------------------------------------------
SlMLO15_FLOWER    58 ----------------------------------------------------------GE
SlMLO15_FRUIT     61 ------------------------------------------------------------
SlMLO16_LEAF     263 ------DVHGWNIYLWVSFLPLITVLVIGTKLETIVAQMALQLENQESVVIGSPLVQPND
```

```
                                       TM6
SlMLO1_LEAF      317 HLFWFNRPALVLFLINFVLFQ-NAFQVAFFFWSWWKFGFPSCFHKNAADLAIRLTMGVII
SlMLO1_ROOT      317 HLFWFNRPALVLFLINFVLFQ-NAFQVAFFFWSWWKFGFPSCFHKNAADLAIRLTMGVII
SlMLO1_FLOWER    317 HLFWFNRPALVLFLINFVLFQ-NAFQVAFFFWSWWKFGFPSCFHKNAADLAIRLTMGVII
SlMLO2_LEAF      350 DHFWFHRPRLVLFLIHILLFQ-NSFEIAFFFWIWAQYRFNSCIMGQVGYIIPRPVIGVFV
SlMLO3_LEAF      358 HLFWFNRPRLILYLINFVLFQ-NAFQLAFFAWTWYEFGLKSCYHDHTEDIVIRITMGVLI
SlMLO4_LEAF      342 NYFWFGRPQLVLHLIHFALFQ-NAFQITYFLWIWYEYGLKSCFHDKFGFVIAKIALGVGV
SlMLO5_LEAF      331 DLFWFNRPDLLHFFIHFVLFQ-NAYQLAFFAWSWWKFNLPSCFHKNVTDIAITLSMGALI
SlMLO5_FLOWER    331 DLFWFNRPDLLHFFIHFVLFQ-NAYQLAFFAWSWWKFNLPSCFHKNVTDIAITLSMGALI
SlMLO5_FRUIT     354 DLFWFNRPDLLHFFIHFVLFQ-NAYQLAFFAWSWWKFNLPSCFHKNVTDIAITLSMGALI
SlMLO6_LEAF      342 KYFWFGRPRLVLHLIHFALFQQNAFQITYFLWIWYEYGLKSCFHEAFELVIAKIVIG---
SlMLO6_ROOT      343 KYFWFGRPRLVLHLIHFALFQ-NAFQITYFLWIWYEYGLKSCFHEAFELVIAKIVIGVGV
SlMLO6_FLOWER    343 KYFWFGRPRLVLHLIHFALFQ-NAFQITYFLWIWYEYGLKSCFHEAFELVIAKIVIGVGV
SlMLO6_FRUIT     343 KYFWFGRPRLVLHLIHFALFQ-NAFQITYFLWIWYEYGLKSCFHEAFELVIAKIVIGVGV
SlMLO7_LEAF       61 ------------------------------------------------------------
SlMLO8_LEAF      355 DLFWFNRPRLLLFLINFVLFQ-NAFQLAFFAWTWYEFGLKSCFHDQTEDIVIRMTMGVLI
SlMLO9_LEAF      309 NHFWFDNPQIILLLIHFILFQ-NAFEIAFFFWILFQYGFHSCIMGKYVFVIPRLVIGVII
SlMLO9_FLOWER    372 NHFWFDNPQIILLLIHFILFQ-NAFEIAFFFWILFQYGFHSCIMGKYVFVIPRLVIGVII
SlMLO9_FRUIT     372 NHFWFDNPQIILLLIHFILFQ-NAFEIAFFFWILFQYGFHSCIMGKYVFVIPRLVIXVII
SlMLO10_LEAF     352 ELFWFGKPRFLLRIIQFVSFQ-NAFEMATYIWSLWEIKGSSCFTDNHTFLVIRLSFGVVS
SlMLO10_ROOT     352 ELFWFGKPRFLLRIIQFVSLQ-NAFEMATYIWSLWEIKGSSCFTDNHTFLVIRLSFGVVS
SlMLO10_FLOWER   352 ELFWFGKPRFLLRIIQFVSFQ-NAFEMATYIWSLWEIKGSSCFTDNHTFLVIRLSFGVVS
SlMLO10_FRUIT        ------------------------------------------------------------
SlMLO11_LEAF     332 ELFWFDSPNLVLYLIHFILFQ-DSFEIAFFVWIWCTYGFKSCIMEDLGFIIPRLIIGVIV
SlMLO11_ROOT         ------------------------------------------------------------
SlMLO11_FLOWER   332 ELFWFDSPNLVLYLIHFILFQ-NSFEIAFFVWIWCTYGFKSCIMEDLGFIIPRLIIGVIV
SlMLO11_FRUIT    332 ELFWFDSPNLVLYLIHFILFQ-NSFEIAFFVWIWCTYGFKSCIMEDLGFIIPRLIIGVIV
SlMLO12_FLOWER   359 RNFWFGKPTLVLHLVHLTLFQ-NAFEITYFLWITYEFGLHSCFHDNFYLSLLRVAIGLGV
SlMLO13_LEAF         ------------------------------------------------------------
SlMLO13_ROOT     350 ELFWFGKPKILLRLIQFISFQ-NAFEMATFIWSLWGLKQRSCFMKNHAMVMIRLISGVLV
SlMLO13_FLOWER   350 ELFWFGKPKILLRLIQFISFQ-NAFEMATFIWSLWGLKQRSCFMKNHAMVMIRLISGVLV
SlMLO13_FRUIT    350 ELFWFGKPKILLRLIQFISFQ-NAFEMATFIWSLWGLKQRSCFMKNHAMVMIRLISGVLV
SlMLO14_LEAF     350 ELFWFKKPELLLSLIHFVLFQ-NAFELASFFWFWWQFGYNSCFIKNHTLVYLRLIMGFAG
SlMLO15_LEAF     298 QFFWFGKPKLLLLLMHFILFQYN--------------YGLNSCFHHKTVDIVTRLVMGVLV
SlMLO15_ROOT      52 ------------------------------------------------------------
SlMLO15_FLOWER    60 EIF----------------------------------NVKSSF-----------------
SlMLO15_FRUIT     61 ----------------------SAFNIC------------SCFL----------------
SlMLO16_LEAF     317 SLFWFNQPQFVLTLLHYTLF-INAFELAFFVWVTWQFGISSCYHEHVEIIVVRVVLALMV
```

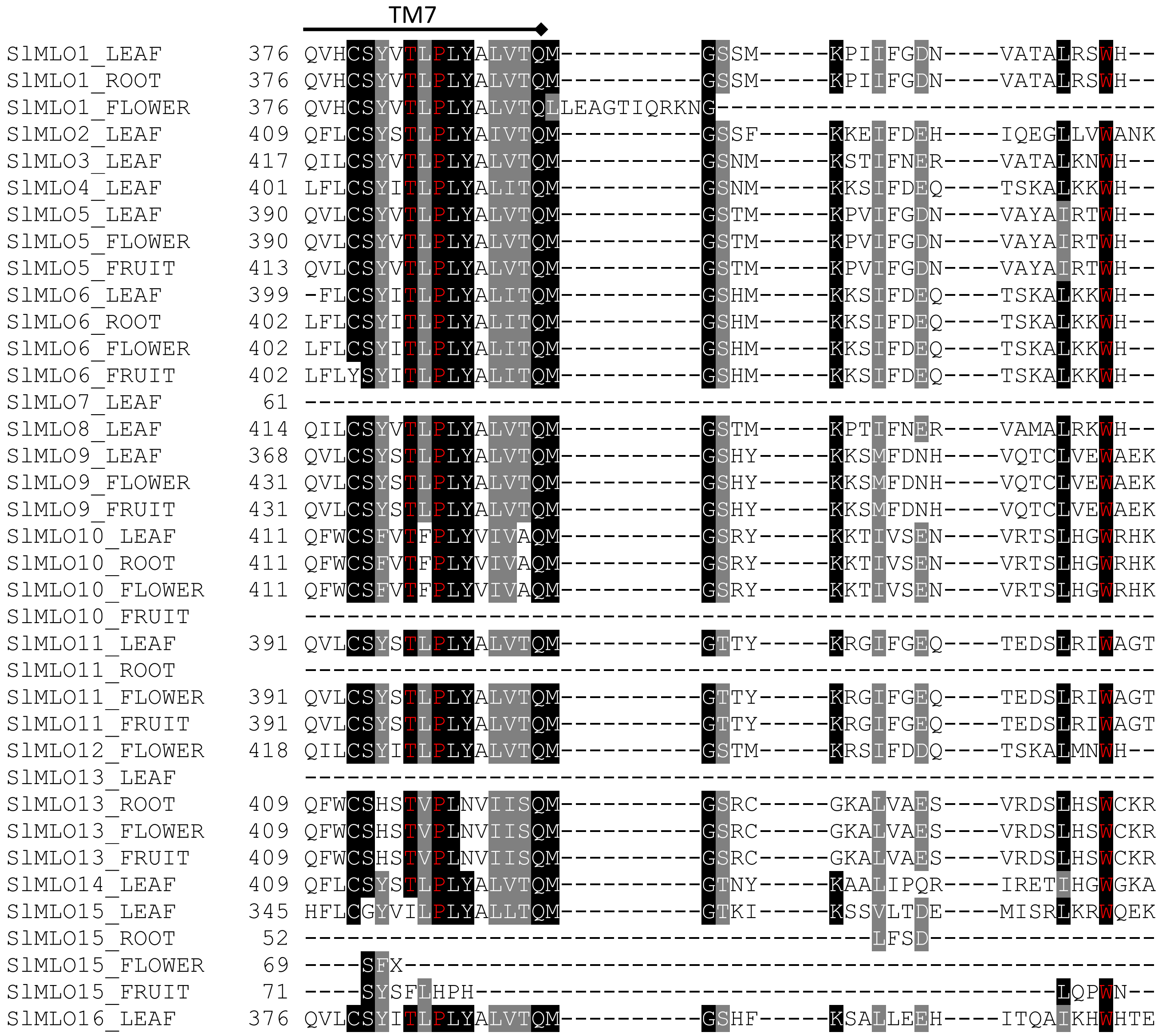
TM7
SlMLO1_LEAF 376 QVHCSYVTLPLYALVTQM----------GSSM-----KPIIFGDN----VATALRSWH--
SlMLO1_ROOT 376 QVHCSYVTLPLYALVTQM----------GSSM-----KPIIFGDN----VATALRSWH--
SlMLO1_FLOWER 376 QVHCSYVTLPLYALVTQLLEAGTIQRKNG-------------------------------
SlMLO2_LEAF 409 QFLCSYSTLPLYAIVTQM----------GSSF-----KKEIFDEH----IQEGLLVWANK
SlMLO3_LEAF 417 QILCSYVTLPLYALVTQM----------GSNM-----KSTIFNER----VATALKNWH--
SlMLO4_LEAF 401 LFLCSYITLPLYALITQM----------GSNM-----KKSIFDEQ----TSKALKKWH--
SlMLO5_LEAF 390 QVLCSYVTLPLYALVTQM----------GSTM-----KPVIFGDN----VAYAIRTWH--
SlMLO5_FLOWER 390 QVLCSYVTLPLYALVTQM----------GSTM-----KPVIFGDN----VAYAIRTWH--
SlMLO5_FRUIT 413 QVLCSYVTLPLYALVTQM----------GSTM-----KPVIFGDN----VAYAIRTWH--
SlMLO6_LEAF 399 -FLCSYITLPLYALITQM----------GSHM-----KKSIFDEQ----TSKALKKWH--
SlMLO6_ROOT 402 LFLCSYITLPLYALITQM----------GSHM-----KKSIFDEQ----TSKALKKWH--
SlMLO6_FLOWER 402 LFLCSYITLPLYALITQM----------GSHM-----KKSIFDEQ----TSKALKKWH--
SlMLO6_FRUIT 402 LFLYSYITLPLYALITQM----------GSHM-----KKSIFDEQ----TSKALKKWH--
SlMLO7_LEAF 61 --
SlMLO8_LEAF 414 QILCSYVTLPLYALVTQM----------GSTM-----KPTIFNER----VAMALRKWH--
SlMLO9_LEAF 368 QVLCSYSTLPLYALVTQM----------GSHY-----KKSMFDNH----VQTCLVEWAEK
SlMLO9_FLOWER 431 QVLCSYSTLPLYALVTQM----------GSHY-----KKSMFDNH----VQTCLVEWAEK
SlMLO9_FRUIT 431 QVLCSYSTLPLYALVTQM----------GSHY-----KKSMFDNH----VQTCLVEWAEK
SlMLO10_LEAF 411 QFWCSFVTFPLYVIVAQM----------GSRY-----KKTIVSEN----VRTSLHGWRHK
SlMLO10_ROOT 411 QFWCSFVTFPLYVIVAQM----------GSRY-----KKTIVSEN----VRTSLHGWRHK
SlMLO10_FLOWER 411 QFWCSFVTFPLYVIVAQM----------GSRY-----KKTIVSEN----VRTSLHGWRHK
SlMLO10_FRUIT --
SlMLO11_LEAF 391 QVLCSYSTLPLYALVTQM----------GTTY-----KRGIFGEQ----TEDSLRIWAGT
SlMLO11_ROOT --
SlMLO11_FLOWER 391 QVLCSYSTLPLYALVTQM----------GTTY-----KRGIFGEQ----TEDSLRIWAGT
SlMLO11_FRUIT 391 QVLCSYSTLPLYALVTQM----------GTTY-----KRGIFGEQ----TEDSLRIWAGT
SlMLO12_FLOWER 418 QILCSYITLPLYALVTQM----------GSTM-----KRSIFDDQ----TSKALMNWH--
SlMLO13_LEAF --
SlMLO13_ROOT 409 QFWCSHSTVPLNVIISQM----------GSRC-----GKALVAES----VRDSLHSWCKR
SlMLO13_FLOWER 409 QFWCSHSTVPLNVIISQM----------GSRC-----GKALVAES----VRDSLHSWCKR
SlMLO13_FRUIT 409 QFWCSHSTVPLNVIISQM----------GSRC-----GKALVAES----VRDSLHSWCKR
SlMLO14_LEAF 409 QFLCSYSTLPLYALVTQM----------GTNY-----KAALIPQR----IRETIHGWGKA
SlMLO15_LEAF 345 HFLCGYVILPLYALLTQM----------GTKI-----KSSVLTDE----MISRLKRWQEK
SlMLO15_ROOT 52 --LFSD----------------
SlMLO15_FLOWER 69 ----SFX---
SlMLO15_FRUIT 71 ----SYSFLHPH---LQPWN--
SlMLO16_LEAF 376 QVLCSYITLPLYALVTQM----------GSHF-----KSALLEEH----ITQAIKHWHTE

```
SlMlo1_LEAF      415 -HTAKKRVKH--GL-SGHTTPANSRP---TTPLR-GTSPVHLLRGYPQYNEDSVQASPR-
SlMLO1_ROOT      415 -HTAKKRVKH--GL-SGHTTPANSRP---TAPLR-GTSPVHFLRGYPQYNEDSVQASPR-
SlMLO1_FLOWER    436 ------------------------------------------------------------
SlMLO2_LEAF      450 ARRRAVN--GSNQVEHKESTSLMSVQ---LAQVGTQESAMEEGNGGEISHANDQLNSKV-
SlMLO3_LEAF      456 -HTAKKHVKDQSK-HSNPVTPMSSRP---GTPSHHGMSPVHLLRGHYRSDMGSLQNSPRR
SlMLO4_LEAF      440 -----MAVKKKQVAKGDKS-IRTLGN---ASPRSSVGSPLHPSIGPTLHRFKTTGHSTRF
SlMLO5_LEAF      429 -QTAKQRAKD--GRPSKNASPVRSRA---VSPLR-G--------GSSPVQQKHGQLYPP-
SlMLO5_FLOWER    429 -QTAKQRAKD--GRPSKNASPVRSRA---VSPLR-G--------GSSPVQQKHGQLYPP-
SlMLO5_FRUIT     452 -QTAKQRAKD--GRPSKNASPVRSRA---VSPLR-G--------GSSPVQQKHGQLYPP-
SlMLO6_LEAF      437 -----MAVKKRTGARGDRSPTRTLGN---ASPRSAMSSPVHPS-GPGLHRYKTTGHSSRF
SlMLO6_ROOT      441 -----MAVKKRTGARGDRSPTRTLGN---ASPRSAMSSPVHPS-GPGLHRYKTTGHSSRF
SlMLO6_FLOWER    441 -----MAVKKRTGARGDRSPTRTLGN---ASPRSAMSSPVHPS-GPGLHRYKTTGHSSRF
SlMLO6_FRUIT     441 -----MAVKKRTGARGDRSPTRTLGN---ASPRSAMSSPVHPS-GPGLHRYKTTGHSSRF
SlMLO7_LEAF       61 ------------------------------------------------------------
SlMLO8_LEAF      453 -HSAKKHIKEINKQHSNPTTPMSSRP---PTPSH-GMSPVHLLRGIRTSDMDV---GPRR
SlMLO9_LEAF      409 VKKKKGHKYGRDGSTRSNDGSVVAAS---LS----------------VNDHKDLPQNGV-
SlMLO9_FLOWER    472 VKKKKGHKYGRDGSTRSNDGSVVAAS---LS----------------VNDHKDLPQNGV-
SlMLO9_FRUIT     472 VKKKKGHKYGRDGSTRSNDGSVVAAS---LS----------------VNDHKDLPQNGV-
SlMLO10_LEAF     452 VKTRLEGSVVSPETLLATTSLDSMAEDEVDQIHSVATI---------STEVFDESTVESE
SlMLO10_ROOT     452 VKTRLEGSVVSPETLLATTSLDSMAEDEVDQIHSVATI---------STEVFDESTVESE
SlMLO10_FLOWER   452 VKTRLEGSVVSPETLLATTSLDSMAEDEVDQIHSVATI---------STEVFDESTVESE
SlMLO10_FRUIT        ------------------------------------------------------------
SlMLO11_LEAF     432 SSHNKMVTETQESITVSNRPACNEIQ---------------DISSTVELSYPNKPHTTP-
SlMLO11_ROOT         ------------------------------------------------------------
SlMLO11_FLOWER   432 SSHNKMVTETQESITVSNRPACNEIQ---------------DISSTVELSYPNKPHTTP-
SlMLO11_FRUIT    432 SSHNKMVTETQESITVSNRPACNEIQ---------------DISSTVELSYPNKPHTTP-
SlMLO12_FLOWER   457 -----KNAKKKRPTKPGQIETRKLGS---PG-ESPETSPTKGGLGS---RRKLS------
SlMLO13_LEAF         ------------------------------------------------------------
SlMLO13_ROOT     450 VKDRSKHDALRSITTRSTCSLGSTI-DEGDEIATVASVTLSPCSSRGSFNHLDEKVLSND
SlMLO13_FLOWER   450 VKDRSKHDALRSITTRSTCSLGSTI-DEGDEIATVASVTLSPCSSRGSFNHLDEKVLSND
SlMLO13_FRUIT    450 VKDRSKHDALRSITTRSTCSLGSTI-DEGDEIATVASVTLSPCSSRGSFNHLDEKVLSND
SlMLO14_LEAF     450 ARRKRRMRMFPDDSTVHTDTSTVMSLEEYDNDQLVDS-PRTVHGAGTEIELQPPPTVTED
SlMLO15_LEAF     386 AK--RKLAKRSNYLLAQNLLSLNISPSFETSLDVTYLSTDTENDGEIVDDQRQIQQHTEF
SlMLO15_ROOT      56 ------------------------------------------------------------
SlMLO15_FLOWER       ------------------------------------------------------------
SlMLO15_FRUIT     84 ------------------------------------------------------------
SlMLO16_LEAF     417 VKRKKKN--KLQELESPHYSVATTKI---TSPEIKLSDSTHEIQEISEESPKVNIVHP--
```

```
SlMlo1_LEAF      466 ----------------TSNVENEGWANE-----------------NQEGEILQHASTDHN
SlMLO1_ROOT      466 ----------------TSNVENEGWANE-----------------NQEGEILQHASTDHN
SlMLO1_FLOWER    473 ------------------------------------------------------------
SlMLO2_LEAF      504 ------------------------------------------------------------
SlMLO3_LEAF      511 SNYD-FDHWDNEDSPSPSRFYQEAVDGSLHHIQLGQLDHELQQVIEPNSSQVVPLSQEGR
SlMLO4_LEAF      491 NGYDDLEASDLENDPTTPIIR-------------------------AEEHSTAHVTDHDD
SlMLO5_LEAF      473 -------------SPNPSRRRSGG--NP-----------------ESSSRQIFDDGSHEQ
SlMLO5_FLOWER    473 -------------SPNPSRRRSGG--NP-----------------ESSSRQIFDDGSHEQ
SlMLO5_FRUIT     496 -------------SPNPSRRRSGG--NP-----------------ESSSRQIFDDGSHEQ
SlMLO6_LEAF      488 QGYSDQEASDLENDPTTPMTR-------------------------AEI-ATTHI-DHDD
SlMLO6_ROOT      492 QGYSDQEASDLENDPTTPMTR-------------------------AEI-ATTHI-DHDD
SlMLO6_FLOWER    492 QGYSDQEASDLENDPTTPMTR-------------------------AEI-ATTHI-DHDD
SlMLO6_FRUIT     492 QGYSDQEASDLENDPTTPMTR-------------------------AEI-ATTHI-DHDD
SlMLO7_LEAF       61 ------------------------------------------------------------
SlMLO8_LEAF      505 SSYNNIDHWDIEGSPSPNRH-----DSEVH---------------EPNLSEI-----EAR
SlMLO9_LEAF          ------------------------------------------------------------
SlMLO9_FLOWER    512 ------------------------------------------------------------
SlMLO9_FRUIT     512 ------------------------------------------------------------
SlMLO10_LEAF     503 QRVPIEQSCTNE-------------ISECDEL----------HIP---------------
SlMLO10_ROOT     503 QRVPIEQSCTNE-------------ISECDEL----------HIP---------------
SlMLO10_FLOWER   503 QRVPIEQSCTNE-------------ISECDEL----------HIP---------------
SlMLO10_FRUIT        ------------------------------------------------------------
SlMLO11_LEAF     476 ------------------------------------------------------------
SlMLO11_ROOT         ------------------------------------------------------------
SlMLO11_FLOWER   476 ------------------------------------------------------------
SlMLO11_FRUIT    476 ------------------------------------------------------------
SlMLO12_FLOWER   499 ------QVEPLSSDQT------------------------------ANIVAS--------
SlMLO13_LEAF         ------------------------------------------------------------
SlMLO13_ROOT     509 HQEDCIVETTNQPGHELSFRNSEVLVTDAEEIVDDE----ADKIE---------------
SlMLO13_FLOWER   509 HQEDCIVETTNQPGHELSFRNSEVLVTDAEEIVDDE----ADKIE---------------
SlMLO13_FRUIT    509 HQEDCIVETTNQPGHELSFRNSEVLVTDAEEIVDDE----ADKIE---------------
SlMLO14_LEAF     509 HHHSVTINDTSSRIGTPLLRPCASISSATSPTLPPEVISRSSSMP---------------
SlMLO15_LEAF     444 GSFGGFHLSK--------------------------------------------------
SlMLO15_ROOT      56 ------------------------------------------------------------
SlMLO15_FLOWER       ------------------------------------------------------------
SlMLO15_FRUIT     84 ------------------------------------------------------------
SlMLO16_LEAF     470 ----------------------------------------------------------NE
```

```
SlMlo1_LEAF       493 KQIEITM----------SDFTFGNK--X--
SlMLO1_ROOT       493 KQIEITM----------SDFTFGNK--X--
SlMLO1_FLOWER     481 ------------------------------
SlMLO2_LEAF       504 -----------------------------N
SlMLO3_LEAF       570 DQHEITIAGS-------RDFSFEKRTTSI-
SlMLO4_LEAF       526 TELQVHVPQNEESSGNEDDFSFAKPAP---
SlMLO5_LEAF       501 SEIEITL----------NDLSLENKLSX--
SlMLO5_FLOWER     501 SEIEITL----------NDLSLENKLSX--
SlMLO5_FRUIT      524 SEIEITL----------NDLSLENKLSX--
SlMLO6_LEAF       521 TEIHVHIPQNGESTRNEDDFSFVKPAPQRX
SlMLO6_ROOT       525 TEIHVHIPQNGESTRNEDDFSFVKPAPQRX
SlMLO6_FLOWER     525 TEIHVHIPQNGESTRNEDDFSFVKPAPQRX
SlMLO6_FRUIT      525 TEIHVHIPQNGESTRNEDDFSFMKPAPQRX
SlMLO7_LEAF        61 -----------------------------S
SlMLO8_LEAF       540 EQYEINIARS-------RDFSFDKRTTSVX
SlMLO9_LEAF           ------------------------------
SlMLO9_FLOWER     512 -----------------------------X
SlMLO9_FRUIT      512 -----------------------------X
SlMLO10_LEAF      525 --------------------LSPRNHGEVX
SlMLO10_ROOT      525 --------------------LSPRNHGEVX
SlMLO10_FLOWER    525 --------------------LSPRNHGEVX
SlMLO10_FRUIT         ------------------------------
SlMLO11_LEAF      476 -----------------------------X
SlMLO11_ROOT          ------------------------------
SlMLO11_FLOWER    476 -----------------------------X
SlMLO11_FRUIT     476 -----------------------------X
SlMLO12_FLOWER    515 ----VDIPXEKPPS-NPDLLTGLM------
SlMLO13_LEAF          ------------------------------
SlMLO13_ROOT      550 --------------------TLFELFQKTX
SlMLO13_FLOWER    550 --------------------TLFELFQKTX
SlMLO13_FRUIT     550 --------------------TLFELFQKTX
SlMLO14_LEAF      554 --------------------ARGHLNLDKQ
SlMLO15_LEAF      454 -----------------------SRAAQHX
SlMLO15_ROOT       56 -----------------------------L
SlMLO15_FLOWER        ------------------------------
SlMLO15_FRUIT      84 -----------------------------T
SlMLO16_LEAF      472 SQIGVD------------------------
```
