# Supplementary material for: Genome-Wide Study of the Tomato SlMLO Gene Family and Its Functional Characterization in Response to the Powdery Mildew Fungus Oidium neolycopersici
Source: Front Plant Sci. 2016 Apr 6;7:380. doi: 10.3389/fpls.2016.00380 (PMC4986958; doi:10.3389/fpls.2016.00380)
Supplement: Supplementary file 3 [file DataSheet3.pdf]

Genome-wide study of the tomato *SlMLO* gene family and its functional characterization in response to the powdery mildew fungus *Oidium neolycopersici*

Supplementary material

Supplementary Fig. 1 (.pdf) Sequence alignments of *SlMLO* homologs cloned from different tissues of the tomato cv. Moneymaker showing deviating transcripts when compared with the predicted CDS sequence, as inferred by the SGN database, against their respective genomic regions. Deviating transcripts are identified for *SlMLO1* in flower, *SlMLO5* in fruit, *SlMLO6*, *SlMLO7*, *SlMLO8* and *SlMLO9* in leaf, *SlMLO10* in fruit, *SlMLO11* in root, *SlMLO13* in leaf and *SlMLO15* in all the tissues investigated. Alignments were obtained with default parameters by the CLC sequence viewer software. This legend corresponds to the file named Data Sheet 1 that can be downloaded on the right-hand side of the article abstract.

Supplementary Fig. 2 (.pdf) Sequence alignment of a set of 37 MLO proteins cloned from four tissues (leaf, root, flower and fruit) of the cv. Moneymaker. When no deviating transcripts were detected, the translated nucleotide sequence of leaf was used in the alignment, with the exception of *SlMLO12* which was cloned only from flower. Shading was obtained by feeding the alignment, obtained with default parameters by the CLC sequence viewer software, to the online server BoxShade (http://sourceforge.net/projects/boxshade/) and setting as 0.7 the fraction of sequences that must agree for shading. In black shade, the amino acids fully conserved, in gray the amino acids substitutions which are predicted to have similar biochemical properties and in red the amino acids identified by Elliot et al., 2005 as invariable for the whole MLO protein family. Transmembrane domains are indicated as inferred by the TMHMM online server (http://www.cbs.dtu.dk/services/TMHMM/) fed with the SlMLO1 protein sequence of leaf. This legend corresponds to the file named Data Sheet 2 that can be downloaded on the right-hand side of the article abstract.

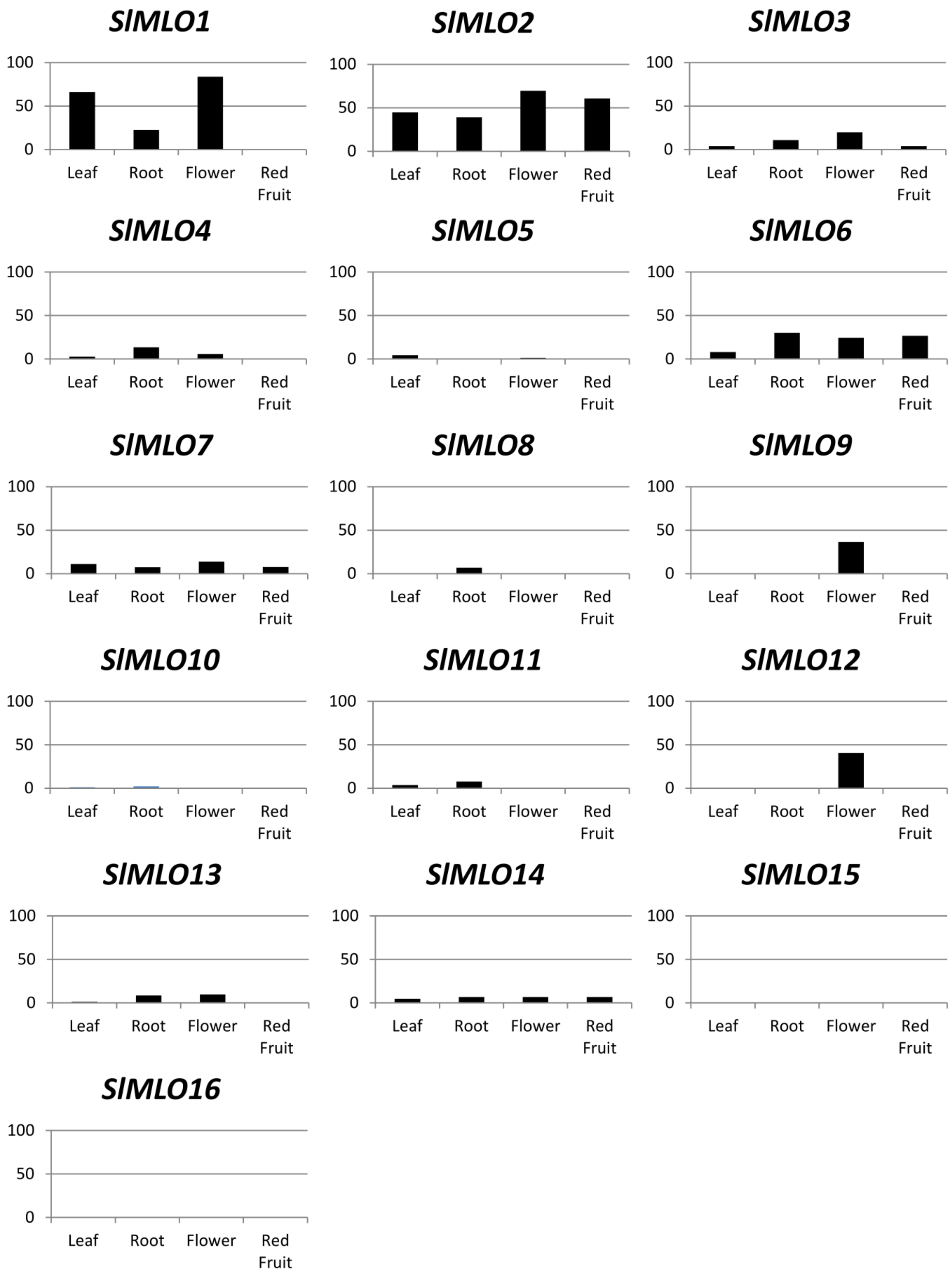

Supplementary Fig. 3 FPKM values (fragments per kilobase of exon per million fragments mapped) depicted as columns for each of the four tissues investigated in this study obtained from the RNA-seq data of each of the 16 *SlMLO* homologs as reported by the SGN database.

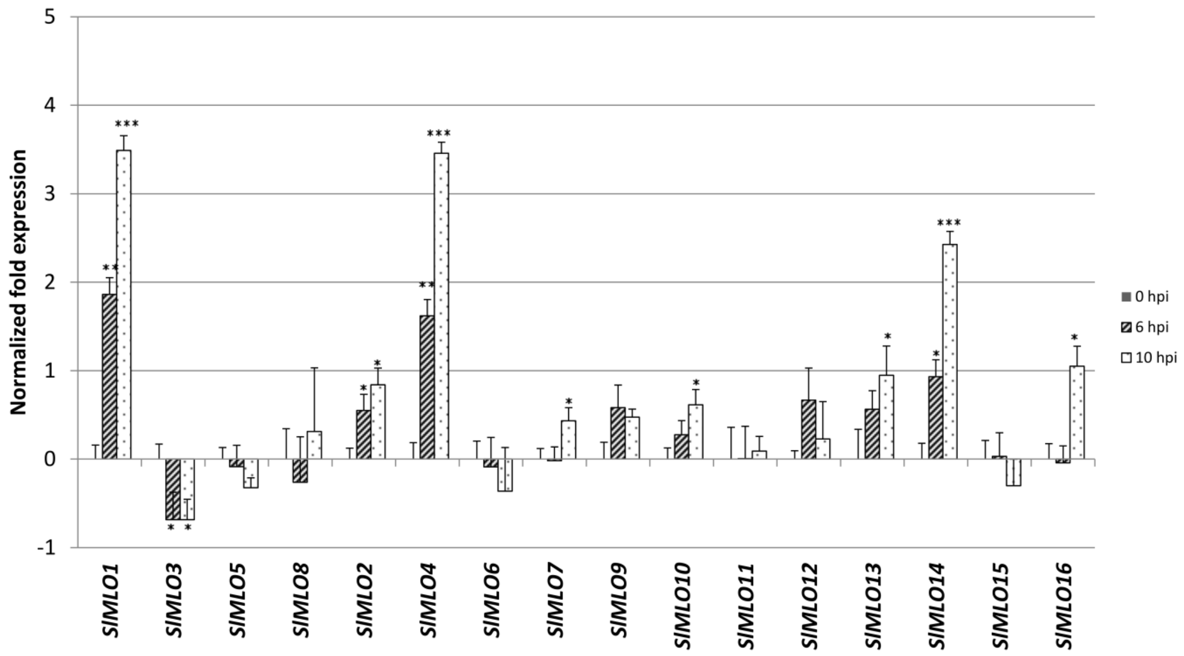

Supplementary Fig. 4 Relative expression level of the *SlMLO* gene family members in response to *O. neolycopersici* infection. Samples were collected at 0, 6 and 10 hrs after inoculation (hpi). Transcript levels of each *SlMLO* homolog were normalized against the transcription level of Ef1α as reference gene. Bars show standard errors based on four biological replicates. Asterisks refer to significant differences with respect to non-inoculated plants (0 hpi), inferred by mean comparisons with a Student's t-test (* $p < 0.05$, ** $p < 0.01$, *** $p < 0.001$).

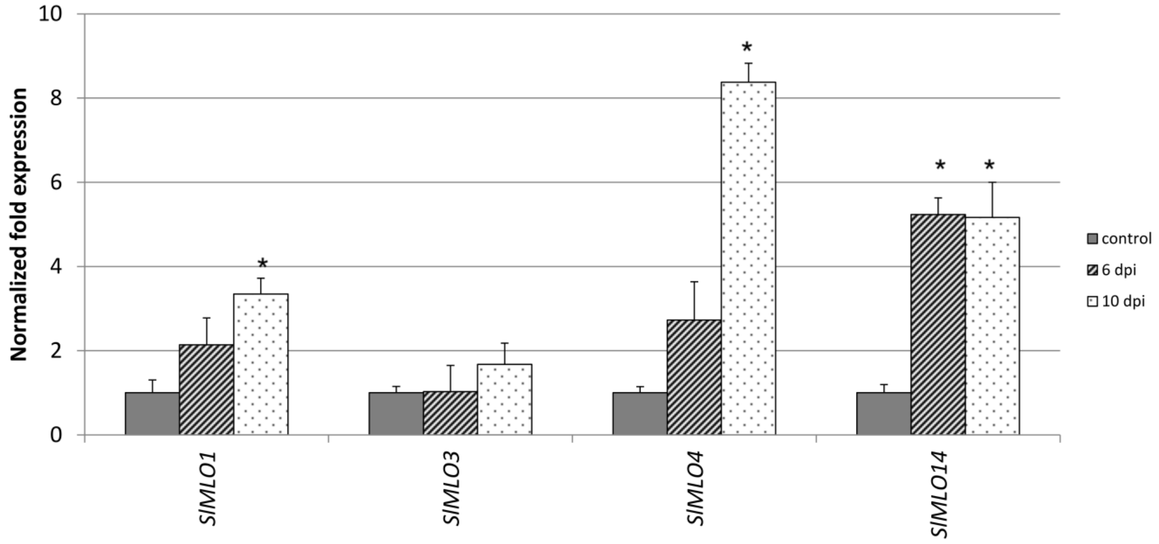

Supplementary Fig. 5 Relative expression level of *SlMLO1*, *SlMLO3,* *SlMLO4* and *SlMLO14* compared to the reference gene Ef 1α upon inoculation with *O. neolycopersici*. Means are calculated from duplicate technical measurements. Bars show standard errors based on three biological replicates. Asterisks refer to significant differences with respect to non-inoculated plants used as control (0 hpi), inferred by mean comparisons with a Student's t-test (* $p < 0.05$).

```
SlMlo3    1 ATGGCCGGTGGTGGTGGTGGTGGAAGAT------------CGTTGGAGCAAACGCCAACT
SlMlo8    1 ATGGCGGGAGGAGGAGGAGG---AAGAT------------CGTTGGAGCAAACGCCGACG
SlMlo1    1 ATGG------------------------------------------AGGCAACCCCTACG
SlMlo5    1 ATGGCTAGCACAGGCTGTATTAGAACGTGTGATGAACGTCCTCTAGATGAGACACCAACA

SlMlo3   49 TGGGCGGTTGCCGTTGTTTGTTTTGTGTTAGTTGCAATATCTATTGTCATTGAACATATT
SlMlo8   46 TGGGCGGTTGCCCTAGTTTGTTTTGCATTGGTTGCCATTTCCATTGTAATAGAGCTCATC
SlMlo1   19 TGGGCAATTGCTGTGGTTTGCTTCATCTTGCTCGCTATTTCTATTTTTATTGAACAAATT
SlMlo5   61 TGGGCTGTAGCCATGGTTTGCTTTGTATTAGTTGTAATCTCCCTTTTCATTGAACAACTT

SlMlo3  109 ATCCACCTTATTGGAAAGTGGTTGAAGTCTAAAAATAAAAGTGCCTTGTATGAAGCACTT
SlMlo8  106 ATCCATCTTATTGGCAAGTGGTTGAAGTCTAAACATAAAAGAGCATTATATGAAGCACTT
SlMlo1   79 ATTCATCACATTGGAGAGTGGTTACTGGAAAAGCGGAAAAAGTCTCTATATGAAGCACTT
SlMlo5  121 ATTCATCATCTTGGAGAGTGGTTATGGAAGAAACAAAAGAGACCATTGTATGAAGCACTT

SlMlo3  169 GAAAAGATCAAAGCAGAGCTTATGCTGCTGGGATTCATATCATTGTTGTTAACAGTAGGA
SlMlo8  166 GAGAAGATAAAAGCAG---------------------------------------TAGGG
SlMlo1  139 GAAAAGATCAAAGCTGAACTTATGCTGTTGGGATTCTTATCACTGTTGTTGACAGTGTTG
SlMlo5  181 GAGAAGATCAAGTCAGAACTCATGTTATTAGGGTTTATATCCTTATTCTTGACGGTTGTA

SlMlo3  229 CAAAGTCCAATTTCGAACATATGTGTATCAGAAAAATTAGGAAATTCATGGCATCCATGT
SlMlo8  187 CAAGATCCAATTTCAAATATTTGTGTATCTGAAAAAATTGCAAGTACGTGGCATCCATGT
SlMlo1  199 CAAGATCCAGTTTCTAACTTATGTGTCCCCAAGAGTGTTGGTTATTCATGGCATCCTTGT
SlMlo5  241 CAGGATCCTATGTCTAAGATATGTATTCCTAGGAGTGTTGGACGCTCTTGGCATCCATGT

SlMlo3  289 AGTAAAAAAGAAGAAGATAGTA-------GTATAATTTCAGAAGATTCGTTGTCGGAGCA
SlMlo8  247 AGTAAACAAAAAGAAGCTGAAATGAACAAATATATTTCCGGTGACTT-------AGAGGG
SlMlo1  259 ATGGCAAAGGAAGATGCCAAG---------------------------------------
SlMlo5  301 GACATAAACAAACATATTGAT---------------------------------------

SlMlo3  342 ACACCGCCGGAGACTTCTTATGGATGCTGCCGGCGGTGGCGTACGACGAATATTGGCTGG
SlMlo8  300 TCATCGCCGGCGACTTTTCACGG---CTGACGATGGCGGAGTCCGGCGAGTTTTGGCGGC
SlMlo1  280 --------------------------------------------TCTGAGTAT-------
SlMlo5  322 --------------------------------------------GACCAATAT-------

SlMlo3  402 TGGTGGTGGAGATGACAAATGTGCAGCAAAGGGAAAAGTACCATTTGTGTCTGCTGATGG
SlMlo8  357 TGCCGG---AACTGACAAATGTGCAGACAAGGGAAAAGTAGCATTTGTGTCTGCTGATGG
SlMlo1  289 ----------GATGACCCTTGTCTACCAAAGGGAAAAGTGCAATTTGCATCTTCATATGC
SlMlo5  331 ----------CTCGATCCATGTAGAATTAAGGGGAAACTCCAATTTGCTTCAAAATATGC

SlMlo3  462 AATTCATCAATTACACATTTTCATCTTTGTGCTGGCTGTATTTCATGTCCTCTATTGTGT
SlMlo8  414 TATTCATCAATTACATATTTTCATTTTTGTGCTGGCTATTTTTCATGTATTTTATTGTGT
SlMlo1  339 AATACACCAGCTCCATATCTTCATCTTTGTATTGGCAGTTGCTCATGTATTGTACTGTAT
SlMlo5  381 AATTCACCAACTCCACATTTTTATCTTTGTGTTAGCCGTTGCACATGTGTTGTATTGTAT

SlMlo3  522 TACAACTTTGGCTTTGGGGAGAGCTAAGATGAGAAGTTGGAAGTCATGGGAAAATGAAAC
SlMlo8  474 TACCACATTGGCATTGGGAAGAGCTAAGATGAGTCGTTGGAAGATATGGGAAAAGGAAAC
SlMlo1  399 AGCAACTTTTGCTTTGGGCAGGCTAAAGATGAGAAAATGGAGGGCATGGGAGGATGAAAC
SlMlo5  441 TACCACTTTGGGAATTGGCAAACTAAGGATGAGGACATGGAGAGCTTGGGAGGATGAATC

SlMlo3  582 TAAAACAGCTGAATACGAATTCTCTCACGATCCTGAAAGATTTCGATTTACAAGAGAAAC
SlMlo8  534 AAGAACAGCTGAGTACCAATTTTCTCATGATCCAGAGAGATTTCGATTTGCTAGAGATAC
SlMlo1  459 AAAAACAATGGAGTACCAATTCTACAACGACCCTGAGAGATTCAGATTTGCAAGGGAGAC
SlMlo5  501 TAAAACAATTGAATACCAATTCTATAACGATCCTGAGAGATTTAGATTTGCAAGAGAAAC
```

```
SlMlo3   642 ATCATTTGGAAGAAGACACTTGAGCTTTTGGACCAAAAATCCTGTCCTACTTTGGATCGT
SlMlo8   594 ATCATTTGGAAGAAGACATTTGAGTTTTTGGACTAAAAATTCAGTTCTTCTATGGATTGT
SlMlo1   519 CTCGTTTGGACGTAGGCATTTGCATTTCTGGAGCAAGTCCCCCGTGTTGCTCTCGATAGT
SlMlo5   561 ATCATTTGGACGTAAACATTTGCATTTCTGGAGCAACTCTCCAATTCTTCTCTGGATAGT

SlMlo3   702 TTGTTTCTTTAGGCAATTTGTAAGATCTGTCCCAAAAGTTGATTACTTAACACTTAGGCA
SlMlo8   654 TTGTTTCTTCAGGCAATTTGTAAGATCTGTTCCAAAAGTTGATTATTTGACCCTACGACA
SlMlo1   579 TTGTTTCTTTCGGCAATTCTTCTCATCAGTTGCAAAAGTTGACTATTTAACCCTTAGACA
SlMlo5   621 TTGTTTCTTCAGACAGTTCTATGCATCAGTTGAAAAAGTAGACTATCTTACCCTTAGACA

SlMlo3   762 TGGATTTATTACGGCACATTTAGCACCACAGAGCCACCAAAAATTTGATTTCCGAAAGTA
SlMlo8   714 TGGTTTTATCACG-----------------------------------------------
SlMlo1   639 TGGGTTCATGATGGCACATTTAACTCCACAAAATCAAAATAATTTTGATTTTCAATTATA
SlMlo5   681 TGGCTTTGCTATGGCACATTTAGCACCTCAGCAAGAAAAGAATTTTGATTTTCAATTGTA

SlMlo3   822 CATTAAACGATCACTTGAAGAAGATTTTAAAGTGGTTGTAGGAATCAGTCCCCCAATATG
SlMlo8   727 ------------------------------------------------TCCACCAATTTG
SlMlo1   699 CATTAACAGAGCAGTTGACAAAGACTTCAAAGTTGTTGTTGGAATAAGTCCTGCATTATG
SlMlo5   741 TATAAATAGAGCACTTGAAGAAGATTTTAAAGATGTTGTGGGAATAAGTCCACTGTTATG

SlMlo3   882 GTTCCTCGCCGTGCTCTTCTTACTCTTTAATACTCATGGCTGGTATTCCTATCTTTGGCT
SlMlo8   739 GTTCCTTGC-------------------------------TGGTATTCTTATCTGTGGCT
SlMlo1   759 GCTCTTCACGGTGCTATATTTTCTGACTACTACCGATCGATTGTACTCGTATCTTTGGGT
SlMlo5   801 GATGTTTGCAGTCCTCTACTTTCTCACTACTACCAATGGTTGGTATTCATACTATTGGCT

SlMlo3   942 ACCATTCATCCCCTTGATTGTGATATTATTAGTAGGGACAAAGCTACAAGTGATAATAAC
SlMlo8   768 ACCGTTCATTCCGTTACTTGTGATATTATTAGTAGGGACTAAACTACAAGTGATTATAAC
SlMlo1   819 GCCATTTATCCCACTTGTAATAATATTGCTAGTTGGCACAAAACTTCAAATGATCATAAC
SlMlo5   861 GCCGTTCATTCCTTTAATTATAATATTACTGGTGGGCACAAAATTACAAGTGATTATAAC

SlMlo3  1002 AAAAATGGGGCTAAGGATTCATGAAAGAGGAGAAGTAGTAAAAGGGGTACCTGTGGTTCA
SlMlo8   828 AAAAATGGGATTAAGAATTCAAGAAAGGGGAGAAGTAGTGAAAGGGGTACCTGTGGTTCA
SlMlo1   879 AGAAATGGGAGTAAGGATTTCAGAAAGGGGAGACATAGTAAAAGGTGTACCTGTGGTGGA
SlMlo5   921 AAAAATGGGATTAAGGATTAAAGAAAGAGGAGACATTGTTAAAGGAACACCATTAGTTGA

SlMlo3  1062 GCCAGGAGATCACCTTTTTTGGTTCAACCGTCCTCGTCTCATTCTTTATCTTATTAACTT
SlMlo8   888 ACCTGGAGATGATTTATTTTGGTTTAATCGTCCTCGTCTACTACTTTTTTTAATTAATTT
SlMlo1   939 GACTGGTGACCATCTTTTCTGGTTTAATCGCCCTGCCCTTGTCCTATTCTTGATTAACTT
SlMlo5   981 ACCAGGGGATGATCTTTTCTGGTTTAATCGTCCTGATCTTTTGCACTTCTTCATTCACTT

SlMlo3  1122 TGTTCTCTTTCAGAATGCTTTTCAGTTGGCCTTCTTTGCTTGGACTTGGTATGAATTTGG
SlMlo8   948 TGTTCTTTTTCAGAATGCTTTTCAATTGGCTTTCTTTGCTTGGACTTGGTATGAATTTGG
SlMlo1   999 TGTACTCTTTCAGAATGCGTTTCAAGTTGCTTTCTTTTTTTGGAGTTGGTGGAAATTTGG
SlMlo5  1041 TGTTCTCTTTCAGAATGCATATCAACTTGCTTTCTTTGCTTGGAGCTGGTGGAAATTTAA

SlMlo3  1182 GTTGAAATCTTGTTACCATGACCATACTGAGGATATTGTCATCAGAATTACTATGGGGGT
SlMlo8  1008 GCTGAAATCTTGTTTCCATGACCAAACTGAGGACATCGTAATTAGAATGACAATGGGGGT
SlMlo1  1059 TTTCCCATCTTGCTTTCATAAGAATGCTGCAGACCTAGCCATAAGGCTAACCATGGGGGT
SlMlo5  1101 TTTACCATCTTGCTTCCACAAAAATGTAACAGACATAGCCATAACACTTTCCATGGGGGC

SlMlo3  1242 CCTCATTCAGATTCTGTGCAGTTATGTCACTCTTCCTCTTTATGCCCTTGTGACACAGAT
SlMlo8  1068 TCTTATTCAGATTCTTTGCAGCTATGTTACTCTTCCATTATATGCTCTTGTAACACAGAT
SlMlo1  1119 GATCATACAGGTCCATTGCAGCTATGTGACTCTCCCTCTTTATGCCTTAGTTACACAGAT
SlMlo5  1161 TCTCATTCAAGTTCTTTGCAGCTACGTGACACTCCCCCTATATGCCTTAGTTACTCAGAT
```

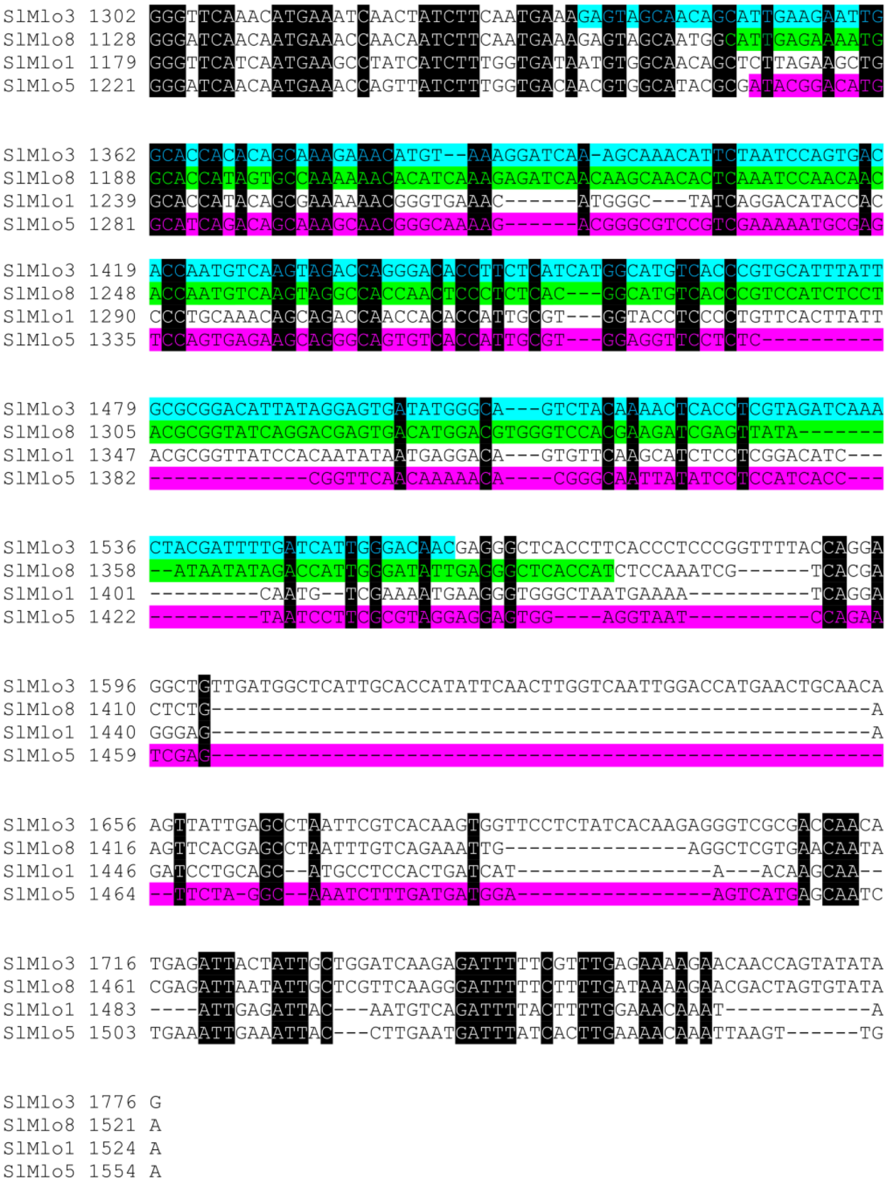

Supplementary Fig. 6 Sequence alignment of the coding sequences of the four *SlMLO* homologs found in clade V, namely *SlMLO1*, *SlMLO3*, *SlMLO5*, and *SlMLO8*. The alignment was generated by CLC sequence view software using default parameter and given to BoxShade online server (http://sourceforge.net/projects/boxshade/) to allow the shading. The fraction of sequences that must agree for shading was set as 1. Black shade indicates nucleotides that are fully conserved among the four sequences. Regions of each homolog used for the RNAi construct are highlighted in yellow, light blue, green and pink for *SlMLO1*, *SlMLO3*, *SlMLO8*, and *SlMLO5* respectively. Primer pairs used to prepare each RNAi construct are listed in Supplementary Table 3.

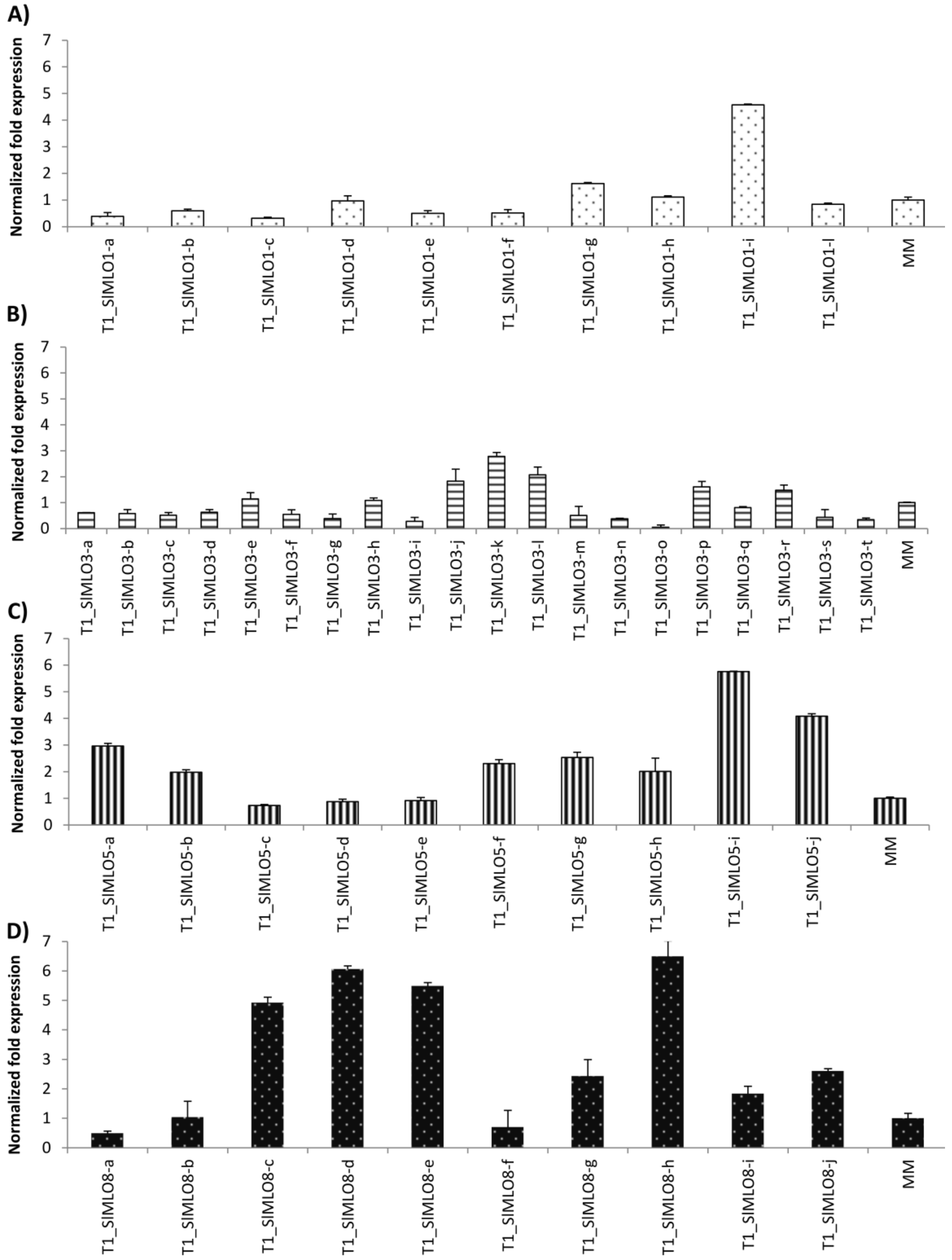

Supplementary Fig. 7 Relative expression level of the target *SlMLO* gene in T_1 plants obtained with the RNAi silencing construct for *SlMLO1* (panel A), *SlMLO3* (panel B), *SlMLO5* (panel C) and *SlMLO8* (panel D), respectively. Bars shows standard error of three technical replicates.

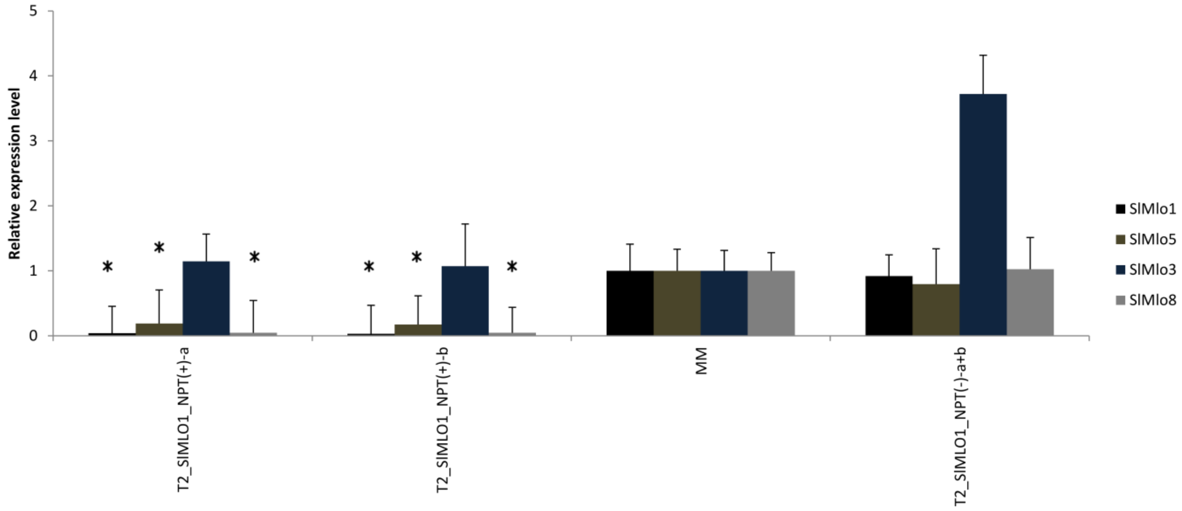

Supplementary Fig. 8 Relative expression level of the clade V *SlMLO* genes in two T_2 families segregating for the RNAi construct designed to target *SlMLO1*. Bars show standard errors of four transgenic individuals T2_SlMLO1_NPT2(+)-a, four transgenic individuals of the T2_SlMLO1_NPT2(+)-b, four Moneymaker (MM) and four non-transgenic T2_SlMLO1_NPT2(-)-a+b plants (from both T_2 families). Asterisks refer to significant differences (* $p < 0.05$) with respect to the expression of the target gene in MM that was used as genetic background for the transformation.

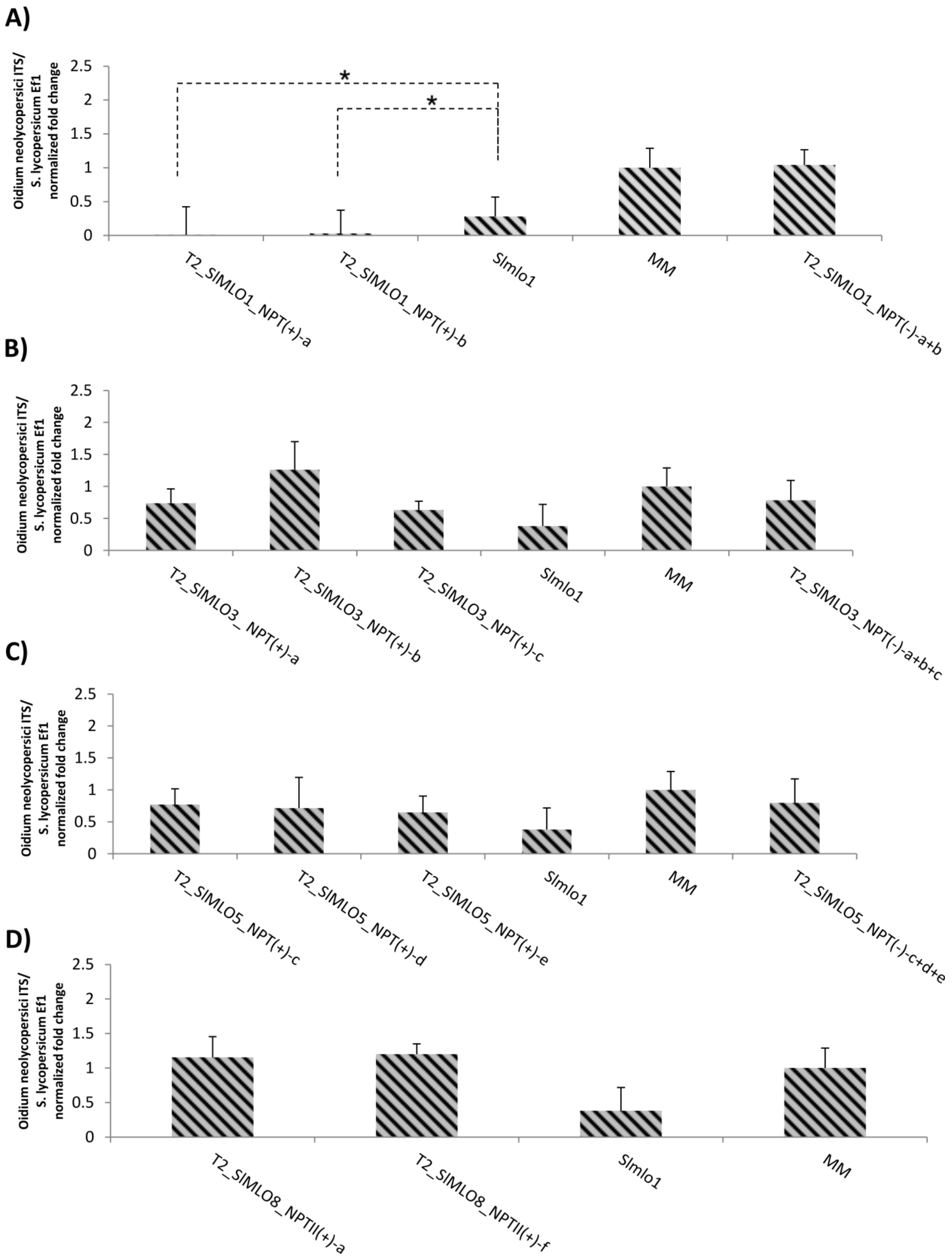

Supplementary Fig. 9 Fungal biomass measured by relative quantification of the ratio between *Oidium neolycopersici* and plant gDNAs on independent T_2 families obtained with the silencing constructs targeting *SlMLO* genes of clade V. Panel A and panel D refer to the two T_2 families tested in this study obtained with the constructs designed to silence *SlMLO1*

and *SlMLO8*, respectively. Panel B and panel C refer to the three T_2 families obtained with the constructs designed to silence *SlMLO3* and *SlMLO5*, respectively. The *SlMLO* gene name and extension “a” to “f” of the T_2 families refer to the ones in Supplementary Fig. 7.

For MM and Slmlo line in each panel, bars show standard errors based on 10 plants. In panel A, bars show standard errors of four transgenic plants (NPT+) for each of the two T_2 families (T2_SlMLO1-a and -b) and four plants that do not carry the silencing construct obtained from the two T_2 families. Asterisks refer to significant differences compared to the Slmlo1 line (* $p < 0.05$; Student’s t-test). In panel B, bars show standard errors of 10 transgenic plants of the T2_SlMLO3-a and -b families, eight plants of the T2_SlMLO3-c family and seven plants that do not carry the silencing construct obtained from the three families. In panel C, bars show standard errors of nine transgenic plants for each of the three T_2 families (T_2_SlMLO5-c, -d and -e) and six plants that do not carry the silencing construct obtained from the three families. In panel D, bars show standard errors of 10 transgenic plants for each of the two T_2 families (T2_SlMLO8-a and –f). For these two T_2 families, we did not find plants without the NPTII marker.

Supplementary Table 1. **Primers pairs used to amplify and clone *SlMLO* homologs**. Primer pairs A and B were used to amplify overlapping fragments at the 5’ and the 3’ end of each gene, respectively. Successful amplifications were followed by assembly of the two products to obtain full-length sequences, which were submitted to the NCBI database and accession numbers are reported. Unsuccessful amplifications are indicated by the symbol (−).

SlMLO	Product A (bp)/ product B (bp)	Primer pair A(5’…3’)	Primer pair B (5’…3’)	Leaf	Root	Flower	Fruit
SlMLO1	925 / 753	TTGACATTTCCCCTTCTTCTTA/ CCCTTTCTGAAATCCTTACTCC	TATTGCTAGTTGGCACAAAACT/ TACAAAATCATTGCCATTTGAA	KU759512	KU759511	KU759510	−
SlMLO2	800 / 931	GAATCAACATAGTGTTTTTCATCTT/ TCTAATTTTGTGACAGATCCAAAG	CGGTTTCTAGGATTTGGTAAAA/ TCCTTTACAGTCATAACAGCTTTG	KU759516	KU759515	KU759513	KU759514
SlMLO3	1089 / 824	TCTGAGAAACAAGTTTTGAGTGA/ AACCACAGGTACCCCTTTTACT	AATAACAAAAATGGGGCTAAGG/ GAATATAGTATTAATGTGCTCCTTGTT	KU759519	KU759518	−	KU759517
SlMLO4	1016 / 954	GCTTTCTCTGCATCTGAAAGTT/ AGATGTCAAGAGCCATTCTTGT	TTCCAAAAGTATATCAAAAGGTCAT/ GAAGAAGCAAGCCACTATTCAG	KU759520	−	−	−
SlMLO5	852 / 916	CTCTCACAAATTTCCCTTATCATTA/ TAACCTTATTCCCACAACATCTTTA	CAAGAAAAGAATTTTGATTTTCAAT/ ATTTCAAAGGTAAACACGTACAAGA	KU759523	−	KU759521	KU759522
SlMLO6	402 / 311	TTCTGTTTCAAGAAGATGATGTAAA/ CAATATCACGAAATTCTTCCTC	TGCCATTAACTTATGATTTCCA/ TGTCTAAGGTTGTGGATTCTGT	KU759527	KU759526	KU759524	KU759525
SlMLO7	596 / 971	CATTTGTGAAGAAGAAGAAGAGTTT/ CACGAATCATGTACTTGTGAAAAT	TTCTTTAAGCAATTTTATGGTTCTG/ GAATATGGTTCCTCAGAAGAGTTG	KU759528	−	−	−
SlMLO8	978 / 998	TCTCATTAATTTCAATTTACGCTACG/ TAACGGAATGAACGGTAGCC	TCCAGAGAGATTTCGATTTGC/ AAGTGACTTTGCCACTCATGATA	KU759529	−	−	−
SlMLO9	730 / 1077	CATATTTGCCTAATCCATGAAA/ GCTCCTCCAAATACAATAGTCAA	TTGCATCACCTTCATGTTTTTA/ TGGTATACTGGATCATACCTAACTTTT	KU759532	−	KU759530	KU759531
SlMLO10	857 / 1050	TGATACATCTGAAGCTGAAATACTG/ CCTCTTGATGTACTTTTGGAAGTTA	TTTTTCAAGTCTGTCAGTAAGTCAG/ ACATTGATATGACTTGAGGGAAAAG	KU759536	KU759535	KU759533	KU759534
SlMLO11	511 / 850	TGTCTTTAATTCATCAAGACATGC/ ACCTCTTGAGCCAATTCTGTTA	CTTCTAGTAGGAACAAAATTGGAG/ TTTAAAGAATAATGTATTGTTCATCCT	KU759540	KU759539	KU759537	KU759538
SlMLO12	972 / 982	TTCCCTTTTTCCCTCACTGAA/ AGGTGCTAAATGGACCGAGA	TTTCAGGTCAGTTAGGAAAGCTG/ GATGACACAGCCAATCTCCA	−	−	KU759541	−
SlMLO13	833 / 853	GAGATGAAGATCCCATTTGGT/ GTCGTAATAGTATCTTCGGTTTTCC	GAAGGGTCCACTTATTGGATTAC/ ACAGAATAAATACTGTGTAGCATGA	KU759545	KU759544	KU759542	KU759543
SlMLO14	810 / 780	TTTGAAGTTCTGGTAGATTAGTTGG/ GTAATTCCAGCACTCTCCAATG	ACTACTCGTGGGTACAAAACTGC/ GAAGTATCAACACCAACTTCACTTC	KU759546	−	−	−
SlMLO15	910 / 750	AATAAAGAGAGATCTTTACCCCTGA/ CATGCCGGAGAGTTAAGTAGTCT	TGAGGCAATTTTATGGATCAGTAT/ GAAGTTTCAAAAGATGGTGAAATA	KU759550	KU759549	KU759547	KU759548
SlMLO16	940 / 946	TCGTCTGTGATTCATTCAACCT/ GGCCACAATTGTCTCCAACT	GATCCCTCAGCTTTGGATCA/ CCTACCAAACAACCCCAAGT	KU759551	−	−	−

Supplementary Table 2. Protein sequences used for the phylogenetic analysis of Fig. 1 of this study.

Species	***Protein abbreviation***	***Accession number NCBI database***
Arabidopsis thaliana	AtMLO1	AEE82203
A. thaliana	AtMLO2	Q9SXB6
A. thaliana	AtMLO3	Q94KB9
A. thaliana	AtMLO4	AEE28674
A. thaliana	AtMLO5	AEC08867
A. thaliana	AtMLO6	Q94KB7
A. thaliana	AtMLO7	AEC06624
A. thaliana	AtMLO8	AEC06633
A. thaliana	AtMLO9	AEE31927
A. thaliana	AtMLO10	AED98133
A. thaliana	AtMLO11	AED96404
A. thaliana	AtMLO12	AEC09645
A. thaliana	AtMLO13	AEE84878
A. thaliana	AtMLO14	AEE30722
A. thaliana	AtMLO15	AEC10377
Pisum sativum	PsMLO1	AGJ01118
Capsicum annuum	CaMLO2	AFH68055
Lotus japonicus	LjMLO1	AAX77015
Medicago truncatula	MtMLO1	ADV40949
Triticum aestivum	TaMLO_B1	AAK94904
Triticum aestivum	TaMLO_A1b	AAK94905
Hordeum vulgare	HvMLO	P93766
Oriza sativa	OsMLO2	AAK94907
Nicotiana tabacum	NtMLO1	AIT98396
Cucumis sativus	CsaMLO8	XP_004142393
Malus domestica	MdMLO18	XP_008383677
Malus domestica	MdMLO20	XP_008390437
Prunus persica	PpMLO9	XP_007211843
Prunus persica	PpMLO13	XP_007212960
Fragaria vesca	FvMLO13	XP_004308899
Fragaria vesca	FvMLO15	XP_004293900

Supplementary Table 3. Primer pairs used for qPCR and for RNAi constructs preparation.

Label	Size PCR product	Fw primer sequence (5'-3')	Rev primer sequence (5'-3')
L33	138 bp	GGGAAGAGGCTGGGATACATC	AGGAGGCAAATTGGACTTGAAC
Ef1α	120 bp	ACAGGCGTTCAGGTAAGGAA	GAGGGTATTCAGCAAAGGTCTC
Ubi	134 bp	GGACGGACGTACTCTAGCTGAT	AGCTTTCGACCTCAAGGGTA
Act	159 bp	GGAATAGCATAAGATGGCAGACG	ATACCCACCATCACACCAGTAT
*SlMLO*1	128 bp	CTTTGGGCAGGCTAAAGATG	AATGCCTACGTCCAAACGAG
*SlMLO*2	145 bp	AGCTGTCAATGGCTCAAACC	TGGAGTTGAGTTGGTCGTTC
*SlMLO*3	93 bp	TCAGTCCCCCAATATGGTTC	TCAAGGGGATGAATGGTAGC
*SlMLO*4	144 bp	AGATGGGCTCCAACATGAAG	AGGACCTTGGACTTGCATTG
SlMLO5	91 bp	GCTTTCTTTGCTTGGAGCTG	CCCCCATGGAAAGTGTTATG
SlMLO6	95 bp	TACATGGATGGCGACACAAG	CGCCATGGAGTCTAATGATG
SlMLO7	128 bp	ATACGTCAACGGAAGCACTG	CCATACCCACAAATCGATCC
SlMLO8	108 bp	ATTGCAAGTACGTGGCATCC	ATCGTCAGCCGTGAAAAGTC
SlMLO9	70 bp	GGGCTGAGAAGGTGAAAAAG	ACCGTCATTTGAACGGGTAG
SlMLO10	136 bp	AACAACACCGATGACTCGTG	AGGAGCAGGCTTCACAAATG
SlMLO11	93 bp	ATGCCCTATTCAAGGCACTC	TTATTGCCCCTTGAGACACC
SlMLO12	230 bp	AGACACCTGAGGCTGGAGAA	TTCCCACTCCTTCCACTCAC
SlMLO13	83 bp	CTTGTGGGGATTGAAGCAAC	GCACAAGAACCCCTGAAATC
SlMLO14	84 bp	CTCTGCGCAAGGGTTTTATC	CTTCTTCCATCGAGCGAATC
SlMLO15	128 bp	TAACTCTCCGGCATGGATTC	CCAAATCCATGGGCTAATTG
SlMLO16	119bp	GAGCTGGCCTTCTTTGTTTG	CACTTCTGTGTGCCAATGCT
RNAi::SlMLO1	216 bp	CACCCGTATCTTTGGGTGCCATTT	CAGGGCGATTAAACCAGAAA
RNAi::SlMLO3	225 bp	CACCAGAGTAGCAACAGCATTGAAGAAT	GTTGTCCCAATGATCAAAATC
RNAi::SlMLO5	226 bp	CACCATACGGACATGGCATCAGAC	CATGACTTCCATCATCAAAGATT
RNAi::SlMLO8	219 bp	CACCCATTGAGAAAATGGCACCATAG	ATGGTGAGCCCTCAATATCC
